# A monograph of the Australopacific Saprininae (Coleoptera, Histeridae)

**DOI:** 10.3897/zookeys.689.12021

**Published:** 2017-08-14

**Authors:** Tomáš Lackner, Richard A.B. Leschen

**Affiliations:** 1 Bavarian State Collection of Zoology, Münchhausenstraße 21, 81247 Munich, Germany; 2 Landcare Research, New Zealand Arthropod Collection, Private Bag 92170, Auckland, New Zealand

**Keywords:** Histeridae, Saprininae, Coleoptera, Australopacific Region, monograph

## Abstract

The Australopacific Saprininae, containing twelve genera and forty species, are reviewed, illustrated and keyed to genera and species. Two new genera, *Australopachylopus* **gen. n.** (New Zealand, type species *Saprinus lepidulus* Broun, 1881) and *Iridoprinus* **gen. n.** (Australia, type species *I. myrmecophilus* **sp. n.**) and four new species: Saprinus (Saprinus) rarus **sp. n.** (Australia), Saprinus (Saprinus) chathamensis **sp. n.** (Chatham Islands, New Zealand), Saprinus (Saprinus) pseudodetritus **sp. n.** (Chatham Islands, New Zealand) and Saprinus (Saprinus) pacificus **sp. n.** (Kiribati) are described. The Saprininae fauna of the Australopacific Region is a mixture of northern invaders that most likely arrived to the region in early Cenozoic by ‘island hopping’ from north (*Hypocaccus*, *Hypocacculus*, several *Saprinus*) and truly autochthonous taxa either with uncertain phylogenetic affinities (*Iridoprinus* **gen. n.**, *Saprinodes* Lewis, 1891, *Reichardtia* Wenzel, 1944, *Australopachylopus* **gen. n.**), primitive Australopacific endemics (e.g. *Tomogenius* Marseul, 1862) or presumed relicts (several species of *Saprinus* Erichson, 1834). Several Saprininae taxa (*Chalcionellus aeneovirens* (Schmidt, 1890); (*Gnathoncus rotundatus* (Kugelann, 1792); *G. communis* (Marseul, 1862); Euspilotus (Neosaprinus) rubriculus (Marseul, 1855); Hypocaccus (Nessus) interpunctatus interpunctatus (Schmidt, 1885); Saprinus (S.) chalcites (Illiger, 1807) and Saprinus (S.) cupreus Erichson, 1834)) were introduced into the region with human activity. We report the first cases of myrmecophily (*Iridoprinus myrmecophilus* **gen. et sp. n.**) and termitophily (*Saprinus rarus* **sp. n.**) in the Saprininae from the Australopacific Region. Lectotypes and paralectotypes of the following taxa are designated herein: *Saprinus amethystinus* Lewis, 1900, *Saprinus apricarius* Erichson, 1834, *Saprinus artensis* Marseul, 1862, *Saprinus auricollis* Marseul, 1855, *Saprinus australasiae* Blackburn, 1903, *Saprinus bistrigifrons* Marseul, 1855, *Saprinus certus* Lewis, 1888, *Saprinus communis* Marseul, 1862, *Saprinus cupreus* Erichson, 1834, *Saprinus cyanellus* Marseul, 1855, *Hister cyaneus* Fabricius, 1775, *Saprinus dentipes* Marseul, 1855, *Saprinus desbordesi* Auzat, 1916, *Saprinus gayndahensis* MacLeay, 1871, *Saprinus hyla* Marseul, 1864, *Saprinus incisisternus* Marseul, 1862, *Saprinus incisus* Erichson, 1842, *Saprinus irinus* Marseul, 1862, *Saprinus laetus* Erichson, 1834, *Saprinus lepidulus* Broun, 1881, *Saprinus mastersii* MacLeay, 1871, *Saprinus nitiduloides* Fairmaire, 1883, *Saprinus pedator* Sharp, 1876, *Saprinus pseudocyaneus* White, 1846, *Saprinus rubriculus* Marseul, 1855, *Saprinus sinae* Marseul, 1862, *Saprinus tasmanicus* Marseul, 1855, *Saprinus tyrrhenus* Blackburn, 1903, *Saprinus varians* Schmidt, 1890, *Saprinus vernulus* Blackburn, 1903, *Saprinus viridanus* Lewis, 1899, *Saprinus viridipennis* Lewis, 1901, and *Saprinus westraliensis* Blackburn, 1903. The synonymy of *Saprinus tyrrhenus* Blackburn, 1903 is revoked and the species is considered as valid (**stat. n.**). Seven new synonymies are proposed: *Saprinus gayndahensis* MacLeay, 1871 = *Saprinus laetus* Erichson, 1834 **syn. n.**, *Saprinus pseudocyaneus* White, 1846 = *Saprinus laetus* Erichson, 1834 **syn. n.**, *Saprinus mastersii* MacLeay, 1871 = *Saprinus laetus* Erichson, 1834 **syn. n.**, *Saprinus dentipes* Marseul, 1855 = Hypocaccus (Baeckmanniolus) gaudens (J.L. LeConte, 1851) **syn. n.**, Hypocaccus (Hypocaccus) vernulus (Blackburn, 1903) = Hypocaccus (Hypocaccus) sinae (Marseul, 1862) **syn. n.**, Saprinus (Saprinus) lindrothi Dahlgren, 1968 = Saprinus (Saprinus) prasinus Erichson, 1834 **syn. n.**, and Saprinus (Saprinus) certus Lewis, 1888 = Saprinus (Saprinus) frontistrius Marseul, 1855 **syn. n.** The following new records are: Euspilotus (Neosaprinus) rubriculus (Marseul, 1855) (= *Saprinus gnathoncoides* Bickhardt, 1909) (Australia), Saprinus (Saprinus) laetus Erichson, 1834 (Lord Howe Island) and Saprinus (Saprinus) cyaneus cyaneus (Fabricius, 1775) (Lord Howe Island and Fiji).

## Introduction

“*These structures will be figured by me in a future revision of the Saprininae of the Australian Region*” – R. Wenzel (1955).

The Saprininae of the Australopacific Region (we use the term “Australopacific” here which corresponds with the Australian and Pacific Regions of Löbl and Löbl 2015; see also Material and methods below) are poorly known. Apart from Dahlgren’s study (1976) on the New Zealand *Tomogenius* Marseul, 1862 and Dégallier’s (1993) revision of the endemic Australian genus *Saprinodes* Lewis, 1891, no genus from the Australopacific Region has been systematically revised with all species covered. Most Saprininae species from the Australopacific Region were described by Marseul (1855, 1862, 1864, 1870) and Lewis (1891, 1899, 1900, 1901); additional species were described by Fabricius (1775), Boisduval (1835), Erichson (1834, 1842), White (1846), Sharp (1876), Fairmaire (1883), Dahlgren (1968), Kanaar (1989), Mazur (1990), Dégallier (1993) and Gomy (2007). Australian Saprininae have been studied by Blackburn (1903) and MacLeay (1871), who both described several species, most of which have been discovered to be junior synonyms of previously described species. The New Zealand beetle fauna was described by Broun (1880, 1881, 1886, 1914 and 1921) who described two saprinine species: *Saprinus* (=*Tomogenius*) *latipes* Broun, 1881 and *Saprinus* (=*Australopachylopus*) *lepidulus* Broun, 1881; Dahlgren (1976) described two species of *Tomogenius*. The histerid fauna of New Caledonia was revised by Wenzel (1955), who mentioned three species of *Saprinus* as present on the island, although he doubted that the record of the Palaearctic species *Saprinus acuminatus* (Fabricius, 1798) (=*subnitidus* Marseul, 1855) was correct; his intention to revise the group (see above quote) never eventuated.

The native Saprininae of the Australopacific Region are rather poorly diverse compared to other regions. In total there are only nine native and three introduced genera totaling 40 known species, of which seven are introduced into the area. When we break down the subfamily’s fauna to the major islands and Australia (termed here as ‘areas’; Fig. 751, see below), we can see that most of the species are found in Australia (19 native and 6 introduced species), followed by New Zealand (8 native and 3 introduced species), New Guinea (7 native and 0 introduced species), New Caledonia (2 native and 0 introduced species) and the Pacific Region of Löbl and Löbl (2015) (1 native and 0 introduced species). The Saprininae fauna of other regions is substantially richer (see Lackner 2014d). The reason for this paucity in species could be attributed to the long-standing isolation of the Australian continent in combination with the (originally) densely forested large islands like New Zealand and New Guinea. Saprininae are predators of flies and other arthropods and prefer open arid landscapes over wetter forested areas. On the other hand, the Australopacific Region harbors several species with very interesting morphologies and biologies; see Discussion of the genera *Iridoprinus* gen. n., *Saprinodes*, *Saprinus* and *Tomogenius* for details.

This work presents another contribution to on-going revisionary work on the genera of the subfamily Saprininae by the first author (Lackner 2009a, b, c, 2010, 2011a, b; Lackner 2012; Lackner 2013a,b,c; Lackner 2014a,b,c,d; Lackner 2015; Lackner 2016a,b; Tishechkin and Lackner 2012; Lackner and Gomy 2013; Lackner and Tishechkin 2014; Lackner and Gomy 2016; Lackner 2017).

## Material and methods

The taxa in the present study were based on the Saprininae catalogued in Mazur (2011), but restricted to Australian and Pacific Regions *sensu* Löbl and Löbl (2015), which include the following sub-regions termed here as ‘areas’ (Fig. 751): 1) the island of New Guinea together with all surrounding islands (but without Moluccas), Aru Islands, New Britain, New Ireland as well as the Solomon Islands; 2) the island of New Caledonia, together with Vanuatu, Fiji, Tuvalu, Wallis and Futuna, American Samoa, Tokelau, Tonga, The Cook Islands, French Polynesia and Niue; 3) Australia with Lord Howe Island, Tasmania with surrounding islands, Kangaroo Island, Tiwi Islands and numerous small islands around Australia’s coasts; 4) New Zealand with the Chatham Islands and other tiny surrounding islands as well as subantarctic islands; 5) the Pacific Region of Löbl and Löbl (2015), which is in this paper represented by a single new species discovered in Kiribati.

Taxa are presented alphabetically, because phylogenetic information is lacking for most taxa covered here (*sensu* Lackner 2014d). Diagnoses and descriptions of many Australopacific representatives, except for a few exceptions, were based on numerous examined specimens. The work is structured as follows: 1) only native, mainly endemic and newly-described taxa are provided with full (re)descriptions; 2) in case of externally highly similar species (e.g. *Saprinus detritus* and *S. pseudodetritus*) usually only the earlier-described species is provided with a full re-description; the other species is provided with diagnostic description outlining the differences between the taxa only; 3) non-native taxa are provided only with diagnoses and comments on their morphology and/or references to where their full (re)descriptions can be found; 4) for the sake of better species recognition, all taxa are provided with habitus photographs, SEM micrographs as well as illustrations of male terminalia; 5) taxa are provided only with synonymies of the names that have been applied in the region; 6) phylogenetic information is given mainly for the authochthonous taxa that were included in the previously published analysis by the senior author (Lackner 2014d). For the full synonymies and literature references the reader is referred to the catalogues of Mazur (1984, 1997 and 2011).

All dry-mounted specimens were relaxed in warm water for several hours or overnight, depending on the body size. After removal from original cards, the beetles were side-mounted on triangular mounting cards and observed under a Nikon 102 stereoscopic microscope with diffused light. Some structures were studied using methods described by Ôhara (1994): the head and male genitalia were macerated in a hot 10% KOH solution for about 15 minutes, cleared in 80% alcohol, macerated further in lactic acid with fuchsine, incubated at 60°C for two hours, and subsequently transferred into a mixture of glacial acetic acid 1 part and methyl salicylate 1 part heated at 60°C for 15 minutes and cleared in xylene. Specimens were then observed in α-terpineol in a small glass dish. The mentum, labium, labrum, mandibles and antennae were disarticulated. Digital photographs of the male terminalia, mouthparts and antenna were taken by a Nikon 4500 Coolpix camera and edited in Adobe Photoshop CS4. Structures were drawn using a Hakuba klv-7000 light-box. SEM photographs were taken with a JSM 6301F microscope at the laboratory of Faculty of Agriculture, Hokkaido University, Sapporo, Japan, and and habitus illustrations were automontaged by Birgit Rhode at Landcare Research. Measurements were made with an ocular micrometer. Morphological terminology follows that of Ôhara (1994), Bousquet and Laplante (2006), Lackner (2010) and Lawrence et al. (2011). Separate lines of the same label of the type specimens are marked by slash (/); spec. = unsexed specimen. Material examined for New Zealand taxa is listed according to their Crosby codes (Crosby et al. 1998). Distributional maps were created using Free Vector Maps (www.freevectormaps.com). Whenever the locality label was too vague (e.g. ‘Queensland’) no concrete locality could be decided and therefore such information is not depicted on the distributional maps. Also, localities that we were not able to locate using Google Earth software or the Internet were dropped from the distributional maps. The following acronyms of museums and private collections are used throughout the text:

**AMNZ** Auckland Institute and Museum, Auckland, New Zealand (J. Early);

**AMS** Australian Museum, New South Wales, Sydney, Australia (D. Smith);

**ANIC** Australian National Insect Collection, Canberra, Australia (T. Weir);

**BMNH** The Natural History Museum, London, United Kingdom (M. Barclay);

**BPBM** Bernice P. Bishop Museum, Honolulu, Hawaii, USA (N. Evenhuis);

**CJN** Private collection of John Nunn (Christchurch, New Zealand);

**CPK** Private collection of Pete Kovarik (Columbus, USA);

**CYG** Private collection of Yves Gomy (Nevers, France; currently housed in ZSM);

**HNHM** Hungarian Natural History Museum, Budapest, Hungary (O. Merkl);

**LUNZ** Lincoln University Collection, Canterbury, New Zealand (J. Marris);

**MAMU** University of Sydney, MacLeay Museum, Sydney, Australia (D. Smith);

**MNHN** Muséum National d’Histoire Naturelle, Paris, France (Th. Deuve & A. Taghavian);

**NCB** Naturalis, Leiden, The Netherlands (H. Huijbregts);

**NHRS** Naturhistoriska Riksmuseet, Stockholm, Sweden (J. Bergsten);

**NMPC** National Museum Prague Collection, Prague, Czech Republic (J. Hájek);

**NZAC** New Zealand Arthropod Collection, Landcare Research, Auckland, New Zealand (R. Leschen);

**OUMNH** Oxford Museum of Natural History, Oxford, United Kingdom (D. Mann);

**QM** Queensland Museum, South Brisbane, Australia (G. Monteith);

**SAMA** South Australian Museum, Adelaide, Australia (P. Hudson);

**SMF** Senckerberg Museum, Frankfurt am Main, Germany (A. Allspach);

**TLAN** T. Lackner’s private collection, temporarily housed at ZSM;

**UUZM** Uppsala University Collection, Uppsala, Sweden (H. Mejlon);

**ZMHUB** Museum für Naturkunde, Leibnitz Geselschaft, Berlin, Germany (B. Jaeger);

**ZMUC** Zoological Museum, University of Copenhagen, Denmark (A. Solodovnikov);

**ZSM** Zoologische Staatssammlung, München, Germany (M. Balke).

**Abbreviations.** Abbreviations of morphological measurements follow Ôhara (1994) and are used throughout the text as follows:

**APW** width between anterior angles of pronotum

**EL** length of elytron along elytral suture

**EW** maximum width between outer margins of elytra

**PEL** length between anterior angles of pronotum and apices of elytra

**PPW** width between posterior angles of pronotum.

## Taxonomy

### Key to the genera of the Saprininae of the Australopacific Region

**Table:** 

1(8)	Frontal and supra-orbital striae completely absent (Fig. 241)
2(3)	Both sets of prosternal striae absent (Fig. 245); prosternal process with sparse setae; clypeus strongly swollen; outer margin of protibia with a row of more than 20 denticles (Fig. 249); meso- and metafemora strongly swollen; pygidium almost impunctate	***Reichardtia* Wenzel, 1944** (New Zealand)
3(2)	Both sets of prosternal striae present (Fig. 51); prosternal process asetose; clypeus not swollen, flattened; protibia with approximately four moderately large to low teeth topped by tiny denticle, followed by several minute denticles (Fig. 55); meso- and metafemora not swollen.
4(5)	Carinal prosternal striae joined anteriorly by deep sulcus (Fig. 51); elytral disc between fourth dorsal elytral and sutural elytral striae without a hooked appendix (Fig. 48); marginal epipleural stria	**Euspilotus (Neosaprinus) rubriculus (Marseul, 1855)** (adventive to Australia and New Zealand)
5(4)	Carinal prosternal striae not joined anteriorly by deep sulcus (Fig. 72); elytral disc between fourth dorsal elytral and sutural elytral striae with a characteristic short hooked appendix (Fig. 90); marginal epipleural stria double; prosternal process apically with two deep median foveae or with single (tiny) median fovea (absent in *Gnathoncus communis*).
6(7)	Prosternal process apically with two large median foveae separated by the apex of prosternal process (Fig. 642)	***Tomogenius* Marseul, 1862** (Australia, New Guinea and New Zealand)
7(6)	Prosternal process apically with a single tiny median fovea (Fig. 94; in *G. communis* fovea absent), never with two large median foveae	***Gnathoncus* Jacquelin du Val, 1857** (adventive to Australia and New Zealand, possibly introduced also elsewhere in Oceania).
8(1)	Supraorbital striae always present, frontal stria also often present (Fig. 27), although occasionally interrupted medially and in some cases even prolonged onto the clypeus.
9(12)	Both sets of prosternal striae absent (Fig. 8), occasionally vestiges of carinal prosternal striae present between procoxae.
10(11)	Prosternal process widely depressed laterally, setose (Fig. 8); mesoventrite setose (Fig. 9)	***Australopachylopus* gen. n.** (New Zealand)
11(10)	Prosternal process keel-like, not depressed laterally, convex, asetose (Fig. 224); disc of mesoventrite asetose	***Notosaprinus* Kryzhanovskij, 1972** (Australia)
12(9)	At least one pair of the prosternal striae developed, more often both sets of the prosternal striae present (Fig. 206).
13(14)	Sutural elytral and inner subhumeral striae completely absent; elytra with five strongly carinate dorsal elytral striae (Fig. 202); deep longitudinal groove (Fig. 208) present on the metepisternum for the accommodation of mesotarsus	***Iridoprinus* gen. n.** (Australia)
14(13)	Sutural elytral stria always present, sometimes shortened basally; elytral disc with up to four dorsal elytral striae, striae never strongly carinate; metepisternum without deep longitudinal groove for reposing mesotarsus (Fig. 286); inner subhumeral stria normally also present.
15(18)	Prosternal foveae absent (Fig. 284).
16(17)	Protibia devoid of teeth or denticles, apically narrowed, terminating in a massive protibial tooth (Fig. 269); labral pits and setae absent	***Saprinodes* Lewis, 1891** (Australia)
17(16)	Protibia with teeth topped by denticles or with denticles only (Fig. 308), not narrowed apically, not terminating in a massive protibial tooth; labral pits and setae present	***Saprinus* Erichson, 1834** (entire Australopacific Region)
18(15)	Prosternal foveae present (Fig. 32).
19(20)	Pronotal depressions present; frontal stria not carinate, somewhat weakened medially (Fig. 27); prosternal foveae (Fig. 32) large and deep, anteriorly connected by marginal prosternal stria	***Chalcionellus aeneovirens* (Schmidt, 1890)** (introduced to to Western Australia, Victoria and Queensland)
20(19)	Pronotal depressions absent; frontal stria often carinate; prosternal foveae (Fig. 114) anteriorly not connected by marginal prosternal stria.
21(22)	Frons (Fig. 113) with punctation, without elongate rugae..........	***Hypocacculus*** (one native species known from New Guinea)
22(21)	Frons (Fig. 145) with elongate rugae; punctures almost never present	***Hypocaccus* C. Thomson, 1867** (Australia, New Guinea)

#### Australopachylopus gen. n.

Taxon classificationAnimaliaColeopteraHisteridae

http://zoobank.org/CF8C725A-72E0-4792-8C4C-313C3095134A

Figs 1 , 2–9 , 10–13 , 14–16 , 17–25 , 752

##### Type species.

*Saprinus lepidulus* Broun, 1881: 665.

##### Diagnosis.

Cuticle dark brown to black with faint metallic luster; pronotum almost glabrous, only with faint scattered lateral punctation; elytra punctate and striate; frontal stria weakened, occasionally absent; pronotal hypomeron, prosternum, disc of mesoventrite, lateral disc of metaventrite, metepisternum + fused metepimeron and lateral sides of all abdominal ventrites setose; pronotal depressions present; prosternal foveae absent; prosternal apophysis strongly constricted between procoxae, prosternal process thence strongly expanded; carinal prosternal striae present as vague rudiments on prosternal apophysis; lateral prosternal striae absent; meso- and metafemora thickened, with rows of setigerous punctures. Eighth sternite of the male genitalia fused medially, apices with a row of sparse setae. Eighth tergite densely covered in pores and pseudopores. Spiculum gastrale dilated on both ends. Aedeagus narrow, parameres fused on their basal two-thirds. The densely setose venter in combination with coarsely punctate elytra will readily distinguish this New Zealand endemic from all other Saprininae present in the country.

##### Biology.

A psammophilous taxon, found usually in carcasses or under coastal wrack. Several specimens were collected from pitfall traps.

##### Distribution.

*Australopachylopus* is endemic to New Zealand and is found on both North and South Islands, but has not been recorded from the Chatham Islands so far (Fig. 752).

##### Etymology.

Generic epithet of this new genus has been created combining the Latin word for south ‘austral’ and generic name *Pachylopus*.

##### Remarks.

Six species are included in the genus *Neopachylopus* Reichardt, 1926 (Mazur 2011: 211). In the published phylogenetic analysis of Saprininae by the first author (Lackner 2014d) *Neopachylopus lepidulus* falls separately from the other two members of the genus (*N. sulcifrons* (Mannerheim, 1843) and *N. kochi* Thérond, 1963)) which were likewise included in the analysis in order to test the monophyly of the genus. All three taxa (*N. lepidulus*, *N. sulcifrons* and *N. kochi*) were recovered inside a large polytomy of global, mostly psammophilous species united by one ‘strong’ and three ‘weak’ synapomorphies. *N. lepidulus*, in fact, comes out as sister to the clade uniting another New Zealand endemic *Reichardtia* Wenzel and *Reichardtiolus pavlovskii* Kryzhanovskij, 1959; although this purported monophyletic group is not strongly supported (see more in the discussion of *Reichardtia*). It is interesting to note, however, that the male genitalia of both *Reichardtia* and *Australopachylopus* share several similarities, e.g. overall gestalt of the eighth sternite and tergite (including the numerous pores and pseudopores; compare Figs 17–19 and 256–258), a tuft of short setae situated on the apices of eighth sternite, and, very similarly shaped aedeagus (observed from the lateral view; compare Figs 25 and 262). The on-going molecular studies by the senior author will hopefully shed more light on the relationships between New Zealand Saprininae, since all higher taxa have been included. We place *N. lepidulus* into a new genus based on the setose underside and prosternal apophysis strongly constricted between procoxae, strongly expanded prosternal process; carinal prosternal striae present as vague rudiments on prosternal apophysis; and absent lateral prosternal striae. All of these characters are different from the type species of *Neopachylopus* (*N. sulcifrons*).

#### Australopachylopus lepidulus

Taxon classificationAnimaliaColeopteraHisteridae

(Broun, 1881) comb. n.

Figs 1 , 2–9 , 10–13 , 14–16 , 17–25 , 752

Saprinus lepidulus Broun, 1881: 665.

##### Type locality.

New Zealand: Wellington: Lyall Bay.

##### Type material examined.

*Saprinus lepidulus* Broun, 1881: Lectotype, present designation: unsexed specimen, with the following labels: “Type” (round, red-margined printed label); followed by: “New Zealand / Broun Coll. / Brit. Mus. / 1922-482” (printed); followed by: “Lyall Bay / Wellington” (written); followed by: “Pachylopus / lepidulus” (written); followed by: “Saprinus lepidulus / Broun, 1881 / LECTOTYPE 2014 / Des. Lackner & Leschen” (red label, written) (BMNH). Because the number of specimens in the original description is not given this specimen is selected as the lectotype. There are another two specimens in Broun’s collection, both of unknown sex: one bearing the following labels: “1169” (written); followed by “New Zealand / Broun Coll. / Brit. Mus. / 1922-482” (printed). Another one bears identical labels as the preceding specimen, but has one more label: “Invercargill” (printed) (BMNH). These specimens do not belong to the type series of Broun (1881) because Broun (1881) provides the type locality as Lyall Bay, and the second specimen lacks locality data.

##### Additional material examined.

NEW ZEALAND: North Island. ND: 4 specs., Ruakaka Beach, north Auckland, 22.ix.1932 (BMNH); 1 spec., Kai Iwi, 28.i.[19]15, Sand in Sun (NZAC); 2 specs., Himatangi, 9.ii.[19]58, R.A. Cumber leg. (NZAC); 1 spec., Kawerua Beach, 5.ii.1975, K. Hoson leg. (LUNZ). WO: 1 spec., Waikato Heads, under *Muehlenbeckia*, 1.i.1959, B.M. May leg. (NZAC); 1 spec., Kawhia Harbor, 27.i.1958, B.M. May leg., under dead fish on beach (NZAC); 1 ♂, 2 ♀♀, 4 specs., Raglan, 19.ix.1981, C.F. Butcher leg., under dead bird, fish (NZAC). HB: 1 spec., Porangahau, 29.i.1991, C. Duffy leg., pitfall traps (NZAC); SC: 1 spec., Temuka Beach, 22.iii.2008, on dead seagull (CJN). RI: 1 spec., Foxton Beach, 19.v.1996, in dead dry puffer fish on sandy beach (CJN). WN: 3 specs., Paekarariki Beach, 4.xii.1941, G.V. Hudson (BMNH); 1 spec., Wellington (BMNH); 2 specs., Wellington, J.J. Walker (BMNH). South Island. NN: 8 specs., Westport, J.J. Walker (BMNH); 1 spec., Westport, Beach Nelson, 15.x.1971, J.C. Watt leg., under logs (LUNZ); 1 spec., Farewell Spit, Outer Beach, 9.ii.1981, J.W. Early leg., carcass of dolphin (LUNZ); 1 ♂ & 4 specs., Wharariki Beach, 7.ii.1981, J.W. Early leg., under carcass on sand (LUNZ). MC: 2 specs., Lyttelton, xii.1901, J.J. Walker (BMNH). WD: 8 specs., Okarito lagoon, 7.–9.xii.2012, beach (under dead *Larus*), 43°13.5'S, 170°09.6'E, 1 m, M. Fikáček, J. Hájek & R. Leschen leg. (NMPC; 1 ex. in coll. TLAN); 1 ♀, Mananui Beach, 17.i.1982, J.W. Early leg., sandy beach (LUNZ). DN: 1 ♀, Kuri Bush, nr. Talieri Mouth, 18.iii.2001, on sandy beach (CJN). Unknown localities: 1 spec., New Zealand, 1162, Broun leg. (NZAC); 1 spec., New Zealand, without further data, J.J. Walker (BMNH); 1 spec., New Zealand, without further data, Broun, 5.ix.[18]89 (BMNH); 1 spec., New Zealand, no further data, vi.1904 (BMNH); 1 spec., New Zealand, no further data (BMNH).

##### Re-description.

**Figure 1.**
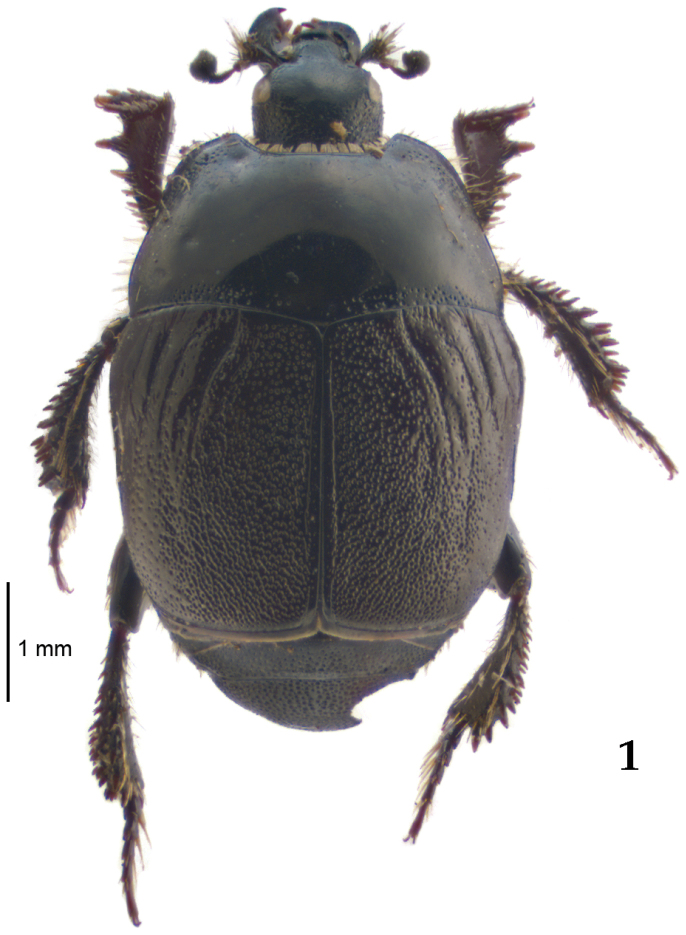
*Australopachylopus lepidulus* (Broun, 1881) comb. n., habitus, dorsal view.

Body length: PEL: 4.10–5.50 mm; APW: 1.10–1.75 mm; PPW: 3.15–4.00 mm; EL: 2.65–3.50 mm; EW: 3.50–4.50 mm. Body (Fig. 1) rectangular oval, dorsal surface convex, ventral surface flattened, cuticle black with slight metallic luster, legs, mouthparts and antennae dark brown, antennal club blackish.

**Figures 2–9.**
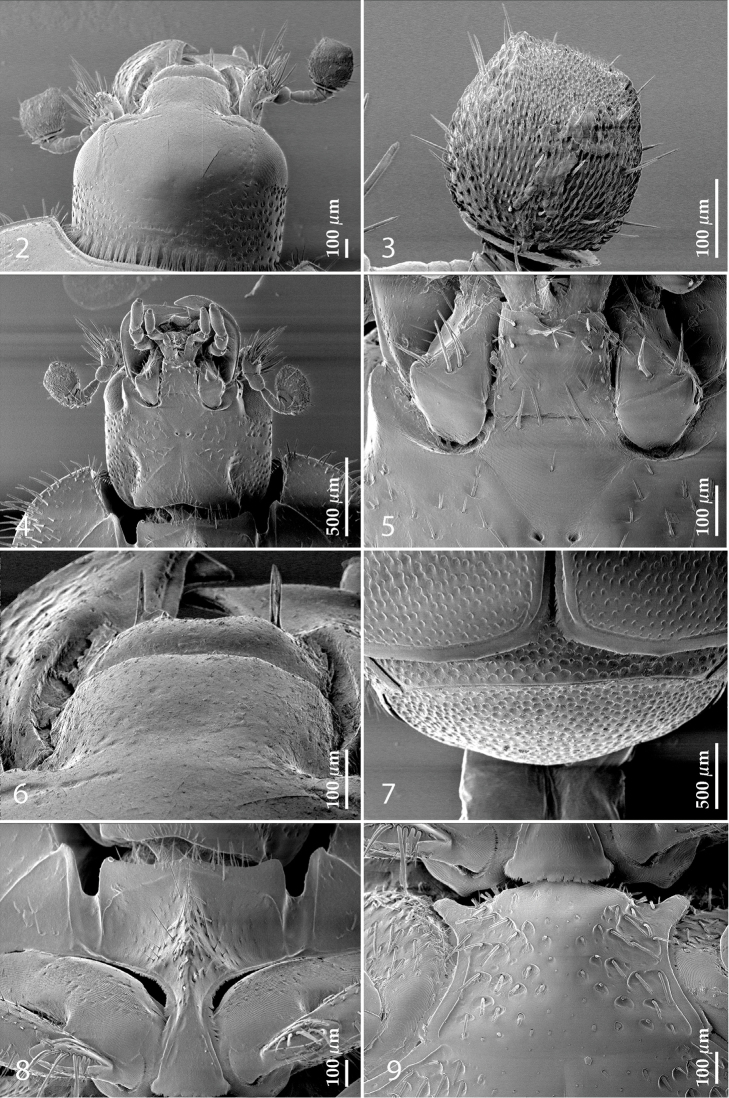
**2** *Australopachylopus lepidulus* (Broun, 1881) comb. n., head, dorsal view **3** antennal club, dorsal view **4** head, ventral view **5** mentum, ventral view **6** labrum + clypeus, dorsal view **7** propygidium + pygidium **8** prosternum **9** mesoventrite.

Antennal scape (Fig. 2) with carinate margins, with numerous long setae; antennal club (Fig. 3) round, slightly depressed dorso-ventrally; without visible articulation, entire surface with thick short yellow sensilla intermingled with sparse longer erect sensilla, apically with a flat sensory area with short dense sensilla; sensory structures of antennal club (Fig. 16) in a form of a single pear-shaped vesicle situated under sensory area on inner-ventral side of club.

Mandibles stout, thickened (Fig. 14) with rounded outer margin strongly curved inwardly, dorsally with sparse punctures, acutely pointed, sub-apical tooth on inner margin of left mandible large, blunt; labrum (Figs 6, 15) punctate, dorsally convex with slight median depression; labral fold small, setae of lateral fringe moderately long; two labral setae present; terminal labial palpomere (Fig. 4) elongated, its width about one-third its length; palpal organ present on both labial and maxillary palpi; mentum (Fig. 5) square-shaped, anterior angles not produced, anterior margin with deep median excavation, surface around it with four longer setae; lateral margins with double row of shorter ramose setae; disc of mentum imbricate, with several short scattered setae; cardo of maxilla with few short setae on lateral margin, stipes triangular, with three short setae; lacinia without lacinial hook (=uncus); terminal maxillary palpomere elongated, its width about one-third its length, approximately twice as long as penultimate.

**Figures 10–13.**
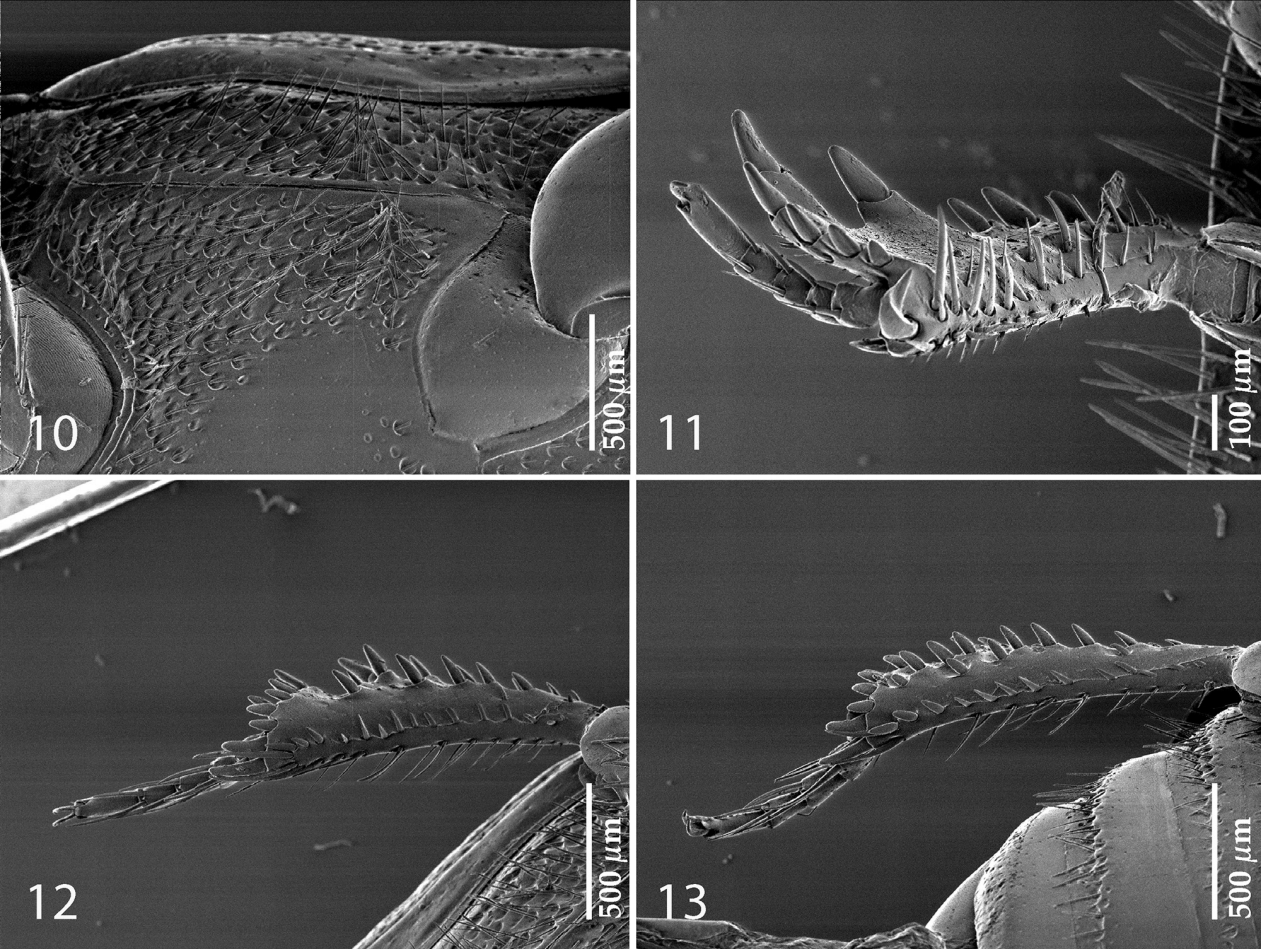
**10** *Australopachylopus lepidulus* (Broun, 1881) comb. n., lateral disc of metaventrite + metepisternum **11** protibia, ventral view **12** mesotibia, ventral view **13** metatibia, ventral view.

Clypeus (Fig. 6) sub-quadrate, rounded laterally, slightly concave medially, with scattered microscopic punctures; frontal stria largely interrupted anteriorly (at times completely absent), continuous with weakly carinate supraorbital stria; frontal disc (Fig. 2) with irregular scattered fine punctation; eyes convex, visible from above.

Pronotal sides (Fig. 1) bisinuate, moderately convergent anteriorly, apical angles acute; pronotal depressions well-impressed (occasionally shallow); marginal pronotal stria complete, carinate; disc of pronotum laterally with a band of coarse and dense punctures (occasionally completely smooth), a band of similar punctation present also along pronotal base, pronotal disc otherwise glabrous; pronotal hypomeron with long yellow setae; scutellum small, visible.

Elytral humeri prominent; elytral epipleura smooth; marginal epipleural stria complete; marginal elytral stria straight and carinate, continued as well impressed complete apical elytral stria continuous with sutural elytral stria; along elytral marginal stria a regular row of round punctures present. Humeral elytral stria well impressed on basal fifth (occasionally longer); inner subhumeral stria present medially, rather long, almost attaining marginal elytral stria apically; elytral disc with four deeply impressed dorsal elytral striae 1–4, about the same length, not reaching elytral half apically (occasionally third and fourth dorsal elytral striae intermittent or obliterated by coarse punctation); fourth elytral stria basally not connected with sutural elytral stria; sutural elytral stria well impressed, abbreviated on basal tenth and apical sixth (occasionally continuous with apical elytral stria), interspace between it and elytral suture somewhat elevated, especially on apical third, on basal half a row of fine punctures present; punctation of elytral disc (with exception of elytral humeri and elytral flanks) coarse and dense, punctures separated by less than twice their diameter, on intervals between elytral striae punctation somewhat weakened.

**Figures 14–16.**
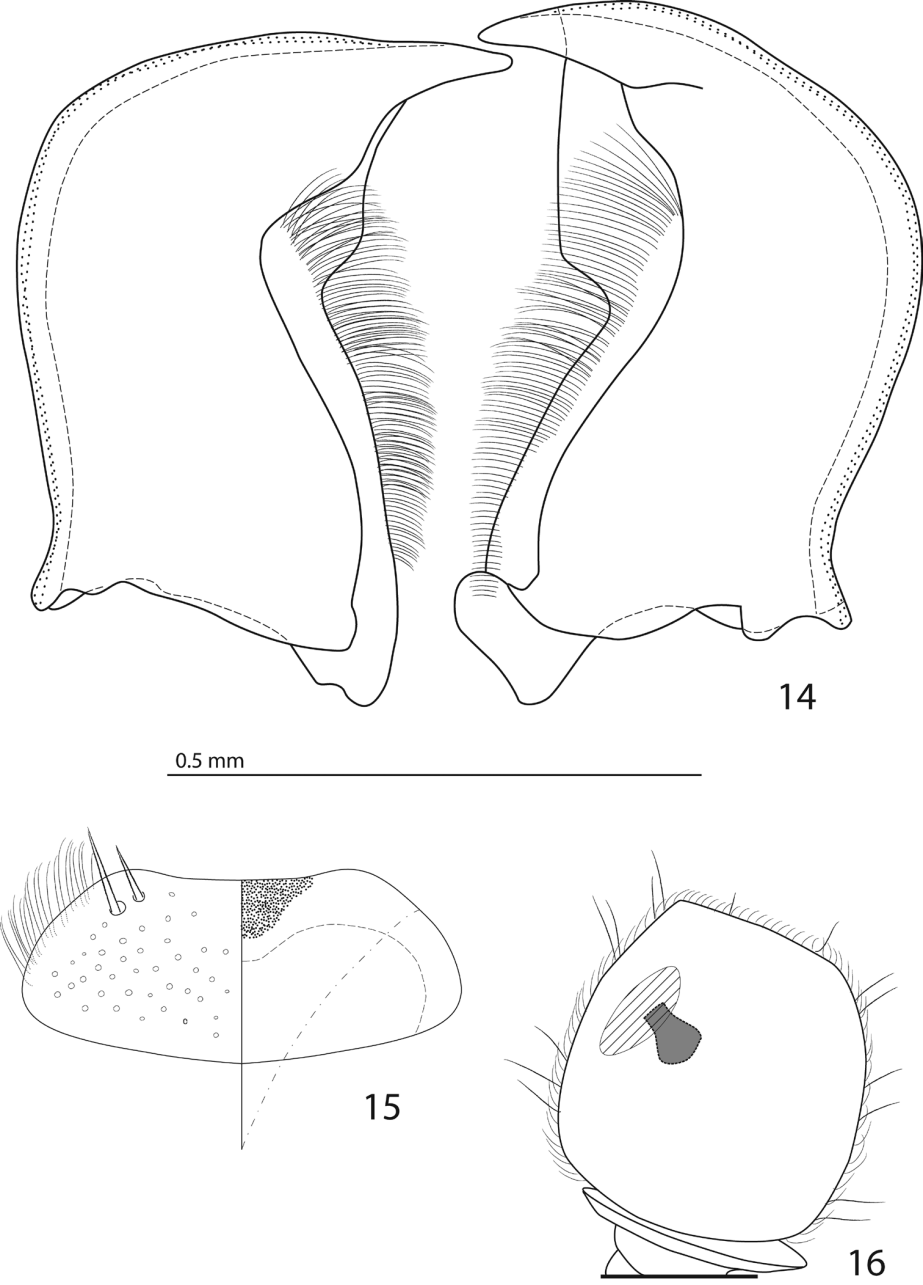
**14** *Australopachylopus lepidulus* (Broun, 1881) comb. n., mandibles, dorsal view **15** labrum: left half depicting dorsal view and right half depicting epipharynx **16** antennal club depicting sensory structures of the antenna.

Propygidium (Fig. 7) largely covered by elytra; its punctation similar to that of elytral disc; pygidium (Fig. 7) also densely and coarsely punctate, punctures becoming sparser towards extreme apex.

Anterior margin of median portion of prosternum (Fig. 8) slightly projected medially, setose; prosternal foveae absent; marginal prosternal stria vaguely impressed but complete, distanced from anterior margin; prosternal apophysis strongly constricted between procoxae, knife-like, prosternal process thence strongly widening and sloping down anteriorly, surface imbricate, dorso-medially with numerous setigerous punctures; occasionally vestiges of carinal prosternal striae present on prosternal apophysis; lateral prosternal striae absent.

Anterior margin of mesoventrite (Fig. 9) with slight median projection; discal marginal mesoventral stria anteriorly absent, laterally present; disc of mesoventrite convex, with deep and dense setigerous punctures of various sizes; meso-metaventral suture indistinct; meso-metaventral sutural stria absent; intercoxal disc of metaventrite smooth, in male with a longitudinal median depression; laterally with coarse round setigerous punctures; a narrow band of fine punctation present along posterior margin; lateral metaventral stria absent; lateral disc of metaventrite (Fig. 10) slightly excavate, with shallow dense setigerous punctures; metepisternum + fused metepimeron (Fig. 10) with even denser and coarser setigerous punctures, separated by less than half of their diameter; lateral metepisternal stria absent.

Intercoxal disc of first abdominal ventrite with shortened and vaguely impressed lateral stria; disc laterally and anteriorly with round deep punctures of various sizes, median part of disc impunctate, laterally punctures setigerous; all visible abdominal ventrites setose laterally.

Protibia (Fig. 11) slightly widening apically, outer margin with three large widely spaced distal teeth topped by large denticle, diminishing in size in proximal direction, followed by approximately six smaller stout proximal denticles, diminishing in size in proximal direction; setae of outer row thin, sparse; setae of median row approximate to inner protibial margin, but shorter than those of outer row; protarsal groove deep; anterior protibial stria vaguely impressed, shortened on posterior half; protibial spur large, hooked, growing out near tarsal insertion; outer part of posterior surface of protibia smooth, demarcation line between outer and median of posterior surface carinate; posterior protibial stria complete, with dense row of strongly sclerotized long setae growing in size and girth apically; inner margin with double row of short setae.

Mesotibia somewhat thickened, outer margin with a row of approximately 5 denticles growing in size apically, one more denticle present near apical margin; setae of outer row dense and long, strongly sclerotized, growing in size apically; setae of median row rather distanced from outer row, thinner and sparser; posterior mesotibial stria inconspicuous (absent?); anterior surface of mesotibia (Fig. 12) with another dense row of short denticles, becoming sparser and thinner towards inner mesotibial margin; anterior mesotibial stria undulate, with a row of regular short setae growing in size and girth apically; mesotibial spur large; apical margin of mesotibia with a row of about 5 stout denticles; first and second tarsomere ventrally with four long strongly sclerotized setae; third and fourth tarsomere with only two such setae; fifth tarsomere devoid of setae ventrally; dorsally first two tarsomeres with two strongly sclerotized setae; third, fourth and fifth tarsomere with only single seta dorsally; claws of apical tarsomere bent, approximately one-third its length; metatibia (Fig. 13) generally similar to mesotibia, but more slender; denticles of outer margin shorter and sparser; anterior surface of metatibia with three rows of short stout denticles.

**Figures 17–25.**
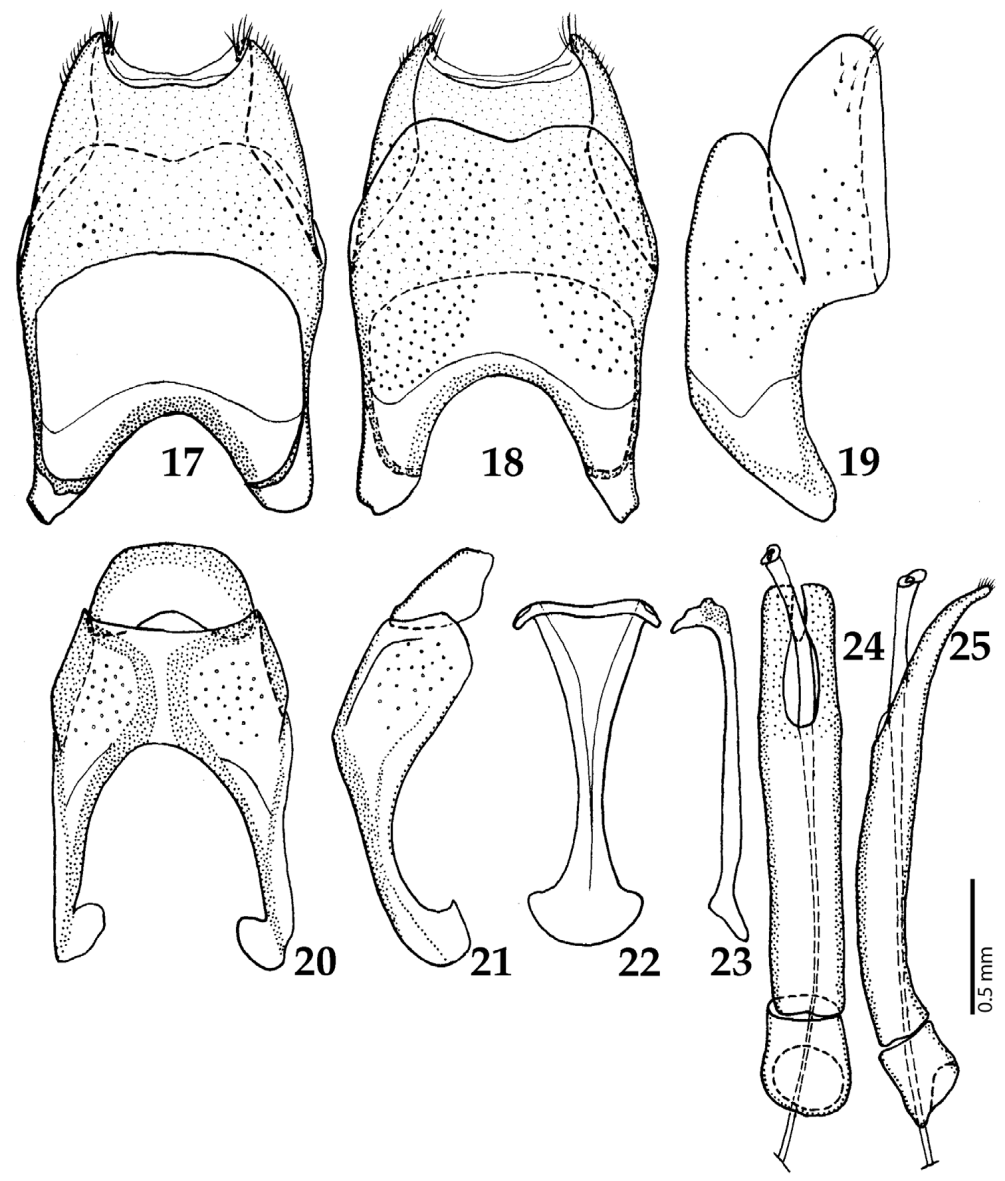
**17** *Australopachylopus lepidulus* (Broun, 1881) comb. n., male terminalia: 8^th^ sternite + 8^th^ tergite, ventral view **18** ditto, dorsal view **19** ditto, lateral view **20** male terminalia: 9^th^ + 10^th^ tergites, dorsal view **21** ditto, lateral view **22** male terminalia: spiculum gastrale, ventral view **23** ditto, lateral view **24** male terminalia: aedeagus, dorsal view **25** ditto, lateral view.

Male genitalia. Eighth sternite (Figs 17–18) fused longitudinally; vela short, apices of eighth sternite with a tuft of short setae; eighth sternite laterally with several even shorter setae (Figs 17–19); eighth tergite covered with pores and pseudopores, apical margin inwardly arcuate, basal margin strongly inwardly arcuate; eighth tergite and eighth sternite fused laterally (Fig. 19). Ninth tergite (Figs 20–21) longitudinally fused medially, surface medially with two strongly sclerotized lines; tenth tergite basally inwardly arcuate, apically outwardly arcuate; spiculum gastrale (Figs 22–23) gradually dilated in most of apical half, apex strongly sclerotized; basal end dilated, rounded. Aedeagus (Figs 24–25) slightly thickened, almost parallel-sided; basal piece of aedeagus short, ratio of its length : length of parameres 1 : 5; parameres of aedeagus fused almost along their basal two-thirds; aedeagus only slightly curved from lateral view, apex of aedeagus flattened dorso-ventrally, fringed with a tuft of very short setae (Fig. 25).

#### Chalcionellus

Taxon classificationAnimaliaColeopteraHisteridae

Reichardt, 1932

Figs 26 , 27–38 , 39–47 , 753

Chalcionellus Reichardt, 1932: 16. Type species Saprinus amoenus Erichson, 1834, original designation.

##### Diagnosis.

Diagnosis of this genus is based solely on the species *C. aeneovirens* that has been recorded from the Australopacific Region. Rather small, metallic ovoid beetle; elytra lighter than pronotum; frons with scattered fine punctures; frontal stria complete; pronotal depressions present, most of pronotal surface punctate, punctures most coarse in pronotal depressions. Dorsal elytral striae well developed, in punctures; prosternum with prosternal foveae, which are linked by marginal prosternal stria. Protibia with 7–8 moderately large teeth topped by denticle.

**Figure 26.**
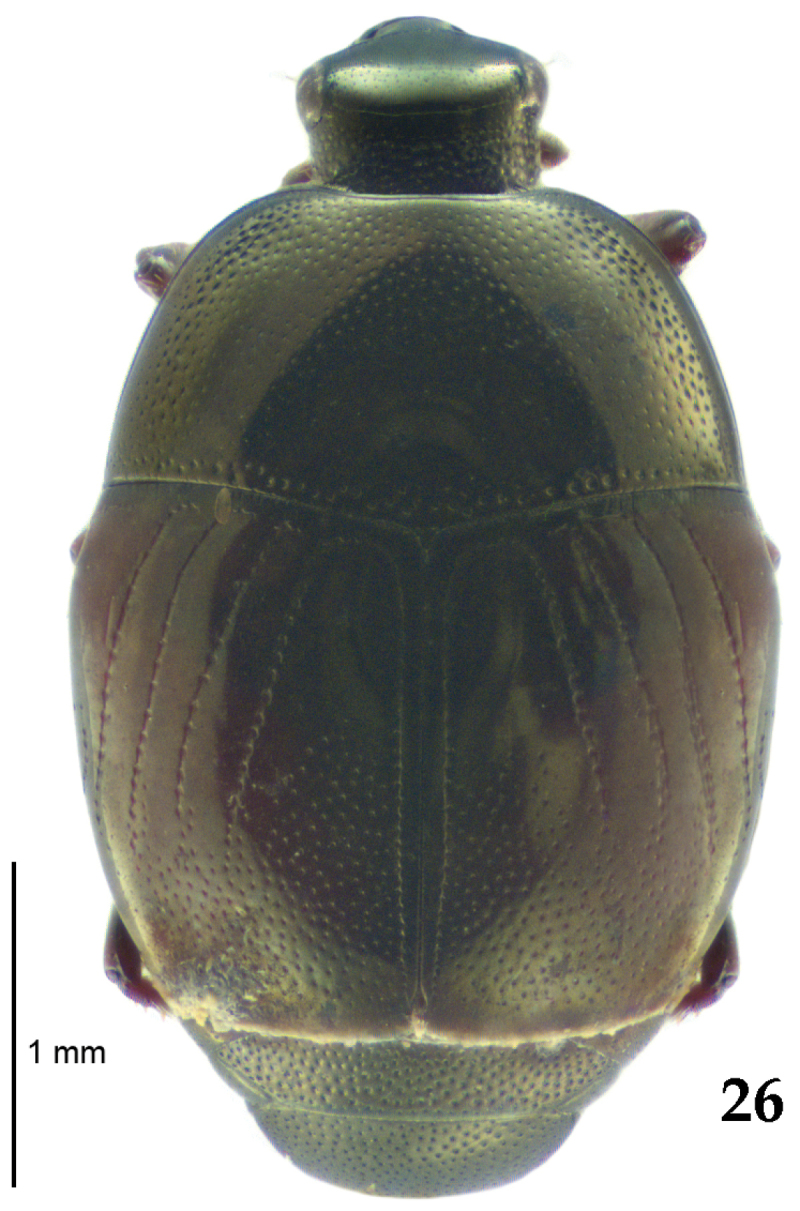
*Chalcionellus aeneovirens* (Schmidt, 1890) habitus, dorsal view.

##### Biology.

The species *C. aeneovirens* is found in mammal dung and on vertebrate carcasses.

##### Distribution.

This genus is widespread in the Old World; in the Australopacific Region a single introduced species, *Chalcionellus aeneovirens* (native to the Afrotropical Region) is found in Western Australia, Victoria and Queensland (Fig. 752).

##### Remarks.

The sole species of Australian *Chalcionellus*, *C. aeneovirens* (Schmidt, 1890) differs from the rest of the Australian Saprininae by the presence of pronotal depressions in combination with well developed and deep prosternal foveae, connected by marginal prosternal stria. Superficially it could be confused with the similarly introduced species Hypocaccus (Nessus) interpunctatus interpunctatus (Schmidt, 1885), but the prosternal foveae of the former species are not connected by the marginal prosternal stria and, furthermore, H. (N.) interpunctatus interpunctatus possesses a characteristic ‘mirror’ (=polished area) on the second elytral interval, absent in *Chalcionellus aeneovirens*. From the externally similar species of the genus *Hypocaccus*, the species *C. aeneovirens* differs by the anteriorly connected prosternal foveae as well as by the absence of frontal rugae, typical for *Hypocaccus*.

#### Chalcionellus aeneovirens

Taxon classificationAnimaliaColeopteraHisteridae

(Schmidt, 1890)

Figs 26 , 27–38 , 39–47 , 753

Saprinus aeneovirens Schmidt, 1890: 84.

##### Type locality.

Somalia.

##### Type material examined.

*Saprinus aeneovirens* Schmidt, 1890: Lectotype, ♂, designated by Gomy & Vienna in 1999, glued on the tip of a triangular mounting card, left mesotibia broken off and glued to the same mounting card as the specimen, genitalia extracted and glued next to the mesotibia on the mounting card, with the following labels: “♂” (written); followed by: “Somaliland / Deyrolle 1.3.85” (written); followed by: “aeneovirens / Schm. Typ” (written); followed by: “coll. J. Schmidt” (printed); followed by: “coll. Schmidt - / Bickhardt” (printed); followed by: “Type” (brick-red label, printed); followed by: aeneovi- / rens * J. Schmidt” (light blue label, written); followed by: “aeneovirens / J. Schmidt / H. Bickhardt det. 1919” (printed-written); followed by: “LECTOTYPUS / Chalcionellus / aeneovirens (Schmidt) / Gomy et Vienna des., 1999” (red label, printed) (ZMHUB). Paralectotype, ♀, designated by Gomy & Vienna in 1999, right mesotarsus and left metatarsus broken off and glued onto the mounting card of the specimen together with the extracted female genitalia, with the following labels: “♀” (written); followed by: “Somali / land” (written); followed by: “Type” (brick-red label, printed); followed by: “coll. Schmidt - / Bickhardt” (printed); followed by: “PARALECTOTYPUS / Chalcionellus / aeneovirens (Schmidt) / Gomy et Vienna des., 1999” (red label, printed) (ZMHUB).

**Figures 27–38.**
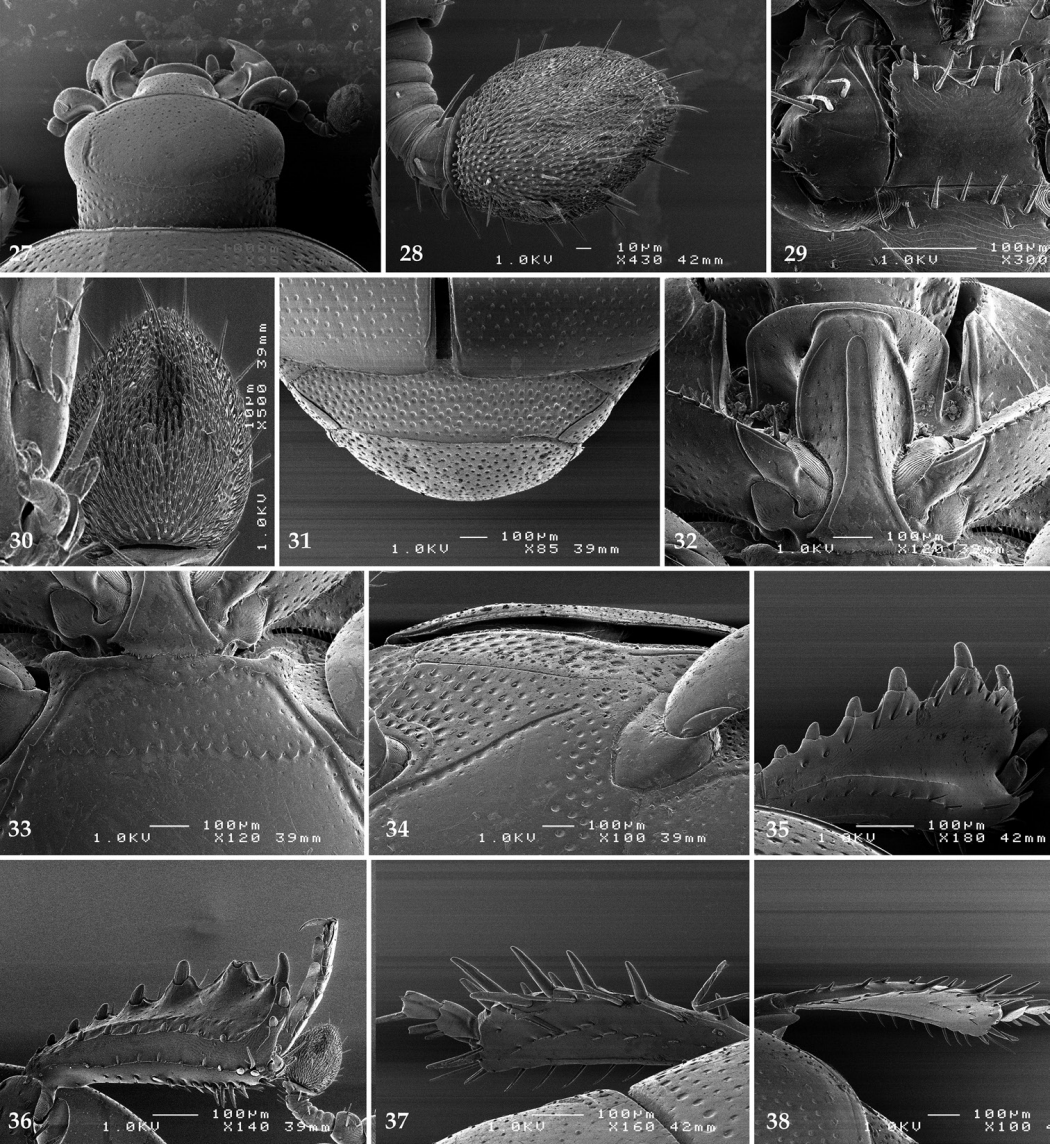
**27** *Chalcionellus aeneovirens* (Schmidt, 1890) head, dorsal view **28** antennal club, dorsal view **29** mentum, ventral view **30** antennal club, ventral view showing sensory structures of the antenna **31** propygydium + pygidium **32** prosternum **33** mesoventrite **34** lateral disc of metaventrite + metepisternum **35** protibia, dorsal view **36** ditto, ventral view **37** mesotibia, dorsal view **38** metatibia, dorsal view.

##### Additional material examined.

AUSTRALIA. Western Australia: 1 spec., Dardanup, 1.ii.1979, G. Hall (howden trap) (ANIC); 1 spec., Gingin Brook, 6.x.1977, J. Ridsdill Smith (ANIC); 1 spec., Cataby, 12.vi.1979, G. Hall (howden trap) (ANIC); 4 specs., 15 km S Busselton, 11.i.1983, G.P. Hall (ANIC); 3 specs., ditto, but 12.i.1983 (ANIC); 1 spec., ditto, but 15.i.1983 (ANIC); 1 spec., ditto, but 27.i.1983 (ANIC); 5 specs., 5 km NE Dardanup, 11.i.1983, G.P. Hall (ANIC); 6 specs., ditto, but 13.i.1983 (ANIC; 2 exs. in coll. TLAN); 3 specs., ditto, but 12.i.1983 (ANIC); 10 specs., ditto, but 15.i.1983 (ANIC); 3 specs., ditto, but 27.x.1982 (ANIC); 2 specs., ditto, but 19.x.1982 (ANIC); 1 spec., ditto, but 21.x.1982 (ANIC); 1 spec., ditto, but 4.xi.1982 (ANIC).

Victoria: 2 ♂♂ & 1 ♀, Cheltenham, 2.–3.ii.1918, H. Pottinger (QM).

Queensland: 2 specs., Bangalee Beach, 10 m, 23°04'S, 150°46'E, 16.–19.xii.1999, D. & I. Cook (littoral R/F dung pitfall) (QM).

**Figures 39–47.**
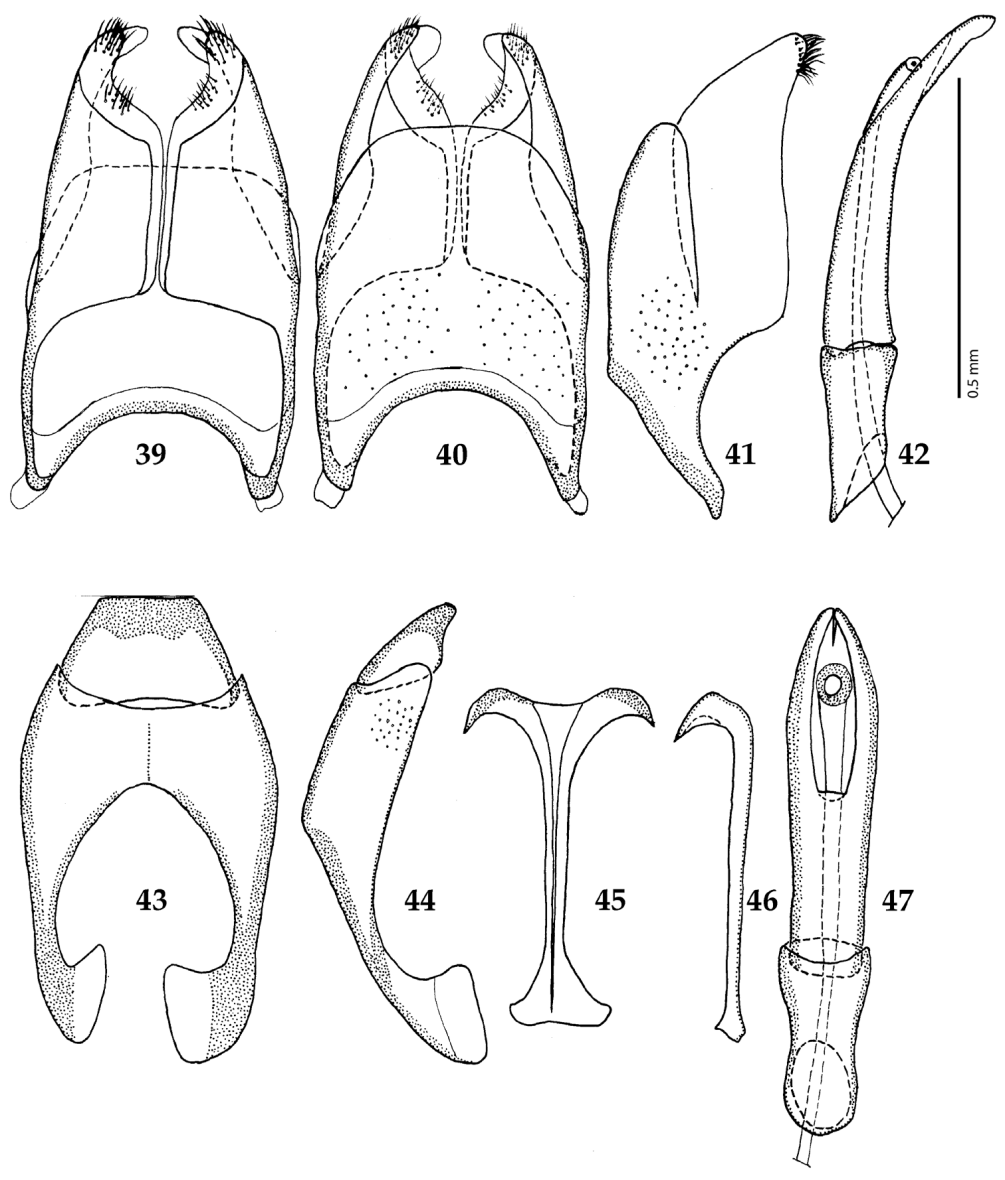
**39** *Chalcionellus aeneovirens* (Schmidt, 1890) male terminalia: 8^th^ sternite + 8^th^ tergite, ventral view **40** ditto, dorsal view **41** ditto, lateral view **42** male terminalia: aedeagus, lateral view **43** male terminalia: 9^th^ + 10^th^ tergites, dorsal view **44** ditto, lateral view **45** male terminalia: spiculum gastrale, ventral view **46** ditto, lateral view **47** male terminalia: aedeagus, dorsal view.

##### Biology.

Saprobiont, found often in dung or on carcass.

##### Distribution.

Described from Somalia, and widespread in East and South Africa; introduced into Australia (Queensland, Western Australia and Victoria; Fig. 753) (Mazur 2011).

##### Remarks.

The species is diagnosed above, as well as provided with a differential diagnosis that separates it from other Australian taxa. We chose not to fully re-describe it here, leaving its re-description to the revision of the genus *Chalcionellus*. For the sake of the better species recognition, however, we decided to depict it here, including its male terminalia.

#### Euspilotus

Taxon classificationAnimaliaColeopteraHisteridae

Lewis, 1907

Figs 48 , 49–57 , 58–60 , 61–67 , 754

Euspilotus Lewis, 1907: 320. Type species Euspilotus zonalis Lewis, 1907, original designation.

##### Distribution.

This species-rich genus with 76 described species is distributed across North, Central and South America (Mazur 2011), with one species known also from the Palaearctic and one from Oriental Region, respectively. In the Australopacific Region we report only one introduced species, E. (Neosaprinus) rubriculus (Marseul, 1855) represented in collections by several old specimens presumably collected from New Zealand and Australia.

#### Subgenus Neosaprinus

Taxon classificationAnimaliaColeopteraHisteridae

Bickhardt, 1909

Neosaprinus Bickhardt, 1909: 243. Type species Saprinus gnathoncoides Bickhardt, 1909 (=Euspilotus rubriculus (Marseul, 1855)), by monotypy.

##### Diagnosis.

Diagnosis of this subgenus is based solely on the species E. (N.) rubriculus that has been recorded from the Australopacific Region. Frontal and supraorbital striae absent; eyes visible from above; cuticle dark brown to black; elytral striae 1-4 well developed; carinal prosternal striae divergent on apical half, thence running parallel, united anteriorly by deep sulcus; lateral prosternal striae shortened, attaining carinal prosternal striae at the point where these turn parallel; meso-metaventral stria absent; metepisternal stria complete.

**Figure 48.**
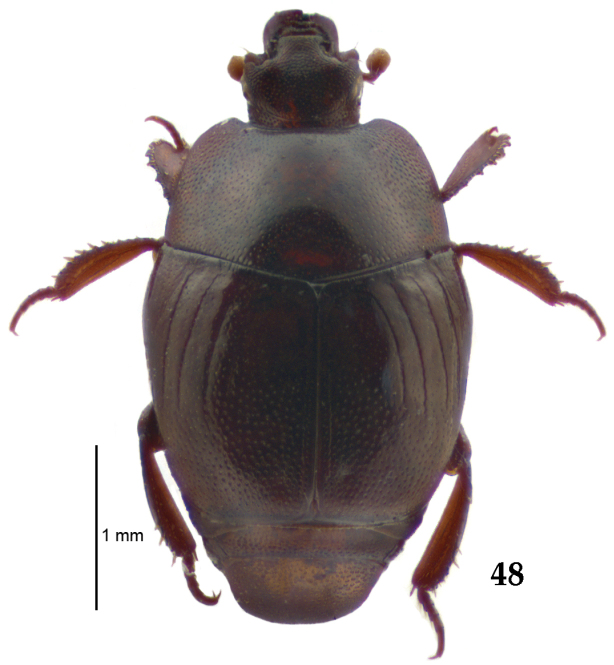
Euspilotus (Neosaprinus) rubriculus (Marseul, 1855) habitus, dorsal view/

##### Biology.

The biology of the members of the subgenus Neosaprinus is poorly documented, but species of this subgenus are usually found on the carcasses of vertebrates (Arriagada, pers. comm., 2014) or in caves (A.K. Tishechkin, unpublished). Gomy (2005) reports this species from the Island of La Réunion, collected inside a lava tube in the faeces of the Mascarene Swiftlet (*Collocalia francica* Gmelin, 1789) and concluded that its cavernicolous habitat is nothing exceptional. He (Gomy 2005) hypothesized that this species could have come from Brazil with a shipment of some kind of legumes, probably soybeans or corn. The labels of specimens of E. (N.) rubriculus recorded from Australia and/or New Zealand did not bear any information regarding their biology.

##### Distribution.

The subgenus Neosaprinus of the genus *Euspilotus* currently comprises nine described species, most of them occurring in the Neotropical Region (Mazur 2011). One species, E. (N.) scrupularis (J.L. LeConte, 1860) is distributed across North America, from British Columbia (Canada, with doubt), through Texas, Arizona, Utah, California, Washington, Oregon (with doubt) to Florida (USA), as well as in Mexico (Bousquet and Laplante 2006; Mazur 1997; Hatch 1962). One species, E. (N.) loebli Mazur, 1974 has been described from Malaysia and another one, E. (N.) perrisi (Marseul, 1872) occurs in the Palaearctic Region. In the Australopacific Region, we have examined three old specimens of E. (N.) rubriculus: two from Australia and one from New Zealand (Fig. 754).

##### Remarks.

This taxon is most likely to be confused with the genus *Gnathoncus*, with which it shares the absence of both frontal and supraorbital striae, but can easily be separated from it by single marginal epipleural stria (double in *Gnathoncus*) and absence of the hooked appendix between the fourth dorsal elytral and sutural elytral striae, characteristic for *Gnathoncus*. Furthermore, E. (N.) rubriculus has small prosternal foveae connected by a transverse sulcus whereas species of the genus *Gnathoncus* lack these structures. The examined specimens from Australopacific Region of this species are: a single female from Sydney (OUMNH), a single male from Brisbane (QM), both Australia and a single female from Tairua, New Zealand (NZAC). This New Zealand specimen was possibly traded or given originally to Thomas Broun who lived in Tairua. We treat this species in our study as a potential introduction because it has also been reported elsewhere outside of its native range (e.g. La Réunion; see Lackner 2013c).

#### Euspilotus (Neosaprinus) rubriculus

Taxon classificationAnimaliaColeopteraHisteridae

Marseul, 1855

Figs 48 , 49–57 , 58–60 , 61–67 , 754

Saprinus rubriculus Marseul, 1855: 489.
Saprinus gnathoncoides Bickhardt, 1909: 243 – Synonymized by Arriagada (1987): 68.

##### Type locality.

America? (without further data).

##### Type material examined.

*Saprinus rubriculus* Marseul, 1855: Lectotype, present designation: ♂, with genitalia placed in a small vial under the specimen, both protarsi, left mesotarsus, right metatarsus and left hind leg missing, pinned, with the following labels: tiny, rectangular pink label, followed by: “107 / Saprinus / rubriculus / m. / N.” (round, green label, written); followed by: “Horn / 11 mars 43 ?” (almost illegible label, written); followed by: “6a rubriculus / N. Am” (written); followed by: “MUSEUM PARIS / COLL. / DE MARSEUL 1890” (printed); followed by: “TYPE” (red-printed label); followed by: “Saprinus rubriculus / Marseul, 1855 / LECTOTYPE / des. T. Lackner, 2014” (red label, written) (MNHN). The type locality of this species is “America?” with a question mark. It is unclear why Mazur (1997, 2011) does not mention this doubt with the type locality. This species was designated from unknown number of specimens and the lectotype designation fixes the identity of the species.

**Figures 49–57.**
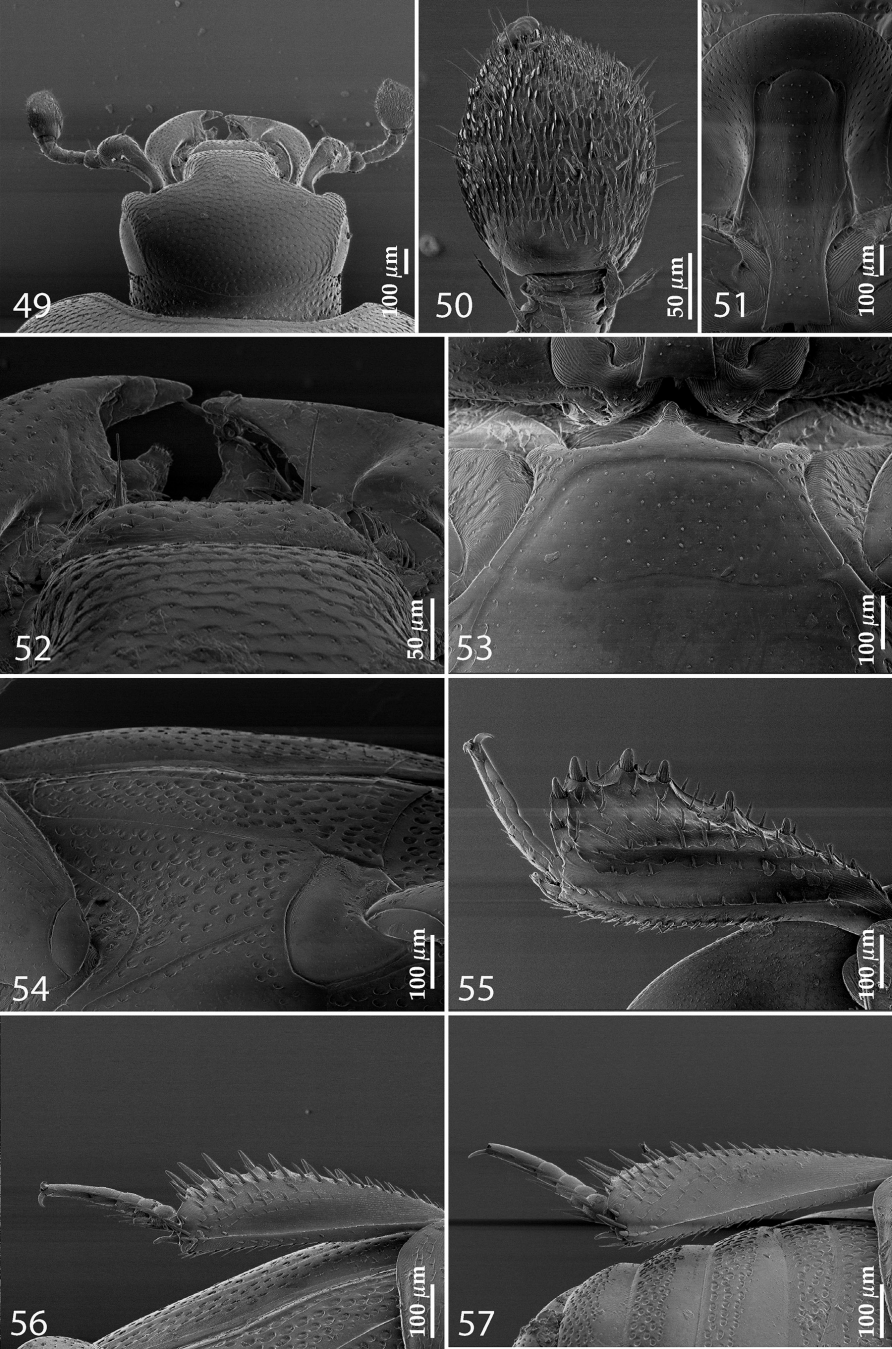
**49** Euspilotus (Neosaprinus) rubriculus (Marseul, 1855) head, dorsal view **50** antennal club, dorsal view **51** prosternum **52** clypeus + labrum **53** mesoventrite **54** lateral disc of metaventrite + metepisternum **55** protibia, ventral view **56** mesotibia, ventral view **57** metatibia, ventral view.

*Saprinus gnathoncoides* Bickhardt, 1909: holotype, ♂, side-mounted on a triangular mounting card, with the following labels: “Montevideo / 28.xi.[19]08 / J. Tremoleras” (black-margined printed-written); followed by: “*gnathoncoides* / Bickh.” (written); followed by: “*gnathoncoides* / m / det. Bickhardt” (written-printed); followed by: “Type” (red label, printed); “Zool. Mus. / Berlin” (printed); followed by: “10-152” (yellow label, pencil-written, added by the senior author); followed by: “10-166” (yellow label, pencil-written, added by the senior author) (ZMHUB). This species was described from a single specimen (holotype by monotypy) examined here.

##### Additional material examined.

URUGUAY: 1 ♀, most likely collected with the type specimen, but without the type label: Montevideo, 28.xi.[19]08, J. Tremoleras (ZMHUB). ARGENTINA: 3 ♂♂, Cordoba prov., Balnearia, 17.–25.ii.2000, collector unknown (TLAN). La REUNION: 1 ♂, L’Eperon, Boyau de lave, Deject salanganes, 28.xi.2003, J. Pousserau leg. (CYG). AUSTRALIA: Queensland: 1 ♂, Brisbane, O.W. Tiegs (QM); 1 ♀ New South Wales: Sydney District, J.J.W, iii.1900 (OUMNH). NEW ZEALAND: North Island: CO: 1 ♀ Broun Tairua, Coll. Wakefield, Arthur Parrott Collection donated June 1983 (NZAC).

**Figures 58–60.**
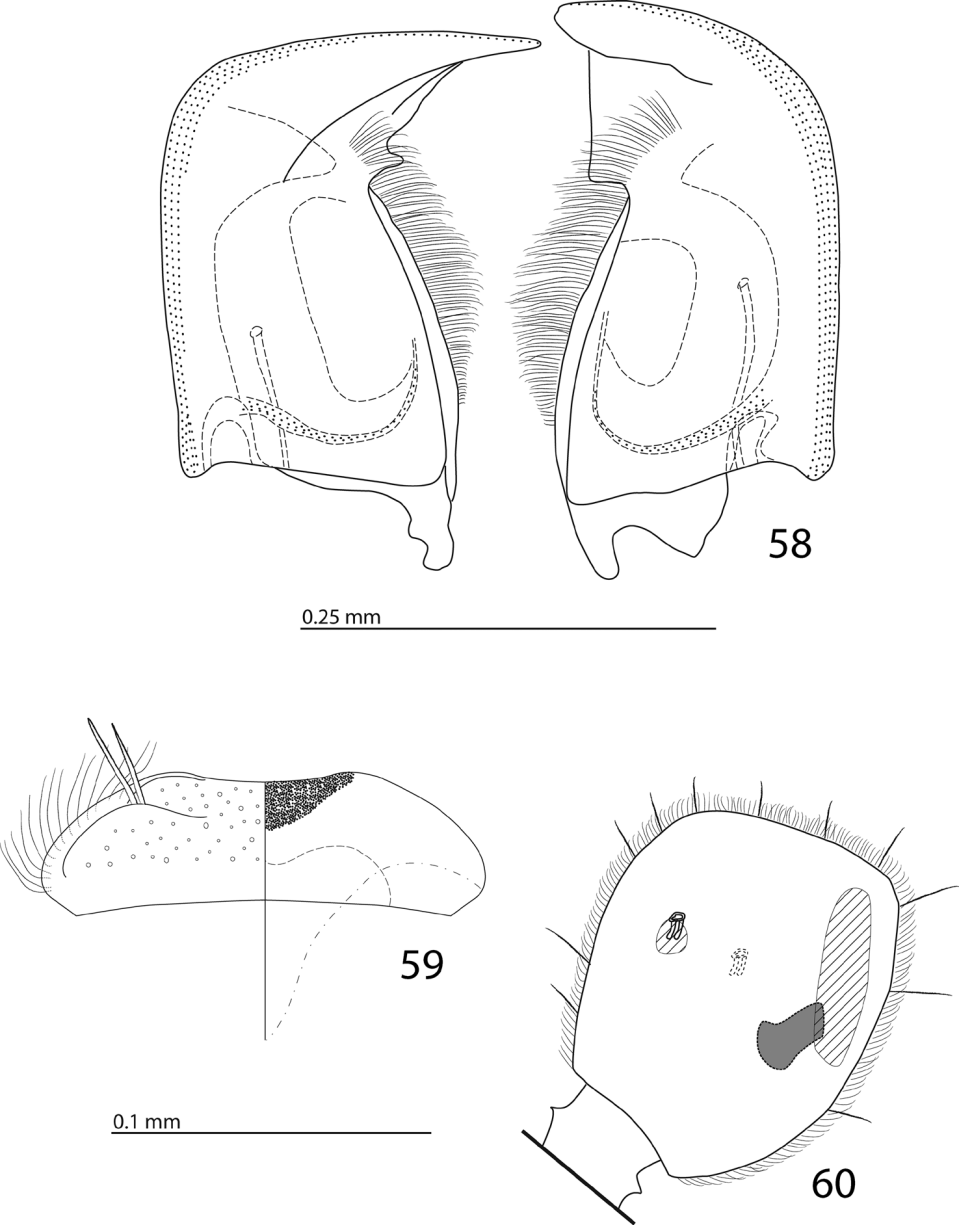
**58** Euspilotus (Neosaprinus) rubriculus (Marseul, 1855) mandibles, dorsal view **59** labrum: left half depicting dorsal view and right half depicting epipharynx **60** antennal club, ventral view showing sensory structures of the antenna.

##### Biology.

Biology of this species is not well documented, but it has been found inside a lava tube on feces of the Mascarene Swiftlet (Lackner 2013c; see also above) where it has been introduced to La Reunion. Arriagada (1987) reports this species from the excrements of common vampire bat (*Desmodus rotundus* Geoffroy, 1810) in a cave in the Cordoba province of Argentina.

##### Distribution.

Described from “America?”, recorded from Argentina, Uruguay, Brazil, Venezuela as well as from islands of St. Helena and La Réunion; possibly adventive to New Zealand and Australia (Fig. 754).

**Figures 61–67.**
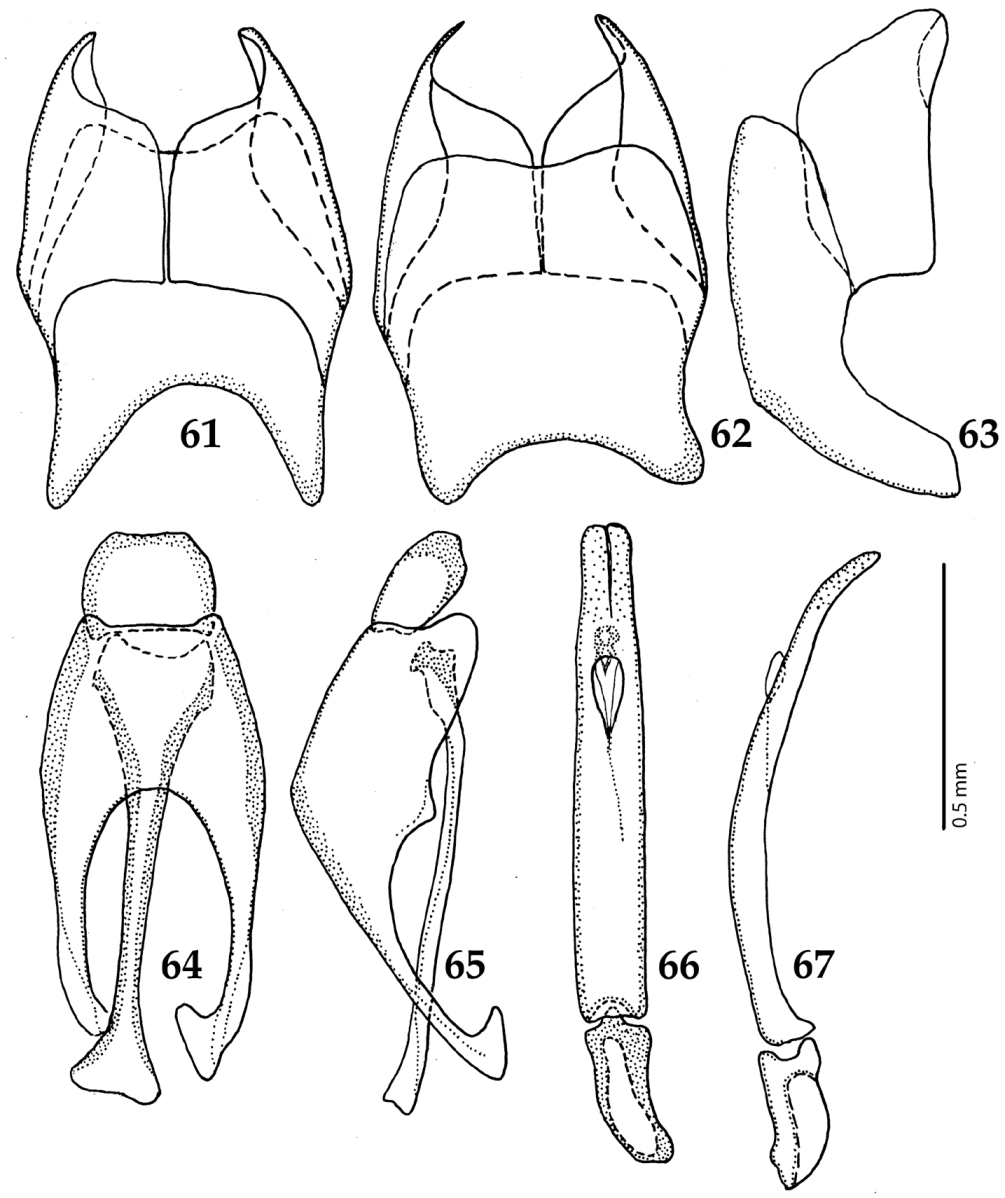
**61** Euspilotus (Neosaprinus) rubriculus (Marseul, 1855) male terminalia: 8^th^ sternite + 8^th^ tergite, ventral view **62** ditto, dorsal view **63** ditto, lateral view **64** 9^th^ + 10^th^ tergites, dorsal view; spiculum gastrale, ventral view **65** 9^th^ + 10^th^ tergites + spiculum gastrale, lateral view **66** aedegus, dorsal view **67** ditto, lateral view.

##### Remarks.

The specimen from “Sydney District” (OUMNH) is slightly larger (PEL: 2.85 mm; APW: 1.05 mm; PPW: 2.00 mm; EL: 1.75 mm; EW: 2.125 mm) in size and has a short median fragment of the inner subhumeral stria, compared to the syntype specimen of *S. gnathoncoides* Bickhardt, 1909 (ZMHUB) that is slightly smaller (PEL: 2.25 mm; APW: 1.00 mm; PPW: 1.625 mm; EL: 1.25 mm; EW: 1.875 mm) and bears no trace of inner subhumeral stria. It is possible that E. (N.) rubriculus represents a complex of cryptic species though the male terminalia of the lectotype of *S. gnathoncoides* and those of the specimen from Brisbane are almost identical. The species is diagnosed above, as well as provided with a differential diagnosis that separates it from other Australopacific taxa. We chose not to fully re-describe it here, leaving its re-description to the revision of the genus *Euspilotus* (Arriagada, in prep.) For the sake of better species recognition, however, we decided to depict it here, including its male terminalia.

#### Gnathoncus

Taxon classificationAnimaliaColeopteraHisteridae

Jacquelin du Val, 1857

Figs 68 , 69–80 , 81–89 , 90 , 91–98 , 99–102 , 103–110 , 754

Gnathoncus Jacquelin du Val, 1857: 112. Type species Hister rotundatus Kugelann, 1792, designated by Thomson (1859: 75).

##### Diagnosis.

Cuticle brown to black, never metallic; frontal, supraorbital striae absent; pronotal hypomeron glabrous; pronotal depressions absent; elytra occasionally imbricate, punctures on apical part of elytra sometimes forming longitudinal rugae; marginal epipleural stria double; fourth dorsal elytral stria never connected with sutural stria; apical elytral stria usually shortened; elytra with characteristic hooked appendix between fourth dorsal and sutural striae at elytral base; anterior ends of fourth dorsal elytral and sutural elytral striae form a small hook. Prosternum without prosternal foveae; median fossa often present; carinal prosternal striae strongly convergent anteriorly, united under sharp angle; lateral prosternal striae shortened, strongly convergent anteriorly; prosternal process flattened, broad; outer-lateral costa reaches prosternal process, its basal margin distinctly elevated; metaventrite of males at times longitudinally concave; ninth tergite of male genitalia divided longitudinally.

**Figure 68.**
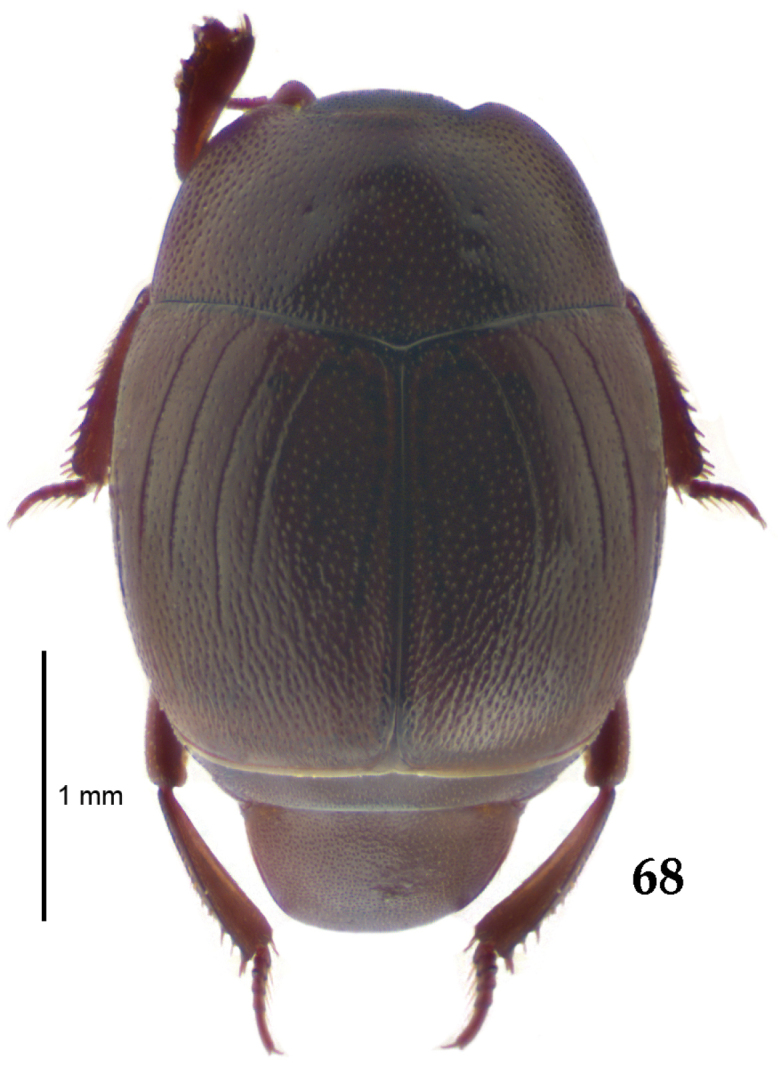
*Gnathoncus communis* (Marseul, 1862) habitus, dorsal view.

##### Biology.

*Gnathoncus* is predominantly composed of inquilinous species, present in the nests of birds or mammals; some species are found exclusively inside these nests where they are predators (Kryzhanovskij and Reichardt 1976), presumably preying upon larvae of fleas and other tiny arthropods. Some species, however, are occasionally collected on carrion. Both species of *Gnathoncus* occurring in the Australopacific Region are typical synanthropes and are often collected in pigsties, dovecotes or chicken coops.

##### Distribution.

Twenty-four species and subspecies are known to occur worldwide, most in the Holarctic Region (Mazur 2011); several species were possibly distributed over the globe by human activity. In Australopacific Region two introduced species have been collected in New Zealand and Australia (Fig. 754).

##### Remarks.

**Figures 69–80.**
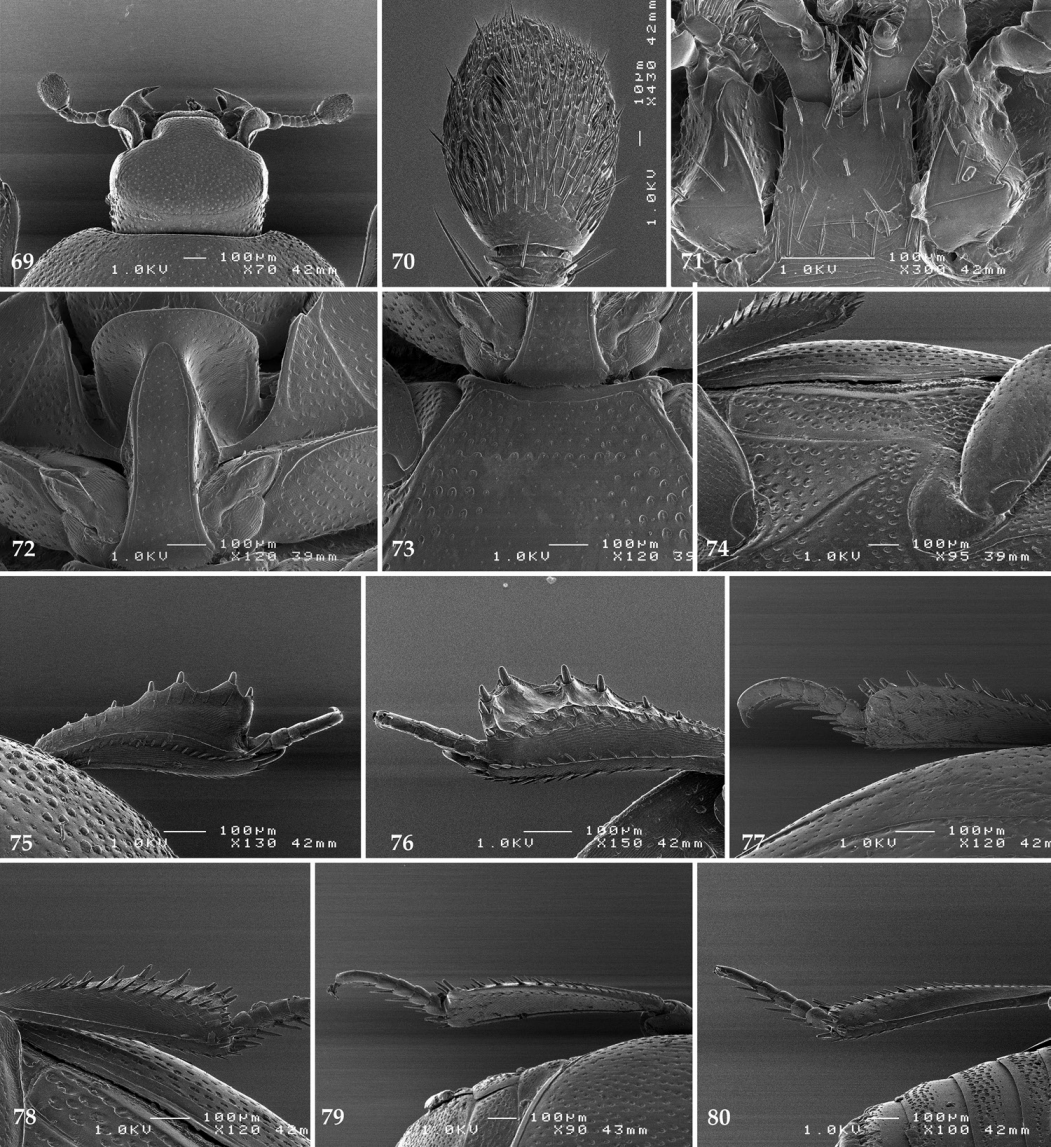
**69** *Gnathoncus communis* (Marseul, 1862) head, dorsal view **70** antennal club, ventral view showing sensory structures of the antenna **71** mentum, ventral view **72** prosternum **73** mesoventrite **74** lateral disc of metaventrite + metepisternum **75** protibia, dorsal view **76** ditto, ventral view **77** mesotibia, dorsal view **78** ditto, ventral view **79** metatibia, dorsal view **80** ditto, ventral view.

This genus can most easily be confused with the species of *Tomogenius*, endemic to the Australopacific Region, by the combination of absent frontal and supraorbital striae (Fig. 69) and presence of two marginal epipleural striae. Species of the genus *Gnathoncus* differ from *Tomogenius* by having a smaller body size, but chiefly by the absence of two large median foveae on the apex of prosternal process (Fig. 702; present in *Tomogenius*).

##### Key to the Australopacific species of the genus *Gnathoncus* Jacquelin du Val, 1857

**Table:** 

1	Apical third of elytra with confluent punctures forming almost longitudinal rugae, median fossa of prosternum absent (Fig. 72); male genitalia (Figs 81–89): apical margin of tenth tergite inwardly arcuate, with a tiny seta on each side; apical third of aedeagus strongly narrowed	***G. communis* (Marseul, 1862)**
–	Punctures of apical elytral third not confluent and not forming longitudinal rugae, median fossa of prosternum present (Fig. 94); male genitalia (Figs 103–110): apical margin of tenth tergite not inwardly arcuate, asetose; apical third of aedeagus only moderately narrowed	***G. rotundatus* (Kugelann, 1792)**

#### Gnathoncus communis

Taxon classificationAnimaliaColeopteraHisteridae

(Marseul, 1862)

Figs 68 , 69–80 , 81–89 , 754

Saprinus communis Marseul, 1862: 501.

##### Type locality.

USA: New York.

##### Type material examined.

*Saprinus communis* Marseul, 1862: Lectotype, present designation: most likely a ♀, pinned, left protarsus, right mesotarsus and both metatarsi missing, with the following labels: “Gnathoncus / communis m / Albany / illegible” (green round label, written); followed by: “2937” (written); followed by: “Horn / 19 man / 73” (written); followed by: “1 communis / Et. univ. illegible” (written); followed by: “MUSEUM PARIS / COLL. / DE MARSEUL 1890” (green label, printed); followed by: “TYPE” (red-printed label); followed by: “Saprinus communis / Marseul, 1862 / LECTOTYPE 2014 / des. T. Lackner” (red label, written) (MNHN). This species was described from unknown number of specimens and the lectotype designation fixes the identity of species.

**Figures 81–89.**
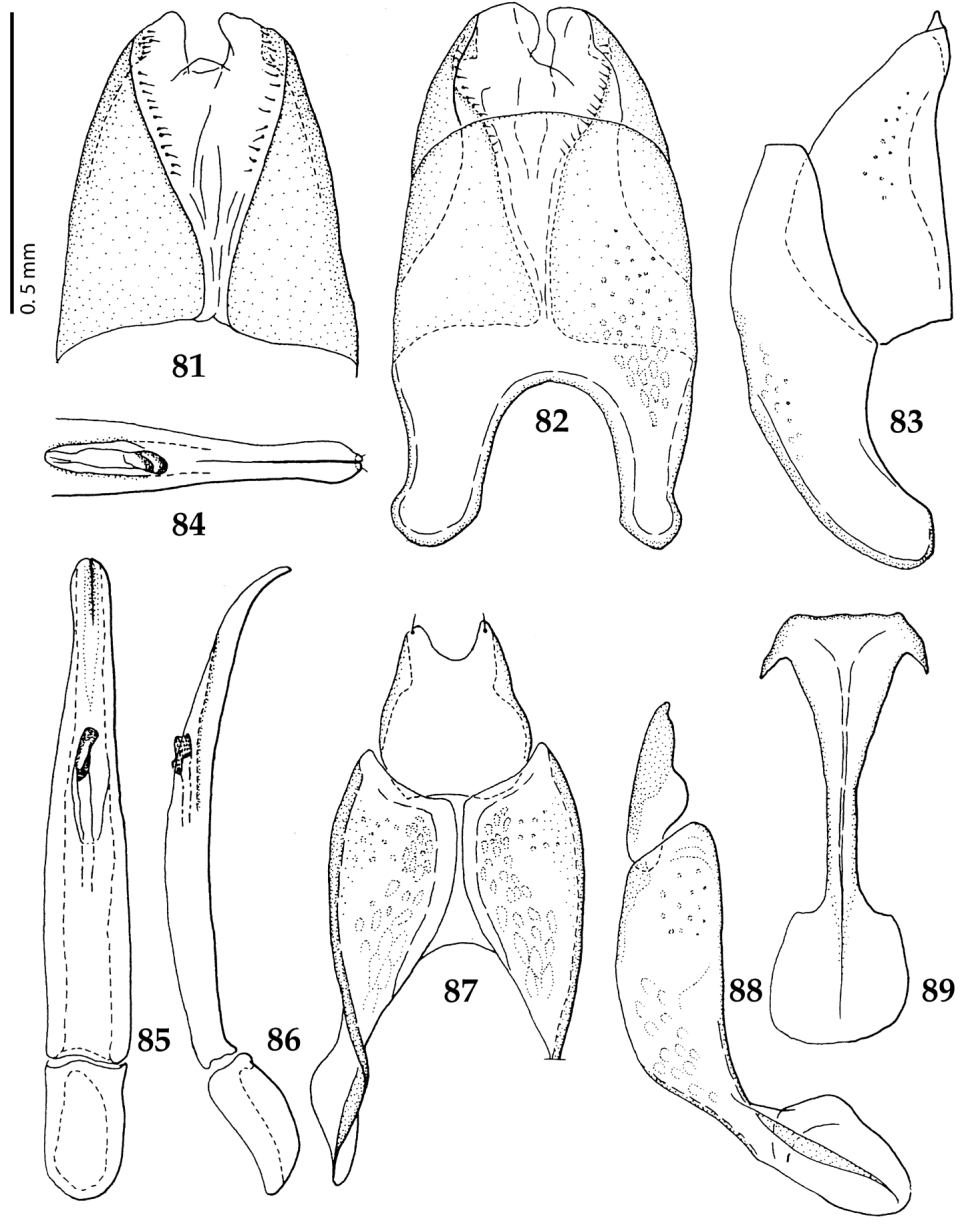
**81** *Gnathoncus communis* (Marseul, 1862) male terminalia after Ôhara (1994): 8^th^ sternite, ventral view **82** male terminalia: 8^th^ sternite + 8^th^ tergite, dorsal view **83** ditto, lateral view **84** male terminalia: apex of aedeagus, dorsal view **85** male terminalia: aedeagus, dorsal view **86** ditto, lateral view **87** male terminalia: 9^th^ + 10^th^ tergites, dorsal view **88** ditto, lateral view **89** male terminalia: spiculum gastrale, ventral view.

##### Additional material examined.

NEW ZEALAND. North Island. AK: 2 ♂♂ & 1 ♀, Lynfield, 19.vi.1976, G. Kuschel (hen house) (NZAC); 1 spec., ditto, but 14.xii.1980 (chicken yard) (NZAC); 2 specs., Ranui, Henderson, 29.xi.1955, K.A.J. Wise (ex debris in poultry feed shed) (NZAC); 1 ♂, 1 ♀ & 1 spec., Tropicana Dr., 19.vi.1976, G. Kuschel (hen house) (NZAC); 1 spec., ditto, but 14.viii.1976 (NZAC); 7 ♂♂ & 3 ♀♀, Mahinerang, 26.i.1977, J. Painter (*Sturnus vulgaris* nest) (NZAC); 3 ♂♂ & 3 ♀♀, Kumeu, 21.vi.1975, J.C.Watt (ex fowl manure) (NZAC). BP: 1 ♂, Tauranga, 13.xi.1976, R.J. Ball (on plant) (NZAC). HB: 2 specs., Longlands, ii.1976, J.A. McKeenan (ex starling nest) (NZAC); 1 spec., Havelock North, 14.xi.1977, collector unknown (on large *Pinus* sp., ex nest of *Sturnus vulgaris*) (NZAC); 2 ♂♂ & 3 ♀♀, Graeme Friis’ Farm, Twyford, 2.xi.1976, B.A. Holloway (poultry manure) (NZAC).

##### Biology.

In New Zealand found mostly in bird’s nests, chicken coops, less commonly on bird carrion.

##### Distribution.

Described from New York (USA), where it was probably introduced and widespread across North America. This species is normally spread in Europe, North Africa, Russian Far East, Japan and has been introduced into Australia (Mazur 2011). Mazur (1990:744) reports this species as introduced into Australia (ACT: Canberra). The specimen Mazur examined should be housed in ANIC. However, we were unable to examine it or any other specimens of *G. communis* from Australia. From New Zealand reported already by Kuschel (1990).

##### Remarks.

*G. communis* can be confused with *G. rotundatus*, but can easily be separated from it by the absence of median fossa of prosternum (present with *G. rotundatus*), dense and confluent elytral punctation (especially in the apical half of the elytra) and different male genitalia. Most important diagnostic characters to separate the two *Gnathoncus* species present in the region are outlined in the identification key. We chose not to fully re-describe *Gnathoncus communis* here, leaving its re-description to the revision of the genus *Gnathoncus*. For the sake of the better species recognition, as well as for the easier separation from the related *G. rotundatus*, we decided to depict it here, including its male terminalia.

#### Gnathoncus rotundatus

Taxon classificationAnimaliaColeopteraHisteridae

(Kugelann, 1792)

Figs 90 , 91–98 , 99–102 , 103–110 , 754

Hister rotundatus Kugelann, 1792: 304.

##### Type locality.

Poland: Masuria: Ostróda.

##### Type material examined.

None examined for this study. Kugelann’s collection was destroyed during the WWII (T. Huflejt, Warsaw, Poland, pers. comm). It is not known, whether it contained the type material of *Hister rotundatus* or not.

**Figure 90.**
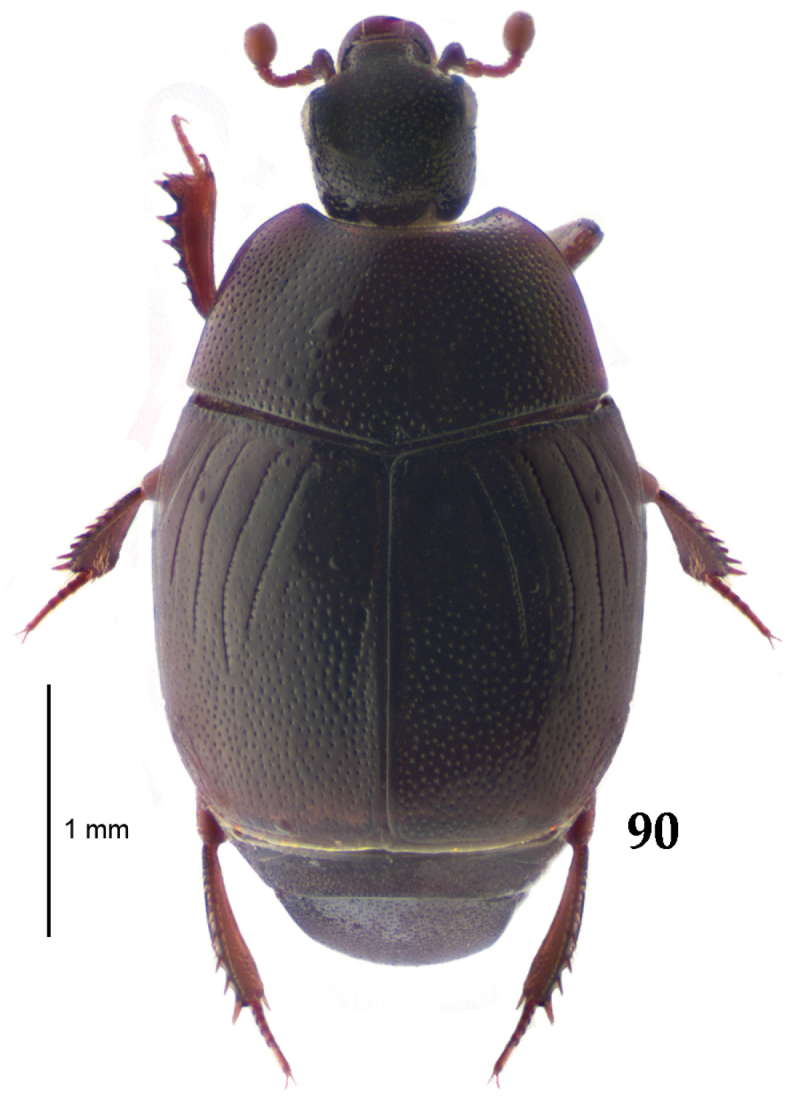
*Gnathoncus rotundatus* (Kugelann, 1792) habitus, dorsal view.

##### Additional material examined.

NEW ZEALAND. North Island. CO: 1 ♂, Earnscleugh, 28.x.1979, G. McLaren (suction trap) (NZAC). HB: 2 ♂♂, Longlands, ii.1976, J.A. McLemmans (starling nest) (NZAC); 1 ♂, Cape Kindnappers, Black Reef, 10.iii.1980, C.F. Butcher & M.F. Tacker (bird’s nest) (NZAC); 1 ♀, Motupiko, 9.ix.1951, G.G. Hole (NZAC); 6 ♂♂ & 7 ♀♀, Twijford, Hawke Bay, 2.xi.1976, B.A. Holloway (poultry manure) (NZAC); 34 ♂♂ & 29 ♀♀, Hastings, Twijford Poultry Farms, 21.x.1976, B.A. Holloway (poultry manure) (NZAC). AK: 1 spec., Auckland, New Zealand, Broun Coll. (BMNH); 1 ♂, Milford, 4.iv.1979, T. Thomas (nest of *Passer domesticus*) (NZAC); 1 ♂, Pareora Gorge, ii.1951, L.J. Dumbleton (shag nest) (NZAC); 1 ♂, Lynfield, 14.xii.1980, G. Kuschel (hen house) (NZAC); 1 spec., ditto, but 7.iii.1977 (sheep) (NZAC); 1 ♀, ditto, but 27.ii.1978, B.A. Holloway (guinea pig den material) (NZAC); 1 ♀, Kumeu, 21.vi.1975, J.C. Watt (ex fowl manure) (NZAC); 4 ♂♂ & 4 ♀♀, Nelson, 29.i.1971, E.W. Valentine (NZAC). South Island. 1 spec., Addington, Christchurch, 14.ix.1969, M.G. McPherson (inside house) (NZAC); 1 spec., Lincoln College, 18.i.1967, C. Boswell (NZAC); 2 specs., ditto, but 22.xii.1966 (NZAC); 1 spec., ditto, but 19.xi.1966 (NZAC); 2 specs., Prebbleton, 21.ix.1968, R.J. McPherson (ex fowl manure) (NZAC). DN: 6 specs., Otago Peninsula, Tairoa Head, 26.iii.1970, J.R. Jackson (ex *Larus dominicanus* or *Phalacrocorax punctatus* nest) (NZAC); 1 spec., Lincoln College, Hilgendorf wing, 8.x.1973, R.D. Welsh (ex window) (NZAC). MC: 2 specs., Banks Penisula, Murrays Mistake, 10.i.1970, J.R. Jackson (ex *Phalacrocorax punctatus* nest) (LUNZ); 1 spec., Hawkes Bay, Mangaome River, Dartnoor, 28.i.1959, B.M. May (on shingle) (NZAC); 1 spec., Lincoln College, 29.xii.1966, C.C. Boswell (NZAC); 1 ♀, ditto, but 18.vi.1967 (NZAC); 1 ♂, Rangiara, 7.xi.1947, D. Spiller (NZAC); 1 ♀, Maharoa, 9.xii.1941, J. Spiller (ex copra) (NZAC). Unknown localities: 1 ♂, no data (NZAC); 1 spec., Rakaia, 20.ii.1912, Broun Coll. (BMNH).

**Figures 91–98.**
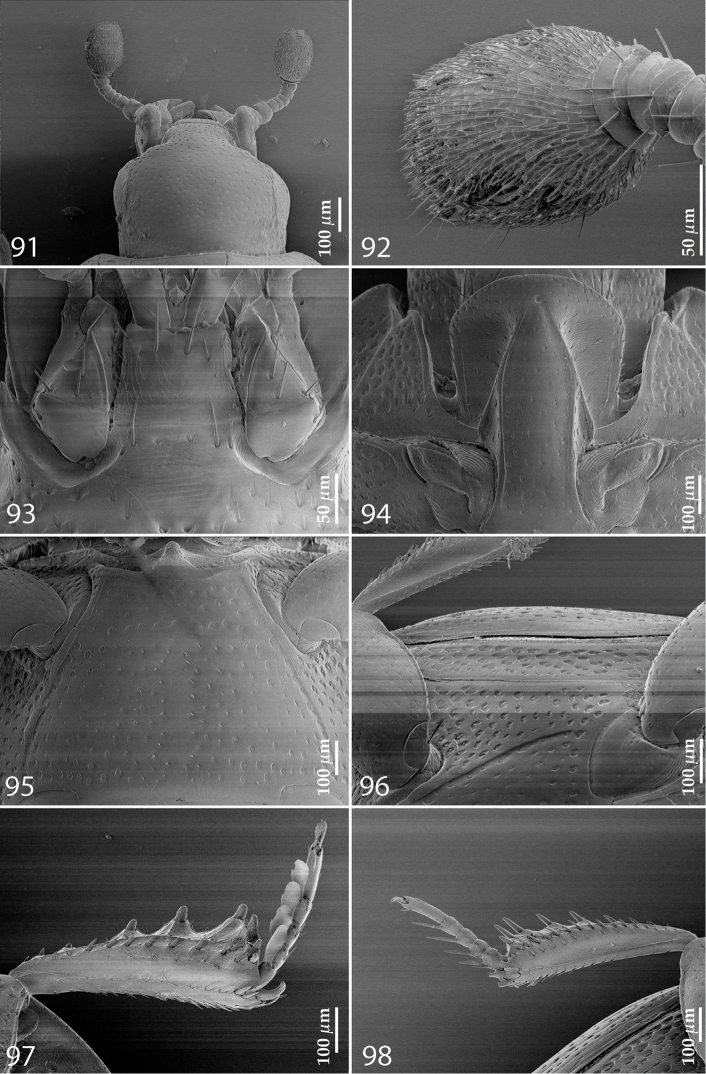
**91** *Gnathoncus rotundatus* (Kugelann, 1792) head, dorsal view **92** antennal club, ventral view **93** mentum, ventral view **94** prosternum **95** mesoventrite + metaventrite **96** lateral disc of metaventrite + metepisternum **97** protibia, ventral view **98** mesotibia, ventral view.

AUSTRALIA. Tasmania: 1 ♂ & 1 ♀, Launceston, 4 & 9.ii.1915, Littler Collection (NZAC). Australian Capital Territory: 2 ♂♂, Lyneham, 5.iv.1969, B.P. Moore, garden compost (ANIC).

##### Biology.

A typical synanthrope, found mainly in anthropogenic settings, but also apparent inquiline of bird’s nests.

##### Distribution.

Holarctic Region, Republic of South Africa, Chile, Saint Paul Island (Mazur 2011). From New Zealand reported already by Kuschel (1990), newly reported here for Australia (Fig. 754).

**Figures 99–102.**
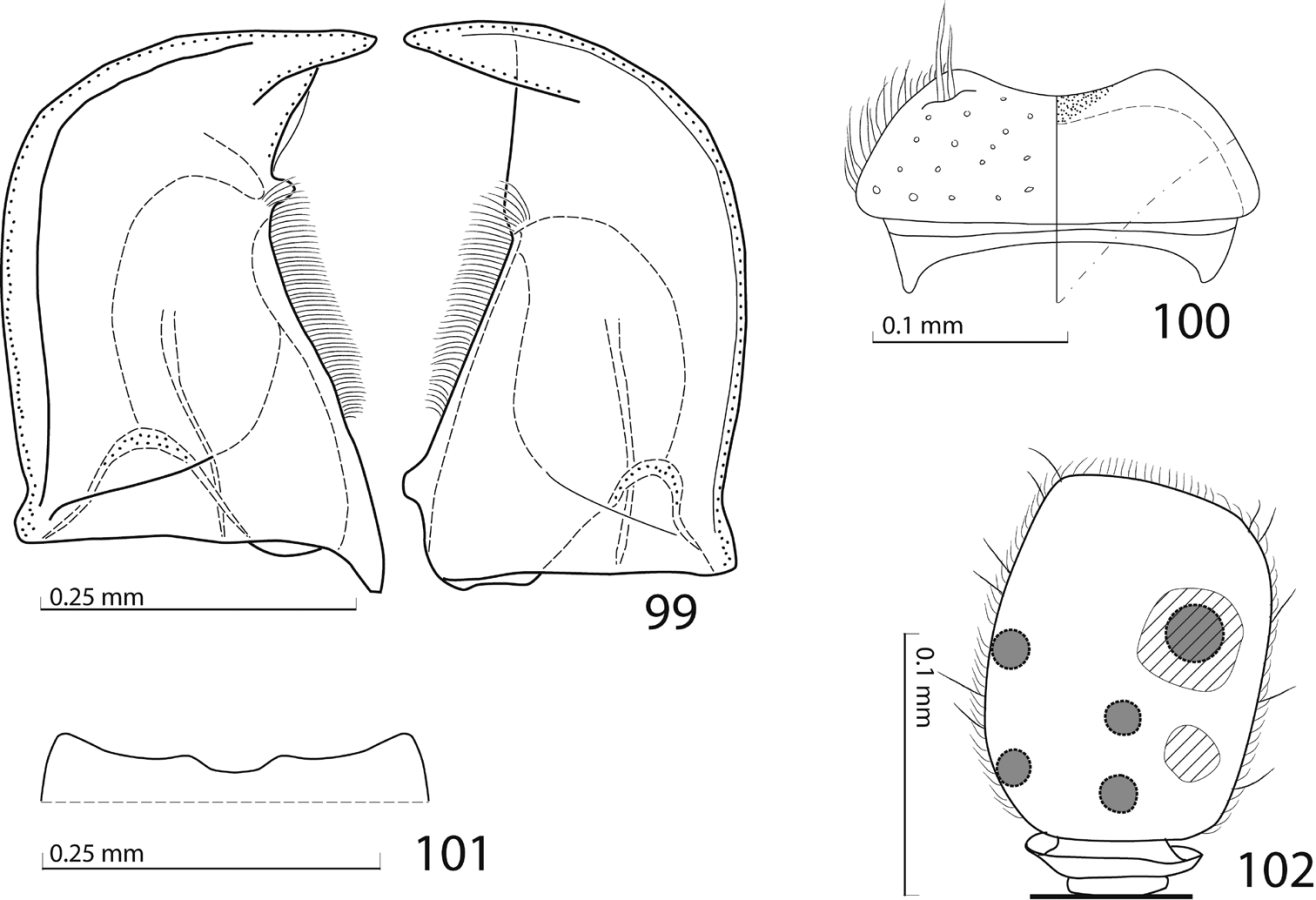
**99** *Gnathoncus rotundatus* (Kugelann, 1792) mandibles, dorsal view **100** labrum: left half depicting dorsal view and right half depicting epipharynx **101** anterior margin of mentum, ventral view **102** antennal club, ventral view showing sensory structures of the antenna.

##### Remarks.

This is a widely distributed species exhibiting some degree of variability regarding the length of elytral striae and density of dorsal punctation. Most important diagnostic characters to separate the two *Gnathoncus* species present in the region are outlined in the identification key. We chose not to fully re-describe *Gnathoncus rotundatus* here, the reader is instead referred to Lackner (2010: 118) for the detailed re-description. For the sake of better species recognition, as well as for easier separation from the related *G. communis*, we decided to depict it here, including its male terminalia.

**Figures 103–110.**
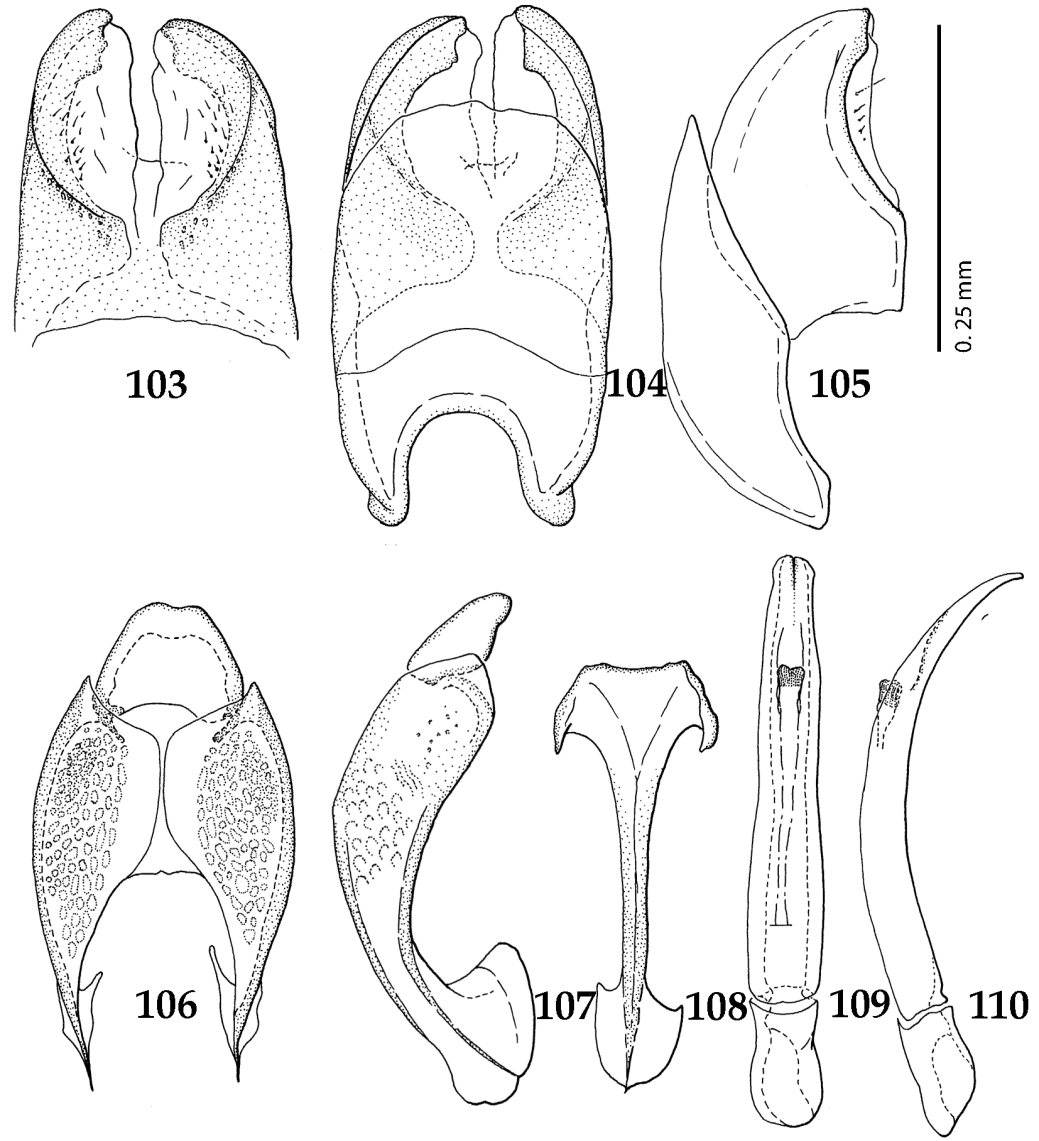
**103** *Gnathoncus rotundatus* (Kugelann, 1792) male terminalia after Ôhara (1994): 8^th^ sternite, ventral view **104** male terminalia: 8^th^ sternite + 8^th^ tergite, dorsal view **105** ditto, lateral view **106** male terminalia: 9^th^ + 10^th^ tergites, dorsal view **107** ditto, lateral view **108** male terminalia: spiculum gastrale, ventral view **109** male terminalia: aedeagus, dorsal view **110** ditto, lateral view.

#### Hypocacculus

Taxon classificationAnimaliaColeopteraHisteridae

Bickhardt, 1914

Figs 111 , 112 , 113–118 , 119–127 , 755

Hypocacculus Bickhardt, 1914: 311. Type species Saprinus metallescens Erichson, 1834, designated by Bickhardt 1916: 96.

##### Diagnosis.

Diagnosis of this genus is here based solely on the species *H. hyla* that has been recorded from the Australopacific Region. A small, ovoid beetle, cuticle dark brown with a faint metallic tinge; frontal stria complete, supraorbital stria lacking, frontal disc punctate. Pronotal depressions absent, almost entire dorsal surface in punctures. Prosternal foveae large; outer margin of protibia with 6–8 low teeth topped by denticle.

##### Biology.

*Hypocacculus* species are normally distributed in open landscapes, often in arid places, where they are usually found on carrion or in mammal excrement. Examined specimens of *H. hyla* do not carry any biological information on their labels.

##### Distribution.

The bulk of species of *Hypocacculus* are distributed in Palaearctic and Afrotropical Regions, with several species occurring also in the Oriental Region (Mazur 2011). Hypocacculus (H.) hyla (Marseul, 1864) was described from New Guinea and is the sole representative of the genus present in the Australopacific Region (Fig. 755).

##### Remarks.

Species of the genus *Hypocacculus* are similar in size and general habitus to those of *Hypocaccus* or *Chalcionellus*. They differ from members of the genus *Hypocaccus* by having a punctate frons, which is smooth and adorned with several deep rugae in *Hypocaccus*, and from *Chalcionellus aeneovirens*, the sole species of the genus *Chalcionellus* present in the region, by the absence of pronotal depressions, present in *C. aeneovirens*.

#### Subgenus Hypocacculus

Taxon classificationAnimaliaColeopteraHisteridae

s.str.

##### Diagnosis.

See above for the diagnosis of *Hypocacculus* s.l.

##### Biology.

See the biology of *Hypocacculus* s.l.

##### Distribution.

Members of this subgenus are spread in southern Palaearctic and Afrotropical Regions, with three species occurring also in the Oriental Region. Species H. (H.) metallescens (Erichson, 1834) has been introduced to USA (Florida) (Mazur 2011). In the Australopacific Region, one species, H. (H.) hyla, is present and was described from New Guinea, and occurs also in Indonesia (Java and Sunda), Vietnam, and Myanmar (Mazur 2011). Newly reported from India (see below).

#### Hypocacculus (Hypocacculus) hyla

Taxon classificationAnimaliaColeopteraHisteridae

(Marseul, 1864)

Figs 111 , 112 , 113–118 , 119–127 , 755

Saprinus hyla Marseul, 1864: 339.

##### Type locality.

New Guinea.

##### Type material examined.

*Saprinus hyla* Marseul, 1864: Lectotype, present designation: ♀, side-mounted on a triangular card with right metatibia (metatarsus missing) and left antennal club broken off and glued to the same mounting card, left metatibia missing, last two segments of left mesotarsus missing, both protarsi missing, with the following labels: “*Saprinus* / *hyla* / N. Guinée / Wallace illegible text” (yellow, round label, written); followed by: “*Saprinus* / *Hyla* / N. Guinea” (written); followed by: “MUSEUM PARIS / Coll. De Marseul / 2842-90” (printed); followed by: “TYPE” (red-printed label); followed by: “Saprinus / hyla Marseul, 1864 / LECTOTYPE / Des. by Lackner & / Leschen, 2011” (red label, written) (MNHN). This species has been described from an unknown number of specimens and the lectotype designation fixes its taxonomic identity.

##### Additional material examined.

PAPUA NEW GUINEA: 1 ♂, Huon Golf, Simbang, 1899, Biró leg. (HNMH). INDIA. Orissa: 1 ♂, Koraput Jeypore, 22.x.2006, G. de Rougemont (NCB).

##### Biology.

Unknown, probably a saprobiont.

##### Distribution.

See above (Fig. 755).

##### Remarks.

The male specimen from India matches the form of the female lectotype, only differing by its color, which is somewhat darker, and the punctation of the elytra, which is less dense apically compared to the New Guinean lectotype.

##### Re-description.

Body length: PEL: 1.625–1.80 mm; APW: 0.625–0.75 mm; PPW: 1.25–1.625 mm; EL: 1.00–1.25 mm; EW: 1.425–1.75 mm.

**Figure 111.**
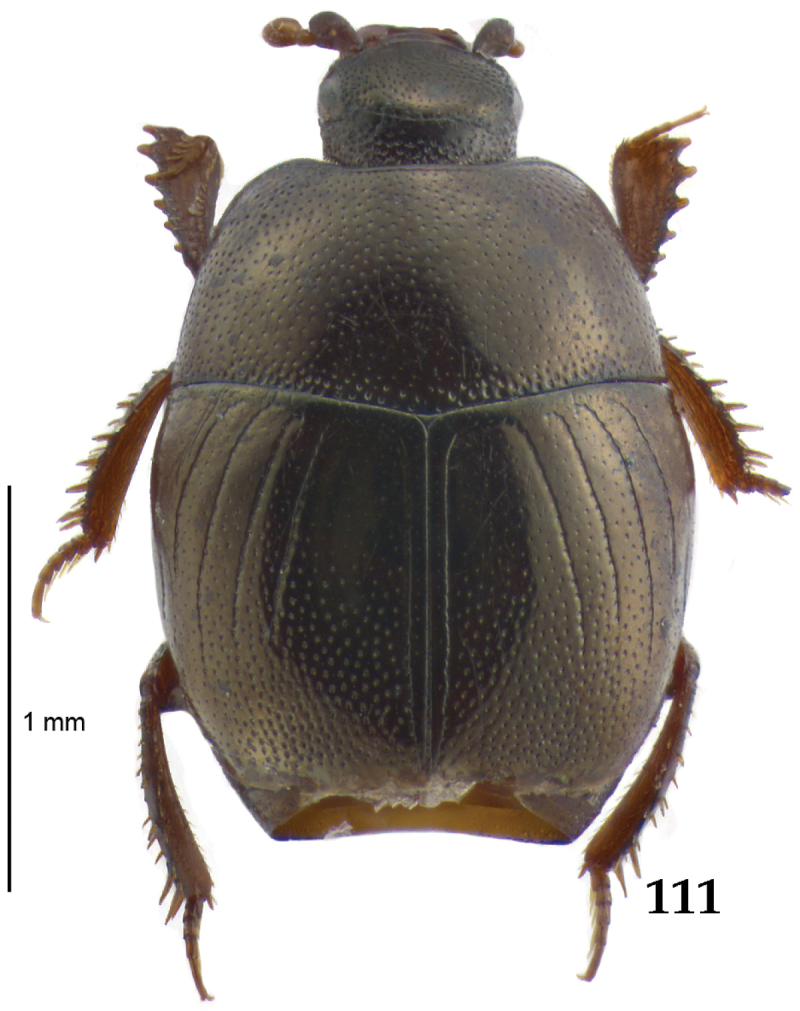
Hypocacculus (Hypocacculus) hyla (Marseul, 1864) habitus, dorsal view.

Body (Fig. 111) rectangular oval, dorsal surface convex, ventral surface flattened, cuticle light to darker brown with bronze metallic luster, legs, mouthparts and antennae light brown.

**Figure 112.**
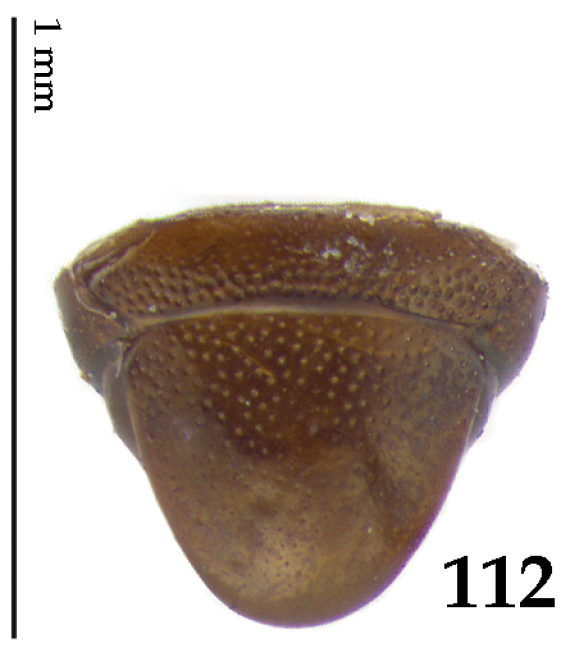
Hypocacculus (Hypocacculus) hyla (Marseul, 1864) propygidium + pygidium.

**Figures 113–118.**
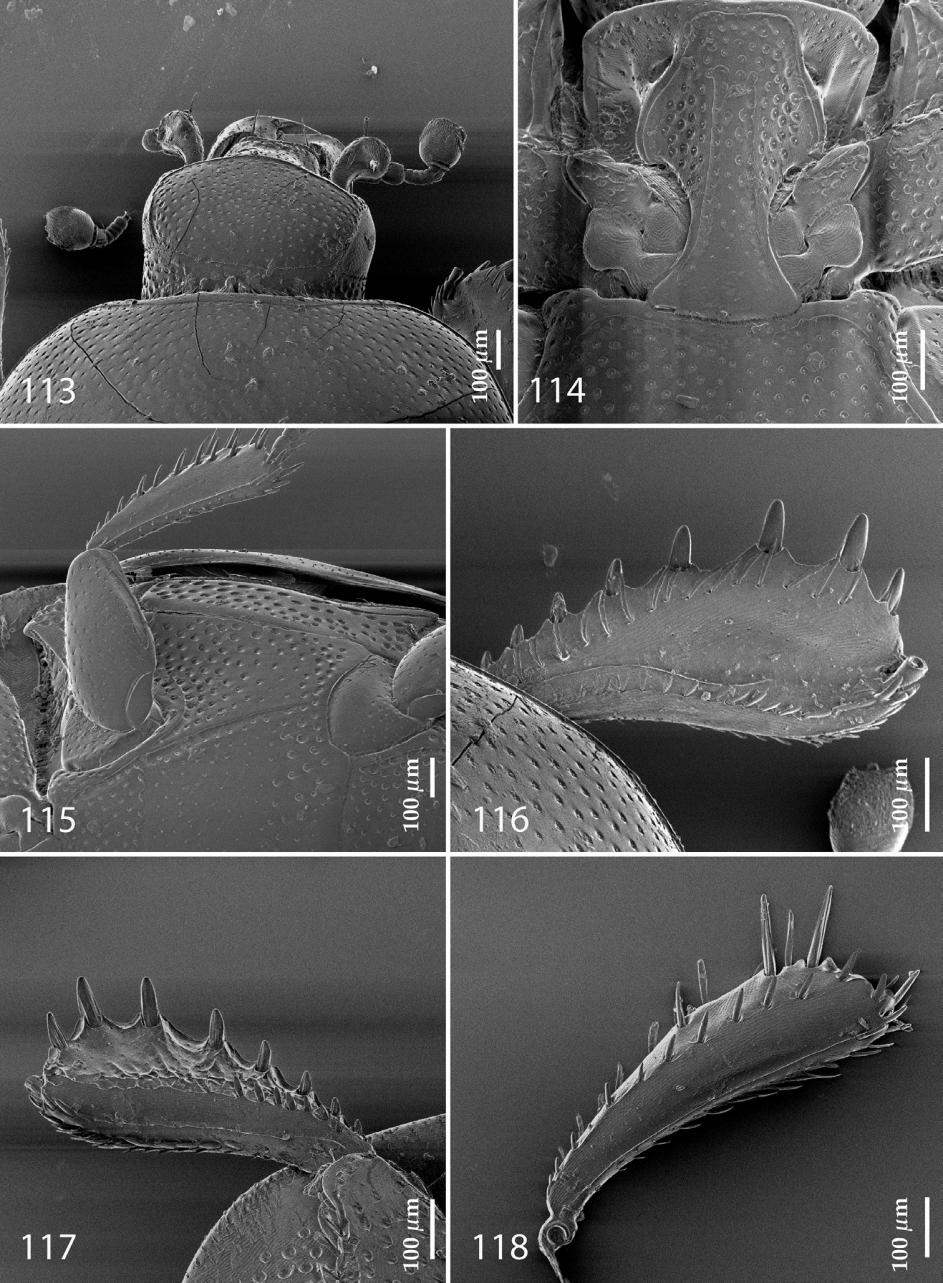
**113** Hypocacculus (Hypocacculus) hyla (Marseul, 1864) head, dorsal view **114** prosternum + mesoventrite **115** lateral disc of metaventrite + metepisternum **116** protibia, dorsal view **117** ditto, ventral view **118** metatibia, ventral view.

Antennal scape (Fig. 113) with lower margin carinate, with two short setae; antennal club (Fig. 113) round, lower half of its surface glabrous, upper half (approximately) with thick short yellow sensilla; sensory structures of antennal club not examined.

Mandibles stout, with rounded outer margin strongly curved inwardly, dorsally with sparse fine punctures, acutely pointed, sub-apical tooth on inner margin of left mandible not examined; labrum punctate, dorsally with median costiform elevation; terminal labial palpomere elongated, its width about one-third its length, about twice as long as penultimate; mentum square-shaped, anterior angles slightly produced, anterior margin with deep median excavation, surface of mentum with sparse setae; stipes triangular, with three short setae; terminal maxillary palpomere elongated, its width about one-third its length, approximately twice as long as penultimate; other mouthparts not examined.

Clypeus (Fig. 113) sub-quadrate, rounded laterally, slightly concave medially, anterior margin slightly carinate, surface mesad to it with dense punctures; frontal stria complete and slightly carinate, slightly outwardly arcuate, continuous with weakly carinate supraorbital stria; frontal disc (Fig. 113) with regular punctation, punctures separated by several times their diameter; eyes slightly flattened, visible from above.

Pronotal sides (Fig. 111) slightly convergent anteriorly, apical angles obtuse; pronotal depressions absent; marginal pronotal stria complete, carinate; disc of pronotum laterally with shallow, moderately dense punctation, punctures become sparser and finer medially; along pronotal base a denser row of ovoid punctures present; pronotal hypomeron with microscopic sparse amber setae; scutellum small, visible.

Elytral humeri not particularly prominent; elytral epipleura with scattered microscopic punctures; marginal epipleural stria complete; marginal elytral stria straight and carinate; apical elytral stria absent. Humeral elytral stria well impressed on basal third; inner subhumeral stria present as a short median fragment; elytral disc with four deeply impressed dorsal elytral striae 1–4, all striae in punctures which are more prominent apically, striae about the same length, somewhat diminishing in length from first to fourth, reaching approximately two-thirds of elytral length apically; fourth elytral stria basally well connected with sutural elytral stria; sutural elytral stria well impressed, almost complete; basal third of elytral disc and elytral flanks only with scattered microscopic punctation, apical two-thirds with much denser and coarser punctation, punctures separated by about twice their diameter, on apical fifth punctation becomes even denser, punctures separated by their own diameter, extreme apex of elytra with a glabrous band.

Propygidium (112) on anterior half almost impunctate, on posterior half with dense punctures separated by about their diameter; pygidium (112) on basal third with punctures separated by about twice their own diameter, punctures becoming sparser and finer apically; extreme apex of pygidium almost impunctate.

Anterior margin of median portion of prosternum (Fig. 114) rounded; prosternal foveae large and deep; marginal prosternal stria present only laterally; prosternal process densely and coarsely punctate; carinal prosternal striae slightly divergent on prosternal apophysis, running parallel, not united apically; lateral prosternal striae carinate, widely united in front of carinal prosternal striae.

Discal marginal mesoventral stria (Fig. 114) anteriorly slightly inwardly arcuate, complete; disc of mesoventrite with deep and dense punctures, separated by about their own diameter, interspaces imbricate; meso-metaventral suture indistinct; meso-metaventral sutural stria bisinuate and undulate, slightly distanced medially from meso-metaventral suture; intercoxal disc of metaventrite on basal three-fourths with scattered fine punctures, in apical third and especially behind metacoxae much larger and deeper punctures present; lateral metaventral stria carinate, almost complete; lateral disc of metaventrite (Fig. 115) slightly excavate, with deep dense punctures of various sizes, apically becoming sparser; metepisternum + fused metepimeron with even denser and coarser setigerous punctures; lateral metepisternal stria present only on fused metepimeron.

Intercoxal disc of first abdominal ventrite almost completely striate laterally; disc laterally and anteriorly with punctures of various sizes, median part of disc almost impunctate, with alutaceous microsculpture.

Protibia (Fig. 116) slightly widening apically, outer margin with seven obtuse teeth topped by prominent denticle, diminishing in size in proximal direction, followed by two minute proximal denticles; setae of outer row thin, sparse, regular; setae of median row even shorter than those of outer row; protarsal groove deep; anterior protibial stria well impressed, bisinuate, shortened apically; protibial spur small, growing out from anterior protibial margin; outer part of posterior surface of protibia (Fig. 117) almost smooth, demarcation line between outer and median of posterior surface marked by double undulate stria, basally that stria with about seven strongly sclerotized setae; median part of posterior protibial surface almost smooth, imbricate, posterior protibial stria complete, fine, apically ending in two short denticles; inner margin with double row of short setae.

Mesotibia slender, outer margin with a row of several thin denticles growing in size apically; setae of outer row strongly sclerotized, about the size of denticles, growing in size apically; setae of median row present medially, much finer and shorter, posterior mesotibial stria not examined; anterior surface of mesotibia with another dense row of short denticles; anterior mesotibial stria straight and complete, terminates in two short denticles; mesotibial spur short; apical margin of mesotibia with three short denticles; claws of apical tarsomere bent, shorter than its half; metatibia (Fig. 118) similar to mesotibia, but denticles on its outer margin even sparser.

**Figures 119–127.**
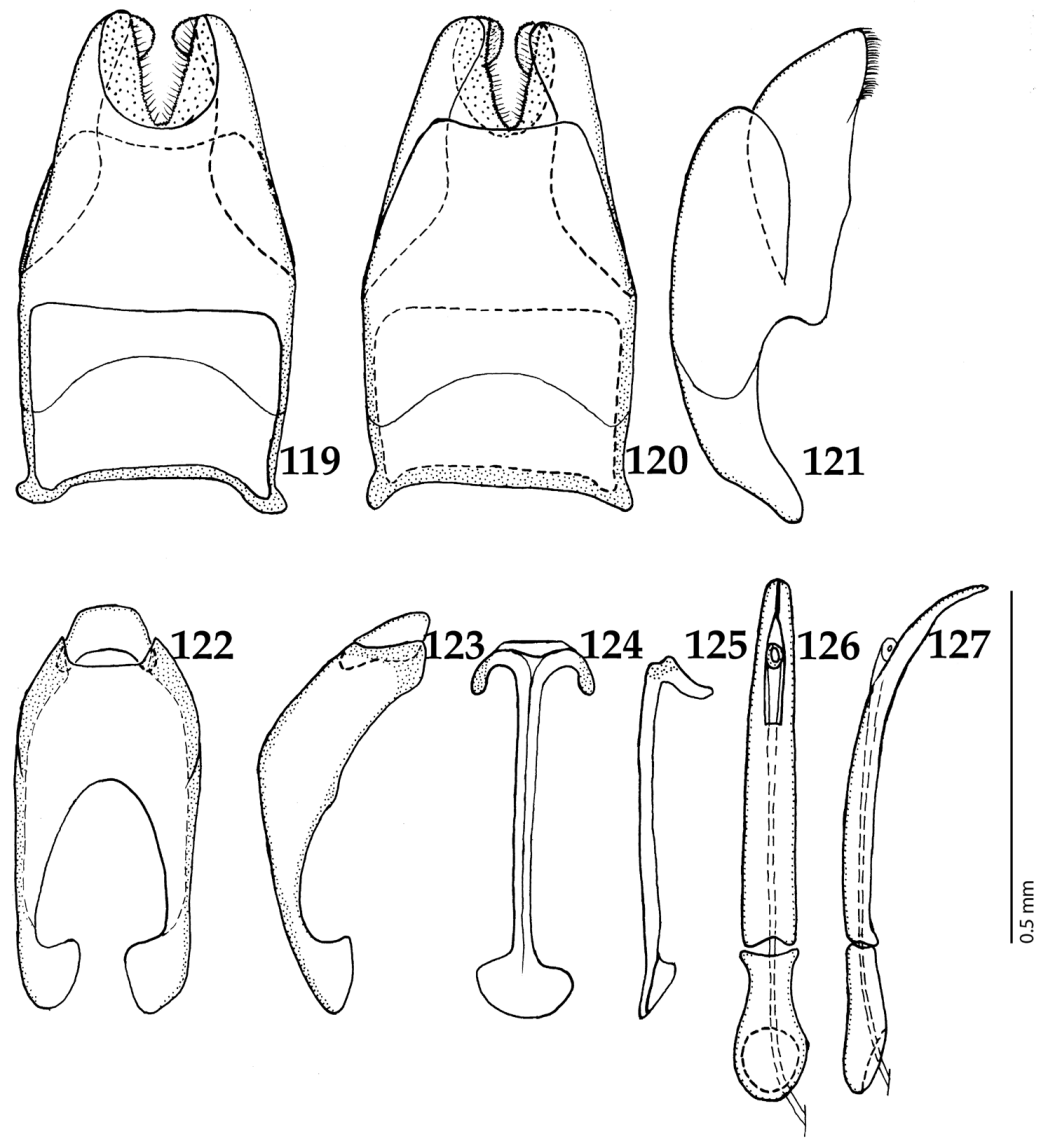
**119** Hypocacculus (Hypocacculus) hyla (Marseul, 1864) male terminalia: 8^th^ sternite + 8^th^ tergite, ventral view **120** ditto, dorsal view **121** ditto, lateral view **122** male terminalia: 9^th^ + 10^th^ tergites, dorsal view **123** ditto, lateral view **124** male terminalia: spiculum gastrale, ventral view **125** ditto, lateral view **126** male terminalia: aedeagus, dorsal view **127** ditto, lateral view.

Male genitalia (based on a male from India: Orissa; see above). Eighth sternite (Figs 119–120) gradually narrowing apically, entirely fused medially; apex of eighth tergite (Fig. 120) shallowly emarginate; apex of eighth sternite with vela covered with dense microscopic setae. Eighth sternite and tergite fused medially (Fig. 121). Ninth sternite (Figs 122–123) typical for the subfamily; tenth tergite (Fig. 122) very small, basally inwardly arcuate. Spiculum gastrale (Figs 124–125) dilated on both ends, its body otherwise parallel-sided; basal end spatula-like. Aedeagus (Figs 126–127) gradually narrowing apically, parameres fused on their basal two-thirds (roughly); basal piece of aedeagus rather short, its ratio to fused parameres approximately 1:3.

#### Hypocaccus

Taxon classificationAnimaliaColeopteraHisteridae

C. Thomson, 1867

Figs 128 , 129–134 , 135–143 , 144 , 145–153 , 154–160 , 161 , 162–173 , 174–182 , 183 , 184–192 , 193–201 , 753 , 755 , 756 , 760

Hypocaccus C. Thomson, 1867: 400. Type species Hister quadristriatus Hoffmann, 1803 (=Hypocaccus rugiceps (Duftschmid, 1805)), designated by Lewis (1899): 3.

##### Diagnosis.

Diagnosis of this genus is based on the taxa that occur in the Australopacific Region. Small to moderately sized, often metallic beetles; frontal stria usually well developed, straight; frons coarsely punctate or with several to multiple short to long transverse rugae. Pronotal disc either smooth (subgenus Baeckmanniolus Reichardt, 1926) or adorned with longitudinal rugae or very coarse punctures (subgenera *Hypocaccus* s.str. or *Nessus* Reichardt, 1932). Pronotum devoid of pronotal depressions, hypomeron asetose; both sets of prosternal striae present, carinal prosternal striae approximate, usually united apically posterad united lateral prosternal striae; prosternal foveae present, often well developed. Protibia on outer margin with 5–11 teeth topped by denticle; metatibia on outer margin with two (*Hypocaccus* s.str., *Nessus*) or three (subgenus Baeckmanniolus) rows of denticles.

**Figure 128.**
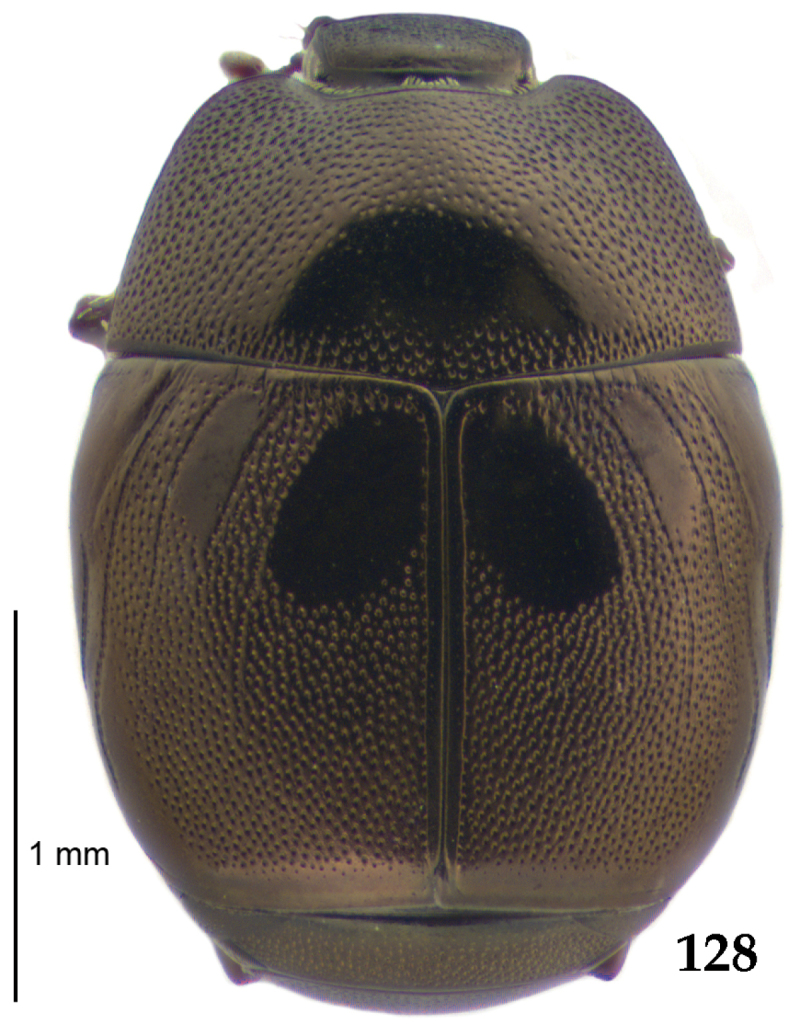
Hypocaccus (Nessus) interpunctatus interpunctatus (Schmidt, 1885) habitus, dorsal view.

##### Biology.

Species of the subgenus Hypocaccus are found on the sandy shores of seas, lakes and rivers (sometimes also on inland sand dunes without the presence of water) where they prey upon dipteran larvae developing in various decomposing organic substances such as excrement, carcasses, seaweed, etc. Species of the subgenus Baeckmanniolus are confined to seashores with similar feeding habits (Lackner 2010).

##### Distribution.

With four recorded species (one of them introduced), the genus *Hypocaccus* is poorly represented in the Australopacific Region and has been collected in Australia, New Caledonia and New Guinea (Figs 753, 755, 756, 760). We would expect the widespread species H. (H.) brasiliensis (see Mazur 2011) or H. (H.) sinae to be eventually collected elsewhere within the Region.

##### Remarks.

Species of the genus *Hypocaccus* are typified by the presence of smooth to rugose frons, which can be also furnished with transverse rugae and cannot be confused with any other Australian Saprininae.

**Key to the subgenera of the genus *Hypocaccus* of the Australopacific Region**

**Table:** 

1(4)	Pronotum (at least laterally) with punctation (Fig. 144); metatibia with two rows of denticles on outer-lateral margin
2(3)	Frons coarsely punctate, with numerous short transverse rugae (Fig. 129)	**subgenus Nessus Reichardt, 1932**
3(2)	Frons smooth, with one to several deep rugae (Fig. 145)	***subgenus Hypocaccus* s. str.**
4(1)	Pronotum (except for row of punctures along base) almost smooth (Fig. 183); metatibia with at least three rows of denticles on outer-lateral margin	**subgenus Baeckmanniolus Reichardt, 1926**

#### Subgenus Nessus

Taxon classificationAnimaliaColeopteraHisteridae

Reichardt, 1932

Figs 128 , 129–134 , 135–143 , 753

Nessus Reichardt, 1932: 61. Type species Saprinus rubripes Erichson, 1834, original designation.

##### Diagnosis.

The single introduced Australian species of the subgenus, H. (N.) interpunctatus interpunctatus can easily be differentiated from the rest of the Australopacific Saprininae by its small size (1.70–2.20 mm), presence of well developed prosternal foveae and a characteristic ‘mirror’ (=polished area) found within the second elytral interval. From the other subgenera this species differs by smaller size and absence of several deep rugae on frontal disc.

**Figures 129–134.**
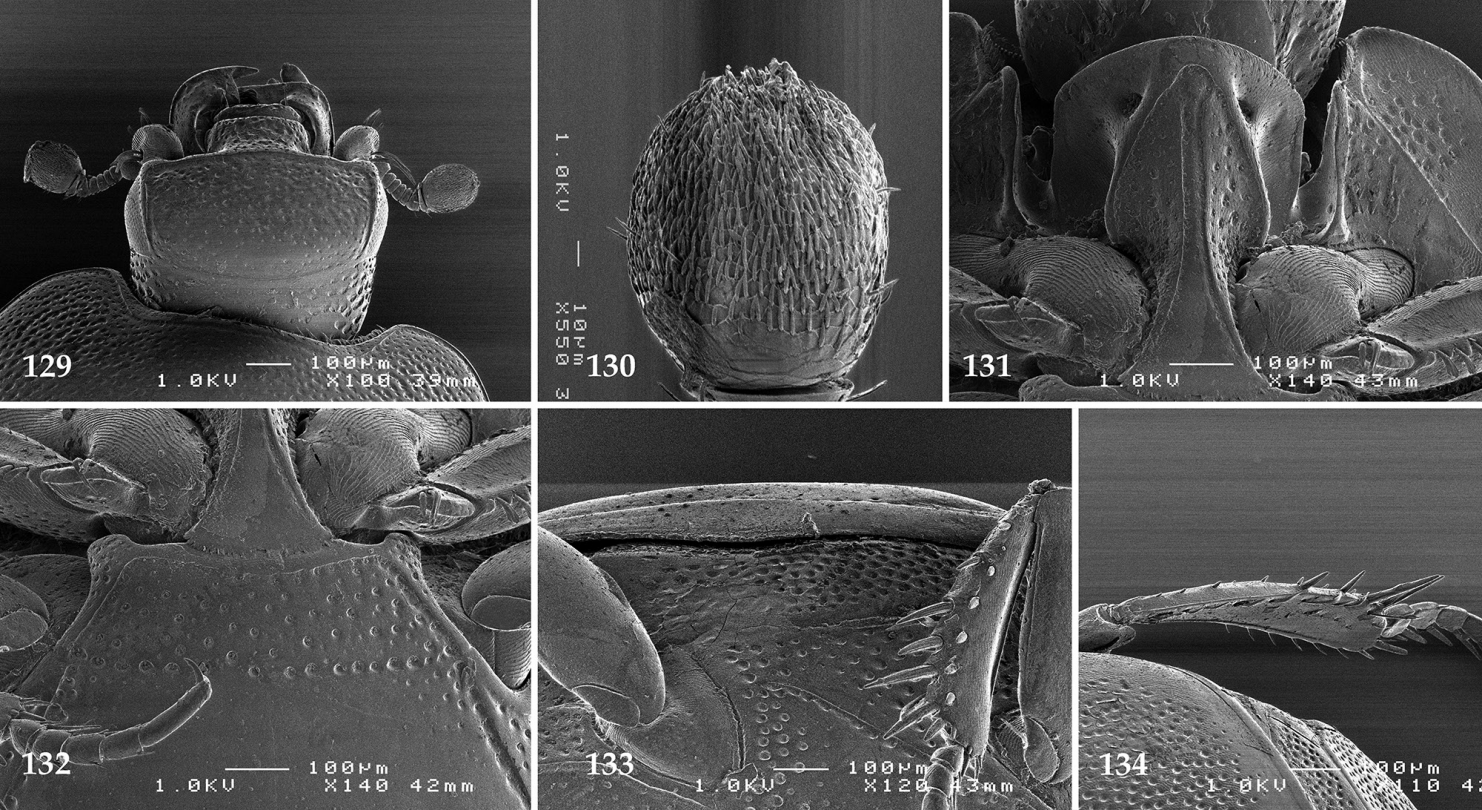
**129** Hypocaccus (Nessus) interpunctatus interpunctatus (Schmidt, 1885) head, dorsal view **130** antennal club, dorsal view **131** prosternum **132** mesoventrite **133** lateral disc of metaventrite + metepisternum, with visible mesotibia **134** metatibia, dorsal view.

##### Biology.

Hypocaccus (Nessus) interpunctatus interpunctatus is found in decaying organic matter, most commonly dung or carrion. Often collected using pitfall traps; occasionally found also under stones on beaches.

##### Distribution.

In the Australopacific Region there is a single introduced species, Hypocaccus (Nessus) interpunctatus interpunctatus recorded from Western Australia (Fig. 753). This species has a scattered distribution: Sicily, Tanzania, Syria, Eritrea, Mozambique, Republic of South Africa, Mongolia and Turkey (Mazur 2011).

##### Remarks.

Mazur (2011) transferred the subgenus Nessus from *Hypocacculus* to genus *Hypocaccus* without comment.

#### Hypocaccus (Nessus) interpunctatusinterpunctatus

Taxon classificationAnimaliaColeopteraHisteridae

(Schmidt, 1885)

Figs 128 , 129–134 , 135–143 , 753

Saprinus interpunctatus Schmidt, 1885: 313.

##### Type locality.

Italy: Sicily.

##### Type material examined.

*Saprinus interpunctatus* Schmidt, 1885: Lectotype, designated by Vienna & Colla in 2003, ♂, glued to a mounting card, with following labels: “Sicilien / Ragusa” (written); followed by: “coll. J. Schmidt” (printed); followed by: “Zool. Mus / Berlin” (printed); followed by: “interpunctat. / Schm. Typ” (written); followed by: “Type” (brick-red label, written); followed by: “interpuncta / tus Schmidt” (double black-margined label, written); followed by: “♂” (written); followed by: “LECTOTYPUS / Hypocacculus (Nessus) / interpunctatus (Schmidt, 1885) / Vienna & Colla des., 2003” (red label, printed) (ZMHUB). Paralectotypes, designated by Vienna & Colla in 2003, 2 ♀♀, one lacks its left hind leg, the other one lacks its left metatibia, with following labels: “Sicilia / Baudi” (written); followed by: “Type” (brick-red label, written); followed by: “Zool. Mus / Berlin” (printed); followed by: “♀” (written); followed by: “PARALECTOTYPUS / Hypocacculus (Nessus) / interpunctatus (Schmidt, 1885) / Vienna & Colla des., 2003” (ZMHUB). Another paralectotype, designated by Vienna & Colla in 2003, ♂, with genitalia extracted and mounted in Canada balsam on a separate slide under the specimen, with the same “Sicilien/Ragusa” label like the lectotype, followed by: “Zool. Mus / Berlin” (printed); followed by: “Saprinus / interpunctatus Schmidt / Coll. Schmidt-Bickhardt” (printed); followed by: “♂” (written); followed by: “PARALECTOTYPUS / Hypocacculus (Nessus) / interpunctatus (Schmidt, 1885) / Vienna & Colla des., 2003” (ZMHUB).

**Figures 135–143.**
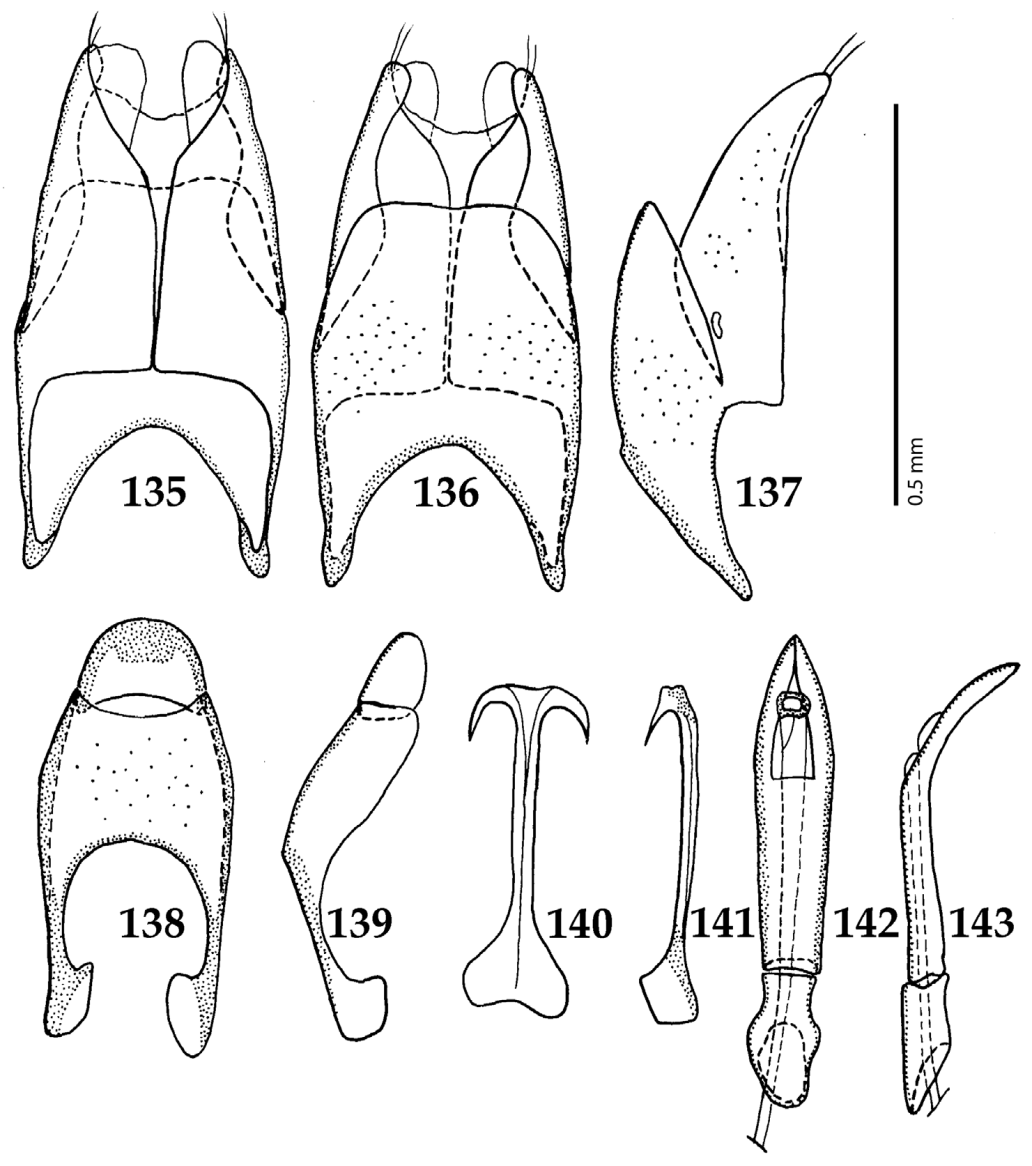
**135** Hypocaccus (Nessus) interpunctatus interpunctatus (Schmidt, 1885) male terminalia: 8^th^ sternite + 8^th^ tergite, ventral view **136** ditto, dorsal view **137** ditto, lateral view **138** male terminalia: 9^th^ + 10^th^ tergites, dorsal view **139** ditto, lateral view **140** male terminalia: spiculum gastrale, ventral view **141** ditto, lateral view **142** male terminalia: aedeagus, dorsal view **143** ditto, lateral view.

##### Additional material examined.

AUSTRALIA. Western Australia: 1 spec., Dardanup, 1.ii.1979, G. Hall (howden trap) (ANIC); 69 specs., 15 km S Busselton, 4.xi.1982, G.P. Hall (ANIC); 30 specs., ditto, but 19.i.1983 (ANIC); 46 specs., ditto, but 9.viii.1983 (ANIC); 1 spec., ditto, but 13.viii.1983 (ANIC); 11 specs., ditto, but 16.vi.1983 (ANIC); 29 specs., ditto, but 12.i.1983 (ANIC); 25 specs., ditto, but 13.i.1983 (ANIC); 26 specs., ditto, but 27.i.1983 (ANIC); 2 specs., ditto, but 27.x.1982 (ANIC); 2 specs., ditto, but 21.x.1982 (ANIC); 4 specs., ditto, but 23.x.1982 (ANIC); 3 specs., ditto, but 11.viii.1983 (ANIC); 3 specs., ditto, but 25.viii.1983 (ANIC); 1 spec., ditto, but 17.viii.1983 (ANIC); 19 specs., ditto, but 15.i.1983 (ANIC); 17 specs., 5 km NE Dardanup, 28.x.1981, G.P. Hall (ANIC); 71 specs., ditto, but 20.x.1981 (ANIC); 20 specs., ditto, but 16.x.1981 (ANIC); 39 specs., ditto, but 4.xi.1982 (ANIC); 9 specs., ditto, but 19.x.1982 (ANIC); 30 specs., ditto, but 27.x.1982 (ANIC); 13 specs., ditto, but 21.x.1982 (ANIC); 5 specs., ditto, but 20.x.1982 (ANIC); 24 specs., ditto, but 23.x.1982 (ANIC); 3 specs., ditto, but 27.x.1982 (ANIC); 8 specs., ditto, but 19.i.1983 (ANIC); 11 specs., ditto, but 27.i.1983 (ANIC); 18 specs., ditto, but 15.i.1983 (ANIC); 6 specs., ditto, but 13.i.1983 (ANIC); 8 specs., ditto, but 12.i.1983 (ANIC); 4 specs., ditto, but 11.i.1983 (ANIC); 16 specs., ditto, but 16.vi.1983 (ANIC); 1 spec., ditto, but 8.vi.1983 (ANIC); 2 specs., ditto, but 11.viii.1983 (ANIC); 6 specs., ditto, but 10.viii.1983 (ANIC); 13 specs., ditto, but 9.viii.1983 (ANIC); 2 specs., ditto, but 14.x.1981 (ANIC); 1 spec., ditto, but 13.x.1981 (ANIC); 1 spec., ditto, but 12.x.1981 (ANIC); 1 spec., ditto, but 1.ii.1979, 33.24S 115.45E, J.D. Majer (ANIC); 4 specs., Rossiter Bay, Cape Le Grand NP, 26.xii.1985, C. Reid (under stones, low heath by beach) (ANIC).

##### Biology.

Saprobiont, collected on carrion and dung alike; several Australian specimens were collected under stones or using traps.

##### Distribution.

Australia: Western Australia (Fig. 753).

##### Remarks.

This species consists of two subspecies: *H. interpunctatus interpunctatus* that is known from the above-mentioned countries and *H. interpunctatus muelleri* Colla, Gomy & Vienna, 2004 occurring in Kenya and southern Ethiopia. The two subspecies differ chiefly in elytral punctation and intervals among striae; furthermore there are differences in the punctation of pygidium and clypeus (Colla, Gomy and Vienna 2014). Hypocaccus (N.) interpunctatus interpunctatus is diagnosed above, as well as provided with a differential diagnosis that separates it from other Australopacific taxa. Most important differences between the Australopacific members of the genus *Hypocaccus* are outlined in the key to the subgenera above. We chose not to fully re-describe Hypocaccus (N.) interpunctatus interpunctatus here, leaving its re-description to the revision of the genus *Hypocaccus*. For the sake of the better species recognition, however, we decided to depict it here, including its male terminalia.

#### Subgenus Hypocaccus

Taxon classificationAnimaliaColeopteraHisteridae

s.str.

Figs 144 , 145–153 , 154–160 , 161 , 162–173 , 174–182 , 755 , 756 , 760

##### Diagnosis.

From the sole Australian representative of the subgenus Nessus, H. (N.) interpunctatus interpunctatus, members of the nominotypical subgenus differ by larger size and presence of several deep rugae on otherwise almost glabrous frontal disc. From the sole Australopacific member of the subgenus Baeckmanniolus, H. (B.) varians varians, the two species of the nominotypical subgenus are easily separated by the presence of punctation on the pronotum.

**Figure 144.**
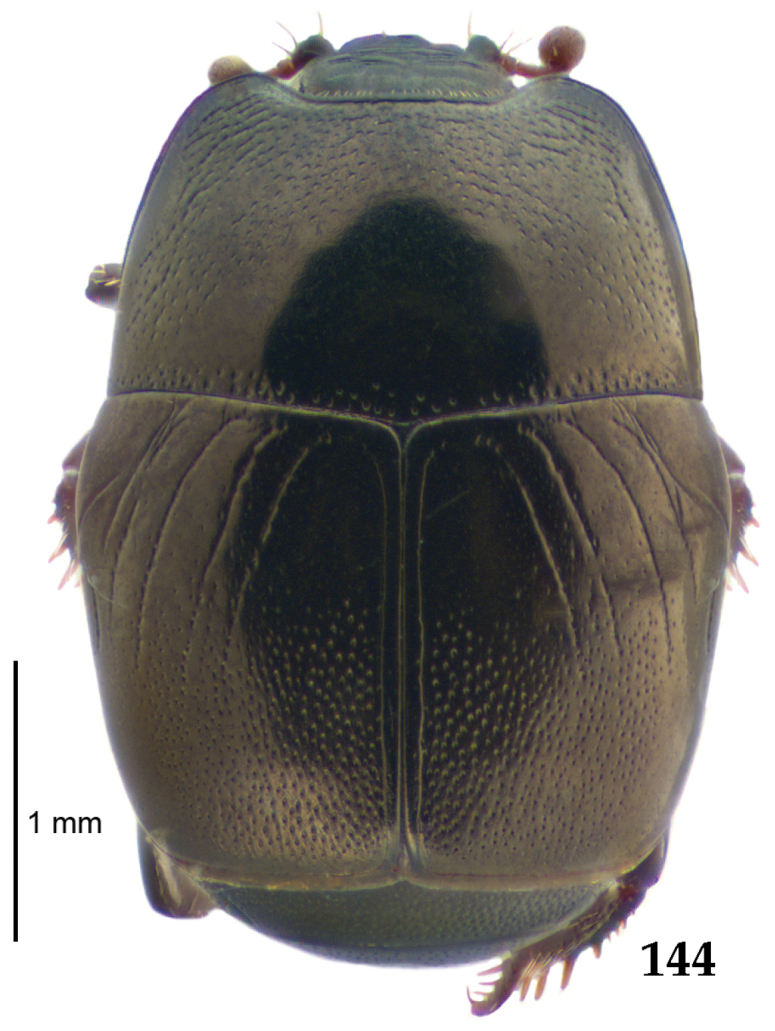
Hypocaccus (Hypocaccus) brasiliensis (Paykull, 1811) habitus, dorsal view.

##### Biology.

Both species of the nominotypical subgenus that occur in Australopacific Region are littoral, occurring under wrack on beach, one specimen of H. (H.) brasiliensis from Australia was also found on a riverbank.

##### Distribution.

Two very similar littoral psammophilous species, H. (H.) brasiliensis and H. (H.) sinae are present in Australopacific Region (Figs 755, 756, 760). Both species are most likely invaders from north (*sensu* Matthews and Bouchard 2008: 17), as they occur in the Palaearctic and Indo-Malaysian Regions and the genus *Hypocaccus* is widely represented there too. The species H. (H.) brasiliensis is tropicopolitan, distributed along beaches in all warmer regions around the globe (Mazur 2011); in Australia it was also found inland on a riverbank of the Colo River (Fig. 756).

##### Remarks.

Regarding the differences between the nominotypical subgenus and subgenus Baeckmanniolus, Bousquet and Laplante (2006: 80, 196) mention another character, the number of rows of denticles on the outer margin of metatibia (two in Hypocaccus s. str. versus three in the subgenus Baeckmanniolus) and treat the subgenus Baeckmanniolus as a distinct genus. For more discussion see Lackner (2010: 145).

##### Key to the species of the subgenus Hypocaccus C. Thomson, 1867 of the genus *Hypocaccus* C. Thomson, 1867 occuring in the Australopacific Region

**Table:** 

1	Elytral punctation with dense alutaceous microsculpture between punctures; elytral punctation sparser, puncures separated by more than their diameter (Fig. 144). Apex of eighth sternite of male genitalia without small setose velum (Fig. 154). Aedeagus gradually broadening apically (Fig. 159)	**Hypocaccus (Hypocaccus) brasiliensis (Paykull, 1811)**
–	Elytral punctation without dense alutaceous microsculpture between punctures; elytral punctation denser, punctures separated by less than their diameter (Fig. 161). Apex of eighth sternite of male genitalia with small setose velum (Fig. 174). Aedeagus tube-like, not particularly broadening apically (Fig. 181)	**Hypocaccus (Hypocaccus) sinae (Marseul, 1862)**

#### Hypocaccus (Hypocaccus) brasiliensis

Taxon classificationAnimaliaColeopteraHisteridae

(Paykull, 1811)

Figs 144 , 145–153 , 154–160 , 755 , 756 , 760

Hister brasiliensis Paykull, 1811: 66.
Saprinus apricarius Erichson, 1834: 194 – Synonymized by Bickhardt (1910): 225.
Saprinus bistrigifrons Marseul, 1855: 729 – Synonymized by Mazur (1987): 32.
Saprinus dentipes Marseul, 1855: 728 – Synonymized by Mazur (1987): 32.
Saprinus piscarius Blackburn, 1903: 108 – Synonymized by Mazur (1987): 32.

##### Type locality.

Brazil.

##### Type material examined.

*Hister brasiliensis* Paykull, 1811: Lectotype, ♀, designated by Dahlgren in 1968, together with extracted genitalia glued to a rectangular mounting card, right foreleg missing, right elytron missing, right metatibia broken off, glued next to the specimen, with the following labels: “24” (pencil-written); followed by: “Uppsala Univ. Zool. Mus. / Gyllenhals saml. TYP nr. / 1153” (red label, printed); followed by: “LECTOTYP / HYPOCACCUS / BRASILIENSIS / PAYK. G. DAHL- / GREN 27.II.1968” (written in black ink) (UUZM).

**Figures 145–153.**
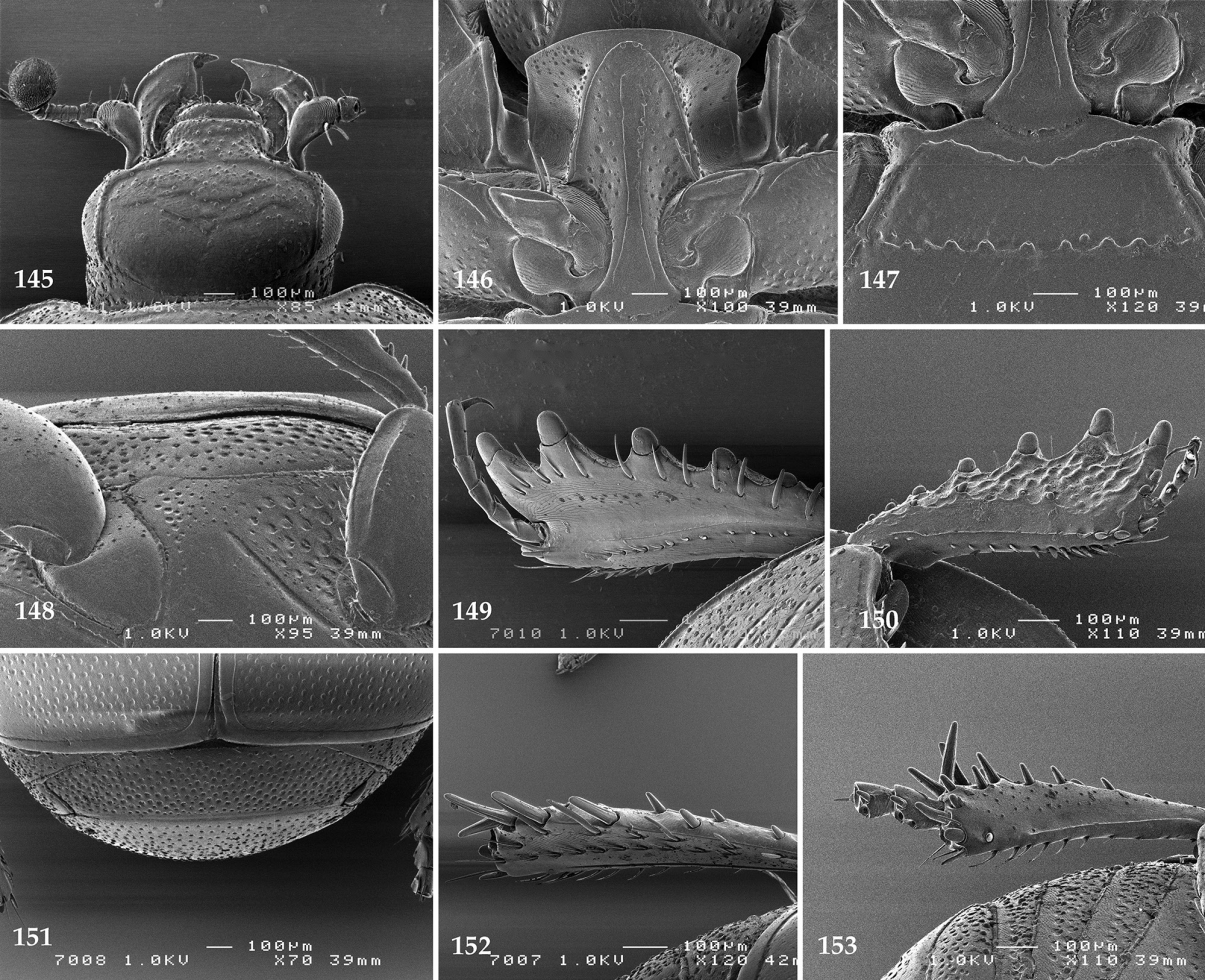
**145** Hypocaccus (Hypocaccus) brasiliensis (Paykull, 1811) head, dorsal view **146** prosternum **147** mesoventrite **148** lateral disc of metaventrite + metepisternum **149** protibia, dorsal view **150** ditto, ventral view **151** propygidium + pygidium **152** metatibia, dorsal view **153** ditto, ventral view.

*Saprinus apricarius* Erichson, 1834: Lectotype, present designation: most likely a ♀, pinned, left hind leg missing, with the following labels: “Aegypt / xxxxiv Er.(?)” (dark green label, written); followed by: “49233” (printed); followed by: “Hist. -Coll. (Coleoptera) / Nr. 49233 / Saprinus apricarius Er. x / Aegypt., Ehrenberg / Zool. Mus. Berlin” (black-framed label, printed); followed by: “Saprinus / apricarius / Erichson, 1834 / LECTOTYPE 2014 / des. T. Lackner” (red label, written) (ZMHUB). Paralectotypes, present designation: 1 spec., possibly a ♀, pinned, with the following label: “Hist. -Coll. (Coleoptera) / Nr. 49233 / Saprinus apricarius Er. x / Aegypt., Ehrenberg / Zool. Mus. Berlin” (black-framed label, printed); followed by: “Saprinus / apricarius / Erichson, 1834 / PARALECTOTYPE 2014 / des. T. Lackner” (red label, written) (ZMHUB). 1 spec., possibly a ♀, pinned, with the following labels: “49233” (printed); followed by: “Hist. -Coll. (Coleoptera) / Nr. 49233 / Saprinus apricarius Er. x / Sicil, Dahl / Zool. Mus. Berlin” (black-framed label, printed); followed by: “apricarius / Er. / Sicil. Dahl.” (black-framed label, written); followed by: “Saprinus / apricarius / Erichson, 1834 / PARALECTOTYPE 2014 / des. T. Lackner” (red label, written) (ZMHUB). 1 spec., possibly a ♀, pinned, with the following labels: “47”; followed by: “Hist. -Coll. (Coleoptera) / Nr. 49233 / Saprinus apricarius Er. x / Sicil, Dahl / Zool. Mus. Berlin” (black-framed label, printed); followed by: “apricarius / Er. / Sicil. Dahl.” (black-framed label, written); followed by: “Saprinus / apricarius / Erichson, 1834 / PARALECTOTYPE 2014 / des. T. Lackner” (red label, written) (ZMHUB). 1 spec., pinned, with the following labels: “54”; followed by: “Hist. -Coll. (Coleoptera) / Nr. 49233 / Saprinus apricarius Er. x / Sicil, Dahl / Zool. Mus. Berlin” (black-framed label, printed); followed by: “apricarius / Er. / Sicil. Dahl.” (black-framed label, written); followed by: “Saprinus / apricarius / Erichson, 1834 / PARALECTOTYPE 2014 / des. T. Lackner” (red label, written) (ZMHUB). 2 specs., pinned, with the following labels: “Hist. -Coll. (Coleoptera) / Nr. 49233 / Saprinus apricarius Er. x / Sicil, Dahl / Zool. Mus. Berlin” (black-framed label, printed); followed by: “apricarius / Er. / Sicil. Dahl.” (black-framed label, written); followed by: “Saprinus / apricarius / Erichson, 1834 / PARALECTOTYPE 2014 / des. T. Lackner” (red label, written) (ZMHUB). This species has been described from both Sicily and Egypt, among the type series we designate a single female specimen from Egypt as lectotype to fix the taxonomic identity of the species.

*Saprinus bistrigifrons* Marseul, 1855: Lectotype, present designation: ♂, pinned, right mesotarsus missing, with following labels: tiny pink label, followed by: “161 / Saprinus / bistrigifrons / m. / Mexique / illegible text” (green, round label, written); followed by: “MUSEUM PARIS / COLL. / DE MARSEUL 1890” (green, printed label); followed by: “TYPE” (red-printed label); followed by: “Saprinus / bistrigifrons / Marseul, 1855 / LECTOTYPE 2014 / des. T. Lackner” (red label, written) (MNHN). This species has been described from unknown number of specimens and the lectotype designation fixes the species identity.

*Saprinus dentipes* Marseul, 1855: Lectotype, present designation: probably a ♂, pinned, left metatarsus and right hind leg missing, with the following labels: “160 / Saprinus / dentipes / illegible text / Mexique / illegible text” (round, green label, written); followed by: “MUSEUM PARIS / COLL. / DE MARSEUL 1890” (green, printed label); followed by: “TYPE” (red-printed label); followed by: “Saprinus dentipes / Marseul, 1855 / LECTOTYPE 2014 / des. T. Lackner” (red label, written) (MNHN). This species has been described from unknown number of specimens and the lectotype designation fixes the species identity.

*Saprinus piscarius* Blackburn, 1903: Cotype, sex unidentified, with printed label: “Australia / Blackb’s Coll” (printed), followed by “*Hypocaccus* / *vernulus* Blackb” (written), followed by “*piscarius* Bl. Cot. / *vernulus* placed with Cotype / *piscarius* in the Bl. Coll” (written), followed by “SAMA Database / No. 25-029592” (printed); 1 spec., “*Hypo. piscarius* / co-type” (written), followed by “SAMA Database / No. 25-029591 (printed)”; 1 spec., “*Saprinus* / *piscarius* / Cotype” (written), followed by “SAMA Database / No. 25-029590” (written) (SAMA). This species was described from ‘near Adelaide’, as well as ‘Also from Victoria (Frankston, Mr. Kershaw)’ (Blackburn, 1903: 108). Because of the ambiguous label data (see above) as well as no locality indication of the cotype we decided not to designate a lectotype of this species.

**Figures 154–160.**
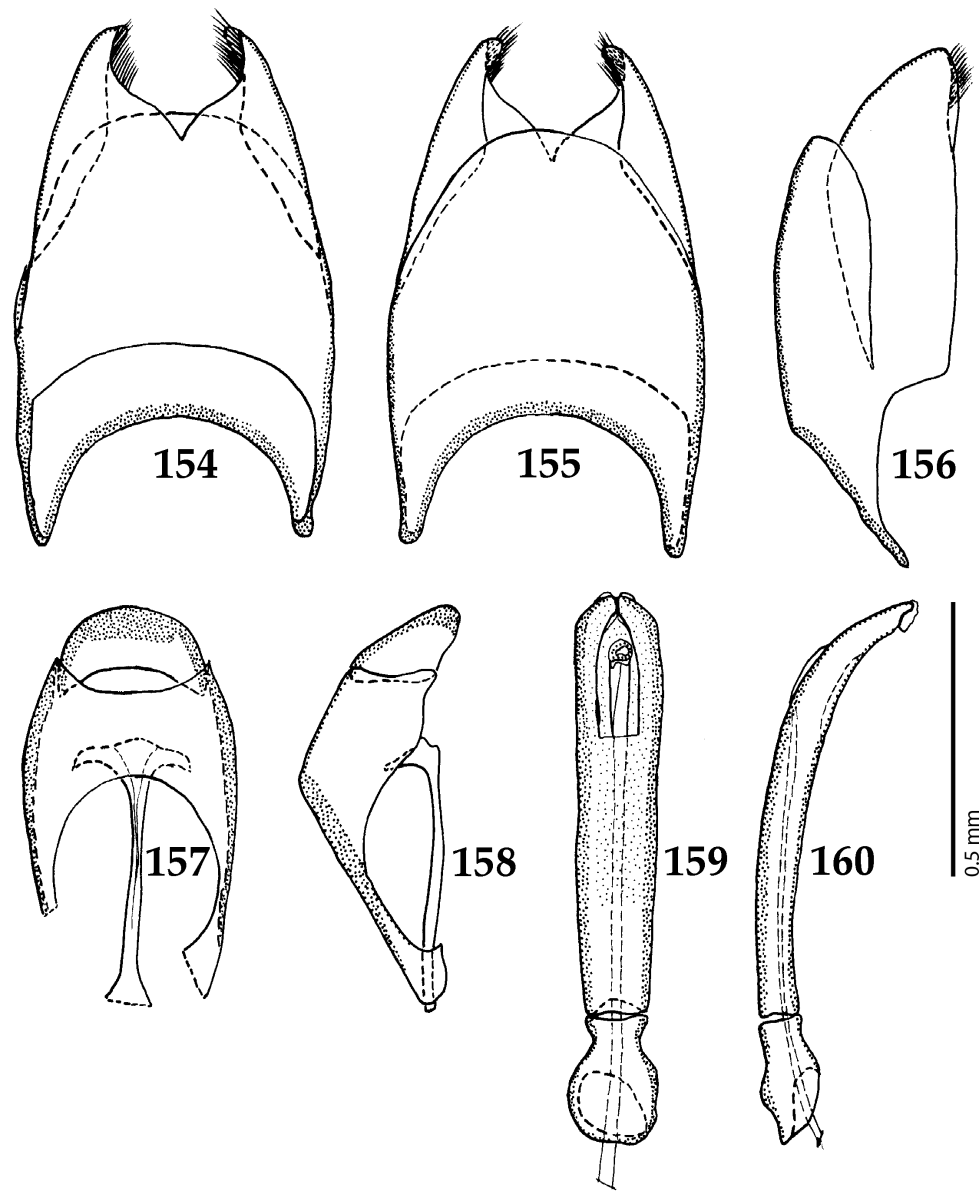
**154** Hypocaccus (Hypocaccus) brasiliensis (Paykull, 1811) male terminalia: 8^th^ sternite + 8^th^ tergite, ventral view **155** ditto, dorsal view **156** ditto, lateral view **157** male terminalia: 9^th^ + 10^th^ tergites, dorsal view; spiculum gastrale, ventral view **158** male terminalia: 9^th^ + 10^th^ tergites; spiculum gastrale, lateral view **159** male terminalia: aedeagus, dorsal view **160** ditto, lateral view.

##### Additional material examined.

NEW CALEDONIA. 1 ♂ & 4 specs., Isle Atire, 1.ix.1982, T. Lovegrove (ex dead white-capped noddy) (AMNZ).

New Guinea. New Britain: 12 specs., Ralum, F. Dahl S. (ZMHUB; 2 specs. in coll TLAN).

AUSTRALIA. New South Wales: 2 specs., Tomakin, 35.49S 150.11E, 25.xii.1988, W. Dressler (ex dead shark head) (ANIC); 1 spec., Colo River, Near Colo, 8.xii.1979, D.P. Came (on river sands) (ANIC); 2 specs., Sydney, K.K. Spence (ANIC); 1 spec., Sydney, Lea (SAMA). South Australia: 4 specs., Adelaide, Griffith (SAMA); 1 spec., Marino Rook: Spelhore, 4.–5.i.1978, G. Szelényi leg. (HMNH). Western Australia: 2 specs., Geraldton (SAMA).

##### Remarks.

*Saprinus dentipes* has been synonymized with H. (H.) brasiliensis by Mazur (1987). However, upon inspecting its type specimen we conclude that it is not synonymous with H. (H.) brasiliensis, but with the species Hypocaccus (Baeckmanniolus) gaudens (J.L. LeConte, 1851) instead. Thus *Saprinus dentipes* Marseul, 1855 = Hypocaccus (Baeckmanniolus) gaudens (J.L. LeConte, 1851) syn. n. H. (H.) brasiliensis is a widespread and variable species with variable dorsal punctation. The taxonomic status of both H. (H.) brasiliensis and the following H. (H.) sinae requires further clarification, especially with respect to their rather similar genitalia and minimal differences in dorsal sculpture. Hypocaccus (H.) brasiliensis is above included in the key to the Australopacific species of *Hypocaccus* that separates it from the related Hypocaccus (H.) sinae. We chose not to fully re-describe Hypocaccus (H.) brasiliensis here, leaving its re-description to the revision of the genus *Hypocaccus*. For the sake of the better species recognition, however, we decided to depict it here, including its male terminalia.

#### Hypocaccus (Hypocaccus) sinae

Taxon classificationAnimaliaColeopteraHisteridae

(Marseul, 1862)

Figs 161 , 162–173 , 174–182 , 756

Saprinus sinae Marseul, 1862: 496.
Saprinus vernulus Blackburn, 1903: 108 – **syn. n.**

##### Type locality.

China: Shanghai.

**Figure 161.**
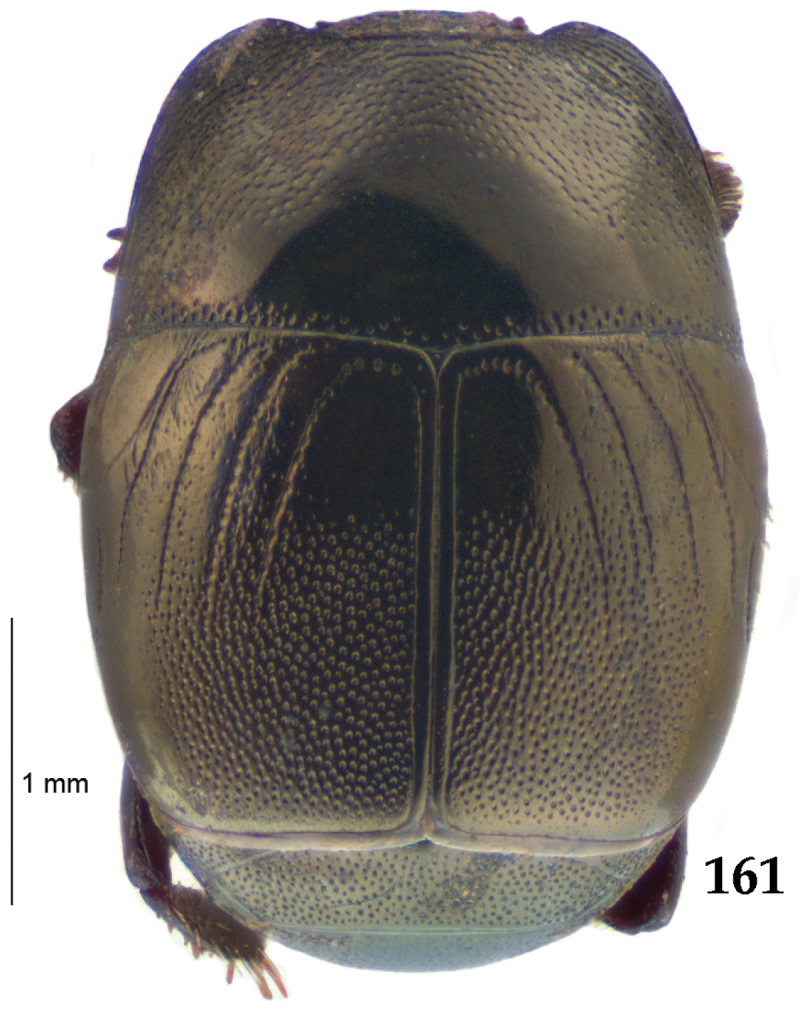
Hypocaccus (Hypocaccus) sinae (Marseul, 1862) habitus, dorsal view.

##### Type material examined.

*Saprinus sinae* Marseul, 1862: Lectotype, present designation: ♂, mounted on tip of mounting card, with genitalia extracted, dismembered and placed in a separate plastic slide under the specimen, right antennal club, right protarsus and right hind-leg missing, with the following labels: tiny, dark blue rectangular label, followed by: “Saprinus / Sinae m. / Shanghai / Dej. 63” (round, yellow label, written); followed by: “Shanghai” (written); followed by: “52 (158a) Saprinus / Sinae m.60 / Shanghai” (written); followed by: “MUSEUM PARIS / COLL. / DE MARSEUL 1890” (printed); followed by: “TYPE” (red-printed label); followed by: “Saprinus sinae / Marseul, 1862 / LECTOTYPE 2014 / des. T. Lackner” (red label, written) (MNHN). This species was described from unknown number of specimens and the lectotype designation fixes the identity of the species.

**Figures 162–173.**
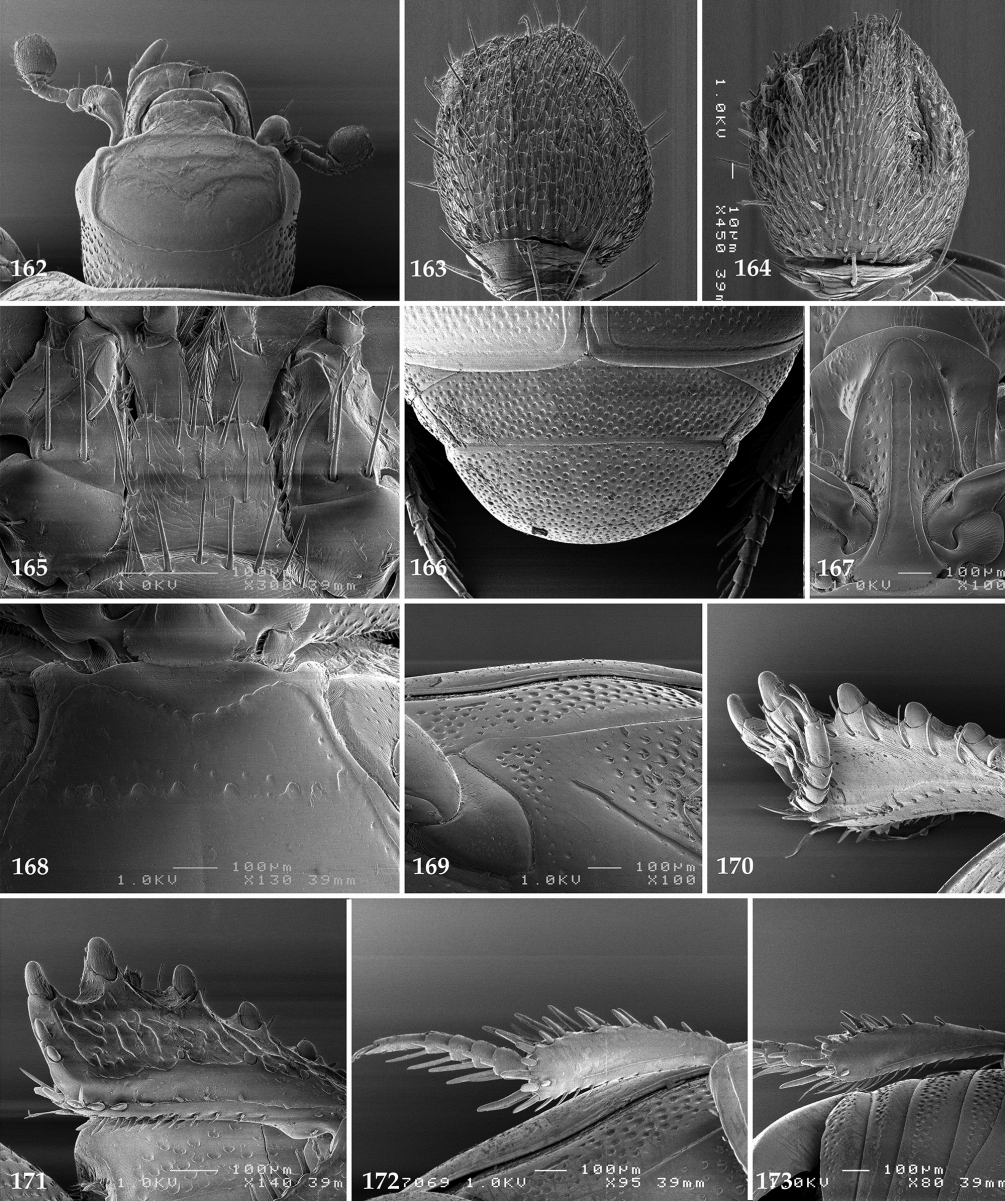
**162** Hypocaccus (Hypocaccus) sinae (Marseul, 1862) head, dorsal view **163** antennal club, dorsal view **164** ditto, ventral view **165** mentum, ventral view **166** propygidium + pygidium **167** prosternum **168** mesoventrite **169** lateral disc of metaventrite + metepisternum **170** protibia, dorsal view **171** ditto, ventral view **172** mesotibia, ventral view **173** metatibia, ventral view.

*Saprinus vernulus* Blackburn, 1903: Lectotype, present designantion: ♀, mounted on a card with “♀” (printed), followed by: “7249” (mount card with a red number, printed); followed by: “N.S. Wales” (printed); followed by: “K. 270214” (printed); followed by: “*Hypocaccus* / *vernulus* / Cotype” (written); followed by: “*Hypocaccus* / *vernulus* Bl.” (written); followed by: “SYNTYPE” (yellow label, written); followed by: “Saprinus vernulus / Blackburn, 1903 / LECTOTYPE des. / T. Lackner 2016” (red label, written) (AMS). This specimen has been described based on unknown number of specimens and the lectotype designation fixes the identity of the species.

**Figures 174–182.**
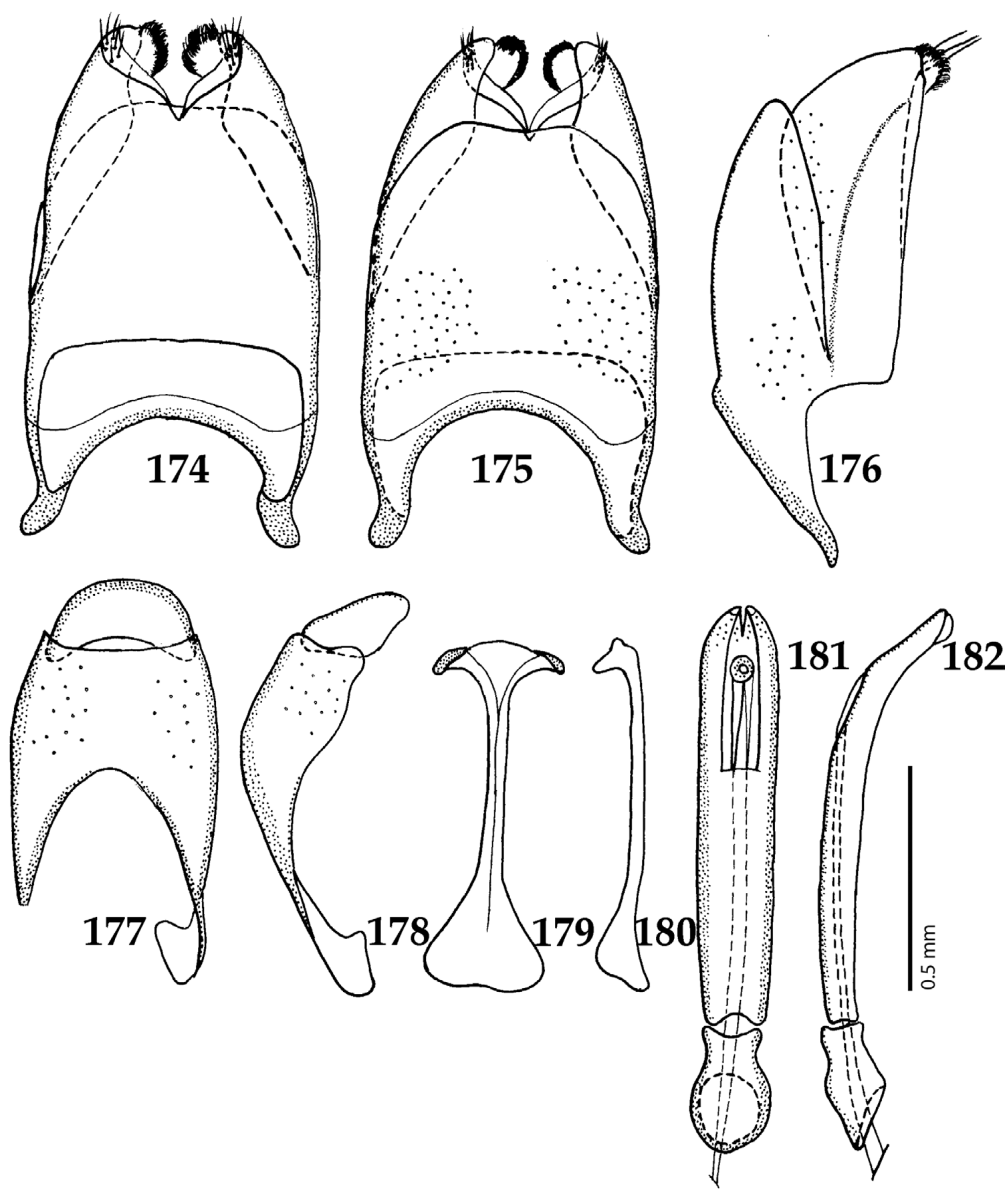
**174** Hypocaccus (Hypocaccus) sinae (Marseul, 1862) male terminalia: 8^th^ sternite + 8^th^ tergite, ventral view **175** ditto, dorsal view **176** ditto, lateral view **177** male terminalia: 9^th^ + 10^th^ tergites, dorsal view **178** ditto, lateral view **179** male terminalia: spiculum gastrale, ventral view **180** ditto, lateral view **181** male terminalia: aedeagus, dorsal view **182** ditto, lateral view.

##### Additional material examined.

AUSTRALIA. New South Wales: 2 specs., re-mounted on a single pin, with the same labels as the lectotype, (and certainly from the same collection – D. Smith pers. comm.) except the original mount with red number “7248” instead of “7249” and with “K270229” instead of “K. 270214” of the lectotype (AMS). 2 ♂♂, without further data (MAMU); 9 specs., New South Wales, without further data (SAMA); 3 specs., Sydney, Lea (SAMA). South Australia: 3 specs., Adelaide, Griffith (SAMA).

##### Biology.

A typical littoral psammophile found on beaches.

##### Distribution.

Described from Shanghai (China), distributed along the coasts of China, Indonesia, Russian Far East, Thailand, Vietnam, Cambodia, Myanmar, Japan, Korea, Malaysia, Australia (Mazur 2011; present paper). Reported also from Afghanistan by Thérond (1969) together with the preceding species. From the Australopacific Region found only in Australia (New South Wales and South Australia; Fig. 756).

##### Remarks.

Blackburn (1903: 108) mentioned ‘distinctly green colour in combination with elytra having a very strong external subhumeral stria’ as the main distinguishing characters between *H. vernulus* and *H. sinae*. Since the external morphological characters of many Saprininae species are known to be intra-specifically rather variable, we assign the characters used by Blackburn for the separation of *H. vernulus* from *H. sinae* as intra-specific variation and synonymize the two species. A single syntype of *Saprinus vernulus* Blackburn, 1903 (currently Hypocaccus (Hypocaccus) vernulus) housed at ANIC has been inspected and we can herewith conclude that it is conspecific with the species Hypocaccus (Hypocaccus) sinae (Marseul, 1862); thus Hypocaccus (Hypocaccus) vernulus Blackburn, 1903 = Hypocaccus (Hypocaccus) sinae (Marseul, 1862) syn. n. Hypocaccus (H.) sinae is above included in the key to the Australopacific species of *Hypocaccus* that separates it from the related Hypocaccus (H.) brasiliensis. We chose not to fully re-describe Hypocaccus (H.) sinae here, leaving its re-description to the revision of the genus *Hypocaccus*. For the sake of the better species recognition, however, we decided to depict it here, including its male terminalia.

#### Subgenus Baeckmanniolus

Taxon classificationAnimaliaColeopteraHisteridae

Reichardt, 1926

Figs 183 , 184–192 , 193–201 , 756

Baeckmanniolus Reichardt, 1926: 14. Type species Hister dimidiatus Illiger, 1807; original designation.

##### Diagnosis.

The single Australopacific taxon, H. (B.) varians varians can be separated from other congeners by almost smooth pronotum that bears fine punctation in the apical pronotal angles.

**Figure 183.**
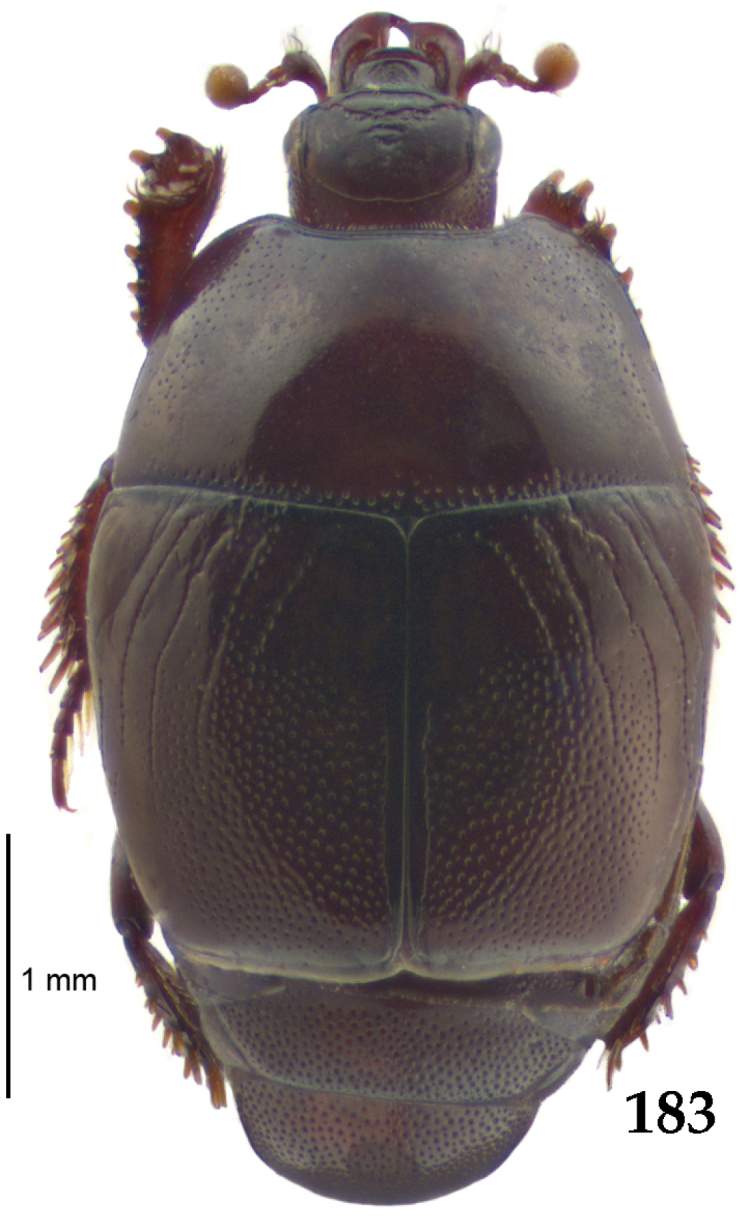
Hypocaccus (Baeckmanniolus) varians varians (Schmidt, 1890) habitus, dorsal view.

##### Biology.

H. (B.) varians varians is a typical littoral psammophilous taxon, found on the beach under dead fish, algae or coastal wrack.

##### Distribution.

In the Australopacific Region there is only one species present, H. (B.) varians varians, recorded from Western Australia, Northern Territory and Queensland.

#### Hypocaccus (Baeckmanniolus) variansvarians

Taxon classificationAnimaliaColeopteraHisteridae

(Schmidt, 1890)

Figs 183 , 184–192 , 193–201 , 756

Saprinus varians Schmidt, 1890: 55.

##### Type locality.

Japan.

##### Type material examined.

*Saprinus varians* Schmidt, 1890: Lectotype, present designation: ♂, glued on the mounting card, with the following labels: “Japan” (written); followed by: “Type” (brick-red label, printed); followed by: “coll. J. Schmidt” (printed); followed by: “varians / m.” (black-yellow margined label, written); followed by: “Saprinus / varians Schmidt / Coll. Schmidt-Bickhardt” (printed); followed by: “Saprinus varians / Schmidt, 1890 / LECTOTYPE 2014 / des. Lackner & Leschen” (red label, written) (ZMHUB). Paralectotypes, present designation: 4 specs., probably ♀♀, carrying the same hand-written labels “Japan” and “Type” and the printed label “Saprinus / varians Schmidt / Coll. Schmidt-Bickhardt”, followed by the paralectotype labels identical to that of the lectotype (ZMHUB). This species has been described from unknown number of specimens and the lectotype designation fixes the taxonomic identity of the species.

**Figures 184–192.**
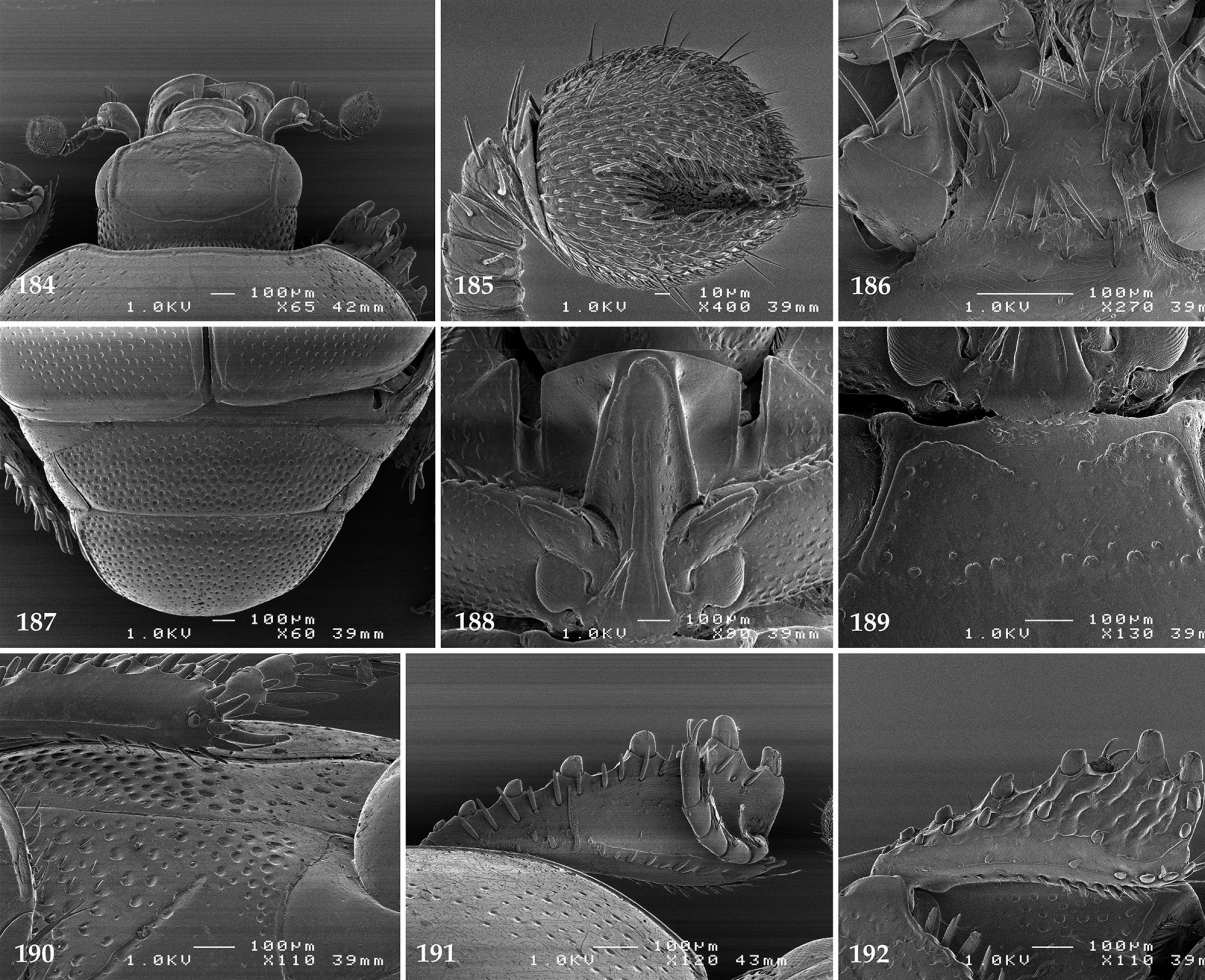
**184** Hypocaccus (Baeckmanniolus) varians varians (Schmidt, 1890) head, dorsal view **185** antennal club, ventral view **186** mentum, ventral view **187** propygidium + pygidium **188** prosternum **189** mesoventrite **190** lateral disc of metaventrite + metepisternum **191** protibia, dorsal view **192** ditto, ventral view.

##### Additional material examined.

AUSTRALIA. Western Australia: 1 spec., Monte Bello Island (ANIC). Northern Territory: 1 ♂ & 1 ♀, Groote Eylandt, N.B. Tindale (SAMA). Queensland: 1 ♀, Cairns (SAMA).

##### Biology.

See above.

**Figures 193–201.**
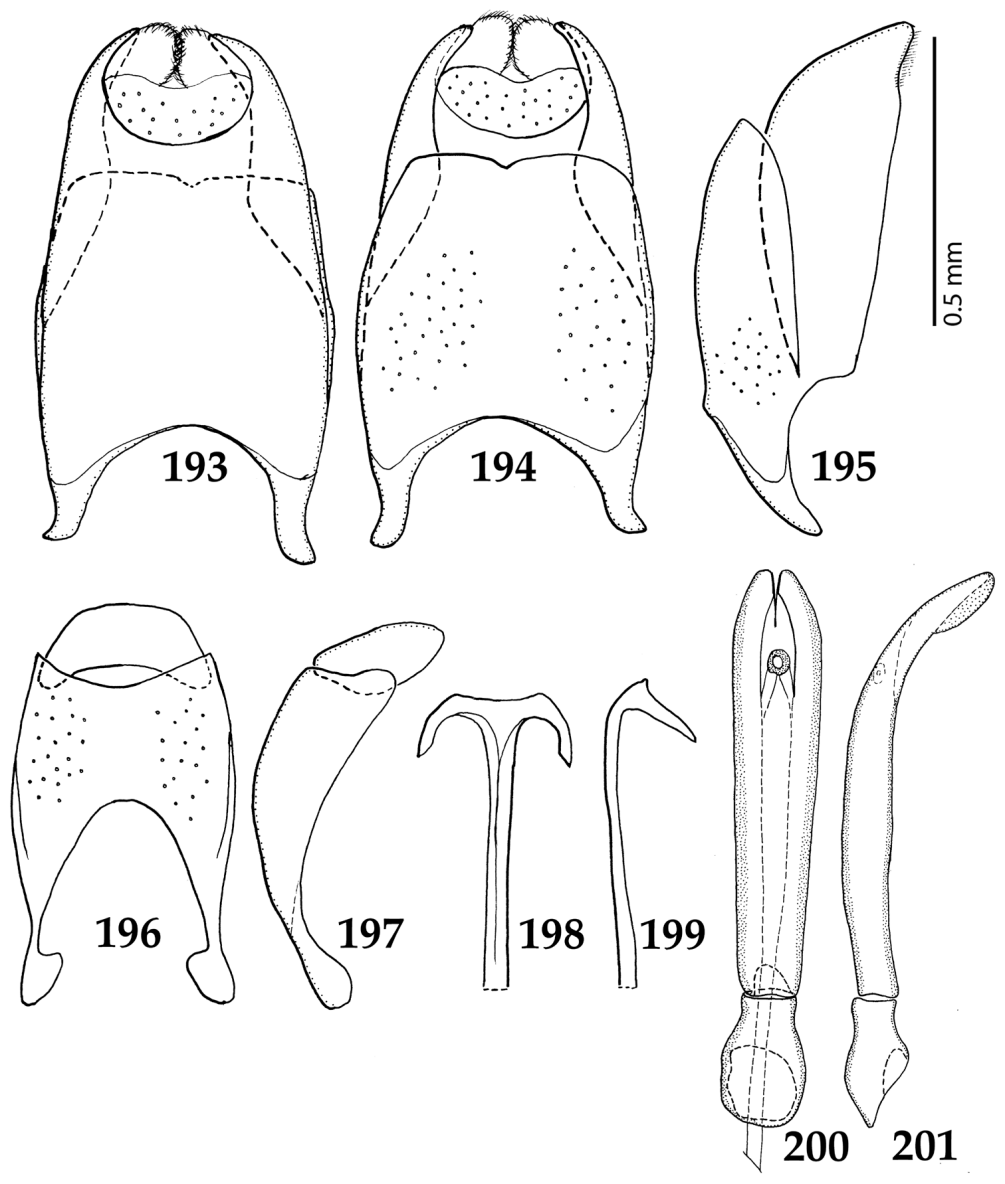
**193** Hypocaccus (Baeckmanniolus) varians varians (Schmidt, 1890) male terminalia: 8^th^ sternite + 8^th^ tergite, ventral view **194** ditto, dorsal view **195** ditto, lateral view **196** male terminalia: 9^th^ + 10^th^ tergites, dorsal view **197** ditto, lateral view **198** male terminalia: spiculum gastrale, ventral view **199** ditto, lateral view **200** male terminalia: aedeagus, dorsal view **201** ditto, lateral view.

##### Distribution.

Described from Japan, the nominotypical subspecies is found also on Sakhalin Island, Taiwan, Philippines, Vietnam, Sri Lanka, and Australia. The two other subspecies are H. (B.) varians hatsune Nakane, 1977, described from the Ogasawara Islands (southern Japan) and H. (B.) varians continentalis Reichardt, 1941 described from Russian Far East (Primorsky Kray) and found also in eastern China (Shandong) (Mazur 2011).

##### Remarks.

The species H. (B.) varians has a faint pronotal punctation indicating that it should be transferred into the nominotypical subgenus Hypocaccus. According to Bousquet and Laplante (2006; see above) the outer margin of metatibia of the subgenus Baeckmanniolus has three rows of denticles instead of two rows present in *Hypocaccus*. The metatibia of Hypocaccus (B.) varians possesses two rows on the metatibia supplemented by another 2 (occasionally 3) denticles between the two rows. The presence of vague and weakened pronotal punctation and by possessing denticles between the two rows on the outer margin of metatibia, this species represents a transitional form between the two subgenera and its exact taxonomic position should be determined by a thorough morphological study of all known species of *Hypocaccus* s. l. Hypocaccus (B.) varians varians is diagnosed above, as well as provided with a differential diagnosis that separates it from other Australopacific taxa. Most important differences between the members of the genus *Hypocaccus* are outlined in the key to the subgenera above. We chose not to fully re-describe Hypocaccus (B.) varians varians here, leaving its re-description to the revision of the genus *Hypocaccus*. For the sake of the better species recognition, however, we decided to depict it here, including its male terminalia.

#### Iridoprinus gen. n.

Taxon classificationAnimaliaColeopteraHisteridae

http://zoobank.org/EB713AF8-076C-4EBD-B299-7B1932618AF8

Figs 202 , 203–211 , 212–218 , 757

##### Type species.

*Iridoprinus myrmecophilus* sp. n.

**Figure 202.**
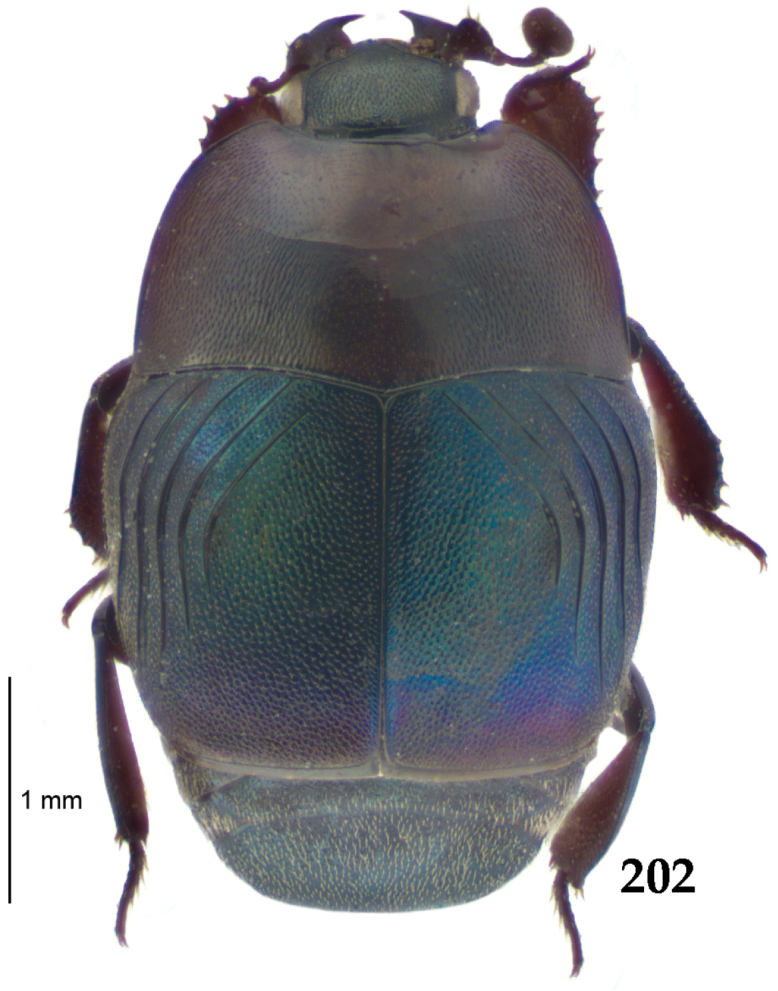
*Iridoprinus myrmecophilus* sp. n., habitus, dorsal view.

##### Diagnosis.

Cuticle light brown, elytra with strong blue iridescent metallic luster; frontal stria prolonged far onto clypeus; antennal club large, oval and depressed dorso-ventrally; pronotal disc on apical two-thirds coriarious-punctate, punctures forming elongate wrinkles, confluent; elytra densely imbricate-punctate, punctures with microscopic setae; five dorsal elytral striae present, curved and carinate; inner subhumeral and sutural elytral striae absent; abdominal segments dorsally with microscopic setae; prosternal foveae absent; metepisternum with deep elongate groove for reposing mesotarsus; pro- and mesotibiae slightly dilated.

##### Biology.

The paratype has been collected in the nest of Meat Ant *Iridomyrmex purpureus* (Smith, 1858). Based on the collecting circumstances of the paratype, as well as its morphological characters (dilated tibiae, metepisternum with groove for reposing mesotarsus) we presume that the newly described taxon is a myrmecophile.

##### Distribution.

Endemic to Australia; known from Northern Territory and New South Wales (Fig. 757).

##### Etymology.

The generic epithet is created by combining part of the name of the host ant ‘*Iridomyrmex*’ with part of the word *Saprinus* (- *prinus*); the specific epithet of this new taxon relates to its apparently myrmecophilous biology.

##### Remarks.

By the presence of five strongly curved and carinate dorsal elytral striae and absence of inner subhumeral and sutural striae and presence of peculiar deep longitudinal groove on the metepisternum for the accommodation of mesotarsus, this myrmecophilous Australian genus cannot be confused with any other currently known higher taxon of Saprininae. This is a highly autapomorphic genus (Lackner 2014d), though, due to the paucity of material, critical characters from the antenna and mouthparts were not examined. However, this is the only Saprininae from the Australopacific and Indo-Malayan Regions collected from ant nests, indicating that it may be an obligate myrmecophile (for the outline of ant-inquilinous Saprininae see Lackner 2017). The deep longitudinal groove of the metepisternum to accommodate the mesotarsus while retracted as well as setose body (the microsetae are presumed to exude appeasing liquid substances which are licked by ants), dilated tibiae that probably serve to cover larger space of the venter when retracted, are possible adaptations to life inside ant nests. Its role inside the ant community is unknown, and it does not have obvious trichomes as in other obligate inquilines such as Chlamydopsinae. This taxon was included in the published phylogeny of the subfamily by the senior author (Lackner 2014d) under the name ‘Saprininae gen. n. (Australia)’. In the published cladogram it was recovered as sister to another Australian taxon of unknown biology (*Saprinodes falcifer* Lewis, 1891); this relationship was supported by one ‘strong’ and two ‘weaker’ synapomorphies. It is interesting to note that another apparent myrmecophile, Euspilotus (Platysaprinus) latimanus (Schmidt, 1890) has been recovered sister to this clade, but this purported monophyly should be regarded as spurious since it might be based on homoplasy rather than homology (the characters supporting this triad are among the ‘weak’ synapomorphies and the resolution of the tree is rather low).

#### Iridoprinus myrmecophilus sp. n.

Taxon classificationAnimaliaColeopteraHisteridae

http://zoobank.org/8E437DF2-59F5-4044-86E7-51986937A7EB

Figs 202 , 203–211 , 212–218 , 757

##### Type locality.

Australia: Coniston Station near Alice Springs.

##### Type material examined.

Holotype, ♂, side-mounted on a triangular card with male genitalia glued to the same card as specimen with the following labels: “Coniston Station, / near Alice Springs / N.W. Mules” (printed), with hand-written text on the reverse side: “Nov 1. Dec.31 1931”; “Good/*Saprinus*” (hand-written); followed by: “SAMA Database/No.25-029415” (printed); followed by: “10-157” (yellow, pencil-written label added by the senior author); followed by: “Iridoprinus / myrmecophilus / n. gen. & sp. / HOLOTYPE / det. T. Lackner 2010” (red label, written) (SAMA). Paratype, ♀, labelled: “AUSTRALIA / Kudgee / 16.xii.[19]72 / B.P. Moore (printed-written), with written text on the reverse side: “In nest of *Iridomyrmex purpureus*”; followed by: “10-139” (yellow, pencil-written label added by the senior author); followed by: “Iridoprinus / myrmecophilus / n. gen. & sp. / PARATYPE / det. T. Lackner 2010” (red label, written) (ANIC).

##### Description.

Body length (only 1 specimen, the holotype was measured): PEL: 3.00 mm; APW: 0.85 mm; PPW: 2.35 mm; EL: 2.00 mm; EW: 2.65 mm.

Body (Fig. 202) rectangular oval, moderately convex from above, underside slightly flattened, cuticle light brown, pronotum rufous, elytra with blue iridescent metallic luster; legs and body appendages rufo-castaneous.

**Figures 203–211.**
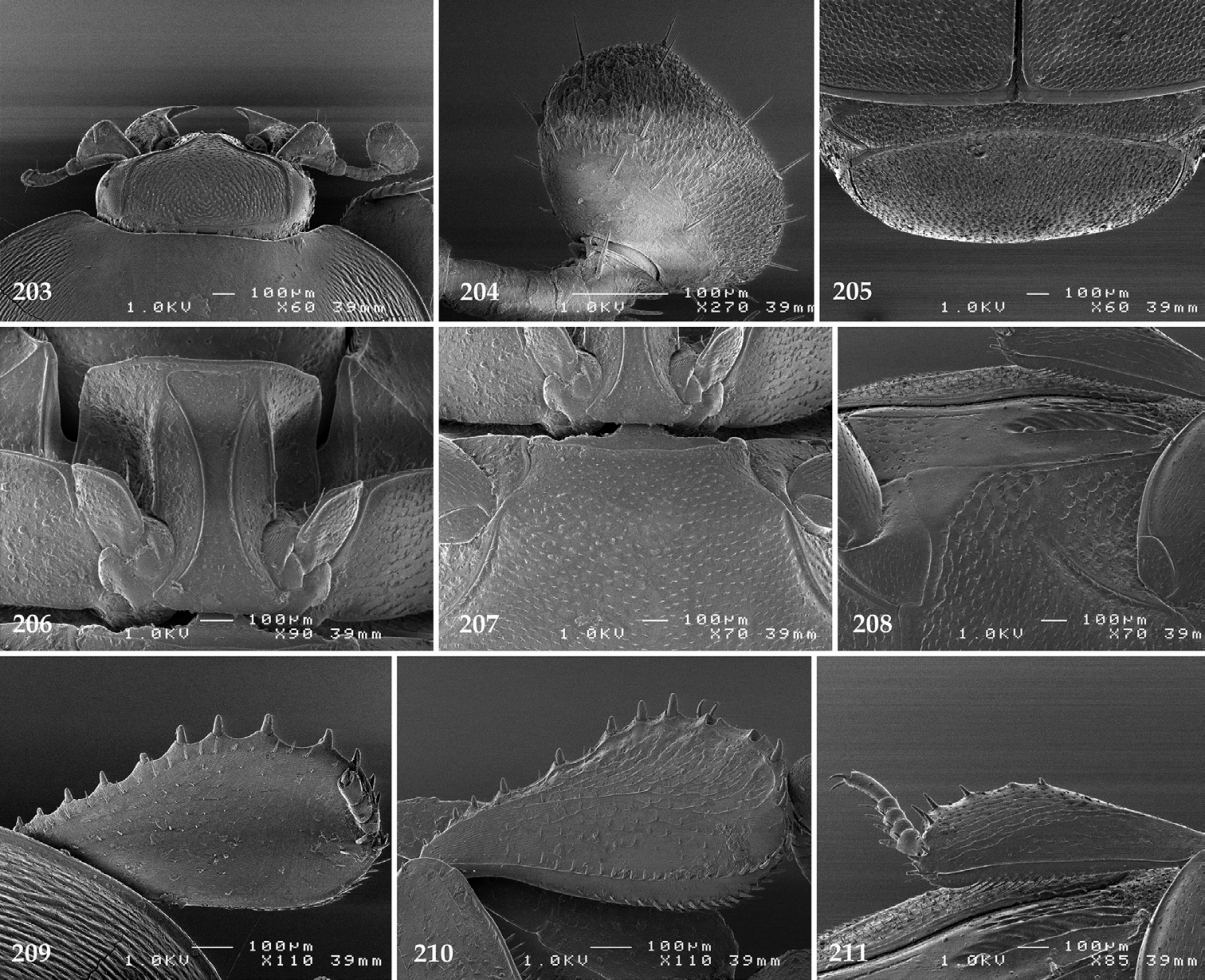
**203** *Iridoprinus myrmecophilus* sp. n. head, dorsal view **204** antennal club, dorsal view **205** propygidium + pygidium **206** prosternum **207** mesoventrite **208** lateral disc of metaventrite + metepisternum **209** protibia, dorsal view **210** ditto, ventral view **211** mesotibia, ventral view.

Antennal scape (Fig. 203) triangular, thickened, with several setae; club (Fig. 204) rather large, balloon-shaped, on apical two-thirds with short dense sensilla intermingled with sparse longer erect sensilla; sensory structures of antennal club not examined.

Mandibles with almost rectangular outer margin, acutely pointed, sub-apical tooth on inner margin of left mandible obtuse; mentum sub-trapezoid, anterior margin almost straight, without notch; other parts of mouth not examined.

Clypeus (Fig. 203) dorsally flattened, rounded laterally, with fine elongated punctures, separated by several times their diameter; supraorbital stria well developed, frontal stria carinate, prolonged onto clypeus; frontal disc (Fig. 203) densely punctuate, punctures deep, separated by about their diameter becoming elongate anteriorly, interspaces imbricate; eyes convex, well visible from above.

Pronotal sides (Fig. 202) feebly convergent anteriorly, apical angles obtuse, marginal pronotal stria present, laterally carinate, absent behind head; disc behind head with almost glabrous triangularly shaped ‘mirror’ (=polished area) with only scattered microscopic punctures, rest of pronotal disc coriarious-punctate, punctures becoming denser and coarser laterally, forming confluent elongate wrinkles; pronotal hypomeron glabrous; scutellum small, well visible.

Elytral epipleuron with microscopic punctures furnished with short setae; marginal epipleural and marginal elytral striae well impressed, complete; marginal elytral stria carinate, continuous along elytral apex as weakened apical elytral stria; humeral elytral stria inconspicuous (absent?); inner subhumeral stria absent; elytral disc with five curved strongly carinate dorsal elytral striae 1–5, striae sub-equal in length, fifth stria the shortest; sutural elytral stria absent. Entire elytral disc coarsely and very densely punctate, punctures rugulose, separated by less than half their diameter, each puncture with microscopic yellow seta well visible from lateral view.

Propygidium (Fig. 205) transverse, about four times as broad as long, partially covered by elytra, its punctation somewhat less dense and coarse than that of the elytra, punctures adorned with setae; pygidium (Fig. 205) with similarly dense and coarse setigerous punctures like those of the elytra, becoming sparser and finer towards apex.

Anterior margin of median portion of prosternum (Fig. 206) almost straight; marginal prosternal stria interrupted; prosternal process flattened, interspace between carinal prosternal striae excavated, smooth, laterally with scattered microscopic punctation, each puncture with minuscule seta; carinal prosternal striae (Fig. 206) divergent on both ends, united anteriorly; lateral prosternal striae carinate, almost straight, attaining apices of united carinal prosternal striae.

Anterior margin of mesoventrite (Fig. 207) almost straight; discal marginal mesoventral stria present only anteriorly, straight; disc flattened, with dense small punctures separated by about their own to twice their diameter, each puncture with microscopic seta, interspaces with alutaceous micro-sculpture; meso-metaventral suture distinct, straight, meso-metaventral sutural stria absent; intercoxal disc of metaventrite completely covered with punctation identical to that of mesoventrite, but interspaces without micro-sculpture. Lateral metaventral stria (Fig. 208) well impressed, shortened, carinate, curved outwardly; lateral disc of metaventrite (Fig. 208) excavated, with confluent shallow elongate punctures fringed with microscopic setae; metepisternum on apical half with deep longitudinal groove (Fig. 208) designed for accommodating mesotarsus, surface of metepisternum glabrous; fused metepimeron with scattered shallow punctures; lateral metepisternal stria present only on fused metepimeron, on metepisternum several large deep elongate punctures present.

Intercoxal disc of first abdominal ventrite completely striate laterally; surface of disc with punctation identical to that of mesoventrite, interspaces with alutaceous microsculpture.

Protibia (Fig. 209) flattened and dilated, outer margin almost without teeth, with 12–14 short thin denticles; setae of outer row short, sparsely paced; median row of setae absent; protarsal groove narrow but deep; anterior protibial stria absent; two thin, very short tarsal denticles present apically; protibial spur minute, growing out from apical protibial margin; outer and median part of posterior surface of protibia (Fig. 210) with several irregular rows of minute setae; demarcation line between outer and median part of posterior surface absent; posterior protibial stria complete, with regularly spaced minuscule setae turning into several thicker setae apically; inner margin with regular row of short setae becoming thicker apically.

Mesotibia (Fig. 211) dilated, outer margin with five widely spaced short denticles growing in size apically; setae of outer row sparse, regular, thin; setae of median row indistinguishable, entire posterior surface of protibia with several irregular rows of minuscule setae; posterior mesotibial stria inconspicuous (absent?); anterior surface of mesotibia with several irregular rows of minuscule setae; mesotibial spur stout, short; apical margin with two tiny denticles; claws of apical tarsomere about half its length; metatibia more slender than mesotibia, outer margin with only two short proximal denticles, in all other aspects similar to mesotibia.

**Figures 212–218.**
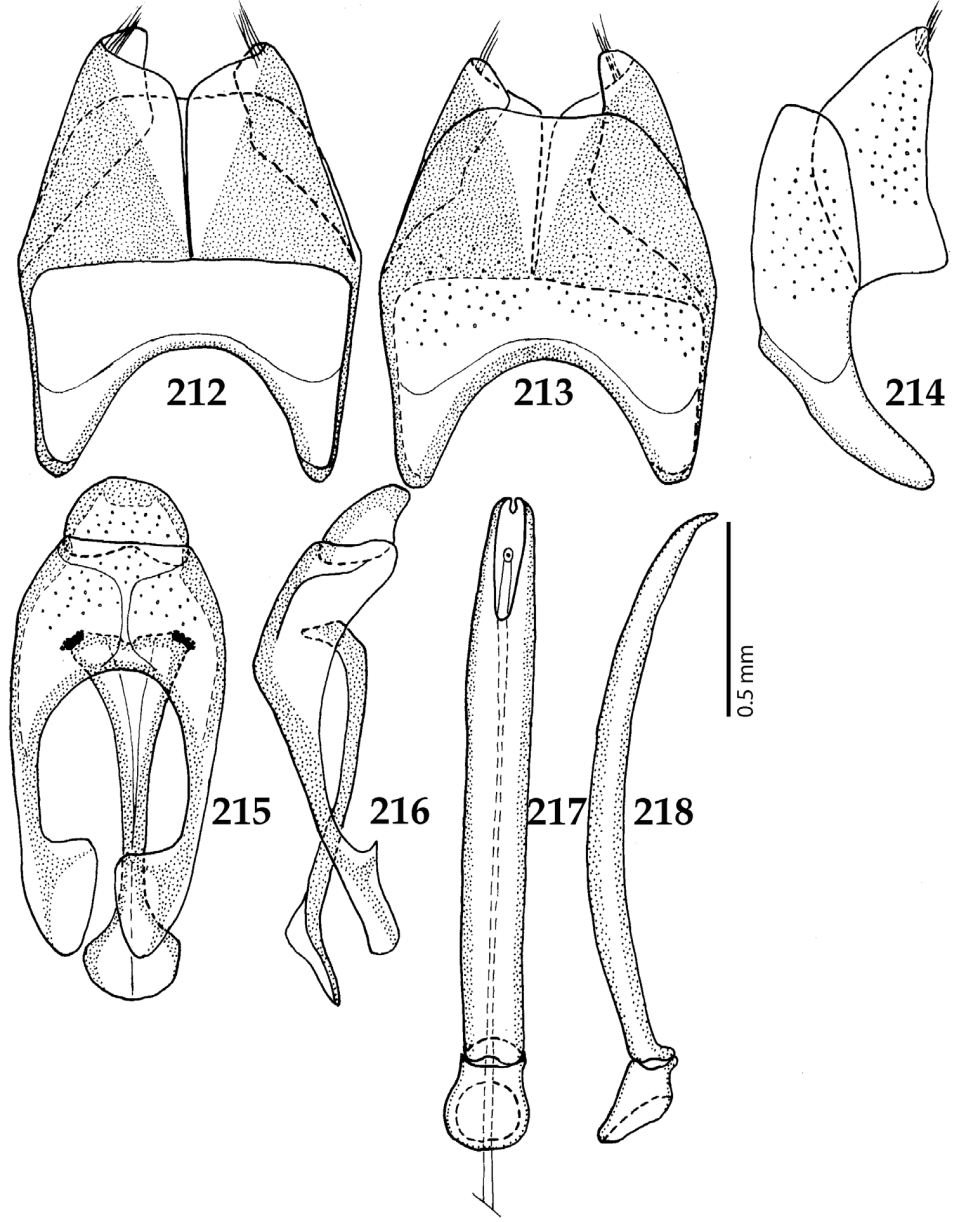
**212** *Iridoprinus myrmecophilus* sp. n. male terminalia: 8^th^ sternite + 8^th^ tergite, ventral view **213** ditto, dorsal view **214** ditto, lateral view **215** male terminalia: 9^th^ + 10^th^ tergites, dorsal view; spiculum gastrale, ventral view **216** male terminalia: 9^th^ + 10^th^ tergites; spiculum gastrale, lateral view **217** aedeagus, dorsal view **218** ditto, lateral view.

Male genitalia. Eighth sternite (Figs 212–213) apically longitudinally separated medially; vela absent, eighth sternite apically with several setae; eighth tergite and eighth sternite not fused laterally (Fig. 214). Ninth tergite (Fig. 215) longitudinally fused medially; spiculum gastrale (Fig. 215) gradually dilated in most of apical half, basal half moderately dilated, rounded. Aedeagus (Figs 217–218) conspicuously long compared to eighth sternite and tergite, slender; basal piece of aedeagus short, ratio of its length : length of parameres 1 : 6; parameres fused along their basal three-fourths; aedeagus slightly curved from lateral view (Fig. 218).

#### Notosaprinus

Taxon classificationAnimaliaColeopteraHisteridae

Kryzhanovskij, 1972

Figs 219 , 220–227 , 228–230 , 231–239 , 757

Notosaprinus Kryzhanovskij, 1972: 20. Type species Saprinus irinus Marseul, 1862, original designation.

##### Diagnosis.

Body comparatively large; pronotum with bronze luster, elytra with blue metallic luster (old specimens can be dark brown to black without luster); labrum in males with large median projection (Fig. 229); labral pits with a single labral seta; dorsal elytral striae reduced, usually only fourth dorsal elytral stria discernible; supraorbital stria absent; frontal stria prolonged onto clypeus; both sets of prosternal striae absent; metepisternum comparatively widened; sixth abdominal ventrite of male with conspicuous semicircular median elevation; pygidium laterally carinate; metatibia in male more strongly curved than in female; remaining characters as in *Saprinus*.

**Figure 219.**
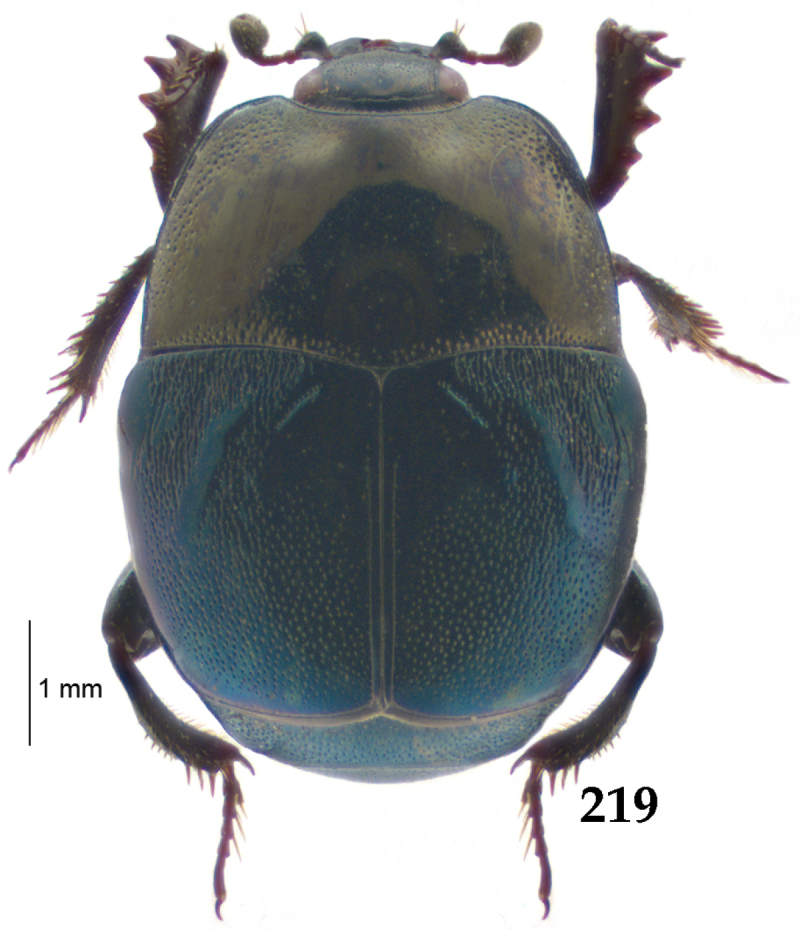
*Notosaprinus irinus* (Marseul, 1862) habitus, dorsal view.

##### Biology.

This is a typical saprobiont of the open landscapes and forest openings, mostly collected on carrion, where it presumably preys on fly larvae. Some specimens have been collected in pitfall and flight intercept traps.

##### Distribution.

*Notosaprinus* is a monotypic genus endemic to Australia: most records are from coastal areas of Queensland and New South Wales with a dubious record from Western Australia, see below (Fig. 757).

##### Remarks.

*Notosaprinus* is most similar to species of the genus *Saprinus*, differing from them chiefly by the absence of both sets of prosternal striae. The median elevation present on the sixth abdominal ventrite in males and sexually dimorphic labrum with single labral seta and absent supraorbital striae could be additional autapomorphies for this genus, however these features must be verified by extensive study of *Saprinus* species. In the phylogenetic study by Lackner (2014d), *Notosaprinus irinus* was recovered as sister taxon to Saprinus (Saprinus) semistriatus (Scriba, 1790), the type species of the species-rich genus *Saprinus*; albeit this relationship received only low support. The clade containing S. (S.) semistriatus and *N. irinus* was recovered sister to other higher taxa: type species of the genera *Xerosaprinus* Wenzel, 1962, *Hemisaprinus* Kryzhanovskij, 1976, *Styphrus* Motschulsky, 1845, *Paraphilothis* Vienna, 1994, and *Pilisaprinus* Kanaar, 1996. Apart from the absence of both sets of prosternal striae, *Notosaprinus* might be a member *Saprinus*, for it shares most of other synapomorphies with members of that genus. However, because there was only moderate statistical support for the phylogenetic placement, we maintain these taxa as separate genera.

#### Notosaprinus irinus

Taxon classificationAnimaliaColeopteraHisteridae

(Marseul, 1862)

Figs 219 , 220–227 , 228–230 , 231–239 , 757

Saprinus irinus Marseul, 1862: 443.

##### Type locality.

Australia.

##### Type material examined.

*Saprinus irinus* Marseul, 1862: Lectotype, present designation: ♀, (genitalia extracted and lost; specimen’s mounting card was pencil-marked with a female sign; probably not the original mounting card of Marseul), side-mounted, both protarsi, right mesotarsus, left metatibia and right metatarsus missing, with the following labels: “22a / Saprinus irinus m. / N. Holl. / T. Dré” (pink, round label, written); followed by one more identical label; followed by: “MUSEUM PARIS / irinus / COLL. / De MARSEUL 1890” (pink label, printed-written); followed by: “TYPE” (red-printed rectangular label); followed by: “Saprinus irinus / Marseul, 1862 / LECTOTYPE / des. T. Lackner 2014” (red label, written) (MNHN). This species has been described from an unknown number of specimens and the lectotype designation fixes the identity of the species.

##### Additional material examined.

AUSTRALIA. New South Wales: 3 ♀♀, Richmond River, 1909, collector unknown (BMNH); 2 ♂♂, Richmond River, without further data, Coll Van de Poll (MNHN); 1 ♀, Gerroa, 29.xii.1974, H. & A. Howden (MNHN); 1 ♀, Sydney, without further data (MNHN); 2 specs., N.S. Wales, without further data (MNHN); 1 spec., Chichester S.F., Allyn River, 32.08S 151.27E, T. Weir leg. (ANIC); 3 ♀♀ & 2 ♂♂, Lots, Caparra, 14.iii.1993, S. Watkins leg., under dead cat (ANIC); 1 spec., Mosgeil, without further data (MAMU); 1 spec, Route: Sydney to Mt. Abundance, thence to Maranoa and the Warrego; from there to Nivelle river, back to the Warrego (S., Centr. and W. Queensland, Hely’s Expedition 1852) (MAMU); 1 ♀ & 1 spec., Sydney, without further data, Griffith (SAMA). Queensland: 1 spec., Cairns, 7.ii.1927, no collector (BPBM); 1 spec., Queensland, Challenger exp., no further data (BMNH); 3 ♀♀, Mackay, Queensland, Turner, no further data (BMNH); 3 specs., Ewan Road, 8 miles W of Poluna, 18.i.1970, J.G. Brooks leg., fish lure (ANIC); 1 spec., Atherton, 26.iii.1965, Bornemisza leg. (ANIC); 1 spec., Thorton Range, Daintree, 12.–18.vii.1982, S. & J. Peck leg. (ANIC); 2 specs., Cape Tribulation, 40 km N of Daintree, 10 m, 18.vii.1982, S. & J. Peck leg. (ANIC); 2 specs., Kuranda St. For, 360 m, 27.–31.vii.1982, S. & J. Peck leg. (ANIC); 3 specs., Mount Mee, 7.5 km SW, 27°05'S, 152°42'E, 12.x.1991, Tom Gush leg., under dead snake on the road (ANIC); 4 ♂♂ & 7 ♀♀, Mt. Gavial, 3 km SSW, 18.xii.–14.iii.1999, 23°37'S, 150°28'E, D.J. Cook leg., vine forest 320 m, intercept trap (QM); 1 ♀, Tallebudgera Valley, 420 m, 4.–28.iv.1997, 153°18'E 28°14'S, D. Cook leg., carrion baited pitfall (QM); 2 specs., Brisbane valley, near Ravensbourne Nat. Park, 25.xi.1973, K. McDonald leg. (QM); 3 ♀♀, 2 ♂♂ and 3 specs., Baily Knob summit, 6.xii.1998–6.ii.1999, 17°39'S, 145°30'E, 1100 m, Monteith & Cook leg., intercept (QM); 3 specs., McAfee’s Lookout, 27°26'S, 152°53'E, 300 m, 18.x.1999, G.B. Monteith, under dead goanna (QM); 7 specs., Fraser Island, Yidney Scrub nr. VB52, 1.–2.xii.1975, G. Thompson & A. Slater (QM); 1 spec., Russel R., at Bellenden, Ker Landing, 5 m, 24.x.–9.xi.1981, Baited window trap (QM); 1 spec., Mt. Tamborine, 27.x.1957, S. Breden leg. (QM); 1 spec., Kirrama Range, Douglas Creek Road, 800 m, 10.xii.1986–11.i.1987, Monteith, Thompson & Hamlet leg., flight intercept trap (QM); 1 spec., Kroombit Tops, 65 km SW from Gladstone, 1000–1100 m, 22.–26.ii.1982, open forest, Monteith, Thompson & Yeates leg. (QM); 1 ♀, Mt. Blackwood, 590 m, 21°02'S, 148°57'E, 18.xi.1992–mid iv.1993, D. Cook & G.B. Monteith leg., intercept traps and pitfalls (QM); 1 spec., Maalan Road, 2 km S of Palmerston Highway, 17°36'S, 145°42'E, 7.iii.–15.v.1995, 750 m, Monteith & Hasenpusch leg., intercept trap (QM); 1 spec., Stone Creek, 17°28'S, 146°01'E, 100 m, 1.xi.1995–6.ii.1996, intercept trap, ground level (QM); 11 specs., Mt. Tambourine, xii. 1919, H. Pottinger leg. (QM); 1 spec., Gayndah, Masters (SAMA). Western Australia: 1 spec., K.G. Sound [probably King’s Sound; due to the uncertainty of exact locality not shown on the map] (MAMU).

Unknown localities: 2 ♀♀ & 1 spec., Australia, without further data (BMNH).

##### Biology.

A common species found on carrion and often collected by flight intercept traps.

##### Distribution.

Australia: Coastal regions of Queensland and New South Wales, the single record from Western Australia, is, due to the uncertain locality not shown on the map (Fig. 757).

##### Remarks.

This species is sexually dimorphic: males have a conspicuous median projection on the labrum, strongly curved metatibia, and sixth abdominal ventrite has a median elevation.

##### Re-description.

Body length: PEL: 4.25–6.20 mm; APW: 1.50–2.25 mm; PPW: 3.25–4.75 mm; EL: 2.75–3.85 mm; EW: 3.75–5.00 mm.

Body (Fig. 219) rectangular oval, convex; cuticle shining; pronotum dark brown with bronze metallic luster, elytra with blue metallic luster; legs, mouthparts and antennae castaneous brown; antennal club black.

**Figures 220–227.**
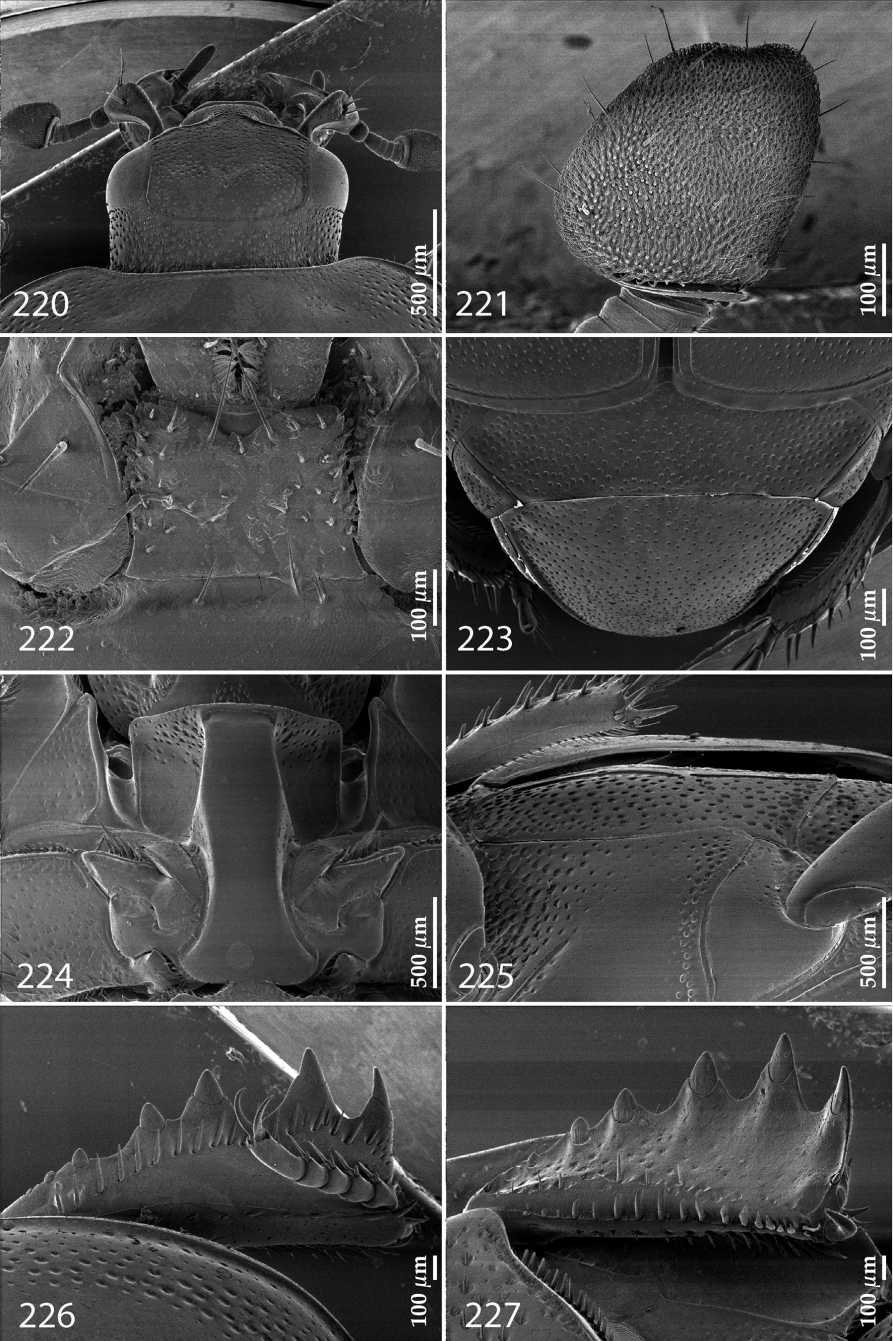
**220** *Notosaprinus irinus* (Marseul, 1862) head, dorsal view **221** antennal club, dorsal view **222** mentum, ventral view **223** propygidium + pygidium **224** prosternum **225** lateral disc of metaventrite + metepisternum **226** protibia, dorsal view **227** ditto, ventral view.

Antennal scape (Fig. 220) slightly thickened, imbricate-punctate, with few short setae; antennal club (Fig. 221) round, depressed dorso-ventrally, without visible articulation, entire surface with dense short sensilla intermingled with sparse longer erect sensilla; sensory structures of antennal club (Fig. 230) in form of four rather large sensory areas on ventral side and one vesicle situated under internal distal margin.

**Figures 228–230.**
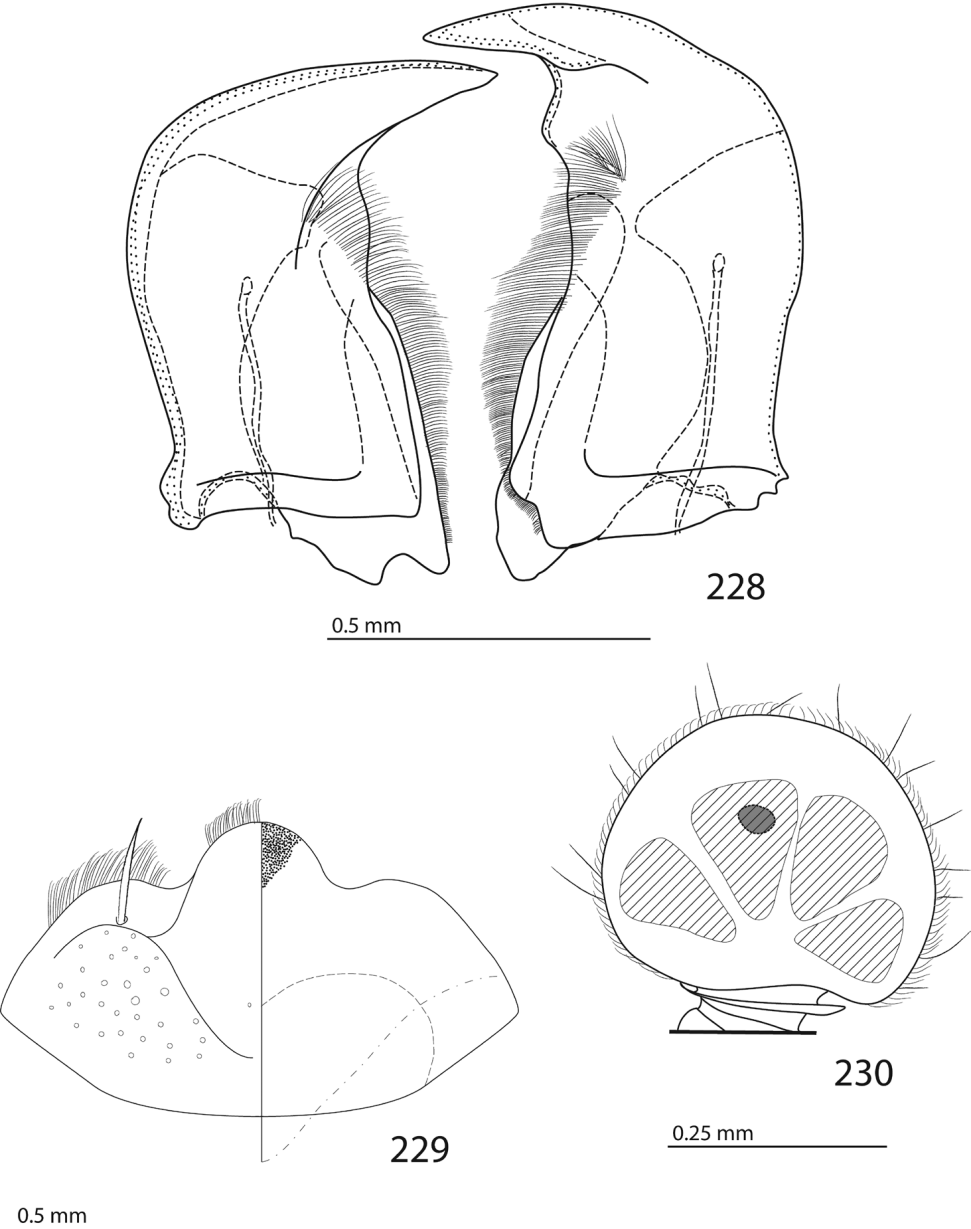
**228** *Notosaprinus irinus* (Marseul, 1862) mandibles, dorsal view **229** male labrum: left half depicting dorsal view and right half depicting epipharynx **230** antennal club, ventral view showing sensory structures of the antenna.

Mandibles (Fig. 228) punctate, with rounded outer margin, curved inwardly, mandibular apex acutely pointed; sub-apical tooth on inner margin of left mandible small, obtuse; labrum (Fig. 229) convex, densely punctate, depressed medially, in males with large median projection, in females without such projection, with two shallow labral pits, each with single short labral seta; labral fold inconspicuous; terminal labial palpomere elongated, its width about one-third its length; palpal organ minute, but present on both labial and maxillary palpi; mentum (Fig. 222) sub-trapezoid, anterior margin medially with deep notch (Fig. 222) surrounded with two long setae, lateral margins with several shorter setae; disc of mentum imbricate, with several sparse microscopic setae; cardo of maxilla on lateral margin with few short setae; stipes triangular, medially with single long seta, another long seta present laterally; lacinia without lacinial hook (=uncus); terminal maxillary palpomere elongated, its width about one-fourth its length, approximately twice as long as penultimate.

Clypeus (Fig. 220) punctate, narrowed anteriorly, depressed laterally; frontal stria widely interrupted medially (Fig. 220), prolonged onto clypeus, supraorbital stria absent; frontal disc (Fig. 220) with coarse and dense punctures; two vague depressions present on posterior half; eyes large, convex, well visible from above.

Pronotal sides (Fig. 219) moderately narrowing anteriorly, apical angles obtuse, pronotal depressions deep, rather large; marginal pronotal stria complete, carinate; disc of pronotum laterally with a band of coarse and dense punctation, not reaching posterior corners, between it and pronotal margin thin impunctate band present; pronotum medially almost smooth, with scattered microscopic punctation; along pronotal base two to three rows of coarse punctation present; pronotal hypomeron glabrous; scutellum small, well visible.

Elytral humeri slightly prominent, impunctate; elytral epipleura with scattered fine punctures; marginal epipleural stria fine, complete; marginal elytral stria nearly straight, in deep round punctures, continued as complete apical elytral stria. Humeral elytral stria vaguely impressed on basal third; outer subhumeral stria well impressed, thin; inner subhumeral stria erased by coarse elongate wrinkles; dorsal elytral striae 1–3 completely obliterated by coarse longitudinal wrinkles, fourth dorsal elytral stria deeply impressed, straight, shortened apically, present only on basal third of elytral length; sutural elytral stria shortened on basal sixth, well-impressed, apically connected with apical elytral stria. Elytral disc between elytral humerus and fourth dorsal elytral stria along elytral base with very coarse and dense elongate wrinkles reaching apically approximately one-third of elytral length, surface between this coarse band and fourth dorsal elytral stria creating a glabrous band, only with vague row of sparse punctures; surface between fourth dorsal elytral stria and elytral suture on basal third creating a glabrous triangular ‘mirror’ (= polished area); elytral surface otherwise with deep coarse and dense punctures becoming somewhat sparser and finer apically and towards elytral suture; elytral flanks glabrous.

Propygidium (Fig. 223) completely exposed, laterally with depressions, punctate, punctures separated by up to twice their diameter, interspaces finely imbricate; pygidium (Fig. 223) longer than broad along median line; laterally with moderately dense punctures becoming sparser medially, interspaces finely imbricate; carinate laterally.

Anterior margin of median portion of prosternum (Fig. 224) straight; marginal prosternal stria present only laterally; prosternal process flattened, glabrous, antero-laterally large coarse punctures present; both sets of prosternal striae absent.

Anterior margin of mesoventrite shallowly emarginate medially; discal marginal mesoventral stria well impressed, complete; disc of mesoventrite smooth; meso-metaventral sutural stria absent; meso-metaventral suture well impressed, thin; intercoxal disc of metaventrite glabrous, longitudinal suture of metaventrite deeply impressed, area along lateral metaventral stria and posterior margin with fine scattered punctation; along basal margin of metaventrite a band of coarser and denser punctures present; lateral metaventral stria shortened, straight; lateral disc of metaventrite (Fig. 225) slightly concave, with dense large setigerous punctures, interspaces finely substrigulate; metepisternum + fused metepimeron (Fig. 225) comparatively widened, with sparser and finer punctures, punctures not setigerous, interspaces substrigulate; on fused metepimeron punctation somewhat sparser, interspaces glabrous; lateral metepisternal stria complete, deeply impressed.

Intercoxal disc of the first abdominal ventrite almost completely striate laterally; disc with fine scattered punctation; sixth abdominal ventrite in males with distinctive median semicircular projection.

Protibia (Fig. 226) slightly dilated, outer margin with about three large distal teeth topped by stout triangular denticle, followed by four–five much smaller denticles diminishing in size in proximal direction; setae of outer row regular, short; protarsal groove deep; anterior tibial stria shortened apically; setae of median row much shorter and sparser than those of outer row; two apical denticles present near tarsal insertion; protibial spur well developed, bent, growing out from apical margin of protibia; apical margin of protibia ventrally with a single short denticle (occasionally there can be more than one denticle present); outer part of posterior surface (Fig. 227) of protibia smooth, only with several scattered shallow punctures, separated from glabrous median part by vague boundary and an undulate row of short sclerotized setae; posterior protibial stria complete, with moderately dense row of short, well-sclerotized setae; inner margin of protibia with double row of well-sclerotized lamellate setae growing in size and girth apically.

Mesotibia slender, outer margin with two rows of short denticles growing in size apically; setae of outer row regular, dense, about as long as denticles themselves; setae of median row shorter, regular; posterior mesotibial stria shortened apically; anterior surface of mesotibia almost smooth, with scattered shallow punctures; anterior tibial stria complete, terminating in two tiny inner ventral denticles; mesotibial spur rather long and thick; claws of apical tarsomere bent, longer than half its length, each mesotarsomere ventrally with two long, well sclerotized setae; metatibia in males more curved than in females, slenderer and longer than mesotibia, in all aspects similar to it, but denticles on outer margin much sparser.

**Figures 231–239.**
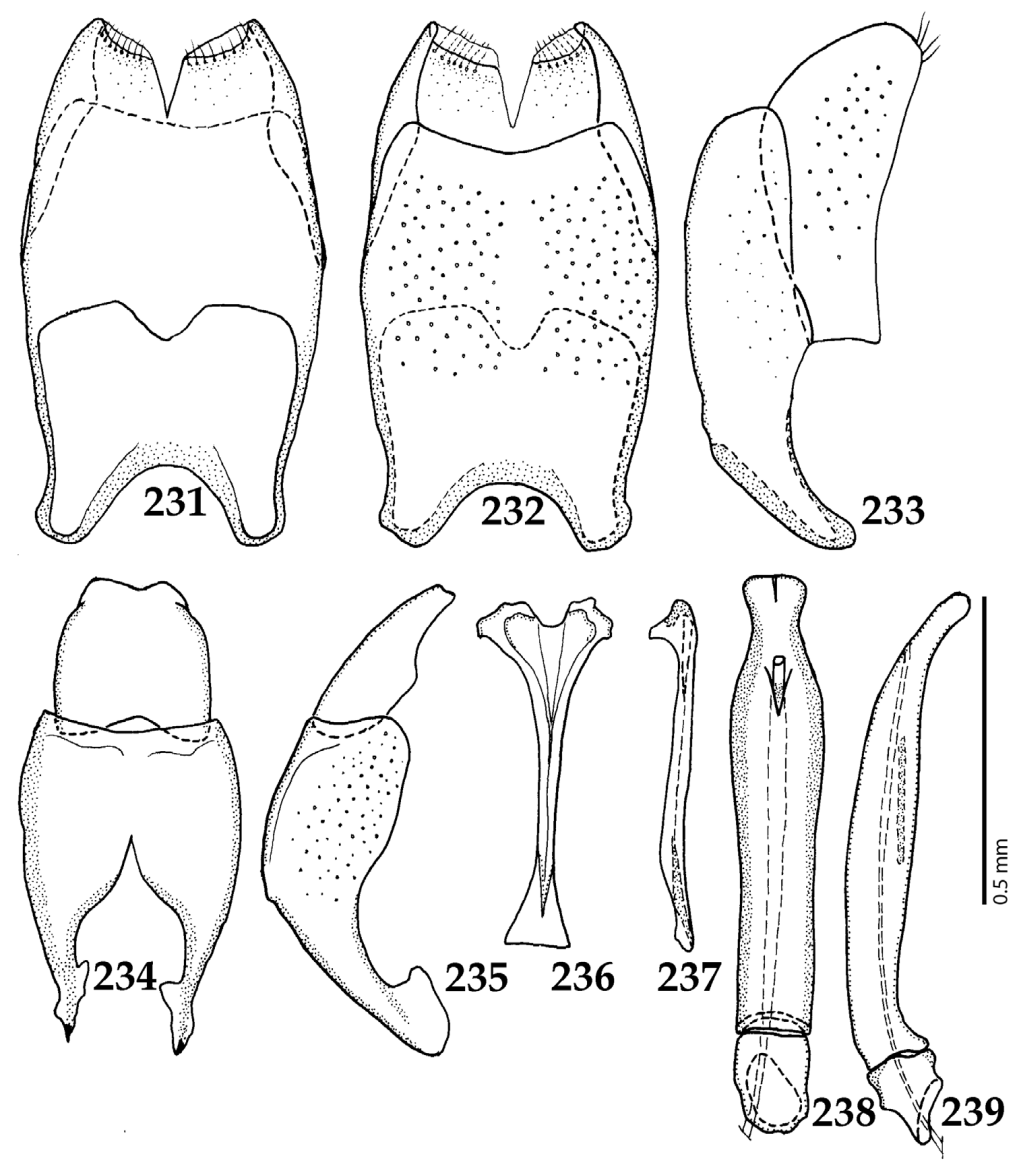
**231** *Notosaprinus irinus* (Marseul, 1862) male terminalia: 8^th^ sternite + 8^th^ tergite, ventral view **232** ditto, dorsal view **233** ditto, lateral view **234** male terminalia: 9^th^ + 10^th^ tergites, dorsal view **235** ditto, lateral view **236** male terminalia: spiculum gastrale, ventral view **237** ditto, lateral view **238** aedeagus, dorsal view **239** ditto, lateral view.

Male genitalia. Eighth sternite (Figs 231–232) fused longitudinally; vela with single row of sparse short setae; apex of eighth sternite laterally asetose; eighth tergite and eighth sternite not fused laterally (Fig. 233). Tenth tergite (Fig. 234) comparatively large, elongate; ninth tergite (Figs 234–235) longitudinally fused medially; spiculum gastrale (Figs 236–237) abruptly largely expanded on apical third, basally only slightly expanded, triangular. Aedeagus (Figs 238–239) thickened, constricted before apex; basal piece of aedeagus short, ratio of its length : length of parameres 1 : 4; parameres of aedeagus fused almost along their entire length with only a small apical circular opening for median duct; aedeagus only slightly curved from lateral view (Fig. 239).

#### Reichardtia

Taxon classificationAnimaliaColeopteraHisteridae

Wenzel, 1944

Figs 240 , 241–252 , 253–255 , 256–262 , 758

Reichardtia Wenzel, 1944: 91. Type species Saprinus pedator Sharp, 1876, original designation.

##### Diagnosis.

Cuticle light to dark brown, without metallic luster, entire dorsal surface glabrous; mandibles massive, strongly carinate dorsally; clypeus large, triangular and strongly convex; supraorbital and frontal striae absent; pronotal depressions absent; pronotal hypomeron with long yellow setae; prosternal foveae absent; prosternal apophysis strongly constricted between procoxae, prosternal process thence strongly expanded; both sets of prosternal striae absent, prosternal process setose; mesoventrite constricted between mesocoxae; all femora, meso- and metatibiae strongly swollen; protibia with a dense row of long thin denticles on outer margin; meso- and metatibiae with rows of setigerous punctures.

**Figure 240.**
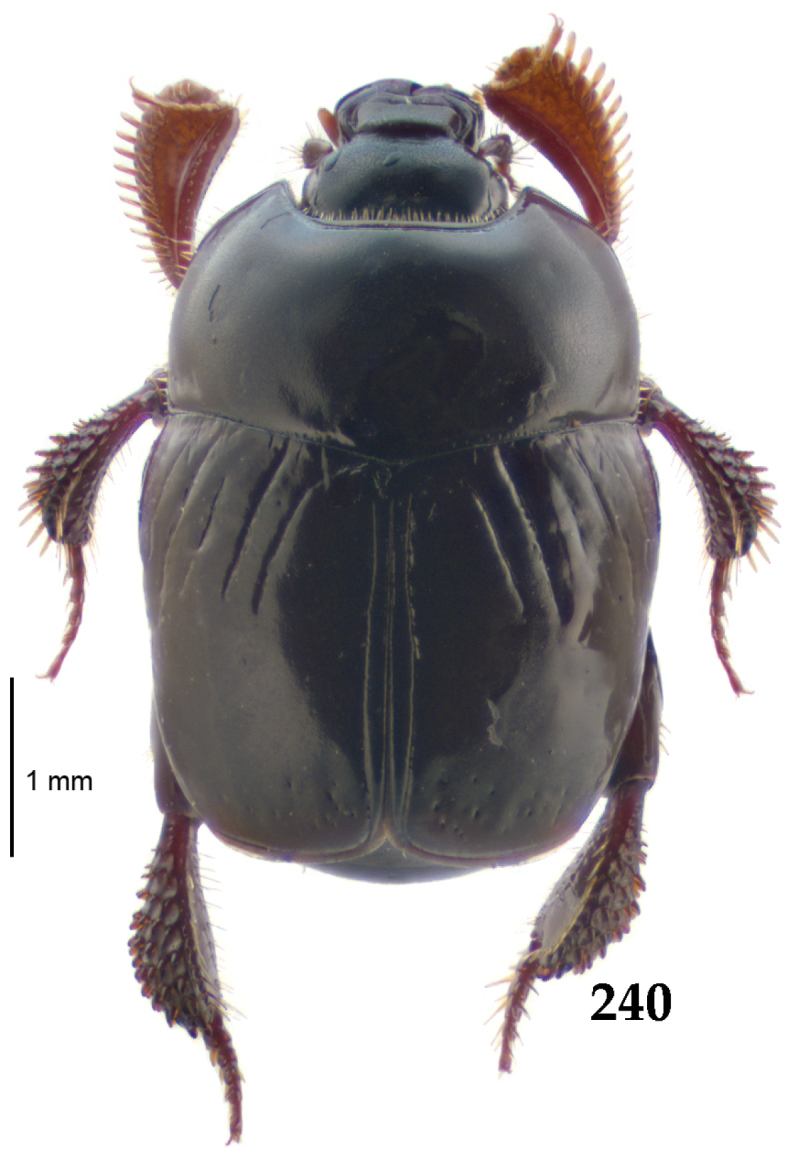
*Reichardtia pedator* (Sharp, 1876) habitus, dorsal view.

##### Biology.

This is a monotypic psammophilous taxon found on the beach under carrion or kelp at depths of 20 cm or more, occasionally also collected walking on sand surface.

##### Distribution.

*Reichardtia* is endemic to New Zealand and is found on both North and South Islands, but absent from off-shore islands (Fig. 758).

##### Remarks.

Based on the characters outlined above, especially the leg morphology, it is impossible to confuse *Reichardtia pedator* with any other taxon in the region. This New Zealand monotypic endemic is characterized by numerous autapomorphies, including almost impunctate dorsal surface in combination with the absence of supraorbital and frontal striae and a setose pronotal hypomeron. It is a rather derived member of the subfamily (Lackner 2014d), belonging to a global psammophilous clade. In the analysis of the senior author (loc. cit.) *Reichardtia* was recovered inside a purported monophyletic group *Australopachylopus lepidulus* (*Reichardtia pedator* + *Reichardtiolus pavlovskii*). However, as already noted by Leschen and Ôhara (2017) this relationship will probably not hold true as the characters uniting the triad may be a result of convergence, rather than synapomorphy.

#### Reichardtia pedator

Taxon classificationAnimaliaColeopteraHisteridae

(Sharp, 1876)

Figs 240 , 241–252 , 253–255 , 256–262 , 758

Saprinus pedator Sharp, 1876: 25.

##### Type locality.

New Zealand: Auckland.

##### Type material examined.

*Saprinus pedator* Sharp, 1876: Lectotype, present designation: 1 spec., with “Saprinus / pedator / N. Zeal. Type / D.S.” written on the actual mounting card with the specimen, followed by: “Auckland / New Zealand” (printed); followed by: “Sharp Coll. / 1905-313”; followed by: “Type” (red-margined printed round label); followed by: “Saprinus pedator / Sharp, 1876 / LECTOTYPE 2014 / Des. Lackner & Leschen” (red label, written) (BMNH). Paralectotypes, present designation: 1 spec., “Saprinus pedator / D.S. / Otago Hulton 1878” (written on the actual mounting card), followed by: “Sharp Coll. / 1905-313”; followed by: “Saprinus pedator / Sharp, 1876 / PARALECTOTYPE 2014 / Des. Lackner & Leschen” (red label, written) (BMNH). 1 spec., “Saprinus pedator / Sharp / Ind. Typ. / N. Zeald D.S.” (written on the actual mounting card); followed by: “Auckland” (red label, written); followed by: “Auckland / New Zealand” (printed); followed by: “Saprinus pedator / Sharp, 1876 / PARALECTOTYPE 2014 / Des. Lackner & Leschen” (red label, written) (BMNH). 1 spec., “Saprinus pedator / Sharp. / Otago Hulton 1878” (written on the actual mounting card); followed by: “Sharp Coll. / 1905-313”; followed by: “Saprinus pedator / Sharp, 1876 / PARALECTOTYPE 2014 / Des. Lackner & Leschen” (red label, written) (BMNH). 1 spec., with the following labels: “Saprinus / pedator / N. Zeald. Ind. Typ. / D.S.” (written); followed by: “G. Lewis Coll. / B.M. 1926-369” (printed); followed by: “Saprinus pedator / Sharp, 1876 / PARALECTOTYPE 2014 / Des. Lackner & Leschen” (red label, written) (BMNH). Sharp (1876) does not mention the exact number of specimens in his original description and the lectotype designation serves to fix the identity of the species.

**Figures 241–252.**
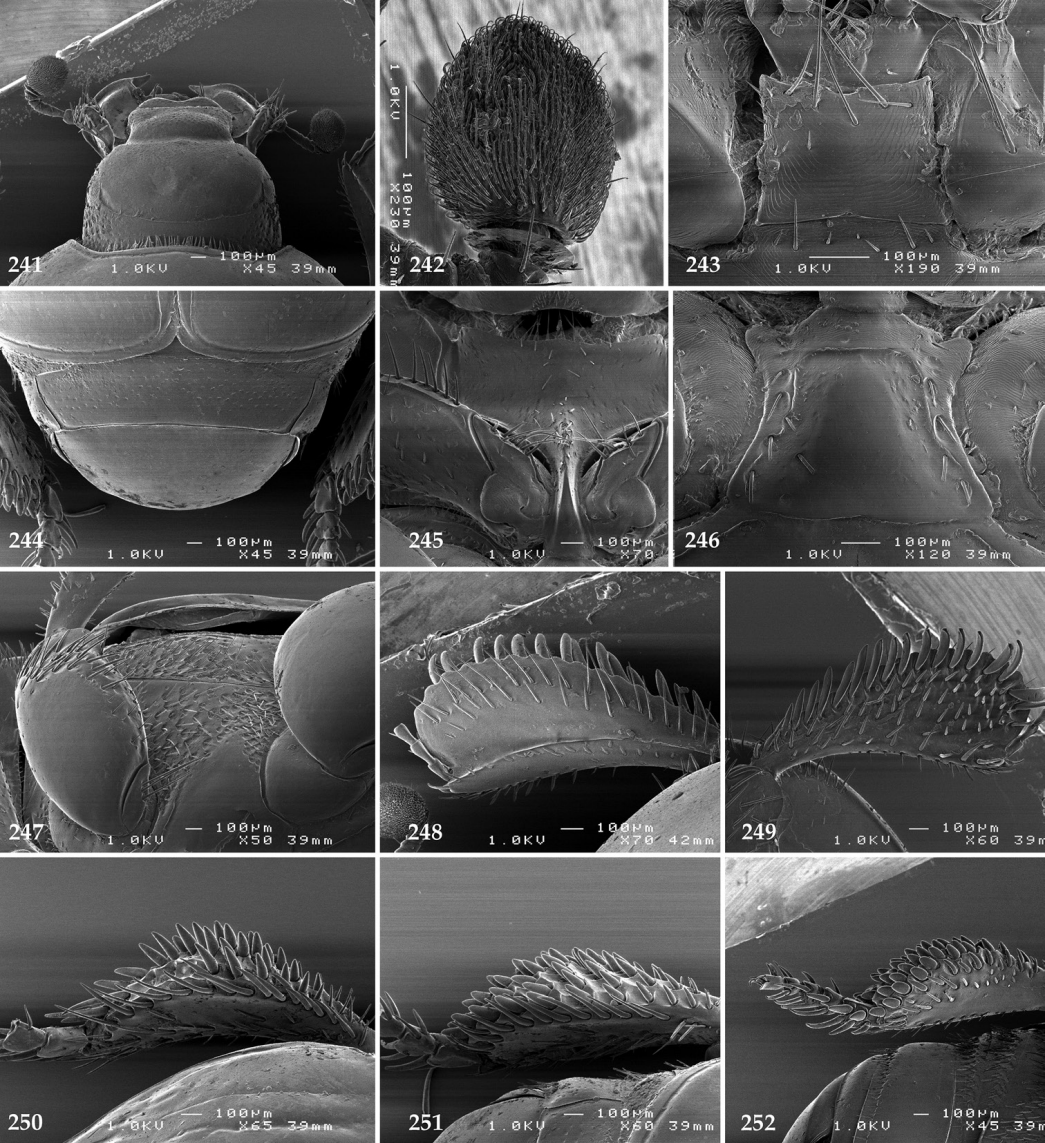
**241** *Reichardtia pedator* (Sharp, 1876) head, dorsal view **242** antennal club, ventral view **243** mentum, ventral view **244** propygidium + pygidium **245** prosternum **246** mesoventrite **247** lateral disc of metaventrite + metepisternum **248** protibia, dorsal view **249** ditto, ventral view **250** mesotibia, dorsal view **251** metatibia, dorsal view **252** ditto, ventral view.

##### Additional material examined.

NEW ZEALAND. North Island: ND: 1 spec., Waipapakauri, 7.xi.1991, G. Kuschel leg. (NZAC); 1 ♀, Cape Maria, 20.i.1963, L. Price leg. (NZAC). AK: 1 spec., Ruakaka Beach, N. Auckland, 22.ix.[19]32, C.E. Clarke coll. (BMNH); 1 spec., Muriwri Beach, 12.ix.[19]32, C.E. Clarke coll. (BMNH). TK: 1 spec., Taranaki, New Zealand, Broun Coll. (BMNH). WI: 1 spec., Himatangi Beach, 20.iii.1988, M.O.E. Peters leg. (AMNZ). WN: 1 spec., New Zealand, Parapararaumu, 17.ii.[19]24, O’Connor, G.V. Hudson, in dead fish on beach (BMNH); 1 spec., Wellington, ii.1902, J.J. Walker (BMNH); 1 spec., Manawatu Estuary, 25.iii.1984 (CJN); 1 spec., Waikawa Beach, 2.iv.1995 (CJN); 1 spec., Waikanae Beach, 9.ii.1995 (CJN). South Island. NN: 3 specs., Farewell Spit, Outer beach, 9.ii.1981, J.W. Early leg. (LUNZ); 7 specs., Wharariki Beach, 7.ii.1981, J.W. Early leg. (LUNZ, 3 exs. in coll. TLAN); 1 ♂, Tahunanui, Beach Nelson 21.viii.1976, J.C. Watt leg. (NZAC). SC: 1 spec., Temuka Beach, 22.iii.2008 (CJN). SL: 4 specs., South Island, Curio Bay, 50 km E of Invercargill, sandy beach, 4.xii.2000, Erber (ZMHUB).

DN: 2 specs., Kuri Bush, near Taieri Mouth, 3.xi.2001 (CJN); 2 specs., Sandfly Bay, Otago Peninsula, 18.i.2004 (CJN); 1 spec., Smails beach, Dunedin, 16.iii.1999 (CJN); 1 spec., Boulder Beach, Otago Peninsula, 5.i.2005 (CJN); ditto, but 29.xii.2001 (CJN); 1 spec., Waikouati, 14.i.2002 (CJN). Unknown localities: 1 spec., N. Zealand, no further data, G. Lewis Coll. (BMNH); 1 spec., New Zealand, no further data, Broun Coll. (BMNH); 1 spec; New Zealand, 25.ii.1917, Broun Coll. (BMNH).

##### Biology.

Littoral species found on beach under kelp, dead fish, often buried in the sand.

##### Distribution.

Endemic to New Zealand (Fig. 758).

##### Re-description.

Body length: PEL: 3.25–4.25 mm; APW: 1.25–1.60 mm; PPW: 2.35–3.00 mm; EL: 2.15–2.90 mm; EW: 2.60–3.35 mm.

Body (Fig. 240) rectangular oval, elytral humeri prominent, strongly convex, cuticle light to dark brown, shining, without metallic luster, entirely glabrous; legs, antennae and maxillary palpi similarly colored.

Antennal scape (Fig. 241) slightly thickened, with numerous long setae; antennal club (Fig. 242) comparatively small, rounded, entirely covered in dense short sensilla, intermingled with sparse longer erect sensilla; sensory structures of antennal club (Fig. 255) in form of a single ventral sensory area with pear-shaped vesicle situated beneath it.

**Figures 253–255.**
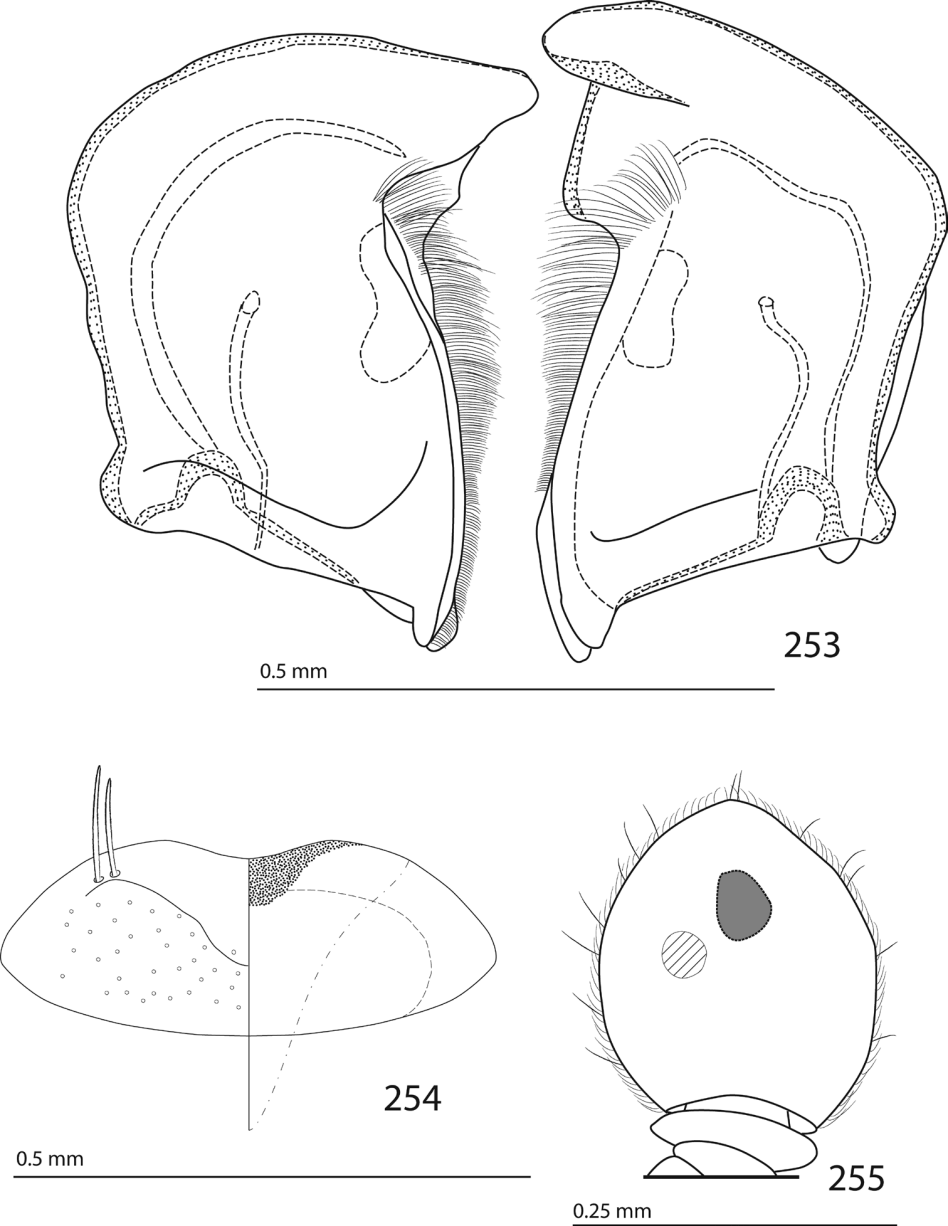
**253** *Reichardtia pedator* (Sharp, 1876) mandibles, dorsal view **254** labrum: left half depicting dorsal view and right half depicting epipharynx **255** antennal club, ventral view showing sensory structures of the antenna.

Mandibles (Fig. 253) massively thickened with rounded outer margin, strongly carinate dorsally, bluntly pointed, sub-apical tooth on inner margin of left mandible large, blunt; labrum (Fig. 254) faintly convex dorsally, sparsely microscopically punctate, anterior margin slightly emarginate; labral fold tiny, not conspicuous; setae of lateral fringe conspicuously short; labrum with two labral setae; terminal labial palpomere elongated, its width about one-third its length; palpal organ present on both labial and maxillary palpi; mentum (Fig. 243) sub-trapezoid, anterior angles slightly produced, anterior margin with shallow median excavation and tiny median notch (Fig. 243), surface around it with four long setae, lateral margins with several microscopic setae, surface of mentum imbricate; cardo of maxilla with few short setae on lateral margin; stipes triangular, with four long setae; lacinia without lacinial hook (=uncus); terminal maxillary palpomere elongated, its width about one-fourth its length, about three times as long as penultimate palpomere.

Clypeus (Fig. 241) large, rectangular, strongly convex, sloping down anteriorly, rounded laterally, glabrous, surface between clypeus and frontal disc strongly concave; supraorbital and frontal striae absent; frontal disc (Fig. 241) about twice as broad as long, with conspicuous median fovea; eyes slightly flattened, but visible from above.

Pronotal sides (Fig. 240) rounded, apical angles blunt, marginal pronotal stria complete, carinate, somewhat weakened behind head; disc strongly convex, glabrous; pronotal hypomeron with long yellow setae; scutellum small, but visible.

Elytral epipleura punctate; marginal epipleural stria thin, faintly impressed; marginal elytral stria well impressed, continuous along elytral apex as apical elytral stria, connected with sutural elytral stria; humeral elytral stria well impressed, occasionally continuous with inner subhumeral stria forming a stria parallel to first dorsal elytral; dorsal elytral striae deeply impressed, first the longest, reaching approximately half of elytral length apically, striae 2–4 shorter, reaching about one-third of elytral length apically; sutural elytral stria complete, deeply impressed, continuous with apical as well as 4^th^ dorsal elytral striae. Except for scattered punctures near elytral apex entire elytral disc glabrous.

Propygidium (Fig. 244) transverse, about three times as broad as long, completely exposed, sparsely punctate, punctures separated by several times their diameter, interspaces between punctures with fine alutaceous microsculpture; pygidium (Fig. 244) almost smooth, only on basal third with several scattered shallow almost inconspicuous punctures.

Anterior margin of median portion of prosternum (Fig. 245) bisinuate, setose, anterior angles somewhat produced; marginal prosternal stria absent; prosternal apophysis strongly constricted between procoxae, prosternal process knife-like, strongly widening and sloping down anteriorly, surface imbricate, with scattered punctures fringed with setae.

Anterior margin of mesoventrite (Fig. 246) straight; discal marginal mesoventral stria well impressed, fringed with several short setae; disc convex, only with microscopic punctation, strongly constricted between mesocoxae; meso-metaventral suture deeply impressed, meso-metaventral sutural stria well impressed, overriding meso-metaventral suture; intercoxal disc of metaventrite broad, smooth, in males with concave median longitudinal depression. Lateral metaventral stria well impressed, strongly curved outwardly; lateral disc of metaventrite (Fig. 247) convex, with round shallow large setigerous punctures; metepisternum + fused metepimeron (Fig. 247) with even denser setigerous punctures than those of lateral disc of metaventrite, punctures almost disappear on fused metepimeron; lateral metepisternal stria intermittent, present only on metepisternum.

Intercoxal disc of first abdominal ventrite in males with large depression medially, in females depression much smaller, lateral striae shortened apically; glabrous.

Protibia (Fig. 248) widening apically, outer margin with a row of about 17 densely-set straight long lamellate denticles growing in size apically; setae of outer row moderately dense, long and regular; setae of median row approximate to outer row, but shorter and sparser; protarsal groove deep; anterior protibial stria complete, carinate, next to it another row of much shorter sparse setae present, apically setae growing in length; protibial spur short, hooked, growing out near tarsal insertion; outer part of posterior surface of protibia (Fig. 249) with dense moderately long strongly sclerotized setae, demarcation line between outer, median and inner part of posterior surface of protibia absent; inner margin with double row of sparse sclerotized setae growing in size and becoming denser apically.

Mesotibia (Fig. 250) strongly thickened, outer margin with a dense row of thick denticles growing in size apically; setae of outer row strongly sclerotized, dense, growing in size but becoming thinner apically; setae of median row rather distanced from outer row, thinner and sparser; between the two rows several additional setae present; posterior mesotibial stria inconspicuous (absent?); anterior surface of mesotibia convex, with several dense rows of short denticles, becoming sparser and thinner towards inner mesotibial margin; anterior mesotibial stria inconspicuous (absent?); mesotibial spur inconspicuous; first and second tarsomeres ventrally with four long strongly sclerotized setae; third and fourth tarsomeres with only two such setae; fifth tarsomere devoid of setae ventrally; first two tarsomeres dorsally with two strongly sclerotized setae; third, fourth and fifth tarsomeres dorsally with only single seta; claws of apical tarsomere bent, shorter than half its length; metatibia (Figs 251–252) basically similar to mesotibia, but even more thickened, denticles of outer margin and anterior surface much denser and stouter than those of mesotibia.

**Figures 256–262.**
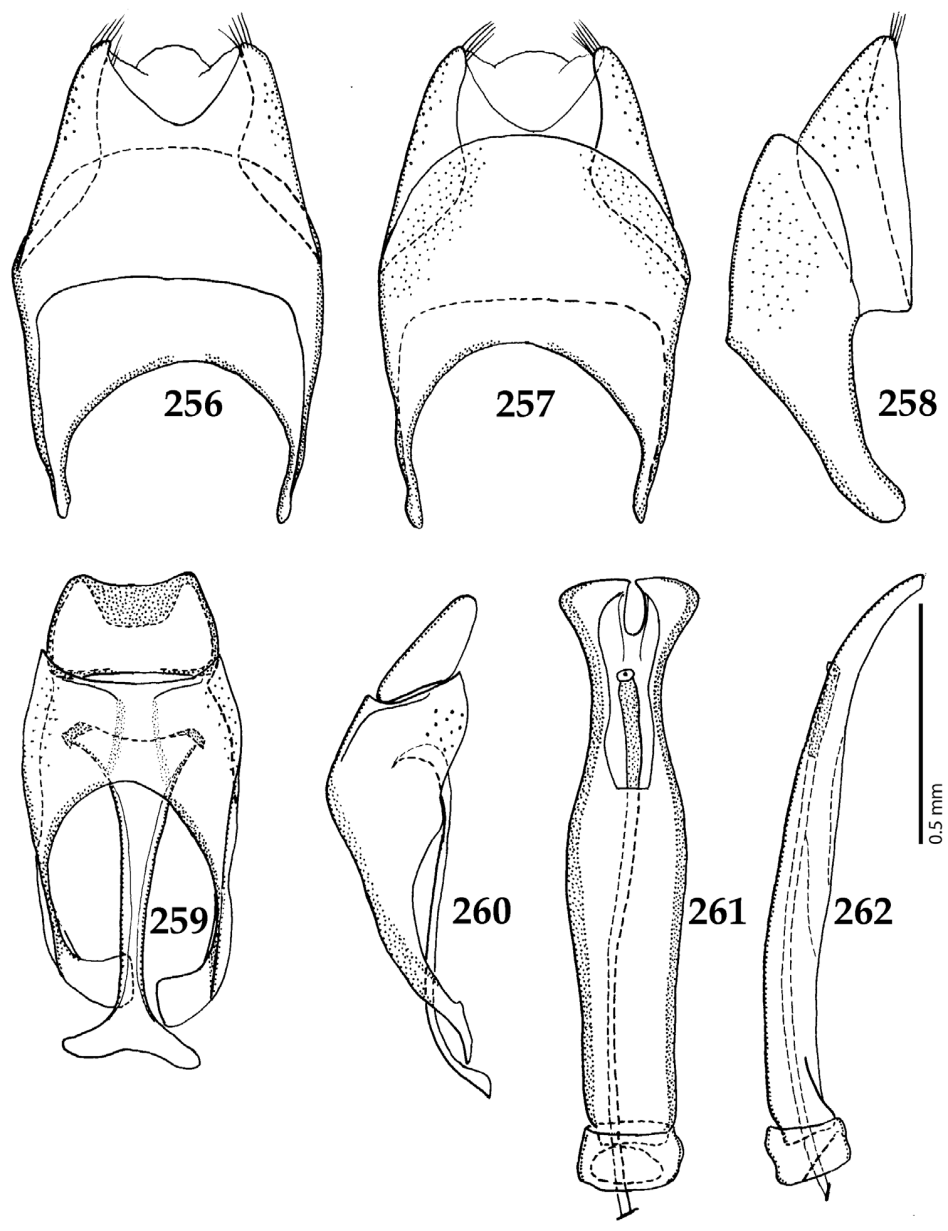
**256** *Reichardtia pedator* (Sharp, 1876) male terminalia: 8^th^ sternite + 8^th^ tergite, ventral view **257** ditto, dorsal view **258** ditto, lateral view **259** male terminalia: 9^th^ + 10^th^ tergites, dorsal view; spiculum gastrale, ventral view **260** male terminalia: 9^th^ + 10^th^ tergites; spiculum gastrale, lateral view **261** male terminalia: aedeagus, dorsal view **262** ditto, lateral view.

Male genitalia. Eighth sternite (Figs 256–257) fused longitudinally; vela absent; apex of eighth sternite laterally with several short setae; eighth tergite and eighth sternite not fused laterally (Fig. 258). Ninth tergite (Figs 259–260) longitudinally fused medially; spiculum gastrale (Fig. 259) gradually dilated on apical half, basal end strongly dilated, resembling flukes of a whale. Aedeagus (Figs 261–262) slightly thickened, constricted before apex; basal piece of aedeagus very short, ratio of its length : length of parameres 1: 7; parameres of aedeagus fused almost along their basal two-thirds; aedeagus only slightly curved from lateral view, apex of aedeagus flattened dorso-ventrally.

#### Saprinodes

Taxon classificationAnimaliaColeopteraHisteridae

Lewis, 1891

Figs 263 , 264–269 , 270–278 , 279 , 280–288 , 289–291 , 292–300 , 759

Saprinodes Lewis, 1891: 396. Type species Saprinodes falcifer Lewis, 1891, by monotypy.

##### Diagnosis.

Cuticle light to dark brown with faint bronze metallic tinge, entire dorsal surface (with the exception of vaguely delimited ‘mirrors’ on pronotal disc and elytra) rugulose-lacunose; labral pits and setae absent; pronotal depressions absent; prosternal foveae absent; bases of lateral prosternal striae with distinctive projection; protibia without teeth or denticles, very slender and elongate; protarsal groove very deep; protibial spur large; spiculum gastrale not expanded basally.

**Figure 263.**
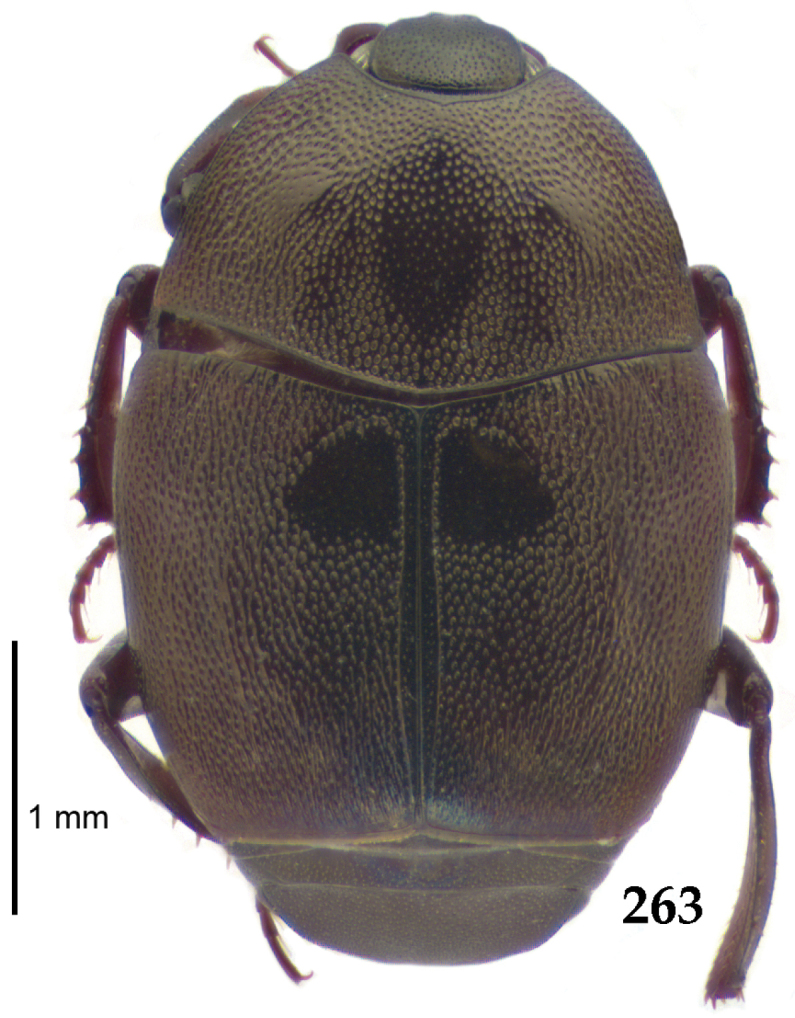
*Saprinodes distinctus* Dégallier, 1993, habitus, dorsal view.

##### Biology.

Unknown, most of the specimens have been collected using pitfall traps.

##### Distribution.

Endemic to Australia: Queensland and New South Wales (Fig. 759).

##### Remarks.

*Saprinodes* is the only Australopacific saprinine with the protibia devoid of teeth or denticles on its outer margin. Furthermore, its protibia is apically narrowed, terminating in a large protibial tooth. The peculiarly-shaped protibia of *Saprinodes* is not found in any other currently known Saprininae genus. The absence of labral pits and setae and the distinctive prosternal projection near the bases of lateral prosternal striae are also autapomorphies. In the morphology-based performed phylogenetic analysis published by the senior author (Lackner 2014d), the type species of the genus, *S. falcifer* Lewis, 1891 was recovered sister to *Iridoprinus myrmecophilus* with one ‘strong’ and two ‘weaker’ synapomorphies supporting their relationship (see also remarks section of *Iridoprinus*).

##### Key to the species of the genus *Saprinodes* Lewis, 1891

**Table:** 

1	Clypeus margined (Fig. 264), anterior margin of mentum weakly inwardly arcuate (Fig. 266), elytral striae distinguishable (Fig. 263); elytral ‘mirror’ (=polished area) larger (Fig. 263)	***Saprinodes distinctus* Dégallier, 1993** (Australia: Queensland)
–	Clypeus not margined (Fig. 280), anterior margin of mentum deeply inwardly arcuate (Fig. 282), elytral striae almost indistinguishable, erased under coarse punctures and strioles (Fig. 279); elytral ‘mirror’ (=polished area) smaller (Fig. 279)	***Saprinodes falcifer* Lewis, 1891** (Australia: New South Wales, Queensland)

#### Saprinodes distinctus

Taxon classificationAnimaliaColeopteraHisteridae

Dégallier, 1993

Figs 263 , 264–269 , 270–278 , 759

Saprinodes distinctus Dégallier, 1993: 49, figs 6–11.

##### Type locality.

Australia: Queensland: Danbulla S.F.

##### Type material examined.

*Saprinodes distinctus* Dégallier, 1993: holotype, ♂, genitalia glued to the same mounting card as the specimen, with the following labels: “AUSTRALIA: n. Qld. / Danbulla S.F., 13 km / NE of Yungaburra / 28.VII - 3.IX. 1987 / Storey & De Faveri” (printed-written); followed by: “MDPI Intercept / Trap Site No. 27” (printed-written); followed by: “Saprinodes / distinctus / HOLOTYPE / N. DEGALLIER / T. 13244” (red label, written) (QM). Allotype, ♀, ditto, but 20.XII.[19]86 - 13.I.[19]87, and Trap Site No. 21 (QM). Paratype, ♀, with genitalia glued to the same mounting card as the specimen, with the following labels: “The Crater, near / Herberton, N. Qld. / 16 dec. 1961 / McAlpine + Lossin” (written); followed by: “*Saprinodes* / *distinctus* / DEGALLIER / PARATYPE” (red label, written); followed by: “K 204388” (printed); followed by: “10-114” (yellow label, pencil-written, added by the senior author) (AMS).

##### Additional material examined.

AUSTRALIA. Queensland: 1 spec., Wongabel S.F., 5 km S Atherton, 800 m, 5.–14.xii.1988, Monteith & Thompson (FIT) (QM); 1 ♂ & 2 specs., Batavia Downs, 12.41S 142.41E, 22.vi.–23.viii.1992, P. Zborowski & J. Cardale (FIT) (ANIC).

##### Biology.

Unknown.

##### Distribution.

Endemic to Australia: Queensland (Fig. 759).

##### Remarks.

*S. distinctus* is rather similar to *S. falcifer*. Characters that best separate the two species are mentioned in the above key. The males of the two species differ only in the structure of eighth abdominal ventrite (compare Figs 270–271 with 292–293). Since we provide the type species of the genus, *S. falcifer*, with a full re-description we give for *S. distinctus* only a brief diagnostic description.

##### Diagnostic description.

**Figures 264–269.**
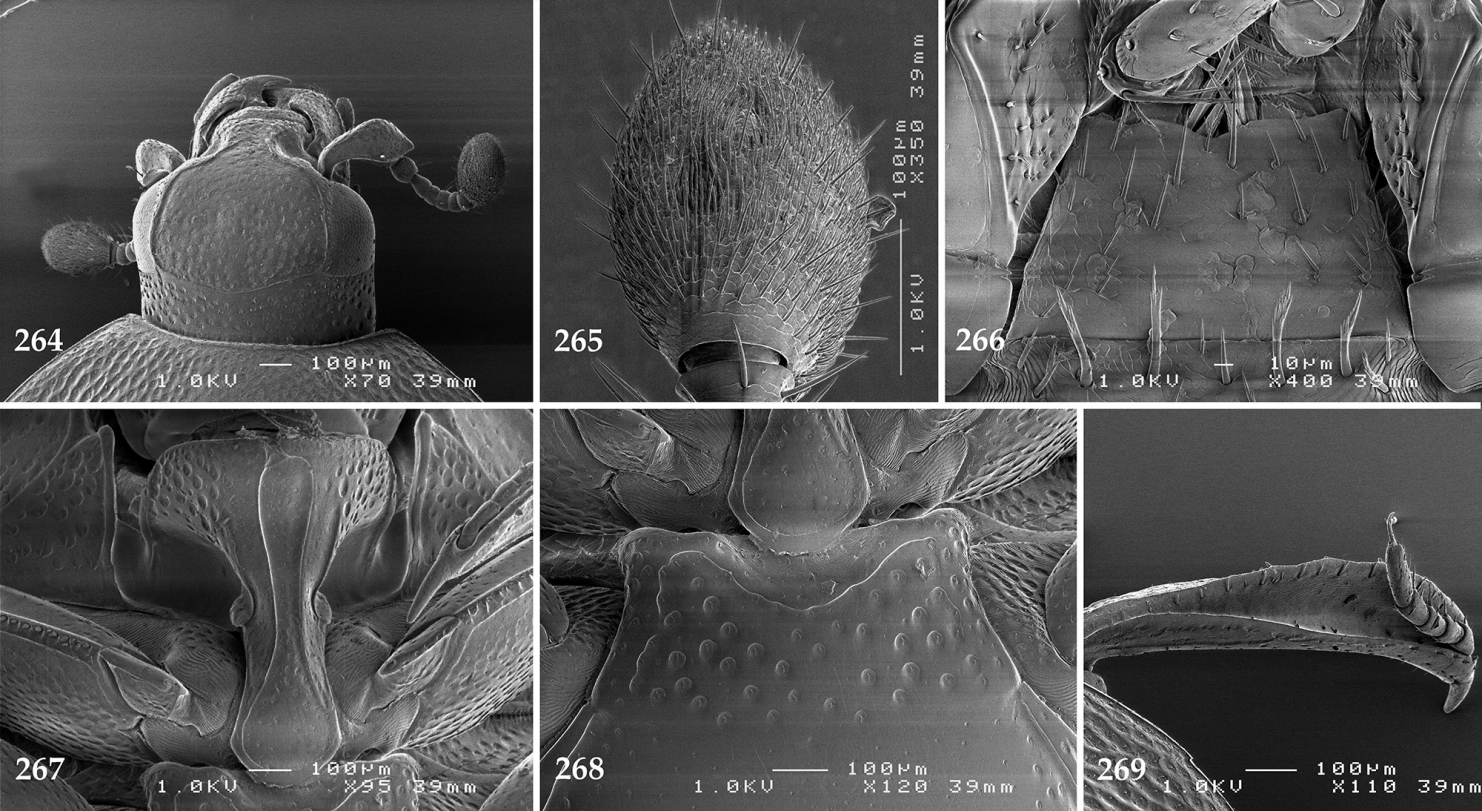
**264** *Saprinodes distinctus* Dégallier, 1993 head, dorsal view **265** antennal club, ventral view **266** mentum, ventral view **267** prosternum **268** mesoventrite **269** protibia, dorsal view.

Body length: PEL: 2.50–2.80 mm; APW: 0.75–0.85 mm; PPW: 1.75–2.00 mm; EL: 1.50–1.80 mm; EW: 2.00–2.25 mm. Body (Fig. 263) generally similar to *S. falcifer*, but elytral ‘mirror’ (=polished area) slightly larger, punctures slightly larger than in *S. falcifer* (compare Figs 263 and 279); legs, antennae and mouthparts similarly coloured between the two species. Antennal scape (Fig. 264) as well as antennal club (Fig. 265) almost identical to *S. falcifer*; sensory structures of antennal club not examined. Mandibles with rounded outer margin, acutely pointed, sub-apical tooth on inner margin of left mandible moderately large, rounded; labrum faintly convex dorsally, knobby, otherwise completely agrees with that of *S. falcifer*; mentum (Fig. 266) very similar to that of *S. falcifer*, but anterior margin slightly bisinuate and median emargination less deep than in following species (compare Figs 266 and 282); other mouthparts not examined.

Clypeus (Fig. 264) also similar between two species, but more constricted laterally (compare Figs 264 and 280), and more coarsely punctuate, margined laterally by prolonged divided frontal striae; supra-orbital striae well developed, carinate; frontal stria (Fig. 264) widely separated medially, continuous even further on clypeus than in *S. falcifer*; frontal disc (Fig. 264) more coarsely punctuate than that of *S. falcifer*, otherwise very similar to it; eyes convex, well visible from above.

**Figures 270–278.**
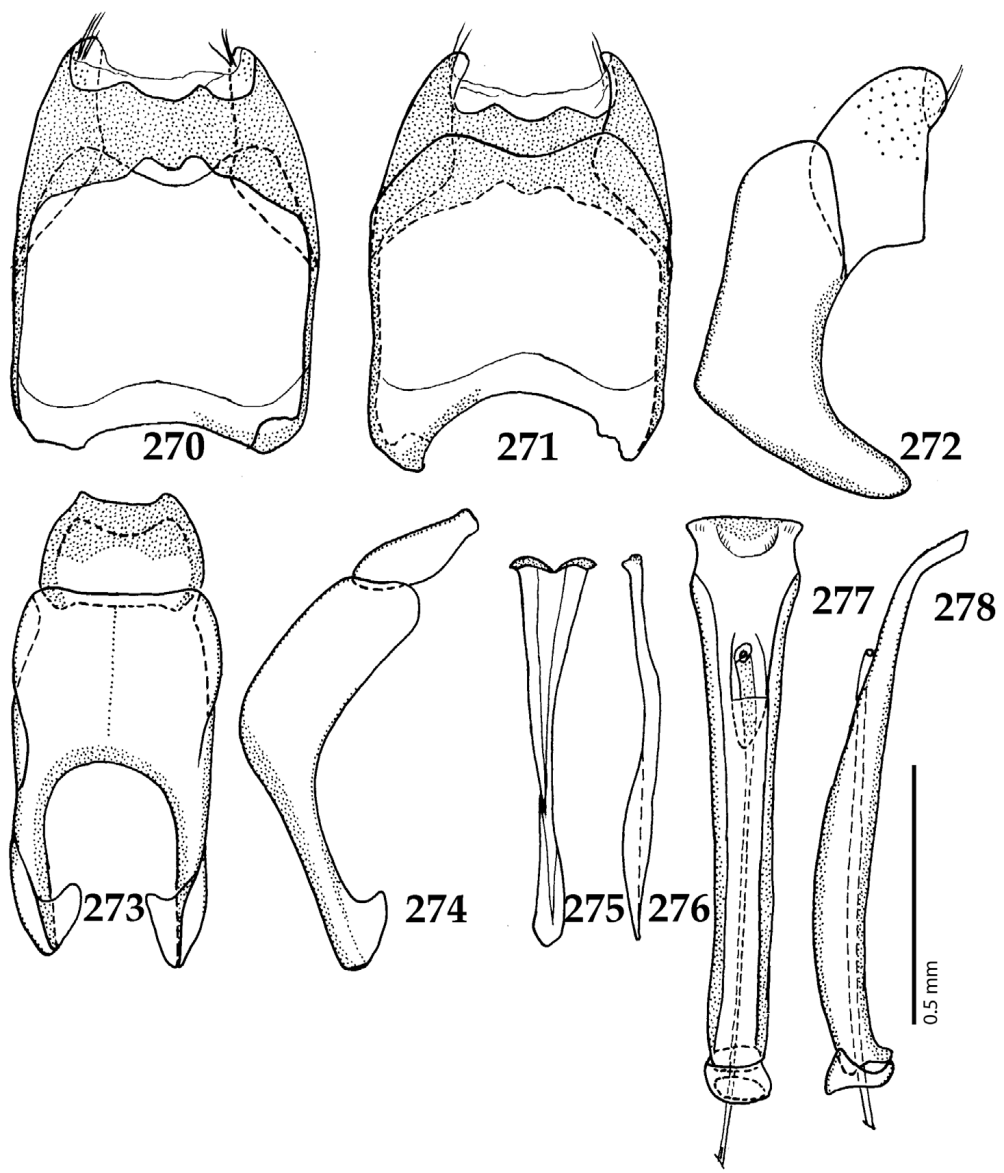
**270** *Saprinodes distinctus* Dégallier, 1993 male terminalia: 8^th^ sternite + 8^th^ tergite, ventral view **271** ditto, dorsal view **272** ditto, lateral view **273** male terminalia: 9^th^ + 10^th^ tergites, dorsal view **274** ditto, lateral view **275** male terminalia: spiculum gastrale, ventral view **276** ditto, lateral view **277** male terminalia: aedeagus, dorsal view **278** ditto, lateral view.

Pronotal sides (Fig. 263) strongly convergent apically, stronger so than in following species, rest of pronotum extremely similar to *S. falcifer* with exception of small angulate projection of frontal stria medially behind head which is absent in *S. distinctus* and present in *S. falcifer*; scutellum very small. Elytral striae striae 1–4 faintly recognizable. Entire elytral disc very coarsely and densely punctuate, punctation rugulose-lacunose, even coarser than that of *S. falcifer*, punctures on apical half confluent, aciculate and striolate (Fig. 263) on apical third punctures disappear, forming deep elongate wrinkles; basally between fourth dorsal elytral and sutural stria a small (however, slightly larger than that of *S. falcifer*) round ‘mirror’ (=polished area) present, its surface glabrous. Propygidium and pygidium very similar between the two species. Prosterna of the two species almost identical, curious projection near basal ends of lateral prosternal striae of *S. distinctus* (Fig. 267) even more prominent than in following species (Fig. 284). Mesoventrites (Fig. 268) as well as metaventrites of the two closely related species very similar; metepisterna also very similar. Intercoxal disc of first abdominal ventrite very similar in both species. Legs of both species (compare e.g. Figs 269 and 287) almost identical. Male genitalia (Figs 270–278) of *S. distinctus* differ basically only in the structure of eighth abdominal ventrite that is in *S. distinctus* fused medially (separated in *S. falcifer*) and vela that is in the case of *S. distinctus* almost asetose; apices of eighth sternite are with few setae in *S. distinctus* whereas they are without setae in *S. falcifer* (compare Figs 270–271 with Figs 292–293). Eighth abdominal tergite is medially deeply inwardly arcuate in *S. distinctus* whereas it is less so in *S. falcifer*.

#### Saprinodes falcifer

Taxon classificationAnimaliaColeopteraHisteridae

Lewis, 1891

Figs 279 , 280–288 , 289–291 , 292–300 , 759

Saprinodes falcifer Lewis, 1981: 396.

##### Type locality.

Australia: Queensland: Rockhampton.

**Figure 279.**
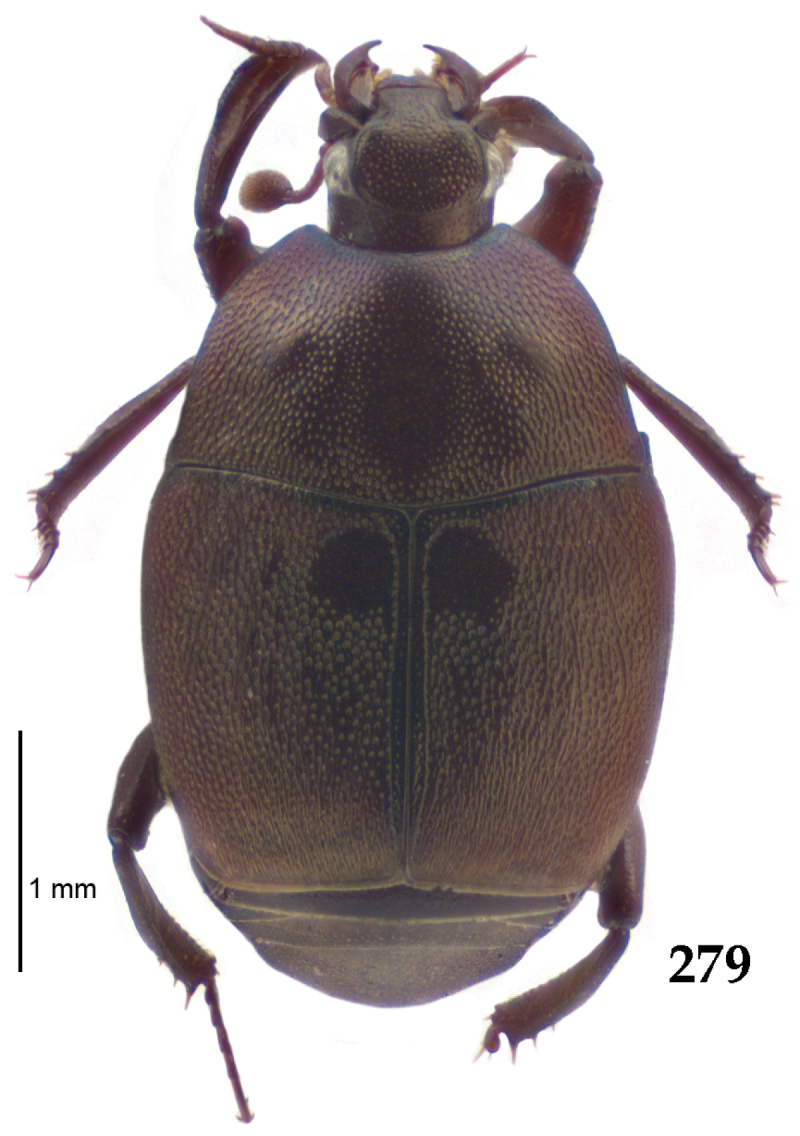
*Saprinodes falcifer* Lewis, 1891 habitus, dorsal view.

##### Type material examined.

*Saprinodes falcifer* Lewis, 1891: lectotype, designated by Dégallier in 1993, ♂, with genitalia glued to the same mounting card as the specimen, labelled: “Rockhampton/Queensland” (hand-written); followed by: “*Saprinodes*/*falcifer*/Type Lewis” (hand-written); followed by: “G. Lewis Coll./B.M. 1926-369.” (printed); followed by: “Type” (round label with red margins, printed); and a consecutive red label: “*Saprinodes*/*falcifer*/LECTOTYPE” (hand-written); followed by a yellow label: “10-120” (pencil-written, added by the senior author) (BMNH).

##### Additional material examined.

AUSTRALIA. Queensland: 11 specs., 25°27'S, 150°08'E, Taroom District, Nathan Gorge, Riverine Forest, 12.ix.–13.xi.1996, P. Lawless leg., pitfall (QM; 2 exs. in coll. TLAN). New South Wales: 2 ♂♂, Bogan River, F.H. Taylor leg. (ANIC); 7 ♂♂ & 3 ♀♀, ditto, but J. Armstrong (all exs. ANIC; 1 ♂ in coll. TLAN); 2 ♂♂ and 7 specs., ditto, but J. Armstrong leg. (MAMU).

##### Biology.

Unknown.

##### Distribution.

Endemic to Australia: New South Wales and Queensland (Fig. 759).

##### Remarks.

In this sexually dimorphic species the male has a large depression on the metaventrite and the first abdominal ventrite has coarser and larger punctures than that of the female. Females are also substantially larger than males. The minute setae associated with dorsal punctures observed by Dégallier (1993: 48) were not observed in our specimens.

##### Re-description.

Body length: PEL: 2.50–3.75 mm; APW: 0.85–1.05 mm; PPW: 1.80–2.55 mm; EL: 1.55–2.20 mm; EW: 2.10–3.00 mm.

Body (Fig. 279) ovoid, flattened dorso-ventrally, cuticle light brown to castaneous, with slight metallic tinge; ventral side darker than dorsal; legs and body appendages similarly colored.

**Figures 280–288.**
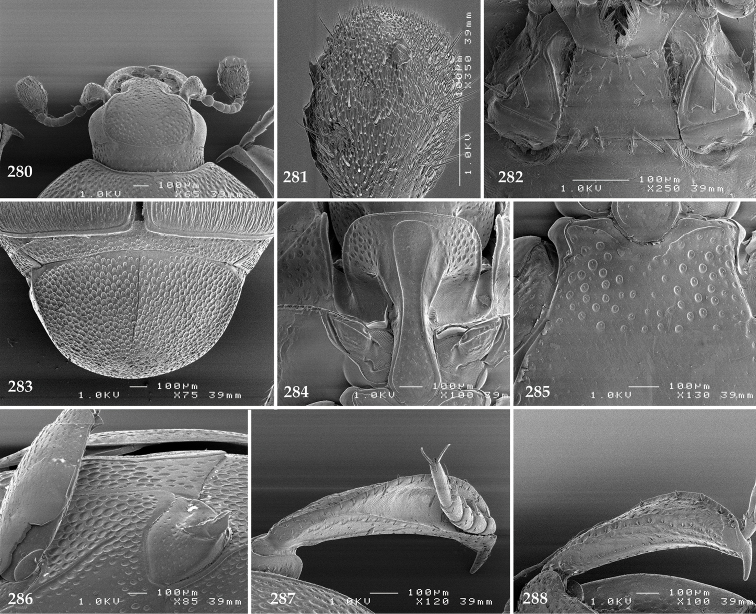
**280** *Saprinodes falcifer* Lewis, 1891 head, dorsal view **281** antennal club, ventral view **282** mentum, ventral view **283** pygidium **284** prosternum **285** mesoventrite **286** lateral disc of metaventrite + metepisternum **287** protibia, dorsal view **288** ditto, ventral view.

Antennal scape (Fig. 280) slightly thickened, punctuate dorsally, with few short setae; antennal club (Fig. 281) rather large, oval, dorsally without any visible structures, ventrally with two visible oval sensory areas, entirely covered in dense short sensilla, intermingled with sparse longer erect sensilla; sensory structures of antennal club (Fig. 291) in form of two ventral sensory areas, apical sensory area larger than basal, with pear-shaped vesicle situated beneath it.

**Figures 289–291.**
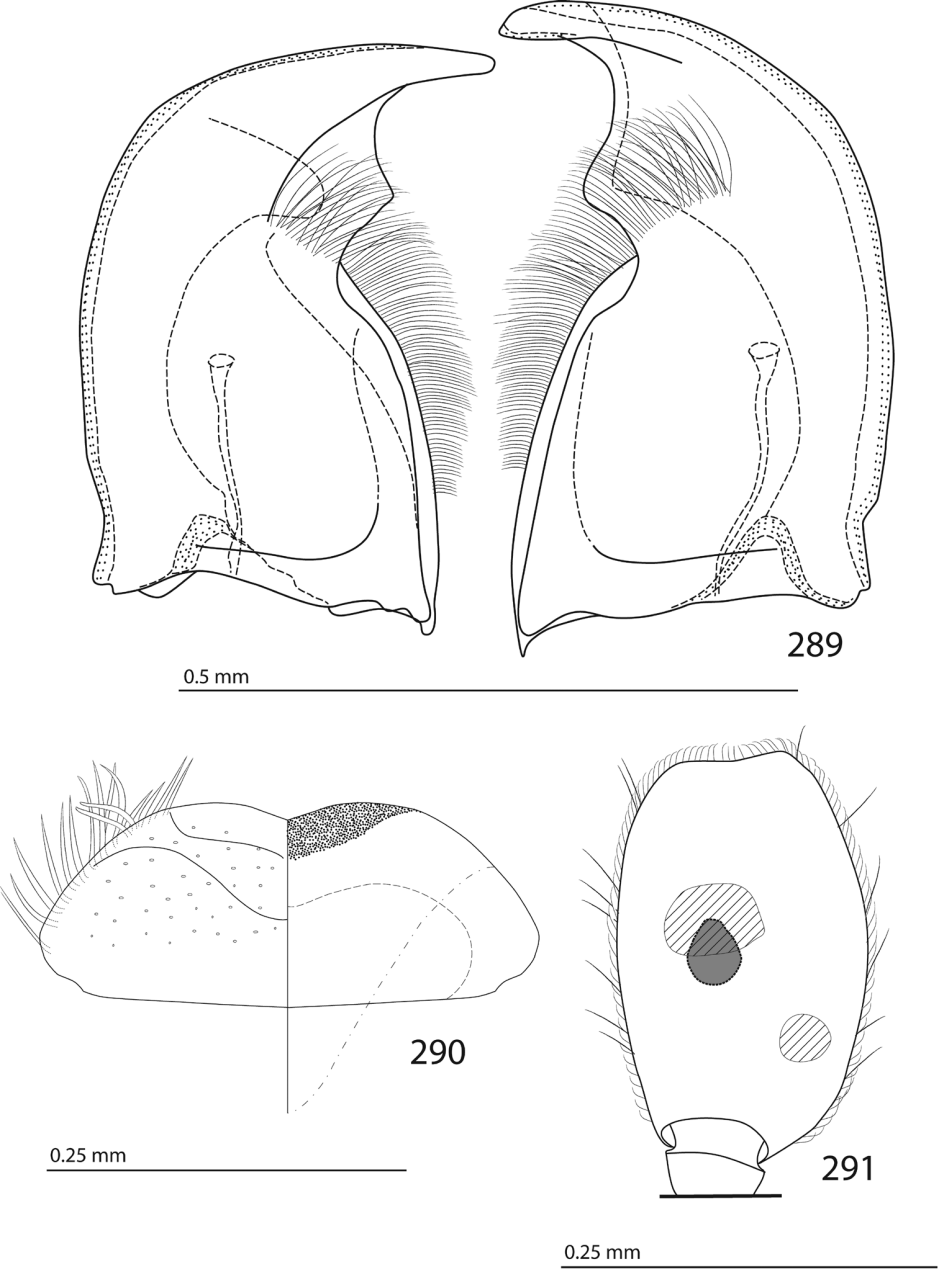
**289** *Saprinodes falcifer* Lewis, 1891 mandibles, dorsal view **290** labrum: left half depicting dorsal view and right half depicting epipharynx **291** antennal club, ventral view showing sensory structures of the antenna.

Mandibles (Fig. 289) with rounded outer margin, acutely pointed, sub-apical tooth on inner margin of left mandible moderately large, rounded; labrum (Fig. 290) faintly convex dorsally, sparsely punctuate, anterior margin slightly emarginate; labral fold not particularly developed; setae of lateral fringe moderately long; labrum without pits or setae; terminal labial palpomere elongated, its width about half its length; palpal organ present on both labial and maxillary palpi; mentum (Fig. 282) sub-trapezoid, anterior angles produced, anterior margin with deep median excavation, surface around it with several short setae, lateral margins with a single row of much shorter sparse ramose setae, disc on apical half with few scattered setae, basal half asetose; cardo of maxilla with few short setae on lateral margin; stipes triangular, with two short setae; lacinia without lacinial hook (=uncus); terminal maxillary palpomere elongated, its width about half its length, about twice as long as penultimate palpomere.

Clypeus (Fig. 280) large, rectangular, flattened, rounded laterally, with sparse fine punctures, separated by several times their diameter; supra-orbital striae well developed, carinate; frontal stria (Fig. 280) prolonged onto clypeus; frontal disc (Fig. 280) anteriorly somewhat depressed, punctuate, punctures separated by about their diameter, becoming coarser and denser near frontal stria, disappearing medially; eyes convex, well visible from above.

Pronotal sides (Fig. 279) on posterior half feebly convergent anteriorly, on anterior half strongly convergent, apical angles produced, marginal pronotal stria complete, carinate, behind head medially with tiny angulate projection; disc antero-laterally with deep, rugulose-lacunose oval punctation, medially punctures becoming much sparser and finer, separated by several times their diameter, forming vague impunctate areas (in some specimens these can be better delimited forming ‘mirrors’), interspaces between punctures on entire pronotal disc with sparse microscopic punctation; pronotal hypomeron glabrous; scutellum very small.

Elytral epipleura punctuate; marginal epipleural stria thin; marginal elytral stria well impressed, thin, continuous along elytral apex as apical elytral stria, connected with sutural elytral stria; humeral elytral stria erased under elytral punctation; inner subhumeral stria present medially, almost unrecognizable beneath elytral punctation; dorsal elytral striae almost unrecognizable from dorsal view, from oblique view striae 1–4 faintly recognizable, first the longest, originating near elytral base running to 3/4 of elytral length apically, striae 2–4 abbreviated basally and apically; sutural elytral stria complete, deeply impressed, surface between it and elytral margin with scattered microscopic punctation. Entire elytral disc very coarsely and densely punctate, punctation rugulose-lacunose, punctures separated by less than half of their diameter, on apical third punctures disappear, forming deep elongate wrinkles; basally between fourth dorsal and sutural striae a small round ‘mirror’ (= polished area) present, its surface with scattered microscopic punctation.

Propygidium transverse, about five times as broad as long, partially covered by elytra, its punctation much finer and sparser than those of the elytra, punctures on apical half separated by about their diameter, on basal half propygidium almost impunctate, interspaces between punctures with fine alutaceous microsculpture; punctation of pygidium (Fig. 283) much coarser and denser, resembling that of elytra; pygidium carinate laterally.

Anterior margin of median portion of prosternum (Fig. 284) straight; marginal prosternal stria almost complete, interrupted laterally; prosternal process deeply concave on anterior half, laterally with sparse large oval punctures, interspaces imbricate; dorsally prosternal process with sparse shallow punctures; carinal prosternal striae (Fig. 284) carinate, slightly divergent anteriorly and united in front under a rounded loop; lateral prosternal striae carinate, widely convergent and ‘open’ anteriorly, near their origin a curious tiny projection present, unseen in any other members of the subfamily (Fig. 284). Lateral costa of antennal groove not reaching prosternal process.

Anterior margin of mesoventrite (Fig. 285) deeply emarginate medially; discal marginal mesoventral stria anteriorly well impressed, absent laterally; disc with antero-lateral depressions, with sparse large round punctures separated by several times their diameter, each puncture with a microscopic seta, interspaces between punctures with scattered microscopic punctation; meso-metaventral suture indistinct, meso-metaventral sutural stria absent; intercoxal disc of metaventrite in males medially with large depression; metaventrite of females with only slight median longitudinal depression. Disc of metaventrite in both sexes almost smooth, punctation appears only along lateral and posterior margins. Lateral metaventral stria (Fig. 286) well impressed, almost straight, not reaching metacoxa; lateral disc of metaventrite (Fig. 286) depressed, with round shallow large punctures; metepisternum evenly punctate with similar punctation, punctures on fused metepimeron much sparser than those of metepisternum; lateral metepisternal stria (Fig. 286) present, deeply impressed, complete.

Intercoxal disc of first abdominal ventrite in males with large depression, in females not depressed, completely striate laterally; surface of disc in males with scattered oblong punctation, punctures becoming sparser and finer medially, in females punctation much sparser and finer.

Protibia (Fig. 287) slender, only very slightly dilated apically, terminating in massive protibial spur; setae of outer row very short, sparsely spaced; setae of median row even shorter than those of outer row, even; protarsal groove deep, designed to accommodate entire protarsus in repose; anterior protibial stria complete, costate; outer part of posterior surface of protibia (Fig. 288) with a row of sparsely spaced minuscule denticles; median part of posterior surface with a single row of regular minuscule setae; posterior protibial stria complete, very thin, with scattered minuscule setae; inner margin with single row of sparse microscopic setae.

Mesotibia slender, outer margin with a single row of around five denticles growing in size apically; setae of outer row sparse, minuscule; setae of median row similar to those of outer row, between the two rows an additional complete stria present; posterior mesotibial stria shortened apically; anterior surface of mesotibia convex, with dense row of well-sclerotized short setae; anterior mesotibial stria complete; mesotibial spur inconspicuous; claws of apical tarsomere about half its length; metatibia basically similar to mesotibia, but denticles of outer margin much sparser than those of mesotibia.

**Figures 292–300.**
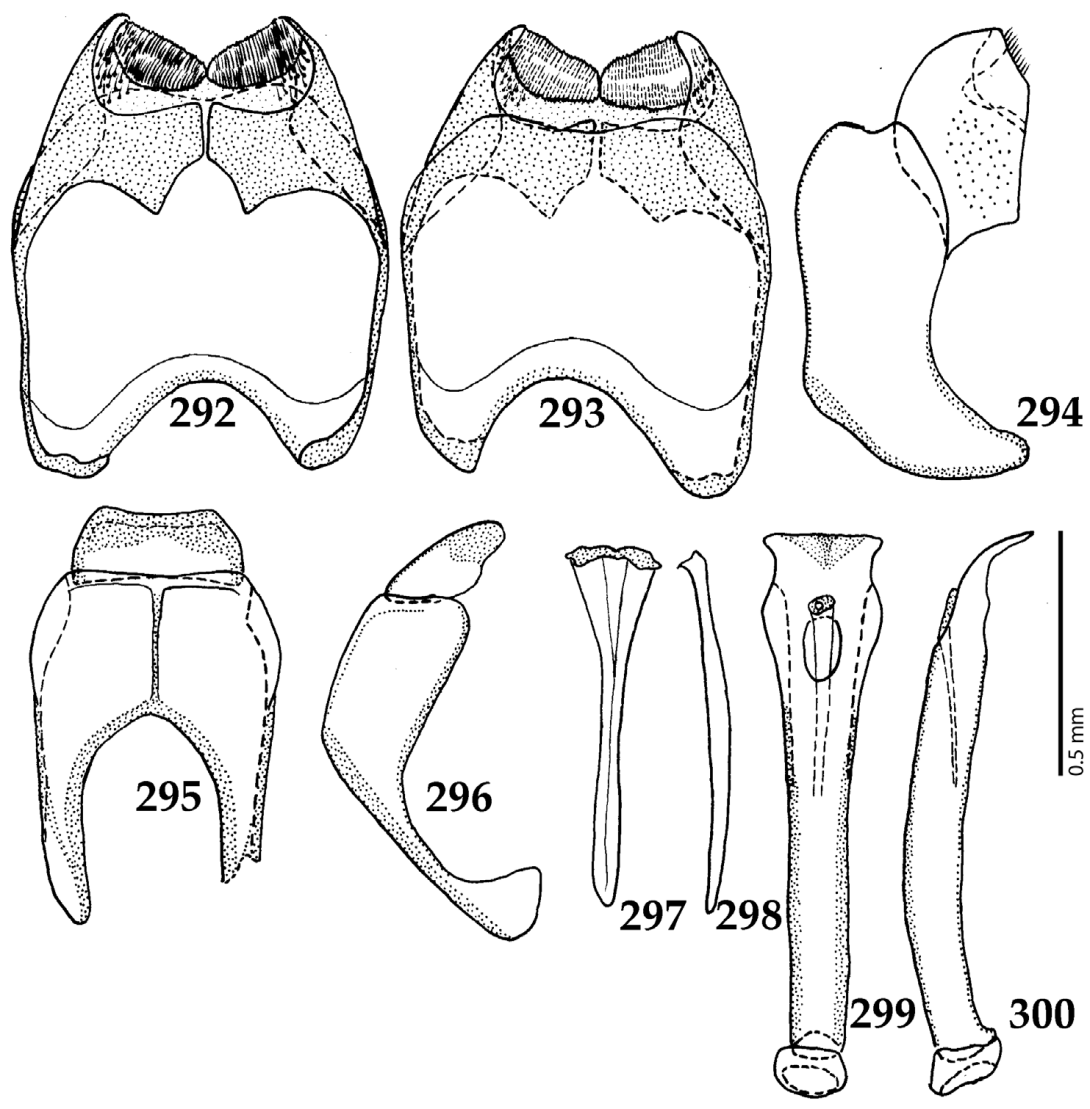
**292** *Saprinodes falcifer* Lewis, 1891 male terminalia: 8^th^ sternite + 8^th^ tergite, ventral view **293** ditto, dorsal view **294** ditto, lateral view **295** male terminalia: 9^th^ + 10^th^ tergites, dorsal view **296** ditto, lateral view **297** male terminalia: spiculum gastrale, ventral view **298** ditto, lateral view **299** male terminalia: aedeagus, dorsal view **300** ditto, lateral view.

Male genitalia. Eighth sternite (Figs 292–293) separated longitudinally; vela with sparse short setae; eighth tergite and eighth sternite not fused laterally (Fig. 294). Ninth tergite (Figs 295–296) longitudinally fused medially; spiculum gastrale (Figs 297–298) gradually dilated on apical half, basal end slender, not dilated. Aedeagus (Figs 299–300) slender, slightly widening apically, slightly constricted before apex; basal piece of aedeagus very short, ratio of its length: length of parameres 1 : 10; parameres of aedeagus fused almost along their entire length, with small circular aperture for median duct; aedeagus only slightly curved from lateral view, apex of aedeagus flattened dorso-ventrally.

#### Saprinus

Taxon classificationAnimaliaColeopteraHisteridae

Erichson, 1834

Figs 301 , 302–310 , 311–319 , 320 , 321–329 , 330–336 , 337 , 338–343 , 344–352 , 353 , 354–362 , 363–371 , 372 , 373–381 , 382–388 , 389 , 390–398 , 399–407 , 408 , 409–414 , 415–421 , 422 , 423–431 , 432–438 , 439 , 440–448 , 449–457 , 458 , 459–467 , 468–476 , 477 , 478–486 , 487–493 , 494 , 495–503 , 504–510 , 511 , 512–520 , 521–527 , 528 , 529–537 , 538–546 , 547 , 548–556 , 557–564 , 565 , 566–577 , 578–584 , 585 , 586–594 , 595–602 , 603 , 604–611 , 612–618 , 753 , 755 , 758 , 759 , 760 , 761 , 762 , 763 , 764 , 765

Saprinus Erichson, 1834: 172. Type species Hister nitidulus Fabricius, 1801 (=Saprinus semistriatus (L.G. Scriba, 1790)), designated by Westwood (1838): 22.

##### Diagnosis.

Some members of *Saprinus* are the largest of the Australian saprinines with specimens of *S.* (*S.*) *viridanus* measuring up to 6.70 mm (PEL). The genus is also the most species-rich; it contains 18 species, including 4 newly described and 2 introduced species. All Australopacific species (with the exception of *S. detritus*, *S. pseudodetritus*, and *S. chathamensis* from New Zealand/Chatham Islands, and S. *nitiduloides* Fairmaire, 1883 from New Britain/Solomon Islands and *S. artensis* Marseul, 1862 from New Caledonia that are dark brown) are characterized by strong metallic luster on the dorsum of their bodies, especially on elytra, and absence of red, orange or yellow elytral patches (often present in Palaearctic, Oriental and African species). Likewise, all have complete frontal stria (with the exception of *S. grandiclava* from New Guinea and two newly described species from the Chatham Islands). The frontal stria is usually widely interrupted in the Palaearctic or African species and is used to diagnose the Palaearctic members of *Saprinus*. One species, *S. viridipennis* (Australia), lacks carinal prosternal striae and two species, *S. grandiclava* (New Guinea) and *S. viridanus* (Australia), share unusually large, circular antennal clubs.

**Figure 301.**
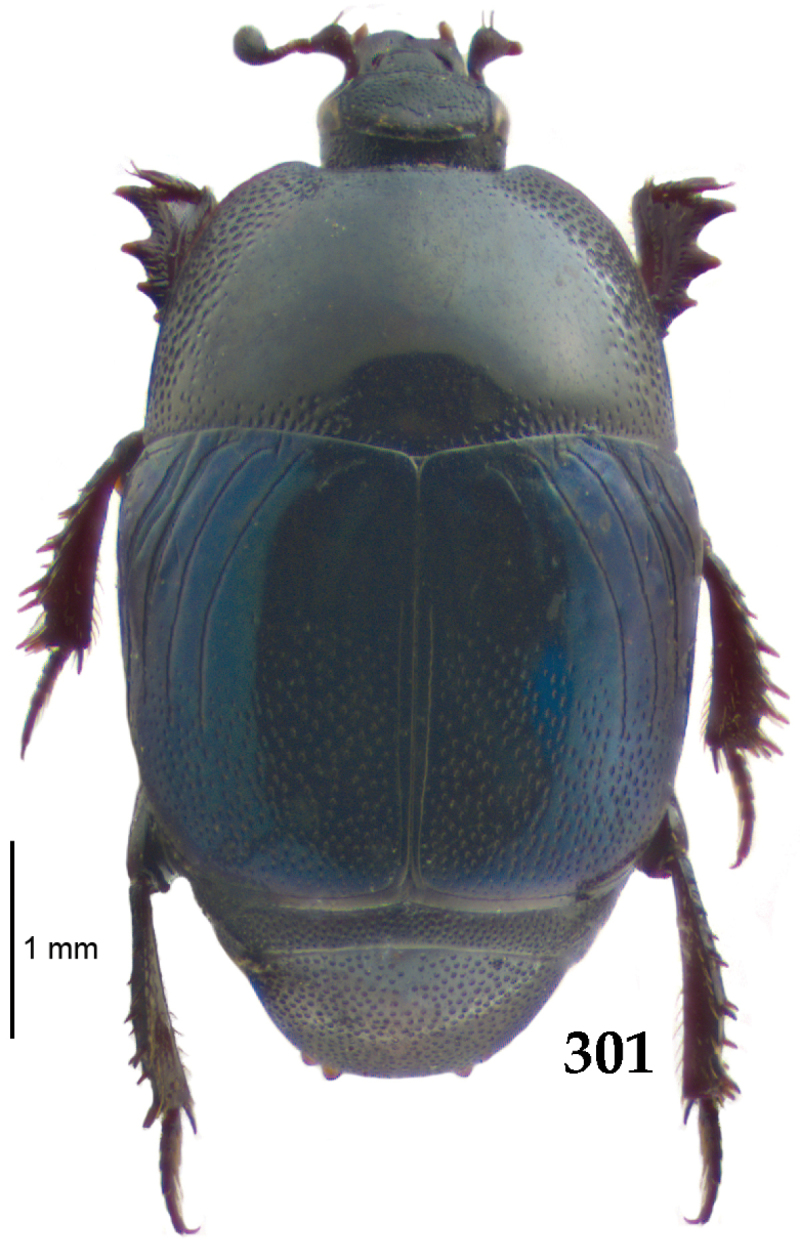
Saprinus (Saprinus) amethystinus Lewis, 1900 habitus, dorsal view.

An aedeagal peculiarity among Australopacific *Saprinus* is the presence of well-separated parameres (see also below; e.g. Fig. 475) in the Australian endemic species *S. amethystinus* Lewis, 1900, *S. australis* (Boisduval, 1835), *S. laetus* Erichson, 1834, and *S. tyrrhenus* Blackburn, 1903. The three New Zealand endemics, as well as *S. grandiclava* Kanaar, 1989 (endemic to New Guinea), have their parameres separated in the apical half and are more approximate than in the Australian species (Fig. 456).

##### Biology.

Species of *Saprinus* are typical volant predators with a preference for open grasslands and xerothermic habitats where they prey upon fly larvae found in decomposing organic matter, such as mammalian dung or carcasses (Lackner 2010). Their large and well-developed sensory patches on the ventral side of their antennal clubs probably enable them to locate the carrion from a long distance (but these are lacking in *S. grandiclava*). The natural history of several rare species (*S. grandiclava*, *S. amethystinus* and *S. tyrrhenus*) is completely unknown. *S. rarus* sp. n. was collected in the nest of the arboreal Tree termite (*Nasutitermes walkeri* (Hill, 1942)) and is presumed to be a specialised termitophile.

##### Distribution.

There are eighteen species of Australopacific *Saprinus*: nine native and two introduced (*S. chalcites* and *S. cupreus*) are known from Australia; three species of *Saprinus* are present in New Zealand/Chatham Islands; New Caledonia has one endemic and one non-endemic species. New Guinea has a single endemic species, with two other, non-endemic congeners. We describe one endemic species from the Kiribati atoll (Figs 753, 755, 758–765).

##### Remarks.

Australopacific species of the genus *Saprinus* differ from the similar-looking species *Notosaprinus irinus* (Marseul, 1862) by the presence of lateral prosternal striae (usually both carinal and lateral prosternal striae are present). See discussion of *Notosaprinus* for more details. The genus *Saprinus* is the most species-rich genus of the subfamily (see e.g. Lackner 2010 or Mazur 2011 for details) with the bulk of its representatives occurring in the Palaearctic and Afrotropical Regions. Although moderately diverse also in the Oriental (Indo-Malayan) and Nearctic Regions, *Saprinus* of the Australopacific Region with 16 native species are rather species-poor when compared to their counterparts from the other regions. Only the Neotropical Region, with only 7 known species has fewer than Australopacific Region.

Based on the structure of the aedeagus there are two main groups of Australopacific *Saprinus*: in each group there are eight species with widely separated parameres and with fused parameres. Regarding the group of species with fused parameres, the following species are morphologically very similar: *S. cyaneus cyaneus*, *S. artensis*, *S. nitiduloides* and *S. pacificus* (compare Figs 335, 420, 493 and 510). Based on their overall similarities, including the structure of male genitalia we believe that they likely share a recent common ancestor that probably reached the region from the north, since a subspecies of *S. cyaneus*, *S. cyaneus auricollis*, is distributed also in the Indo-Malayan Region up to Southern Japan. These species can possibly be regarded among the so-called northern invaders, *sensu* Mitchell and Bouchard (2008). Another species with fused parameres, *Saprinus splendens*, is widely distributed in the tropical regions of Africa/Arabian peninsula and the Oriental (Indo-Malayan) Region as far east and north as Japan, and its occurrence in the Australopacific Region (Australia and Marianna Islands) is not surprising. The three remaining species with fused parameres (*S. rarus*, *S. viridanus*, and *S. viridipennis*) are Australian endemics. *Saprinus rarus* has a uniquely triangulate dilated and thickened antennal scape (Fig. 529), strongly dilated tibiae (Fig. 536) and elongate body shape (Fig. 528), features that are probably reflecting its termitophilous lifestyle, and may protect it against termites (thickened antennal club and dilated tibiae protect larger space of body venter when in repose). *S. viridipennis* lacks the carinal prosternal striae entirely (Fig. 609; an autapomorphic feature within *Saprinus*), while the antennal club of *S. viridanus* is unusually large and circular, resembling the form present in the New Guinean endemic *S. grandiclava* (compare Figs 460 and 586).

The remaining eight species with (widely) separate parameres (Australian *S. amethystinus*, *S. australis*, *S. tyrrhenus*, *S. laetus*; New Guinean *S. grandiclava* and New Zealand *S. detritus*, *S. pseudodetritus* and *S. chathamensis*) are unique among the 157 described species of *Saprinus* (*sensu* Mazur 2011): no other species of *Saprinus* have this character. In the published phylogenetic analysis of the senior author (Lackner 2014d) only the type species of the genus, S. (S.) semistriatus, a taxon with fused parameres, was analysed. As noted above (see remarks section of *Notosaprinus*), S. (S.) semistriatus was recovered sister to *Notosaprinus irinus*; this clade itself was recovered sister to a larger clade containing North American *Xerosaprinus*, African *Pilisaprinus* and *Paraphilothis*, Middle-Asian *Styphrus*, Palaearctic *Phaonius* (itself subgenus of *Saprinus*). None of these relatives of *Saprinus* possess separated parameres. On the other hand, two members of the genus *Microsaprinus* Kryzhanovskij, 1976 that was recovered in the aforementioned analysis closer to the root, possess separated parameres of the aedeagus (albeit not widely; see Secq and Secq 1995 for figures). Looking at the character value nodes of *Saprinus* near and even more distant relatives the basal aedeagal condition does not appear therefore to be the separated-parameres character state, with the single exception of the two *Microsaprinus* species.

##### Key to the Australopacific species of *Saprinus* Erichson, 1834

**Table:** 

1 (16)	Pronotal hypomeron with scattered microscopic setae, or distinctly setose
2 (9)	Protibia on outer margin with approximately 5 low teeth topped by denticle (Fig. 466)
3 (4)	Bi-colored species (Fig. 458), elytra usually dark blue; pronotum often metallic green; parameres of aedeagus widely separated on their apical half (Fig. 475)	**S. (S.) laetus Erichson, 1834** (Australia, Lord Howe Island)
4 (3)	Unicolored, castaneous to dark brown species without metallic luster (Fig. 511); parameres of aedeagus not widely separated (Fig. 526) (New Zealand and Chatham Islands species)
5 (6)	Dorsum matte, with alutaceous microsculpture, apical third of elytra with aciculate punctures (Figs 511, 515)	**S. (S.) pseudodetritus sp. n.** (New Zealand: Chatham Islands)
6 (5)	Dorsum shining, with punctures but never with alutaceous microsculpture, apical third of elytra with simple, not aciculate punctures (Figs 372, 376)
7 (8)	Pronotum laterally with a band of deep, dense punctures (Fig. 422); eighth tergite not particularly narrowing apically, apex with a denser tuft of setae, apex of aedeagus dilated (Figs 432–438)	**S. (S.) detritus (Fabricius, 1775)** (New Zealand, Chatham Islands)
8 (7)	Pronotum completely glabrous (Fig. 372); eighth tergite gradually narrowing apically, apex with only several setae, apex of aedeagus not particularly dilated (Figs 382–388)	**S. (S.) chathamensis sp. n.** (New Zealand: Chatham Islands)
9 (2)	Protibia on outer margin with three large triangular teeth topped by denticle (Fig. 603)
10 (11)	Carinal prosternal striae absent (Fig. 609); setae of pronotal hypomeron long; male genitalia (Figs 612–618): eighth sternite and tergite rather wide, their width more than half their length; apex of eighth sternite with only few microscopic sparse setae; aedeagus on apical third dilated; apex with a cluster of tiny setae (best visible from lateral view)	**S. (S.) viridipennis Lewis, 1901** (Australia)
11 (10)	Carinal prosternal striae present (Fig. 589); setae of pronotal hypomeron rather short
12 (13)	Large species, PEL = 5.80–6.70 mm; antennal clubs large, circular (Fig. 587); parameres of aedeagus fused (Fig. 601)	**S. (S.) viridanus Lewis, 1899** (Australia)
13 (12)	Smaller species, PEL = max 3.85 mm; antennal clubs not large, oval to circular shaped (Fig. 303); parameres of aedeagus separated (Figs 318, 456)
14 (15)	Body elongate oval (Fig. 301); PEL = 3.25–3.85 mm; mesoventrite punctate (Fig. 306); male genitalia (Figs 311–319) eighth sternite strongly sclerotized, apically with several tiny setae; parameres of aedeagus separated nearly to base	**S. (S.) amethystinus Lewis, 1900** (Australia)
15 (14)	Body roundly oval (Fig. 565); PEL max 2.75 mm; mesoventrite smooth (Fig. 571); male genitalia (Figs 578–584): eighth sternite weakly sclerotized, apex with a tuft of sparse short setae; aedeagus medially widened, parameres separated in apical half only	**S. (S.) tyrrhenus Blackburn, 1903** (Australia)
16 (1)	Pronotal hypomeron glabrous
17 (18)	Antennal club unusually large, almost heart-shaped (Fig. 441); ventral surface of antennal club without any apparent sensory areas or patches (Fig. 442)	**S. (S.) grandiclava Kanaar, 1989** (New Guinea)
18 (17)	Antennal club not unusually large, circular or oval; ventral surface of antennal club usually with visible sensory areas or patches (Fig. 549)
19 (20)	Elongate species (Fig. 528); tibiae dilated (Figs 536, 537); antennal scape thickened and triangularly dilated (Fig. 529)	**S. (S.) rarus sp. n.** (Australia)
20 (19)	Roundly oval species (Fig. 389); tibiae not particularly dilated (Figs 397, 398); antennal scape not particularly thickened or triangularly dilated (Fig. 390)
21 (24)	Smaller species, PEL = max 2.75 mm, often frontal stria widely interrupted (Fig. 390)
22 (23)	Punctures of elytra very dense, interspaces between them almost non-existent (Fig. 389); male genitalia (Figs 399–407): eighth sternite apically with a tuft of long dense setae; spiculum gastrale (ninth sternite) dilated on both ends; aedeagus widened at apex, strongly curved in lateral view	**S. (S.) cupreus Erichson, 1834** (species adventive to Australia)
23 (22)	Punctures of elytra moderately dense, interspaces between them approximately as large as punctures themselves (Fig. 353); male genitalia (Figs 363–371): eighth sternite apically with a dense brush of short setae; apex of aedeagus only moderately widened	**S. (S.) chalcites (Illiger, 1807)** (species adventive to Australia)
24 (21)	Larger species, PEL = min 3.15 mm, frontal stria in most cases complete, rarely slightly interrupted medially (Fig. 338)
25 (26)	Third dorsal elytral stria always strongly shortened apically, with fourth stria as long as second (Fig. 337); elytra usually dark blue, pronotum normally dark brown with bronze metallic luster; parameres of male aedeagus widely separated (Fig. 351)	**S. (S.) australis (Boisduval, 1835)** (Australia)
26 (25)	Third dorsal elytral stria normally never strongly shortened apically, while fourth stria can be shorter than second (Fig. 408)
27 (28)	Fourth dorsal elytral stria shortened on basal third (Fig. 547); dorsal elytral striae containing weak tiny punctures; aedeagus near basal third briefly dilated (Fig. 561)	**S. (S.) splendens (Paykull, 1811)** (widely distributed species in Afrotropical and Oriental Regions; in Australopacific Region present in Australia, Papua New Guinea and Marianna Islands)
28 (27)	Fourth dorsal elytral stria normally not shortened on basal third (Fig. 408); dorsal elytral striae containing larger round punctures; aedeagus near basal third not particularly dilated (Figs 420, 510)
29 (30)	Usually bi-colored species (Fig. 408), male genitalia (Figs 415–421): eighth sternite narrowing apically; apex with a sparse cluster of tiny setae; apical angles of tenth tergite strongly sclerotized; aedeagus from dorsal view parallel-sided, slightly curved from lateral view, apex ventrally with a brush of tiny setae (best visible from lateral view) (Australia, Lord Howe Island, New Guinea, New Caledonia, Fiji)	**S. (S.) cyaneus cyaneus (Fabricius, 1775)**
30 (29)	Unicolored species (Fig. 320) from New Caledonia, New Guinea: New Britain, Solomon Islands and Kiribati, best recognized among each other on basis of their male terminalia
31 (32)	Apical half of elytra densely punctate (Fig. 320), interspaces approximately the size of the punctures; male genitalia (Figs 330–336): eighth sternite apically sub-rectangular, with dense short setae; apices setose	**S. (S.) artensis Marseul, 1862** (New Caledonia)
32 (31)	Apical half of elytra sparsely punctate (Fig. 477), interspaces larger than the size of the punctures
33 (34)	Male terminalia (Figs 487–493): eighth sternite apically with two small velae, adorned with a cluster of microscopic setae	**S. (S.) nitiduloides Fairmaire, 1883** (Papua New Guinea: New Britain; Solomon Islands)
34 (33)	Male terminalia (Figs 504–510): eighth sternite strongly narrowing anteriorly, apically without two small velae; apex of eighth sternite densely setose; aedeagi generally similar between the three preceding species	**S. (S.) pacificus sp. n.** (Kiribati)

#### Saprinus (Saprinus) amethystinus

Taxon classificationAnimaliaColeopteraHisteridae

Lewis, 1900

Figs 301 , 302–310 , 311–319 , 759

Saprinus amethystinus Lewis, 1900: 253.

##### Type locality.

Australia: Queensland: Taylor Range.

##### Type material examined.

*Saprinus amethystinus* Lewis, 1900: Lectotype, present designation: ♂, side-mounted, terminalia and pygidium glued to the same card as the specimen, right antennal funicle missing, right mid-leg missing, right metatarsus missing, left protarsal claw missing, with the following labels: “Taylor Range / Queens Land / (Janson)” (written); followed by: “*Saprinus* / *amethystinus* / Type Lewis” (written); followed by: “G. Lewis Coll. / B.M. 1926-369.” (printed); followed by: “Type” (round, red-margined label); followed by: “09-088” (yellow, pencil-written label, added by the senior author); followed by: “Saprinus / amethystinus / LEWIS, 1900 / LECTOTYPE / des. T. Lackner ‘011” (red label, written) (BMNH). This species was described from an unknown number of specimens and the lectotype designation fixes the species identity.

##### Additional material examined.

AUSTRALIA. New South Wales: 1 ♂, Quirindi, G.E. Bryant, 2.xi.[19]08, G. Bryant Coll., 1919–147, Dahlgren det. (BMNH); 1 spec., Caragabal, 13.ix.1966, Z. Liepa (ANIC); 10 specs., Euglo ex., Humbug Creek, 12.xi.1972, D.A. Doolan leg. (AMS; 3 specs. in coll. TLAN). Queensland: 1 ♀, Urangan, Manski, collector unknown (ANIC).

##### Biology.

Unknown.

##### Distribution.

Australia: New South Wales and Queensland (Fig. 759).

##### Remarks.

This is a rare species of Australian *Saprinus* known from only a handful of specimens; the last ones were collected in 1972.

##### Re-description.

Body length: PEL: 3.25–3.85 mm; EL: 2.00–2.40 mm; APW: 1.25–1.60 mm; PPW: 2.25–2.75 mm; EW: 2.50–3.00 mm.

Body (Fig. 301) rectangular oval, dorsally convex, ventrally rather flattened; cuticle dark brown, shining, pronotum darker, piceous black, with bronze metallic luster; elytra on basal third light brown, rest of elytra darker, elytra with dark blue metallic luster; legs, mouthparts and antennal scape castaneous brown; antennal club light brown.

**Figures 302–310.**
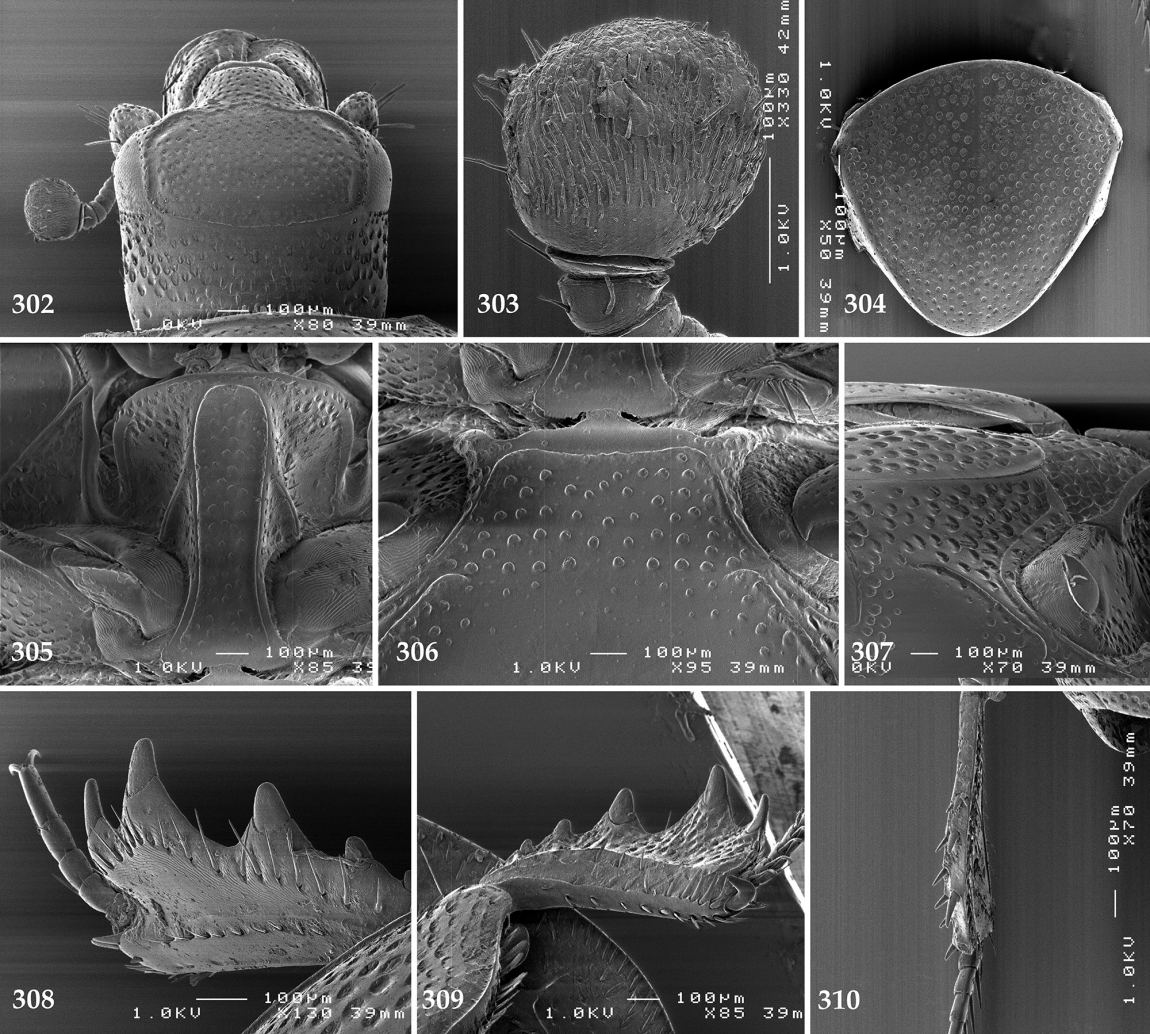
**302** Saprinus (Saprinus) amethystinus Lewis, 1900 head, dorsal view **303** antennal club, dorsal view **304** pygidium **305** prosternum **306** mesoventrite **307** lateral disc of metaventrite + metepisternum **308** protibia, dorsal view **309** ditto, ventral view **310** metatibia, dorsal view.

Antennal scape (Fig. 302) thickened and triangularly dilated, punctuate, with several setae; antennal club (Fig. 303) covered with dense short sensilla intermingled with sparse longer erect setae; lower third of club asetose; sensory structures of antennal club not examined.

Mandibles carinate laterally, dorso-laterally densely and coarsely punctuate, rounded, mandibular apex acute; mentum square-shaped, anterior margin with deep conspicuous median notch; labrum finely and sparsely punctuate, convex, with shallow median depression; labral pits present, each with a single labral seta; other mouthparts not examined.

Clypeus (Fig. 302) evenly densely punctuate; frontal stria weakened, but complete and outwardly arcuate medially, supra-orbital stria carinate; frontal disc (Fig. 302) coarsely and densely punctuate; eyes convex, well visible from above.

Pronotal sides (Fig. 301) moderately narrowing anteriorly, apical angles acute, pronotal depressions absent, anterior incision for head shallow, semicircular; marginal pronotal stria complete, carinate, visible along its entire length from dorsal view; pronotal disc laterally with a band of deep dense elongate punctures, becoming finer and sparser behind head, median part of pronotal disc almost smooth, with only scattered microscopic punctation; double row of fine ovoid punctures present along pronotal base; pronotal hypomeron with short yellow setae; scutellum very small, but visible.

Elytral epipleura with sparse fine punctures; marginal epipleural stria complete; marginal elytral stria well impressed and slightly carinate, continued as complete apical elytral stria. Humeral elytral stria well impressed on basal third, in most cases connected to inner subhumeral stria; variously long fragment of inner subhumeral stria present also laterad of humeral stria (in two of the three studied specimens); three dorsal elytral striae 1–3 well impressed, impunctate, carinate, apically slightly surpassing elytral half, fourth dorsal elytral stria strongly abbreviated, present as short basal fragment, basally not connected with sutural elytral stria; sutural elytral stria well-impressed, in punctures, abbreviated on basal fifth, apically connected with apical elytral stria; elytral disc on apical four-fifths punctuate, punctures becoming denser apically, forming almost elongate strioles near elytral apex.

Propygidium with dense round deep punctures separated by less than their diameter intermingled with much finer and sparser punctures; pygidium (Fig. 304) with similar, if somewhat sparser punctation; punctures much larger than those of propygidium.

Anterior margin of median portion of prosternum (Fig. 305) almost straight, rounded laterally; marginal prosternal stria present laterally and also as a rather long medial fragment; prosternal process flattened, surface between carinal prosternal striae depressed, with coarse punctures, apico-laterally substrigulate-punctate; carinal prosternal striae well-impressed, carinate, sub-parallel, slightly divergent anteriorly, forming a rounded loop, united in front (Fig. 305); lateral prosternal striae carinate, rather short, apically attaining carinal prosternal striae at about four-fifths of their length, surface around them with microscopic setae.

Anterior margin of mesoventrite (Fig. 306) almost straight, slightly inwardly arcuate; discal marginal mesoventral stria well impressed, complete; disc with coarse and large punctation; meso-metaventral sutural stria largely absent, present only as two short lateral fragments; intercoxal disc of metaventrite flattened; disc of metaventrite for the most part almost smooth, only with scattered microscopic punctation, along posterior margin (especially in area behind hind coxae) denser and coarser punctation appears; lateral metaventral stria (Fig. 307) well impressed, carinate, almost straight, shortened; lateral disc of metaventrite (Fig. 307) slightly concave, with dense shallow large setigerous punctures; metepisternum (Fig. 307) with similar setigerous punctures, on basal two-thirds and on fused metepimeron punctures becoming much sparser, not setigerous; metepisternal stria present as short intermittent fragments.

Intercoxal disc of first abdominal ventrite completely striate laterally; disc along basal and lateral margins with deep round punctures becoming finer and sparser medio-apically.

Protibia (Fig. 308) slightly dilated, outer margin with three triangular teeth topped by large triangular denticle, second and third teeth conspicuously larger than first, followed by two tiny low teeth topped by denticle; setae of outer row regular, moderately spaced; protarsal groove shallow; anterior protibial stria present on basal two-thirds, next obliterated; setae of median row shorter and sparser than those of outer row; two tarsal denticles present near tarsal insertion; protibial spur (Fig. 309) massive, bent, growing out from apical margin of protibia; outer part of posterior surface obscurely variolate, separated from glabrous median part of posterior surface by definite carinate boundary; posterior protibial stria complete, almost along entire length with dense row of long well sclerotized setae; inner row of setae single, setae sparse, shorter than those of posterior protibial stria.

Mesotibia moderately dilated, outer margin with a row of sparse long denticles growing in size apically, another row of much shorter sparser denticles situated on anterior surface of mesotibia; setae of outer row regular, thick, almost as long as denticles themselves; setae of median row irregular, shorter and finer; posterior mesotibial stria not examined; anterior surface of mesotibia sparsely punctuate; anterior mesotibial stria almost complete; mesotibial spur stout, moderately long; apical margin of mesotibia anteriorly with two short denticles; inner margin of mesotibia with sparse row of long setae; claws of apical tarsomere slightly bent, shorter than half its length; metatibia (Fig. 310) slenderer and longer than mesotibia, in all aspects similar to it, but denticles on outer margin much shorter and sparser.

**Figures 311–319.**
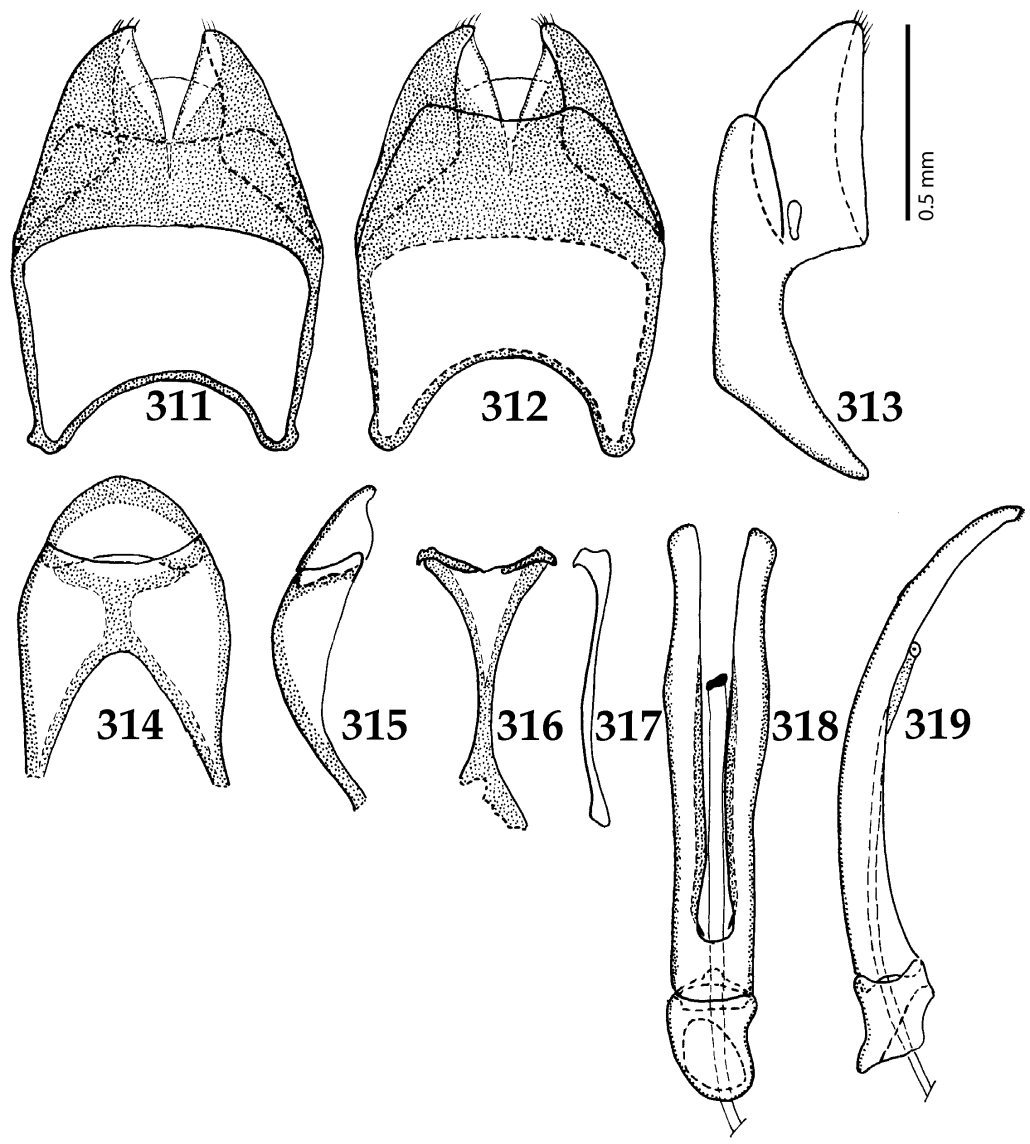
**311** Saprinus (Saprinus) amethystinus Lewis, 1900 male terminalia: 8^th^ sternite + 8^th^ tergite, ventral view **312** ditto, dorsal view **313** ditto, lateral view **314** male terminalia: 9^th^ + 10^th^ tergites, dorsal view **315** ditto, lateral view **316** male terminalia: spiculum gastrale, ventral view **317** ditto, lateral view **318** male terminalia: aedeagus, dorsal view **319** ditto, lateral view.

Male genitalia. Eighth sternite (Figs 311–312) strongly sclerotized, separated medially approximately on its apical half, apex with several microscopic setae, velum absent; eighth tergite and eighth sternite fused laterally (Fig. 313). Ninth tergite (Figs 314–315) typical for the subfamily; tenth tergite (Fig. 314) outwardly arcuate, rounded; spiculum gastrale (Figs 316–317) gradually dilated on most of its apical half; basal end slightly dilated. Aedeagus (Figs 318––319) with parameres separated almost on their entire length; basal piece of aedeagus short, ratio of its length : length of parameres 1 : 5; aedeagus slightly curved from lateral view (Fig. 319).

#### Saprinus (Saprinus) artensis

Taxon classificationAnimaliaColeopteraHisteridae

Marseul, 1862

Figs 320 , 321–329 , 330–336 , 760

Saprinus artensis Marseul, 1862: 445.

##### Type locality.

New Caledonia.

**Figure 320.**
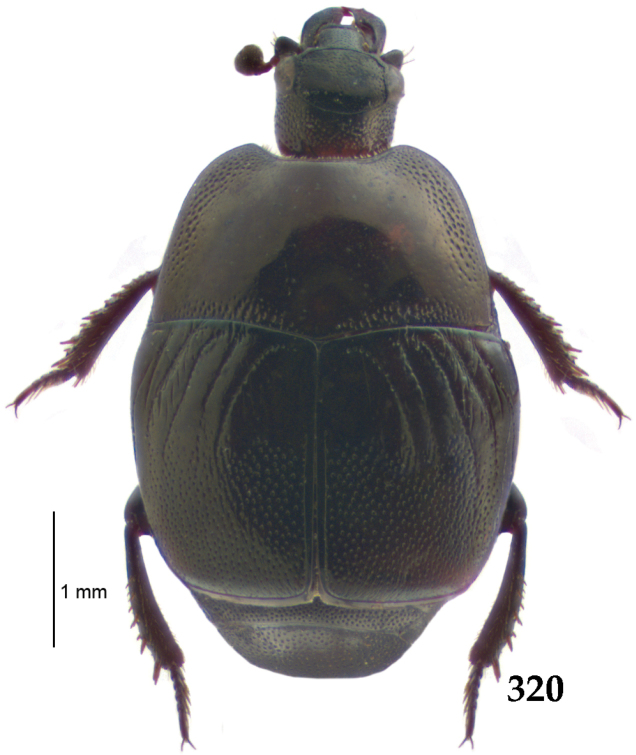
Saprinus (Saprinus) artensis Marseul, 1862 habitus, dorsal view.

##### Type material examined.

*Saprinus artensis* Marseul, 1862: Lectotype, present designation: ♂, side mounted on a triangular card, with terminalia glued on the same triangular card as specimen, right protarsus and left mesotarsus missing, both metatarsal claws broken off, with the following labels: “13 / *Saprinus* / *artensis* m. / illegible text” (round pink label, written); followed by: “*artensis* / type” (pencil-written label added probably by G. Dahlgren); followed by: “TYPE” (red-typed label); followed by: “MUSEUM PARIS / *artensis* / COLL / DE MARSEUL 1890” (pink label, typed-written); followed by: “09-052” (yellow, pencil-written label, added by the senior author); followed by: “Saprinus / artensis / Marseul, 1862 / LECTOTYPE / Des. T. Lackner ’11” (red label, written) (MNHN). This species was described from an unknown number of specimens and the lectotype designation fixes the identity of species.

##### Additional material examined.

1 ♂, New Caledonia (MNHN). Apparently this specimen is also from Marseul’s collection, but does not originate from the same sampling as the lectotype. There is no “Type” label on it and therefore it is not designated as the paralectotype; 5 ♂♂, “New Caledonia”, all from Schmidt’s collection, one male has a small label: “Type” – it is not clear whether it forms a part of the syntype series or not (ZMHUB); 1 ♀, New Caledonia, A. Deyr[olle] (NCB). 1 ♂ & 1 ♀, N. Caledonia, Fauvel (BMNH).

##### Biology.

Unknown, presumably similar to congeners.

##### Distribution.

Endemic to New Caledonia, rarely collected (Fig. 760).

##### Re-description.

Body length: PEL: 3.35 mm; EL: 2.00 mm; APW: 1.25 mm; PPW: 2.50 mm; EW: 2.80 mm (only the type specimen was measured).

Body (Fig. 320), convex, cuticle dark brown, almost black, shining, without metallic luster; legs, mouthparts and antennal scape dark brown; antennal club black.

**Figures 321–329.**
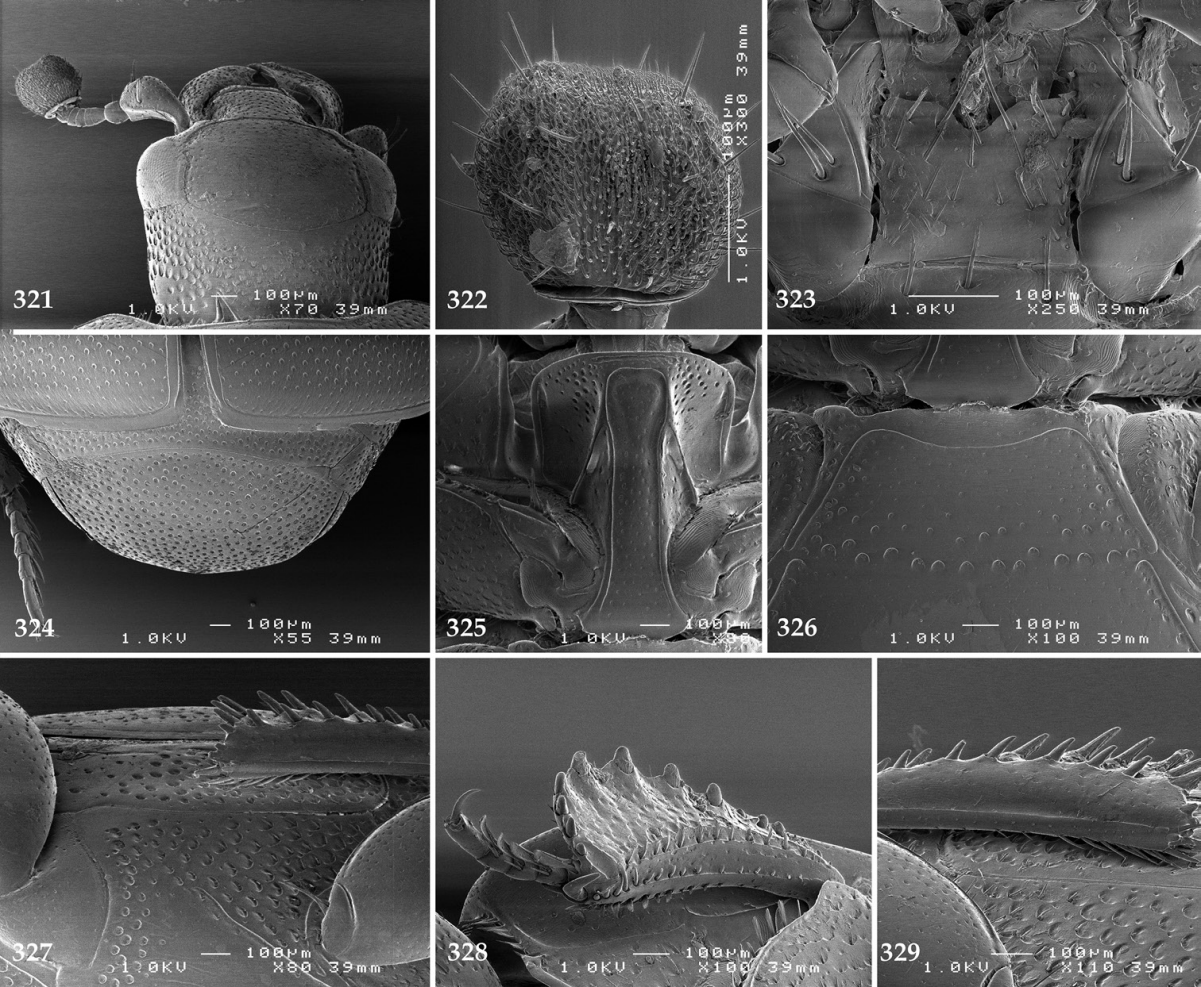
**321** Saprinus (Saprinus) artensis Marseul, 1862 head, dorsal view **322** antennal club, ventral view **323** mentum, ventral view **324** propygidium + pygidium **325** prosternum **326** mesoventrite **327** lateral disc of metaventrite + metepisternum **328** protibia, ventral view **329** mesotibia, ventral view.

Antennal scape (Fig. 321) black, slightly thickened, finely punctuate, with two setae; antennal club (Fig. 322) covered with dense short sensilla intermingled with sparse longer erect setae; sensory structures of antennal club not examined.

Mandibles dorso-laterally densely punctuate, rounded; labrum finely and sparsely punctuate, convex, with shallow median depression; labral pits present, each with a single labral seta; mentum (Fig. 323) almost quadrate, furnished with sporadic seate, anterior margin with deep triangular notch furnished with several longer setae; other mouthparts not examined.

Clypeus (Fig. 321) broad, flattened, sloping down laterally, its punctation denser and coarser than that of labrum; frontal and supra-orbital striae complete, slightly carinate; frontal disc (Fig. 321) finely punctuate, punctures separated by about several times their diameter; eyes convex, well visible from above.

Pronotal sides (Fig. 320) moderately narrowing anteriorly, apical angles obtuse, pronotal depressions shallow but present, anterior incision for head shallow; marginal pronotal stria complete, slightly carinate, visible along its entire length from dorsal view, weakened anteriorly; pronotal disc laterally with a band of deep dense elongate punctures originating approximately in pronotal depressions, but not reaching basal angles of pronotum, between it and pronotal margin a narrow smooth band present; rest of the pronotal disc almost smooth, with only scattered microscopic punctation; double row of fine ovoid punctures present along pronotal base not reaching ante-scutellar area; pronotal hypomeron glabrous; scutellum small, visible.

Elytral epipleura with a double row of fine punctures; marginal epipleural stria complete; marginal elytral stria well impressed and slightly carinate, continued as complete apical elytral stria. Humeral elytral stria well impressed on basal fourth, faintly connected to rather long inner subhumeral stria; four dorsal elytral striae 1–4 well impressed, all about the same length (third elytral stria slightly shorter than others), not reaching elytral half apically; first and second dorsal elytral striae in fine punctures, first elytral interval with elongate strioles; third and fourth striae in large sparsely set punctures, surface of second and third elytral intervals almost smooth, only with microscopic punctation; fourth dorsal elytral stria curved towards sutural elytral stria but not connected with it; sutural elytral stria well-impressed, in fine punctures, abbreviated on basal fourth, apically connected with apical elytral stria; elytral disc on apical 4/7 in dense large punctures; punctures separated by about their own diameter, laterally and especially apically punctures aciculate; elytral flanks almost impunctate.

Propygidium (Fig. 324) very densely punctuate, punctures separated by less than their diameter; pygidium (Fig. 324) with similar, if somewhat sparser punctation, interspaces in both cases imbricate.

Anterior margin of median portion of prosternum (Fig. 325) almost straight, rounded laterally; marginal prosternal stria present laterally and also as apical fragment; prosternal process between carinal prosternal striae flat, punctuate; carinal prosternal striae carinate, parallel on basal three-fourths, on apical fourth slightly divergent, united in front (Fig. 325); lateral prosternal striae carinate, rather short, apically attaining carinal prosternal striae at about three-fourths of their length.

Anterior margin of mesoventrite (Fig. 326) distinctly inwardly arcuate; discal marginal mesoventral stria well impressed, carinate; disc with sparse fine punctation, punctures becoming larger near meso-metaventral suture; meso-metaventral sutural stria indicated by a row of large punctures; intercoxal disc of metaventrite flattened, with slight longitudinal median depression; disc of metaventrite for the most part almost smooth, along posterior margin several rows of punctation appears; lateral metaventral stria (Fig. 327) well impressed, carinate, almost straight, shortened; lateral disc of metaventrite (Fig. 327) slightly concave, with dense shallow large setigerous punctures; metepisternum (Fig. 327) similar, but with deeper and larger punctures without setae, on fused metepimeron punctures becoming much sparser; metepisternal stria present along fused metepimeron, along metepisternum present as short intermittent fragments.

Intercoxal disc of first abdominal ventrite almost completely striate laterally; disc along basal and lateral margins with shallow punctures of various sizes; rest of sternite with scattered microscopic punctation.

Protibia (Fig. 328) slightly dilated, outer margin with five low teeth topped by large denticle, denticles diminishing in size proximally, followed by three minute denticles; setae of outer, median rows, anterior protibial stria and protarsal groove not examined; protibial spur large, bent, growing out from apical margin of protibia; outer part of posterior surface (Fig. 328) slightly obscurely variolate, separated from finely punctuate and narrow median part of posterior surface by a definite stria bearing a row of setae; posterior protibial stria complete, bearing almost along its entire length dense row of setae becoming stiffer and longer apically; apical margin of protibia ventrally with three short denticles; inner row of setae single, setae leaf-like, short, diminishing in size basally.

Mesotibia (Fig. 329) slender, outer margin with a row of sparse long denticles growing in size apically, another row of much shorter sparser denticles situated on anterior surface of mesotibia; setae of outer row regular, thick, almost as long as denticles themselves; setae of median row shorter and finer; posterior mesotibial stria almost complete; anterior surface of mesotibia sparsely punctuate, interspaces imbricate; anterior mesotibial stria complete; mesotibial spur stout, short; apical margin of mesotibia anteriorly with two short denticles; inner margin of mesotibia with sparse row of short setae; claws of apical tarsomere bent, longer than half its length; metatibia slenderer and longer than mesotibia, in all aspects similar to it, but denticles on outer margin much shorter and sparser.

**Figures 330–336.**
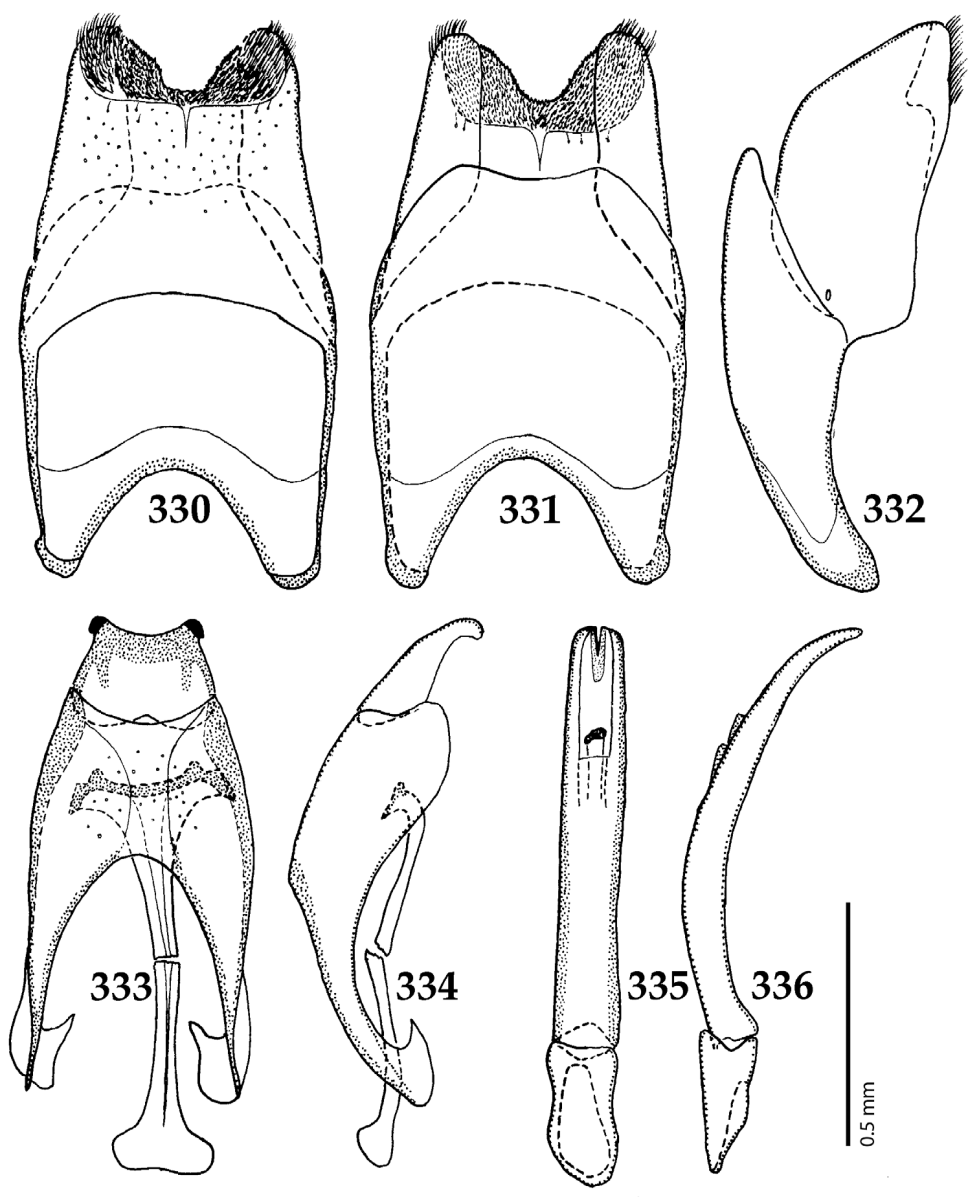
**330** Saprinus (Saprinus) artensis Marseul, 1862 male terminalia: 8^th^ sternite + 8^th^ tergite, ventral view **331** ditto, dorsal view **332** ditto, lateral view **333** male terminalia: 9^th^ + 10^th^ tergites, dorsal view; spiculum gastrale, ventral view **334** 9^th^ + 10^th^ tergites; spiculum gastrale, lateral view **335** male terminalia: aedeagus, dorsal view **336** ditto, lateral view.

Male genitalia. Eighth sternite (Figs 330–331) with pseudo-pores, fused medially, apex with large velum mesally adorned with dense rows of microscopic setae; laterally apex of eighth sternite with rows of longer dense setae; eighth tergite and eighth sternite not fused laterally (Fig. 332). Ninth tergite (Figs 333–334) typical for the subfamily; tenth tergite inwardly arcuate, apical angles strongly sclerotized, bent; spiculum gastrale (Fig. 333) gradually dilated on most of its apical half; basal end heart-shaped, inwardly arcuate. Aedeagus (Figs 335–336) parallel-sided, with parameres fused along their basal half (roughly), basal piece of aedeagus short, ratio of its length : length of parameres 1 : 4; aedeagus slightly curved from lateral view (Fig. 336).

#### Saprinus (Saprinus) australis

Taxon classificationAnimaliaColeopteraHisteridae

(Boisduval, 1835)

Figs 337 , 338–343 , 344–352 , 759

Hister australis Boisduval, 1835: 148.
Saprinus tasmanicus Marseul, 1855: 386 – Synonymized by Gemminger and Harold (1868): 783.

##### Type locality.

Australia.

**Figure 337.**
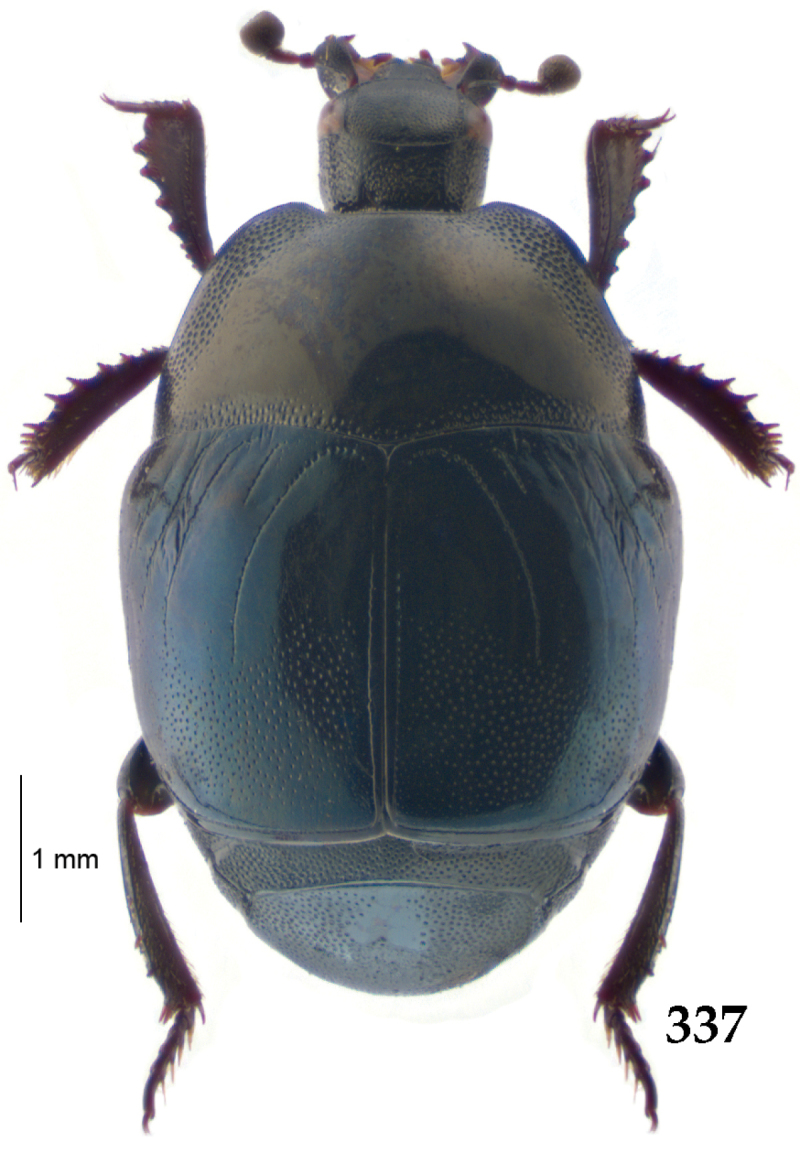
Saprinus (Saprinus) australis (Boisduval, 1835) habitus, dorsal view.

##### Type material examined.

*Hister australis* Boisduval, 1835: none. The type material of this species has not been located despite our extensive search and numerous visits in several European museums and its whereabouts are currently unknown.

*Saprinus tasmanicus* Marseul, 1855: Lectotype, present designation: ♀, glued on a mounting card, three tarsomeres of the left protibia broken off, right meso-tarsus and right hind leg missing, with the following labels: tiny, yellow rectangular label, followed by: “Saprinus / tasmanicus / m. / Australia further illegible / Dej. / V. Diemen / T. Dej. 67” (pink round label, written); followed by: “TYPE” (red-printed label); followed by: “MUSEUM PARIS / tasmanicus / COLL. / DE MARSEUL 1890” (pink label, printed-written); followed by: “Saprinus tasmanicus / Marseul, 1855 / LECTOTYPE / des. T. Lackner 2014” (red label, written) (MNHN). This species has been described from unknown number of specimens and the lectotype designation fixes the identity of the species.

##### Additional material examined.

AUSTRALIA. Queensland: 1 spec., Queensland, without further data (ZMHUB); 3 specs., Ewan Road, 8 miles W Paluma, 15.i.1970, J.G. Brooks (fish lure) (ANIC); 3 specs., Mount Mee, 7 km SW, 27°05'S, 152°42'E, 12.x.1991, Tom Gush (under dead snake on road) (ANIC); 7 specs., Mt. Tambourine, xii.1919, H. Pottinger (QM); 2 specs., National Park, xii.1919, H. Hacker (QM); 1 spec., Brisbane, ii.1920, H. Pottinger (QM); 1 spec., Caloundra, 28.ix.1913, H. Hacker (QM); 1 spec., Kroombit Tops (Lower Dry Creek), 45 km SSW Calliope, 9.–19.xii.1983, G. Monteith & G. Thompson (open forest) (QM); 1 spec., McAfee’s Lookout, 300 m, 27°26'S, 152°53'E, 18.x.1999, G. Monteith (open forest, under dead goanna) (QM); 2 ♀♀ & 1 spec., Bally Knob Summit, 1100 m, 17°39'S, 145°30'E, 6.xii.1998–6.ii.1999, Monteith & Cook (open forest, FIT) (QM); 1 ♂, Mt. Spurgeon, 16°27'S, 145°11'E, 1100 m, 19.–22.xi.1997, D.J. Cook (open forest, dung traps) (QM); 1 ♂, Mt. Spec., 11.i.1969, J.G. Brooks, fish lure (ANIC). Australian Capital Territory: 1 spec., Canberra, 14.x.1965, E. Britton (coast road, under dead wombat) (ANIC). Victoria: 1 spec., Melbourne, without further data (ZMHUB). New South Wales: 1 ♂, Richmond River, N.S. Wales (BMNH); 1 spec., env. Port Macquarie, 5.iii.1965, Exp. Dr. J. Balogh (HNMH); 1 ♂, Great Dividing Range, Mt. Coricudgy, 941 m, 32°50.8'S, 150°17.8'E, 7.–9.x.2000, R. de Keyzer leg. (HMNH); 2 specs., Lilyvale, 9.ix.1972, D.A. Doolan (MAMU); 1 spec., Ulong, East Dorrigo, ii.–iv.1923, W. Heron (MAMU); 2 specs., Mooney Mooney near Gosford, 18.i.1980, B.J. Day & D.K. McAlpine (MAMU); 4 specs., Dorrigo, W. Herron (SAMA). South Australia: 4 specs., Kangaroo Island, Rocky River, xii.1934, Museum Expedition (SAMA). Tasmania: 4 ♂♂ & 5 ♀♀, St. Helena, 10 km NW, 12.xii.1981, Bornemissza leg. (HMNH). Unknown localities: 1 ♀, Ballaarat, 1938, without further data (BMNH); 1 spec., Australien, no further data (ZMHUB); 1 spec., Australia occid. 1192, no further data (HNMH); 1 spec., Dividing Range, Blackburn’s Collection, no further data (SAMA).

##### Biology.

Saprinus (S.) australis is a predator of the open landscape collected both on dung and on carcasses.

##### Distribution.

Australia: Tasmania, New South Wales, Queensland, Australian Capital Territory, and South Australia (Fig. 759).

##### Remarks.

Boisduval’s description (1835: 148) of *Hister australis* is very concise and cannot be used to differentiate the species from other Australian congeners. Gemminger and Harold (1868: 783) synonymized Marseul’s *Saprinus tasmanicus* with *Hister australis* without any explanation. It is possible that Gemminger and Harold had seen Boisduval’s type specimen(s) and based their synonymy on syntype examination. We base our determinations on numerous inspected specimens that had been previously identified as S. (S.) australis by worldwide authorities on the Saprininae, like Dahlgren or Kanaar. This species is well characterised by the strongly shortened third dorsal elytral stria, whereas the second and the fourth are almost the same length, slightly surpassing elytral half apically (Fig. 337).

##### Re-description.

Body length: PEL: 3.50–4.75 mm; EL: 2.00–3.00 mm; APW: 1.25–1.50 mm; PPW: 2.50–3.50 mm; EW: 3.00–4.00 mm.

Body (Fig. 337) rectangular oval, convex, elytra dark blue to black, shining, with slight metallic luster, pronotum dark bronze, metallic; legs, mouthparts and antennal scape castaneous brown; antennal club black.

**Figures 338–343.**
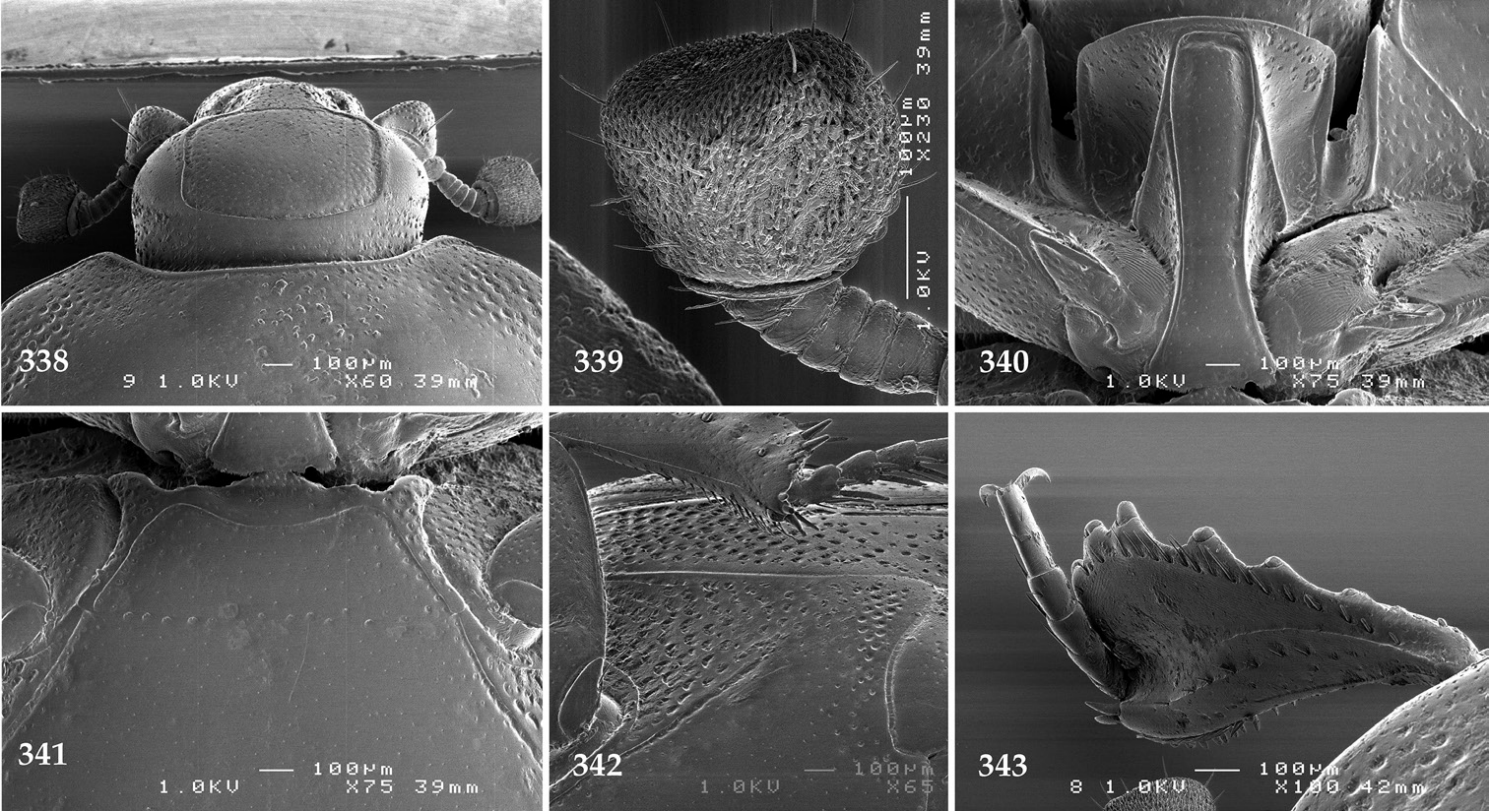
**338** Saprinus (Saprinus) australis (Boisduval, 1835) head, dorsal view **339** antennal club, ventral view **340** prosternum **341** mesoventrite **342** lateral disc of metaventrite + metepisternum **343** protibia, dorsal view.

Antennal scape (Fig. 338) slightly thickened, punctuate, with few setae; antennal club (Figs 339) wider than long, truncate apically, covered with dense short sensilla intermingled with sparse longer erect setae; sensory structures of antennal club in form of four elongate ventral sensory patches, vesicle(s) not examined.

Mandibles dorso-laterally finely punctuate, rounded, outer margin slightly carinate, mandibular apex acute, sub-apical tooth on left mandible obtuse; labrum finely and sparsely punctuate, convex, with deep median excavation; labral pits present, each with two labral setae; other mouthparts not examined.

Clypeus (Fig. 338) even, covered by punctures of various sizes separated by several times their diameter intermingled with microscopic punctation; frontal and supraorbital striae complete, occasionally frontal stria weakened medially; frontal disc (Fig. 338) with similar punctation as that of clypeus, but punctures denser; eyes convex, well visible from above.

Pronotal sides (Fig. 337) moderately narrowing anteriorly, apical angles obtuse, pronotal depressions present, anterior incision for head rather deep; marginal pronotal stria complete, carinate, visible along its entire length from dorsal view; pronotal disc laterally with a band of deep dense elongate punctures originating approximately in pronotal depressions, reaching basal angles of pronotum, between it and pronotal margin a narrow smooth band present; rest of the pronotal disc with only scattered microscopic punctation; several rows of punctures present along pronotal base weakening around ante-scutellar area; pronotal hypomeron glabrous; scutellum small, visible.

Elytral epipleura with sparse fine punctures; marginal epipleural stria complete; marginal elytral stria well impressed and slightly carinate, continued as complete apical elytral stria that is connected to incomplete sutural elytral stria. Humeral elytral stria well impressed on basal third, occasionally doubled, sometimes connected to short inner subhumeral stria; four dorsal elytral striae 1–4 well impressed, in fine punctures, first and second striae reaching approximately elytral half apically, third dorsal elytral stria always abbreviated apically, usually present only on basal elytral fourth, fourth stria slightly shorter than first or second, ending short of elytral mid-length, basally curved towards sutural elytral stria, but not connected with it (only occasionally connected with it); sutural elytral stria abbreviated on basal fifth, well-impressed, in fine punctures, apically connected with apical elytral stria; elytral disc on apical half (roughly) punctate, punctures fine, sparse, separated by several times their diameter; punctures becoming sparser and finer apically, occasionally not reaching elytral apex.

Propygidium very densely punctate, punctures separated by less than their own diameter, almost confluent; pygidium with similar, if somewhat sparser but larger punctation, interspaces in both cases imbricate.

Anterior margin of median portion of prosternum (Fig. 340) rounded; marginal prosternal stria present laterally and also as medial fragment; prosternal process between carinal prosternal striae slightly convex, sparsely and finely punctate, surface near united apices of carinal prosternal striae distinctly depressed; carinal prosternal striae carinate, bisinuate, united in front (Fig. 340); lateral prosternal striae carinate, rather short, apically attaining carinal prosternal striae at about three-fourths of their length.

Discal marginal mesoventral stria (Fig. 341) well impressed, carinate, complete; disc with sparse fine punctation, punctures separated by several times their diameter, occasionally punctation on mesoventrite coarser and denser, intermingled with much finer and sparser punctures; meso-metaventral sutural stria indicated by a row of large punctures; intercoxal disc of metaventrite in males with faint median longitudinal depression, in females convex; disc of metaventrite for the most part almost smooth, surface around longitudinal depression with scattered microscopic punctation, punctures of various sizes, along lateral and basal margin several rows of punctation appear; lateral metaventral stria (Fig. 342) well impressed, carinate, almost straight, shortened; lateral disc of metaventrite (Fig. 342) slightly concave, with dense and large setigerous punctures; metepisternum (Fig. 342) similar, but punctures deeper and smaller and without setae, on fused metepimeron punctures becoming much sparser; metepisternal stria present along fused metepimeron, along metepisternum present as short intermittent fragments.

Intercoxal disc of first abdominal ventrite completely striate laterally; disc along basal and lateral margins with shallow punctures of various sizes; rest of sternite with scattered microscopic punctation.

Protibia (Fig. 343) slightly dilated, outer margin with around 8 low teeth topped by large triangular denticle, denticles diminishing in size proximally; setae of outer row regular, short; protarsal groove shallow; anterior protibial stria present on basal two-thirds, next obliterated; setae of median row shorter and much sparser than those of outer row; two tarsal denticles present near tarsal insertion; protibial spur bent, growing out from apical margin of protibia; apical margin of protibia ventrally with three tiny denticles; outer part of posterior surface slightly obscurely variolate, separated from glabrous and narrow median part of posterior surface by a ridge bearing a row of setae; posterior protibial stria complete, bearing along its length sparse row of microscopic setae turning into several minuscule denticles apically; inner row of setae single, lamellate.

Meso-and metatibia similar to those of other congeners.

**Figures 344–352.**
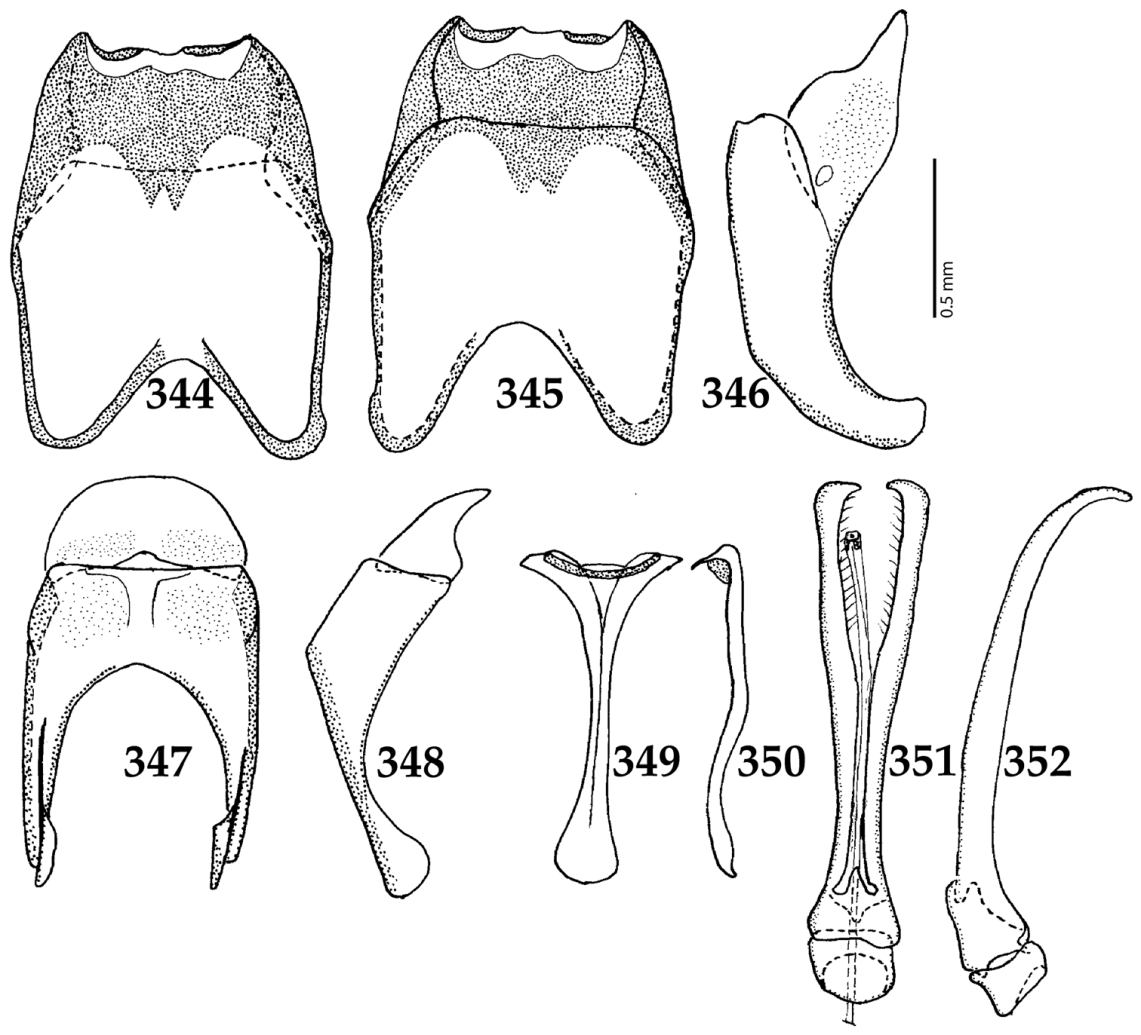
**344** Saprinus (Saprinus) australis (Boisduval, 1835) male terminalia: 8^th^ sternite + 8^th^ tergite, ventral view **345** ditto, dorsal view **346** ditto, lateral view **347** male terminalia: 9^th^ + 10^th^ tergites, dorsal view **348** ditto, lateral view **349** male terminalia: spiculum gastrale, ventral view **350** ditto, lateral view **351** male terminalia: aedeagus, dorsal view **352** ditto, lateral view.

Male genitalia. Eighth sternite (Figs 344–345) strongly sclerotized, fused medially, apex asetose, vela present, asetose; eighth tergite apically not emarginate, straight; eighth tergite and eighth sternite fused laterally (Fig. 346). Ninth tergite (Figs 347–348) typical for the subfamily; tenth tergite basally inwardly arcuate, rounded apically; spiculum gastrale (Figs 349–350) dilated on apical third, apex strongly sclerotized; basal end slightly dilated, outwardly arcuate. Aedeagus (Figs 351–352): parameres widely separated around mid-length (roughly), divergent apically, separated parameres with short regular setae mesally; basal piece of aedeagus short, ratio of its length : length of parameres 1 : 6; aedeagus almost straight, on apical fifth strongly curved from lateral view.

#### Saprinus (Saprinus) chalcites

Taxon classificationAnimaliaColeopteraHisteridae

(Illiger, 1807)

Figs 353 , 354–362 , 363–371 , 753

Hister chalcites Illiger, 1807: 40.

##### Type locality.

Portugal.

**Figure 353.**
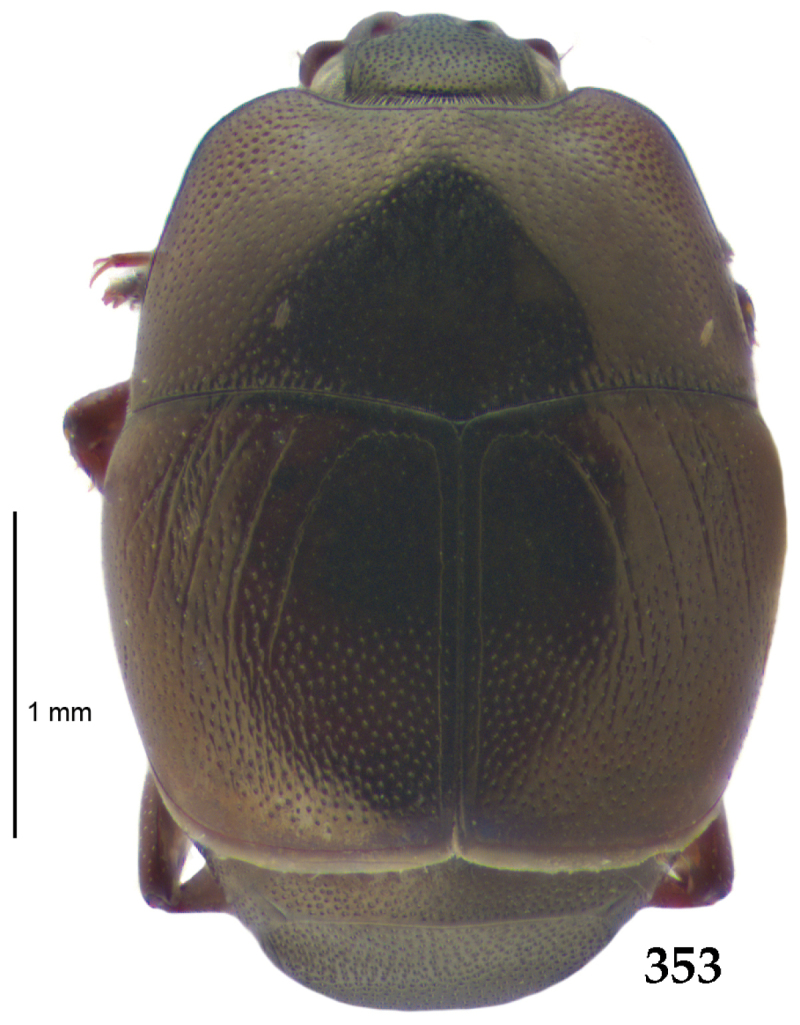
Saprinus (Saprinus) chalcites (Illiger, 1807) habitus, dorsal view.

##### Type material examined.

*Hister chalcites* Illiger, 1807: None for this study. For the problems with the identity of the type specimen(s) of this species the reader is referred to Lackner and Gomy (2016).

##### Additional material examined.

AUSTRALIA. Western Australia: 1 spec., Dardanup, 1.ii.1979, G. Hall (howden trap) (ANIC); 1 spec., Wilga, xi.1973, K. & E. Carnaby (ANIC); 1 spec., 64 km S of Boyup Brook, 6.xii.1976, K. & E. Carnaby (ANIC); 1 spec., Millstream, 21.35S 117.04E, 7.xi.1970, Kangaroo Flat, E. Britton (dung trap) (ANIC); 21 specs., 15 km S Busselton, 15.i.1983, G.P. Hall (ANIC); 7 specs., ditto, but 19.i.1983 (ANIC); 6 specs., ditto, but 12.i.1983 (ANIC); 3 specs., ditto, but 13.i.1983 (ANIC); 1 specs., ditto, but 11.i.1983 (ANIC); 6 specs., Minderoo Stations, Onslow, 31.x.1983, D. Forrest (ANIC); 2 specs., 5 km NE Dardanup, 16.x.1981, G.P. Hall (ANIC); 2 specs., Cataby, 30.44S 115.32E, 12.vi.1979, D. Majer (ANIC); 39 specs., 1 km N Cunderdin, 1.iii.1979, J.N. Matthiessen (ANIC); 1 ♂, Boyup Brook, xi. 1981, Ex. Carnaby coll. (ANIC). Northern Territory: 1 spec., Avon Downs St., 19.viii.1982, J. Harmer (ANIC).

**Figures 354–362.**
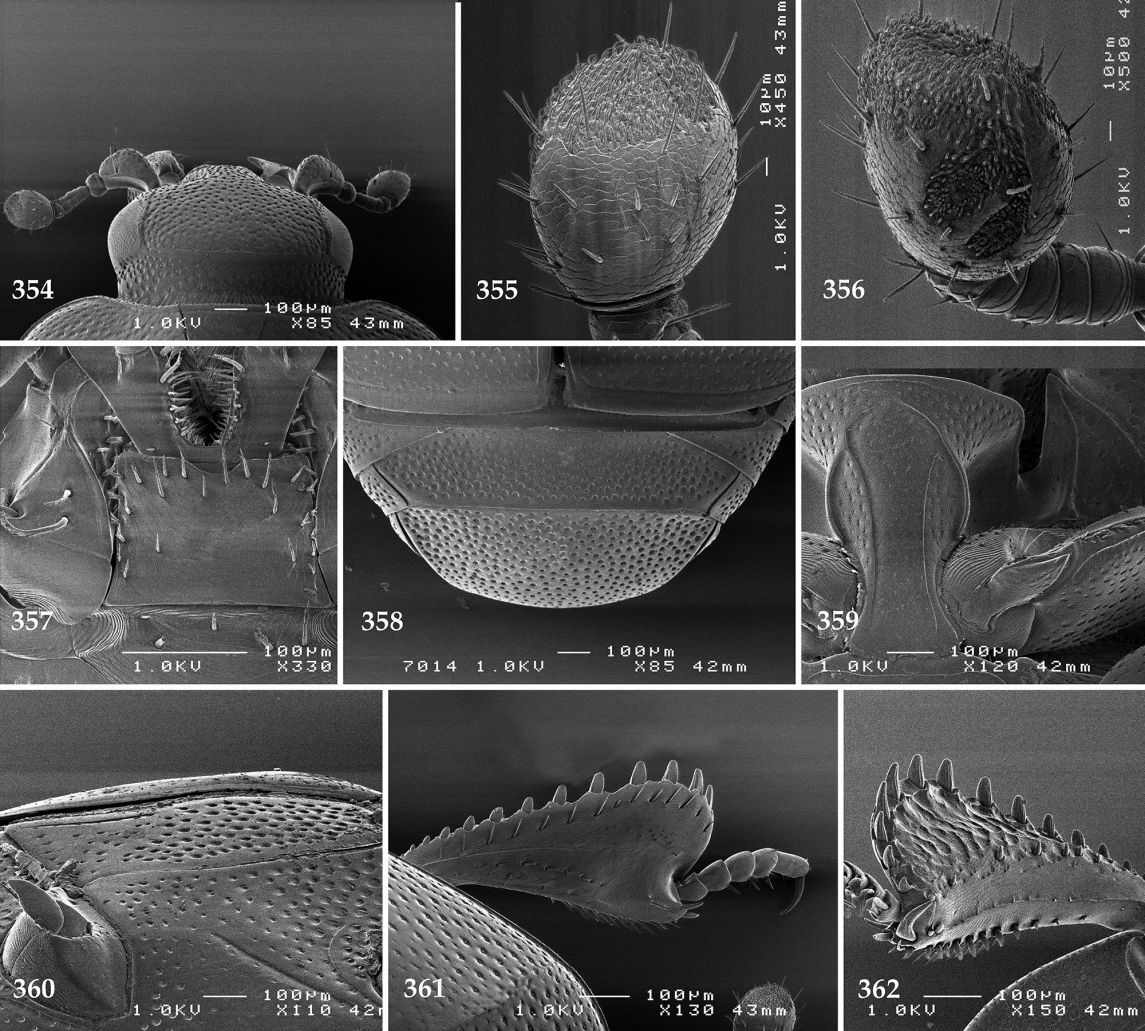
**354** Saprinus (Saprinus) chalcites (Illiger, 1807) head, dorsal view **355** antennal club, dorsal view **356** ditto, ventral view **357** mentum, ventral view **358** propygidium + pygidium **359** prosternum **360** lateral disc of metaventrite + metepisternum **361** protibia, dorsal view **362** ditto, ventral view.

##### Biology.

This species is a typical saprobiont and is most commonly collected on carcasses, and less commonly in dung.

##### Distribution.

Mediterranean Subregion, Africa, Arabian Peninsula, Middle Asia, India, Myanmar; introduced to Australia, where it has been documented from Western Australia and Northern Territory (Mazur 2011; Fig. 753).

##### Remarks.

Desbordes (1919: 413) synonymized the species *Saprinus certus* Lewis, 1888 with *S. chalcites*. This synonymy has been followed by all three Mazur’s editions of the world catalogue of the Histeridae (1984, 1997, 2011) and by Lackner et al. (2015). Upon inspection of its type specimen we can conclude that *Saprinus certus* Lewis, 1888 is not synonymous with *S. chalcites*, it is a synonym of *Saprinus frontistrius* Marseul, 1855 instead. Thus *Saprinus certus* Lewis, 1888 = *Saprinus frontistrius* Marseul, 1855 syn. n. We designate its lectotype here. *Saprinus certus* Lewis, 1888: lectotype, present designation, 1 spec., with the following labels: “Type” (red-margined, round printed label); followed by: “Rangoon / 35” (written); followed by: “Saprinus / certus / Type Lewis”; followed by: “Saprinus certus / Lewis, 1888 / LECTOTYPE / Det. T. Lackner 2014” (red label, written) (BMNH). This species was described from Rangoon [=Yangon, Myanmar] based on two examples. The whereabouts of the second specimen originating from the type series is unknown. The lectotype designation fixes the identity of species. *S. chalcites* is a widely distributed species, with rather variable punctation of the dorsum, always best recognizable by deep pronotal depressions and male terminalia. *S. chalcites* has been recently re-described in detail (Lackner and Gomy 2016). It can be distinguished from the rest of the Australopacific *Saprinus* species using the above key. For the sake of easier identification of the Australopacific taxa we chose to include here the photographs of its habitus, as well as SEM micrographs and male genitalia drawings.

**Figures 363–371.**
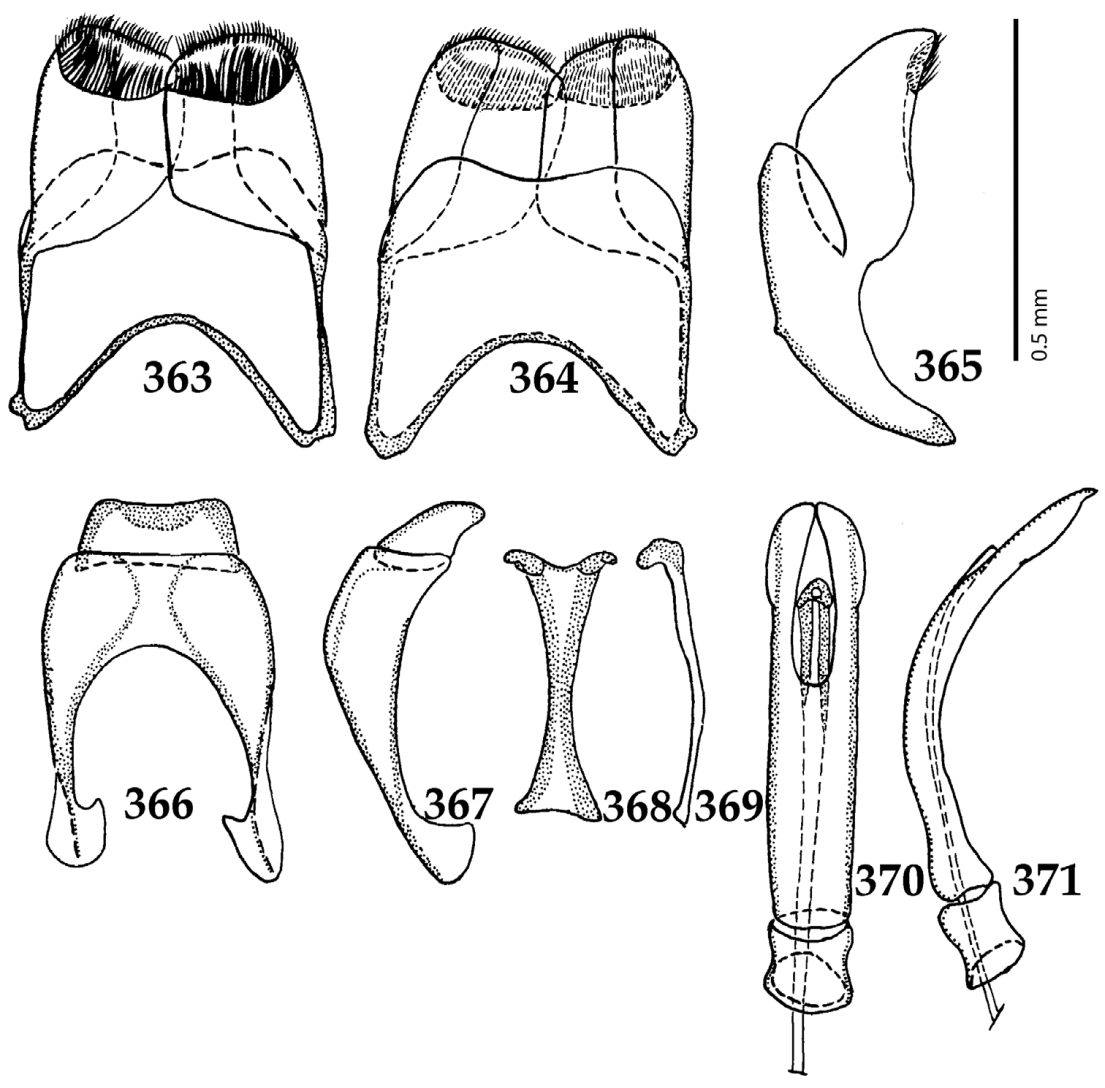
**363** Saprinus (Saprinus) chalcites (Illiger, 1807) male terminalia: 8^th^ sternite + 8^th^ tergite, ventral view **364** ditto, dorsal view **365** ditto, lateral view **366** male terminalia: 9^th^ + 10^th^ tergites, dorsal view **367** ditto, lateral view **368** male terminalia: spiculum gastrale, ventral view **369** ditto, lateral view **370** male terminalia: aedeagus, dorsal view **371** ditto, lateral view.

#### Saprinus (Saprinus) chathamensis sp. n.

Taxon classificationAnimaliaColeopteraHisteridae

http://zoobank.org/145A0716-BA5D-475E-91A7-E15C2A778A1E

Figs 372 , 373–381 , 382–388 , 761

##### Type locality.

New Zealand: Chatham Islands: Point Weeding: Waitangi.

##### Type material examined.

Holotype, ♂, side-mounted on a triangular card, with terminalia extracted and mounted in Canada Balsam on a separate slide under the specimen, with the following labels: “Chatham I. / Exp. Feb. 1967” (printed); followed by: “Pt. Weeding / Waitangi” (printed); followed by: “14.ii.[19]67 / beach” (printed-written); followed by: “G. Kuschel” (printed); followed by: “09-083” (yellow label, pencil-written); followed by: “Saprinus (Saprinus) / *chathamensis* sp. n. / HOLOTYPE / Lackner & Leschen 2010” (red label, printed) (NZAC). Paratypes, 6 ♀♀, 2 ♂♂ & 4 specs., same data as holotype, except for yellow label, that is present only on holotype (NZAC; 3 paratypes in coll. TLAN). 1 spec., same data as holotype, but “Owenga / 25.ii.[19]67” (NZAC). 4 ♀♀ & 10 specs., with following labels: “NEW ZEALAND CI / Pitt I, Waihere Bay / 21.i.1997 / J.W.M. Marris / under rotting pilot whale” (written); followed by: “ENTOMOLOGY / RESEARCH MUSEUM / (LUNZ) / Lincoln University, / Canterbury, New Zealand” (green label, printed); followed by: “Saprinus (Saprinus) / *chathamensis* sp. n. / PARATYPE / Lackner&Leschen 2010” (red label, printed) (LUNZ; 3 paratypes in coll. TLAN). 1 spec., with following labels: “NEW ZEALAND CH / Chatham I / Owenga Beach / 6 Dec 2004 / RM Emberson, P. Syrett” (printed); followed by: “Under kelp” (printed); followed by: “ENTOMOLOGY / RESEARCH MUSEUM / (LUNZ) / Lincoln University, / Canterbury, New Zealand” (green label, printed); followed by: “Saprinus (Saprinus) / *chathamensis* sp. n. / PARATYPE / Lackner&Leschen 2010” (red label, printed) (LUNZ). 1 spec., with following labels: “NEW ZEALAND CI / Pitt I, Waihere Bay / 21.i.1997 / R.M. Emberson / under rotting pilot whale” (printed); followed by: “AMNZ 49382 / AUCKLAND / MUSEUM / NEW ZEALAND”; followed by: “AMNZ 4938**2** / AUCKLAND / MUSEUM NEW ZEALAND” (green label, printed) followed by: “Saprinus (Saprinus) / *chathamensis* sp. n. / PARATYPE / Lackner&Leschen 2010” (red label, printed) (AMNZ).

**Figure 372.**
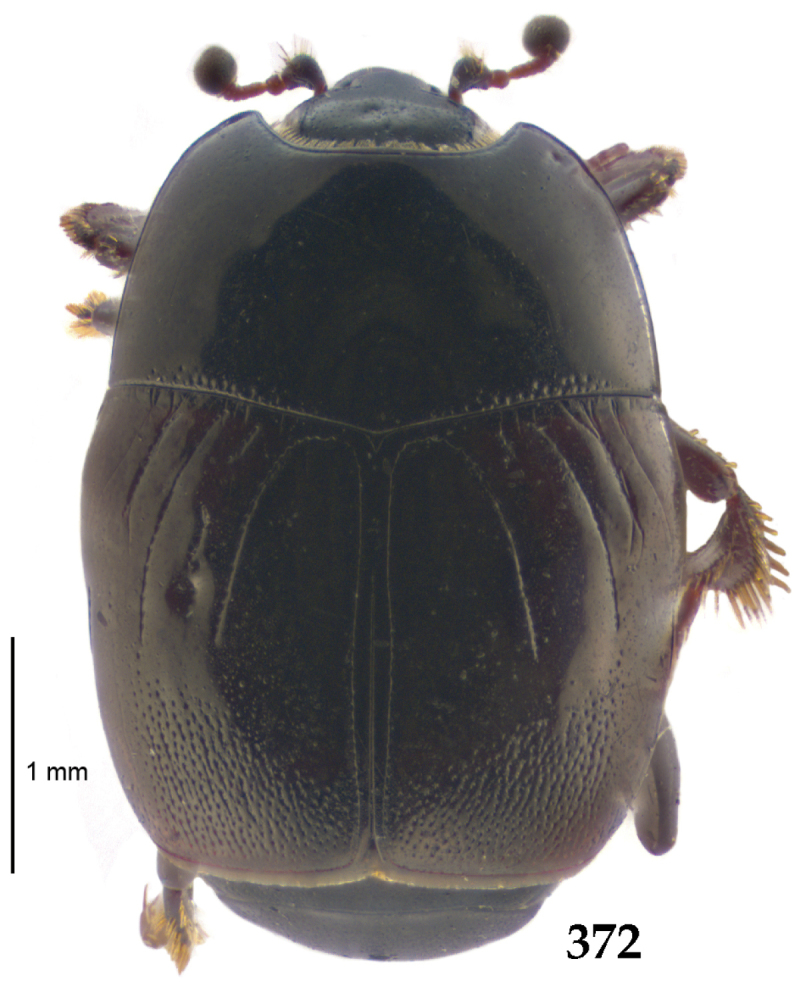
Saprinus (Saprinus) chathamensis sp. n. habitus, dorsal view.

##### Biology.

Found under rotting pilot whale and under kelp.

##### Distribution.

New Zealand: Chatham Islands (Chatham and Pitt Islands; Fig. 761).

##### Diagnosis.

*S. chathamensis* is a species morphologically, including male genitalia, very similar to other two New Zealand endemics (*S. detritus* and *S. pseudodetritus* sp. n.), with which it most likely shares a recent common ancestor. It is a dark-brown species without metallic luster with a strongly reduced third dorsal elytral stria. It differs from the other two species chiefly by almost completely impunctate pronotum (punctate in the other two species) and different male terminalia. Its eighth sternite is strongly sclerotized and narrowing apically (Fig. 382), whereas the eighth sternite of other two species is not so strongly sclerotized and wider apically. Likewise, the eighth sternite of *S. chathamensis* is apically adorned only with several short setae (instead of a tuft of setae in the other two species).

The three New Zealand species of *Saprinus* (*S. chathamensis* sp. n., *S. detritus* and *S. pseudodetritus* sp. n.) share the following character states: absence of pronotal depressions, shortened third dorsal elytral stria, setose pronotal hypomeron, interrupted frontal stria as well as strongly sclerotized eighth sternite of male terminalia (entirely fused), apically on each side with a tuft of setae, gradually dilated apical half of spiculum gastrale and separated parameres of male genitalia, with a short basal piece of aedeagus.

##### Diagnostic description.

**Figures 373–381.**
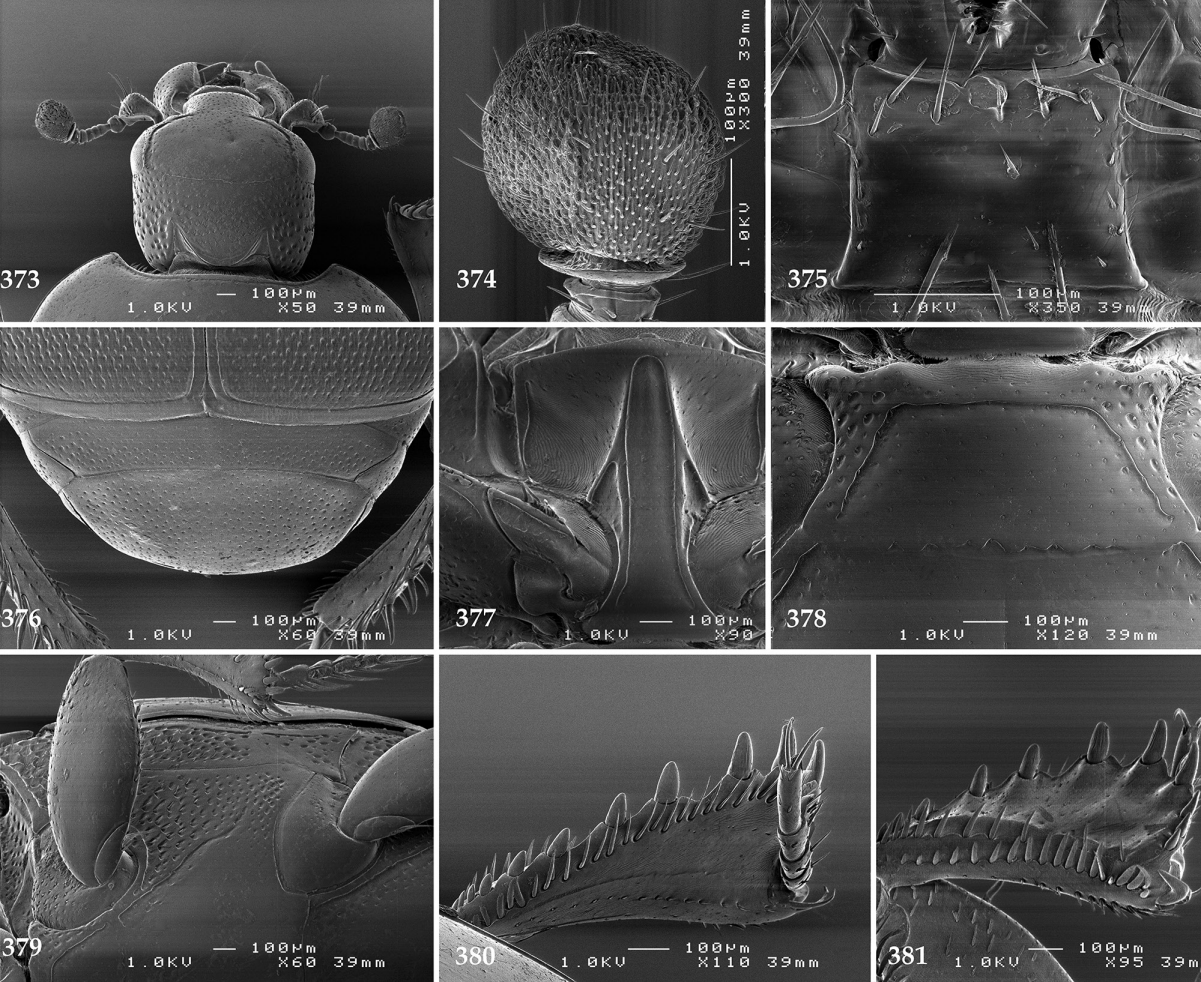
**373** Saprinus (Saprinus) chathamensis sp. n. head, dorsal view **374** antennal club, dorsal view **375** mentum, ventral view **376** propygidium + pygidium **377** prosternum **378** mesoventrite **379** lateral disc of metaventrite + metepisternum **380** protibia, dorsal view **381** ditto, ventral view.

Since *S. chathamensis* is rather similar to *S. detritus* and *S. pseudodetritus* we will provide only its diagnostic description here mostly outlining the chief differences between the three taxa. The figures, as well as male genitalia drawings are kept, for the sake of easier identification of the Australopacific taxa. The same approach is taken with the species *S. pseudodetritus*. On the other hand, the species *S. detritus*, which was described originally as the first of the three New Zealand species, will be provided with full detailed description. Dark-brown, shining species with almost black pronotum (Fig. 372), cuticle without metallic luster, body length: PEL: 2.65–3.75 mm; EL: 1.65–2.50 mm; APW: 1.50–1.60 mm; PPW: 2.00–2.85 mm; EW: 2.20–3.15 mm. Antennae (Figs 373–374) similar to those of *S. detritus* (Figs 423–424) or *S. pseudodetritus* (Figs 512–513); sensory structures of antennal club not examined. Mouthparts generally very similar between the three species, mentum (Fig. 375) also similar to those of *S. detritus* (Fig. 425) and *S. pseudodetritus* (Fig. 514), but disc of mentum finely imbricate, and almost asetose (covered with setae in the other two species). Clypeus and frons (Fig. 373) similar to other two species (compare with Figs 423 and 512) frontal stria deeply impressed, interrupted medially (occasionally narrowly). Pronotal sides (Fig. 372) moderately narrowing apically, pronotum except for a double row of sparse punctures along pronotal base glabrous (other two species have a band of punctures along lateral margins; compare Figs 422 and 511). Elytral structure as well as configuration of elytral striae generally similar between the three species (compare Figs 372, 422 and 511), with characteristically shortened third elytral stria. Elytral punctation confined to apical elytral third, elytral flanks impunctate, punctures rather deep, forming strioles, interspaces without microsculpture, punctures separated by about their own diameter (Fig. 372). Propygidium and pygidium (Fig. 376) with fine small round punctures separated by about their diameter, interspaces imbricate; similar to that of *S. pseudodetritus* (Fig. 515). Prosterna generally similar between the three species (compare Fig. 377 with Figs 426 and 516), but carinal prosternal striae rather narrowing apically, whereas they are almost parallel-sided in *S. pseudodetritus* (Fig. 516) and somewhat divergent and widening apically in *S. detritus* (Fig. 426); course of lateral prosternal striae similar between species. Mesoventrites of the three species (Figs 378, 427 and 517) trapezoidal, discal marginal mesoventral stria well impressed, carinate, complete, anteriorly straight in *S. chathamensis* (Fig. 378), while slightly inwardly arcuate in *S. detritus* (Fig. 427) and *S. pseudodetritus* (Fig. 517); disc in *S. chathamensis* with sparse fine punctation, while punctation in other two species somewhat more prominent (compare with Figs 427 and 517); meso-metaventral stria in all three species undulate. Metaventrites and lateral disc of metaventrites as well as metepisterna very similar between the three species (compare Figs 379, 428 and 518). Legs generally similar between species, for the detailed description of legs see description of *S. detritus* and compare Figs 380–381 with 429 (protibia) and 430–431 with 519–520 (meso-and metatibia).

**Figures 382–388.**
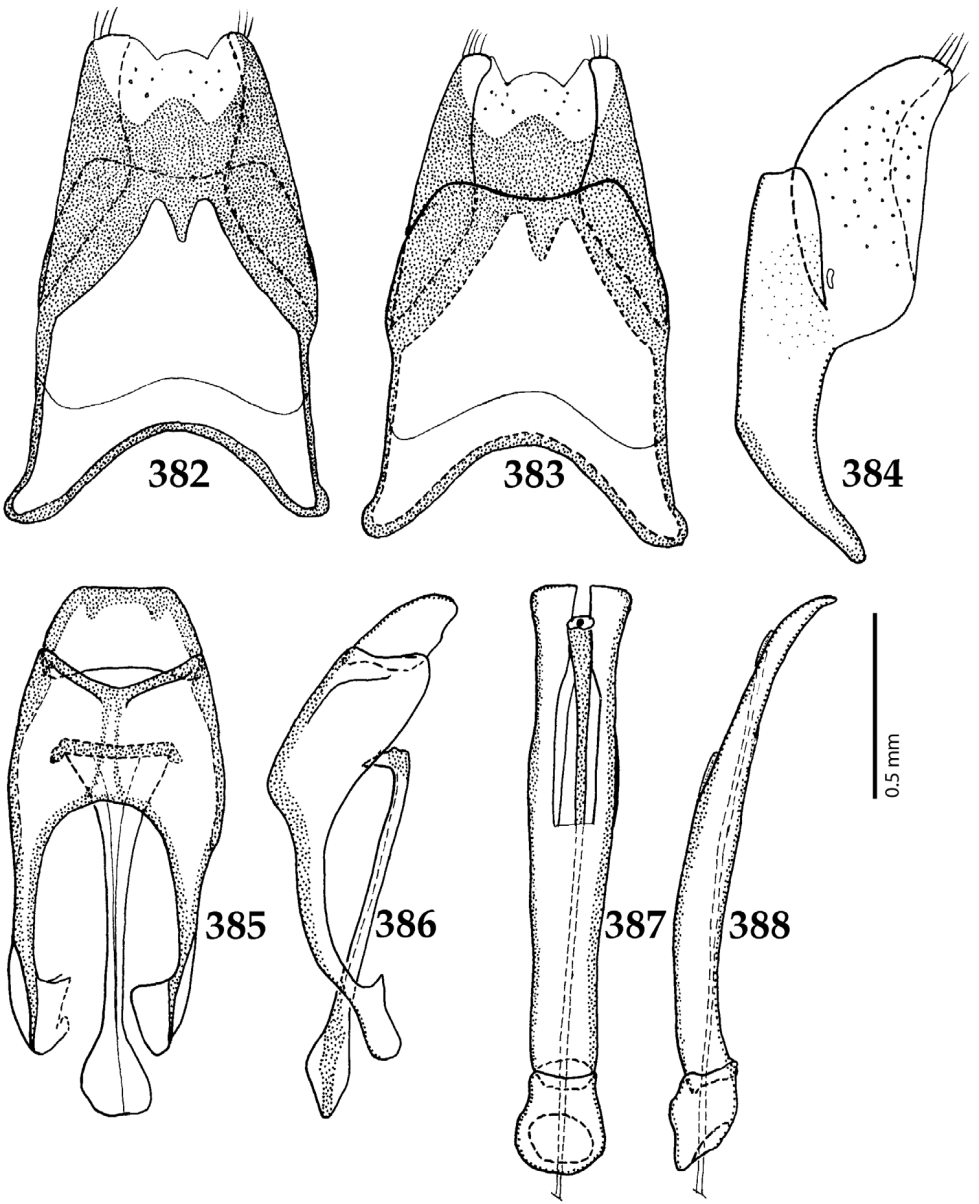
**382** Saprinus (Saprinus) chathamensis sp. n. male terminalia: 8^th^ sternite + 8^th^ tergite, ventral view **383** ditto, dorsal view **384** ditto, lateral view **385** male terminalia: 9^th^ + 10^th^ tergites, dorsal view; spiculum gastrale, ventral view **386** male terminalia: 9^th^ + 10^th^ tergites; spiculum gastrale, lateral view **387** male terminalia: aedeagus, dorsal view **388** ditto, lateral view.

Male genitalia. Eighth sternite (Figs 382–383) apically with several pseudo-pores, entirely fused, strongly sclerotized, fused sternite basally with median projection among otherwise widely separated halves; apex of eighth sternite laterally with two tufts of several moderately long setae; eighth tergite and eighth sternite fused laterally (Fig. 384). Ninth tergite (Figs 385–386) typical for the subfamily; anterior margin of tenth tergite straight; spiculum gastrale (Fig. 385) gradually dilated on apical half; apical end strongly sclerotized; basal end abruptly dilated, almost heart-shaped. Aedeagus (Figs 387–388) slightly gradually dilated from base towards apex, but before apex slightly constricted; parameres fused along their basal half (roughly); basal piece of aedeagus short, ratio of its length : length of parameres 1 : 6; aedeagus almost straight, only apically slightly curved from lateral view (Fig. 388). In general, eighth sternite and tergite of *S. chathamensis* are more strongly sclerotized and narrowing apically, while they are not so strongly sclerotized and more wide apically in the other two species (compare Figs 382–383 with Figs 432–433 and 521–522). Aedeagus of *S. chathamensis* is more similar to that of *S. pseudodetritus* than to that of *S. detritus* (compare Figs 387, 437 and 526).

#### Saprinus (Saprinus) cupreus

Taxon classificationAnimaliaColeopteraHisteridae

Erichson, 1834

Figs 389 , 390–398 , 399–407 , 753

Saprinus cupreus Erichson, 1834: 182.

##### Type locality.

Republic of South Africa: Capeland.

**Figure 389.**
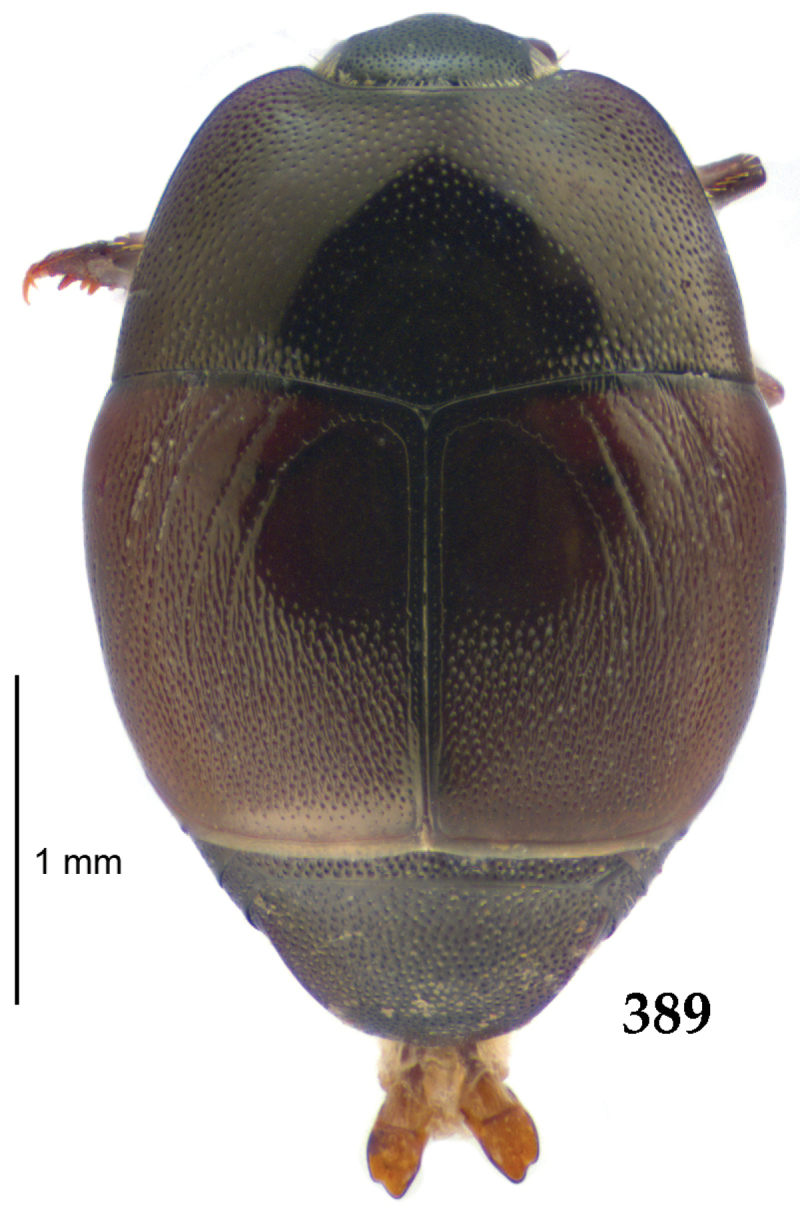
Saprinus (Saprinus) cupreus Erichson, 1834 habitus, dorsal view.

##### Type material examined.

*Saprinus cupreus* Erichson, 1834: Lectotype, present designation: specimen of unidentified sex, right hind leg missing, with the following labels: “49149” (printed); followed by: “Pr. b. sp” (light green label, written); followed by: “cupreus / Er.” (light green label, written); followed by: “Type” (brick-red label, printed); followed by: “Hist. Coll. Coleoptera / Nr. 49149 / Saprinus cupreus Er. x / Promont b. sp. Bergius / Zool. Mus. Berlin” (blue, black margined label, printed); followed by: “Saprinus cupreus / Erichson, 1834 / LECTOTYPE / des. T. Lackner 2014” (red label, written ZMHUB). Three more specimens, all of unidentified sex, paralectotypes, with hand-written label “49149”; followed by the brick-red label “Type” (added there probably by Bickhardt), and the blue ZMHUB label. The two hand-written light-green labels of the lectotype are not present with the three paralectotypes (ZMHUB). This species has been described based on unknown number of specimens and the lectotype designation fixes its taxonomic identity.

**Figures 390–398.**
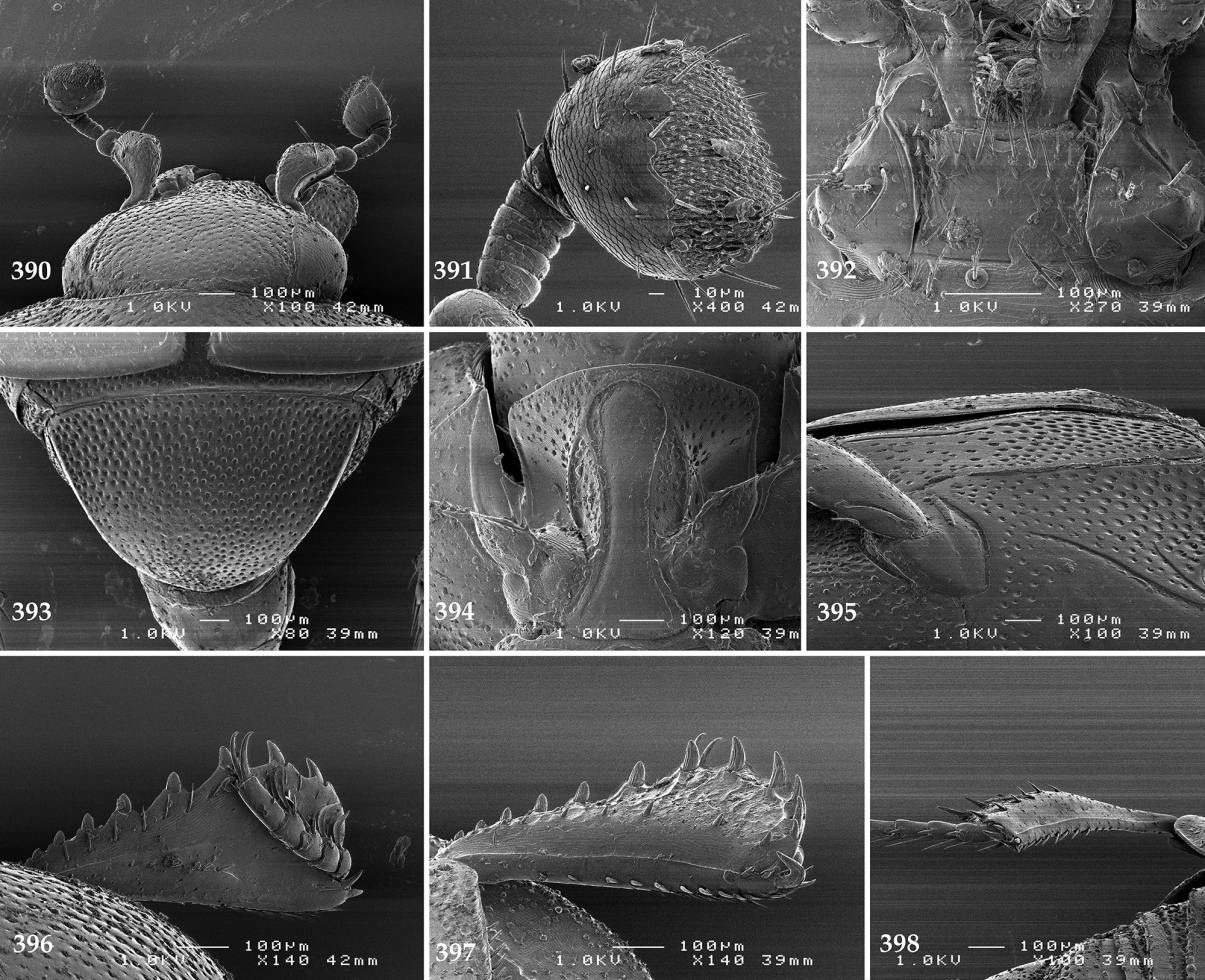
**390** Saprinus (Saprinus) cupreus Erichson, 1834 head, dorsal view **391** antennal club, dorsal view **392** mentum, ventral view **393** pygidium **394** prosternum **395** lateral disc of metaventrite + metepisternum **396** protibia, dorsal view **397** ditto, ventral view **398** metatibia, ventral view.

##### Additional material examined.

AUSTRALIA. Western Australia: 4 specs., 15 km S Busselton, 11.i.1983, G.P. Hall (ANIC); 4 specs., ditto, but 12.i.1983 (ANIC); 5 specs., ditto, but 15.i.1983 (ANIC); 5 specs., Dardanup, 19.i.1983, G. Hall (ANIC); 2 specs., Madura Station, E of Nesserman, 24.xii.1981, H. Ryan (ANIC); 1 spec., Augustus Island, CALM site 26/1, 15.25S 124.38E, 11.–16.vi.1988, I.D. Naumann (ANIC). Australian Capital Territory: 2 specs., Black Mountain, iii.1979, J.M. Walker (in fly culture) (ANIC); 1 spec., illegible locality, ii.–iii.1965, W.J.M. Vestjens (ANIC); 1 spec., O’ Connor, x.1965, K. Pullen (cow dung) (ANIC); 1 spec., no locality, 9.xii.1957, W.J.M. Vestjens (ANIC); 2 specs., Ginninderra, iii.1975, K.R. Pullen (ANIC).

New South Wales: 2 specs., 24 km N Condobolin, 7.xi.1986, W.W.K. Houston (ANIC); 1 spec., NSW, Ilford, 56 km, SSE Mudgee, 26.i.1991, on a very old dead animal (ANIC); 3 specs., NSW, Bogan River, J. Armstrong (ANIC). South Australia: 1 spec., Green Valley, Marre-B’ville, 16.ix.1972, collector unknown (ANIC); 2 specs., Adelaide, A.H. Elston (MAMU); 3 specs., Murray River, no date, A.H. Elston (MAMU); 6 specs., Halletts Cove, 26.i.1967, N. McFarland (on freshly dead sheep carcass) (SAMA); 6 specs., 15 km W of Eladuna Homestead, 7.–8.iii.1972, E.G. Matthews (SAMA); 2 specs., Karoonda to Perbinga, G.E.H. Wright (SAMA); 1 spec., Murray River, H.S. Cope (SAMA); 6 specs., near Victory Well, Everard Pk., Stn., 29.x.1970, E.G. Matthews (under bark) (SAMA); 3 specs., Edithburg, 17.iv.1908 (SAMA); 2 specs., Kimba, E. Broomhead (SAMA); 2 specs., Cooper’s Creek, J.G. Reuther (SAMA). Queensland: 3 specs., 3 km NE Mt. Webb, 15.03S 145.09E, 3.v.1981, J. Feehan (ANIC); 1 spec., McLeod R Xing, 16.29S 145.00E, c 330 m, 22.xi.1997, C. Reid (paperbark, rubbervine, sand; cow dung) (ANIC); 2 specs., Mount Mee, 7.5 km SW, 27.05S 152.42E, 12.x.1991, T. Gush (under dead snake on the road) (ANIC); 3 specs., Hay Point, 21.17S 149.15E, 28.xi.1990, T. Gush (on dead kangaroo) (ANIC); 2 specs., Charter Towers, 5.iv.1965, Bornemisza-Yapp; 1 spec., Atherton, 26.iii.1965, Bornemisza (ANIC); 6 specs., Isla Gorge National Park, NE corner, 25°10'S, 150°01'E, 4.–6.iii.1998, D.J. Cook (vine scrub, dung pitfall) (QM); 1 spec., ditto, but 15.–19.xii.1997, 240 m; 1 spec., Mt. Rose, via Taroom, Site 2, 25°26'S, 149°59'E; 30.iii.1997, 200 m, D.J. Cook (at road-killed macropod) (QM); 2 specs., Brigalow Reservation Station Site 7, 24°49'S, 149°45'E, 28.–29.iii.2001, 170 m, Monteith, Cook (paddock dung trap) (QM); 1 spec., East Woodmillar, brigalow, 25°41'S, 151°36'E, 25.–27.i.1999, 240 m, D.J. Cook (dung pitfall) (QM); 1 spec., Baldy Mt. Road, 2.4 km from S end, 1130 m, 17°20'S, 145°25'E, 30.xi.–1.xii.1997, G. Monteith (dung traps) (QM); 1 spec., Elsey NP, 12 mile camp, 14°57'S, 133°13'E, 25.–27.vi.2003, G.B. Monteith (dung trap) (QM); 1 spec., Lake Broadwater via Dalby, 22.iv.–12.vi.1986, Queensland Museum & Bennie (pitfall traps) (QM); 5 specs., Isla Gorge National Park, lookout turnoff, 240 m, 25°12'S, 149°58'E, 15.–19.xii.1997, D.J. Cook (open forest, dung trap) (QM); 1 spec., Bogantungan, 2 km N, 360 m, 23°38'S, 147°17'E; 17.–18.xii.2000, G.B. Monteith (open forest, dung pitfall) (QM); 5 specs., Expedition Range National Park, ‘Amphitheatre Campsite’, 25°12'S, 148°59'E, 25.–26.ix.1997, D.J. Cook (dung baited pitfall traps) (QM); 3 specs., BoomerRa, Site 2, 23°12'S, 149°45'E, 28.–30.ix.1999, Monteith & Cook (open forest dung trap) (QM); 1 spec., BoomerRa, Python Scrub, Site 5, 240 m 23°12'S, 149°44'E, 28.–29.ix.1999, G.B. Monteith (dung trap, vine scrub) (QM); 1 spec., L. Torquine, Kamaran Downs Stn., 24°32'S, 138°39'E, 29.–30.viii.1994, C. Eddie & G. Ford (*Acacia georginae* Shrudd) (QM); 2 specs., Moranbah, 6 km S, 25.vi.–20.xii.1997, G. Monteith & E. Kruck (Fit intercept, Box Flat) (QM); 2 specs., Jundah “Noonah-Lohern” Boundary, 24°06'S, 143°13'E, 15.iv.1993, A. Emmott (Boobok owl carcass) (QM); 4 specs., Bogantungan, 9 km N, 840 m, 23°34'S, 147°18'E, 25.–26.x.2000, D.J. Cook (dung pitfall) (QM); 2 specs., Isla Gorge Lookout, 380 m, 25°12'S, 149°58'E, 3.–5.iv.1998, G. Monteith (dung trap) (QM); 1 spec., ditto, but 22.–23.ix.1997 (QM); 1 spec., Brigalow Res. Site 3, 28.–29.x.2000, 160 m, 24°48'S, 149°46'E, D.J. Cook (dung pitfall belah/brigalow) (QM); 2 specs., MacDonald Point site 1, 22°20'S, 150°11'E, 5 m, 2.viii.2001, D. Sands & J.Haines (dung trap) (QM); 1 spec., Gregory Dev Road and Sandy Crook crossing, 300 m, 19°36'S, 145°46'E, 17.–18.xii.2006, G. Monteith (open forest faeces pitfall trap) (QM); 1 spec., Brigalow Res., Site 7, 170 m, 24°49'S, 149°45'E, 28.–29.iii.2001, Monteith & Cook (fungus trap, paddock) (QM); 2 specs., 16 km N of Springsure, 23°59'S, 148°06'E, 250 m, 4.–10.iii.2006, G. Monteith (mushroom baited pitfall brigalow regrowth) (QM); 2 specs., Wallaroo Highway, 7.5 km NW, 25°15'S, 148°38'E, 480 m, Burwell (mushroom/pitfall, Eucalypt/cycad woodland) (QM); 4 specs., Capella, 16.5km S, 210 m, 23°13'S, 148°04'E, G. Monteith (mushroom baited pitfall, open forest) (QM); 6 specs., Wallaroo Highway, 6km WNW, 25°16'S, 148°39'E, 550 m, 4.–10.iii.2006, J. Burwell (mushroom baited pitfall, open woodland) (QM); 1 spec., Auburn River National Park, 200 m, 25°43'S, 151°03'E, 24.–26.xii.2005, G. Monteith (vine scrub) (QM); 1 spec., 5th Galway, 25°34'S, 142°09'E, 22.ix.2001 (dung pitfall) (QM); 2 specs., ditto, but 29.ix.2001 (QM); 1 spec., ditto, but 2.ix.2001 (QM); 1 spec., Lords Table, SE base, site 1, 22°40'S, 148°01'E, 440 m, 4.–6.iii.2006, QM party (dung trap, eucalypt woodland) (QM); 1 spec., Barkly Highway, Six Mile Creek, 20°01'S, 137°20'E, Monteith & Cook (dung trap, gidgee gravelly soil) (QM); 1 spec., Hillview, 22°15'S, 144°16'E, 12.iv.2002, NHT Project (dung pitfall) (QM); 1 spec., Andromeda, 23°53'S, 150°11'E, 20.iii.2002, NHT Project (dung pitfall) (QM); 1 spec., Amaroo, 22°06'S, 147°20'E, 20.i.2002, NHT Project (dung pitfall) (QM); 3 specs., Lolworth National Park, 19°50'S, 146°05'E, 270 m, 12.–14.xii.2006, D.J. Cook (faeces baited pitfall, open forest) (QM); 1 spec., Toomba, 390 m, 19°58'S, 145°34'E, 14.–17.xii.2006, G.B. Monteith & D.J. Cook (faeces baited pitfall, rainforest) (QM); 2 specs., ditto, but 14.–16.xii.2006 (QM); 2 specs., ditto, but 14.–15.ii.2007, G.B. Monteith (open forest dung trap, basalt) (QM); 2 specs., Yuleba SF, site 6, 270 m, 26°56'S, 149°44'E, 9.–10.iii.2002, Monteith & Cook (dung trap, cypress & gums) (QM); 2 specs., Wongi Ponds, S turnoff, 25°26'S, 152°38'E, 20 m, 18.–21.xii.2007, D.J. Cook (heath & eucalypt) (QM); 8 specs., Southwood NP, site 4, 255 m, 27°49'S, 150°05'E, 8.–10.xii.2005, G. Monteith (black soil, mushroom baited pitfall) (QM); 4 specs., ditto, but 8.–11.xii.2005 (mushroom baited pitfall) (QM); 1 spec., Moonie, 8km SW, 270 m, 27°45'S, 150°18'E, G. Monteith (mushroom baited pitfall, black soil, brigalow) (QM); 7 specs., Coonardoo, Fletcher nr. Stanthorpe, 14.xii.1962, E. Sutton (under dead snake) (QM); 2 specs., Boggomoss, via. Taroom, 25°27'S, 150°02'E, 12.–13.xi.1996, G.B. Monteith (dung trap) (QM); 4 specs., ditto, but 14.–15.xi.1996 (QM); 21 specs., Boggom, via Taroom, 25°25'S, 150°01'E, 11.xi.1996–i.1997, Cook & Monteith (FIT trap) (QM); 2 specs., Wallum, 26°57'S, 147°45'E, ii.2002, NHT Project (dung pitfall) (QM); 1 spec., Ethabuka, 2007, C. Free (QM); 1 spec., “Araluen”, 24°25'S, 150°31'E, 12.iii.2002, NHT Project (dung pitfall) (QM). Northern Territory: 5 specs., Alexandria Downs Station, 8.viii.1982, J. Harmer (ANIC); 5 specs., Avon Downs Station, 19.viii.1982, J. Harmer (ANIC); 1 spec., McArthur River Survey, Caranbirini, 22.iv.1976, J. Feehan (ANIC); 1 spec. Borroloola, 17.i.1973, R.W.G. Jenkins (ANIC); 1 spec., NT, 24.06S 132.46E, Finke Gorge NP, Little Palm Creek, 13.iii.1995, T. Weir, rock pools, palms, algal growth, overhanging vegetation (ANIC); 2 specs., Barkly Rdhse, 129 km E, 20°04'S, 136°55'E, 16.–21.iv.2004, Monteith & Cook (dung trap, acacia/grevillea shrubland) (QM); 1 spec., ditto, but 17.–20.iv.2004, 12 km NE, 19°37'S, 135°54'E (dung trap, gidgee, red soil) (QM); 1 spec., Wunarah, near turnoff, 19°59'S, 136°38'E, 270m, 8.–11.x.2007, D.J. Cook (QM). Victoria: 2 specs., Sea Lake, no date, J.C. Goudie (MAMU).

##### Biology.

Found on carcasses as well as in dung; often collected in pitfall traps.

##### Distribution.

This species is known from tropical Africa, including Madagascar, the Cape Verde Archipelago and the British territory of Saint Helene, across the Arabian Peninsula eastward to India, Myanmar and Vietnam (Mazur 2011). It is introduced and widespread in Australia, absent from Tasmania (Fig. 753).

**Figures 399–407.**
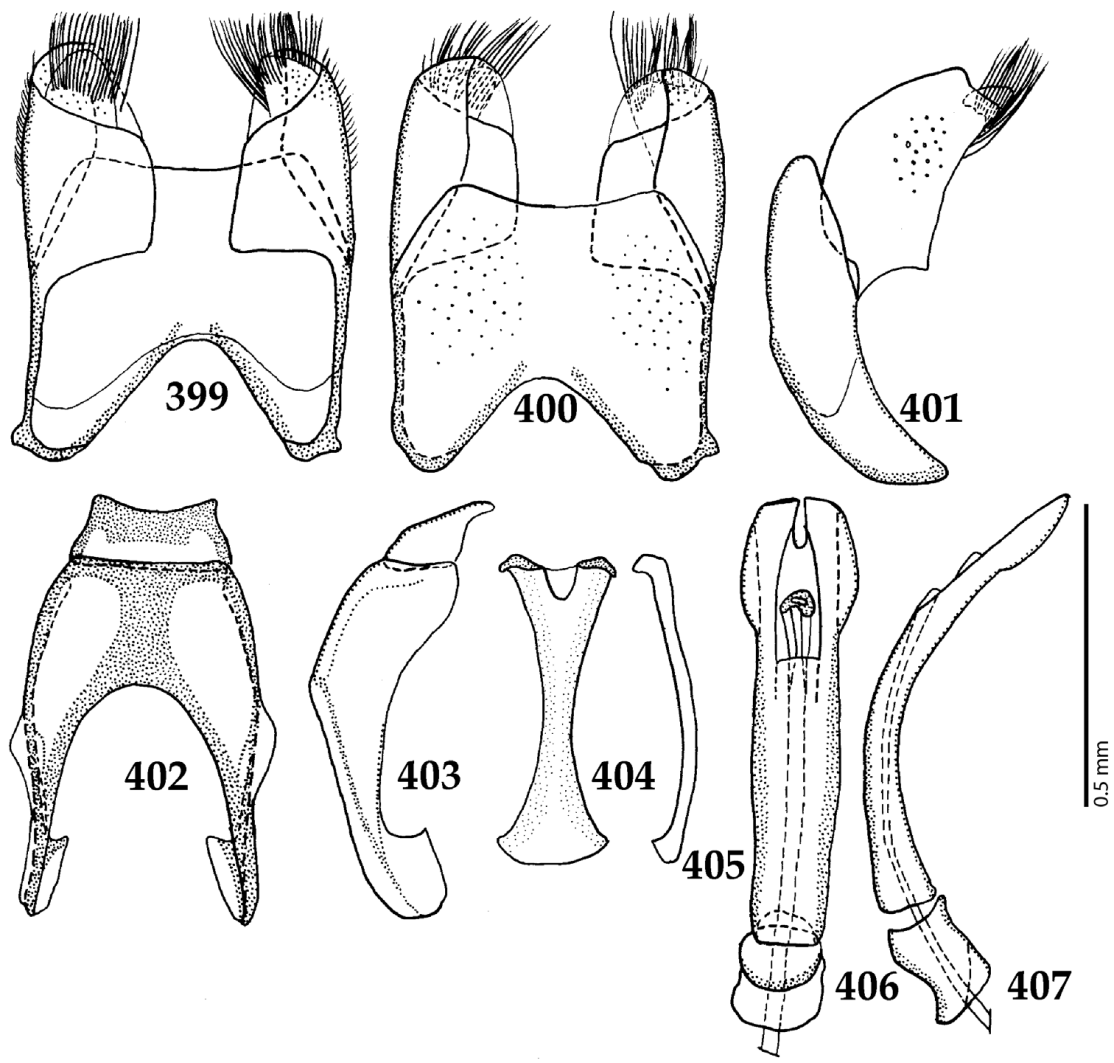
**399** Saprinus (Saprinus) cupreus Erichson, 1834 male terminalia: 8^th^ sternite + 8^th^ tergite, ventral view **400** ditto, dorsal view **401** ditto, lateral view **402** male terminalia: 9^th^ + 10^th^ tergites, dorsal view **403** ditto, lateral view **404** male terminalia: spiculum gastrale, ventral view **405** ditto, lateral view **406** male terminalia: aedeagus, dorsal view **407** ditto, lateral view.

##### Remarks.

A sexually dimorphic species, males differ from females by the presence of two prominent tubercles situated near metaventral base as well as by finer punctation of both dorsum and venter. This species is similar to *S. chalcites* and can be confused with it, differing from the latter by much more coarse and dense punctation of dorsum as well as more dilated apex of aedeagus (compare Figs 370 and 406). *S. cupreus* has been recently re-described in detail (Lackner and Gomy 2016). It can be distinguished from the rest of the Australopacific *Saprinus* species using the above key. For the sake of easier identification of the Australopacific taxa we chose to include here the photographs of its habitus, as well as SEM micrographs and male genitalia drawings.

#### Saprinus (Saprinus) cyaneuscyaneus

Taxon classificationAnimaliaColeopteraHisteridae

(Fabricius, 1775)

Figs 408 , 409–414 , 415–421 , 755 , 760 , 762

Hister cyaneus Fabricius, 1775: 52.
Saprinus australasiae Blackburn, 1903: 107 – Synonymized by Wenzel (1955): 609.

##### Type locality.

Australia.

**Figure 408.**
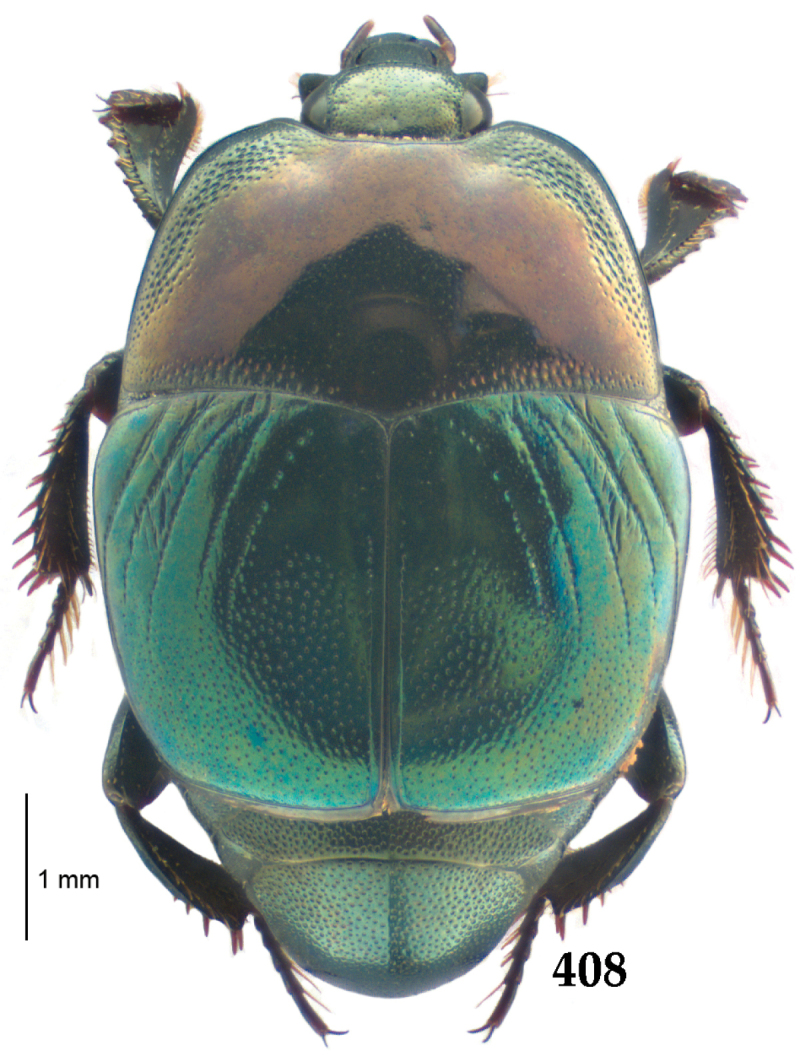
Saprinus (Saprinus) cyaneus cyaneus (Fabricius, 1775) habitus, dorsal view.

##### Type material examined.

*Hister cyaneus* Fabricius, 1775: lectotype, present designation, sex undetermined, pinned, right hind leg, left metatarsus, right mesotarsus, two last mesotarsomeres of left mesotibia missing, with written label “cyaneus”; followed by: “Hister cyaneus / Fabricius, 1775 / LECTOTYPE / des. T. Lackner 2016” (red label, written) (ZMUC). This species was described from an unknown number of specimens and the lectotype designation fixes the identity of this species.

*Saprinus australasiae* Blackburn, 1903: lectotype, present designation, sex undetermined, with the following labels: “Saprinus / australasiae / cotype” (written); followed by: “15898 / Saprinus / Australasiae / Bl. / N. Territory / Cotype” (written); followed by: “SAMA Database / No. 25-029674” (printed); followed by: “Saprinus / australasiae / Blackburn, 1903 / LECTOTYPE des. T. Lackner / 2016” (red label, written) (SAMA). This species was described from an unknown number of specimens and the lectotype designation fixes the identity of this species.

##### Additional material examined.

AUSTRALIA. Victoria: 1 ♂ & 1 ♀, N. Victoria, Nieringua, 3.i.1931, C.E. Clarke (AMNZ); 1 ♀, Australia 1934, C.E. Clarke (AMNZ); 1 ♀, Victoria, Laue & Rolle (ZMHUB). Western Australia: 3 ♂♂ & 1 ♀, Marloo Station 1934, Wurarga, Gebr. Goerling S.G. (ZMHUB); 1 ♀, Denmark (?), 1938, de Boulay (AMNZ); 1 spec., 10 km S by W of Malcolm, 29.025S 121.29E, 12.xi.1977, T.A. Weir (ex fox carcass) (ANIC); 1 spec., 3 km W Armedale 30.xii.1977, L. Haylos (Howden Trap) (ANIC); 1 spec., Red Bluff, 27.44S 114.11E, 23.xi.1971, N. McFarland (ANIC); 1 spec., Wilga, 8.xi.1973, K. Carnaby (ANIC); 1 spec., Blow Holes Road, Carnavon, 3.ix.1978, K. & E. Carnaby (ANIC); 14 specs., Carnavon, viii.1980, K. & E. Carnaby (ANIC); 1 spec., Coral Bay, Turn Off, 25.vii.1980, K. & E. Carnaby (ANIC); 1 spec., North West Cape, 21.vii.1981, K. & E. Carnaby (ANIC); 1 spec., Maidu Vale, 28.viii.1959, B.P. Moore (ANIC); 5 specs., Stirling Range Natural Park, Bluff Knoll Road, 31.xii.1985, C. Reid (on dead kangaroo) (ANIC); 1 spec., Claremont, 25.ii.1955, M.M.H. Wallace (ANIC); 1 spec., ditto, but 20.ii.1955 (ANIC); 1 spec., Nullabor, 1.iv.1967, T.O. Wolve (ANIC); 1 spec., Applecross, 24.ii.1959, F.H. Utner Baker (ANIC); 1 spec., Blow Holes Road, Carnavon, 3.ix.1978, K. & E. Carnaby (ANIC); 1 spec., Exmouth, 13.viii.1975, K. & E. Carnaby (ANIC); 1 ♀, 90 m Tank, E Lake King, 28.i.1985, K. & E. Carnaby (ANIC); 1 ♀, 43 km W of SA/WA border, 13.i.1992, Joe Bugeja (ANIC); 1 spec., Kimberley, Drysdale River National Park, Solea Falls, 14°41'S, 127°00'E, 4.–5.v.1998, D.J. Cook (pitfall, fish bait) (QM); 11 specs., Stirling Ranges, Bluff Knoll, 150 m, 12.xi.1963, J. Sedláček (QM); 1 spec., Eucla, 5 m, 28.x.1963, J. Sedláček (QM); 9 specs., Murchison River, 21.xi.1963, J. Sedláček (QM); 2 specs., 16 km N Northampton, 21.xi.1963, J. Sedláček (QM); 1 spec., Broome, 24.xi.1963, J. Sedláček (QM); 1 spec., Mt. Barker, Helms; Fitzroy & Margaret Rivers, Calvert Expedition, 1896 (SAMA); 1 spec., Beverly, F.H. du Boulay; Faure Island, Shark Bay, 15 km SE Monkey Mia, 25°53'50"S, 113°54'27"E, 27.–31.v.2000, J.A. Forrest (dung trap) (SAMA); 1 spec. Geraldton & Mullewa, Lea (SAMA). Australian Capital Territory: 1 spec., Canberra, 14.x.1965, E. Britton (coast road under dead wombat) (ANIC); 1 spec., Turner, x.1965, K. Pullen (ANIC); 1 spec., Acton, 17.xi.1967, K. Pullen (ANIC); 7 specs., Canberra, 9.xii.1957, W.J.M. Vestjens (ANIC); 1 spec., Monash, 35.24S 149.06E, 26.x.1992, W. Dressler (grass clippings) (ANIC); 1 spec., ditto, but xii.1987 (ANIC); 1 spec., ditto, but 8.ii.1987 (ANIC); 1 spec., ditto, but 25.ii.1990 (ANIC); 2 specs., Gunguhlin, 5.xii.1960, W.J.M. Vesjtens (at light) (ANIC); 3 specs., ditto, but 2.iii.1965 (ANIC); 3 specs., ditto, but 28.xi.1967, A. d’Andria (carrion) (ANIC); 1 ♀, Downer, 7.xii.1987, A. Polak (ANIC); 1 ♀, Black Mtn. Canberra, 2.iii.1982, R. Dallwitz, feeding on *Lucilla puparia* (ANIC); 1 ♀, Mt. Mujura, 10.xii.1993, A. Polak (ANIC).

Northern Territory: 3 specs., Litchfield NP, 40 km E of Daly River, 14.xii.2008, Sváťa Bílý leg. (NMPC); 2 specs., c. 85 km NW of Yuendumu, 22.15S 131.48E, 29.vi.1970, S. Parker (ANIC); 2 specs., Nourlangie Creek, 8 km N of Mt. Cahill, 12.48S 132.42E, 16.vi.1973, Upton & Feehan (ANIC); 1 spec., Nangalala, i.1973, H. Reeve (ANIC); 1 spec., Barkly Rdhse, 1.8 km SE, 19°43'S, 135°50'E, 230 m, 8.–10.ii.2007, D.J. Cook (dung pitfall trap) (QM); 1 spec., Caranbirini, Amphitheatre, 16°17'S, 136°05'E, 18.–19.iv.2004, Monteith & Cook (dung trap, *Euc. miniata*, sandstone) (QM); 1 spec., Roper River, N.B. Tindale (SAMA); 1 spec., Mary River, Woods (SAMA); 7 specs., Groote Eylandt, N.B. Tindale (SAMA); 3 specs., Darwin, G.F. Hill; (SAMA); 1 ♂ & 2 ♀♀, 15 km S Katherine, 14.vi.2000, dead wallaby, M. Hielkema (HMNH); 1 ♂, 40 km N Mataranka, 15.vi.2000, wild boar, M. Hielkema leg. (HMNH); 1 ♂, ditto, but 40 km N Mataranka, dead wallaby (HMNH). Queensland: 1 ♂, Cape York, F. Schneider (ZMHUB); 1 ♂, Endeavour River, H. Rolle, Berlin SW 11 (ZMHUB); 1 ♂, Cape Bedford (ZMHUB); 5 ♂♂ & 6 specs., N. Queensland, Yorkey’s Knob, 12 km S Cairns, 6.ix.1983, Bornemissza leg. (HMNH); 1 ♂ & 1 spec., Wallaroo, 17.i.1968, G. Hangay leg. (HMNH); 1 ♂, Edungalba, 8.i.1968, G. Hangay leg. (HMNH); 1 spec., Mackenzie River, 29.i.1968, G. Hangay leg. (HMNH); 2 specs., Yungaburra, 23.iii.1965, G.F. Bornemissza leg. (HMNH); 1 spec., Mt. Cootha (ANIC). 1 spec., upper Jardine River, Cape York peninsula, 11°17'S, 142°35'E, 20.x.1979, M.S. & B.J. Moulda (MAMU); 1 spec., Longreach, A.M. Lea (SAMA); 1 spec., Brisbane, 9.vi.1914, E. Jarvis (SAMA); 3 specs., Bowen, A. Simson (SAMA); 2 specs., BoomerRa, Python Scrub, Site 5, 240 m, 23°12'S, 149°44'E. 28.–29.ix.1999, G.B. Monteith (dung trap, vine scrub) (QM); 1 spec., Boggom, via Taroom, 25°27'S, 150°02'E, 12.xi.1996–i.1997, Cook & Monteith (FIT trap, baited) (QM); 17 specs., Boggom, via Taroom, 25°25'S, 150°01'E, 12.xi.1996–i.1997, Cook & Monteith (FIT trap, baited) (QM); 2 specs., Boggom, via Taroom, 25°29'S, 150°08'E, 14.xi.1996–i.1997, Cook & Monteith (FIT trap, baited) (QM); 8 specs., Boggom, 12/1, (NathanG) via Taroom, 25°27'S, 150°08'E, 13.xi.1996–i.1997, Cook & Monteith (FIT trap, baited) (QM); 6 specs., Boggom, via Taroom, 25°27'S, 150°03'E, 11.xi.1996–i.1997, Cook & Monteith (FIT trap, baited) (QM); 15 specs., ABRS Area 5, 142°50'E 11°40S, Captain Billy Creek, Cape York Peninsula, 9.–13.vii.1975, G.B. Monteith (fish bait) (QM); 1 spec., Telegraph Crossing, Dulhunty River, Cape York Peninsula, 2.–4.vii.1975, G.B. Monteith (QM); 1 spec., Captain Billy Beach, 142°50'E 11°40S, 9.–13.vii.1975, G.B. Monteith (QM); 2 specs., Road Crossing, Jardine River, Cape York Peninsula, 16.–27.ix.1974, G.B. Monteith (QM); 5 specs., Stanthorpe, 20.x.1926, E. Sutton (QM); 1 spec., ditto, but 17.ii.1892, collector unknown (QM); 1 spec., ditto, but 11.x.1926 (QM); 3 specs., Cairns District, J.A. Anderson (QM); 1 spec., Cairns; 4 specs., Wyreema, no date, O.W. Tiegs (QM); 1 spec., Beenlugh, 18.x.1929, collector unknown (QM); 5 specs., Nelly Bay, Magnetic Is., 5.v.1997, S. Feam (under dead cane toad) (QM); 1 spec., ditto, but v.1997, S. Feam (black light) (QM); 5 specs., Toomba, 390 m, 19°58'S, 145°34'E, 14.–17.xii.2006, G.B. Monteith & D.J. Cook (feces baited pitfall, rainforest) (QM); 5 specs., Mt. Gavial, 1 km S, 400 m, 23°36'S, 150°29'E, 17.xii.1998–14.iii.1999, D.J. Cook (open forest intercept trap) (QM); 3 specs., Toomba, 390 m, 19°58'S, 145°34'E, 14.–17.xii.2006, G.B. Monteith & D.J. Cook (feces baited pitfall, rainforest) (QM); 6 specs., Red Falls Basalt, 340 m, 19°55.6'S, 145°44'E, 16.–17.xii.2006, D.J. Cook (feces-baited pitfall trap, vinescrub, basalt) (QM); 2 specs., Gregory x Herveys Range, Dev. Road, 350 m, 19°31.7'S, 145°44.3'E, 17.–18.xii.2006, D.J. Cook (open forest, faeces pitfall trap) (QM); 3 specs., Gregory Dev. Road, 14 km NW Clarke River, 400 m, 19°07.9'S, 145°20.2'E, 17.–18.xii.2006, Monteith & Cook (faeces-baited pitfall, vinescrub) (QM); 1 spec., ditto, but 394 m, 17.xii.2006–15.ii.2007 (pitfall vinescurb/limestone) (QM); 3 specs., Lolworth National Park, 19°49.7'S, 146°05.4'E, 270 m, 12.–14.xii.2006, D.J. Cook, G. Monteith & S. Wright (hand collection, open forest) (QM); 2 specs., Mt. Robert, 2 km NNW, 21°21'S, 148°29'E, 360 m, 23.x.–18.xii. 2000, Cook & Monteith (softwood scrub, FIT) (QM); 1 ♀, Pearlinga, nr. Munduberra, 160 m, 25°36'S, 151°07'E, 20.x.2000–23.iii.2001, Cook & Monteith (vine scrub, FIT) (QM); 2 ♀♀ & 3 ♂♂, St. Lawrence, 5 km E, 22°21'S, 149°34'E, 40 m, 21.x.–19.x.2000, Cook & Monteith (softwood scrub, FIT) (QM); 2 specs., Lake Bindegolly, near bridge, 28°06'S, 144°13'E, 26.–27.viii.2000, D.J. Cook (dung pitfall) (QM); 3 specs., Isla Gorge National Park, NE corner, 25°10'S, 150°01'E, 4.–6.iii.1998, D.J. Cook (vine scrub, dung pitfall) (QM); 1 spec., BoomerRa, Site 2, 180 m, 23°12'S, 149°45'E, 28.–30.xii.1999, Monteith & Cook (open forest dung trap) (QM); 1 spec., Mt. Rose, via Taroom, 25°25'S, 149°58'E, 23.ix.–15.xii.1997, 260 m, D.J. Cook (vine scrub remnant, pitfall) (QM); 4 specs., Deepwater National Park, 65 km NW Bundaberg, 20.–26.ix.1992, G.B. & S.R. Monteith (QM); 2 specs., Moreton Island, Tertiary Dune nr. Blue Lagoon, 4.–11.xii.1988, S. Hamlet (FIT) (QM); 1 spec., Mt. Cleveland Summit, 25 km E Townsville, 13.i.1991, A. Graham (hand collecting) (QM); 2 specs., Russel River at Bellenden, Ker Landing, 5 m, 24.x.–9.xi.1981, Earthwatch/QM (baited window trap) (QM); 2 specs., Pine Mt., 1.7 km S, 21°46'S, 148°50'E, 240 m, 17.–18.xii.1999, D. Cook (vine scrub & *Eucalyptus*; dung trap); 1 spec., “Washpol Trap”, 26°22'S, 151°18'E, 28.xii.2001, NHT project (dung pitfall) (QM); 1 spec., Hartley’s Creek, 2.5km S, 16°40'S, 145°34'E, 10 m, 3.–11.ii.1999, Monteith & Cook (open forest, fish pitfall) (QM); 1 spec., Rochford’s Scrub, site 1, 20°07'S, 146°37'E, 1270 m, 11.xii.2006, G.B. Monteith (open forest, hand collection at faeces) (QM); 1 spec., McAfee’s Lookout, 300 m, 27°26'S, 152°53'E, 18.x.1999, G. Monteith (open forest, under dead goanna) (QM); 1 spec., Wongabel S.F., 5 km S Atherton, 800 m, 5.–14.xii.1988, Monteith & Thompson (FIT) (QM); 3 specs., Wallaroo Highway, 7.5 km NW, 25°15'S, 148°38'E, 480 m, Burwell (mushroom/pitfall, Eucalypt/cycad woodland) (QM); 1 spec., Jundah “Noonah-Lohern” Boundary, 24°06'S, 143°13'E, 15.iv.1993, A. Emmott (Boobok owl carcass) (QM); 1 spec., Jundah, “Noonbah Station”, 24°07'S, 143°11'E, 14.iv.1993, A. Emmot (ex cow dung) (QM); 1 spec., Dubbo Zoo, 32°15'S, 148°37'E, 5.–6.i.2000, K. Neale (wombat dung pitfall trap) (QM); 1 spec., Stony Creek, Blue Mountains, 260 m, 21°38'S, 148°59'E, 4.–5.x.1999, D.J. Cook (dung pitfall) (QM); 1 spec., Caloundra, 29.x.1913, H. Hacker (QM); 1 spec., Iron Range, 12.viii.1978, G. Czekura (QM); 5 specs., 39 miles, North Morven, 2.v.1963, E.C. Dahms (QM); 1 spec., Keysland, 26°12'S, 151°44'E, 26.i.–20.iv.1995, G.B. Monteith (pitfall trap, open forest) (QM); 1 spec., Blackdown Tableland, via Dingo, 1.–6.ii.1981, G.B. Monteith (QM); 1 spec., Bangalee Beach, 23°04'S, 150°46'E, 10 m, 16.–19.xii.1999, D.&I. Cook (dung pitfall) (QM); 1 spec., Wunarah, near turnoff, 19°59'S, 136°38'E, 270 m, 8.–11.ii.2007, D.J. Cook (QM); 1 spec., Mount Isa, 7 km east, 20°43.2'S, 139°33.8'E, 7.–11.ii.2001, 420 m, D.J. Cook (dung trap) (QM); 1 spec., Expedition Range National Park, ‘Amphitheatre scrub’, 25°13'S, 148°59'E, 17.xii.1997–5.iii.1998, Monteith & Cook (vine, intercept) (QM); 1 spec., Taroom, 6 km N on highway, 25°36'S, 149°46'E, 21.ix.–17.xii.1997, 200 m, G. Monteith (brigalow, intercept trap) (QM); 1 spec., Expedition Range National Park, ‘Amphitheatre Campsite’, 25°12'S, 148°59'E, 17.–19.xii.1997, Monteith & Cook (open forest, pitfall trap) (QM); 1 spec., Miles, 11km E, 26°40'S, 150°18'E, 360 m, 3.–6.iii.1998, D.J. Cook (open forest) (QM); 2 specs., 3 km NE of Mt Webb, 15.03S 145.09E, 1.–3.x.1980, T. Weir (open forest, human dung trap) (ANIC); 4 specs., ditto, but 30.iv.–3.v.1981, J. Feehan (ANIC); 1 spec., Iron Range, 24.xi.1985, D.J. Ferguson (ANIC); 3 specs., Anne Cay, 17.25S 151.53E, 20.iv.1994, S. Donaldson (ANIC); 3 specs., Great Barrier Reef, Frigate Cay, 21.44S 152.26E, 9.vii.1980, H. Heathwole (ANIC); 1 spec., Willis Island, 16.18S 149.58E, 25.ii.1978, collector unknown (ANIC); 3 specs., Dandabah, 6 km SE, 26°56'S, 151°37'E, 20.x.1991, Tom Gush (on dead kangaroo) (ANIC); 8 specs., Trebonne, 10 km W, 18°37'S, 146°00'E, 1.xii.1990, Tom Gush (under dead kangaroo) (ANIC); 27 specs., Hay Point, 4 km SW, 21°17'S, 149°15'E, 28.xi.1990, Tom Gush (on dead kangaroo) (ANIC); 13 specs., Great Barrier Reef, Swain Reefs, Bell Cay, i.1985, 21.49S 151.15E, H. Heatwole (ANIC); 3 specs., ditto, but 3.vii.1981, H. Heatwole & G. Burns (ANIC); 1 spec., ditto, but i.1984 (ANIC); 2 specs., ditto, but Bylund Cay, 21.47S 152.24E, vii.1984 (under dead bird) (ANIC); ditto, but Gillet Cay, i.1985; 21.44S 152.24E (ANIC); 1 spec., Mount Mee, 7.5 km SW, 27°05'S, 152°42'E, 12.x.1991, T. Gush (under dead snake on the road) (ANIC); 1 spec., 1 mile S of Herberton, 17.24S 145.23E, 14.v.1969, Brooks & Nebois (on dead wallaby) (ANIC); 2 specs., Broken Hill, 17.x.1946, R.A. Cederblad (ANIC); 1 spec., Springsure, 2.xii.1930, Mackerras (ANIC); 1 spec., Bucasia, 30.xi.1993, J. Sandery (ANIC); 4 specs., Leyburn, 22.iii.1975, J. Macqueen (ANIC); 1 spec., Rollingstone, 4.vii.1972, B.P. Moore (ANIC); 1 spec., Carnavon National Park, West Branch camp site, 8.–21.iv.1999, M. Neave, E.D. Edwards, M. Yee & J. Cardale (ANIC); 1 ♂, Gannet Cay, Great Barrier Reef, 3.xi.1967, H. Heatvole (ANIC); 1 spec., Mt. Webb, Cooktown, 10.v.1986, E. Holm (ex Howden trap, human & pig dung) (ANIC); 1 spec., 14 km W by N of Hope Vale Mission, 15.16S 144.59E, 8.–10.x.1980, T. Weir (human dung trap, open forest) (ANIC); 1 ♀, Mt. Spec., 11.i.1969, J.G. Brooks, fish lure (ANIC). New South Wales: 1 spec., without further data (HMNH); 1 spec., Coricudgy, 20.ix.2006, G. Hangay leg. (HMNH); 2 specs., New England, Glenrock Station, 17.xii.2001, G. Hangay leg. (HMNH); 1 ♂, Macquarie Marshes, 50 km SE Carinda, 28.–29.x.1985, G. Hangay leg. (HMNH); 1 spec., env. Port Macquarie, 5.iii.1965, Exp. Dr. J. Balogh (HMNH); 1 spec., Salt Hole creek, 38 km NE of Broken Hill, 27.ix.1975, Z. Liepa (ANIC); 1 spec., Murwillumbah, 7.iii.1965, Bornemissza (ANIC); 23 specs., Lightgow, 8 km SW, 33°31'S, 150°05'E, 17.xi.1991, Tom Gush (on dead kangaroo) (ANIC); 7 specs., Tenterfield, 37 km N, 28°45'S, 152°04'E, 25.xi.1990, Tom Gush (under dead kangaroo) (ANIC); 1 spec., Morisset, 3 km SW, 33°08'S, 151°27'E, 1.ix.1990, Tom Gush (on dead cow) (ANIC); 1 spec., Luke Benunee, 25.xi.1970, B.P. Moore; 1 spec., London Foundation, Kioloa, 28.xii.1980, J. Conran; 2 specs., Mullengandra, 2 km N, 35°53'S, 147°11'E, 13.x.1990, Tom Gush (on dead kangaroo) (ANIC); 2 specs., Yathong Natural Reserve, 33.45S 145.30E, near Mt. Hope, x.–xi.1992, C. Schlesinger (pitfall trap, dune; malllee with *Triodia*) (ANIC); 4 specs., Sandy Hollow, 5 km NW, 32°18'S, 150°31'E, 7.vi.1992, Tom Gush (from dead fox) (ANIC); 2 specs., Lotar Gorge, 10.xii.1969, W.N.M. Vestjens (ANIC); 1 spec., Galindary Station, 10.ix.1970, W.J.M. Vestjens (ANIC); 4 specs., Heathland, 11.45S 142.35E, 20.–22.vi.1992, T. Weir (human dung trap, open forest) (ANIC); 4 ♂♂ & 3 ♀♀, Lots 22,73, 148, Caparra, 14.iii.1993, S. Watkins (ANIC); 1 spec., Tomakin, 35.49S 150.11E, 25.xii.1988, W. Dressler (dead shark head) (ANIC); 1 spec., Congo, 8 km SE by E of Moruya, 22.–26.iii.1982, M.S. Upton (ANIC); 2 specs., 12 km E Walcha, 22.xi.1992, S. Watkins (carrion) (ANIC); 16 specs., Clyde Mt., 30.i.1975, D.P. Carne (in carrion) (ANIC); 4 specs., Gundaroo Road, 17.xi.1971, B.P. Moore (ANIC); 1 ♂ & 3 ♀♀, Matakana NE Hillston, 19.ix.1993, S.G. Watkins (carrion) (ANIC); 1 spec., 60 km W Gunnedah, 6.iii.1980, A. Newton & M. Thayer (ex wallaby carcass) (ANIC); 1 spec., 8.5 km NE Gubbata, 33.35S 146.37W, 4.–12.i.1999, D. Driscolim (pitfalls, ungrazed roadside, no spinifex) (ANIC); 1 spec., Allyn R.F. Park, 32.08S 151.27E, 27.–31.i.1986, R.B. Haliday (carrion baited trap) (ANIC); 7 specs., Parramatta P.C., 28.x.1968, D.A. Doolan (MAMU); 3 specs., North Ryde P.C., 17.iii.1967, D.A. Doolan (MAMU); 5 specs., Mogriguy, 8.xi.1979, Goonoo S.F., D.A. Doolan Coll. (MAMU); 1 spec., Bilpin, 12.iii.1979, B.J. Day (on dead bird) (MAMU); 1 spec., Warrumbungle National Park, 25.iii.1973, R.H. Mulder (MAMU); 1 spec., Blue Mountains, Blackburn (SAMA); 1 spec., Vicinity of Jenolan Caves, J.C. Wilburd (SAMA); 3 specs., Lake Callabona, A. Zietz (SAMA); 1 spec., Sydney, Lea (SAMA); 1 spec., Goulburn (SAMA); 2 specs., Broken Hill, R.J. Burton (SAMA); 1 spec., Sydney; Girl’s Lunch Room, Museum, 6.iii.1961, B. Hubbard (SAMA). South Australia: 1 ♂, nr. Tarlee, 20.x.1988, under carrion, Mrs. Lawley leg. (HMNH); 1 spec., 9 miles S of Nullabor H. Std., 6.i.1967, M. Upron (ANIC); 2 specs., Wardang Island, Spencer Gulf, 34.30S 137.22E, 4.–5.i.1995, K. Wardaugh (cowdung baited pitfall trap) (ANIC); 1 spec., 16 km S of Port Augusta, 10 m, 26.x.1963, J. Sedláček (BPBM); 5 specs., Murray River, H.S. Cope (SAMA); 15 specs., Halletts Cove, 26.i.1967, N. McFarland (on freshly dead sheep carcass) (SAMA); 6 specs., Mt. Lofty, J.G.O. Tepper (SAMA); 3 specs., Ardossan, J.G.O. Tepper (SAMA); 1 spec., Adelaide, J.G.O. Tepper (SAMA); 1 spec., Cooper’s Creek, J.G. Reuther (SAMA); 1 spec., Adelaide (SAMA); 2 specs., Port Lincoln (SAMA); 1 spec., Flinders Island, F. Wood Jones (SAMA); 6 specs., Tappanappa, xi.1948, Mitchell & Lawson (SAMA); 1 spec., Fulham, 3.iv.1909 (SAMA). Victoria: 1 spec., Leopold (MAMU); 2 specs., Marree, 8.ix.1957, I.G. Filmer (QM); 3 specs., Murray Valley Highway, 19 km E by S of Hattah, 34.47S 142.29E, 2.xi.1988, T. Weir, J. Lawrence & M. Hansen (from dead kangaroo) (ANIC); 1 spec., Manjimup, 28.x.1979, R.M. Bohart (ANIC); 1 spec., Tavonga, 2 km W, 36°40'S, 147°08'E, 13.x.1990, Tom Gush (on dead sheep) (ANIC). Tasmania: 1 spec., St. Helena, 10 km NW, 12.xii.1981, Bornemissza leg. (HMNH); 2 specs., Hollow Tree, 9 km S of, 42°37'S, 146°56'E, 12.ii.1992, Tom Gush (from dead Tasmanian Devil) (ANIC); 23 specs., Newstead, Launceston, 20.xi.1981, S. Fearn (on Stink Lily flower) (ANIC); 3 specs., Flinders Island, Bass Strait, 1.–6.ix.1989, B.M. Doube & K.G. Wardaugh (dung baited pitfall traps) (ANIC). Unknown localities: 1 ♀, 2 ♂♂ & 5 specs., Australia, no further data (HMNH); 6 specs., Australia occid., 1192, no further data (HMNH); 1 ♀, no locality, E.C. Gourlay Acq. 1970, Ent. Div. (NZAC). 4 specs., Emerald, iii.1914, E. Allen (QM); 2 specs., Cor’s River, 4.ix.1932, Chadwick (under dead rabbit) (ANIC); 1 ♂, Summer Hill, “Kelvin”, xi.1937, C. Storyles (AMNZ); 1 ♂, Koranda, 22.vi.1938, C.E. Clarke (AMNZ). Lord Howe Island: 1 spec., 21.–30.i.1985, G.F. Bornemissza leg. (HMNH).

NEW CALEDONIA. 1 spec., Nouméa env., 4.ii.1914, P.D. Montague (BMNH); 1 spec., Mt. Mou, 23.iv.1914, P.D. Montague (BMNH); 10 specs., Isle Atire, 1.ix.1982, T. Lovegrove (ex dead white-capped noddy) (AMNZ); 3 specs., Tiea reserve, 30 m, 21°07.9'S, 164°57'E, 5.–26.xi.2001–31.i.2002, G. Monteith (FIT Trap) (QM); 5 ♂♂, 1 ♀ & 22 specs., In Mts. up Beulari River, 3.–4.xi.1958, C.R. Joyce (BPBM); 1 ♀, ditto, but 17.xi.1958, same collector (BPBM); 2 ♂♂, 1 ♀ & 15 specs., Beach nr. la Foa, 19.xi.1958, same collector (BPBM); 1 ♀ & 2 specs., Anse Vata, 23.xi.1958, same collector (BPBM); 1 spec., ditto, but 9.xi.1958, same collector (BPBM); 1 spec., ditto, but 25.x.1958, same collector (BPBM); 3 ♂♂ & 1 spec., La Crouen, 12.iii.1961, J. Sedláček (BPBM); 1 ♂ & 4 specs., Nouméa, 26.iv.1945, H.E. Milliron (BPBM); 5 specs., La Foa, 4.ii.1945, same collector (BPBM).

PAPUA NEW GUINEA. 8 specs., Laloki, CSIRO Screw Worm Lab., iv.1987, S. Bakker (FIT) (ANIC); 1 ♂, Kelesi, xi.–xii.1890, L. Loria leg. (ZMHUB); 1 ♂ & 1 ♀, Ighibrei, vii–viii.1890, L. Loria (ZMHUB).

FIJI. 1 ♀, Lautoka, 24.viii.1936, H. Phillips (BPBM) (Fijian distribution not shown on a map).

##### Biology.

*S. cyaneus cyaneus* is found chiefly on decomposing organic matter, most commonly on carcasses; some specimens were trapped in pitfall traps baited with dung, fish or mushrooms, others were collected on flowers of Stink Lily (*Dracunculus vulgaris* Schott). This is one of the most common species found on carrion in Australia and an important predator of larval circular-seamed flies (Diptera), the adults of which are vectors of various diseases (see e.g. Lackner 2010 for details).

##### Distribution.

Australia (all states), newly reported from Lord Howe Island; New Guinea, New Caledonia; new to Fiji (Figs 755, 760, 762).

##### Remarks.

Another subspecies, S. (S.) cyaneus auricollis Marseul, 1855 is found in the Philippines, Indonesia (islands of Bali and Buru, westernmost New Guinea), and Japan (Ogasawara archipelago; Mazur 2011). This subspecies differs from Saprinus (S.) cyaneus cyaneus by its lighter color. However, variation in the elytral punctation and striae and shape of carinal prosternal striae indicate that the taxonomic status of the subspecies requires further study. Elytral aciculations also vary between the sexes (see also Wenzel 1955). We designate the lectotype of *S. auricollis* here: *Saprinus auricollis* Marseul, 1855: Lectotype, present designation: ♀, pinned, left protarsus broken off, left metatarsus broken off, right mid-leg broken off, right metatarsus broken off, with the following labels: “Saprinus / 31 auricollis / I. Phillip. ♀ / illegible” (round yellow label, written); followed by an illegible written label; followed by: “MUSEUM PARIS / auricollis / COLL. / DE MARSEUL 1890” (printed-written); followed by: “TYPE” (red-printed label); followed by: “Saprinus auricollis / Marseul, 1855 / LECTOTYPE / des. T. Lackner 2014” (red label, written) (MNHN). This species was described from unknown number of specimens and the lectotype designation fixes the identity of the species.

##### Re-description.

Body length: PEL: 3.25–5.10 mm; APW: 1.25–2.00 mm; PPW: 2.50–4.10 mm; EL: 1.85–3.10 mm; EW: 2.75–4.50 mm. Body (Fig. 408) rectangular oval, convex, elytra widest at humeri, dark green to dark brown or even black, occasionally dark blue, shining, with metallic luster, pronotum purple, occasionally dark bronze with slight greenish hue, metallic; legs, mouthparts and antennal scape castaneous to dark brown; antennal club even darker, almost black.

**Figures 409–414.**
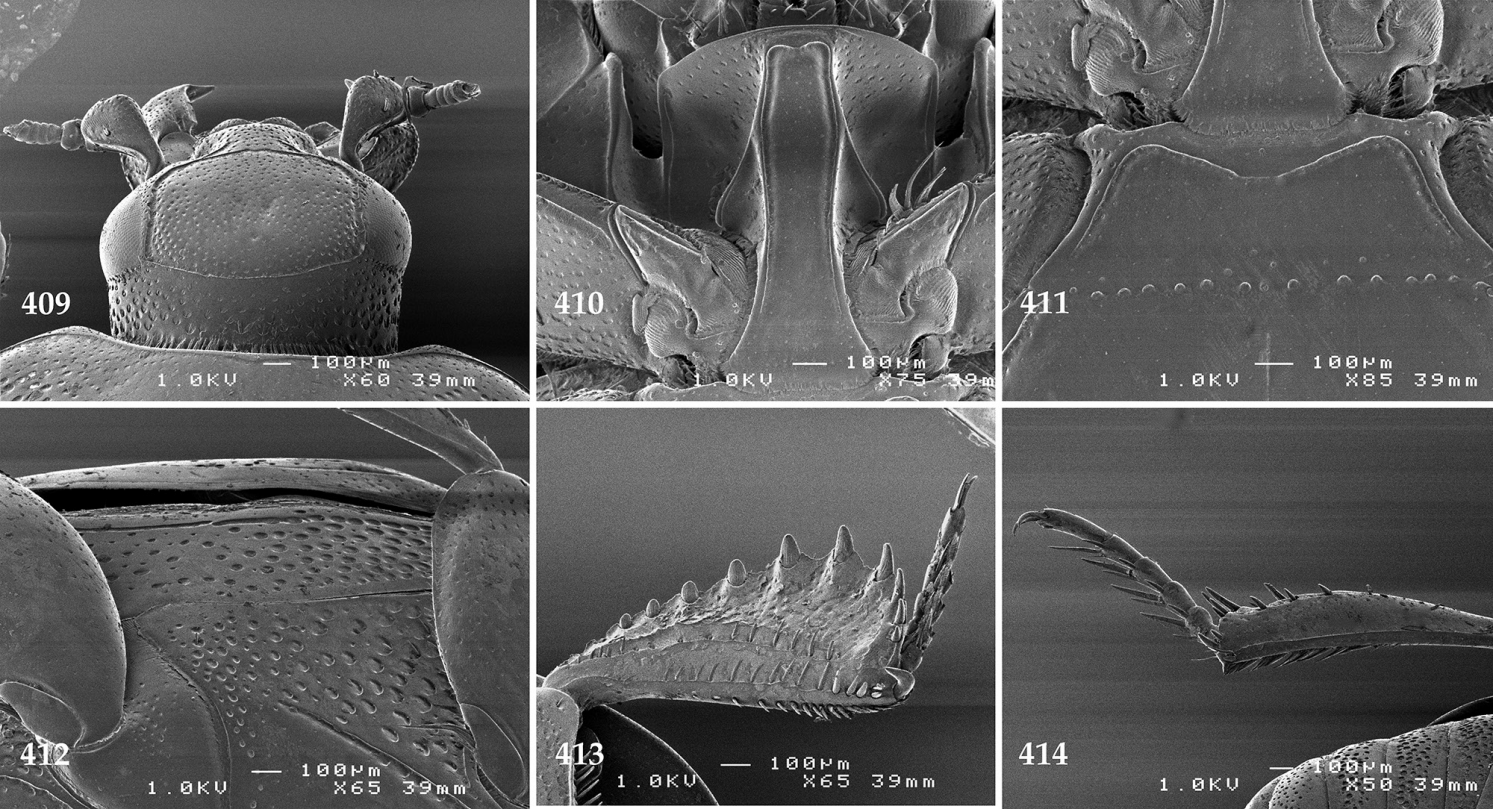
**409** Saprinus (Saprinus) cyaneus cyaneus (Fabricius, 1775) head, dorsal view **410** prosternum **411** mesoventrite **412** lateral disc of metaventrite + metepisternum **413** protibia, ventral view **414** metatibia, ventral view.

Antennal scape (Fig. 409) slightly thickened, punctuate, with two rather long and several shorter setae; antennal club wider than long, semi-circular, somewhat truncate apically, covered with dense short sensilla intermingled with sparse longer erect setae; sensory structures of antennal club in form of four large oval ventral sensory patches, which are usually difficult to discern; vesicle(s) not examined.

Mandibles (Fig. 409) dorso-laterally punctuate, rounded, outer margin slightly carinate, mandibular apex acute, sub-apical tooth on left mandible obtuse; labrum finely and sparsely punctuate, convex, with deep median depression; labral pits present, each with two short labral setae; terminal labial palpomere elongate, pointed apically, approximately twice as long as pen-ultimate, its width about three times its length; terminal maxillary palpomere elongate, about 2.5 times as long as pen-ultimate, approximately 4.5–5 times as long as wide, pointed apically; mentum sub-quadrate, laterally with a double row of short ramose setae, anterior margin medially with prominent notch surrounded by a tuft of several longer sparse setae.

Clypeus (Fig. 409) even, occasionally very faintly convex or even concave, constricted laterally, punctate; frontal and supraorbital striae complete, occasionally frontal stria weakened medially, its vestiges prolonged onto clypeus; frontal disc (Fig. 409) with similar punctation as that of clypeus, near posterior margin approximately medially a definite fovea present; eyes convex, well visible from above.

Pronotal sides (Fig. 408) narrowing anteriorly, apical angles obtuse, pronotal depressions present, rather deep, anterior incision for head moderate; marginal pronotal stria carinate, visible along its entire length from dorsal view, but ending short of pronotal base; pronotal disc laterally with a depressed band of deep dense coarse elongate punctures originating approximately in pronotal depressions, not reaching basal angles of pronotum, between it and pronotal margin a narrow finely punctate band present; rest of the pronotal disc with only scattered microscopic punctation; several rows of punctures present along pronotal base, ante-scutellar area almost impunctate; pronotal hypomeron asetose, with scattered fine punctures; scutellum small, visible.

Elytral epipleura with sparse fine punctures, occasionally almost impunctate; marginal epipleural stria complete; marginal elytral stria well impressed and carinate, continued as weakened, but complete apical elytral stria that is connected to incomplete sutural elytral stria. Humeral elytral stria well impressed on basal third, sometimes connected to median fragment of inner subhumeral stria; outer subhumeral stria present as short basal fragment situated on elytral humerus; elytra usually with four dorsal elytral striae 1–4 well impressed, striae 1–2 in fine punctures, striae 3–4 in larger punctures, occasionally fourth stria impressed only as a row of round punctures; first and second striae in most cases longer than striae 3–4, slightly surpassing elytral half apically, third and fourth dorsal elytral striae usually slightly shorter, reaching approximately elytral half apically, in first elytral interval often dense elongate strioles present, at times these strioles present also on intervals 2–3; fourth elytral stria basally curved toward shortened sutural elytral stria, but never connected with it; sutural elytral stria abbreviated on basal fourth to fifth, well-impressed, in round punctures, apically connected with apical elytral stria; elytral disc on apical half (roughly) punctate, punctures usually fine, sparse, separated by several times their diameter, occasionally aciculate (especially those near elytral intervals); almost not entering elytral intervals, except for fourth interval where they reach their climax; punctures occasionally not reaching elytral apex.

Propygidium very densely punctate, punctures separated by less than their own diameter; pygidium with sparser but larger punctation.

Anterior margin of median portion of prosternum (Fig. 410) rounded; marginal prosternal stria present laterally and also as medial fragment; prosternal process between carinal prosternal striae slightly convex, sparsely and finely punctate, surface near united apices of carinal prosternal striae distinctly depressed; carinal prosternal striae carinate, slightly bisinuate, occasionally slightly divergent on anterior third, in most cases united in front (Fig. 410); lateral prosternal striae carinate, rather short, apically attaining carinal prosternal striae at about three-fourths of their length.

Discal marginal mesoventral stria (Fig. 411) well impressed, carinate, complete, anteriorly distinctly inwardly arcuate; disc often smooth, at times with sparse fine punctation; meso-metaventral sutural stria indicated by a row of large punctures; intercoxal disc of metaventrite in males with prominent median longitudinal depression, in females slightly convex; disc of metaventrite for the most part almost smooth, only in post-metacoxal area and along basal margin of metaventrite several rows of fine punctures appear; lateral metaventral stria (Fig. 412) well impressed, carinate, straight, not reaching metacoxa; lateral disc of metaventrite (Fig. 412) slightly concave, with dense and large round punctures separated by less than their to about their own diameter, occasionally between large punctures another type of much finer sparse punctures present; metepisternum (Fig. 412) similar, but punctures deeper and denser, on fused metepimeron punctures becoming much sparser; metepisternal stria present often only along fused metepimeron, at times along metepisternum also present in a form of short intermittent fragments (in rare cases complete).

Intercoxal disc of first abdominal ventrite with faint to moderate median depression in males, in females slightly convex, completely striate laterally; disc along basal and lateral margins with shallow punctures of various sizes; rest of sternite with scattered microscopic punctation.

Protibia (Fig. 413) slightly dilated, outer margin with around four low teeth topped by triangular denticle, followed by approximately four tiny denticles diminishing in size proximally; setae of outer row regular, short; protarsal groove shallow; anterior protibial stria present on basal two-thirds, next obliterated; setae of median row shorter and much sparser than those of outer row; two tarsal denticles present near tarsal insertion; protibial spur (Fig. 413) bent, growing out from apical margin of protibia; apical margin of protibia ventrally with three tiny denticles; outer part of posterior surface slightly obscurely variolate, punctate, separated from glabrous and narrow median part of posterior surface by a ridge bearing a row of setae; posterior protibial stria complete, bearing along its length sparse row of microscopic setae turning into several minuscule denticles apically; inner row of setae single, lamellate.

Mesotibia on outer margin with a row of about seven distally growing in size denticles, several of them situated on low teeth; setae of outer row rather strongly sclerotized, sparse, but longer than denticles; setae of median row shorter and finer than those of outer row; posterior mesotibial stria not complete; mesotibial spur rather long and stout; on anterior face of mesotibia a row of about 5 widely-spaced denticles present; anterior face of mesotibia with scattered fine punctation; anterior mesotibial stria incomplete (in rare cases almost complete); inner anterior denticles weakly developed, usually only one or two present; inner row of setae rather dense. Metatibia (Fig. 414) basically similar to mesotibia, but longer and more slender, and denticles of both rows sparser.

**Figures 415–421.**
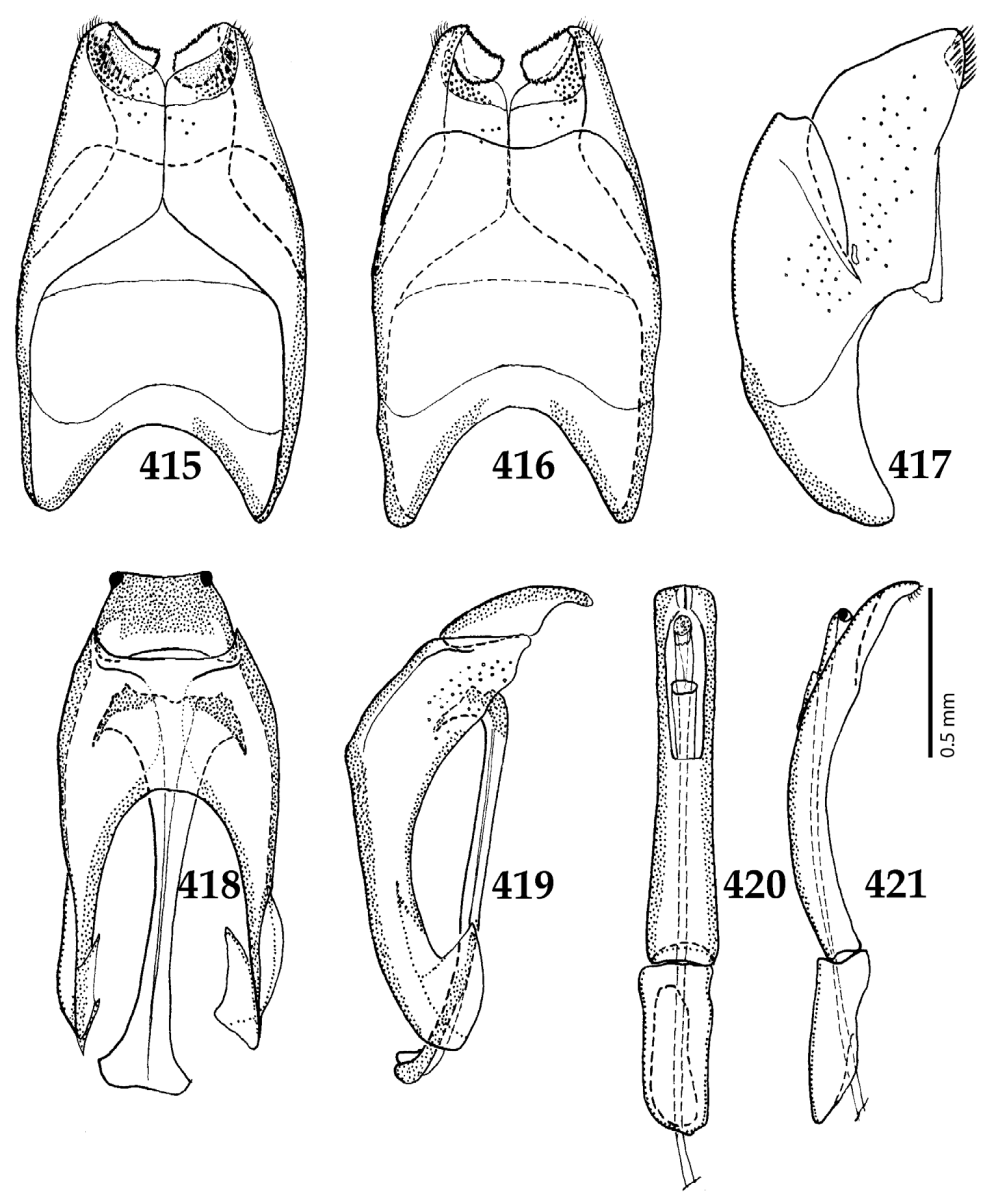
**415** Saprinus (Saprinus) cyaneus cyaneus (Fabricius, 1775) male terminalia: 8^th^ sternite + 8^th^ tergite, ventral view **416** ditto, dorsal view **417** ditto, lateral view **418** male terminalia: 9^th^ + 10^th^ tergites, dorsal view; spiculum gastrale, ventral view **419** male terminalia: 9^th^ + 10^th^ tergites; spiculum gastrale, lateral view **420** male terminalia: aedeagus, dorsal view **421** ditto, lateral view.

Male genitalia. Eighth sternite (Figs 415–416) strongly sclerotized, fused medially, with sparse pores and pseudopores, apex laterally with sparse short setae, vela present, with several rows of pores furnished with short setae; eighth tergite apically faintly inwardly arcuate, basally strongly inwardly arcuate; eighth tergite and eighth sternite fused laterally (Fig. 417). Ninth tergite (Figs 418–419) laterally strongly sclerotized, medially with a strongly sclerotized part resembling a fusion seen also in other congeners, with pores and pseudopores, well visible especially laterally, ninth tergite basally strongly inwardly arcuate, apical parts of ninth tergite with extra sclerotized parts resembling enforcements; tenth tergite basally and apically inwardly arcuate, apices very strongly sclerotized and prominent; spiculum gastrale (Fig. 418) dilated on both ends, otherwise almost parallel-sided, apical ends strongly sclerotized, basal end slightly inwardly arcuate. Aedeagus (Figs 420–421) almost parallel-sided with parameres fused along basal two-thirds, curved from lateral view; basal piece of aedeagus moderately long, ratio of its length : length of parameres approximately 1 : 3; apex of aedeagus ventrally with a tuft of microscopic setae.

#### Saprinus (Saprinus) detritus

Taxon classificationAnimaliaColeopteraHisteridae

(Fabricius, 1775)

Figs 422 , 423–431 , 432–438 , 758 , 761

Hister detritus Fabricius, 1775: 53.
Saprinus antipodus Dahlgren, 1971: 48 – Synonymized by Mazur (1984): 50.

##### Type locality.

Australia (error). This New Zealand endemic was erroneously described from Australia. A complete explanation of its taxonomic history is given in Kuschel (1987).

**Figure 422.**
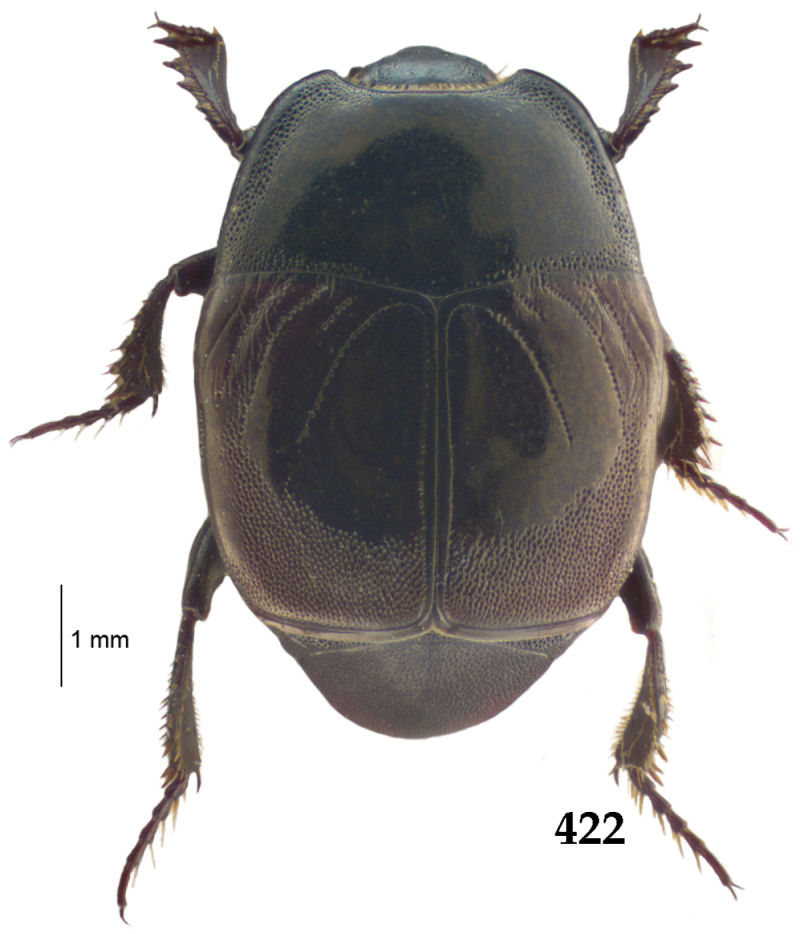
Saprinus (Saprinus) detritus (Fabricius, 1775) habitus, dorsal view.

##### Type material examined.

*Hister detritus* Fabricius, 1775: Lectotype, ♂, designated by J.C. Watt in 1985, pinned specimen with a lump of yellow glue on its venter covering both metatibiae, with the following labels: “Hister detritus / Fab. Entom. p. 53 p. 10” (black-margined, printed-written label); followed by: “LECTOTYPE ♂ / Hister / detritus F. / det. J.C.Watt / (Saprinus) 1985” (printed written); followed by a label with a QR code and “NHMUK010601207” (printed) (BMNH).

*Saprinus antipodus* Dahlgren, 1971: Holotype, ♂, originally pinned, glued to a rectangular mounting card, genitalia extracted, glued onto the same card as the specimen, with the following labels: “Neu-Seeland” (written); followed by: “pseudocyaneus / Bickhardt” (written); followed by: “MUSÉUM PARIS / 1933 / Coll. Desbordes” (printed); followed by: “Saprinus / antipodus / Dahlgren, 1971 / HOLOTYPE / det. T. Lackner ‘17” (red label, written) (MNHN). Dahlgren (1971) described the species based on a single male, describing its labels exactly. The specimen found at MNHN completely agrees with Dahlgren’s description and is thus the holotype by inference.

##### Additional material examined.

New Zealand. North Island. ND: 1 spec., Kawerua Beach, Northland, 5.ii.1975, R.A. Harrison (LUNZ); 1 spec., Puketi Forest, Waipapa R. Tk., 30.i.–3.ii.1995, J. Klimaszewski (pittrap with carrion, broadleaf forest) (NZAC). AK: 1 spec., Mangere, 2.i.1979, R.R. Scott (ex garden) (LUNZ); 8 specs., Auckland, without further data (BMNH); 1 ♂, 1 ♀ & 1 spec., Little Barrier, Mt. Hobson, H. Swale, 1913–117 (BMNH); AK: 2 specs., Whatipu Beach, Waitakere, 16.iii.1994 (under dead seagull) (NZAC); 1 spec., Lynfield, 8.x.1991, G. Kuschel (in swimming pool) (NZAC); 1 spec., Lynfield, 1.ii.1975, G. Kuschel (NZAC); 3 specs., Waitakere Ranges, Karekare, 81 Lone Kauri Rd., 220 m, 15.iv.2000, R.F. Gilbert (LUNZ); 1 spec., Upper Aorere, 24.iii.1938, A. Richardson (AMNZ); 8 specs., AK, Auckland Western Springs, 10.xi.2001, S.E. Thorpe (under dead rat) (AMNZ). CL: 2 specs., The Aldermen Is., Raumahuaiti I., 5 m, 5.–11.xii.1994, J.W. Early, R.F. Gilbert (coastal taupata/karo Hymenanthera/ngaio scrub, yellow pan trap) (LUNZ); 4 specs., Mercury Is., Green I., 23.xii.1987, G. Hall (under dead penguin) (NZAC); 3 specs., Middle I., Mercury I., i.1983, I. Southey (NZAC); 2 specs., Deep Stream, Taieri Rd., 24.x.1924, C.E. Clarke (AMNZ); 1 spec., The Aldermen Is., Hongora, 17.xi.2003, J.W. Early (under dead seabird on soil) (AMNZ). WO: 4 specs., Okoroire, 24.xii.1920, C.E. Clarke (AMNZ). BP: 1 spec., Kaituna, 23.xii.1981, A. Siddique (LUNZ). GB: 6 specs., Te Urewera NP, 22.–26.xi.2012, Lake Waikaremoana Motorcamp, 38°45.3'S, 177°09.4'E, 600 m, A. Becker, M. Fikáček & J. Hájek leg. (5 specs. in coll. NMPC; 1 spec. in coll. TLAN). TO: 1 spec., Rangipo Dam, 26.xii.1996, B.J. Gill (from corpse of hare) (LUNZ); WN: 1 spec., Wellington, x.1893, S.V.H. (AMNZ); 3 specs., Somes Island, Wellington, 18.i.1996 (in seagull’s nest) (CJN); 1 spec., Island Bay, x.1991 (in compost) (CJN); 1 spec., Karore; 27.xii.1920 (BMNH). South Island. NN: 1 spec., Nelson, 4.iii.1960, E.S. Gourlay (NZAC); ditto, but 10.ii.1959 (NZAC); ditto, but 22.iii.1960 (NZAC). SD: 1 spec., Stephens Island, Keepers Bush, 18.ii.1994, J.W.M. Marris (on tree at night) (LUNZ); 5 specs., Waikawa Beach, 2.iv.1995 (under dead seagull on beach) (CJN); 1 spec., Picton, 24.xii.1941, C.E. Clarke (AMNZ); 1 spec., North Brother Island, 9.ii.1993, J.W.M. Marris (ex petrel burrow litter) (LUNZ). MB: 2 specs, Awatere River, 25.xii.1943, C.E. Clarke (AMNZ). BR: 1 ♂ & 1 ♀, Mt. Cook Nat. Park, Murchison 1000 m, F/S 30.iii.1976, W.J. Sweney (LUNZ); 1 spec., Pahautane Bay, 4.ix.1981, R.M. Emberson (under log) (LUNZ). KA: 3 specs., Molesworth, Awatere River, 28.xii.1943, C.E. Clarke (BMNH). NC: 2 specs., Plate Is., 28.iii.1932, R.A.F. (in addled birds egg) (AMNZ). MC: 1 spec., Lincoln, 2.iii.1962 (LUNZ); 1 spec., MC, Lincoln, i.1994, N.C. Schroeder (on dead hedgehog) (LUNZ); 14 specs., Lincoln College, 22.xii.1966, C.C. Boswell (LUNZ); 1 spec., L Georgina 544 m, 23.ii.1982, S.P. Warner (swept tussock) (LUNZ); ditto, but 8.iii.1983 (swept) (LUNZ); 1 spec., ditto, but 18.i.1967 (LUNZ); 2 specs., Kaitorete Spit, 0–5 m, 9.iv.1999, J.W. Early (dried dogfish carcass at top of beach (LUNZ); 1 spec., Christchurch, Kennedy’s Bush Road, 15.xii.1969, R.M. Emberson (inside house) (LUNZ); 1 spec., Heathcote, 5.x.1962, J. Olsson (on floor) (LUNZ); 2 specs., Pleasant Point, 5th Canterbury, 30.x.1961, N.G. Blakemore (LUNZ); 1 spec., Christchurch, 1917 (BMNH); 1 ♀, Lyttelton, x.1902, J.J. Walker (BMNH). MK: 1 spec., Mt. Cook National Park, Black Birch Stream, 1.xii.1999, J.W.M. Marris (under dead hare) (LUNZ); 1 spec., Mt. Gerald St., L. Tekapo, 12.iv.1985, B. Fraser (flying plots study) (LUNZ). OL: 2 specs., Paradise, 9.i.1945, C.E. Clarke (AMNH, BMNH); 1 ♂, Paradise, 9.i.1945 (BMNH); 1 ♂, ditto, but 1.i.1945 (BMNH); 3 specs., Arthurs Pr., near Queenstown, 24.ii.1925, C.E. Clarke (AMNZ). CO: 1 spec., Otago (BMNH); 4 specs., Bannockburn, 20.i.1945, C.E. Clarke (BMNH); 1 spec., Waitaki Co., Omaramu River Bed, 20.i.1962, W.B. Paterson (ex carrion) (AMNZ); 1 spec., Bannockburn, 20.ii.1945, C.E. Clarke (AMNZ); 1 ♀, Bannockburn, C.E. Clarke; 1 ♂ & 1 ♀, Paradise (BMNH). DN: 1 spec., Upper Wedderburn, 22.x.1923, C.E. Clarke (AMNZ); 1 spec., Lowar, Otago Peninsula, Tertatello, 14.ii.1940, C.E. Clarke (BMNH); 1 spec., Pilots Beach, Otago Harbour, iii.1999 (in dry seabird carcasses) (CJN); 1 spec., Taiaroa Head, 26.iii.1970, R. Jackson (*Puffinus griseus* nest) (LUNZ). SD: 1 spec., South Trio Island, 15.ii.1995, R.M. Emberson (litter ex bird nest) (LUNZ). Unknown localities: 1 spec., 296, Canterbury (BMNH); 16 specs., New Zealand, without further data (BMNH). Chatham Islands: 1 spec., Rangatira 30 m, 29.xi.1992, J.W.M. Marris (litter ex *Olearia Macropiper*/*Meticytus* forest) (LUNZ); 1 spec., South East Island, 7.xi.1970, J.I. Townsend (at night in bush) (NZAC); 6 specs., Mangere Island, 75 m, 2.xii.1992, J.W. Marris (in dead and dried up *Puffinus griseus* cadaver) (AMNZ); 6 specs., ditto, but LUNZ; 5 specs., South East I., 31.xii.1998, R.M. Emberson (under dead broad billed prions) (LUNZ); 1 spec., South East Island, Summit track, 20.i.1998, G. Taylor (LUNZ); 3 specs., South East Island, Woolshed Bush, 20.i.1998, R.M. Emberson (on ground at night) (LUNZ); 2 specs., Mangere, 26.xi.2004, R.M. Emberson & P. Syrett (above hut) (LUNZ); 3 specs., South East Island, Whalers Bay, 14.i.1997, J.W.M. Marris (under vegetation near bush edge) (LUNZ); 2 specs., South East Island, Whalers Bay Track,15.i.1997, collector unknown (on trees and logs at night) (LUNZ); 1 spec., Pitt Island, Glory Bay, 5.i.1999, R.M. Emberson (in dry cattle carcass) (LUNZ); 1 spec., ditto, but 2.i.1997 (in rubbish pit) (LUNZ; 6 exs. in coll. TLAN).

##### Distribution.

New Zealand mainland, Chatham Islands (Figs 758, 761).

##### Biology.

Found under vegetation, in dry carrion, on tree logs at night, on ground at night, in litter, in petrel burrows, under logs, in birds nests, swept from tussocks, or in compost.

##### Re-description.

Body length: PEL: 3.75–5.00 mm; EL: 2.35–3.25 mm; APW: 1.40–1.75 mm; PPW: 2.75–3.75 mm; EW: 3.15–4.30 mm.

Body (Fig. 422), rectangular oval, convex, elytral humeri not prominent, elytra light brown, shining, with slight metallic luster, pronotum black, shining; legs, mouthparts and antennal scape dark brown; antennal club black.

**Figures 423–431.**
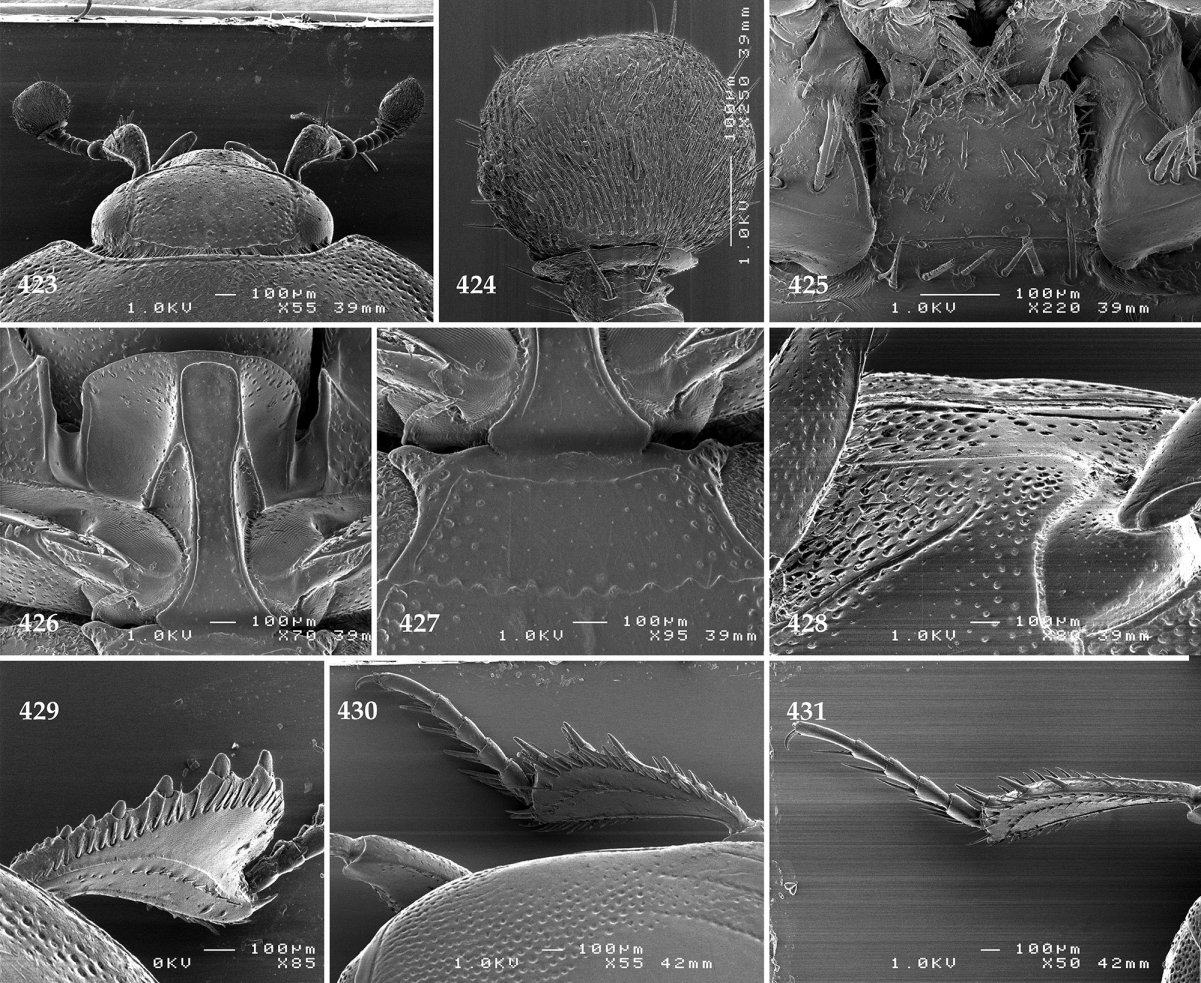
**423** Saprinus (Saprinus) detritus (Fabricius, 1775) head, dorsal view **424** antennal club, dorsal view **425** mentum, ventral view **426** prosternum **427** mesoventrite **428** lateral disc of metaventrite + metepisternum **429** protibia, dorsal view **430** mesotibia, dorsal view **431** metatibia, dorsal view.

Antennal scape (Fig. 423) slightly thickened, punctate, lower margin carinate, with several setae; antennal club (Figs 424) covered with dense short sensilla intermingled with sparse longer erect setae; sensory structures of antennal club not examined.

Mandibles dorso-laterally densely punctate, rounded, outer margin carinate; sub-apical tooth on left mandible large, almost perpendicular to outer mandibular margin; labrum finely and sparsely punctate, convex, with slight median depression; labral pits present, each with two labral setae; mentum (Fig. 425) sub-quadrate, anterior and posterior angles slightly projected, anterior margin with deep median notch, surface around it with several longer setae, lateral margins with a single row of much shorter ramose setae, disc of mentum finely imbricate, with sparse short setae; other mouthparts not examined.

Clypeus rectangular, slightly depressed medially, coarsely and densely punctate; supraorbital stria complete, slightly carinate; frontal stria weakened and interrupted medially (occasionally complete); frontal disc (Fig. 423) with coarse and dense punctation, slightly finer than that of clypeus, interspaces imbricate; eyes convex, visible from above.

Pronotal sides (Fig. 422) gradually narrowing anteriorly, on apical third strongly narrowed, apical angles prominent, pronotal depressions weakly impressed, anterior incision for head rather deep; marginal pronotal stria well impressed, carinate laterally, weakened anteriorly, visible along its entire length from dorsal view; pronotal disc laterally with depressed, coarsely and densely punctate band originating approximately near pronotal depressions, not reaching apical angles; between it and lateral pronotal margin an impunctate band present. Along basal margin of pronotum a double to triple row of variously sized punctures present, weakened before ante-scutellar area; rest of pronotal disc glabrous or only with scattered microscopic punctation. Pronotal hypomeron with scattered microscopic setae; scutellum small, visible.

Elytral epipleura densely and coarsely punctate; marginal epipleural stria complete; marginal elytral stria well impressed, carinate, continued as weakened, but complete apical elytral stria. Humeral elytral stria finely impressed on basal third; inner subhumeral stria present as median fragment (occasionally almost connected basally with humeral elytral); four dorsal elytral striae 1–4 well impressed, in punctures; first and second dorsal elytral striae about the same length, reaching approximately half of elytral length apically; third elytral stria very shortened, present as short basal fragment; fourth dorsal elytral stria the longest, surpassing elytral half apically, curved towards sutural elytral stria and connected with it; sutural elytral stria deeply impressed, impunctate, apically connected with apical elytral stria. Elytral disc on apical fourth with very coarse and dense confluent punctures, laterally entering and filling first elytral interval, mesally punctures present only on apical fourth; several punctures present mesally also along bases of second and third dorsal elytral striae; punctures before elytral margin not creating a glabrous band; along marginal elytral stria a narrow band of punctation present; rest of elytral disc and elytral flanks only with scattered microscopic punctation.

Propygidium on basal half almost impunctate, on apical half with dense punctation, interspaces between punctures less than their diameter; pygidium with even coarser and denser punctation, interspaces imbricate.

Anterior margin of median portion of prosternum (Fig. 426) almost straight; marginal prosternal stria present only laterally; prosternal process between carinal prosternal striae coarsely punctate, slightly depressed; carinal prosternal striae carinate, sub-parallel, slightly divergent apically, united in front (Fig. 426), prosternal process in profile even; lateral prosternal striae carinate, rather short, apically attaining carinal prosternal striae at about two-thirds of their length.

Discal marginal mesoventral stria (Fig. 427) well impressed, carinate, complete, anteriorly slightly inwardly arcuate; disc medially with sparse fine punctation, laterally larger sparse punctures appear; meso-metaventral sutural stria complete, undulate; intercoxal disc of metaventrite flattened, in male with longitudinal median line but without median depression, medially with scattered microscopic punctation, along lateral and basal margins coarse but sparse punctures appear; lateral metaventral stria (Fig. 428) deeply impressed, carinate, almost straight, ending short of metacoxae; lateral disc of metaventrite (Fig. 428) slightly concave, with dense shallow large setigerous punctures; metepisternum (Fig. 428) similar, but in deeper and larger punctures, on fused metepimeron punctures becoming much sparser and not setigerous; metepisternal stria deep, present almost along entire metepimeron and metepisternum, interrupted.

Intercoxal disc of first abdominal ventrite completely striate laterally; disc with the exception of median area with dense punctures of various sizes; along basal margin of sternite much finer microscopic punctation.

Protibia (Fig. 429) slightly dilated, outer margin with five moderately large triangular teeth topped by large denticle, denticles diminishing in size proximally, followed by five minute denticles; setae of outer row regularly spaced, rather short; setae of median row much shorter and somewhat sparser than those of outer row; anterior protibial stria shortened apically; protarsal groove rather deep; protibial spur large, bent, growing out from apical margin of protibia; outer part of posterior surface punctate, separated from glabrous and narrow median part of posterior surface by a ridge; median row of posterior surface with a row of rather long strongly sclerotized setae; posterior protibial stria recognizable dense row of strongly sclerotized setae becoming stiffer and longer apically; apical margin of protibia ventrally with three short denticles; inner row of setae double, setae short, diminishing in size basally.

Mesotibia (Fig. 430) and metatibia (Fig. 431) also similar to other congeners; denticles on outer, and apical margin not as long and stiff as in *S. chathamensis*, and denticles on metatibia sparse (only four present, growing in size apically).

**Figures 432–438.**
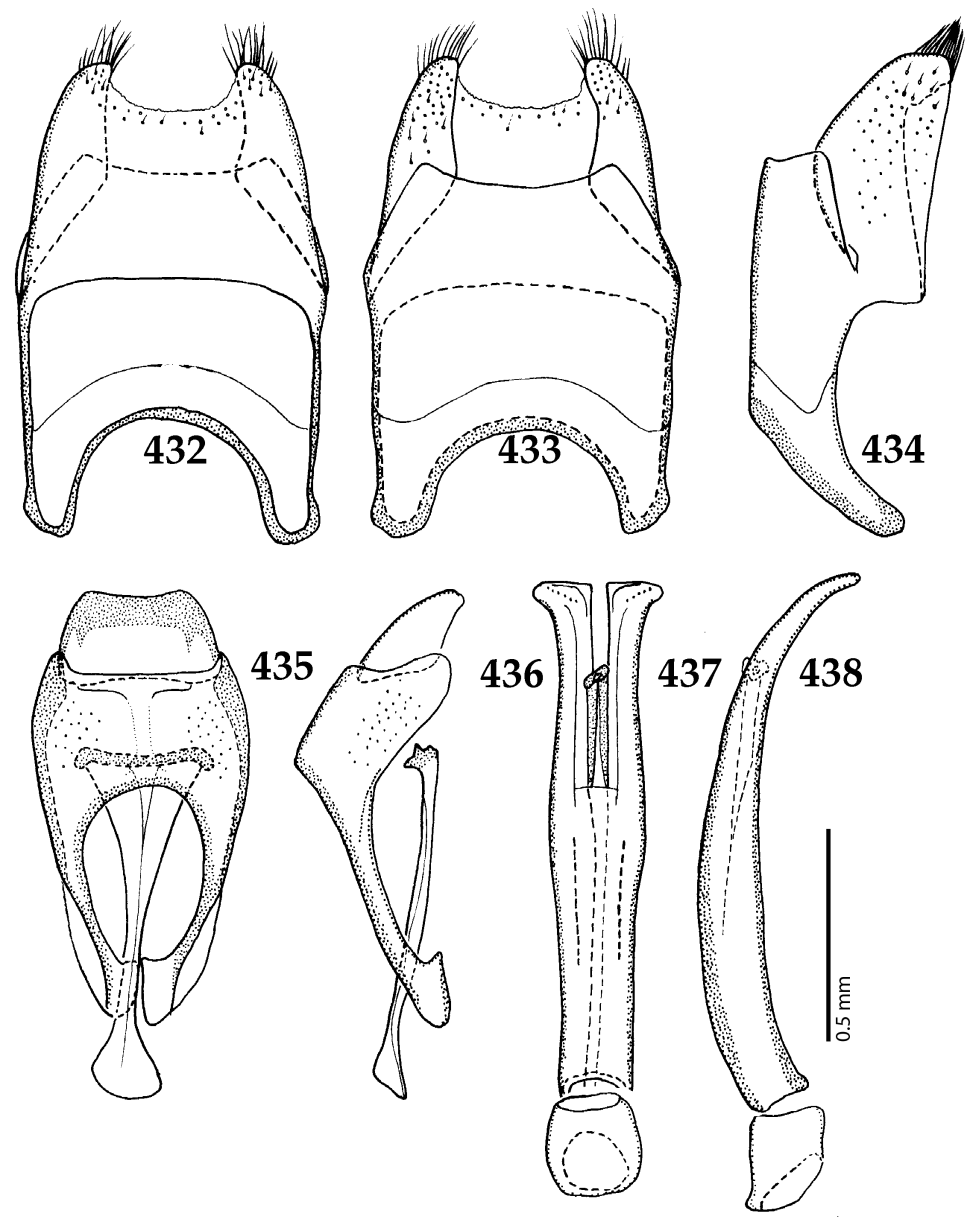
**432** Saprinus (Saprinus) detritus (Fabricius, 1775) male terminalia: 8^th^ sternite + 8^th^ tergite, ventral view **433** ditto, dorsal view **434** ditto, lateral view **435** male terminalia: 9^th^ + 10^th^ tergites, dorsal view; spiculum gastrale, ventral view **436** male terminalia: 9^th^ + 10^th^ tergites; spiculum gastrale, lateral view **437** male terminalia: aedeagus, dorsal view **438** ditto, lateral view.

Male genitalia. Eighth sternite (Figs 432–433) apically with several pseudo-pores, entirely fused; apex of eighth sternite laterally with two tufts of dense, moderately long setae; eighth tergite and eighth sternite fused laterally (Fig. 434). Ninth tergite (Figs 435–436) typical for the subfamily; anterior margin of tenth tergite slightly inwardly arcuate; spiculum gastrale (Fig. 435) gradually dilated on apical half; apical end strongly sclerotized; basal end abruptly dilated, outwardly arcuate. Aedeagus (Figs 437–438) slightly thickened medially, constricted before apex; apex dilated; parameres fused along their basal half (roughly), thence separated; basal piece of aedeagus short, ratio of its length : length of parameres 1 : 6; aedeagus curved from lateral view (Fig. 438).

#### Saprinus (Saprinus) grandiclava

Taxon classificationAnimaliaColeopteraHisteridae

Kanaar, 1989

Figs 439 , 440–448 , 449–457 , 755

Saprinus grandiclava Kanaar, 1989: 285.

##### Type locality.

Indonesia: Papua: Jayapura.

**Figure 439.**
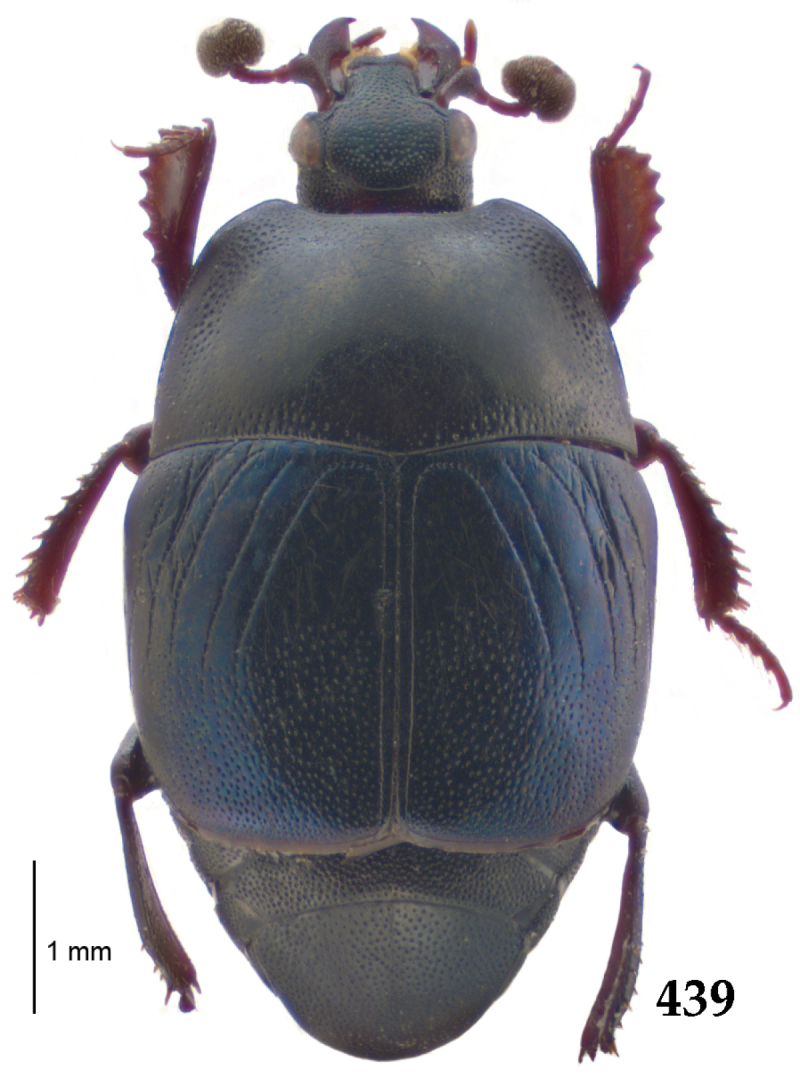
Saprinus (Saprinus) grandiclava Kanaar, 1989 habitus, dorsal view.

##### Type material examined.

*Saprinus grandiclava* Kanaar, 1989: holotype, ♂, with extracted genitalia, glued on a separate triangular mounting card under the specimen, with the following labels: “HOLLANDIA / NW. GUINEA” (black-framed printed label); followed by: “Museum Leiden / Collectie / Doesburg / rec. 1973” (printed); followed by: “HOLOTYPUS, ♂ / Saprinus / grandiclava / P. Kanaar des. 1989” (printed-written label) (NCB). Paratypes: 1 ♂, with identical labels as the Holotype, with an extra blue, margined label: “Museum Leiden / Histeroidea/ collection / dr P. Kanaar” (NCB); 4 ♀♀ paratypes, with extracted genitalia and identical labels to those of the Holotype (NCB). One of the female paratypes bears another light yellow label: “10-118” (pencil-written) added by the senior author; followed by another green label: “Photographed by / B. Rhode” (printed) (NCB).

##### Additional material examined.

Indonesia. West Papua: 1 spec., road Nabire-Ilaga, km 62, 250 m, 24.vii.1991, M. Balke leg. (ZSM). PAPUA NEW GUINEA. 1 ♀, Huon Golf, Sattelberg, 1899, Biró leg. (HNHM).

##### Biology.

Unknown.

##### Distribution.

Indonesia: Papua (former Irian Jaya). New to Papua New Guinea (Fig. 755).

##### Remarks.

This is the only species of the Australopacific Saprininae that lacks sensory areas (or plaques) on the ventral side of the antennal club.

##### Re-description.

Body length: PEL: 4.00–4.50 mm; APW: 1.50–1.75 mm; PPW: 3.25 mm; EL: 2.75 mm; EW: 3.50–3.60 mm.

Body (Fig. 439), convex, pronotum dark brown with faint bronze metallic luster, elytra dark blue with metallic tinge; propygidium and pygidium black; legs, mouthparts and antennal scape castaneous brown to red; antennal club black.

**Figures 440–448.**
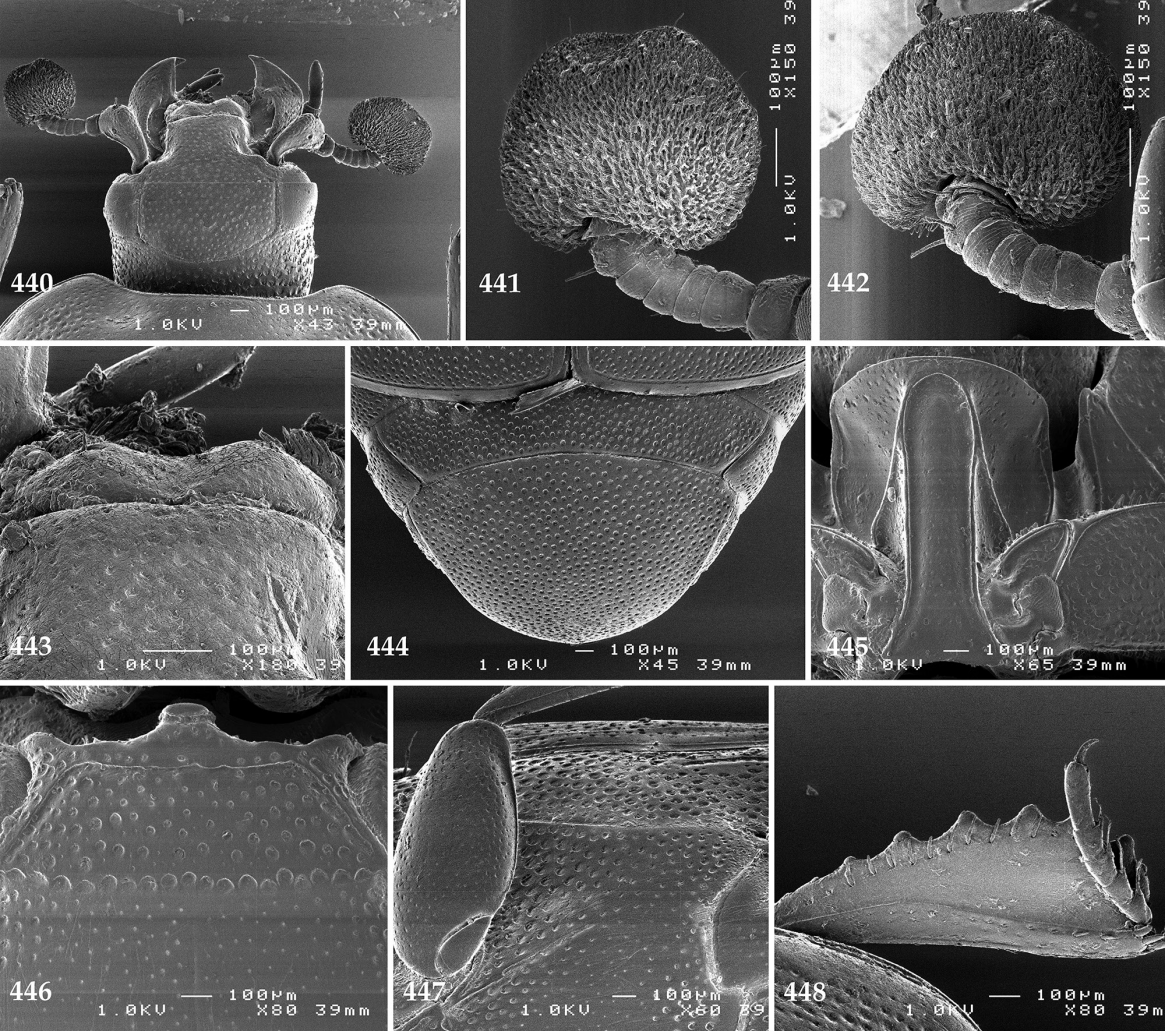
**440** Saprinus (Saprinus) grandiclava Kanaar, 1989 head, dorsal view **441** antennal club, dorsal view **442** ditto, ventral view **443** clypeus + labrum, dorsal view **444** propygidium + pygidium **445** prosternum **446** mesoventrite **447** lateral disc of metaventrite + metepisternum **448** protibia, dorsal view.

Antennal scape (Fig. 440) slightly thickened, coarsely punctate, with several short setae; antennal club (Figs 441–442) unusually large for the genus, shaped like an inverted heart, apical part distinctly depressed, covered with dense short sensilla intermingled with sparse longer erect setae; sensory structures of antennal club externally absent, vesicle(s) not examined.

Mandibles dorso-laterally rather coarsely punctate, rounded, mandibular apex acute, sub-apical tooth on left mandible obtuse; labrum (Fig. 443) finely and sparsely punctate, convex, with distinct median depression; labral pits present, labral setae broken off; other mouthparts not examined.

Clypeus (Fig. 443) flattened, rugosely punctate; frontal stria widely interrupted medially, but not prolonged onto clypeus, supraorbital stria slightly carinate; frontal disc (Fig. 440) densely punctate, punctures separated by about their diameter; eyes convex, well visible from above.

Pronotal sides (Fig. 439) moderately narrowing anteriorly, apical angles obtuse, pronotal depressions deep, well-impressed; anterior incision for head shallow; marginal pronotal stria complete, carinate, visible along its entire length from dorsal view; pronotal disc laterally with a depressed band of deep dense elongate punctures originating approximately in pronotal depressions, but not reaching basal angles of pronotum, between it and pronotal margin a narrow smooth band present; rest of the pronotal disc with only scattered microscopic punctation; several rows of fine ovoid punctures present along pronotal base reaching ante-scutellar area in a form of a single row of fine punctures; pronotal hypomeron glabrous, with scattered punctation; scutellum small, visible.

Elytral epipleura with sparse fine punctures; marginal epipleural stria complete; marginal elytral stria well impressed, carinate, continued as complete (weakened) apical elytral stria. Humeral elytral stria well impressed on basal third, faintly connected to inner subhumeral stria, between it and first dorsal elytral stria a short complementary fragment of stria present; four dorsal elytral striae 1–4 well impressed, in fine punctures, all striae about the same length, approximately apically attaining elytral half; fourth dorsal elytral stria basally connected with complete sutural elytral stria that is apically connected with apical elytral stria; elytral disc on apical half (roughly) punctate, punctures separated by about twice their diameter, along elytral suture punctation reaches slightly further basally than along elytral flanks, punctures almost not entering elytral intervals; punctation becomes finer but denser apically.

Propygidium (Fig. 444) almost completely exposed, densely punctate, punctures separated by less than their diameter; pygidium (Fig. 444) with sparser punctation, punctures separated by about their own diameter; interspaces in both cases imbricate.

Anterior margin of median portion of prosternum (Fig. 445) rounded laterally; marginal prosternal stria present only laterally; prosternal process between carinal prosternal striae slightly convex, on prosternal apophysis and near united apices of carinal prosternal striae depressed, sparsely and finely punctate; carinal prosternal striae carinate, parallel on basal two-thirds, on apical third slightly divergent and thence slightly convergent, united in front (Fig. 465); lateral prosternal striae carinate, strongly convergent, apically attaining carinal prosternal striae almost near their united apices.

Anterior margin of mesoventrite (Fig. 446) almost straight; discal marginal mesoventral stria well impressed, carinate, complete; disc with coarse and dense punctation, punctures of various sizes; meso-metaventral sutural stria indicated by a dense row of large punctures; intercoxal disc of metaventrite flattened; disc for the most part almost smooth, surface around apical corners and along lateral metaventral stria with scattered punctation, punctures of various sizes, along posterior margin and especially behind metacoxa several rows of larger punctures appear; lateral metaventral stria (Fig. 447) well impressed, carinate, almost straight, shortened; lateral disc of metaventrite (Fig. 447) slightly concave, with dense shallow large punctures of various sizes; metepisternum (Fig. 447) with similar punctures; metepisternal stria present only along fused metepimeron.

Intercoxal disc of first abdominal ventrite completely striate laterally; disc along basal and lateral margins with shallow punctures of various sizes; rest of ventrite with scattered microscopic punctation.

Protibia (Fig. 448) slightly dilated, outer margin with six moderately large triangular teeth topped by small rounded denticle, teeth diminishing in size proximally; setae of outer row regular, short; protarsal groove deep; anterior protibial stria complete; setae of median row worn off; two tarsal denticles present near tarsal insertion; protibial spur large, bent, growing out from apical margin of protibia; outer part of posterior surface glabrous, separated from glabrous and narrow median part of posterior surface by a definite stria bearing a sparse row of setae; posterior protibial stria complete, apically with a short but dense row of setae; inner row of setae double, setae dense, though probably worn off partially.

Mesotibia slender, outer margin with a regular row of sparse short denticles slightly growing in size apically, another row of much shorter sparser denticles situated on anterior surface of mesotibia; setae of outer row regular, partially worn off, almost as long as denticles themselves; setae of median row shorter and finer; posterior mesotibial stria almost complete; anterior surface of mesotibia slightly rugulose; anterior mesotibial stria almost complete, terminating in two tiny denticles; mesotibial spur stout, short; apical margin of mesotibia anteriorly with two short denticles; claws of apical tarsomere slightly bent, shorter than half its length; metatibia slenderer and longer than mesotibia, in all aspects similar to it, but denticles on outer margin much shorter and sparser.

**Figures 449–457.**
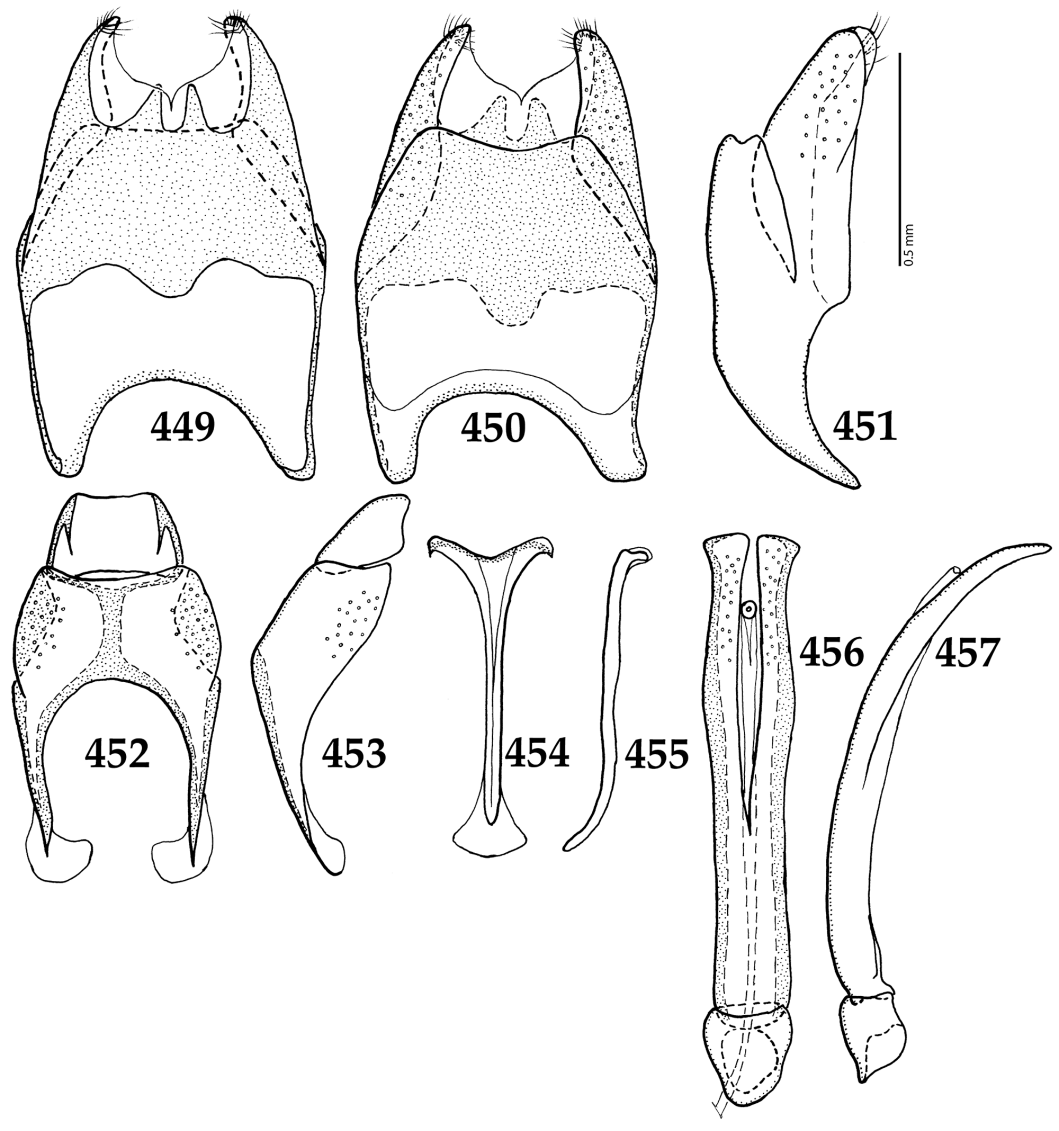
**449** Saprinus (Saprinus) grandiclava Kanaar, 1989 male terminalia: 8^th^ sternite + 8^th^ tergite, ventral view **450** ditto, dorsal view **451** ditto, lateral view **452** male terminalia: 9^th^ + 10^th^ tergites, dorsal view **453** ditto, lateral view **454** male terminalia: spiculum gastrale, ventral view **455** ditto, lateral view **456** male terminalia: aedeagus, dorsal view **457** ditto, lateral view

Male genitalia. Eighth sternite (Fig. 449) apically with thin vela; apices with a tuft of short setae; eighth tergite (Fig. 450) faintly inwardly arcuate apically; eighth sternite and tergite fused laterally (Fig. 451). Ninth tergite with strongly sclerotized median line, apico-laterally with pores, basally strongly sclerotized. Tenth tergite (Fig. 452) basally and apically faintly inwardly arcuate; laterally strongly sclerotized. Spiculum gastrale on basal end sub-triangular, outwardly arcuate; apex inwardly arcuate (Figs 454–455). Aedeagus (Figs 456–457) bi-sinuate, apical third with pores and pseudopores; parameres narrowly separated on their apical half. Phallobase short, its length approximately equals 1/6 of paramere’s length.

#### Saprinus (Saprinus) laetus

Taxon classificationAnimaliaColeopteraHisteridae

Erichson, 1834

Figs 458 , 459–467 , 468–476 , 763

Saprinus laetus Erichson, 1834: 179.
Saprinus cyanellus Marseul, 1855: 387 – Synonymized with S. pseudocyaneus by Dahlgren (1968): 264.
Saprinus westraliensis Blackburn, 1903: 106 – Synonymized with S. pseudocyaneus by Dahlgren (1971): 49.
Saprinus (Saprinus) pseudocyaneus White, 1846 – **syn. n.**
Saprinus mastersii MacLeay, 1871: 158 – **syn. n.**
Saprinus gayndahensis MacLeay, 1871: 158 – **syn. n.**

##### Type locality.

Australia.

**Figure 458.**
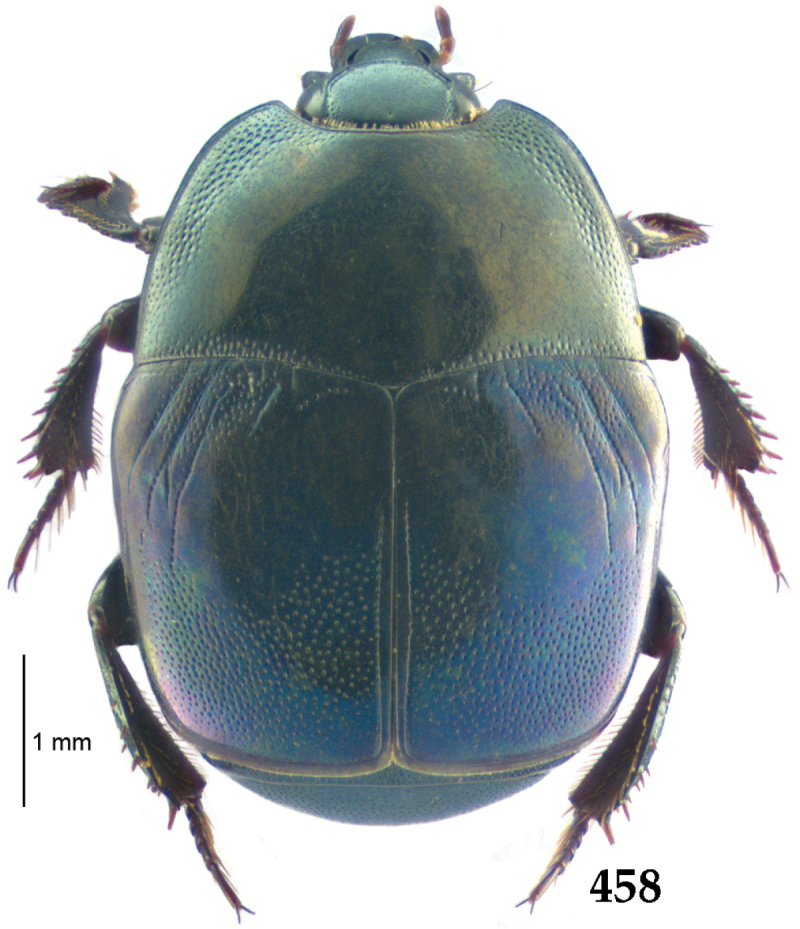
Saprinus (Saprinus) laetus Erichson, 1834 habitus, dorsal view.

##### Type material examined.

*Saprinus laetus* Erichson, 1834: Lectotype, present designation: most likely a ♀, pinned, with the following labels: “49088” (printed); followed by: “Hist. Coll. Coleoptera / Nr. 49088 / Saprinus laetus Er. x / Nova Holland., Hope / Zool. Mus. Berlin” (white, black margined label, printed); followed by: “laetus / Er. / Hist. cyaneus Pk. / N. Holl. Hope” (grey, black-margined label); followed by: “Saprinus laetus / Erichson, 1834 / LECTOTYPE 2014 / des. Lackner & Leschen” (red label, written) (ZMHUB). Paralectotype, designated herein, most likely a ♀, pinned, all tarsi (except right mesotarsus) broken off, with the printed label from Berlin Museum, identical to that of the lectotype, followed by the red paralectotype label. Three more paralectotypes, designated herein, most likely ♂♂, with labels identical to those of the female paralectotype (ZMHUB). This species has been described from unknown number of specimens and its taxonomic identity is hereby fixed by the lectotype designation.

*Saprinus pseudocyaneus* White, 1846: Lectotype, present designation: 1 ♂, with genitalia extracted and glued to the same mounting card as the specimen, left metatarsus missing, with following labels: “New Zealand” (round label, written); followed by: “Type” (round, red-margined printed label); followed by: “Saprinus pseudocyaneus / White Zool. Erebus & Terror” (written); followed by: “Sapr. cyaneus / G Dahlgren det.” (printed-written); followed by: “Saprinus pseudocyaneus / White, 1846 / LECTOTYPE / Des. T. Lackner & R. Leschen 2014” (red label, written) (BMNH). This specimen is clearly the type of *S. pseudocyaneus*, and may be errorneously described from New Zealand, where it normally does not occur; though there are four specimens among *S. pseudocyaneus* material collected from New Zealand in 1930 (see below). For the taxonomic history of this species see Kuschel (1987). This species was described from unknown number of specimens and the lectotype designation fixes the identity of the species.

*Saprinus gayndahensis* MacLeay, 1871: Lectotype, sex unidentified, present designation, with the following labels: “Gayndah, / Queensland / Masters” (printed); followed by: “Co-type” (printed); followed by: “13739 / *Saprinus* / *gayndahensis* / Mast. / Queensland / Cotype” (written); followed by: “SAMA Database / No. 25-029675” (printed); followed by: “Saprinus / gayndahensis / W.S. Macleay, 1871 / LECTOTYPE des. T. / Lackner 2016” (red label, written) (SAMA). This species was described from an unknown number of specimens and the lectotype designation fixes the identity of this species.

*Saprinus mastersii* MacLeay, 1871: Lectotype, present designation: ♂, with genitalia dissected, dismembered and glued together with the right elytron to the same mounting card as the specimen, right antennal club and both metatarsi broken off, with the following labels: “Gayndah” (printed); followed by: “On permanent loan from / MACLEAY MUSEUM / University of Sydney” (printed); followed by: “SYNTYPE” (red label, printed); followed by: “ANIC Database No. / 25 053122” (printed); followed by: “SAPRINUS (s.str.) / pseudocyaneus / White, 1846 / Det. T. Lackner 2010” (printed-written) (ANIC); followed by: “ANIC / image” (yellow label, printed); followed by: “LECTOTYPE / *Saprinus mastersii* / MacLeay, 1871 / Des. T. Lackner & / R. Leschen 2015” (red label, printed). In the collection of ANIC there are two specimens labeled syntypes of *S. mastersii*. One of them (male) is designated as the lectotype and the second one (unsexed) is a misidentified specimen of *S. cyaneus cyaneus* (Fabricius, 1775). There is a large “*Saprinus mastersi*” label, which isn’t actually attached to either specimen but is placed beside them in the unit tray. The syntype material of *S. mastersii* came that way from Macleay Museum (C. Lemann, CSIRO, pers. comm. 2014). This species was described from an unknown number of specimens and the lectotype designation fixes the species identity.

*Saprinus westraliensis* Blackburn, 1903: Lectotype, present designation: ♀, mounting label bears: “T”, “7250” and “W.A.”; followed by: “Type / H.T.” (round, red-margined printed label); followed by: “♀” (written); followed by: “Australia. / Blackburn Coll. / B.M. 1910-236” (printed); followed by: “Saprinus / westraliensis, Blackb.” (written); followed by: “Sapr. cyaneus / G. Dahgren det.” (printed-written); followed by: “Saprinus westraliensis / Blackburn, 1903 / LECTOTYPE / Des. T. Lackner & R. Leschen 2014” (red label, written) (BMNH). This species has been described from an unknown number of specimens and the lectotype designation fixes the identity of the species.

*Saprinus cyanellus* Marseul, 1855: 387: Lectotype, present designation: ♀, pinned, right protibia missing, right mesotibia missing, both metatarsi missing, with the following labels: tiny, rectangular pink label, followed by: “28 / Saprinus / cyanellus m. / ♀ N. Holland / illegible” (pink label, written); followed by: “MUSEUM PARIS / cyanellus / COLL. / DE MARSEUL 1890” (pink label, printed-written); followed by: “TYPE” (red-printed label); followed by: “Saprinus cyanellus / Marseul, 1855 / LECTOTYPE 2014 / des. T. Lackner” (red label, written) (MNHN). This species has been described based on unknown number of specimens and the lectotype designation fixes its taxonomic identity.

##### Additional material examined.

NEW GUINEA. “New Guinea” Schmidt’s collection, locality doubtuful (ZMHUB).

AUSTRALIA. Tasmania: 3 ♂♂ & 1 ♀, Queenstown, 19.i.1982, Bornemissza leg. (HMNH); 3 ♂♂ & 42 specs., Bothwell, 19.iii.1982, Bornemiszza leg. (HMNH); 7 ♂♂ & 28 specs., St. Helens, 10 km NW, 12.xii.1981, Bornemiszza leg. (HNMH); 3 ♂♂ & 4 specs., Borell, 9.iv.1982, Bornemissza leg. (HMNH); 1 ♂ & 1 ♀, Launceston, Littler Collection (NZAC); 4 specs., Flinders Island, Bass Strait, 1.–6.v.1989, B.M. Doube & K.G. Wardaugh (dung baited pitfall traps) (ANIC); 11 specs., Newstead, Launceston, 20.xi.1981, S. Fearn (on Stink Lily flower) (ANIC); 10 specs., Hollow Tree, 9 km S of, 42°37'S, 146°56'E, 12.ii.1992, Tom Gush (from dead Tasmanian devil) (ANIC); 1 spec., Beechford, 1.i.1985, S. Fearn (ANIC); 1 spec., Ocean Beach, Coastal Reserve, 42°09'S, 145°16'E, 6.ii.1992, Tom Gush (in debris on beach) (ANIC); 1 spec., Mole Creek, 21.ii.2000, E. van der Duys (QM); 1 spec., Paratoh (?), no more details (MAMU); 1 spec., King’s Creek, Lea; 2 specs., West Tamar (SAMA); 1 spec., Launceston (SAMA). Victoria: 2 ♀♀, Wartook Reservoir, 10.x.1930, Grampians (NZAC); 2 ♂♂, Wartook Reservoir, Grampians, 10.x.[19]30, C.E. Clarke Collection (BMNH); 2 ♂♂ & 2 ♀♀, Victoria, without further details (ZMHUB); 1 ♂, Langwarrin, 30.x.1930 (AMNZ); 7 specs., Murray Valley Highway, 19 km E by S of Hattah, 34.47S 142.29E, 2.xi.1988, T. Weir, J. Lawrence & M. Hansen (from dead kangaroo) (ANIC); 13 specs., Whitfield, 3 km W, 36°47'S, 146°23'E, 14.x.1990, Tom Gush (on dead kangaroo) (ANIC); 2 specs., Tawonga, 2 km W, 36°40'S, 147°08'E, 13.x.1990, Tom Gush (on dead sheep) (ANIC); 1 spec., Cranbourne, 2.x.1960, B.P. Moore; 2 specs., Yanakie, 9 miles S, 13.v.1963, Bornemisza (ANIC); 1 spec., Kulkyne State Forest, 8.vi.1968, G.W. Anderson (ANIC); 1 spec., upper Jardine River, Cape York peninsula, 11°17'S, 142°35'E, 20.x.1979, M.S. & B.J. Moulda (MAMU). New South Wales: 1 ♂, Blue Mts., i.1909, G.E. Bryant (BMNH); 4 specs., Coricudgy, 20.ix.2006, G. Hangay leg. (HMNH); 1 spec., ditto, but 27.ii.2000, pitfall traps, G. Hangay (HMNH); 1 ♂, ditto, but 5.i.1998, pit trap, G. Hangay leg. (HMNH); 1 ♀, ditto, but 5.–26.ii.2002, pitfall traps, G. Hangay (HMNH); 1 ♀ & 4 specs., Great Dividing Range, Mt. Coricudgy, 941 m, 32°50.8'S, 150°17.8'E, 24.x.2000, Hungarian Entomological Expedition, A. Podlussány, G. Hangay & I. Rozner leg. (HMNH); 20 specs., Congo, 8 km SE by E of Moruya, 22.–26.iii.1982, M.S. Upton (ANIC); 1 spec., ditto, but 6.xi.1983 (ANIC); 6 ♀♀ & 4 ♂♂, 12 km E Walcha, 22.xi.1992, S. Watkins (carrion) (ANIC); 2 specs., Tomakin, 35.49S 150.11E, 25.xii.1988, W. Dressler (dead shark head) (ANIC); 1 spec., 8.5 km NE Gubbata, 33.35S 146.37W, 4.–12.ii.1999, D. Driscoli (pitfalls, ungrazed roadside, no spinifex) (ANIC); 42 specs., Morisset, 3 km SW, 33°08'S, 151°27'E, 19.viii.1990, Tom Gush (on dead cow) (ANIC); 9 specs., Sandy Hollow, 5 km NW, 32°18'S, 150°31'E, 7.vi.1992, Tom Gush (from dead fox) (ANIC); 8 specs., Mullengandra, 2 km N, 35°53'S, 147°11'E, 13.x.1990, Tom Gush (on dead kangaroo) (ANIC); 7 specs., Lightgow, 8 km SW, 33°31'S, 150°05'E, 17.xi.1991, Tom Gush (on dead kangaroo) (ANIC); 5 specs., Peats Ridge, 20.x.1990, Vince Lorimer (on dead dog) (ANIC); 3 specs., Mangrove Mountain, 27.x.1990, Vince Lorimer (on 10-day dead dog) (ANIC); 2 specs., Cox’s River, 4.ix.1932, Chadwick (under dead rabbit) (ANIC); 1 spec., Federal Highway, 19.x.1963, B.P. Moore (ANIC); 1 spec., Gundaroo Road, 18.xi.1971, B.P. Moore (ANIC); 9 specs., Calindary Station, 10.ix.1970, W.J.M. Vestjens (ANIC); 2 specs., Flat Rock State Forest, 35°23'S, 150°16'E, 26.viii.1990, Tom Gush (under dead pig) (ANIC); 1 spec., Mount White, 33°27'S, 151°10'E, 2.ix.1990, Tom Gush (in excrement on rock) (ANIC); 1 ♀, Matakana NE Hillston, 19.ix.1993, S.G. Watkins (carrion) (ANIC); 1 spec., Bombala, xi.1948, A. Dyce (ANIC); 1 ♀, Winghan, 4.xii.1994, J. Stockard (chicken manure) (ANIC); 2 ♀♀, Tallowwood Flat Road, Dingo State Forest, 26.xi.1994, S. Watkins (carrion) (ANIC); 1 ♂, Wherrol Flat, 24.xi.1992, S. Watkins (dead fox) (ANIC); 2 specs., Marulan, 22.x.1964, 13 m, Bornemissza; 2 specs., Durras Lake, 24.xii.1964, Bornemissza (ANIC); 1 ♂, Mt. Coricudgy, nr. Olinda Station, 1.–7.iii.2002, G. & K. Hangay coll. (ANIC); 1 spec., Katarapko Island, 27.vi.1998, ex Black Box Intercept (ANIC); 1 spec., Thalgarrah, 18.viii.1985, M. Coombs (QM); 2 specs., ditto, but 15.viii.1985 (QM); 1 spec., ditto, but 19.viii.1985 (QM); 14 specs., Lilyvale, 9.ix.1972, D.A. Doolan (MAMU); 1 spec., Brooklana Plantation, 23.ix.1979, D.A. Doolan (MAMU); 1 spec., Ashville, 12.ix.1980, D.A. Doolan (MAMU); 2 specs., North Ryde P.C., 17.iii.1967, D.A. Doolan (MAMU); 1 spec., Goonoo S.F. 6 km SW, Mendooran, 6.iv.1979, D.K. McAlpine & B.J. Day (MAMU); 2 specs., 40 km S of Mudgee, on Ilford Road, 13.v.1981, B.J. Day (on dead fox) (MAMU); 1 spec., Warrumbungle National Park, 25.iii.1973, collector unknown (MAMU); 1 spec., Cooma, 800 m, no date, J. Sedláček (BPBM); 1 spec., Lake Callabona, A. Zietz (SAMA). South Australia: 1 ♂, Adelaide, Hait, 1886 (BMNH); 4 specs., Althorpe Island, 17.xi.1979, Mrs. E.F. Lawley (HMNH); 3 ♂♂, 56 km SW Roxby D., 27.viii.2000, dead sheep, M.A. Hielkema leg. (HMNH); 1 ♂ & 1 ♀, nr. Tarlee, 20.x.1988, under carrion, Mrs. Lawley leg (HMNH); 1 ♂, 52 km NW Port Augusta, 25.viii.2000, in/under dead sheep, M.A. Hielkema leg. (HMNH); 4 specs., Flinders Island, 33.43S 134.31E, 27.vii.–2.viii.1987, J.E. Feehan (under dead sheep) (ANIC); 1 spec., Wai Kerie, 24.viii.1958, B.P. Moore (ANIC); 2 specs., 1 mile SW of Carierwerloo HS., 29.ix.1968, Key, Upton & Balderson (ANIC); 2 specs., William Creek, 28.54S 136.21E, 16.vi.1991, I. Gee (ANIC); 1 spec., Wirringina Well, 28.56S 135.45E, 10.viii.1991, I. Gee (ANIC); 1 spec., Old Anderson’s Well, 28.44S 135.55E, 24.viii.1991, I. Gee (ANIC); 1 spec., Murray Bridge, 31.x.1978, R. Laughlin (yellow trap) (ANIC); 3 ♀♀, 33°59'S, 140°30'E, 31 km NW Renmark, Dry Frogamerry paddock, Calperum Station, 8.–21.viii.1995, K. Pullen, FIT (ANIC); 5 ♀♀ & 1 ♂, 34°00'S, 140°47'E, 19 km N of Renmark, Clover Lake paddock, Calperum Station, 10.viii.–7.ix.1995, K. Pullen, F.I.T. (ANIC); 3 ♀♀, 33°53'S, 140°44'E, 32 km N Renmark, Amalia paddock, Calperum Station, 6.ix.–11.x.1995, K. Pullen, F.I.T. (ANIC); 1 spec., Nullabor, 27.x.1963, J. Sedláček; 1 ♀, 16 km S of Port Augusta, 10 m, 26.x.1963, J. Sedláček (BPBM); 1 spec., Penong, ii.1957, C. Warner (SAMA); 1 spec., Ardrossan, J.G.O. Tepper (SAMA); 1 spec., Yerilka Creek, 24.viii.1953, G.F. Gross (SAMA); 1 spec., Kangaroo Island, Vivonne Bay, 1926, Museum Expedition (SAMA); 2 spec., Kangaroo Island, J.G.O. Tepper (SAMA); 1 spec., Purple Downs, without further data (SAMA); 6 specs., Kangaroo Island, Ravine de Casoara, 25.x.1951, G.F. Gross (under wallaby carcass) (SAMA); 12 specs., Hindmarsh Park Station, 19.x.1967, P.E. Gniel (in dustbin) (SAMA); 3 specs., Mt. Lofty, J.G.O. Tepper (SAMA); 1 spec., Kelso (SAMA); 4 specs., Mt. Serle, N Flinders Ra., Hale & Tindale (SAMA); 1 spec., Port Lincoln; 6 specs., Blanchetown, Mrs. Kreusler (SAMA); 1 spec., Halletts Cove, 26.i.1967, N. McFarland (on freshly dead sheep carcass) (SAMA). Western Australia: 2 ♀♀ & 1 ♂, Marloo Station 1934, Wurarga, Gebr. Goerling S.G. (ZMHUB); 1 ♀, Fremantle, 1956, E.C. Chapman (BMNH); 1 spec., Woomera, ix.–xi.1953, F.L. Hill (BMNH); 5 specs., Dumbleyoung, 19.ix.1979, E. Gowing-Scopes (BMNH); 4 specs., 3 km E. of Pingrup, 18.ix.1979, E. Gowing-Scopes (BMNH); 1 ♂, Perth, 22.vii.1908, G.E. Bryant (BMNH); 5 specs., Hamelin Pool, 19.xii.1981, K. & E. Carnaby (ANIC); 16 specs., Carnavon, viii.1980, K. & E. Carnaby (ANIC); 1 spec., Lake Grace, 16.xi.1979, K. & E. Carnaby (ANIC); 2 specs., Katanning, 12.iv.1951, M.M.H. Wallace (ANIC); 3 specs., Wilga, 8.xi.1973, K. Carnaby (ANIC); 3 specs., ditto, but 20.iv.1973 (ANIC); 2 specs., Floreat Park, 18.ix.1978, G.P. Hall (ANIC); 2 specs., Maidu Vale, 28.viii.1959, B.P. Moore (ANIC); 2 ♂♂, Boyup Brook, 1.xi.1981, Ex. Carnaby Coll. (ANIC). 1 spec., Wilga, 23.viii.1981, G.A. Holloway (MAMU). 1 ♂ & 9 specs., Stirling Ranges, Bluff Knoll, 150 m, 12.xi.1963, J. Sedláček (BMNH); 20 specs., Eucla, 5 m, 28.x.1963, J. Sedláček (BMNH); 2 specs., 5 km W of Lake Cave, 1–140 m, 7.–8.xi.1963, J. Sedláček (BMNH); 2 specs., Murchison River, 21.xi.1963, J. Sedláček (BMNH); 1 spec., 16 km N Northampton, 21.xi.1963, J. Sedláček (BMNH); 4 specs., Warren River, W.D. Dodd (SAMA); 1 spec., Eucla (SAMA); 1 spec., Everard ranges to Warburton Ranges, A. Brumby (SAMA). Australian Capital Territory: 11 specs., Canberra, 9.–12.xii.1957, W.J.M. Vestjens (ANIC); 7 specs., ditto, but 28.x.1960, collector unknown (ANIC); 1 spec., Uriarra, 35.17S 148.55E, 28.x.1992, M. Tindale-Biscoe (ANIC); 3 specs., Turner, x.1965, K. Pullen (ANIC); 1 spec., Black Mountain, lower E slope, 3.vi.1985, K.R. Pullen (ANIC); 1 spec., ditto, but 20.ix.1972, T.E. Bellas (ANIC). Queensland: 1 ♀, “Cape York” (ZMHUB); 1 spec., Leyburn, 22.iii.1975, J. Macqueen; 1 spec., Great Barrier Reef, Swain Reefs, Bylund Cay, vii.1985, 21.47S 152.24E, H. Heatwole (under dead bird) (ANIC); 1 spec., Brisbane, vii.1920, collector unknown (ANIC); 1 spec., 1 mile S of Herberton, 17.24S 145.23E, 14.v.1969, Brooks & Nebois (on dead wallaby) (ANIC); 1 ♀, Thomson Creek, 16.06.31S 145.26.25E, 140 m, Trunk F.I.T. #14, 9.xi.–19.xii.1998, Coll. Simon Grove (ANIC); 3 specs., Allinga Chinchilla, 26°41'S, 150°38'E, Grace Lithgow; 1 spec., Boggom, via Taroom, 25°27'S, 150°08'E, 13.xi.1996–i.1997, Cook & Monteith (FIT trap, baited) (QM); 12 specs., Mt. Tambourine, xii.1919, H. Pottinger (QM); 1 spec., Fraser Island, Eurong, 11.vii.2002, I. Thrash (bird carcass) (QM); 4 specs., Caloundra, 28.ix.1913, H. Hacker (QM); 6 specs., Brisbane, 5.ix.1916, H. Hacker (QM); 1 spec., ditto, but vii.1920, H. Pottenger (QM); 2 specs., 39 miles N Morven, 2.v.1963, E.C. Dahms (under Kangaroo body) (QM); 2 specs., Cairns (QM); 1 ♀, Keysland, 26.i.–20.iv.1995, 26°12'S, 151°44'E, G.B. Monteith (pitfall trap, open forest) (QM); 1 spec., near Riversbourne National Park, Brisbane Valley, 25.xi.1973, K. McDonald (QM); 1 ♀, Blackdown, Tableland Via Dingo, 1.–6.ii.1981, G.B. Monteith (QM); 1 spec., Yarran Downs, 27°40'S, 148°38'E, 9.iv.2001, NHT project (pitfall trap, dung) (QM); 1 spec., Mt. Moffat National Park, 1 km WNW Lots Wife, 24°58'S, 147°57'E, 820 m, 1.–30.x.1998, D.J. Cook (dung pitfall) (QM); 1 ♀, Bogantungan, 9 km N, 840 m, 23°34'S, 147°18'E, 25.–26.x.2000, G.B. Monteith (open forest, dung pitfall) (QM); 1 spec., Mill Creek, 17°30'S, 145°27'E, above 1000 m, 5.–6.ii.1999, G. Monteith & D.J. Cook (open forest, rotten bait) (QM); 1 spec., Wallum, 26°57'S, 147°42'E, 15.iv.2001, NHT Project (dung pitfall) (QM); 2 specs., Lake Bindegolly, near bridge, 28°06'S, 144°13'E, 26.–27.viii.2000, D.J. Cook (dung pitfall) (QM); 9 specs., Womblebank Gap, Site 2, 25°49'S, 148°15'E, 620 m, 28.ix.–30.x.1998, D. Cook (dung pitfall, vinescrub in brigalow) (QM). Northern Territory: 3 specs., c. 85 km NW of Yuendumu, 22.15S 131.48E, 29.vi.1970, S. Parker (ANIC); 1 spec., 12.43S 143.17E, 9 km ENE of Mt. Tozer, 5.–10.vii.1986, T. Weir & A. Calder, collected at light (ANIC); 2 ♀, NSW, Bogan River, J. Armstrong (ANIC); 4 specs., Box Hole, 1.–11.x.1953, G.H. Gross (SAMA). Unknown Localities: 5 specs., Australia occid., 1192, no further details (HMNH); 1 ♀ & 7 specs., Australia, without further data (HMNH); 1 ♂ & 1 ♀, Australia 1934, C.E. Clarke, no further details (AMNZ). Lord Howe Island: 2 ♂♂ & 6 specs., 21.–30.i.1985, G.F. Bornemissza leg. (HNMH).

##### Biology.

A common and widespread Australian volant predator, found mainly on decomposing carcasses of mammals.

##### Distribution.

Australia: all states; newly reported from Lord Howe Island (Fig. 763). The species was also doubtfully reported from the island of New Guinea and New Zealand (not shown on the maps).

##### Remarks.

Erichson (1834:179) described the species *Saprinus laetus* from Australia; this species was later synonymized by Marseul (1862:714) with the species *Saprinus cyaneus* (Fabricius, 1775). However, examination of the type specimens of *S. laetus* indicates that this species is conspecific with *Saprinus pseudocyaneus* White, 1846, and has priority over *S. pseudocyaneus* White, 1846. Mazur (1997, 2011) erroneously lists *S. gayndahensis* as a synonym of *S. cyaneus*; we find instead no characters (in the lectotype examined) that separate *S. gayndahensis* from *S. laetus*, and synonymize these two species. *Saprinus laetus* is a variable species, though genitalia support its unity. Specimens from Lord Howe Island are generally most distinctive from the remaining populations, generally lighter in color, especially in the elytra, which are light green as opposed to the dark green/violet or dark blue present in specimens from Australia; also the elytral striation is reduced and the mesoventrite is covered with scattered to moderately dense punctures.

##### Re-description.

Body length: PEL: 3.50–5.25 mm; APW: 1.25–2.00 mm; PPW: 2.85–4.15 mm; EL: 2.25–3.50 mm; EW: 3.00–4.65 mm. Body (Fig. 458) rectangular oval, convex, elytra widest at humeri, elytra light to dark green, dark blue, occasionally dark brown or even black, shining, with metallic luster; pronotum light yellow to dark bronze with slight greenish hue, at times even purple, coppery or entirely dark, in most cases metallic; legs, mouthparts and antennae dark brown to almost black.

**Figures 459–467.**
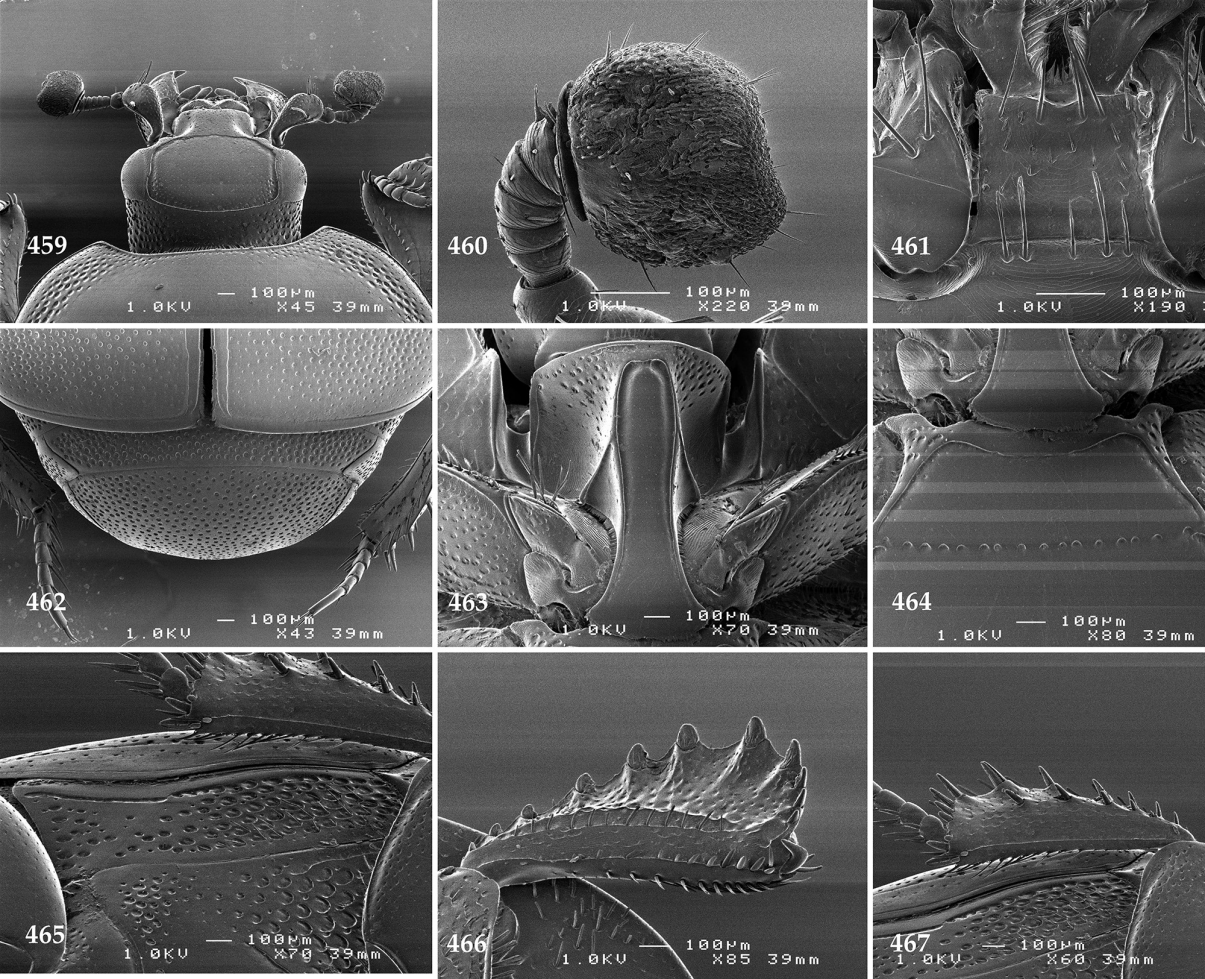
**459** Saprinus (Saprinus) laetus Erichson, 1834 head, dorsal view **460** antennal club, ventral view **461** mentum, ventral view **462** propygidium + pygidium **463** prosternum **464** mesoventrite **465** lateral disc of metaventrite + metepisternum **466** protibia, dorsal view **467** mesotibia, ventral view.

Antennal scape (Fig. 459) slightly thickened, punctuate, with two rather long and several shorter setae; antennal club wider than long, semi-circular, somewhat truncate apically, covered with dense short sensilla intermingled with sparse longer erect setae; sensory structures of antennal club (Fig. 460) in form of four rather small ellipsoid ventral sensory patches, which are usually difficult to discern; vesicle(s) not examined.

Mandibles (Fig. 459) dorso-laterally punctuate, rounded, outer margin slightly carinate, mandibular apex acute, sub-apical tooth on left mandible obtuse; labrum finely and sparsely punctuate, convex, with deep median depression; labral pits present, each with two short labral setae; terminal labial palpomere elongate, pointed apically, approximately twice as long as pen-ultimate, its width about three times its length; terminal maxillary palpomere elongate, about two- and half times as long as pen-ultimate, approximately three times as long as wide, pointed apically; mentum (Fig. 461) sub-quadrate, laterally with a double row of short ramose setae, several setae present also on disc, anterior margin medially with low notch surrounded by a tuft of several longer sparse setae.

Clypeus (Fig. 459) even, occasionally very faintly convex or even concave, sloping down laterally, finely punctate; frontal and supraorbital striae complete, frontal stria not prolonged onto clypeus; frontal disc (Fig. 459) with similar punctation as that of clypeus, near posterior margin approximately medially a fine definite fovea present; eyes convex, well visible from above.

Pronotal sides (Fig. 458) narrowing anteriorly, apical angles acute, pronotal depressions present, fine, anterior incision for head rather deep; marginal pronotal stria carinate, visible along its entire length from dorsal view, reaching pronotal base; pronotal disc laterally with a depressed band of deep dense coarse elongate punctures originating approximately in pronotal depressions, not reaching basal angles of pronotum, between it and pronotal margin a narrow impunctate band present; rest of the pronotal disc glabrous; several rows of punctures present along pronotal base, ante-scutellar area impunctate; pronotal hypomeron with short amber setae often visible only from ventral view, originating from scattered fine punctures; scutellum small, visible.

Punctation of elytral epipleuron ranges from none to rather dense; marginal epipleural stria complete; marginal elytral stria well impressed and carinate, slightly present also on elytral base, continued as weakened, but complete apical elytral stria that is connected to incomplete sutural elytral stria. Humeral elytral stria well impressed on basal third, occasionally doubled, sometimes connected to variably short median fragment of inner subhumeral stria; outer subhumeral stria present as short basal fragment situated on elytral humerus; elytra usually with four dorsal elytral striae; striae 1–2 in most cases longer than striae 3–4, reaching often approximately elytral half apically, but can be considerably shorter, even very reduced, third stria almost always shorter than striae 1–2, at times represented by only a basal fragment, fourth stria always very shortened apically, present only as a basal hooked fragment not linked to shortened sutural elytral stria or only as a very short row of points, in extreme cases completely absent; sutural elytral stria in punctures, erased on basal fifth to fourth, apically connected to apical elytral stria. Surface between humeral elytral stria and first and second elytral intervals usually with sparse to moderately dense punctures; in most cases on first elytral interval elongate strioles present, at times these strioles present also on second interval or punctures in intervals aciculate; sutural elytral stria abbreviated on basal fourth to fifth, well-impressed, in round punctures, apically connected with apical elytral stria; elytral punctation very variable, usually punctures present on apical third to half (roughly), but punctation can cover almost the entire elytron except for glabrous part between third elytral and sutural stria, often glabrous ‘mirror’ (=polished area) considerably larger; punctures usually fine, sparse, separated by about twice their diameter, but can be denser, especially laterally where they can be separated by less than their own interval, occasionally aciculate (especially those near elytral intervals); along elytral flanks often entering elytral intervals, but not necessarily so, punctation usually not reaching elytral apex.

Propygidium (Fig. 462) very densely punctate, punctures separated by less than their own diameter; pygidium (Fig. 462) with sparser punctation.

Anterior margin of median portion of prosternum (Fig. 463) rounded; marginal prosternal stria present laterally and also as medial fragment; prosternal process between carinal prosternal striae slightly convex, glabrous to microscopically punctate, surface near united apices of carinal prosternal striae distinctly depressed; carinal prosternal striae carinate, often distance between them rather narrow, slightly bisinuate, occasionally slightly divergent on anterior third, thence in most cases convergent and united in front (Fig. 463); lateral prosternal striae carinate, rather short, apically attaining carinal prosternal striae at about three-fourths of their length.

Discal marginal mesoventral stria (Fig. 464) well impressed, carinate, complete, anteriorly distinctly inwardly arcuate; disc often smooth, at times with sparse fine punctation, occasionally (specimens from Lord Howe Island) with moderately dense punctures; meso-metaventral sutural stria indicated by a row of punctures; intercoxal disc of metaventrite in males with very faint longitudinal depression, best observable on basal third; in females slightly convex; disc of metaventrite for the most part almost smooth, only in post-metacoxal area several rows of fine punctation appear; lateral metaventral stria well impressed, carinate, straight, shortened; lateral disc of metaventrite slightly concave, with dense and large shallow setigerous punctures separated by less than their to about their own diameter, occasionally between large punctures another type of much finer sparse punctures present; metepisternum (Fig. 465) rather broad, its punctures deeper and denser, on fused metepimeron punctures becoming much sparser; metepisternal stria present often only along fused metepimeron, at times along metepisternum also present in a form of short intermittent fragments or string of deep punctures (in rare cases complete).

Intercoxal disc of first abdominal ventrite completely striate laterally; disc almost smooth, only along basal and lateral margins with shallow fine punctures.

Protibia (Fig. 466) dilated, outer margin with about five low teeth topped by triangular denticle, followed by approximately three tiny denticles diminishing in size proximally; setae of outer row regular, short; protarsal groove shallow; anterior protibial stria present on basal third, next obliterated; setae of median row placed on low stria-like ridge, shorter than those of outer row; two tarsal denticles present near tarsal insertion; protibial spur (Fig. 466) large, bent, growing out from apical margin of protibia; apical margin of protibia ventrally with three tiny denticles; outer part of posterior surface slightly obscurely variolate, punctate, separated from imbricate-punctate narrow median part of posterior surface by a ridge bearing a row of setae; posterior protibial stria complete, bearing along its length a row of microscopic setae turning into well-sclerotized minuscule denticles apically; inner row of setae single, lamellate.

Mesotibia (Fig. 467) on outer margin with a row of about seven distally growing in size denticles, several of them situated on low teeth; setae of outer row rather strongly sclerotized, longer than denticles; setae of median row shorter and finer than those of outer row; posterior mesotibial stria not complete; mesotibial spur rather short; on anterior face of mesotibia (Fig. 476) a row of about five widely-spaced denticles present; anterior face of mesotibia imbricate-punctate; anterior mesotibial stria complete, terminating in two tiny denticles; inner anterior denticles weakly developed, usually only one or two present; inner row of setae rather dense. Metatibia basically similar to mesotibia, but longer and more slender, and denticles of both rows sparser; denticles of outer row do not grow out from low teeth.

**Figures 468–476.**
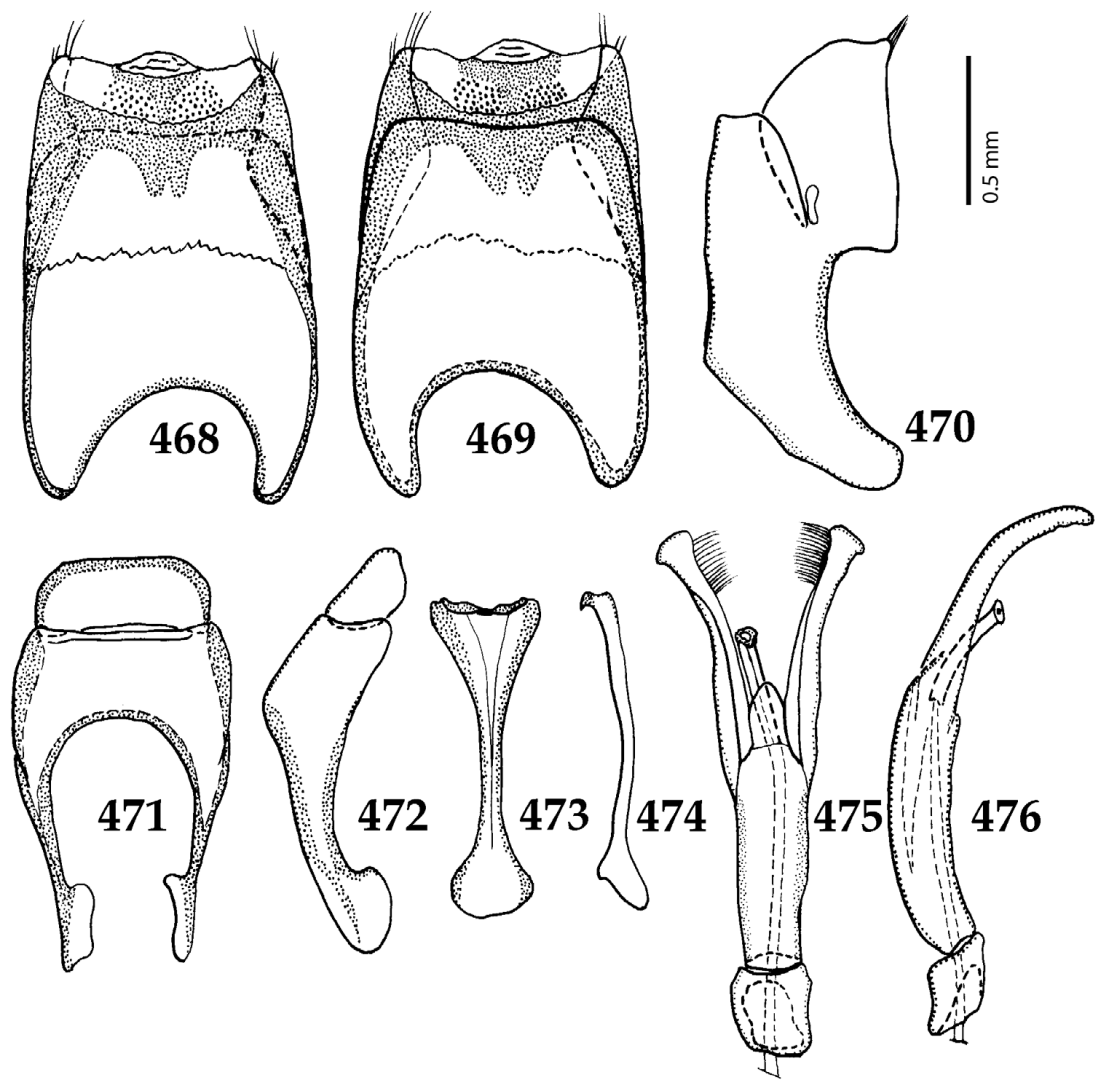
**468** Saprinus (Saprinus) laetus Erichson, 1834 male terminalia: 8^th^ sternite + 8^th^ tergite, ventral view **469** ditto, dorsal view **470** ditto, lateral view **471** male terminalia: 9^th^ + 10^th^ tergites, dorsal view **472** ditto, lateral view **473** male terminalia: spiculum gastrale, ventral view **474** ditto, lateral view **475** male terminalia: aedeagus, dorsal view **476** ditto, lateral view.

Male genitalia. Eighth sternite (Figs 468–469) apically with strongly sclerotized part, completely fused medially, apex laterally with several setae, vela present, with several rows of pores; eighth tergite apically faintly inwardly arcuate, almost straight, basally strongly inwardly arcuate; eighth tergite and eighth sternite fused laterally (Fig. 470). Ninth tergite (Figs 471–472) laterally strongly sclerotized; ninth tergite basally faintly inwardly arcuate; tenth tergite basally inwardly arcuate, rounded apically; spiculum gastrale (Figs 473–474) apically gradually dilated from approximately its mid-length; basal end inwardly arcuate; apex with strongly sclerotized part. Aedeagus (Figs 475–476): parameres widely separated approximately from mid-length, apices of parameres on inner side with a comb of regular setae; basal piece of aedeagus rather short, ratio of its length : length of parameres approximately 1 : 6.

#### Saprinus (Saprinus) nitiduloides

Taxon classificationAnimaliaColeopteraHisteridae

Fairmaire, 1883

Figs 477 , 478–486 , 487–493 , 755

Saprinus nitiduloides Fairmaire, 1883: 3.

##### Type locality.

Papua New Guinea: The Duke of York Islands: Mioko Island.

**Figure 477.**
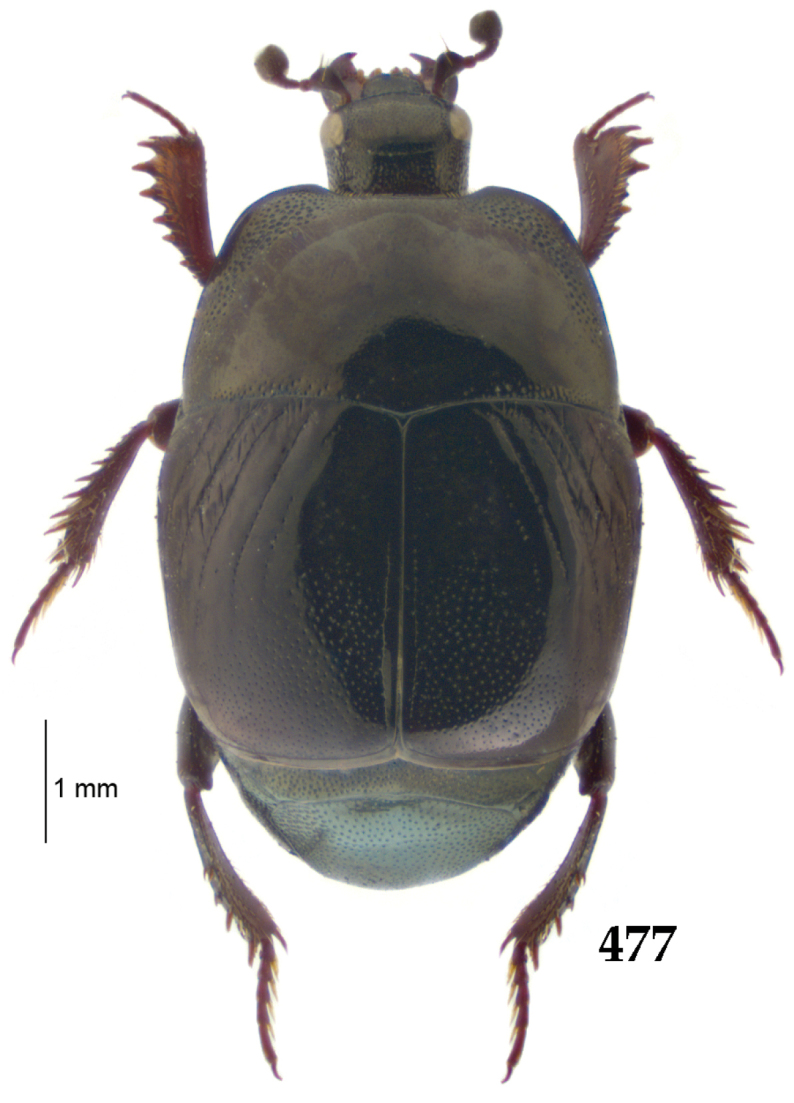
Saprinus (Saprinus) nitiduloides Fairmaire, 1883 habitus, dorsal view.

##### Type material examined.

*Saprinus nitiduloides* Fairmaire, 1883: Lectotype, present designation, ♂, side-mounted on a triangular mounting card, with terminalia glued on another triangular card under the specimen, right metatarsus missing, with the following labels: “Fairmaires / unique specimen / given me in Paris / 8.5.[18]88” (written); followed by: “*Saprinus* / *nitiduloides* / Fairm. /Mioko” (written); followed by: “G. Lewis Coll. / B.M. 1926-369.” (printed); followed by: “Type” (round, red-margined label); followed by: “09-087” (yellow, pencil-written label, added by the senior author); followed by: “Saprinus / nitiduloides / Fairmaire, 1883 / LECTOTYPE / des. T. Lackner ‘11” (red label, written) (BMHN). Although Fairmaire (1883:3) does not mention the number of specimens he based his description of *S. nitiduloides* on, he does mention the sex of the species: male. It is presumed that Fairmaire had only one specimen at hands and he examined its sex. The specimen discovered in BMNH bears Lewis’ note that it was “Fairmaire’s unique specimen given me in Paris”. However, because of the uncertainty of the number of specimens used for the species’ description we herein designate its lectotype to fix the species identity.

##### Additional material examined.

PAPUA NEW GUINEA. New Britain: 1 ♂, Duke of York, date and collector unknown (BMHN); 1 ♂, ditto, but coll. J. Schmidt (ZMHUB); 8 ♂♂ + & 7 ♀♀, Ralum, 30.v.1896, E. Dahl S. (ZMHUB; 3 ♂♂ + 1 ♀ in coll. TLAN); 1 ♂, Gisiluve, Nakansi Mts., 1050 m, 26.vii.1956, R.J. Ford Jr. (BPBM); 1 ♀, Vunakanau, Gazelle Peninsula, 26.v.1956, J.L. Gressit (TLAN).

SOLOMON ISLANDS. 1 ♀, Guadalcanal, Aula, date and collector unknown (BMHN).

##### Biology.

Unknown, presumably similar to congeners.

##### Distribution.

Papua New Guinea: New Britain; Solomon Islands: Mioko, Guadalcanal (Fig. 755).

##### Remarks.

The specimens from New Britain are generally more sparsely punctated than are those from Solomon Islands or islands of Duke of York.

##### Re-description.

Body length: PEL: 3.00–4.35 mm; EL: 1.75–2.85 mm; APW: 1.10–1.55 mm; PPW: 2.40–3.25 mm; EW: 2.55–3.60 mm.

Body (Fig. 477) rectangular oval, convex, cuticle dark brown, shining, with slight metallic luster, pronotum darker, piceous black; elytra lighter; legs, mouthparts and antennal scape castaneous brown; antennal club darker.

**Figures 478–486.**
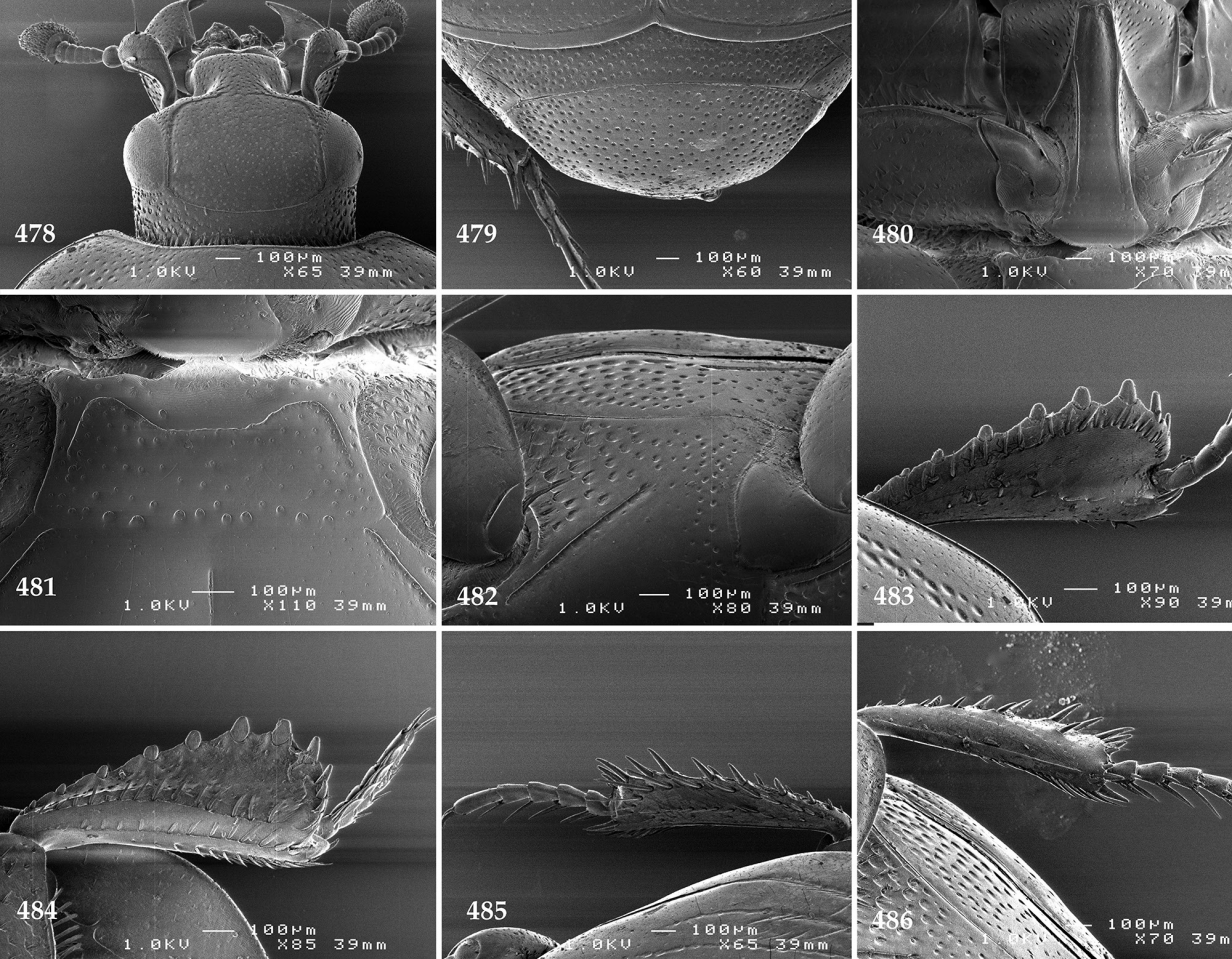
**478** Saprinus (Saprinus) nitiduloides Fairmaire, 1883 head, dorsal view **479** propygidium + pygidium **480** prosternum **481** mesoventrite **482** lateral disc of metaventrite + metepisternum **483** protibia, dorsal view **484** ditto, ventral view **485** mesotibia, dorsal view **486** ditto, ventral view.

Antennal scape (Fig. 478) black, slightly thickened, finely punctate, with two setae; antennal club covered with dense short sensilla intermingled with sparse longer erect setae; sensory structures of antennal club not examined.

Mandibles dorso-laterally finely punctate, rounded, mandibular apex acute, sub-apical tooth on left mandible obtuse; labrum finely and sparsely punctate, convex, with deep median depression; labral pits present, each with a single labral seta; other mouthparts not examined.

Clypeus (Fig. 478) sloping down laterally, finely punctate; frontal stria weakened medially, complete, prolonged onto clypeus, straight, supraorbital stria slightly carinate; frontal disc (Fig. 478) finely punctate, punctures separated several times their diameter, on posterior third often punctation disappears completely; small fovea present on posterior fourth medially; eyes convex, well visible from above.

Pronotal sides (Fig. 477) moderately narrowing anteriorly, apical angles prominent, pronotal depressions present, shallow to moderately deep; anterior incision for head deep; marginal pronotal stria complete (not reaching pronotal base in some individuals), carinate, visible along its entire length from dorsal view; pronotal disc laterally with a band of deep dense elongate punctures originating approximately in pronotal depressions, not reaching basal angles of pronotum, between it and pronotal margin a narrow smooth band present; rest of the pronotal disc with only scattered microscopic punctation, almost glabrous in some individuals; double row of fine ovoid punctures present along pronotal base not reaching ante-scutellar area; tiny ante-scutellar fovea present in several individuals; pronotal hypomeron glabrous; scutellum small, visible.

Elytral epipleura with sparse fine punctures, almost glabrous in some individuals; marginal epipleural stria complete; marginal elytral stria well impressed and slightly carinate, continued as complete (weakened) apical elytral stria. Humeral elytral stria well impressed on basal third, usually connected to long inner subhumeral stria; four dorsal elytral striae 1–4 well impressed, in fine punctures, striae normally reach or even slightly surpass approximately elytral half apically, usually about the same length, but can be variously shortened, intermittent or erased, but usually fourth stria the shortest, basally not connected with sutural elytral stria; sutural elytral stria well-impressed, in fine punctures, abbreviated on basal fourth, apically connected with apical elytral stria; between first and second elytral stria deep sparse longitudinal strioles present; elytral disc on apical half (roughly) punctate, punctures fine, sparse, separated by several times their diameter; punctures not becoming denser apically.

Propygidium (Fig. 479) densely punctate, punctures separated by their own to twice their own diameter; pygidium (Fig. 479) with similar, but sparser punctation, interspaces in propygidium imbricate.

Anterior margin of median portion of prosternum (Fig. 480) almost straight, rounded laterally; marginal prosternal stria present laterally and also as medial fragment; prosternal process between carinal prosternal striae flat, sparsely and finely punctate, on anterior third distinctly convex, surface near united apices of carinal prosternal striae distinctly depressed; carinal prosternal striae carinate, parallel on basal two-thirds, on apical third slightly divergent and thence slightly convergent (can be also almost parallel-sided along their entire course), united in front (Fig. 480); lateral prosternal striae carinate, rather short, apically attaining carinal prosternal striae at about two-thirds of their length.

Anterior margin of mesoventrite (Fig. 481) distinctly inwardly arcuate; discal marginal mesoventral stria well impressed, carinate, shortened laterally; disc with sparse fine punctation, punctures becoming larger near meso-metaventral suture (occasionally disc entirely glabrous); meso-metaventral sutural stria indicated by a (sparse) row of large punctures; intercoxal disc of metaventrite flattened, in male with longitudinal median depression; disc of metaventrite for the most part almost smooth, surface around longitudinal depression with scattered microscopic punctation, punctures of various sizes, along posterior margin several rows of punctation appear (in several specimens disc almost glabrous); lateral metaventral stria (Fig. 482) well impressed, carinate, almost straight, shortened; lateral disc of metaventrite (Fig. 482) slightly concave, with dense shallow large setigerous punctures; metepisternum (Fig. 482) similar, but with deeper and larger punctures without setae, on fused metepimeron punctures becoming much sparser; metepisternal stria present along fused metepimeron, along metepisternum present as short intermittent fragments; occasionally complete.

Intercoxal disc of first abdominal ventrite completely striate laterally; disc along basal and lateral margins with shallow punctures of various sizes; rest of sternite with scattered microscopic punctation.

Protibia (Fig. 483) slightly dilated, outer margin with around ten very low teeth topped by large denticle, denticles diminishing in size proximally; setae of outer row regular, short; protarsal groove shallow; anterior protibial stria present on basal two-thirds, next obliterated; setae of median row shorter and much sparser than those of outer row; two tarsal denticles present near tarsal insertion; protibial spur bent, growing out from apical margin of protibia; outer part of posterior surface (Fig. 484) slightly obscurely variolate, separated from glabrous and narrow median part of posterior surface by a definite stria bearing a row of setae; posterior protibial stria complete, bearing almost along its entire length dense row of setae; inner row of setae double, setae dense, shorter but finer than those of posterior protibial stria.

Mesotibia (Fig. 485) slender, outer margin with a row of sparse long denticles growing in size apically, another row of much shorter sparser denticles situated on anterior surface of mesotibia; setae of outer row regular, thick, almost as long as denticles themselves; setae of median row shorter and finer; posterior mesotibial stria shortened apically; anterior surface of mesotibia (Fig. 486) sparsely punctate; anterior mesotibial stria almost complete; mesotibial spur stout, short; apical margin of mesotibia anteriorly with two short denticles; inner margin of mesotibia with sparse row of short setae; claws of apical tarsomere slightly bent, shorter than half its length; metatibia slenderer and longer than mesotibia, in all aspects similar to it, but denticles on outer margin much shorter and sparser.

**Figures 487–493.**
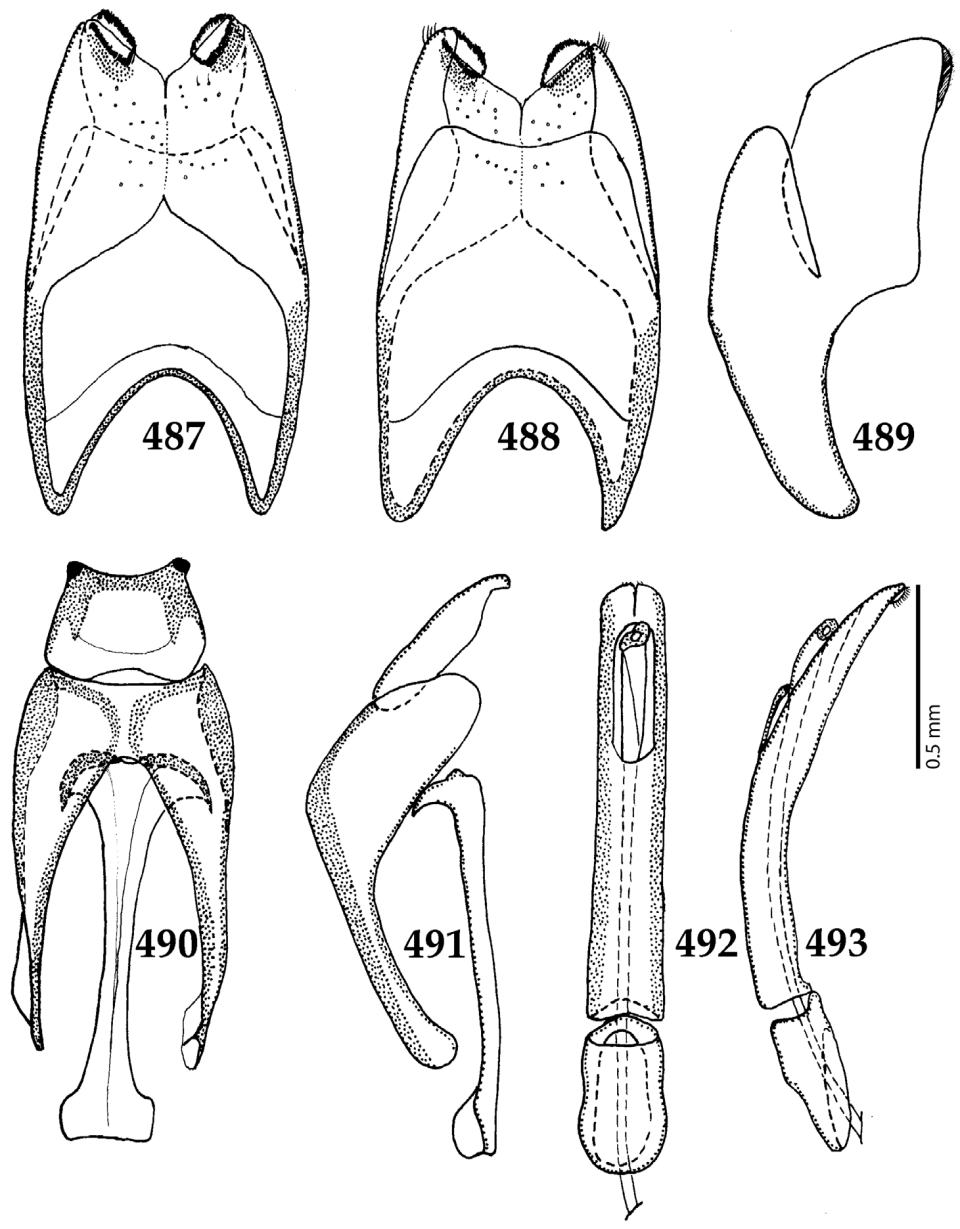
**487** Saprinus (Saprinus) nitiduloides Fairmaire, 1883 male terminalia: 8^th^ sternite + 8^th^ tergite, ventral view **488** ditto, dorsal view **489** ditto, lateral view **490** male terminalia: 9^th^ + 10^th^ tergites, dorsal view; spiculum gastrale, ventral view **491** male terminalia: 9^th^ + 10^th^ tergites; spiculum gastrale, lateral view **492** male terminalia: aedeagus, dorsal view **493** ditto, lateral view.

Male genitalia. Eighth sternite (Figs 487–488) fused medially, apex with several microscopic setae, vela present, adorned with a row of microscopic setae; eighth tergite and eighth sternite fused laterally (Fig. 489). Ninth tergite (Figs 490–491) typical for the subfamily; tenth tergite inwardly arcuate, apical angles strongly sclerotized, bent; spiculum gastrale (Fig. 490) gradually dilated on most of its apical half; basal end slightly dilated. Aedeagus (Figs 492–493) parallel-sided, with parameres fused along their basal half (roughly); basal piece of aedeagus short, ratio of its length : length of parameres 1 : 4; aedeagus slightly curved from lateral view; apex of aedeagus with a tiny patch of microscopic setae resembling a “suction cup”.

#### Saprinus (Saprinus) pacificus sp. n.

Taxon classificationAnimaliaColeopteraHisteridae

http://zoobank.org/A7D712E6-2F71-4DE8-8E57-7C0B64D8A2B5

Figs 494 , 495–503 , 504–509 , 751

##### Type locality.

Kiribati: Bikinibeu.

**Figure 494.**
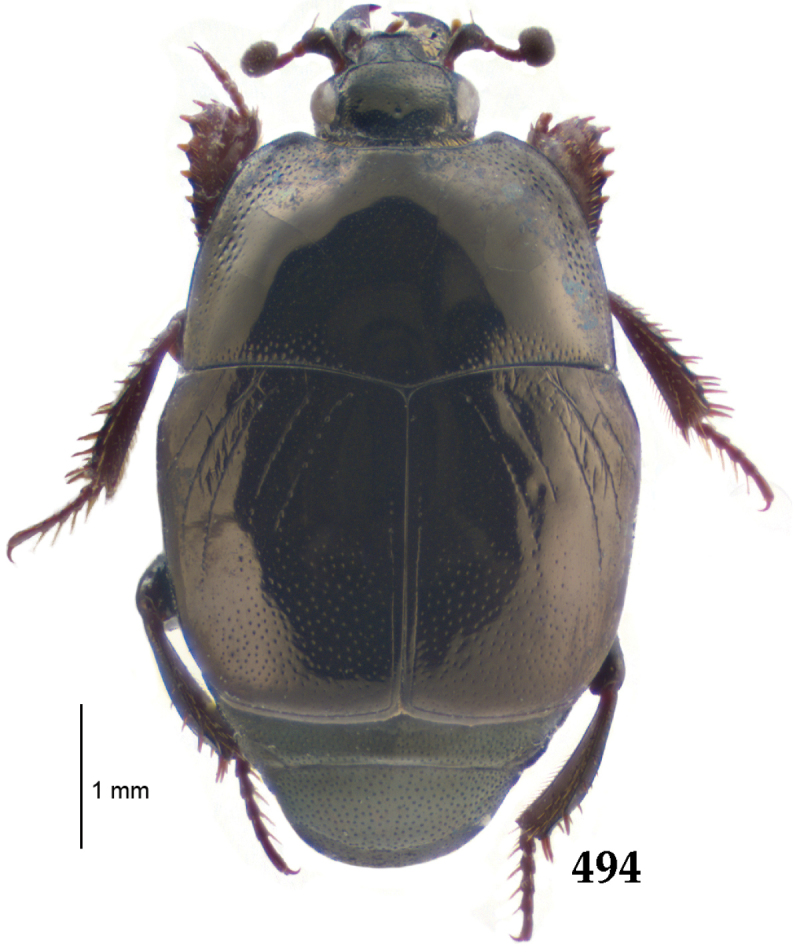
Saprinus (Saprinus) pacificus sp. n. habitus, dorsal view.

##### Type material examined.

Holotype, ♂, side-mounted on a triangular card, with propygidium and pygidium detached, glued to the same triangular card as specimen, terminalia also glued to the same triangular card as specimen, with the following labels: “KIRIBATI / Bikenibeu / N. 1977 / P.J. Simmonds” (written); followed by: “Assoc. with / poultry / dung” (written); followed by: “SAPRINUS / sp. 2 / Det. T. Lackner 2009” (printed-written); followed by: “*Saprinus* / *nitiduloides* / Det. S. Mazur” (printed-written); followed by: “09-078” (yellow label, pencil-written); followed by: “Saprinus (Saprinus) / *pacificus* sp. n. / HOLOTYPE / Lackner&Leschen 2010” (red label, printed) (NZAC). Paratypes, 1 ♂ & 4 specs., with the following labels: “KIRIBATI / Bikenibeu / N. 1977 / P.J. Simmonds” (written); followed by: “Assoc. with / poultry / dung” (written); followed by: “Saprinus (Saprinus) / *pacificus* sp. n./ PARATYPE / Lackner&Leschen 2010” (red label, printed) (NZAC; 1 PT in coll. TLAN).

##### Distribution.

Kiribati atoll (Fig. 751).

##### Biology.

Collected in association with poultry dung.

##### Remarks.

Because the following newly described species, *S. pacificus* sp. n., is in its general appearance rather similar to the preceding species *S. nitiduloides* we provide it with only diagnostic description. The figures, as well as male genitalia drawings are kept, for the sake of easier identification of the Australopacific taxa.

##### Diagnostic description.

**Figures 495–503.**
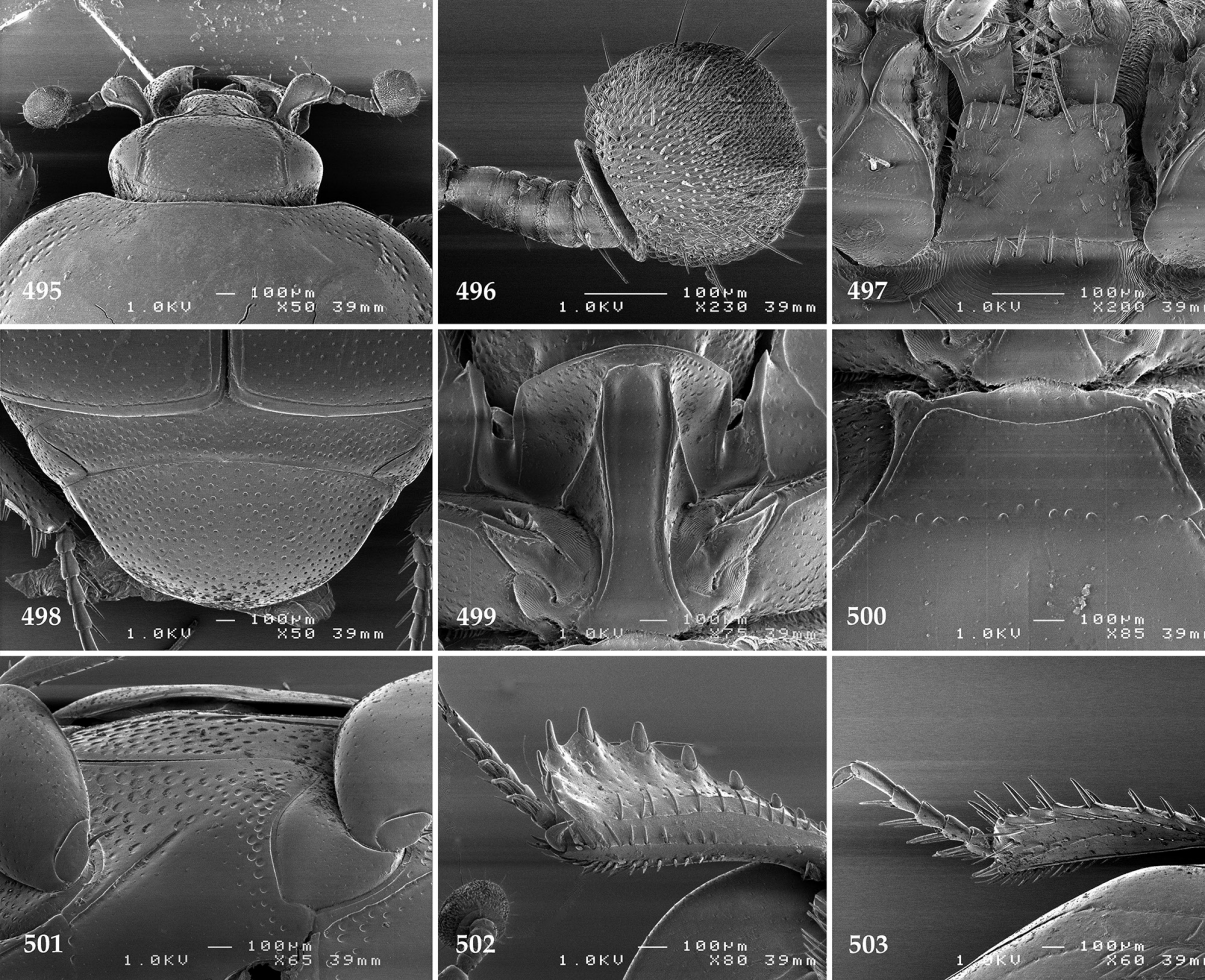
**495** Saprinus (Saprinus) pacificus sp. n. head, dorsal view **496** antennal club, dorsal view **497** mentum, ventral view **498** propygidium + pygidium **499** prosternum **500** mesoventrite **501** lateral disc of metaventrite + metepisternum **502** protibia, ventral view **503** mesotibia, dorsal view.

This species is in its general outlook rather similar to *S. nitiduloides* (compare Figs 477 and 494); body length: PEL: 3.25–4.00 mm; EL: 2.00–2.35 mm; APW: 1.15–1.40 mm; PPW: 2.40–3.00 mm; EW: 2.75–3.25 mm. Antennae (Fig. 495–496) similar to other congeners, e.g. *S. cyaneus* (Fig. 409) or *S. nitiduloides* (Fig. 478).

**Figures 504–510.**
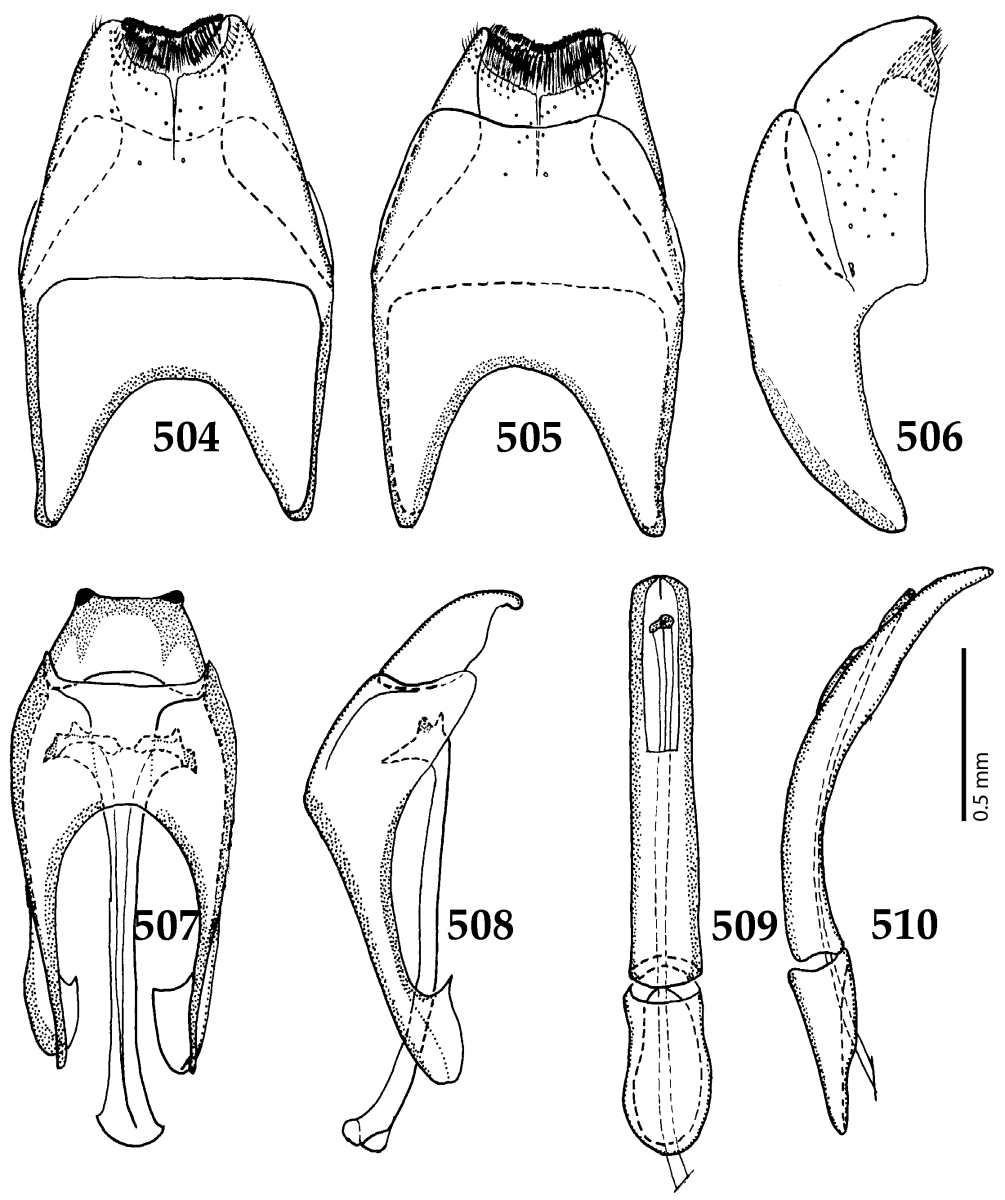
**504** Saprinus (Saprinus) pacificus sp. n. male terminalia: 8^th^ sternite + 8^th^ tergite, ventral view **505** ditto, dorsal view **506** ditto, lateral view **507** male terminalia: 9^th^ + 10^th^ tergites, dorsal view; spiculum gastrale, ventral view **508** male terminalia: 9^th^ + 10^th^ tergites; spiculum gastrale, lateral view **509** male terminalia: aedeagus, dorsal view **510** ditto, lateral view.

Mouthparts similar to congeners; mentum (Fig. 497) with deep median notch on anterior margin, surface around it with two long setae and several much shorter setae, lateral margins with two rows of short ramose setae, disc of mentum finely imbricate, with sparse short setae. Clypeus and frons (Fig. 495) similar to those of *S. nitiduloides* (compare Fig. 478 with Fig. 495); frontal stria prolonged onto clypeus and also almost complete along anterior frontal margin (occasionally intermittent). Pronotal sides moderately narrowing anteriorly, pronotal depressions deeper and apical angles more prominent than with *S. nitiduloides* (compare Figs 477 and 494). Elytral structure and configuration of elytral striae similar to those of *S. nitiduloides*, but punctation even sparser, punctures separated by several times their diameter (compare Figs 477 and 494). Propygidium and pygidium very similar between the two species (compare Figs 479 and 498). Prosternum structurally similar to that of *S. nitiduloides*, but the course of carinal prosternal striae differ between the two species: while sub-parallel and slightly narrowing before apex in *S. nitiduloides* (Fig. 480) they are slightly widening anteriorly with *S. pacificus* (Fig. 499). Mesoventrites similar between the two species, but that of *S. pacificus* slightly wider than that of *S. nitiduloides* and its punctation finer; furthermore the lateral mesoventral stria almost complete with *S. pacificus* while shortened apically with *S. nitiduloides* (compare Figs 481 and 500). Metaventrites and metepisternal similar between the two species (compare Figs 482 and 501). Protibia (Fig. 502) similar to that of *S. nitiduloides* (Figs 483–484); mesotibia (Fig. 503) and metatibia similar to other congeners (e.g. *S. artensis*). Male genitalia. Eighth sternite (Figs 504–505) with pseudo-pores, weakly separated on apical third, otherwise fused, apex with velum adorned with dense rows of brush-like setae; laterally apex of eighth sternite with a single row of microscopic sparse setae; eighth tergite and eighth sternite fused laterally (Fig. 506). Ninth tergite (Figs 507–508) typical for the subfamily; anterior margin of tenth tergite straight, apical angles strongly sclerotized, bent; spiculum gastrale (Fig. 507) abruptly dilated on apical third; apical end strongly sclerotized, with protruding horn-like structures and tiny median notch; basal end only slightly dilated, outwardly arcuate. Aedeagus (Figs 509–510) parallel-sided, with parameres fused along their basal half (roughly); basal piece of aedeagus short, ratio of its length: length of parameres 1 : 4; aedeagus slightly curved from lateral view (Fig. 510). Male terminalia of *S. pacificus* sp. n. are similar to those of *S. artensis*, *S. cyaneus cyaneus*, and *S. nitiduloides* especially regarding strongly sclerotized apices of tenth tergite. Although the structure of spiculum gastrale or ninth tergite is also similar between the four species, they differ in the structure of eighth tergite. Their aedeagi, on the other hand, are strikingly similar indicating their recent common ancestry (compare Figs 330–336; 415–412; 487–493; 504–510).

#### Saprinus (Saprinus) pseudodetritus sp. n.

Taxon classificationAnimaliaColeopteraHisteridae

http://zoobank.org/C235FE0D-3EDB-4069-AA82-879D98FD2E88

Figs 511 , 512–520 , 521–527 , 761

##### Type locality.

New Zealand: Chatham Islands: South East Island.

**Figure 511.**
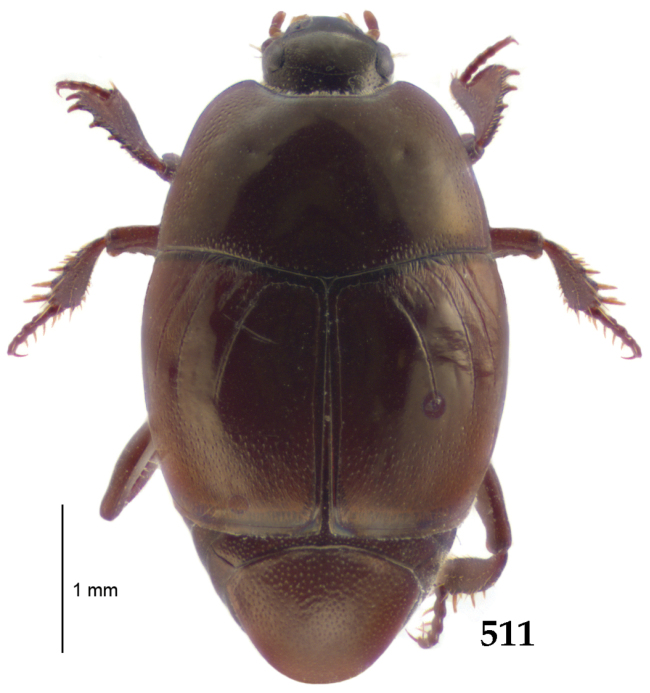
Saprinus (Saprinus) pseudodetritus sp. n. habitus, dorsal view.

##### Type material examined.

Holotype, ♂, side-mounted on a triangular card, with terminalia extracted and mounted in Canada Balsam on a separate slide under the specimen, with following labels: “Chatham Is / N. 1970 / J.I. Townsend” (printed); followed by: “South East Island” (printed); followed by: “At night in bush” (printed); followed by: “09-082” (yellow, pencil-written label); followed by: “Saprinus (Saprinus) / *pseudodetritus* sp. n. / HOLOTYPE / Lackner&Leschen 2010” (red label, printed) (NZAC). Paratypes, 9 specs., same data as holotype, except for yellow label, that is present only on holotype (NZAC); 1 spec., “CHATHAM IS, N.Z. / Rangatira I / (South East I) / 1-14 Dec 1987 / J.S. Dugdale” (printed); followed by: “litter / under / Myosotidium” (printed-written) (NZAC); 1 spec., “CHATHAM IS, N.Z. / SE Island 10m / Northern Olearia / forest” (printed-written) (NZAC); followed by: “Fit trap 2 / Predominantly / Plagianthus / forest” (written) (NZAC); 1 spec., “CHATHAM IS, N.Z. / South East I / 13-16 Dec 1987 / A. Grant (DOC)” (printed) (NZAC); followed by: “Nest of / Pachyptila / vittata” (printed) (NZAC; 3 PT from SE Island collected by J.I. Townsend in coll. TLAN); 1 spec., “CHATHAM IS. N.Z. / South East I, 10-20m / 29.xi.1992 / J.W. Early” (printed); followed by: “On tree trunks at / night” (printed); followed by: “**AMNZ 11324** / AUCKLAND / MUSEUM / NEW ZEALAND” (green label, printed) (AMNZ); 6 specs., ditto as above, but 27-28.xi.1992 and collected on bush floor at night (AMNZ). These paratypes bear specimen registration numbers (AMNZ numbers) as follows: 11325, 11326, 11327, 11328, 11329 and 11330; 4 specs., “NEW ZEALAND, CI / South East Island / 14.v.1996 P. Dilks / ex Chatham Island petrel / burrow litter” (printed); followed by: “ENTOMOLOGY / RESEARCH MUSEUM / (LUNZ) / Lincoln University / Canterbury, New Zealand” (green label, printed) (LUNZ); 2 specs., “NEW ZEALAND, CI / Rangatira / 27.xi.1992 / J.W.M. Marris” (printed); followed by: “on ground at base of tree / at night” (printed) (LUNZ); 9 specs., ditto, but 27–30.xi.1992, J.W. Early, R.M. Emberson & P. Syrett (pitfall traps in Olearia/Plagianthus/Macropiper forest) (LUNZ); 3 specs., “NEW ZEALAND, CH / South East Island / Woolshed Bush / 20.i. 1998 R.M. Emberson / on ground at night” (printed) (LUNZ); 1 spec., NEW ZEALAND, CH / South East Island / 31.xii. 1998 R.M. Emberson / under dead broad billed / prions” (printed) (LUNZ); 1 spec., ditto, but on tree trunks at night (LUNZ); 4 specs., “NEW ZEALAND, CI / South East Island / Woolshed Bush / 15.i.1997, R.M. Emberson / on ground by petrel burrows” (printed) (LUNZ); 4 specs., “NEW ZEALAND, CH / Chatham Is, South East I, / Woolshed Bush / 19.–21.i.1998 / R.M. Emberson, J.W. Marris” (printed); followed by: “pitfall traps in coastal / broadleaved forest” (printed) (LUNZ); 4 specs., ditto, but 13.–17.i.1997 (LUNZ); 8 specs., “NEW ZEALAND, CI / Mangere I., Robin Bush / 30.xi.–3.xii. 1992 / J.W.M. Marris & J.W. Early / pitfall trap in bush” (printed) (LUNZ); 6 specs., “NEW ZEALAND, CH / Star Keys / 23.i.1998 J.W.M. Marris / under rock in low coastal vegetation (sward)” (printed) (LUNZ); 5 specs., “NEW ZEALAND, CI / Rangatira / 28.xi.1992 / P. Syrett / by petrel burrow at night” (printed) (LUNZ); 1 spec., ditto, but 29.i.1998 and on tree at night (LUNZ); 1 spec., “NEW ZEALAND, CI / Glory Scan Res / 30.xi.1992 / R.M. Emberson & P. Syrett” (printed) (LUNZ); 1 spec., “NEW ZEALAND, CI / Chatham I, Hapaupu / 2.xii.1992 / P. Syrett / on trees at night (printed) (LUNZ; 8 exs. in coll. TLAN); 1 spec., “Chathm. / gr. ? (illegible)” (written); “New Zealand” (printed); “Pascoe / Coll. / 93-60” (printed); “this specimen is / most likely from / the Chatham Islands / T. Lackner, 2014” (written); “Saprinus pseudodetritus / sp. n. PARATYPE / Det. Lackner & Leschen, 2014” (red label, written) (BMNH).

##### Distribution.

New Zealand: Chatham Islands: South East Island, Mangere Island and Pitt Island (Fig. 761).

##### Biology.

Specimens of this species were found in litter under *Myosotidium* (Boraginaceae), and in *Olearea* (Asteraceae) and *Plagianthus* (Malvaceae) forests, in the nests and on dead Broad-billed prion (*Pachyptila vittata* (G. Forster, 1777)) and near petrel burrows. The species is also nocturnal, and has been found on tree trunks and on the bush floor at night.

##### Diagnosis.

This is a distinctive New Zeland endemic (so far only collected on Chatham Islands) differing from other two, presumably closely related New Zealand species by matte dorsum covered with alutaceous microsculpture, a lightly colored antennal club, aciculate bases of elytral striae, as well as scratched-like punctation on apical third of elytra.

##### Diagnostic description.

**Figures 512–520.**
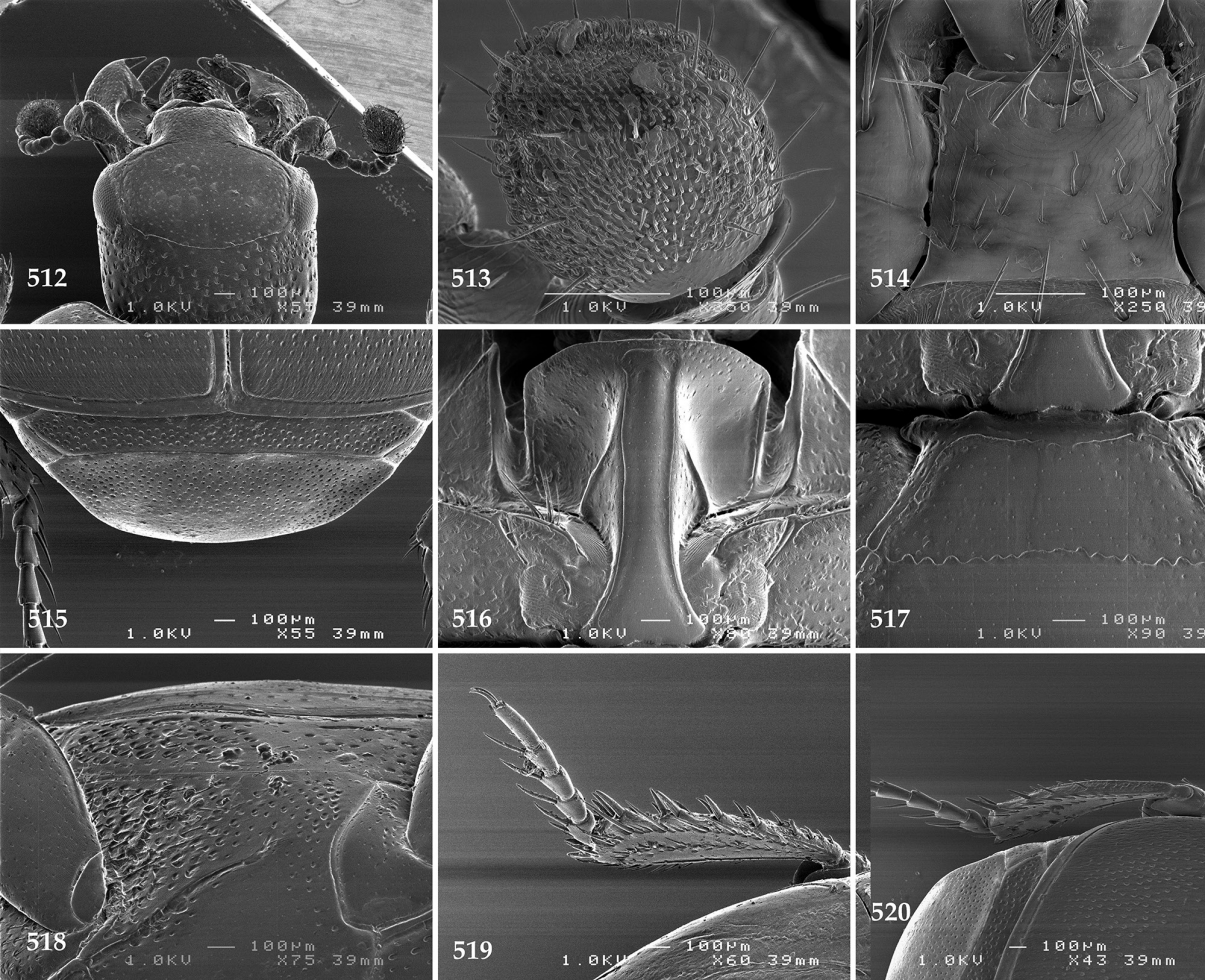
**512** Saprinus (Saprinus) pseudodetritus sp. n. head, dorsal view **513** antennal club, ventral view **514** mentum, ventral view **515** propygidium + pygidium **516** prosternum **517** mesoventrite **518** lateral disc of metaventrite + metepisternum **519** mesotibia, dorsal view **520** metatibia, dorsal view.

Since *S. pseudodetritus* is rather similar to *S. detritus* and *S. chathamensis* we will provide only its diagnostic description here mostly outlining the chief differences between the three taxa. The figures, as well as male genitalia drawings are kept, for the sake of easier identification of the Australopacific taxa. The same approach is taken with the species *S. chathamensis* (see above). On the other hand, the species *S. detritus*, which was described originally as the first of the three New Zealand species, is provided with full detailed description. A light to dark brown species with matte bronze luster (opaque cuticle is due to presence of fine imbricate microsculpture, which can be worn in some specimens, especially on elytra); venter black, without imbricate microsculpture; legs, mouthparts and antennae rufous to rufo-castaneous; antennal club amber-colored to rufous. Body length: PEL: 3.25–4.25 mm; EL: 1.90–2.50 mm; APW: 1.20–1.50 mm; PPW: 2.40–3.10 mm; EW: 2.65–3.50 mm. Antennae (Figs 512–513) similar in structure to other two New Zealand endemics; sensory structures of antennal club not examined.

Mouthparts similar to those of *S. chathamensis* and *S. detritus*; mentum (Fig. 514) quadrate, anterior and posterior angles slightly projected, anterior margin with deep median notch, surface around it with several longer setae, lateral margins apically with a single row of much shorter ramose setae, disc of mentum finely imbricate, with sparse short setae (similar to that of *S. detritus*; Fig. 425). Clypeus and frons (Fig. 512) rather similar to those of *S. chathamensis* and *S. detritus* (compare with Figs 373 and 423). Pronotal sides (Fig. 511) on basal two-thirds moderately narrowing anteriorly, on apical third strongly narrowed, apical angles prominent. Generally, the pronotum of *S. pseudodetritus* is apically more narrowed than those of *S. chathamensis* and S. detritus (compare also Figs 372 and 422). Lateral band of punctures present, punctures not prominent, fine. Elytral surface around bases of striae aciculate; elytral disc on apical third (roughly) with elongate punctures separated by their own to several times their diameter. Configuration of the elytral striae very similar otherwise between species *S. chathamensis*, *S. detritus* and *S. pseudodetritus* (compare Figs 372, 422 and 511) with characteristically strongly shortened third dorsal elytral stria. Although elytral sculpture is also similar between species, the punctation of *S. pseudodetritus* is the finest of the three species; interspaces between punctures in *S. pseudodetritus* strongly aciculate. Propygidium and pygidium (Fig. 515) with fine small round punctures separated by about their diameter; interspaces in both cases imbricate. Prosternum generally similar to those of other two NZ species (compare Figs 377 with 426 and 516) antennal cavities for reposing antennal clubs large; carinal prosternal striae of *S. pseudodetritus* sub-parallel, while they are narrowing apically in *S. chathamensis* and slightly diverging apically in *S. detritus*.

Meso-and metaventrites (Fig. 517–518) similar to those of other two species, for direct comparison between the three species the reader is referred to the diagnostic description of *S. chathamensis*. Legs of *S. pseudodetritus* are generally similar to those of *S. chathamensis* and *S. detritus*.

**Figures 521–527.**
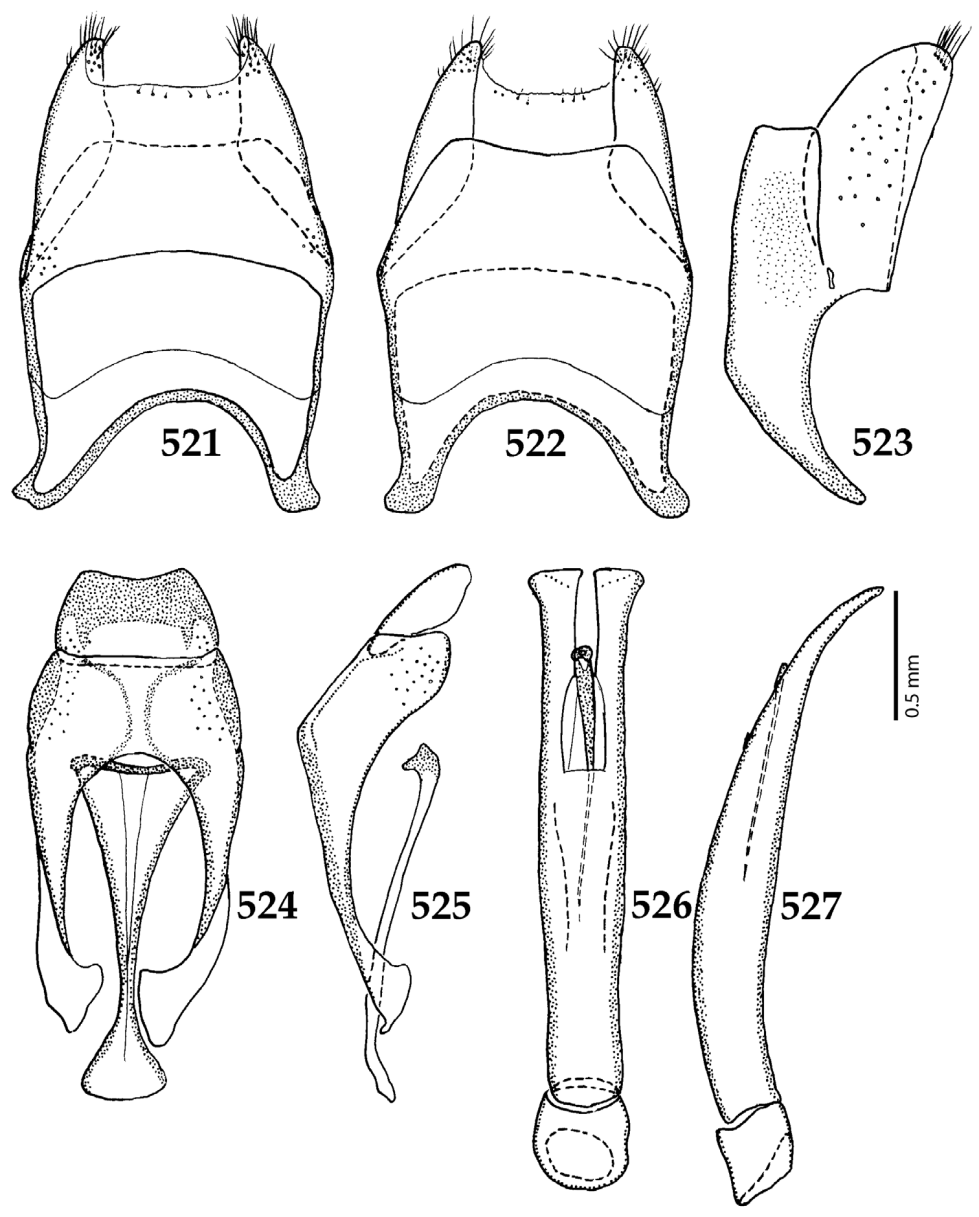
**521** Saprinus (Saprinus) pseudodetritus sp. n. male terminalia: 8^th^ sternite + 8^th^ tergite, ventral view **522** ditto, dorsal view **523** ditto, lateral view **524** male terminalia: 9^th^ + 10^th^ tergites, dorsal view; spiculum gastrale, ventral view **525** male terminalia: 9^th^ + 10^th^ tergites; spiculum gastrale, lateral view **526** male terminalia: aedeagus, dorsal view **527** ditto, lateral view.

Male genitalia. Eighth sternite (Figs 521–522) apically with several pseudo-pores, entirely fused, strongly sclerotized; apex of eighth sternite laterally with two tufts of several short setae; eighth tergite and eighth sternite fused laterally (Fig. 523). Ninth tergite (Figs 524–525) typical for the subfamily; anterior margin of tenth tergite inwardly arcuate; spiculum gastrale (Fig. 524) gradually dilated on apical half; apical end strongly sclerotized; basal end abruptly dilated, triangular, basal end outwardly arcuate. Aedeagus (Figs 526–527) subparallel, before apex slightly dilated; parameres fused along their basal half (roughly); basal piece of aedeagus short, ratio of its length : length of parameres 1 : 6; aedeagus almost straight, only apically slightly curved from lateral view (Fig. 527). The male terminalia of *S. detritus* and *S. pseudodetritus* are very similar (compare Figs 432–438 with 521–527) and generally differ only in the structure of aedeagus, which is slightly constricted before apex in *S. detritus* while it is not constricted and tube-like in *S. pseudodetritus*. In lateral view, aedeadus of *S. pseudodetritus* is thicker than that of *S. detritus* (compare Figs 438 and 527).

#### Saprinus (Saprinus) rarus sp. n.

Taxon classificationAnimaliaColeopteraHisteridae

http://zoobank.org/7DF7647D-3AE5-4071-A734-C9C4EAB9920F

Figs 528 , 529–537 , 538–546 , 764

##### Type locality.

Australia: New South Wales: N of Sydney: Pearl Beach at Broken Bay.

**Figure 528.**
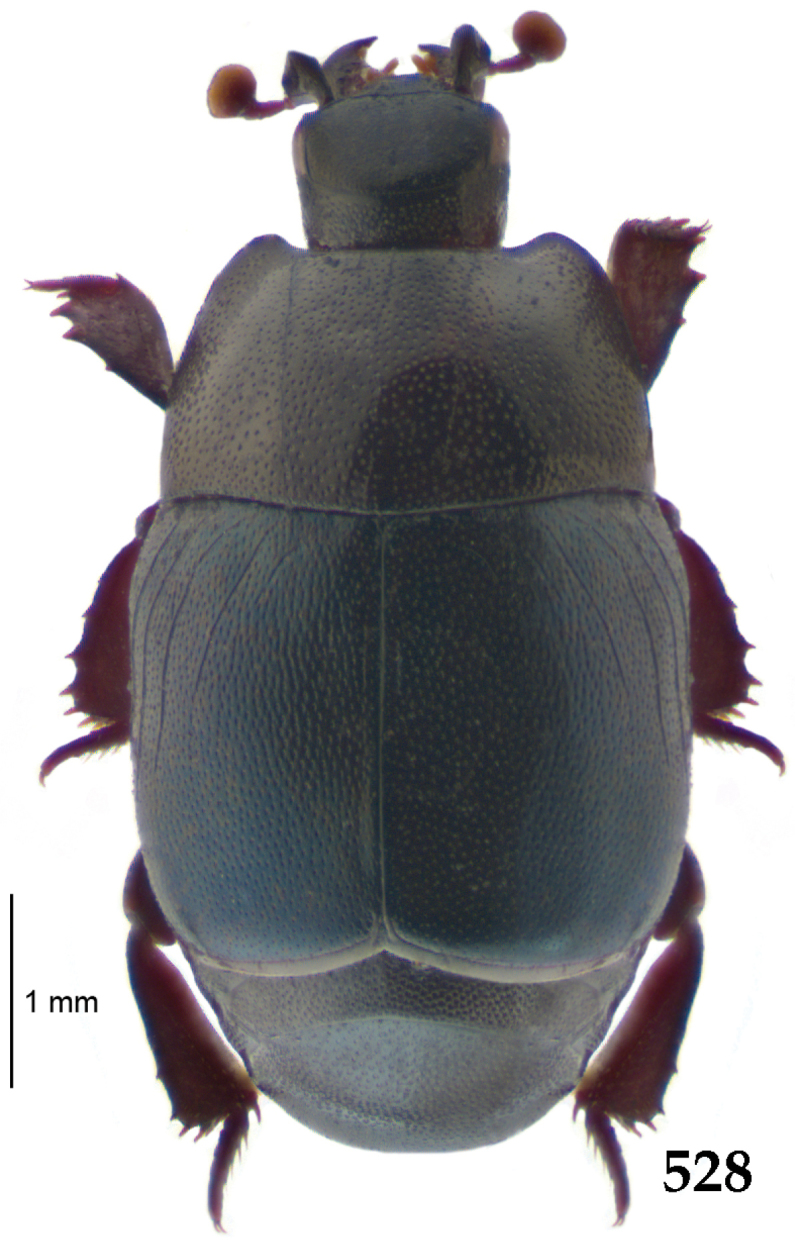
Saprinus (Saprinus) rarus sp. n. habitus, dorsal view.

##### Type material examined.

Holotype, ♂, side-mounted on a triangular card, right metatibia broken off, glued to the same triangular card as the specimen, with male genitalia dismembered, glued to the same mounting card as the specimen, with the following labels: “Australia, / N of Sydney, / Pearl Beach at Broken Bay / 7.iii.1997 / leg. D. Scherbakov” (printed); with a consecutive label: “Coll. of / A. Sokolov” (hand-written); with consecutive label: “Saprinus (s.str.) rarus / n. spec. HOLOTYPE / Det. T. Lackner & R. / Leschen 2013” (red label, hand-written) (MAMU).

Paratype, ♀, side-mounted on a triangular card, right mesotarsus broken off, glued to the same triangular card as the specimen with the following labels: “Blackdown / T’ l Q. / 14.5. [19]81 / N.W. Rodd” (printed-written); followed by: “♀” (beige label, printed); followed by: “Australian Museum / K 270235” (printed); followed by: “*Saprinus* (s.l.) / sp. / G. Arriagada det. 1990” (black-framed label, printed-written); “followed by: “10-115” (light-yellow pencil written label); followed by: “Saprinus (s.str.) rarus / n. spec. PARATYPE / Det. T. Lackner & R. / Leschen 2013” (red label, hand-written); followed by: “Photographed by / B. Rhode” (yellow label, printed) (MAMU); paratype, ♂, mounted on a mounting card, with the following labels: “33°36'07"S; 151°19'17"E / AUSTRALIA: New South Wales, Palm Beach, 15. / VIII.1962, G.R.Wearne / Host: *Nasutitermes walkeri* (Hill)” (printed); followed by: “Australia: N.S.W / Palm Beach, / 15.viii.1962” (printed-written); followed by: “Coll., Host det. / G.R. Wearne” (written); followed by: “Host: / Nasutitermes / walkeri” (written); followed by: “Saprinus rarus / sp.n. PARATYPE / det. T. Lackner 2016” (red label, written) (CPK).

##### Biology.

Collected from the nest of the arboreal Tree termite (*Nasutitermes walkeri* (Hill, 1942)). Based on the morphology (thickened and dilated antennal scape, tibiae) and collection circumstances, *Saprinus rarus* is presumed to be a specialized termitophile. This is the first record of a termitophilic Saprininae from the Australopacific Region and only the third case of termitophily in the subfamily in general (the two other taxa are: African *Pilisaprinus verschureni* (Thérond, 1959) ihabiting dead termitaria of the genus *Macrotermes* (Termitidae) recorded from Congo, Ivory Coast and Benin and *Nannolepidius braunsi* (Bickhardt, 1921) found in nests of *Hodotermes* termites (Hodotermitidae) in the Cape Region of South Africa, respectively).

##### Distribution.

This species is known only from three Australian specimens: two males collected near Sydney (New South Wales) and a female collected in Blackdown Tableland National Park, near Rockhampton (Queensland) (Fig. 764).

##### Etymology.

The specific epithet ‘*rarus*’ refers to the scarcity of this beetle in collections.

##### Diagnosis.

**Figures 529–537.**
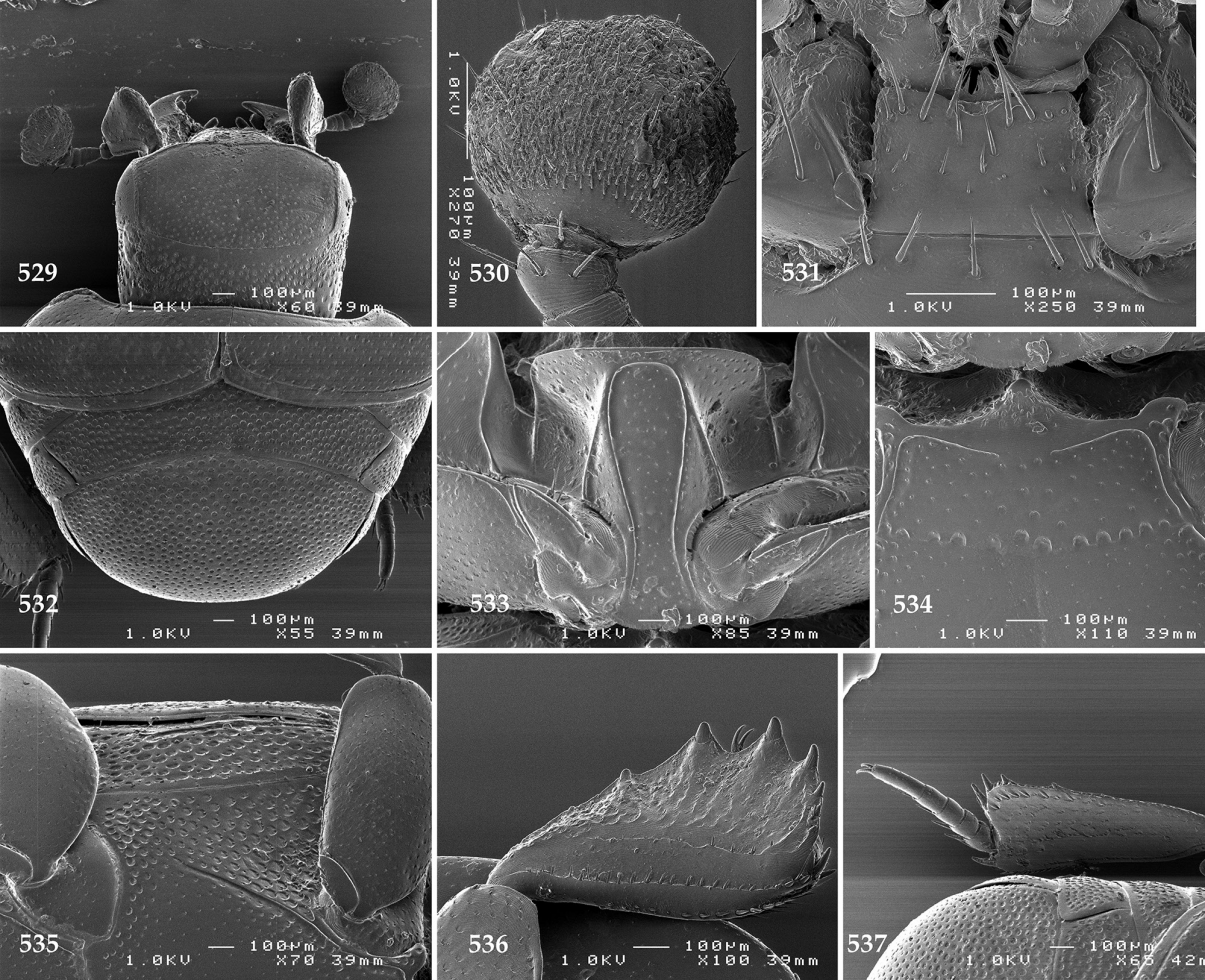
**529** Saprinus (Saprinus) rarus sp. n. head, dorsal view **530** antennal club, ventral view **531** mentum, ventral view **532** propygidium + pygidium **533** prosternum **534** mesoventrite **535** lateral disc of metaventrite + metepisternum **536** protibia, ventral view **537** metatibia, dorsal view.

*Saprinus rarus* has fused parameres (Fig. 545); its elongate body (Fig. 528), dilated tibiae (Figs 536–537), thickened and dilated antennal scape (Fig. 529) and large, almost circular antennal clubs (Fig. 530) will differentiate this species from other species in the genus.

##### Description.

Body length: PEL: 3.50 mm; APW: 1.50 mm; PPW: 2.50 mm; EL: 2.25 mm; EW: 2.90 mm (only one specimen was measured). Body (Fig. 528) elongate, convex, pronotum distinctly narrower than elytra, pronotum piceous, shining, elytra dark blue with metallic tinge; legs, mouthparts and antennal scape+funicle chestnut brown, antennal club lighter, amber colored, becoming progressively lighter apically.

Antennal scape (Fig. 529) strongly triangularly dilated, thickened, punctate, almost asetose (setae worn off?); antennal club (Fig. 530) large, almost circular, slightly depressed dorso-ventrally, entirely covered in dense short sensilla in the female paratype, while in the male holotype the lower third of the antennal club asetose, intermingled with sparse longer erect sensilla; sensory structures of antennal club not examined.

Mandibles with rounded outer margin, acutely pointed, sub-apical tooth on inner margin of left mandible not examined; labrum slightly depressed medially, labral setae not examined (worn off?); terminal labial palpomere elongated, its width about one-third its length; mentum (Fig. 531) almost square-shaped, anterior margin medially with prominent notch, surface around it with four long setae, anterior angles each with two shorter setae; lateral margins with a single row of even shorter sparse ramose setae, disc on apical half (roughly) with few scattered setae of various sizes (none of these setae are as long as those in the anterior corners or near median notch), basal half (roughly) asetose; terminal maxillary palpomere elongated, its width about one-third half its length, other mouthparts not examined.

Clypeus (Fig. 529) rectangular, flattened, with slight median depression, rounded laterally, anterior angles slightly convex, with dense shallow fine punctures; frontal stria (Fig. 529) complete; frontal disc (Fig. 529) anteriorly flattened, entirely punctate, punctures shallow, separated by about their own to twice their diameter, postero-median part of frontal disc with distinct fovea; eyes flattened, visible from above.

Pronotal sides (Fig. 528) widest on basal third, gradually convergent apically, apical angles prominent, marginal pronotal stria complete, carinate laterally, weakened behind head; disc almost entirely punctate, punctures round, deep, medially separated by their own to several times their diameter, laterally creating a band of denser and coarser punctation, between it and lateral pronotal margin a narrow impunctate band present; pronotal hypomeron glabrous; scutellum very small.

Elytral epipleura finely punctate; marginal epipleural stria thin; marginal elytral stria well impressed, thin, continuous along elytral apex as apical elytral stria; humeral elytral stria joined with inner subhumeral stria creating thus a complimentary dorsal elytral stria; four dorsal elytral striae 1–4 present, first the longest, slightly surpassing elytral half, second and third each slightly shorter, fourth stria the shortest, present as a short fragment on basal elytral sixth; sutural elytral stria abbreviated basally, in the female paratype present as a short fragment on (roughly) basal elytral third; in the male holotype entirely missing. Entire elytral disc very coarsely and densely punctate, punctures separated approximately by their diameter, between them another kind of much finer sparser punctures present, interspaces between punctures imbricate; before apical elytral stria punctation weakens, becomes much finer and sparser.

Propygidium (Fig. 532) almost completely exposed, covered with dense but shallow punctures separated by less than half their own diameter; punctation of pygidium (Fig. 532) sparser, but still very dense, punctures separated by less than their diameter, becoming sparser and finer near pygidial apex, interspaces imbricate.

Anterior margin of median portion of prosternum (Fig. 533) straight; marginal prosternal stria inconspicuous; prosternal process between carinal prosternal striae flattened, in sparse fine punctures, laterally with sparse small oval punctures, interspaces imbricate; carinal prosternal striae (Fig. 533) carinate, slightly divergent anteriorly and united in front under a rounded loop; lateral prosternal striae carinate, convergent anteriorly, attaining prosternal process near united apices of carinal prosternal striae.

Discal marginal mesoventral stria laterally well impressed, medially interrupted (Fig. 534); disc with sparse punctures of various sizes separated by several times their diameter; meso-metaventral suture fine; meso-metaventral sutural stria impressed as a row of large shallow punctures; intercoxal disc of metaventrite medially in female with shallow depression, while in the male this depression is deeper; larger punctures appear mostly along median longitudinal line and behind metacoxae, rest of metaventrite with sparse microscopic punctation. Lateral metaventral stria (Fig. 535) well impressed, almost straight, ending short of metacoxa; lateral disc of metaventrite (Fig. 535) depressed, with round shallow large punctures; metepisternum evenly punctate with even denser punctation; lateral metepisternal stria (Fig. 535) present, deeply impressed, absent on fused metepimeron.

Intercoxal disc of first abdominal ventrite completely striate laterally; surface of disc with scattered punctation, punctures becoming sparser and finer medially.

Protibia (Fig. 536) dilated, outer margin with three short triangular teeth topped by short denticle, followed by another short denticle; setae of outer row very short, sparsely paced; setae of median row inconspicuous; protarsal groove very shallow, almost non-existent; protibial spur tiny, growing out from anterior protibial margin; anterior protibial margin ventrally with three tiny denticles; anterior protibial stria complete, very fine; outer part of posterior surface of protibia (Fig. 536) knobby; median part of posterior surface glabrous, separated from outer part by a definite fine stria with minuscule setae; posterior protibial stria complete, with regular minuscule setae turning into two minuscule denticles apically; inner margin with single row of sparse microscopic setae.

Mesotibia dilated, outer margin with 5 widely spaced short denticles growing in size apically; setae of outer row sparse, minuscule; setae of median row inconspicuous; posterior mesotibial stria not examined; anterior surface of mesotibia convex medially; anterior mesotibial stria complete; mesotibial spur short; claws of apical tarsomere short, less than half its length; metatibia (Fig. 537) basically similar to mesotibia, but denticles of outer margin even sparser than those of mesotibia and metatibial spur longer.

**Figures 538–546.**
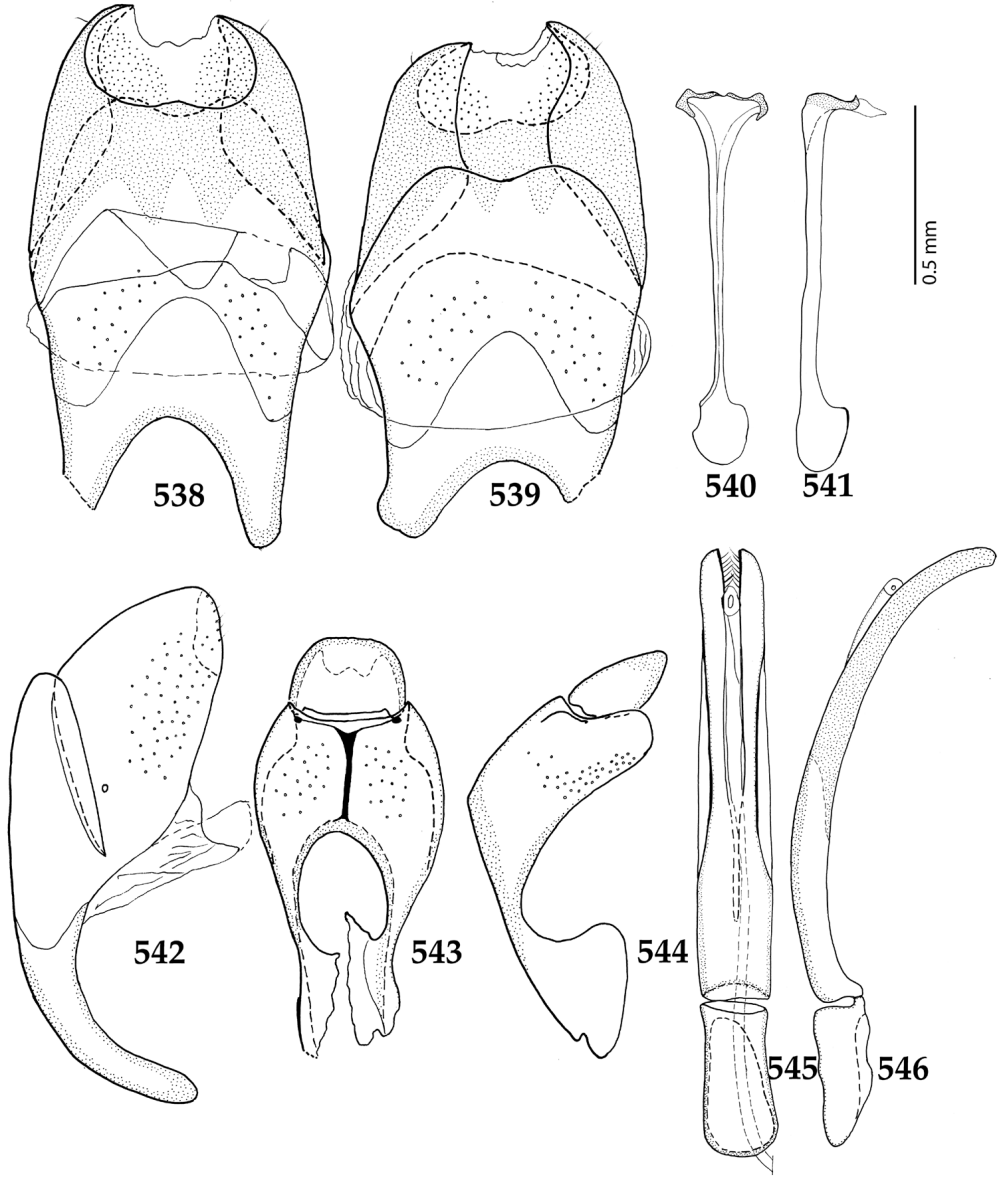
**538** Saprinus (Saprinus) rarus sp. n. male terminalia: 8^th^ sternite + 8^th^ tergite, ventral view **539** ditto, dorsal view **540** male terminalia: spiculum gastrale, ventral view **541** ditto, lateral view **542** male terminalia: 8^th^ sternite + 8^th^ tergite, lateral view **543** male terminalia: 9^th^ + 10^th^ tergite, dorsal view **544** ditto, lateral view **545** male terminalia: aedeagus, dorsal view **546** ditto, lateral view.

Male genitalia. Eighth sternite (Figs 538–539) completely fused medially, apically with asetose velum covered with pseudo-pores, apex of 8^th^ sternite with several short setae. Eighth tergite (Fig. 539) basally with deep emargination, apex only slightly emarginate; 8^th^ sternite and tergite fused (Fig. 542). Ninth tergite (Figs 543–544) medially with strong sclerotization, with pseudo-pores. Apex of spiculum gastrale (Fig. 540) strongly sclerotized, basal end outwardly arcuate. Aedeagus with parameres separated on apical half (Fig. 545), curved, apex of parameres with setae (Fig. 546).

#### Saprinus (Saprinus) splendens

Taxon classificationAnimaliaColeopteraHisteridae

(Paykull, 1811)

Figs 547 , 548–556 , 557–564 , 755 , 764

Hister splendens Paykull, 1811: 53.

##### Type locality.

Republic of South Africa: Capeland.

**Figure 547.**
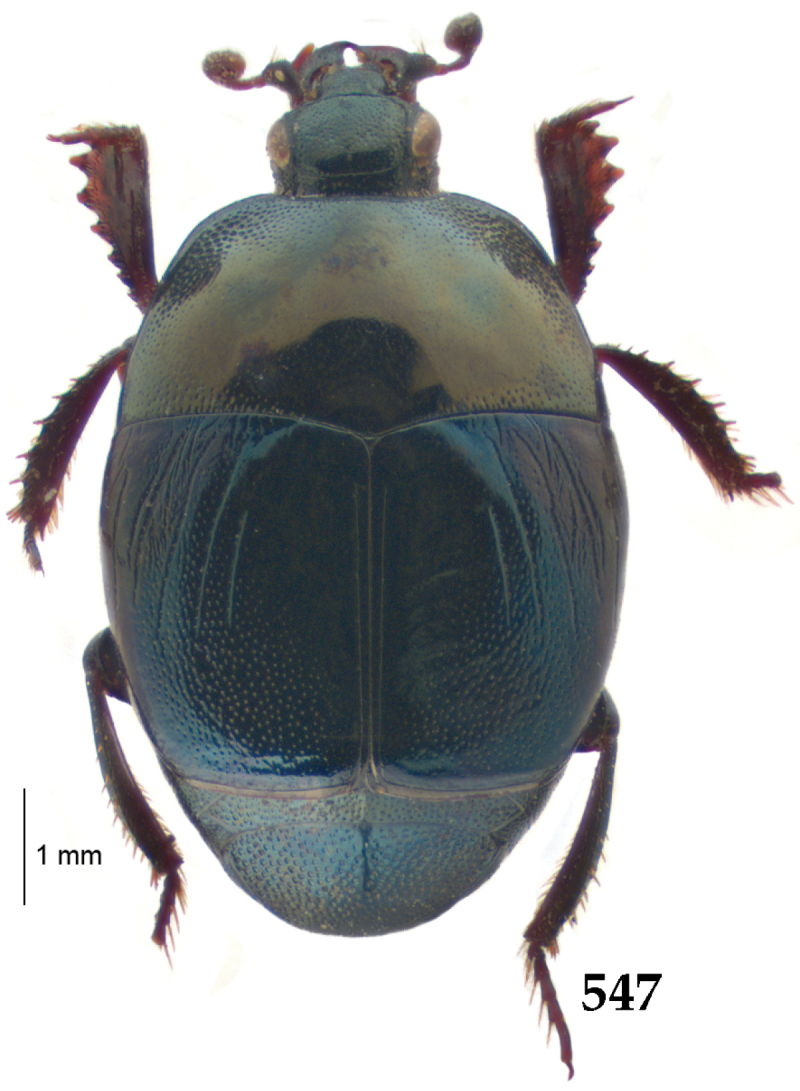
Saprinus (Saprinus) splendens (Paykull, 1811) habitus, dorsal view.

##### Type material examined.

*Hister splendens* Paykull, 1811: Lectotype, designated by T. Théry in 2007, ♀, pinned, left mesotarsus broken off, with the following labels: “♀” (small rectangular label, pencil-written); followed by: “8357 / E91 +” (light-blue label, printed); followed by: “LECTOTYPE” (red label, printed); followed by: “*Saprinus* (s.str.) / *splendens* (Paykull, 1811) / T.Thery det. 2007” (printed); followed by: “Naturhistoriska / Riksmuseet / Stockholm / Loan no 1024/06” (green label, printed) (NHRS).

Lackner and Gomy (2016) examined the lectotypes and paralectotypes of the synonymies of this species: *Saprinus speciosus* Erichson, 1834; *Saprinus advena* Marseul, 1855; *Saprinus ovalis* Marseul, 1855 and *Saprinus rasselas* Marseul, 1855.

##### Additional material examined.

AUSTRALIA. Queensland: 1 spec., Bloomf. River (HMNH); 2 specs., Strandbroke Island, 2.iii.1980, G. Daniels (MAMU): 2 specs., 3 km NE Mt.Webb, 15.03S 145.09E, 3.v.1981, J. Feehan (ANIC); 2 specs., 14 km WbyN Hope Vale Mission, 15.16S 144.59E, 7.–10.v.1981, A. Calder (ANIC); 1 spec., White Mountains National Park, “Rugged Gorge” camp, 20.23S 144.47E, 31.iii.–3.iv.2000, T. Weir (on ground at night) (ANIC); 3 specs., Nelly Bay, Magnetic Is., 5.v.1997, S. Feam (under dead cane toad) (QM); 3 specs., Caloundra, 28.x.1913, H. Hacker; 2 ♀♀ & 6 specs., Bangalee Beach, 23°04'S, 150°46'E, 10 m, 16.–19.xii.1999, D. & I. Cook (dung pitfall) (QM); 2 specs., Cairns District, no date, J.A. Anderson; 1 spec., Bamaga, xii.1983, Sedláček (QM); 1 spec., Cape York Peninsula, Lockerbie area, 13.–27.iv.1973, G.B. Monteith (QM); 1 spec., Cape Flattery Heath, 45 km N of Cooktown, 13.–14.vii.1976, G.B. & S.R. Monteith (QM); 1 spec., Moreton Island, Tertiary Dune nr. Blue Lagoon, 4.–11.xii.1988, S. Hamlet (F.I.T.) (QM); 1 spec., Cairns, Illingworth coll., no date (carrion) (BPBM); 2 specs., Bathurst Head, i.1927, Hale & Tindale (SAMA); 2 specs., Bowen, A. Simson (SAMA); 1 spec., Cairns, no further details (SAMA); 1 spec., Bathurst Head, i.1927, Hale & Tindale (SAMA). Western Australia: 8 specs., Kimberley, Carson River National Park, Solea Falls, 14°29'S, 127°00'E, 20.–22.iv.1998, D.J. Cook (pitfall, fish bait) (QM). Northern Territory: 7 specs., Litchfield NP, 40 km E of Daly River, 14.xii.2008, Sváťa Bílý leg. (NMPC; 1 spec. in coll. TLAN); 1 spec., Douglas Hot Springs, 13°45'S, 131°26'E, 12.xii.2008, Sváťa Bílý leg. (NMPC); 2 specs., Darwin, 20.iii.1968, B.P. Moore (ANIC); 1 ♂, Newcastle Waters, 19.vi.2000, dead wallaby, M.A. Hielkema (HMNH); 1 spec., Cape Crawford, 16°41'S, 135°44'E, 18.–20.iv.2004, Monteith & Cook (woodland, grassy, dung trap); 1 spec., Daly River, G.F. Hill (SAMA); 1 spec., ditto, but H. Wesselman (SAMA); 10 specs., Bathurst Island, x.1916, G.F. Hill (SAMA); 3 specs., Melville Island, G. F. Hill (SAMA); 1 spec., Roper River, H.E. Warren (SAMA); 1 spec., Fitzroy and Margaret Rivers, Calvert Expedition (SAMA); 6 specs., Groote Eylandt, N.B. Tindale (SAMA); 1 spec., Darwin (SAMA). Victoria: 1 spec., Victoria, Dr. Müller, no further details (HMNH). Unknown localities: 1 spec., Australia Occid., no further data, 1192 (HMNH).

**Figures 548–556.**
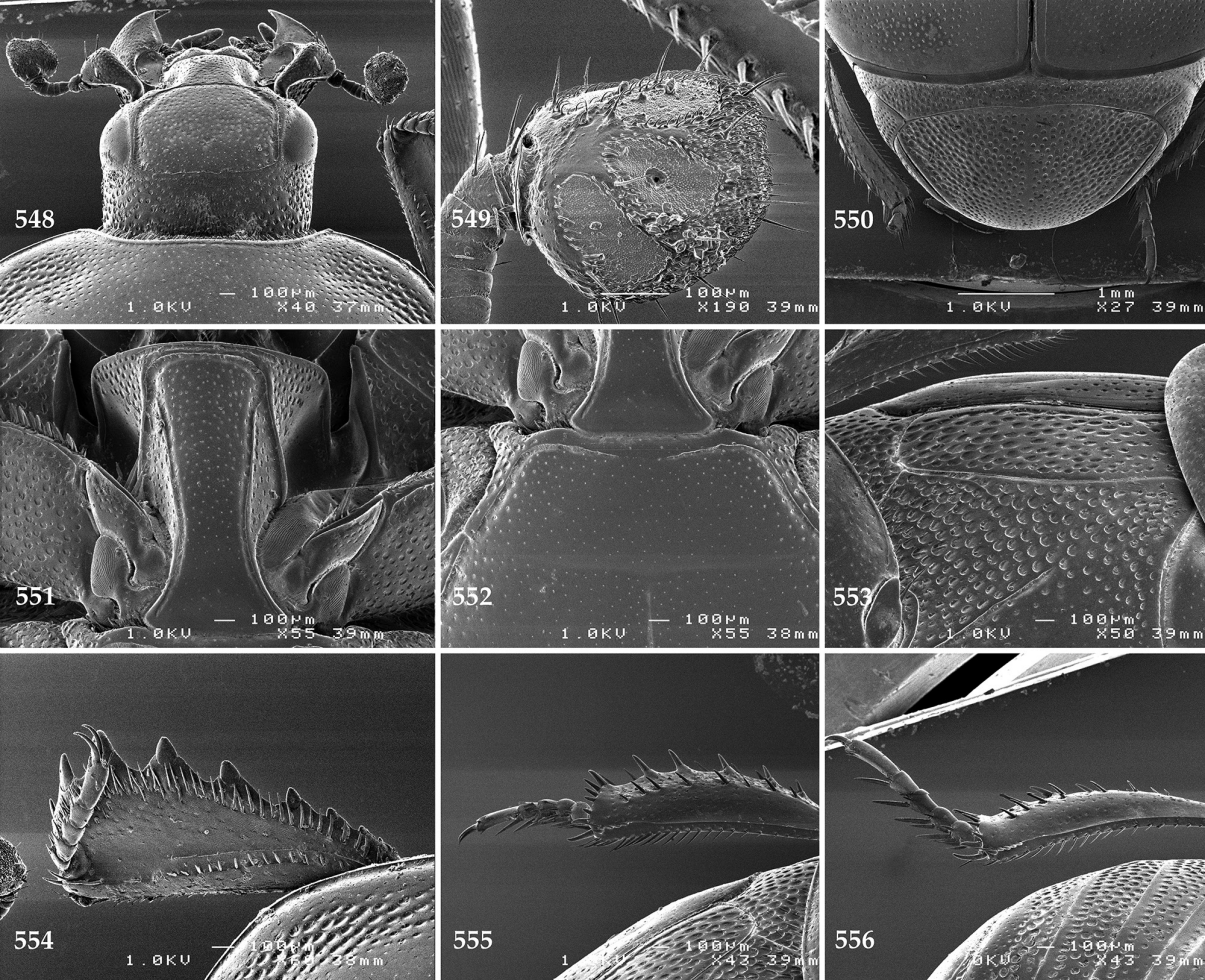
**548** Saprinus (Saprinus) splendens (Paykull, 1811) head, dorsal view **549** antennal club, ventral view **550** propygidium + pygidium **551** prosternum **552** mesoventrite **553** lateral disc of metaventrite + metepisternum **554** protibia, dorsal view **555** mesotibia, ventral view **556** metatibia, ventral view.

PAPUA NEW GUINEA. 1 spec., Friedrich Wilhelm Hafen [=Madang], Biró leg., 1901 (HMNH); 1 spec., Berlinhafen [=Aitape], Tamara, Biró leg., 1896 (HMNH).

MARIANNA ISLANDS. 1 spec., Tinian Island, x.1971, M. Ali (BPBM) (distribution not shown on the map).

##### Biology.

Saprobiont, found especially on large cadavers, occasionally found also in dung.

##### Distribution.

This species was described from the Republic of South Africa, and is widespread across sub-saharan and tropical Africa to the Arab peninsula and westward through Afghanistan, Pakistan and India to the Indo-Malayan Region; it is also found in Taiwan and Japan and has been recorded in Australia (Théry et al. 2009; Mazur 2011). In Australopacific Region it is known from Australia (Queensland, Northern Territory, Victoria and Western Australia) as well as from Papua New Guinea and Marianna Islands (Lackner and Gomy 2016; Figs 755, 764).

##### Remarks.

This is a widely distributed species exhibiting a wide range of variation, especially regarding size, body coloration, density and coverage of elytral punctation, elytral striae, configuration of the two sets of prosternal striae and wing venation. According to Théry et al. (2009) much of this variation is linked to the geographic origins of the specimens. Saprinus (S.) splendens has been re-described recently in detail (Lackner and Gomy 2016) and the reader is referred to its re-description there. The species can be identified using the above key; for the sake of consistency as well as easier recognition we decided to provide it with habitus photograph, SEM micrographs as well as illustrations of male terminalia in this paper.

**Figures 557–564.**
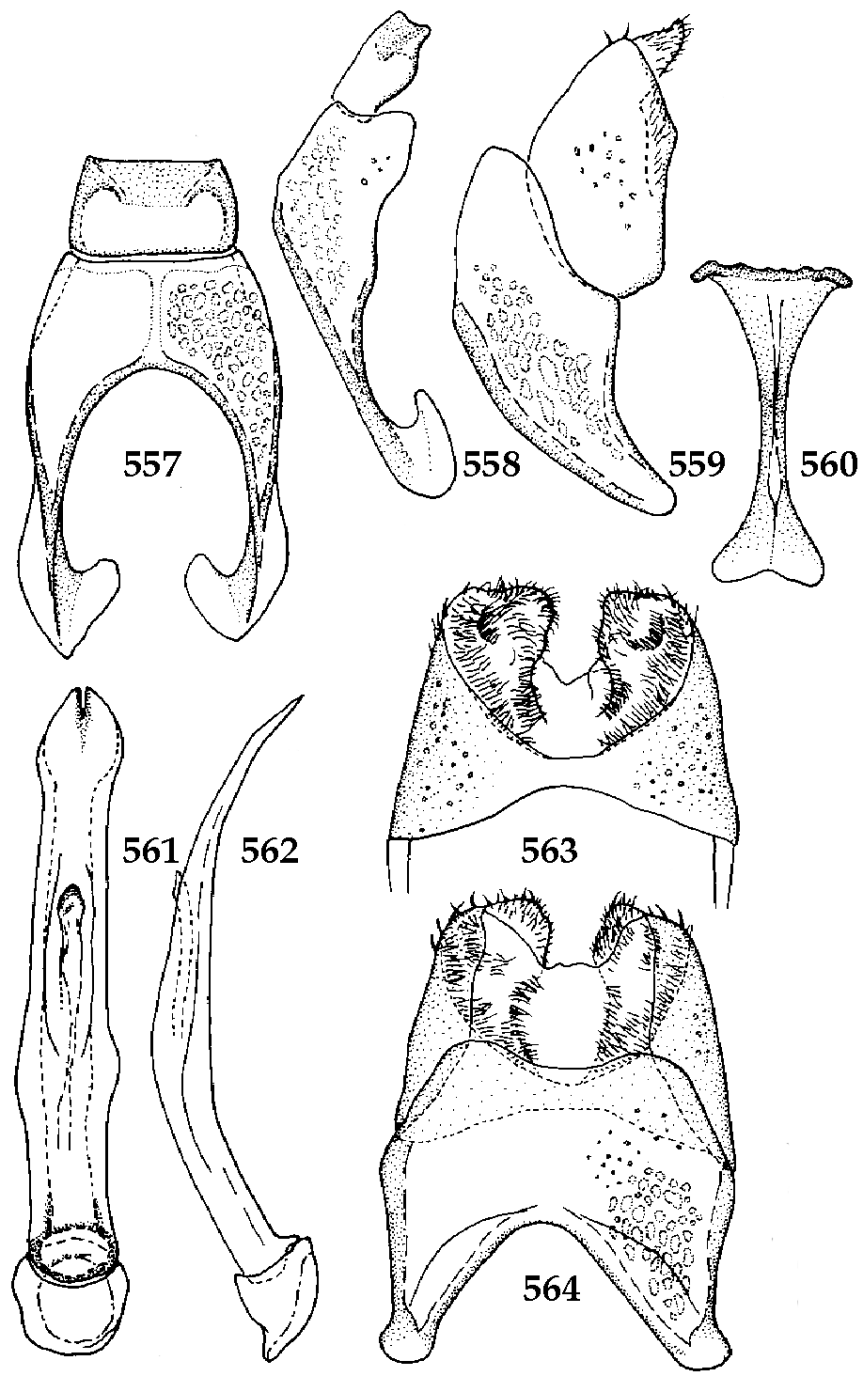
**557** Saprinus (Saprinus) splendens (Paykull, 1811) male terminalia after Ôhara (1994): 9^th^ + 10^th^ tergites, dorsal view **558** ditto, lateral view **559** male terminalia: 8^th^ sternite + 8^th^ tergite, lateral view **560** male terminalia: spiculum gastrale, ventral view **561** male terminalia: aedeagus, dorsal view **562** ditto, lateral view **563** male terminalia: 8^th^ sternite, ventral view **564** male terminalia: 8^th^ sternite + 8^th^ ventrite, dorsal view.

#### Saprinus (Saprinus) tyrrhenus

Taxon classificationAnimaliaColeopteraHisteridae

Blackburn, 1903 stat. n.

Figs 565 , 566–577 , 578–584 , 764

Saprinus tyrrhenus Blackburn, 1903: 106.

##### Type locality.

Australia: New South Wales: Tolarno.

**Figure 565.**
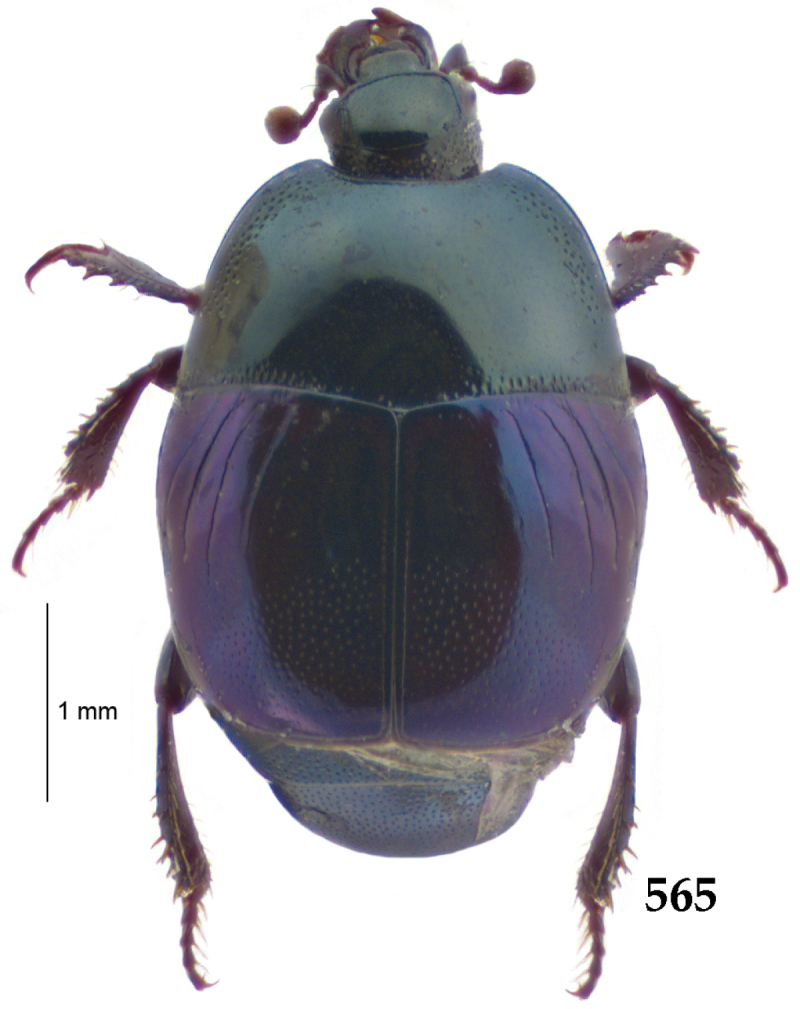
Saprinus (Saprinus) tyrrhenus Blackburn, 1903 habitus, dorsal view.

##### Type material examined.

*Saprinus tyrrhenus* Blackburn, 1903: Lectotype, present designation: ♂, with genitalia extracted and glued to the same mounting card as the specimen, basal piece of aedeagus missing, left mesotarsus and right metatarsus missing, with the following labels: “T / 7131 / TORY” (written-printed, possibly this is the original mounting card of the specimen); followed by: “Type / H.T.” (red-margined, round, printed label); followed by: “Australia. / Blackburn Coll. / B.M. 1910-236” (printed); followed by: “Saprinus / tyrrhenus, Blackb.” (written); followed by: “str. of prost. much nearer / each other than in 1229, but not / close as in 1647. Space btw. / them nitid, impunct. & much more conv. & more pointed in fr. No” (hand-written); followed by: “Saprinus tyrrhenus / Blackburn, 1903 / LECTOTYPE / Des. T. Lackner & R. Leschen 2014” (red label, written) (BMNH). This species has been described from uknown number of specimens and the lectotype designation fixes the identity of the species. Blackburn (1903) mentions the type locality as New South Wales, Tolarno (shown on Fig. 764). Although the lectotype labels do not mention this, we chose to depict the species distribution on the map showing Blackburn’s original type locality as we believe that the type specimen originated from there.

##### Additional material examined.

AUSTRALIA. New South Wales: 1 ♂, Broken Hill, A.H. Elston Collection (MAMU). South Australia: 1 ♂, side-mounted with terminalia extracted and glued to the same mounting card as the specimen, Millers CK, F. Wood Jones (SAMA).

##### Biology.

Unknown, presumably similar to congeners.

##### Distribution.

Known only from three male specimens collected in New South Wales and South Australia, respectively (Fig. 764).

##### Diagnosis.

This species is generally similar to *S. laetus*, differing from it by smaller body size, generally finer and sparser punctation of dorsum. The male genitalia of both species are likewise similar, differing chiefly in the shape of spiculum gastrale (compare Figs 473 and 581); the space between the separated parameres of aedeagus is wider in *S. laetus* than in *S. tyrrhenus* (compare Figs 475 and 583). Mazur (1997) synonymized this species with *S. pseudocyaneus* (=*S. laetus*) without having examined the type specimens of each species (Mazur, pers. comm. 2013). A very rare species, known only from three specimens hitherto.

##### Re-description.

Body length: PEL: 2.60–2.75 mm; EL: 1.625–1.75 mm; APW: 1.40–1.50 mm; PPW: 2.00–2.15 mm; EW: 2.325–2.45 mm.

Body (Fig. 565) round, convex, elytra dark brown, shining, with dark blue to violet metallic luster, pronotum darker, piceous black, with deep green metallic luster; legs, mouthparts and antennae (except for antennal club) light to castaneous brown; antennal club lighter.

**Figures 566–577.**
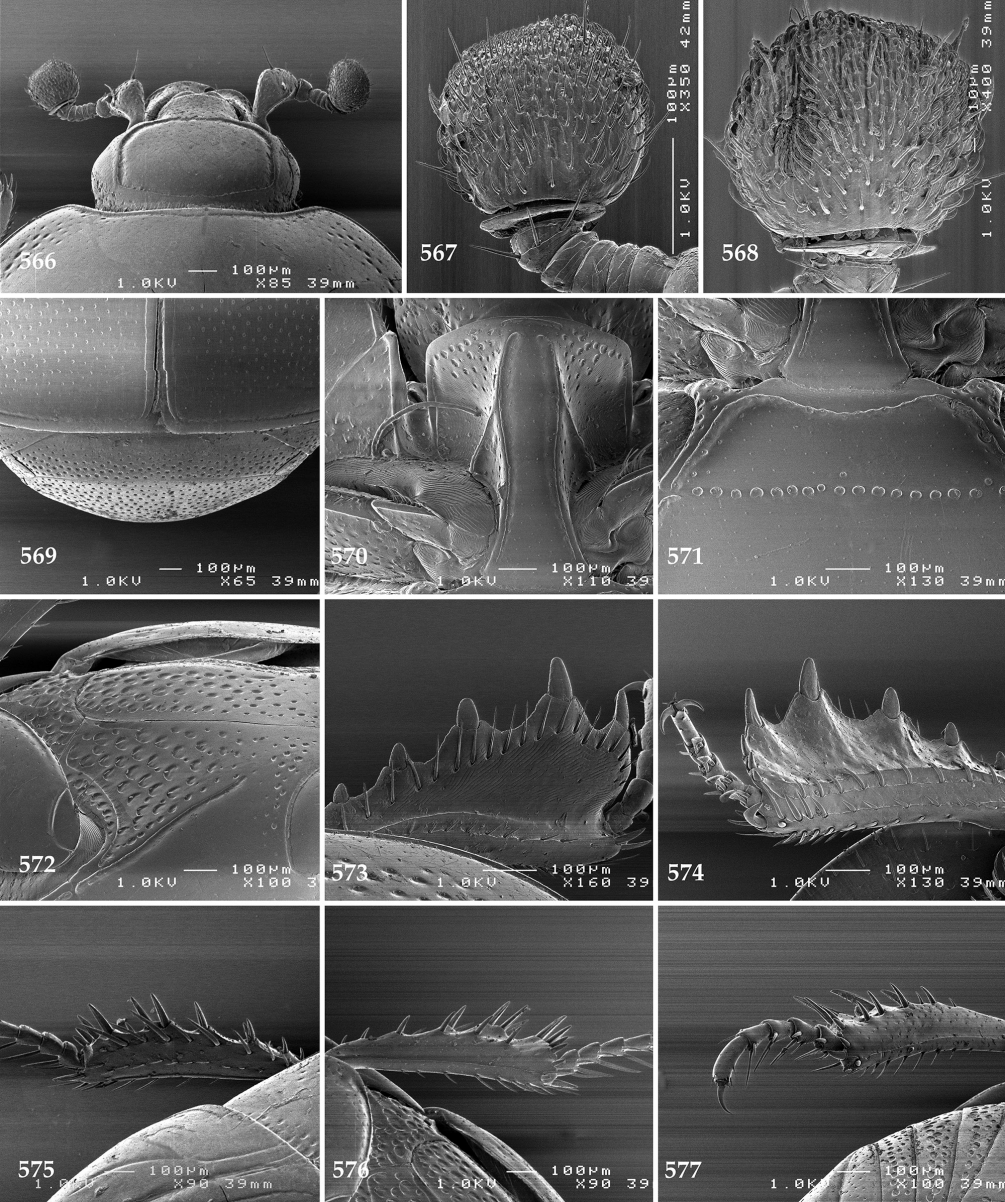
**566** Saprinus (Saprinus) tyrrhenus Blackburn, 1903 head, dorsal view **567** antennal club, dorsal view **568** ditto, ventral view **569** propygidium + pygidium **570** prosternum **571** mesoventrite **572** lateral disc of metaventrite + metepisternum **573** protibia, dorsal view **574** ditto, ventral view **575** mesotibia, dorsal view **576** ditto, ventral view **577** metatibia, ventral view.

Antennal scape (Fig. 566) slightly thickened, finely punctate, with several setae of uneven length; antennal club round (Figs 567–568) covered with moderately dense short sensilla intermingled with sparse longer erect setae; sensory structures of antennal club (Fig. 568) in a form of one oval sensory patch situated on internal distal part of the antennal club; vesicle(s) not examined.

Mandibles dorso-laterally finely punctate, rounded, outer margin carinate; labrum convex, with deep median depression; labral pits present, each with a single labral seta; other mouthparts not examined.

Clypeus (Fig. 566) with slight median depression, anterior margin slightly elevated, finely punctate; frontal and supraorbital striae complete; frontal disc (Fig. 566) finely and sparsely punctate, punctures separated several times their diameter; eyes convex, well visible from above.

Pronotal sides (Fig. 565) moderately narrowing anteriorly, apical angles prominent, pronotal depressions very shallow but present, anterior incision for head deep, semicircular; marginal pronotal stria complete, carinate, visible along its entire length from dorsal view; pronotal disc laterally with a band of deep dense elongate punctures originating approximately in pronotal depressions, but not reaching basal angles of pronotum, between it and pronotal margin a narrow smooth band present; rest of the pronotal disc with only scattered microscopic punctation; double row of fine ovoid punctures present along pronotal base not reaching ante-scutellar area; pronotal hypomeron with short sparse setae; scutellum small, visible.

Elytral epipleura almost glabrous; marginal epipleural stria complete; marginal elytral stria well impressed and slightly carinate, continued as complete apical elytral stria. Humeral elytral stria well impressed on basal third; inner subhumeral stria present as a short median fragment; four dorsal elytral striae 1–4 well impressed, in fine punctures, first, second and in the paratype also third dorsal elytral striae apically ending short of elytral half (in the case of holotype third elytral stria shortened basally), fourth dorsal elytral stria shortened, present as a short basal fragment (in the case of paratype present as an intermittent somewhat longer stria), basally not connected with sutural elytral stria; sutural elytral stria well-impressed, in fine punctures, abbreviated on basal third, apically connected with apical elytral stria; elytral disc on apical half (roughly) punctate, punctures fine, sparse, separated by several times their diameter; punctures becoming finer and sparser apically, rest of elytral disc (including elytral flanks) glabrous.

Propygidium (Fig. 569) on basal half glabrous, on apical half punctate, punctures separated by their own to twice their own diameter; pygidium (Fig. 569) with similar, if somewhat sparser punctation, becoming even sparser apically.

Anterior margin of median portion of prosternum (Fig. 570) rounded; marginal prosternal stria present laterally and also as short medial fragment; prosternal process between carinal prosternal striae slightly convex, sparsely and finely punctate; carinal prosternal striae carinate, bisinuate, not united in front (Fig. 570); lateral prosternal striae carinate, rather short, apically attaining carinal prosternal striae at about two-thirds of their length.

Discal marginal mesoventral stria (Fig. 571) well impressed, carinate, inwardly arcuate anteriorly; disc with sparse microscopic punctation; meso-metaventral sutural stria indicated by a row of large punctures; intercoxal disc of metaventrite flattened, with slight longitudinal median depression; disc of metaventrite for the most part almost glabrous, only with scattered microscopic punctation, behind metacoxa several larger punctures appear; lateral metaventral stria (Fig. 572) well impressed, carinate, almost straight, shortened, not reaching metacoxa; lateral disc of metaventrite (Fig. 572) slightly concave, with dense shallow large setigerous punctures; metepisternum (Fig. 572) similar, but with deeper and larger punctures almost without setae, on fused metepimeron punctures becoming much sparser, almost absent; metepisternal stria absent.

Intercoxal disc of first abdominal ventrite completely striate laterally; disc near metacoxa with several shallow punctures of various sizes; rest of sternite with scattered microscopic punctation, almost glabrous.

Protibia (Fig. 573) slightly dilated, outer margin with four rather large triangular teeth topped by large denticle, teeth and denticles diminishing in size proximally, followed by two minute denticles; setae of outer row regularly spaced, rather long; protarsal groove deep; anterior protibial stria present on basal two-thirds, next obliterated; setae of median row shorter and sparser than those of outer row; two tarsal denticles present near tarsal insertion; protibial spur large, bent, growing out from apical margin of protibia; apical margin of protibia ventrally with three minuscule denticles; outer part of posterior surface (Fig. 574) slightly obscurely variolate, separated from glabrous and narrow median part of posterior surface by a ridge-like stria bearing a row of well-sclerotized rather long setae; posterior protibial stria complete, bearing almost along its entire length a sparse row of setae becoming thicker apically; inner row of setae double and dense.

Mesotibia (Fig. 575) rather slender, outer margin with six denticles situated on low teeth, another row of six shorter denticles positioned on knobs situated on anterior surface of mesotibia; setae of outer row regular, sparse, strongly sclerotized, almost as long as (or even longer than) denticles themselves; setae of median row shorter and finer; posterior mesotibial stria shortened apically, almost complete; anterior surface of mesotibia (Fig. 576) sparsely punctate; anterior mesotibial stria almost complete; mesotibial spur stout, short; apical margin of mesotibia anteriorly with several short, closely-set denticles; inner margin of mesotibia with sparse row of short setae; claws of apical tarsomere bent, longer than half its length; metatibia (Fig. 577) slenderer and longer than mesotibia, in all aspects similar to it, but denticles on outer margin even sparser and knobs on which they are positioned even lower; setae of outer row longer than those of mesotibia.

**Figures 578–584.**
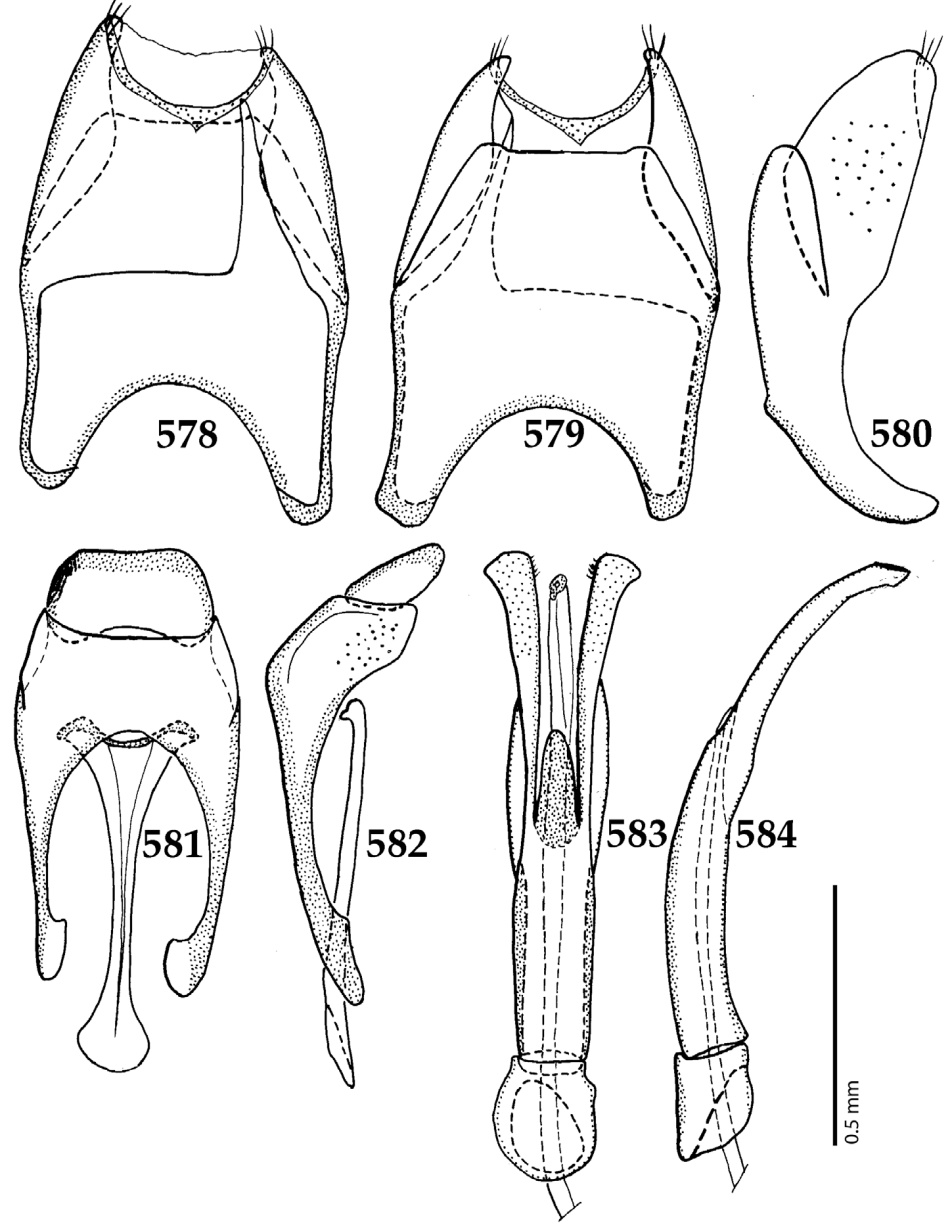
**578** Saprinus (Saprinus) tyrrhenus Blackburn, 1903 male terminalia: 8^th^ sternite + 8^th^ tergite, ventral view **579** ditto, dorsal view **580** ditto, lateral view **581** male terminalia: 9^th^ + 10^th^ tergites, dorsal view; spiculum gastrale, ventral view **582** male terminalia: 9^th^ + 10^th^ tergites; spiculum gastrale, lateral view **583** male terminalia: aedeagus, dorsal view **584** ditto, lateral view.

Male genitalia. Eighth sternite (Figs 578–579) fused medially, apically with several short setae, vela present, asetose; eighth tergite and eighth sternite fused laterally (Fig. 580). Ninth tergite (Figs 581–582) typical for the subfamily; tenth tergite basally inwardly arcuate; spiculum gastrale (Fig. 581) gradually dilated on most of its apical half; apex medially with deep emargination, apices connected by sclerotized ‘bridge’; basal end outwardly arcuate. Aedeagus (Figs 583–584) on basal half (roughly) parallel-sided, parameres fused along their basal half (roughly), thence widely separated, apices of separated parameres with microscopic setae mesally; aedeagus medially somewhat thickened; basal piece of aedeagus short, ratio of its length : length of parameres 1 : 4.5; aedeagus curved from lateral view (Fig. 584).

#### Saprinus (Saprinus) viridanus

Taxon classificationAnimaliaColeopteraHisteridae

Lewis, 1899

Figs 585 , 586–594 , 595–602 , 765

Saprinus viridanus Lewis, 1899: 22.

##### Type locality.

Northwest Australia.

**Figure 585.**
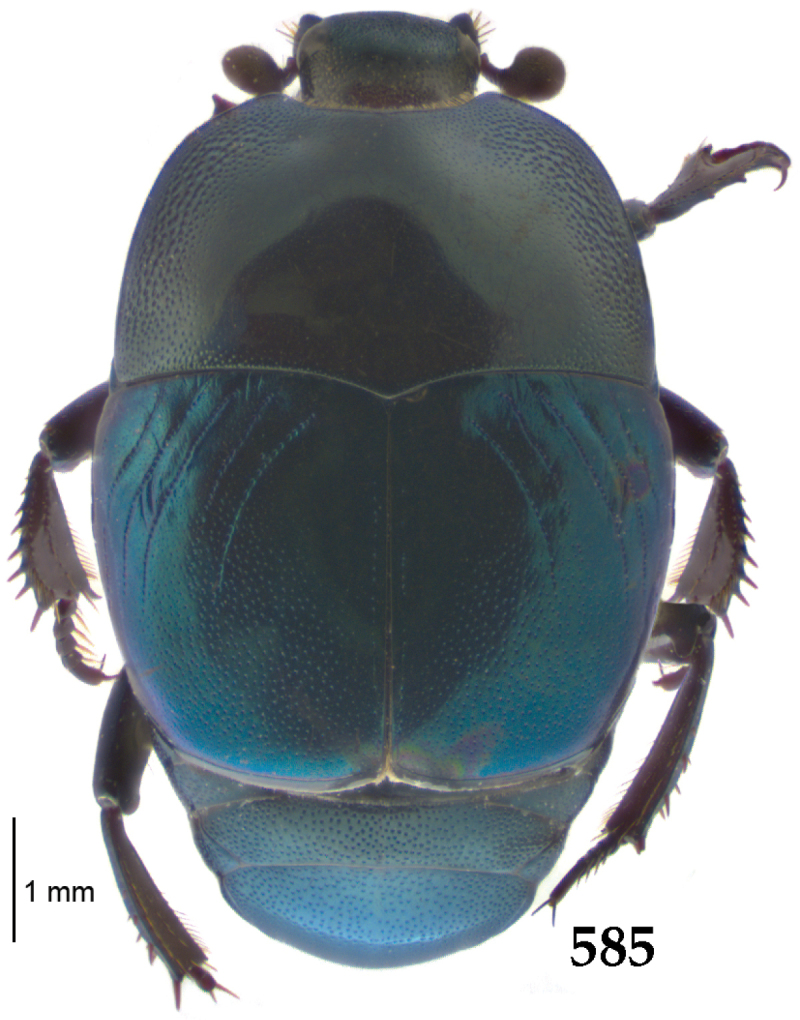
Saprinus (Saprinus) viridanus Lewis, 1899 habitus, dorsal view.

##### Type material examined.

*Saprinus viridanus* Lewis, 1899: Lectotype, present designation: unsexed specimen, pinned, final two right metatarsomeres missing, left protarsus missing, final three left mesotarsomeres missing, with the following labels: “N.W. Australia / Macl. Mus. 1899” (written); followed by: “Note / Book / 1221” (printed-written); followed by: “Saprinus / viridanus / Type Lewis” (written); followed by: “G. Lewis Coll. / B.M. 1926-369.” (printed); followed by: “Type” (round, red-margined label); followed by: “Saprinus / viridanus / LEWIS, 1899 / LECTOTYPE / des. T. Lackner ‘11” (red label, written) (BMNH). Paralectotype, present designation: ♂, side-mounted, with right protibia broken off, glued to the same mounting card as the specimen, right metatarsomere missing, with male terminalia glued to another triangular mounting card under the specimen, with the following labels: “N.W. Australia / ? King’s Sound / Macleay Mus” (written); followed by: “Note / Book / 1221” (printed-written); followed by: “Saprinus / viridanus / Cotype Lewis” (written); followed by: “G. Lewis Coll. / B.M. 1926-369.” (printed); followed by: “Cotype” (round, yellow-margined label); followed by: “09-090” (yellow, pencil-written label, added by the senior author); followed by: “Saprinus / viridanus / LEWIS, 1899 / PARALECTOTYPE / des. T. Lackner ‘11” (red label, written) (BMNH). This species has been described from unknown number of specimens and the lectotype designation fixes its taxonomic identity.

##### Additional material examined.

AUSTRALIA. New South Wales: 1 spec., K.K. Spence coll. (MAMU); 1 spec., Bogan River, no date, J. Armstrong (MAMU); 1 spec., Salt Hole Creek, 38 km NE of Broken Hill, 26.ix.1975, Z. Liepa (ANIC). South Australia: 1 spec., Nichols Bay [=Rapid Bay] (ZMHUB); 1 spec., Coober Pedy, 26.xi.1975, M. Farr (ANIC); 1 spec., 3 miles E of Mundrabilla, Eucla Basin, 22.x.1966, J. Lowry (under decomposed snake on highway) (ANIC); 2 specs., Andamooka Ranges, 29.viii.1948, O.F. Gross (SAMA). Western Australia: 1 ♂, West Australia, 95-64, without further data (BMNH); 1 spec., K.K. Spence coll., no further data (MAMU); 1 spec., Preston Beach, Yalgorup National Park, 32.53S 115.39E, 23.x.1984, J. & N. Lawrence (under dead lizard) (ANIC); 1 spec., Cranmore Park, 11.x.1933, Fouer (in trap) (ANIC). 1 spec., La Grange, 29.vii.1953, N.B. Tindale (SAMA). Northern Territory: 1 ♂, 35 km N of Erlunda, 16.vii.2000, dead kangaroo, M.A. Hielkema (NCB); 1 ♂, 11 km N of Curtin Springs, 25°18'S, 131°56'E, 470 m, 9.–10.i.2009, Sváťa Bílý leg. (NMPC); 1 spec., Wilson Creek, vii.–ix.1971, 19.08S 130.09E, J. Hodgson (ANIC); 1 spec., MacFarlanes Bore, 5.viii.1970, S. Parker (ANIC); 1 spec., ca. 85 km NW of Yuenmundu, 22.15S 131.48E, 29.vi.1970, S. Parker (ANIC); 2 specs., “Wunarah”, near Turnoff, 19°59.4'S, 136°38.3'E, 8.–11.ii.2007, 270 m, D.J. Cook (QM); 1 spec., Barkly Roadhouse, 1.8 km SE, 230 m, 19°43.2'S, 135°50.4'E, 8.–10.ii.2007, D.J. Cook (dung pitfall trap) (QM); 1 spec., McDonnell Ranges, Capt. S.A. White (SAMA). Queensland: 1 spec., N. Queensland, Blackburn’s Coll., no further data (SAMA).

##### Biology.

Found on carcasses, collected also in a dungfall trap; not common.

##### Distribution.

Australia: New South Wales, Queensland, Northern Territory, Western Australia, and South Australia (Fig. 765).

##### Remarks.

This is a rare, sporadically collected species.

##### Re-description.

Body length: PEL: 5.80–6.70 mm; EL: 3.70–4.10 mm; APW: 1.70–1.90 mm; PPW: 4.80–5.10 mm; EW: 5.30–5.50 mm.

Body (Fig. 585) rectangular oval, convex, elytra with stark green or dark blue metallic luster, pronotum darker, piceous black (occasionally with dark green hue); legs, mouthparts and antennal scape castaneous brown; antennal club black.

**Figures 586–594.**
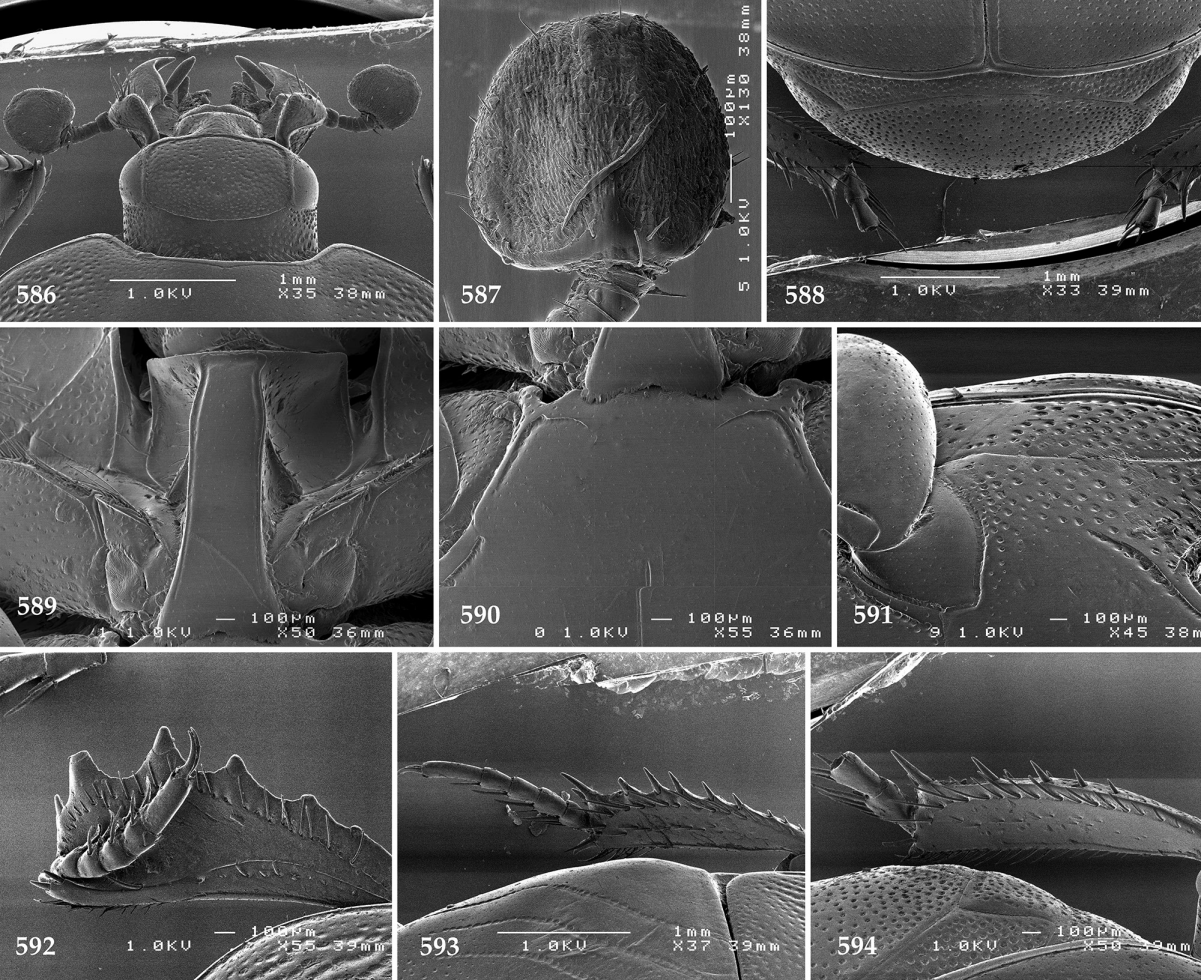
**586** Saprinus (Saprinus) viridanus Lewis, 1899 head, dorsal view **587** antennal club, ventral view **588** propygidium + pygidium **589** prosternum **590** mesoventrite **591** lateral disc of metaventrite + metepisternum **592** protibia, dorsal view **593** mesotibia, dorsal view **594** metatibia, dorsal view.

Antennal scape (Fig. 586) black, slightly thickened, punctuate, dorsally with several long, strongly sclerotized setae, ventrally with numerous much finer and shorter setae; antennal club large, circular, covered with dense short sensilla intermingled with sparse longer erect setae; sensory structures of antennal club (Fig. 587) in form of four large oval sensory patches on ventral side of the club, vesicles not examined.

Mandibles rounded, outer margin carinate, with several short setae, mandibular apex acute, sub-apical tooth on left mandible obtuse; labrum finely and sparsely punctuate, convex, with deep median depression; labral pits present, each with two labral setae; mentum medially with deep notch, surface around it on each side with two long, strongly sclerotized setae, lateral margins and anterior angles of mentum with much shorter denser setae; maxillary and labial palpi thin, elongate; terminal segments of labial and maxillary palpi thin, several times its width, about twice as long as penultimate segment; other mouthparts not examined.

Clypeus (Fig. 586) medially flattened, sloping down laterally, finely punctuate; frontal stria complete, straight, well-impressed; supra-orbital stria carinate; frontal disc (Fig. 586) punctuate, punctures deep and coarse separated by their own to several times their diameter; eyes convex, well visible from above.

Pronotal sides (Fig. 585) on basal half only moderately narrowing anteriorly, on apical half strongly narrowing anteriorly, apical angles obtuse, pronotal depressions present, rather large and deep, anterior incision for head deep; marginal pronotal stria not complete, not reaching basal pronotal angles posteriorly, carinate, slightly distanced from pronotal margin, visible along its entire length from dorsal view, behind head weakened, with tiny median angulate projection; pronotal disc laterally with a band of deep dense variously sized and shaped punctures (occasionally punctures confluent) originating in apical pronotal angles reaching basal angles of pronotum, punctation slightly continuous along pronotal base, but not reaching antescutellar area; rest of the pronotal disc with only scattered microscopic punctation; pronotal hypomeron setose; scutellum very small, visible.

Elytral epipleura with sparse fine punctures; marginal epipleural stria complete; marginal elytral stria well impressed and carinate, continued as complete (weakened) apical elytral stria which is connected to sutural elytral stria. Humeral elytral stria weakly impressed, inner subhumeral stria present as short median fragment (occasionally the two striae connected); four dorsal elytral striae 1–4 present, in punctures, first and second in more coarse punctures than third and fourth (occasionally third or fourth stria shortened apically or even evanescent) surpassing elytral half apically (length of elytral striae varies); fourth (or even third) dorsal elytral stria sometimes intermittent or even missing, basally not connected with sutural elytral stria; sutural elytral stria well-impressed, in fine sparse punctures, abbreviated on basal sixth, apically connected with apical elytral stria; elytral disc punctate, punctures fine, round, separated by their own to several times their diameter, becoming denser apically, usually punctation absent on fourth elytral interval (occasionally punctures absent also on third and even second elytral interval), punctation weakens basally, around sutural elytral stria elytral surface almost glabrous.

Propygidium (Fig. 588) densely punctate, punctures separated by their own to twice their own diameter; pygidium (Fig. 588) with similar, less dense punctation, punctures round, separated several times their diameter.

Anterior margin of median portion of prosternum (Fig. 589) almost straight, rounded laterally; marginal prosternal stria present laterally; prosternal process slightly convex, sloping down on anterior fourth, surface between carinal prosternal striae almost glabrous, with scattered microscopic punctation, surface laterad of carinal prosternal striae setose, cuticle near united apices of carinal prosternal striae slightly depressed; carinal prosternal striae carinate, almost parallel, not united in front (Fig. 589); lateral prosternal striae carinate, short, in setae, apically attaining carinal prosternal striae at about four-fifths of their length.

Discal marginal mesoventral stria (Fig. 590) weakly impressed, widely interrupted medially and shortened laterally; disc convex, with sparse microscopic punctation; meso-metaventral sutural stria absent; meso-metaventral suture fine, thin; intercoxal disc of metaventrite convex, with slight longitudinal median depression; disc of metaventrite for the most part almost smooth, with scattered microscopic punctation, along posterior margin several rows of fine punctation appear; lateral metaventral stria (Fig. 591) well impressed, shortened; lateral disc of metaventrite (Fig. 591) slightly concave, with large deep punctures separated by about their own to twice their diameter; metepisternum (Fig. 591) with similar punctures becoming much sparser on fused metepimeron; metepisternal stria present, deeply impressed.

Intercoxal disc of first abdominal ventrite completely striate laterally; disc along basal and lateral margins with shallow punctures of various sizes; rest of sternite glabrous.

Protibia (Fig. 592) dilated, outer margin with three large triangular teeth topped by triangular denticle, followed by one very low tooth and one microscopic denticle; setae of outer row regular, short; protarsal groove deep; anterior protibial stria complete; setae of median row shorter and much sparser than those of outer row; two tarsal denticles present near tarsal insertion; protibial spur bent, growing out from apical margin of protibia; anterior margin of protibia ventrally with single (occasionally two or even more) short denticle; outer part of posterior surface slightly obscurely variolate, separated from glabrous and narrow median part of posterior surface by an indefinite stria bearing several setae; posterior protibial stria complete, bearing almost along its entire length dense row of strongly sclerotized setae; inner row of setae double, setae dense, shorter and finer than those of posterior protibial stria.

Mesotibia (Fig. 593) slender, outer margin with a row of about seven long denticles growing in size and girth apically, another row of much shorter sparser denticles situated on anterior surface of mesotibia; setae of outer row regular, thin, shorter than denticles; setae of median row even shorter and finer; posterior mesotibial stria shortened apically; anterior surface of mesotibia sparsely punctuate; anterior mesotibial stria almost complete, terminating in single tiny denticle; mesotibial spur stout; apical margin of mesotibia anteriorly with two (occasionally more) short denticles; inner margin of mesotibia with dense row of short setae; claws of apical tarsomere slightly bent, longer than half its length; metatibia (Fig. 594) more slender and longer than mesotibia, in all aspects similar to it, but denticles on outer margin much shorter and sparser.

**Figures 595–602.**
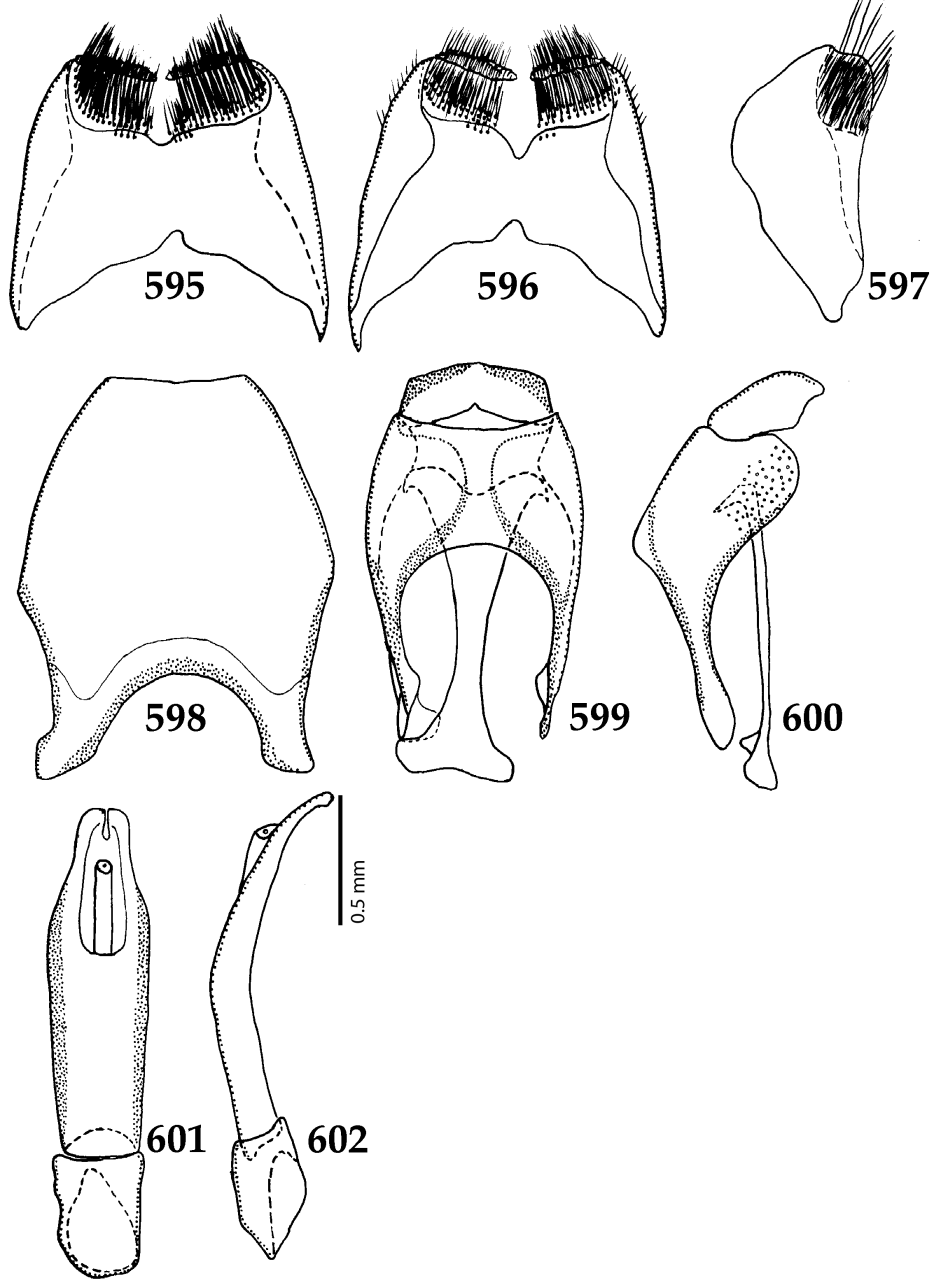
**595** Saprinus (Saprinus) viridanus Lewis, 1899 male terminalia: 8^th^ sternite, ventral view **596** ditto, dorsal view **597** ditto, lateral view **598** male terminalia: 8^th^ tergite, dorsal view **599** male terminalia: 9^th^ + 10^th^ tergites, dorsal view; spiculum gastrale, ventral view **600** male terminalia: 9^th^ + 10^th^ tergites; spiculum gastrale, lateral view **601** male terminalia: aedeagus, dorsal view **602** ditto, lateral view.

Male genitalia. Eighth sternite (Figs 595–596) fused medially, apical third laterally with several microscopic setae, vela present, densely setose; eighth tergite and eighth sternite fused laterally; eighth tergite (Fig. 598) only slightly inwardly arcuate apically. Ninth tergite (Figs 599–600) typical for the subfamily; tenth tergite rounded apically; spiculum gastrale (Fig. 599) gradually dilated on most of its apical half, ‘head’ deeply arcuate medially; basal end dilated, faintly inwardly arcuate. Aedeagus (Figs 601–602) parallel-sided, with parameres fused along their basal two-thirds (roughly), on apical third aedeagus narrowing apically; basal piece of aedeagus short, ratio of its length : length of parameres 1 : 4; aedeagus slightly curved from lateral view (Fig. 602).

#### Saprinus (Saprinus) viridipennis

Taxon classificationAnimaliaColeopteraHisteridae

Lewis, 1901

Figs 603 , 604–611 , 612–618 , 765

Saprinus viridipennis Lewis, 1901: 245.
Saprinus desbordesi Auzat, 1916: 32 – Synonymized by Dahlgren (1967): 220.

##### Type locality.

Australia.

**Figure 603.**
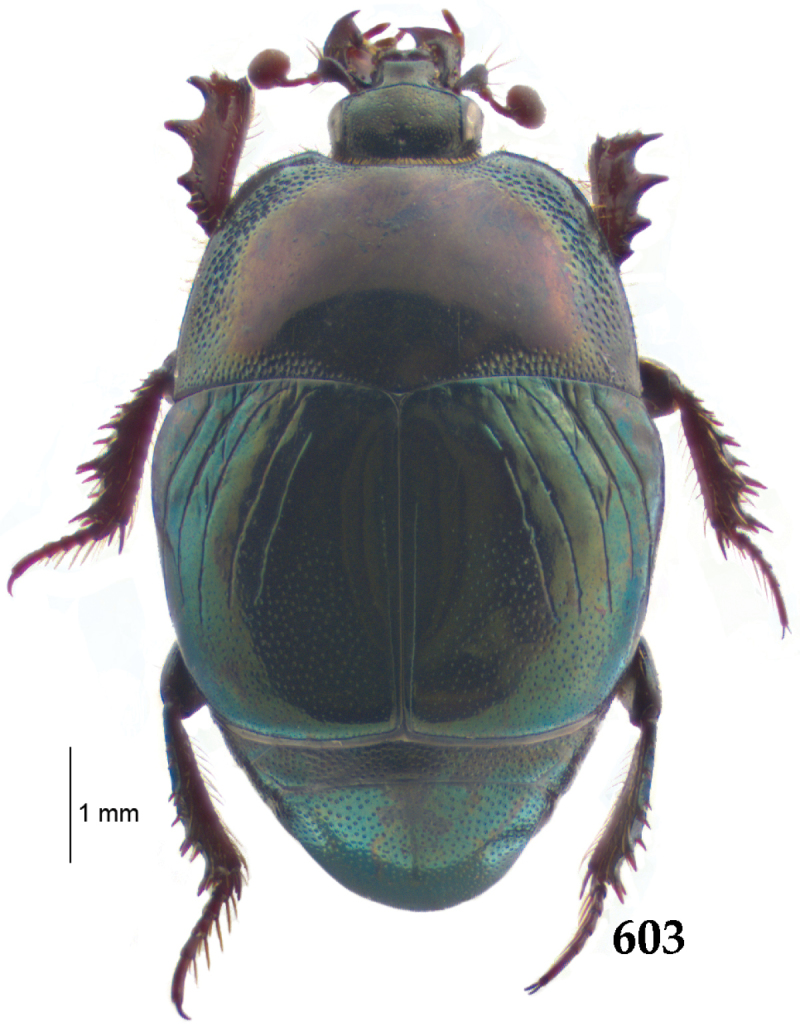
Saprinus (Saprinus) viridipennis Lewis, 1901 habitus, dorsal view.

##### Type material examined.

*Saprinus viridipennis* Lewis, 1901: Lectotype, present designation: ♀, side-mounted on a triangular mounting card with genitalia extracted and mounted on another mounting card under the specimen, except for left mesotarsus all tarsi are broken off to some degree, with the following labels: “Australie / CH FRENCH” (written); followed by: “Prosternum / without striae” (written); followed by: “Saprinus / viridipennis / Type Lewis” (written); followed by: “G. Lewis Coll. / B.M. 1926-369” (printed); followed by: “Type” (round, printed red-margined label); followed by: “08-078” (yellow, pencil-written label, added by the senior author); followed by: “LECTOTYPE / Saprinus viridipennis / Lewis, 1901 / Designated by / T. Lackner, 2008” (red label, written) (BMNH). This species has been described from numerous specimens (“Examples in the Belgium Museum and in my own cabinet.” – Lewis 1901: 245) and the lectotype designation fixes the identity of species. 1 paralectotype (present designation), ♀, with blue round label, followed by: “Co-type” (round, printed, yellow-margined label); followed by: “Australie / CH FRENCH” (written); followed by: “S. viridipennis / Cotype Lewis” (BMNH).

*Saprinus desbordesi* Auzat, 1916: 32: Lectotype, present designation: ♂, all tarsi and right mesotibia missing, male genitalia extracted, glued on the same mounting card as the specimen, with following labels: “Saprinus / Desbordesi / Typus / Dr. Auzat det 1916” (printed-written); followed by: “MUSÉUM PARIS / 1933 / Coll. DESBORDES” (printed); followed by: “TYPE” (red label, printed); followed by: “Sapr. VIRIDIPEN- / NIS LEW. / G Dahlgren det” (printed-written); followed by: “Saprinus desbordesi / Auzat, 1916 / LECTOTYPE 2014 / des. T. Lackner” (red label, written) (MNHN). Paralectotype (present designation): ♀, with genitalia extracted, glued onto the same label as specimen, all tarsi and left metatibia broken off, with the following labels: “Desbordesi / Typus / dr. Auzat det. 1916” (printed-written); followed by: “MUSÉUM PARIS / 1933 / Coll. DESBORDES” (printed); followed by: “TYPE” (red label, printed); followed by: “Sapr. VIRIDIPEN- / NIS LEW. / G Dahlgren det” (printed-written); followed by: “Saprinus desbordesi / Auzat, 1916 / PARALECTOTYPE / des. T. Lackner 2014” (red label, written) (MNHN). Paralectotype (present designation), ♀, pinned, two segments of right mesotarsus, entire left mesotarsus and two segments of left metatarsus missing, with the following labels: “♀” (printed); followed by: “S. Austral.” (written); followed by: “S. Austral. / coll. Théry” (written); followed by: “Saprinus / Desbordesi / Typus / D. Auzat det 1916” (printed-written); followed by: “TYPE” (red label, printed); followed by: “Saprinus / desbordesi / Auzat, 1916 / PARALECTOTYPE / des. T. Lackner 2017” (red label, written) (MNHN, coll. Thérond). This species has been described from three specimens; two of them were from Desbordes collection and third was from Auzat’s collection (Auzat 1916). The lectotype designation fixes the species identity.

##### Additional material examined.

AUSTRALIA. Victoria: 1 ♂, Merinqur, 3.i.1931, C.E. Clarke (AMNZ); 2 specs., Melbourne, without further data (ZMHUB). South Australia: 1 spec., Gawler Geb., without further data (ZMHUB); 1 ♂ & 1 ♀, S. Australia, without further data (BMNH); 1 spec., Maralinga, viii.–x.1956, F.L. Hill (BMNH); 2 ♂♂ & 1 ♀, Killalpanima, 100 miles of L. Eyre, H.J. Hillier (BMNH); 1 spec., Kingonya, R. Harvey (SAMA); 1 spec., Oodnadatta, Blackburn (SAMA); 1 spec., Purple Downs (SAMA); 2 spec., Innamincka, W. Lamb (SAMA); 5 specs., Cooper’s Creek, J.G. Reuther (SAMA); 3 specs., Mt. Serle, N Flinders Ra., Hale & Tindale (SAMA). Northern Territory: 1 ♂ & 3 ♀♀, Hermansburg, H.J. Hillier, 1908-177 (BMNH); Coniston Station, near Alice Springs, M.W. Mules (SAMA). New South Wales: 2 ♂♂, Alexandria, New South Wales, W. Stalker, x.[19]05 (BMNH); 4 specs., Lake Callabona, A. Zietz (SAMA). Queensland: 2 specs., Cunnamulla, H. Hardcastle (both exs. SAMA). Western Australia: 1 ♂, Nicol Bay Distr., Dr. Clement, 1900-220; 1 ♀, West Australia, 95-64 (BMNH).

Unknown localities: 3 specs., Australia occid. 1192, without further data (HNMH).

##### Biology.

Presumably similar to congeners; an uncommon species.

##### Distribution.

Australia: Victoria, South Australia, Western Australia, Northern Territory, New South Wales & Queensland (Fig. 765).

##### Remarks.

A rare species, easily distinguishable from the most similar *S. cyaneus* by the presence of long amber setae of pronotal hypomeron easily visible from above, the complete absence of carinal prosternal striae and setose lateral prosternal striae. The protibiae differ from *S. cyaneus* or *S. laetus* by their larger triangular teeth.

##### Re-description.

Body length: PEL: 4.25–5.50 mm; APW: 1.60–2.00 mm; PPW: 3.50–4.70 mm; EL: 2.60–3.50 mm; EW: 3.75–5.00 mm. Body (Fig. 603) rectangular oval, convex, elytra widest at humeri, elytra stark green, shining, metallic; pronotum purple, metallic; legs, mouthparts and antennal scape castaneous; antennal club light brown.

**Figures 604–611.**
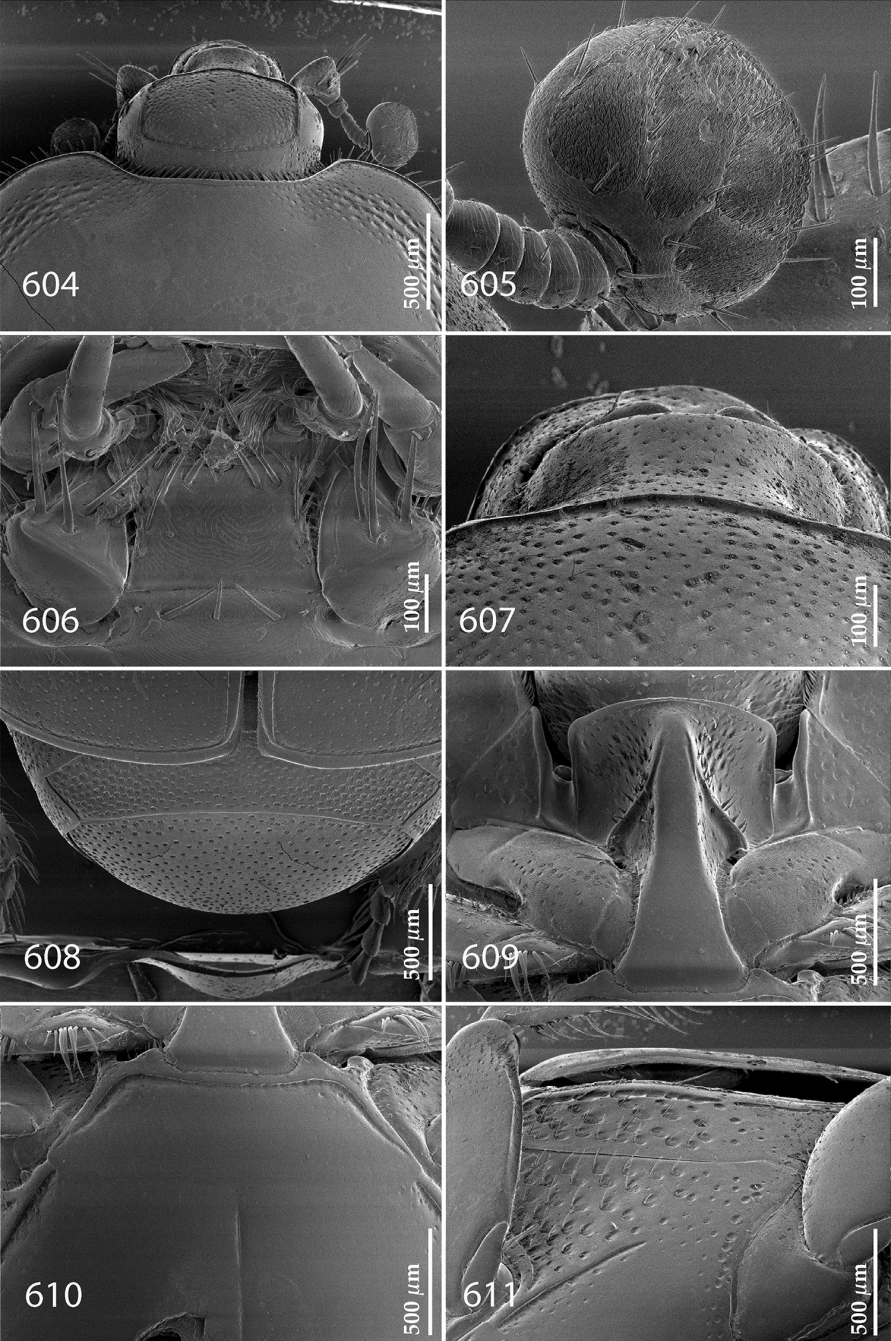
**604** Saprinus (Saprinus) viridipennis Lewis, 1901 head, dorsal view **605** antennal club, ventral view **606** mentum, ventral view **607** clypeus **608** propygidium + pygidium **609** prosternum **610** mesoventrite **611** lateral disc of metaventrite + metepisternum.

Antennal scape (Fig. 604) triangularly thickened, with several long setae; antennal club large, wider than long, depressed dorso-ventrally, somewhat truncate apically, dorsally on lower fourth asetose, rest of club covered with sparse to dense short sensilla intermingled with sparse longer erect setae; ventrally four large oval sensory structures of antennal club (Figs 605) easily observable; vesicle(s) not examined.

Mandibles dorso-laterally punctuate, rounded, stout, outer margin slightly carinate, mandibular apex acute, sub-apical tooth on left mandible obtuse; labrum finely and sparsely punctuate, convex, broadly depressed medially; labral pits present, each with two short labral setae; terminal labial palpomere elongate, pointed apically, approximately twice as long as pen-ultimate, its length about three times its width; terminal maxillary palpomere elongate, about twice as long as pen-ultimate, approximately three times as long as wide, pointed apically; mentum (Fig. 606) sub-trapezoid, laterally with a row of short ramose setae, several short setae present also on imbricate disc; anterior margin broadly inwardly arcuate medially, a tuft of four longer setae present near median notch.

Clypeus (Fig. 607) faintly concave, sloping down laterally, microscopically punctate; frontal and supraorbital striae complete; frontal disc (Fig. 604) punctate, punctures separated by about their own to twice their diameter; eyes convex, well visible from above.

Pronotal sides (Fig. 603) on basal half gently narrowing anteriorly, on apical half strongly narrowing, apical angles prominent, pronotal depressions present, shallow, anterior incision for head moderate; marginal pronotal stria carinate, visible along its entire length from dorsal view, ending short of pronotal base; pronotal disc laterally with a depressed band of deep dense coarse elongate punctures originating approximately in pronotal depressions, reaching basal angles of pronotum, between it and pronotal margin a broad glabrous band present; rest of the pronotal disc with only scattered microscopic punctation (or even glabrous in some specimens); several rows of punctures present along pronotal base, ante-scutellar area impunctate; pronotal hypomeron in dense amber setae well visible also from dorsal view; scutellum small, visible.

Elytral epipleura almost impunctate; marginal epipleural stria complete; marginal elytral stria well impressed and carinate, continued as weakened, but complete apical elytral stria that is connected to incomplete sutural elytral stria. Humeral elytral stria well impressed on basal third, sometimes connected to rather long median fragment of inner subhumeral stria creating thus a complementary dorsal elytral stria parallel to first; elytra usually with four thin impunctate dorsal elytral striae 1–4; striae 1–3 originating at elytral base and slightly surpassing elytral half apically, fourth dorsal elytral stria occasionally slightly shortened basally, can be variously long apically, even intermittent; in first elytral interval often elongate strioles present; fourth elytral stria basally curved toward shortened sutural elytral stria, but never connected with it; sutural elytral stria abbreviated on basal fourth to fifth, well-impressed, apically connected with apical elytral stria; elytral disc on apical half (roughly) punctate, punctures very fine, sparse, separated by several times their diameter, slightly entering elytral intervals and reaching beyond elytral mid-length basally between fourth elytral and sutural stria; elytral flanks impunctate; punctures reach elytral apex.

Propygidium (Fig. 608) densely punctate laterally, punctures becoming sparser medially; pygidium (Fig. 608) with more even, but sparser punctation.

Anterior margin of median portion of prosternum (Fig. 609) straight; marginal prosternal stria present laterally and also as medial fragment; prosternal process on prosternal keel convex, glabrous; carinal prosternal striae absent; lateral prosternal striae carinate, rather short, surface around them with long amber setae.

Discal marginal mesoventral stria (Fig. 610) weakly impressed, complete, anteriorly faintly inwardly arcuate, at times interrupted medially; disc slightly convex, glabrous; meso-metaventral stria absent; meso-metaventral suture visible; intercoxal disc of metaventrite glabrous, only in post-metacoxal area with several tiny punctures, in males with faint median longitudinal depression, in females slightly convex; lateral metaventral stria (Fig. 611) well impressed, carinate, straight, shortened; lateral disc of metaventrite (Fig. 611) slightly concave, with sparse shallow round setigerous punctures separated by less than their to about their own diameter, between large punctures another type of much finer sparse punctures present; metepisternum (Fig. 611) similar, but punctures somewhat deeper, on fused metepimeron punctures disappear; metepisternal stria present, deeply impressed, often complete. Intercoxal disc of first abdominal ventrite completely striate laterally, glabrous.

Protibia slightly dilated, outer margin with two large triangular teeth topped by triangular denticle, followed by one more lower tooth topped by denticle and another two tiny denticles diminishing in size proximally; setae of outer row regular, short; protarsal groove shallow; anterior protibial stria present on basal half, next obliterated; setae of median row approximately as long as those of outer row, but sparser; two tarsal denticles present near tarsal insertion; protibial spur bent, large and stout, growing out from apical margin of protibia; apical margin of protibia ventrally with three tiny denticles; outer part of posterior surface obscurely variolate, punctate, separated from glabrous and narrow median part of posterior surface; posterior protibial stria complete, bearing along its length a dense row of long, well-sclerotized setae; inner row of setae long, brush-like.

Mesotibia on outer margin with about five widely separated denticles, fourth and fifth denticles situated on prominent teeth; median tooth of mesotibia bears two denticles, best observable from ventral view; setae of outer row strongly sclerotized, sparse, almost as long as denticles; setae of median row shorter and finer than those of outer row; posterior mesotibial stria shortened on apical half; mesotibial spur rather long and stout; on anterior face of mesotibia a row of about four widely-spaced denticles present; anterior face of mesotibia with scattered fine punctation; anterior mesotibial stria incomplete; inner anterior denticles weakly developed, usually only one or two present; apical margin of mesotibia with a dense row of about four stout denticles; setae of inner row rather long, sparse. Metatibia longer and more slender than mesotibia; teeth on its outer margin lower than those of mesotibia; denticles of both rows sparser.

**Figures 612–618.**
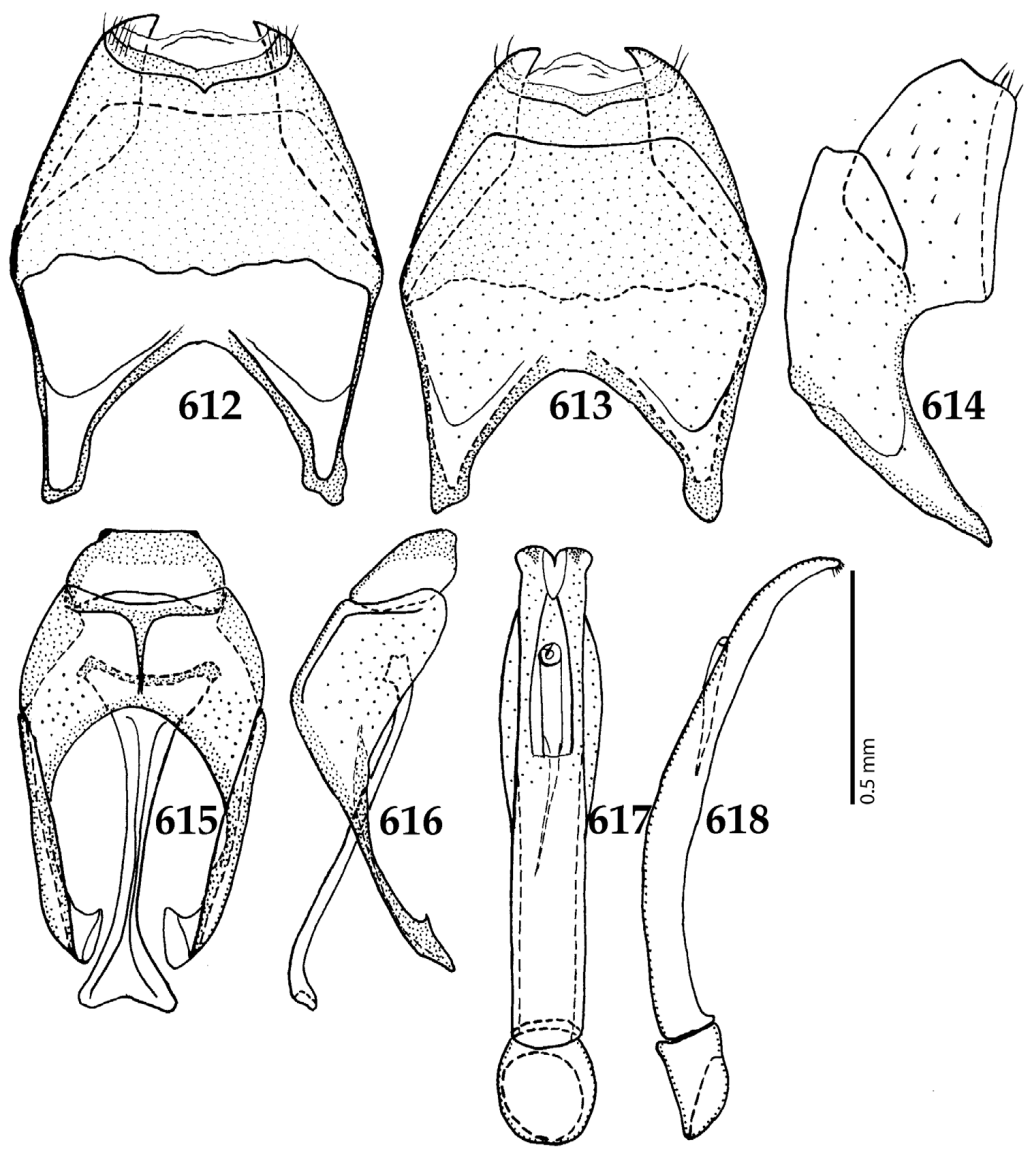
**612** Saprinus (Saprinus) viridipennis Lewis, 1901 male terminalia: 8^th^ sternite + 8^th^ tergite, ventral view **613** ditto, dorsal view **614** ditto, lateral view **615** male terminalia: 9^th^ + 10^th^ tergites, dorsal view; spiculum gastrale, ventral view **616** male terminalia: 9^th^ + 10^th^ tergites; spiculum gastrale, lateral view **617** male terminalia: aedeagus, dorsal view **618** ditto, lateral view.

Male genitalia. Eighth sternite (Figs 612–613) about as broad as long, strongly sclerotized, fused medially, with sparse pores and pseudopores, apex laterally with two short setae, vela present, short, laterally with two tufts of short setae; eighth tergite apically faintly inwardly arcuate, basally strongly inwardly arcuate; eighth tergite and eighth sternite fused laterally (Fig. 614). Ninth tergite (Figs 615–616) laterally strongly sclerotized, medially with a strongly sclerotized part resembling a fusion seen also in other congeners, with pores and pseudopores, well visible, especially laterally; ninth tergite basally faintly inwardly arcuate; tenth tergite basally inwardly arcuate, apices strongly sclerotized; spiculum gastrale (Fig. 615) dilated on both ends, otherwise almost parallel-sided, apical end strongly sclerotized, basal end slightly inwardly arcuate, cordate. Aedeagus (Figs 617–618) on basal half almost parallel-sided, on apical half dilated and thickened; parameres fused along basal two-thirds; aedeagus laterally slightly curved from lateral view; basal piece of aedeagus short, ratio of its length : length of parameres approximately 1 : 6; apex of aedeagus slightly dilated, ventrally with a tuft of microscopic setae.

#### Saprinus lindrothi

Taxon classificationAnimaliaColeopteraHisteridae

Dahlgren, 1968 = Saprinus prasinus Erichson, 1834 syn. n.

Saprinus lindrothi Dahlgren, 1968: 264.

##### Type material examined.

*Saprinus lindrothi* Dahlgren, 1968: holotype, ♂, side-mounted on triangular mounting card, with the following labels: “Australien / 1” (pencil-written); followed by: “Australien” (hand-written with black ink); followed by: “Coll. / E. Witte” (printed); followed by: “Holo- / Typus” (black-margined red label, written-printed); followed by: “*Saprinus* / *lindrothi* / det. S. Mazur ’98” (black-margined white label, printed-written); followed by: “10-125” (yellow label, pencil-written, added by the senior author); followed by: “Senckenberg- / Museum / Frankfurt/Main” (printed) (SMF).

##### Remarks.

*Saprinus lindrothi* is conspecific with *S. prasinus* Erichson, 1834, agreeing with the description of *S. prasinus* as published by Kryzhanovskij and Reichardt (1976) and sharing all significant characters. We compared the type specimen of *S. lindrothi* to verified specimens of *S. prasinus* from Israel. We found no other specimens of *S. lindrothi* among Australopacific material, and therefore conclude that the species has been mislabeled and is not treated in full detail in this study.

#### Tomogenius

Taxon classificationAnimaliaColeopteraHisteridae

Marseul, 1862

Figs 619 , 620–628 , 629–637 , 638 , 639–647 , 648–650 , 651–659 , 660 , 661–669 , 670–678 , 679 , 680–688 , 689–697 , 698 , 699–707 , 708–715 , 716 , 717–722 , 723–731 , 732 , 733–741 , 742–750 , 755 , 766 , 767

Tomogenius Marseul, 1862: 499. Type species Saprinus incisisternus Marseul, 1862 (=Tomogenius incisus (Erichson, 1842)), by monotypy.

##### Diagnosis.

Cuticle brown to black; elytra in one species with faint bluish hue; frontal, supraorbital striae absent (in *T. papuaensis* supraorbital striae vaguely present); lacinial hook present; antennal club with two oval sensory areas dorsally and two slit-like pits ventrally. Elytral epipleuron with double marginal epipleural stria; elytra with short hooked appendix between fourth dorsal and sutural striae (absent in *T. papuaensis*); pronotal depressions absent; prosternum apically with two large deep foveae, separated by thin ‘bridge’ formed by the apex of prosternal process (in case of one species widely separated); lateral costa of antennal groove reaching prosternal process (except in *T. papuaensis*), but not elevated; ninth tergite of male genitalia divided longitudinally.

**Figure 619.**
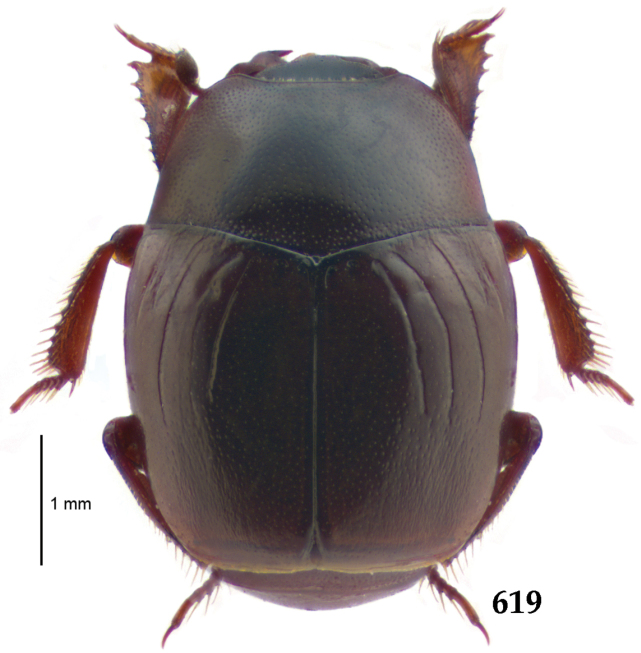
*Tomogenius australis* Dahlgren, 1976 habitus, dorsal view.

##### Biology.

Species of the genus *Tomogenius* are found in bat guano as well as bird’s nests: *T. latipes* has been collected in the guano of the New Zealand lesser short-tailed bat (*Mystacina tuberculata* Gray, 1843) and in the nest of the New Zealand Kingfisher *Halcyon sancta vagans* (Gray, 1844). *Tomogenius australis* and *T. kuscheli* have been found in petrel burrows (Dahlgren 1976; *T. kuscheli* has also been found in the nest of Fluttering Shearwater, *Puffinus gavia* (Forster, 1844)); *T. latipes* has been found in the nest of Kaka (*Nestor meridionalis* Gmelin, 1788; Jackson 1963), as well as sifted from the forest litter; *T. ripicola* was collected in bat guano and under a sheep carcass.

##### Distribution.

The genus is endemic for the Australopacific Region: seven species are currently known from Australia, New Zealand and New Guinea (Mazur 2011; Figs 766–767).

##### Remarks.

*Tomogenius* is most similar to the Holarctic genus *Gnathoncus* Jacquelin du Val, 1857 from which it differs chiefly by larger body size, different arrangement of sensory structures of antennal club, lateral costa of antennal groove not being elevated and the presence of two large median foveae situated at the apex of prosternal process. On the other hand, it shares with *Gnathoncus* several putative synapomorphies: absent both frontal and supraorbital striae, double marginal epipleural stria, presence of characteristic short, hooked appendix between fourth dorsal and sutural stria (except *T. papuaensis*), presence of lacinial hook (=uncus) of maxilla and longitudinally divided ninth tergite of male genitalia. *Tomogenius* and *Gnathoncus* are both found mostly in nests of birds and/or mammals, with some species also found in caves presumably feeding on arthropod larvae present on guano or carrion. They represent an amphi-polar clade with few species from the tropics (only two of the 25 currently known species of *Gnathoncus* are present in the Old World tropics; see Mazur 2011 and Vienna and Ratto 2013). In the published phylogenetic analysis of the senior author (Lackner 2014d), *Tomogenius* and *Gnathoncus* were recovered as sister taxa, supported by three ‘strong’ synapomorphies. Together with the genera *Erebidus* Reichardt, 1941 and *Myrmetes* Marseul, 1862 they were placed near the root of the tree; this position was further confirmed by the ongoing molecular studies (Lackner, unpublished).

**Key to Australopacific species of *Tomogenius* Marseul, 1862**

**Table:** 

1(2)	Elytra without short hooked appendix between fourth dorsal and sutural striae (Fig. 716)	***Tomogenius papuaensis* Gomy, 2007** (New Guinea)
2(1)	Elytra with short hooked appendix between fourth dorsal and sutural striae (Fig. 698) (species from Australia and New Zealand)
3(4)	Two large foveae situated at the apex of the prosternal process widely separated from each other (Fig. 622)	***Tomogenius australis* Dahlgren, 1976** (New Zealand)
4(3)	Two large foveae situated at the apex of the prosternal process united apically by a thin ‘bridge’ (Fig. 642)
5(6)	Larger species, PEL up to 4.40 mm; first dorsal elytral stria missing (Fig. 660)	***Tomogenius kuscheli* Dahlgren, 1976** (New Zealand)
6(5)	Smaller species, PEL up to 4.10 mm; first dorsal elytral stria always present (Fig. 638)
7(8)	Prosternum, meso- and metaventrite of male with distinct dense long setae (Figs 642); carinal prosternal striae distinctly divergent apically (Fig. 642); prosternal foveae large (Fig. 642)	***Tomogenius incisus* (Erichson, 1842)** (Australia)
8(7)	Meso- and metaventrite of male without long dense setae, at most there are short and thin setae; carinal prosternal striae only faintly divergent apically (Fig. 684); prosternal foveae small to medium-sized (Fig. 684)
9(10)	Meso- and metaventrite of male with short thin setae; light brown species (Fig. 698)	***Tomogenius motocola* Mazur, 1990** (Australia)
10(9)	Meso- and metaventrite of male never with setae; dark brown or black species with blueish tinge
11(12)	Punctures on apical half of elytra distinctly aciculate, dark brown species (Fig. 679)	***Tomogenius latipes* (Broun, 1881)** (New Zealand)
12 (11)	Punctures on apical half of elytra not aciculate, bi-colored species: pronotum dark brown, elytra black with faint blueish luster (Fig. 732)	***Tomogenius ripicola* (Marseul, 1870)** (Australia)

#### Tomogenius australis

Taxon classificationAnimaliaColeopteraHisteridae

Dahlgren, 1976

Figs 619 , 620–628 , 629–637 , 766

Tomogenius australis Dahlgren, 1976: 409, fig. 22C, F.

##### Type locality.

New Zealand: Motunau Island.

##### Type material examined.

*Tomogenius australis* Dahlgren, 1976: holotype, ♂, terminalia extracted and dismembered and glued to the same mounting card as the specimen, right metatarsus missing, with following labels: “Motanau Is. / 23.10.[19]63 / In Petrel burrow / B.A. Tunncliffe” (written); followed by: “*Tomogenius* / n.sp. 2” (written-printed); followed by: “HOLOTYPE / TOMOGENIUS AUSTRALIS / G. DAHLGREN / 30.12.1975” (written); followed by: “Entomology / Division / D.S.I.R. / New Zealand” (golden label, printed); followed by: “HOLOTYPE” (bright red label, printed); followed by: “NZ Arthropod Collection / barcode / NZAC04039485” (printed) (NZAC). Paratype, ♀, side-mounted on a triangular card, with following labels: “Motanau Is. / 22.10.[19]63 / In Petrel burrow / B.A. Tunncliffe” (written); followed by: “PARATYPE / TOMOGENIUS AUSTRALIS / G. DAHLGREN / 30.12.1975” (written); followed by: “*Tomogenius australis* / Dahlgren, 1976 / (PARATYPE) / Det. S. E. Thorpe, 2002” (printed); followed by: “Entomology / Division / D.S.I.R. / New Zealand” (golden label, printed); followed by: “09-084” (yellow, pencil-written label added by the senior author) (NZAC).

##### Additional material examined.

New Zealand: 1 spec., Motanau Island, 23.x.1963, G. Tunncliffe leg., in Petrel burrow (MNHN, coll. Thérond).

##### Biology.

Found in a petrel burrow.

##### Distribution.

New Zealand, South Island (NC), Motunau Island (Fig. 766).

##### Remarks.

This species is most similar to *T. kuscheli*, especially by the structure of prosternal keel, elytral punctation, and the doubled first dorsal elytral stria (compare Figs 622 and 665 or 619 and 660). *T. australis* is known only from the type series collected in a petrel burrow on Motunau Island, South Island, New Zealand. Although the original label reads “Motanau”, the correct name of the island is “Motunau”. Motunau Island is an important site for seabirds.

##### Re-description.

Body length: PEL: 3.75–4.10 mm; EL: 2.40–2.65 mm; APW: 1.25–1.60 mm; PPW: 2.50–2.60 mm; EW: 3.00–3.25 mm.

Body (Fig. 619) ovoid, moderately convex, pronotum narrower than elytra; cuticle without metallic luster, castaneous to dark brown, pronotum darker than elytra, almost black; legs, antennae and mouthparts rufous-brown.

**Figures 620–628.**
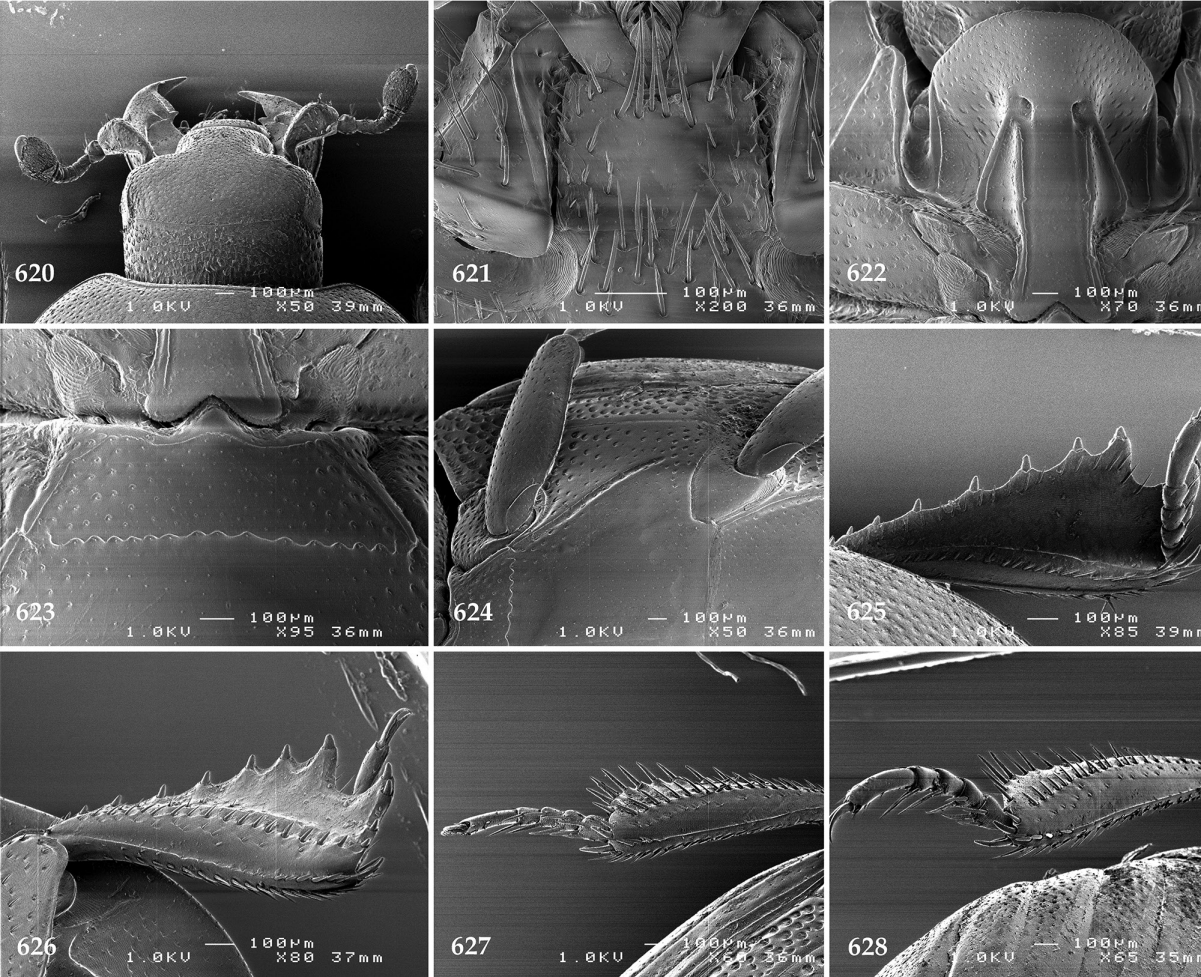
**620** *Tomogenius australis* Dahlgren, 1976 head, dorsal view **621** mentum, ventral view **622** prosternum **623** mesoventrite **624** lateral disc of metaventrite + metepisternum **625** protibia, dorsal view **626** ditto, ventral view **627** mesotibia, ventral view **628** metatibia, ventral view.

Antennal scape (Fig. 620) not particularly thickened, lower margin carinate, with few short setae; club rather oval, entirely covered in dense short sensilla, intermingled with sparser longer erect sensilla; sensory structures of antennal club not examined.

Mandibles finely microscopically punctate, with rounded outer margin, acutely pointed, sub-apical tooth on inner margin of left mandible rather small, obtuse; labrum slightly convex with indistinct median costiform elevation, not depressed medially, with several punctures (especially on basal half) each labral pit with a single moderately long labral seta; terminal labial palpomere elongated, its width about one-fourth its length; mentum (Fig. 621) sub-trapezoid, anterior angles slightly produced, anterior margin with a deep median emargination, surface around it with five tightly spaced long setae, lateral margins with a single one row of much shorter sparse ramose setae, disc with few scattered setae, median part of mentum almost smooth; cardo of maxilla with few short setae on lateral margin; stipes triangular, with seven setae; terminal maxillary palpomere elongated, its width about one-fourth its length, about three times as long as penultimate palpomere; rest of mouthparts not examined.

Clypeus (Fig. 620) large, rectangular, even, rounded laterally, with sparse fine punctures, separated by several times their diameter; frontal and supraorbital striae absent; frontal disc (Fig. 620) with sparse fine round punctures similar to those of clypeus; eyes flattened, visible from above.

Pronotal sides (Fig. 619) feebly convergent anteriorly, apical angles obtuse, marginal pronotal stria complete, thin, slightly carinate, somewhat weakened behind head; disc entirely with punctation, becoming coarser and denser laterally where punctures are separated by less than their diameter, medially becoming finer and sparser separated by several times their diameter; along pronotal base a triple row of larger punctures present; pronotal hypomeron glabrous; scutellum very small.

Elytral epipleura impunctate; marginal epipleural stria double, both striae well impressed, complete; marginal elytral stria well impressed, continuous along elytral apex as weakened but complete apical elytral stria; humeral elytral stria well impressed on basal third, surface around it striolate; inner subhumeral stria absent; elytral disc with four dorsal elytral striae 1–4, first the shortest, only as long as humeral elytral stria (situated near it) and in the case of the female paratype continued as a vague intermittent impression, second to fourth striae well impressed, impunctate, slightly surpassing elytral half; between fourth dorsal elytral and sutural striae a characteristic hooked appendix present; sutural elytral stria present on basal tenth as a short fragment. Elytral disc on basal half (roughly) with punctures separated several times their diameter (fourth elytral interval almost impunctate), on apical half (roughly) punctures becoming larger and denser, but still rather sparse; on apical third punctures strongly aciculate, especially laterally.

Propygidium transverse, about four times as broad as long, partially covered by elytra, with dense and coarse punctures separated by less than their diameter; pygidium with similar round punctures, becoming sparser and finer towards apex.

Anterior margin of median portion of prosternum (Fig. 622) rounded; marginal prosternal stria present only laterally; prosternal process between carinal prosternal striae flattened, broad, anterior third elevated, slightly projecting, laterally with sparse shallow oval punctures, intermingled with alutaceous microsculpture, apically and between carinal prosternal striae punctures much smaller but denser; carinal prosternal striae (Fig. 622) parallel, terminating before large and deep widely separated apical foveae; lateral prosternal striae carinate, convergent anteriorly, apically terminating in apical foveae.

Discal marginal mesoventral stria (Fig. 623) well impressed, somewhat carinate, anteriorly medially projected; disc flattened, laterally with deep large punctures separated several times their diameter becoming finer and even sparser; meso-metaventral suture distinct, meso-metaventral sutural stria well impressed, undulate.

Intercoxal disc of metaventrite in males with small narrow longitudinal furrow before hind margin, in females without such furrow, slightly convex, finely and sparsely punctate, punctures in apical and basal corners becoming larger and coarser. Lateral metaventral stria well impressed, carinate, almost straight, not reaching metacoxa; lateral disc of metaventrite (Fig. 624) flattened, with sparse shallow punctures; metepisternum + fused metepimeron (Fig. 624) evenly with much coarser and denser punctation; lateral metepisternal stria present, deeply impressed and almost complete.

Intercoxal disc of first abdominal ventrite completely striate laterally; surface of disc on basal half with scattered punctation, punctures becoming sparser and finer apically.

Protibia (Fig. 625) flattened and somewhat dilated, outer margin with six low triangular teeth followed by three tiny denticles; first two teeth conspicuously larger than the remaining ones (and separated by a wide gap) that are diminishing in size in proximal direction; teeth topped by short denticle; first (distal-most) tooth topped by double denticle. Setae of outer row short, moderately dense; setae of median row similarly dense and regular, slightly shorter than those of outer row; protarsal groove shallow; anterior protibial stria complete, costate; two thin, rather long tarsal denticles present apically; protibial spur moderately long, not particularly bent, growing out from apical protibial margin; apical margin of protibia posteriorly with two tiny apical denticles; outer part of posterior surface of protibia (Fig. 626) smooth, well divided from median part of posterior surface by ridge like stria; median part of posterior surface with two dense rows of minuscule setae; posterior protibial stria complete with tightly-spaced short and stout denticles near apical margin; inner margin with double row of short setae.

Mesotibia slender, outer margin with a single row of dense thin denticles growing in size apically; setae of outer row regular, rather dense but short, growing somewhat longer apically; setae of median row irregular, much shorter than those of outer row; posterior mesotibial stria complete; anterior surface of mesotibia with another row of denticles shorter, but similar to those of outer margin; a row of microscopic setae situated below it; anterior mesotibial stria (Fig. 627) complete, terminating in three tiny inner anterior denticles; mesotibial spur stout, short; apical margin with two tiny denticles; mesotarsus shorter than mesotibia; claws of apical tarsomere bent, longer than half its length; metatibia (Fig. 628) basically similar to mesotibia, but denticles of outer margin sparser and finer than those of mesotibia.

**Figures 629–637.**
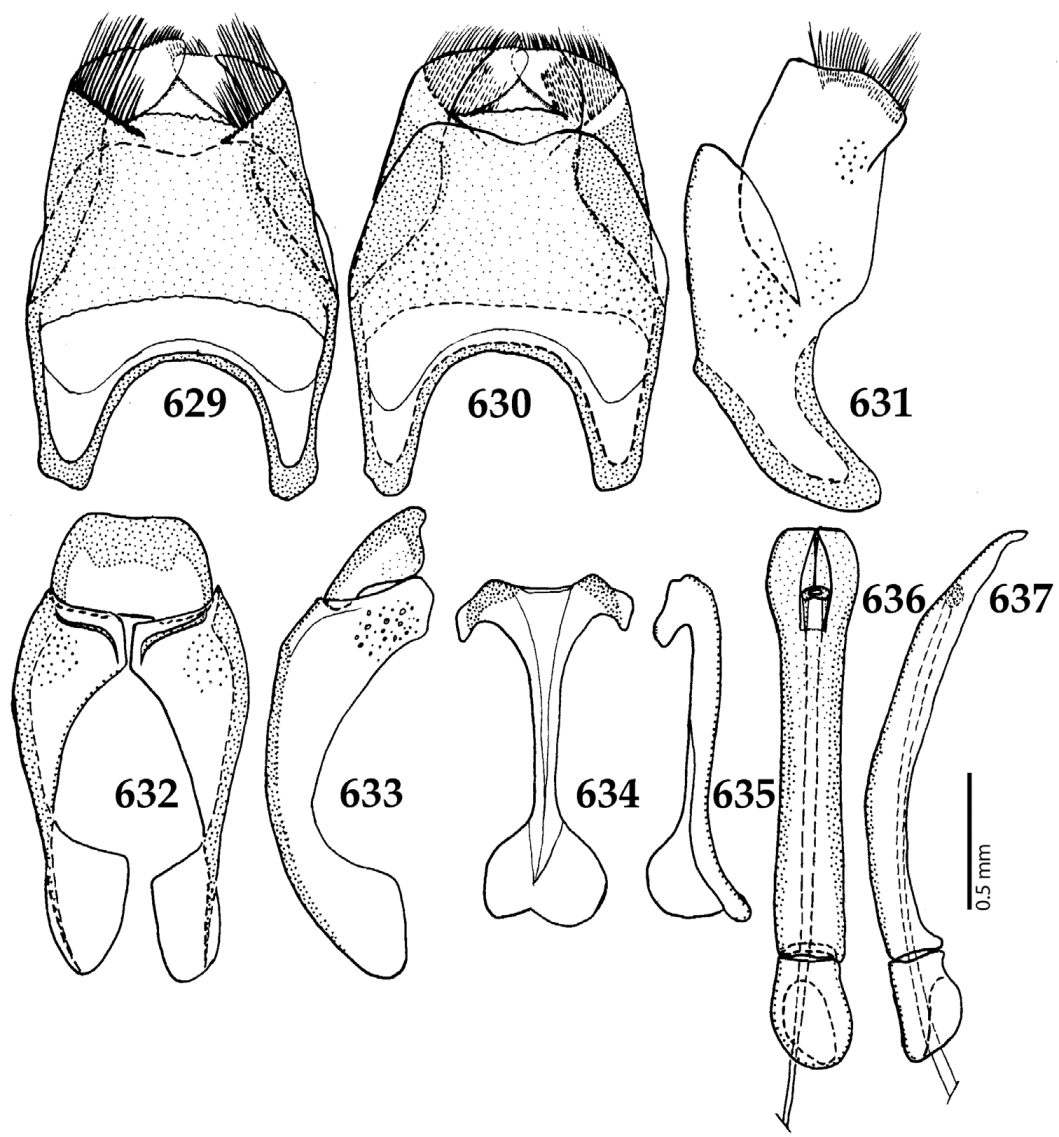
**629** *Tomogenius australis* Dahlgren, 1976 male terminalia: 8^th^ sternite + 8^th^ tergite, ventral view **630** ditto, dorsal view **631** ditto, lateral view **632** male terminalia: 9^th^ + 10^th^ tergites, dorsal view **633** ditto, lateral view **634** male terminalia: spiculum gastrale, ventral view **635** ditto, lateral view **636** male terminalia: aedeagus, dorsal view **637** ditto, lateral view.

Male genitalia. Eighth sternite (Figs 629–630) completely fused medially; apically with a single row of tightly spaced long setae diminishing in size mesally; vela present, apically with another row of shorter but similarly dense setae; eighth tergite and eighth sternite fused laterally (Fig. 631); eighth tergite medially inwardly arcuate. Ninth tergite (Figs 632–633) longitudinally divided medially; outer margin of tenth tergite slightly arcuate inwardly, almost straight; spiculum gastrale (Figs 634–635) abruptly dilated on apical third, basal end abruptly dilated, cordate. Aedeagus (Figs 636–637) slender, almost parallel-sided, slightly dilated on apical fifth and thence convergent apically; apex of aedeagus not pointed, straight; basal piece of aedeagus short, ratio of its length : length of parameres 1:5; parameres fused almost along their entire length, only on apical fifth with an opening for median lobe; aedeagus slightly curved from lateral view (Fig. 637).

#### Tomogenius incisus

Taxon classificationAnimaliaColeopteraHisteridae

(Erichson, 1842)

Figs 638 , 639–647 , 648–650 , 651–659 , 767

Saprinus incisus Erichson, 1842: 152.
Saprinus incisisternus Marseul, 1862: 497, plate 12, fig. 1 (emend.).

##### Type locality.

Australia: Tasmania.

**Figure 638.**
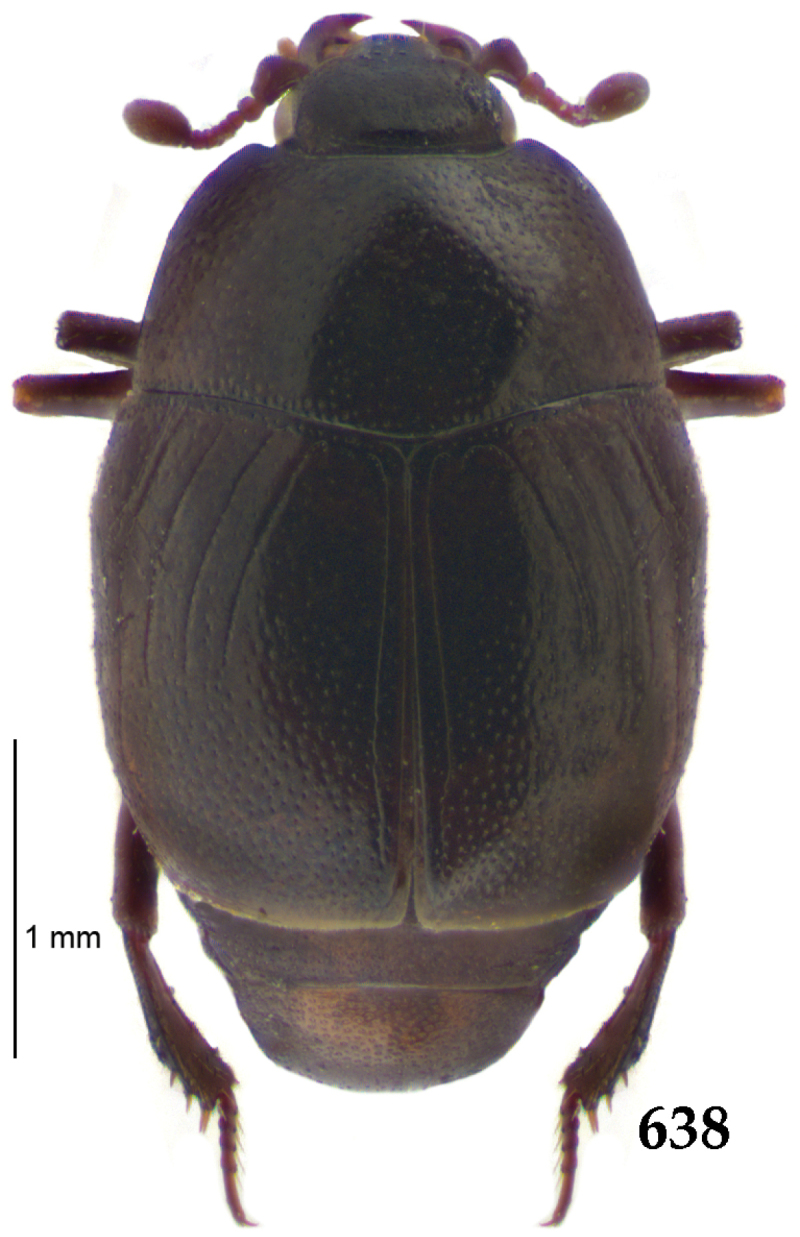
*Tomogenius incisus* (Erichson, 1842) habitus, dorsal view.

##### Type material examined.

*Saprinus incisus* Erichson, 1842: Lectotype, present designation: ♂, right mid-leg missing, pinned, with genitalia extracted, glued to the mounting card under specimen; card with genitalia bears pencil-written inscription on its underside: “INCISUS / TYPE” apparently written there by Dahlgren; followed by: “Terra v Diemen / Schayer / Nr. 49180” (beige, written label); followed by: “Type” (brick-red label, printed); followed by: “Hist. Coll. Coleoptera / Nr. 49180 / Saprinus incisus Er. x / Terra v. Diemen, Schayer / Zool. Mus. Berlin” (violet, black margined label, printed); followed by: “Saprinus incisus / Erichson, 1842 / LECTOTYPE / des. T. Lackner 2014” (red label, written) (ZMHUB). Paralectotype, present designation, ♀, both metatarsi missing, with printed label: “49180”; followed by: “incisus Er. / Van Diem. Schayer” (dark-beige label, written); followed by the two labels of the lectotype, with additional paralectotype label (ZMHUB). This species was described from unknown number of specimens and the lectotype designation fixes the identity of the species.

*Saprinus incisisternus* Marseul, 1862: 497: Lectotype, present designation: ♂ pinned, both protarsi, left mesotarsus and left hind leg missing, with tiny rectangular dark blue label, followed by the following labels: “119a Saprinus / incisus Er. / v Diemen / illegible” (written); followed by: “Gnathoncus / incisisternus m. / incisus Er./ V. Diemen / T. Er. Band 60??” (round pink label, written); followed by: “MUSEUM PARIS / COLL. / DE MARSEUL 1890” (pink label, printed); followed by: “Saprinus / incisisternus / Marseul, 1862 / LECTOTYPE 2014 / des. T. Lackner” (red label, written) (MNHN). This species was described from an unknown number of specimens and we therefore designate a lectotype to formally fix its taxonomic identity.

##### Additional material examined.

AUSTRALIA. Queensland: 1 spec., Cape York, without further data (BMNH); 1 spec., Tallebudgera valley falls area, 150 m, 28°14'S, 153°19'E, 22.iv.2001, D.J. Cook leg. (NCB); 2 specs., ditto (QM); 2 specs., ditto, but 18.vi.2000 (QM); 7 specs., ditto, but 28.i.–21.v.1998, pitfall in bat guano (QM); 10 specs., ditto, but 21.v.–26.ix.1998 (QM); 4 spec, ditto, but 11.xi.1997–28.i.1998 (QM); 7 specs., Keniff Cave, Mt. Mofatt NP, 25°09'S, 148°01'E, 26.ix.1995, G.B. Monteith (QM); 4 specs., Finchhatton, Doolemai Cave, 22.v.1982, M. Crowther leg. (QM); 1 spec., 3 km W of Bangalee Beach, 23°04'S, 150°44'E, open forest, fungus pit, 20 m, D. Cook leg. (QM); 1 spec., Mt. Etna, Johannsens mine, 15.v.1986, ex ghost bat guano, E. Holm leg. (ANIC). New South Wales: 1 spec., Wee Jasper, Church Cave, 27.viii.1985, ex bat droppings; 2 specs., Deua National Park, Deua Cave, 5.iv.1986, ex bat guano (ANIC); 9 ♂♂ & 8 ♀♀, 20 km W of Kempsey, Yessabah Cave, 14.i.1987, E. Holm leg., bat guano (ANIC); 2 ♀♀, Glass Cave, Wombeyan, 21.ii.1965, I.D. Wood (ANIC). Australian Capital Territory: 1 ♂, N Canberra, 7.iii.1970, K.R. Pullen leg. (ANIC). Victoria: 1 ♂ & 3 ♀♀, Novgun’s Cave, 3.xi.1964, K.G. Simpson leg., guano (ANIC); 1 ♀, Guano Cave, Lake Gilleur (?), 8.xii.1962, R.J. Edge (ANIC). South Australia: 1 ♂ & 1 ♀, Naracoorte, 36°58'S, 140°45'E, 8.xi.1987, A. Spate leg., ex bat guano (ANIC).

##### Biology.

Found mostly in caves and in bat guano where it presumably preys on larvae of small arthropods.

##### Distribution.

Australia: Victoria, South Australia, Australian Capital Territory, New South Wales, Tasmania, and Queensland (Fig. 767).

##### Remarks.

This is a sexually dimorphic species, with males having setose prosternites and meso- and metaventrites. The species is widely distributed and variable in size, color and punctation across Australia.

##### Re-description.

Body length: PEL: 2.15–3.50 mm; APW: 0.85–1.25 mm; PPW: 1.50–2.50 mm; EL: 1.40–2.25 mm; EW: 1.65–2.75 mm.

Body (Fig. 638) ovoid, moderately convex from above, underside slightly flattened, cuticle castaneous to dark brown, almost black without metallic luster; legs and body appendages similarly colored.

**Figures 639–647.**
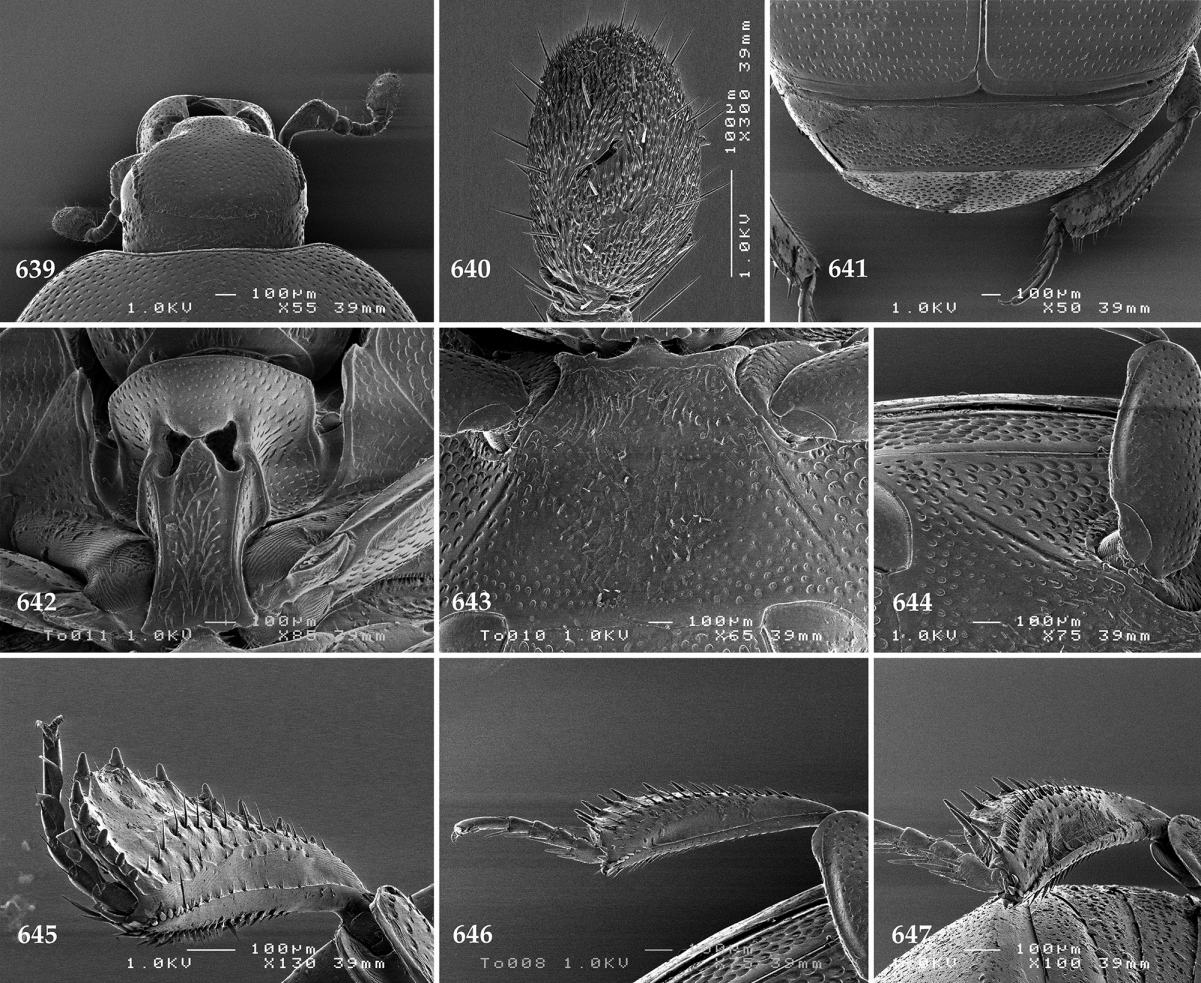
**639** *Tomogenius incisus* (Erichson, 1842) head, dorsal view **640** antennal club, ventral view **641** propygidium + pygidium **642** prosternum **643** mesoventrite + metaventrite (male) **644** lateral disc of metaventrite + metepisternum **645** protibia, ventral view **646** mesotibia, ventral view **647** metatibia, ventral view.

Antennal scape (Fig. 639) not particularly thickened, with few short setae; club (Fig. 640) rather large, oval, ventrally with two slit-like pits, entirely covered in dense short sensilla, intermingled with sparse longer erect sensilla; sensory structures of antennal club (Fig. 650) in form of two dorsal oval sensory areas and two ventral slit like pits and large single ball-like vesicle situated in middle of club.

**Figures 648–650.**
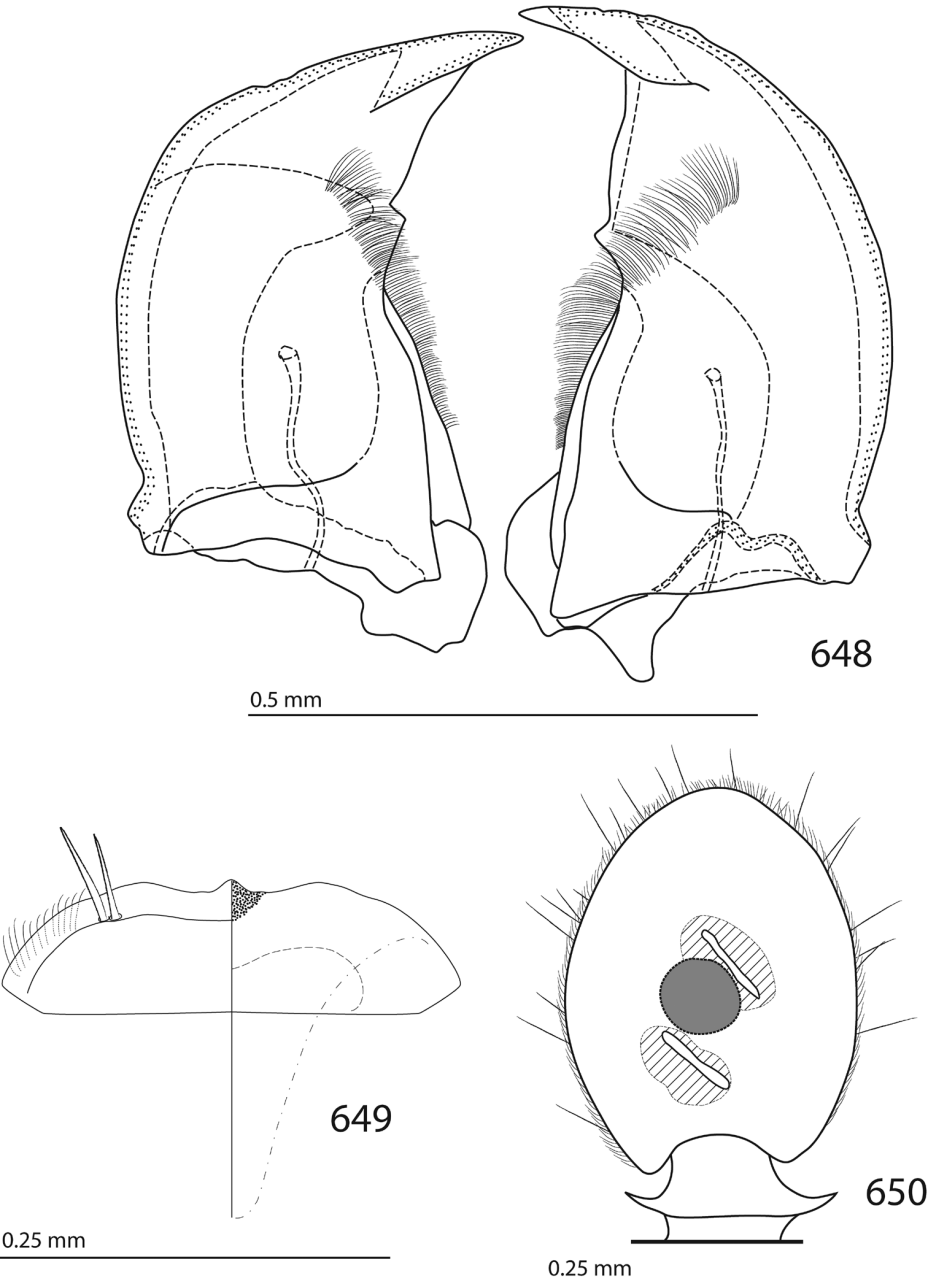
**648** *Tomogenius incisus* (Erichson, 1842) mandibles, dorsal view **649** labrum: left half depicting dorsal view and right half depicting epipharynx **650** antennal club, ventral view showing sensory structures of the antenna.

Mandibles (Fig. 648) with rounded outer margin, acutely pointed, sub-apical tooth on inner margin of left mandible very small, almost inconspicuous; labrum (Fig. 649) slightly convex dorsally, sparsely punctate, approximately three times as wide as long, with small median projection; labral fold significantly small; setae of lateral fringe short; labrum with two moderately long labral setae; terminal labial palpomere elongated, its width about one-fourth its length; palpal organ present on both labial and maxillary palpi; mentum sub-trapezoid, anterior angles slightly produced, anterior margin with a shallow median notch, surface around it with single long seta, lateral margins with a single one row of much shorter sparse ramose setae, disc with few scattered setae; cardo of maxilla with few short setae on lateral margin; stipes triangular, with three short setae; lacinia with lacinial hook; terminal maxillary palpomere elongated, its width about one-fourth its length, about three times as long as penultimate palpomere.

Clypeus (Fig. 639) large, rectangular, rounded laterally, with sparse fine punctures, separated by several times their diameter; frontal and supraorbital striae absent; frontal disc (Fig. 639) with sparse fine round punctures; eyes convex, well visible from above.

Pronotal sides (Fig. 638) feebly convergent anteriorly, apical angles produced, marginal pronotal stria complete, thin, slightly carinate, somewhat weakened behind head; disc entirely with deep, round punctation, becoming coarser and denser laterally, medially punctures separated by several times their diameter; pronotal hypomeron glabrous; scutellum very small.

Elytral epipleura with scattered punctures of various sizes; marginal epipleural stria double, both striae weakly impressed but complete; marginal elytral stria well impressed, continuous along elytral apex as apical elytral stria, stopping in middle of elytral apical margin; humeral elytral stria well impressed on basal third, surface around it striolate; inner subhumeral stria present medially, short; elytral disc with four dorsal elytral striae 1–4, first the longest, reaching about two-thirds of elytral length apically, occasionally longer, second to fourth striae well impressed, only slightly shorter than first; between fourth dorsal elytral and sutural striae a characteristic hooked appendix present; sutural elytral stria almost complete, usually reaching as far as 5/6 of elytral length apically. Entire elytral disc punctate, on basal half (roughly) punctures finer and sparser separated by about their own to twice to three times their diameter (occasionally space between base of fourth dorsal elytral and sutural striae almost smooth), on apical half (roughly) punctures larger and denser, separated approximately by their diameter; punctures near extreme elytral apex with minuscule striolae among them.

Propygidium (Fig. 641) transverse, about four times as broad as long, completely exposed, with dense and coarse punctures separated by less than their diameter, intermingled with tiny scattered punctures; pygidium (Fig. 641) with similar round punctures, separated by about their diameter, becoming sparser and finer towards apex.

Anterior margin of median portion of prosternum (Fig. 642) rounded; marginal prosternal stria present only laterally; prosternal process flattened, broad, laterally with sparse oval punctures, intermingled with alutaceous microsculpture, in males dorsally space between carinal prosternal striae with dense short setae, in females prosternum asetose; carinal prosternal striae (Fig. 642) only slightly divergent anteriorly, terminating near large and deep apical foveae separated by apex of prosternal prosternal process; lateral prosternal striae carinate, slightly convergent anteriorly, attaining apices of carinal prosternal striae. Lateral costa of antennal groove reaching prosternal process, but not elevated (unlike in *Gnathoncus*).

Anterior margin of mesoventrite (Fig. 643) almost straight; discal marginal mesoventral stria well impressed, somewhat carinate; disc flattened, with round punctures separated by about their diameter, in males disc of mesoventrite with moderately long dense yellow setae, mesoventrite in females asetose; meso-metaventral suture indistinct, meso-metaventral sutural stria marked as a row of large punctures.

Intercoxal disc of metaventrite (Fig. 643) in males medially with large setose depression; metaventrite of females asetose, with only slight median longitudinal depression. Disc of metaventrite in both sexes punctate, medially punctures finer and sparser, becoming larger and coarser along lateral margin. Lateral metaventral stria well impressed, carinate, almost straight, not reaching metacoxa; lateral disc of metaventrite (Fig. 644) flattened, with round shallow large punctures fringed with microscopic setae; metepisternum + fused metepimeron (Fig. 644) evenly with much coarser and denser punctation, punctures without setae; lateral metepisternal stria present, deeply impressed and almost complete.

Intercoxal disc of first abdominal ventrite with lateral depressions, almost completely striate laterally; surface of disc with scattered oblong punctation, punctures becoming sparser and finer medially (occasionally almost smooth).

Protibia (Fig. 645) flattened and somewhat dilated, outer margin almost without teeth, with seven–eight widely spaced short denticles; setae of outer row short, moderately dense; setae of median row similarly dense and regular, but even shorter than those of outer row; protarsal groove shallow; anterior protibial stria complete, costate; two thin, rather long tarsal denticles present apically; protibial spur short, straight, growing out from apical protibial margin; apical margin of protibia posteriorly with three to four tiny apical denticles; outer part of posterior surface of protibia (Fig. 645) finely imbricate, with a row of short setae; median part of posterior surface with additional two rows of minuscule setae; posterior protibial stria complete, with scattered minuscule setae turning into five tightly-spaced short and stout denticles near apical margin; inner margin with double row of short lamellate setae.

Mesotibia (Fig. 646) slender, outer margin with a single row of dense thin denticles growing in size apically; setae of outer row sparse, regular, rather dense but short, growing somewhat longer apically; setae of median row irregular, much shorter than those of outer row; posterior mesotibial stria complete; anterior surface of mesotibia with dense row of well sclerotized short setae, with another similar row of much shorter and finer setae situated below it; anterior mesotibial stria complete, terminating in three tiny inner anterior denticles; mesotibial spur stout, short; apical margin with two tiny denticles; mesotarsus shorter than mesotibia; claws of apical tarsomere about half its length; metatibia (Fig. 647) basically similar to mesotibia, but denticles of outer margin much sparser than those of mesotibia; claws of apical tarsomere somewhat shorter, about one-third its length.

**Figures 651–659.**
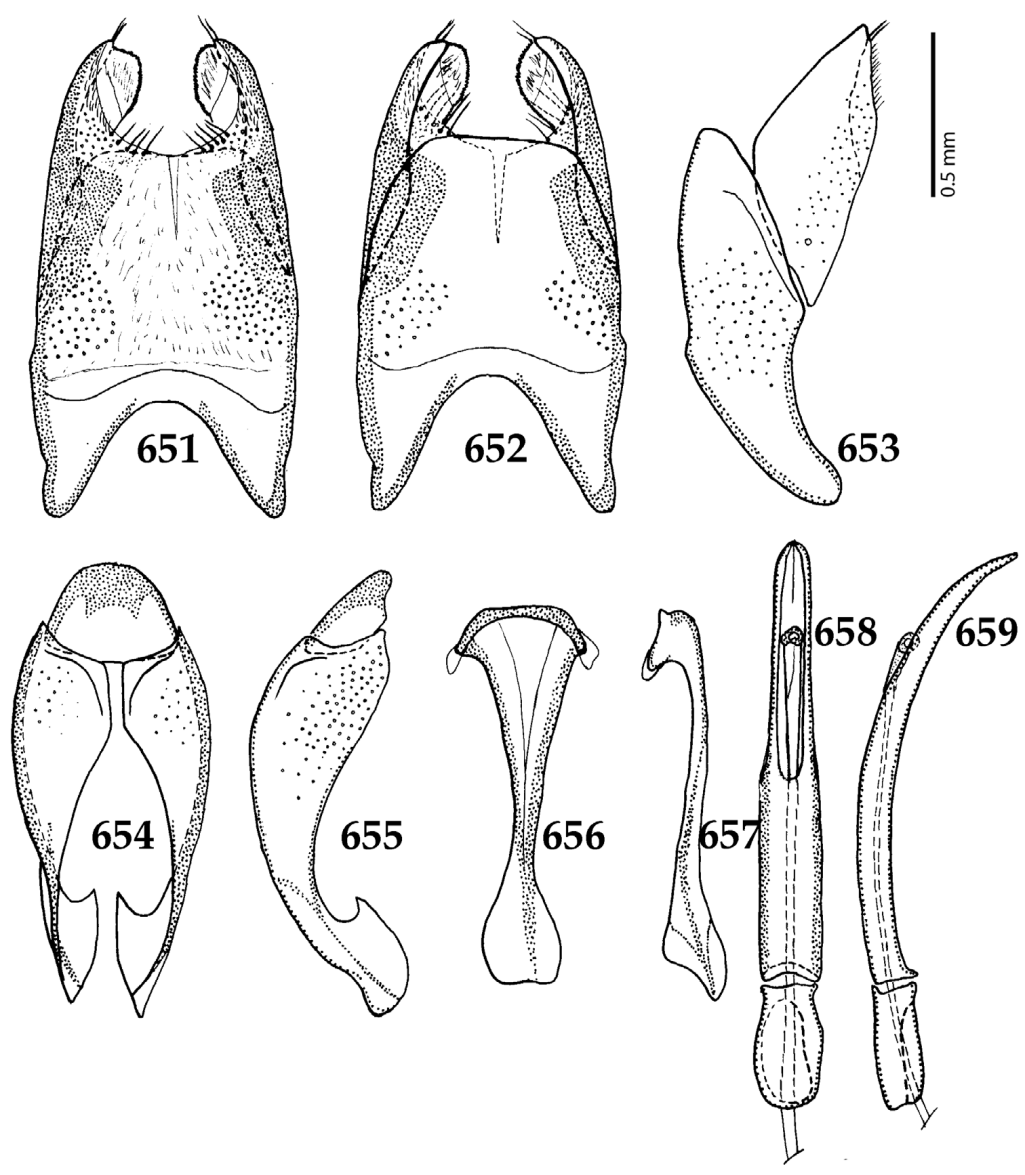
**651** *Tomogenius incisus* (Erichson, 1842) male terminalia: 8^th^ sternite + 8^th^ tergite, ventral view **652** ditto, dorsal view **653** ditto, lateral view **654** male terminalia: 9^th^ + 10^th^ tergites, dorsal view **655** ditto, lateral view **656** male terminalia: spiculum gastrale, ventral view **657** ditto, lateral view **658** male terminalia: aedeagus, dorsal view **659** ditto, lateral view.

Male genitalia. Eighth sternite (Figs 651–652) apically longitudinally separated, fused on basal half; vela with sparse microscopic setae, apically with two longer setae; eighth tergite and eighth sternite not fused laterally (Fig. 653). Ninth tergite (Figs 654–655) longitudinally divided medially; spiculum gastrale (Figs 656–657) gradually dilated in most of apical half, basal end slightly dilated, spoon-like. Aedeagus (Figs 658–659) slender, conspicuously slender on apical half; basal piece of aedeagus short, ratio of its length : length of parameres 1 : 4; parameres fused along their basal half; aedeagus curved from lateral view (Fig. 659).

#### Tomogenius kuscheli

Taxon classificationAnimaliaColeopteraHisteridae

Dahlgren, 1976

Figs 660 , 661–669 , 670–678 , 766

Tomogenius kuscheli Dahlgren, 1976: 409, fig. 22B.

##### Type locality.

New Zealand: Stephens Island.

**Figure 660.**
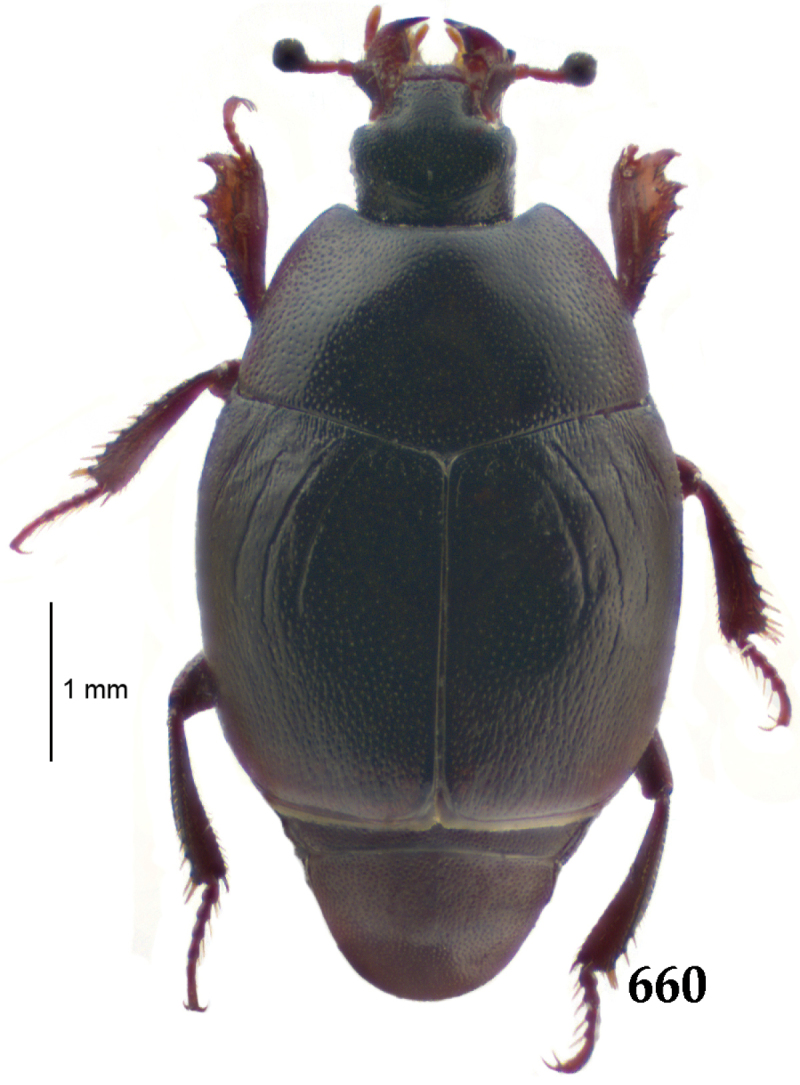
*Tomogenius kuscheli* Dahlgren, 1976 habitus, dorsal view.

##### Type material examined.

*Tomogenius kuscheli* Dahlgren, 1976: holotype, ♀, side-mounted on a triangular card, both protarsi broken off, with the following labels: “Stephens I / Nelson 16 Feb. 76 / G.W. Ramsay” (printed); followed by: “Litter” (printed); followed by: “Tomogenius / sp. n. 1 / Kuschel det. 1974” (written-printed); followed by: “HOLOTYPE / TOMOGENIUS / KUSCHELI / G. DAHLGREN / 30.12.1975” (written); followed by: “HOLOTYPE” (red label, printed); followed by: “Entomology / Division / D.S.I.R / New Zealand” (olive label, printed) (NZAC).

##### Additional material examined.

NEW ZEALAND. South Island. SD: 1 ♀, Motuara Island, 23.i.1971, J. R. Jackson (ex *Puffinus gavia* nest) (AMNZ); 3 ♂♂ & 2 ♀♀, ditto, but LUNZ (1 ♂ in coll. TLAN); 1 spec., Stephens Island, Keepers Bush, 20.ii.1994, J.W.M. Marris (ex petrel burrow) (LUNZ).

##### Biology.

Found in the burrow of a petrel and a shearwater (*Puffinus gavia*).

##### Distribution.

New Zealand, South Island (SD) Stephens and Motuara Islands (Fig. 766).

##### Remarks.

Several specimens from the Motuara Island have a short stria next to humeral elytral stria, which may be homologous with the first dorsal elytral stria. However, in the Saprininae, often the humeral elytral stria is doubled and it is therefore difficult to determine exact homology of this stria. Its basal end is not hooked as in the other dorsal elytral striae; it is less deeply impressed and is not on par with the humeral elytral stria regarding its length. This species is most similar to *T. australis*, especially by the structure of prosternal keel, elytral punctation, or the doubled first striae (compare Figs 622 and 665 or 619 and 660).

##### Re-description.

Body length: PEL: 4.00–4.40 mm; EL: 2.25–2.75 mm; APW: 1.25–1.50 mm; PPW: 2.75–3.00 mm; EW: 3.25–3.50 mm.

Body (Fig. 660) ovoid, moderately convex, pronotum narrower than elytra; cuticle without metallic luster, dark brown to black; legs, antenna and mouthparts dark brown.

**Figures 661–669.**
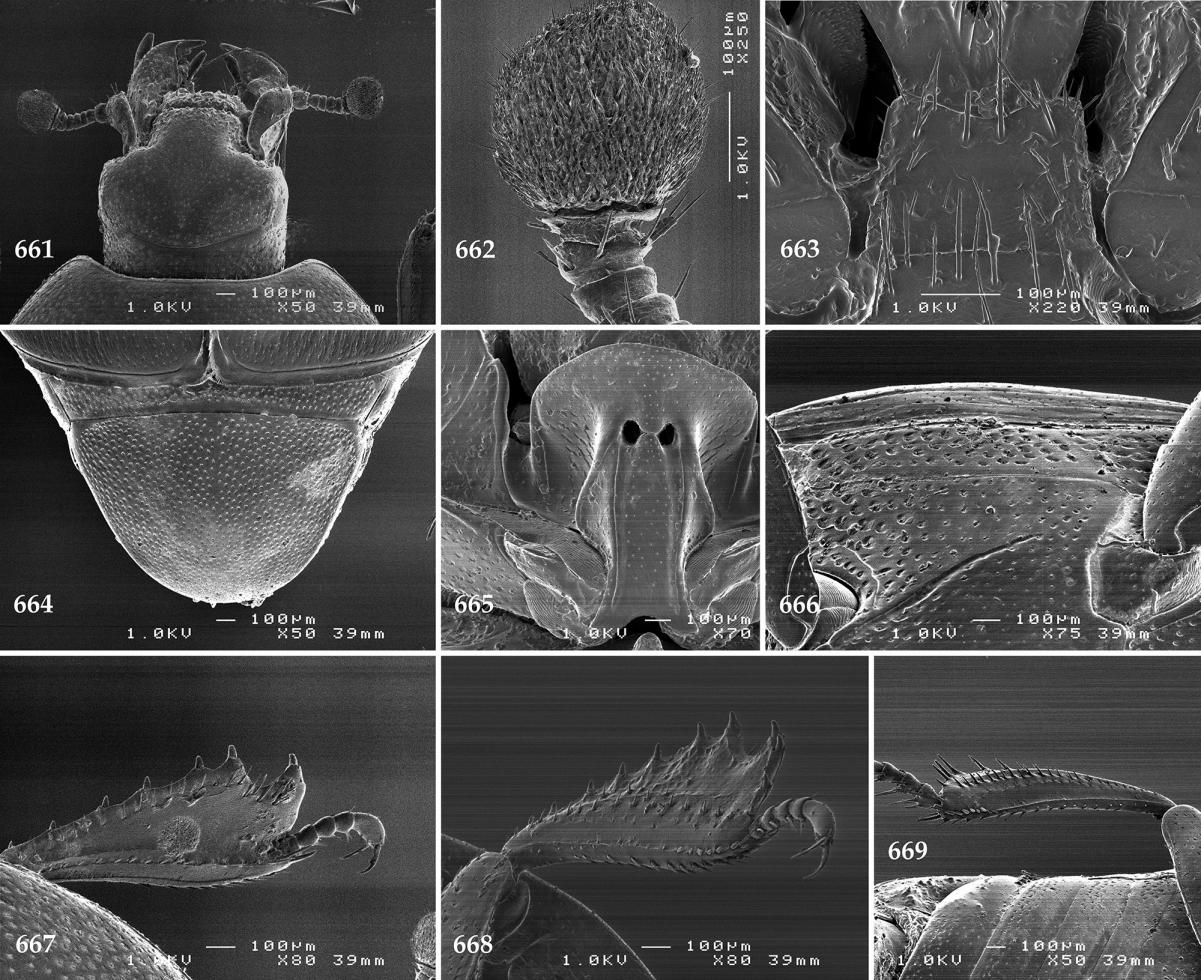
**661** *Tomogenius kuscheli* Dahlgren, 1976 head, dorsal view **662** antennal club, dorsal view **663** mentum, ventral view **664** propygidium + pygidium **665** prosternum **666** lateral disc of metaventrite + metepisternum **667** protibia, dorsal view **668** ditto, ventral view **669** metatibia, ventral view.

Antennal scape (Fig. 661) not particularly thickened, lower margin carinate, with few short setae; club (Fig. 662) rounded, rather small, entirely covered in dense short sensilla, intermingled with sparser longer erect sensilla; ventral side of antennal club with two pairs of slit-like orifices, sensory structures situated within antennal club not examined.

Mandibles finely and sparsely punctate, rounded, acutely pointed, sub-apical tooth on inner margin of left mandible rather small, obtuse; labrum slightly convex, almost even, not depressed medially, with several punctures (especially on basal half); each labral pit with two moderately long labral setae; terminal labial palpomere elongated, its width about one-fourth its length; mentum (Fig. 663) sub-trapezoid, anterior angles slightly produced, anterior margin with a deep median emargination, surface around it with several longer setae, lateral margins with a single one row of much shorter sparse ramose setae, disc with few scattered setae, median part of mentum almost smooth; cardo of maxilla with few short setae on lateral margin; stipes triangular, with several setae; terminal maxillary palpomere elongated, its width about one-fourth its length, about three times as long as penultimate palpomere; rest of mouthparts not examined.

Clypeus (Fig. 661) large, rectangular, even, rounded laterally, with very dense shallow elongate punctures, separated by less than half their diameter; frontal and supraorbital striae absent; frontal disc (Fig. 661) with punctures similar to those of clypeus, becoming more rounded and sparser posteriorly; eyes flattened, visible from above.

Pronotal sides (Fig. 660) feebly convergent anteriorly, apical angles prominent, marginal pronotal stria complete, thin, slightly carinate; disc entirely with punctation, becoming coarser and denser laterally where punctures very coarse and dense separated by less than their diameter, interspaces with isodiametric structures, medially punctures becoming finer and sparser separated by several times their diameter; along pronotal base an irregular row of larger punctures present; distinct pre-scutellar depression present; pronotal hypomeron glabrous; scutellum small.

Elytral epipleura with small punctures and irregular strioles; marginal epipleural stria double, both striae well impressed, complete; marginal elytral stria well impressed, shortly present also along elytral base, apically continuous along elytral apex as fine, complete apical elytral stria; humeral elytral stria deeply impressed on basal third (occasionally doubled), surface around it deeply striolate; inner subhumeral stria absent; elytral disc with three dorsal elytral striae 2–4, first dorsal elytral stria absent, second to fourth striae well impressed, impunctate, slightly surpassing elytral half; between fourth dorsal elytral and sutural striae a characteristic hooked appendix present; sutural elytral stria present on basal eighth as a short fragment. Elytral disc on basal half (roughly) with punctures separated several times their diameter (fourth elytral interval almost impunctate), on apical half (roughly) punctures becoming larger and denser, laterally extremely aciculate, confluent, interspaces imbricate.

Propygidium (Fig. 664) transverse, about four times as broad as long, partially covered by elytra, with dense and coarse punctures separated by less than their diameter; pygidium (Fig. 664) with similar round punctures, becoming sparser and finer towards apex.

Anterior margin of median portion of prosternum (Fig. 665) rounded; marginal prosternal stria present only laterally; prosternal process between carinal prosternal striae flattened, broad, anterior third elevated, slightly projecting, laterally with sparse shallow oval punctures, intermingled with alutaceous microsculpture, apically and between carinal prosternal striae punctures sparse and microscopic; carinal prosternal striae (Fig. 665) slightly bisinuate, terminating in large and deep apical foveae separated by apex of prosternal keel (Fig. 665); lateral prosternal striae carinate, bisinuate, convergent anteriorly, apically terminating near apical foveae.

Discal marginal mesoventral stria well impressed, somewhat carinate, anteriorly medially projected; disc flattened, laterally with deep large punctures separated several times their diameter becoming finer and even sparser, intermingled with sparse microscopic punctures; meso-metaventral suture distinct, meso-metaventral sutural stria well impressed, undulate, slightly distanced from meso-metaventral suture.

Intercoxal disc of metaventrite convex, finely and sparsely punctate, punctures in apical and basal corners becoming larger and coarser. Lateral metaventral stria well impressed, carinate, almost straight, not reaching metacoxa; lateral disc of metaventrite (Fig. 666) flattened, with sparse shallow punctures; metepisternum + fused metepimeron (Fig. 666) evenly with much coarser and denser punctation; lateral metepisternal stria absent.

Intercoxal disc of first abdominal ventrite completely striate laterally; disc laterally with dense and coarse punctation becoming sparser and finer medio-apically.

Protibia (Fig. 667) almost identical to that of *T. australis*, differing from it only by areolate outer part of posterior surface of protibia (Fig. 668) (as opposed to smooth in *T. australis*) and by the presence of two-three irregular rows of short setae on median part of posterior surface of protibia (as opposed to two dense rows of minuscule setae in *T. australis*). Meso- and metatibiae (Fig. 669) basically identical to those of *T. australis*.

**Figures 670–678.**
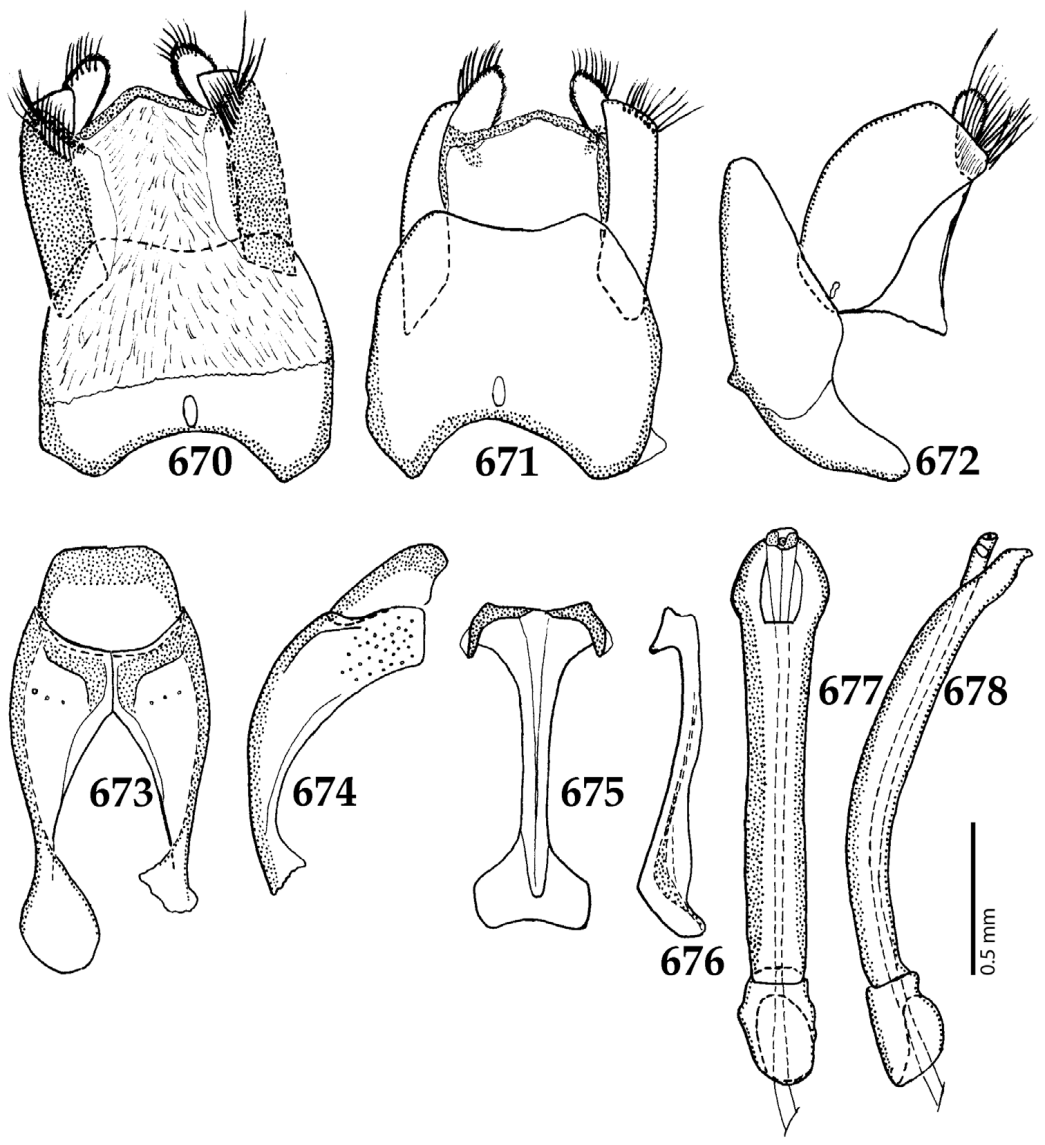
**670** *Tomogenius kuscheli* Dahlgren, 1976 male terminalia: 8^th^ sternite + 8^th^ tergite, ventral view **671** ditto, dorsal view **672** ditto, lateral view **673** male terminalia: 9^th^ + 10^th^ tergites, dorsal view **674** ditto, lateral view **675** male terminalia: spiculum gastrale, ventral view **676** ditto, lateral view **677** male terminalia: aedeagus, dorsal view **678** ditto, lateral view.

Male genitalia. Eighth sternite (Figs 670–671) widely separated medially, their apices with a tuft of long regular setae arranged in two rows; eighth tergite (Fig. 671) only slightly larger than sternite, apically faintly inwardly arcuate; basal margin strongly inwardly arcuate. Basal margin of eithth tergite medially with a curious oval structure; 8^th^ sternite and tergite not fused laterally (Fig. 672). Ninth tergite (Figs 673–674) medially narrowly separated; apical margin of tenth tergite straight. Spiculum gastrale (Figs 675–676) with almost parallel body and dilated ends: apical end strongly sclerotized; basal margin cordate. Aedeagus (Figs 677–678) almost parallel-sided, apical fifth roundly dilated; parameres fused almost along their entire length. Basal piece of aedeagus rather short, its ratio to ratio of parameres approximately 1:4.

#### Tomogenius latipes

Taxon classificationAnimaliaColeopteraHisteridae

(Broun, 1881)

Figs 679 , 680–688 , 689–697 , 766

Saprinus latipes Broun, 1881: 666.

##### Type locality.

New Zeland: Mount Arthur.

**Figure 679.**
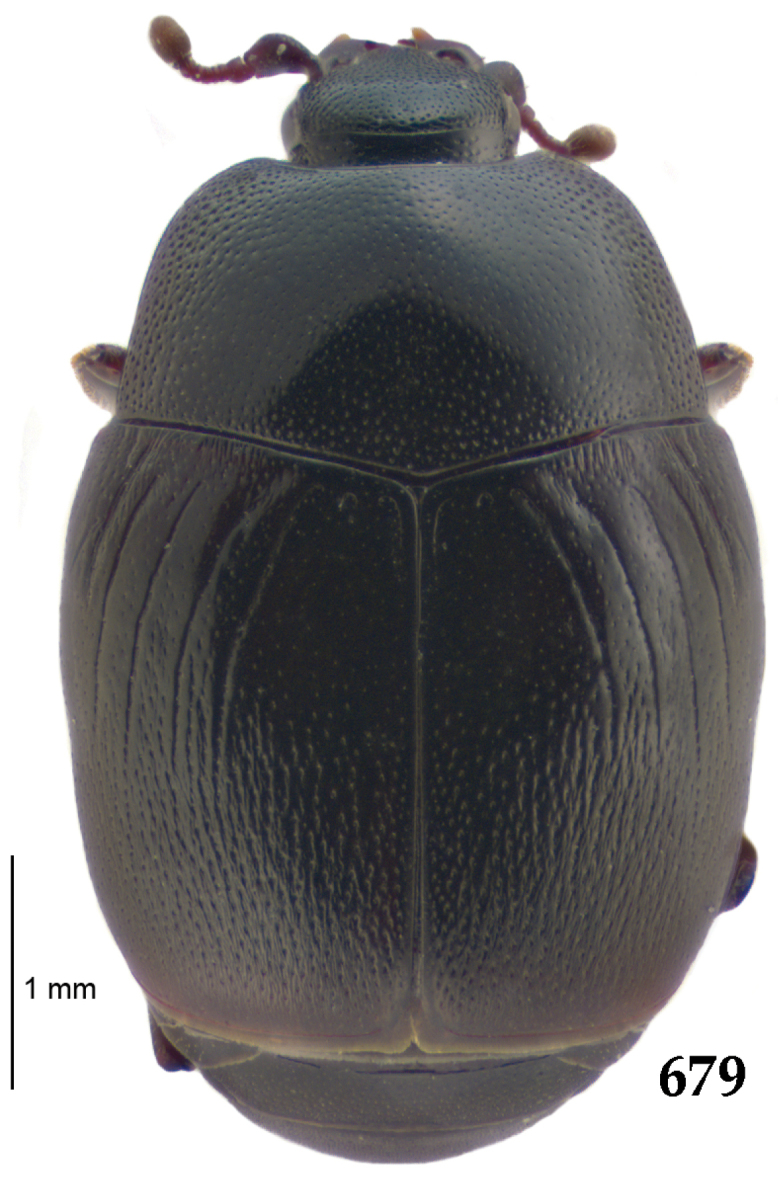
*Tomogenius latipes* (Broun, 1881) habitus, dorsal view.

##### Type material examined.

*Saprinus latipes* Broun, 1881: holotype, ♂, with genitalia extracted and glued to the mounting card, four segments of mesotarsus missing, with following labels: “Mount Arthur” (printed); followed by: “New Zealand / Broun Coll. / Brit. Mus. / 1922-482” (printed); followed by: “Saprinus / latipes” (hand-written); followed by: “1163” (light-green label, printed); followed by: “Type” (round, red-margined printed label); followed by: “Saprinus latipes / Broun, 1881 / HOLOTYPE / Des. Lackner & Leschen 2014” (written) (BMNH). This species was described based on a single specimen (holotype), but there is another specimen in Broun’s collection, sex unidentified, with labels: “1163” (written); followed by: “Cohen” (written); followed by: “New Zealand / Broun Coll. / Brit. Mus. / 1922-482” (printed) (BMNH). This second specimen does not have a syntype status, as the species was described based on a single specimen (holotype by monotypy).

##### Additional material examined.

NEW ZEALAND. North Island: ND: 1 spec., Omahutu SF, Kauri Sanctuary, 8.v.1974, G. Kuschel (guano of *Mystacina tuberculata*) (NZAC); 1 spec., ditto, but Kauri Summit, 8.v.1974, G. Kuschel (NZAC); ND: 1 ♂ & 2 ♀♀, Omahuta, S.F., 3.ii.1975, G. Kuschel (*Mystacina* guano and ex bat) (NZAC); 1 spec., Poor Knights Island, Tawhiti Rahi, 9.xii.1980, G. Kuschel (sifted litter) (NZAC). AK: 1 spec., Great Barrier Island, 22.xi.1940, D. Spiller (ex Kingfisher’s nest) (NZAC); 2 specs., Little Barrier Island, 16.iii.1976, D. & M. Smith (from old short-tailed colony-tree) (NZAC). HB: 1 ♀, Motu-o-Kura (Bare Is.), 20–100 m, 10.xii.1991–18.ii.1992, G. Walls (pit traps) (NZAC); 1 ♀, Botanical Garden, Napier (NZAC). South Island. SI: 4 specs., Stewart Island, Codfish I., Valley Track, 26.xi.1981, B.A. Holloway (guano) (NZAC); 1 spec., Stewart Island, Codfish Island, Summit Tk 250 m, 20.xi.1981, B.A. Holloway (moss and lichens) (NZAC); 1 spec., ditto, but Northwest Track, iii.1982, M.J. Daniel (guano) (NZAC). CH: 1 ♂ & 1 ♀, South-East Island, 6.i.1984, C. Miskelly (*Pachyptila vittata* linings of five nests) (NZAC); 1 ♂, South-East Island, 22.i.1975, E. Young (litter from *Puffinus griseus*) (NZAC); 1 spec., South-East Island, Woolshed Bush, 21.i.1998, R.M. Emberson, J.W.M. Marris (ex sieved litter from *Plagianthus*/*Myrsine* forest and broad-billed prion burrow litter) (AMNZ). 5 specs., ditto, but LUNZ; 1 spec., South-East Island, 31.xii.1998, R.M. Emberson (on tree trunks at night) (LUNZ); 1 spec., Kauri Sanctuary, Omauta Forest, N.D., 8.v.1974, G. Kuschel leg. (MNHN, coll. Thérond).

##### Biology.

This species was found in nests and surrounding litter of pelagic birds (petrel burrows, nests of Broad-billed prion (*Pachyptila vittata* (Forster, 1777)); Sooty shearwater (*Ardenna grisea* (Gmelin, 1789)), in the nest of a kingfisher, and in bat guano (*Mystacina tuberculata*). Additional specimens have been collected from moss and lichens, on tree trunks at night and in pitfall traps.

##### Distribution.

New Zealand: North and South Islands, Chatham Islands (Fig. 766).

##### Remarks.

This species is most similar to *T. kuscheli* differing from it by smaller size, obtuse apical pronotal angles, and stronger punctation of clypeus and frons, presence of dorsal stria 1, coarser elytral punctation and wider tibiae. Male terminalia are very similar between the two species (compare Figs 689–697 with 670–678), differing chiefly in the shape of aedeagal apex. Because of the overall similarity between the two species, we provide *T. latipes* only with a diagnostic description outlining the differences between the two species. The figures, as well as male genitalia drawings are kept, for the sake of easier identification of the Australopacific taxa.

**Figures 680–688.**
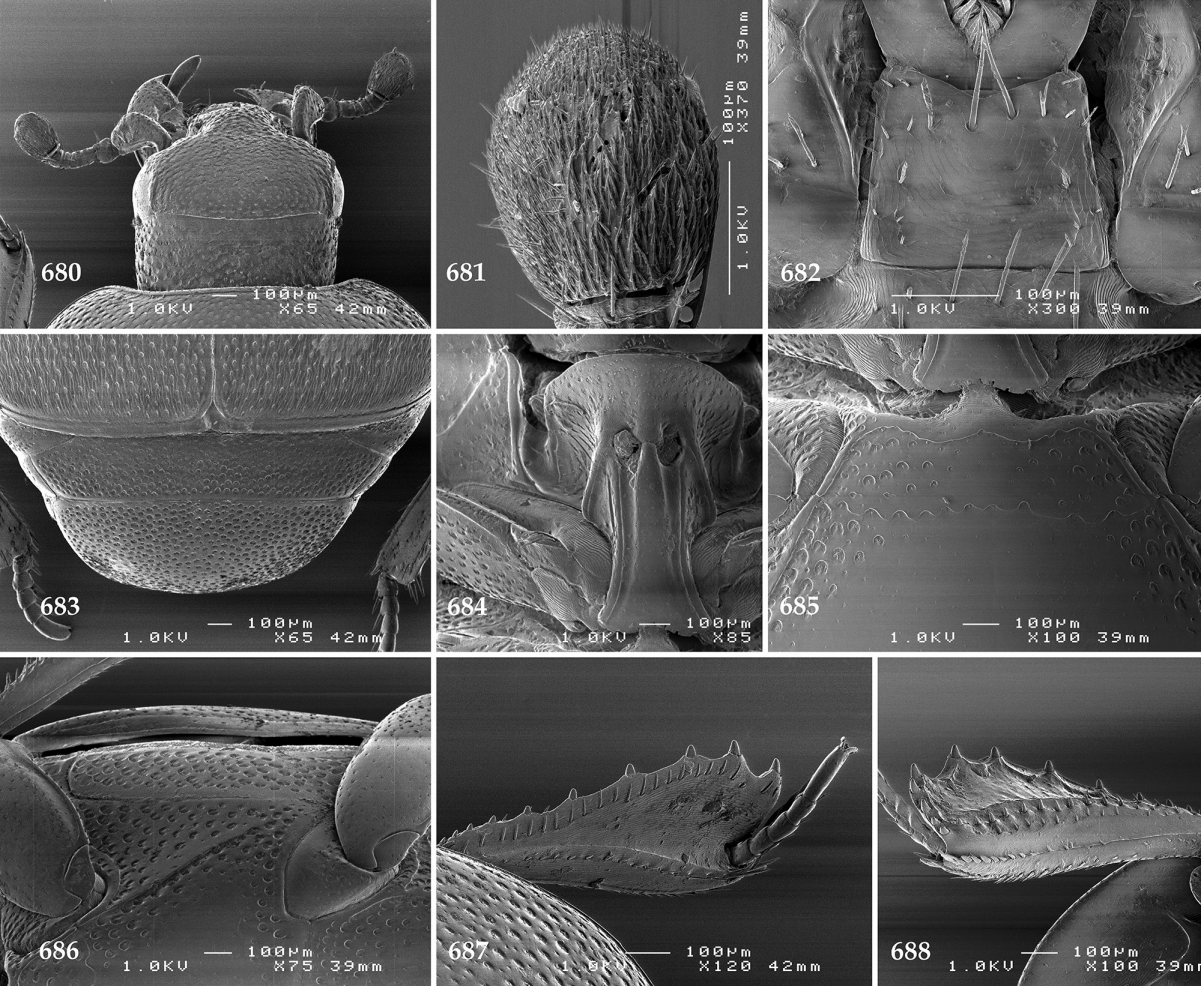
**680** *Tomogenius latipes* (Broun, 1881) head, dorsal view **681** antennal club, ventral view **682** mentum, ventral view **683** propygidium + pygidium **684** prosternum **685** mesoventrite **686** lateral disc of metaventrite + metepisternum **687** protibia, dorsal view **688** ditto, ventral view.

##### Diagnostic description.

**Figures 689–697.**
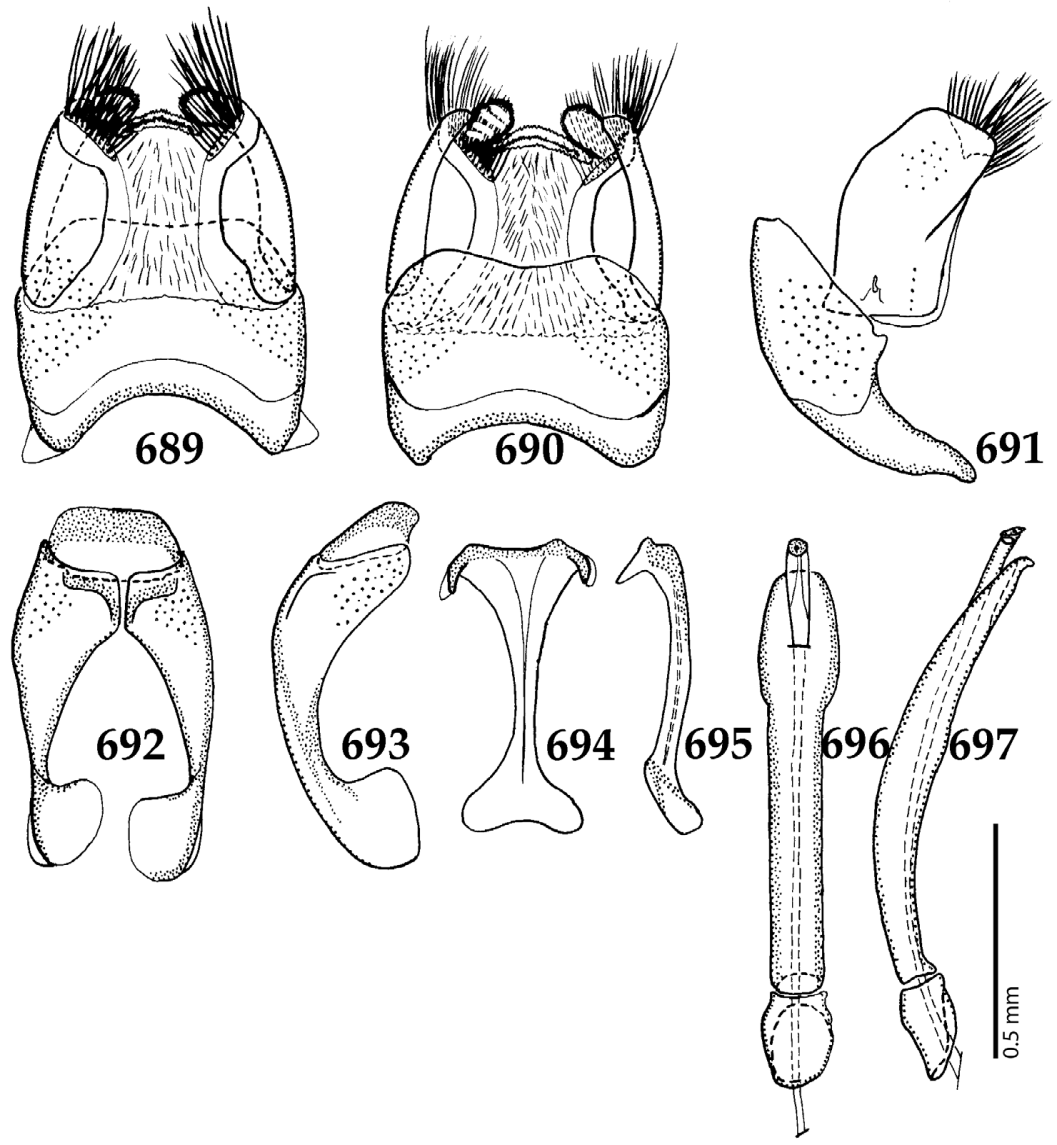
**689** *Tomogenius latipes* (Broun, 1881) male terminalia: 8^th^ sternite + 8^th^ tergite, ventral view **690** ditto, dorsal view **691** ditto, lateral view **692** male terminalia: 9^th^ + 10^th^ tergites, dorsal view **693** ditto, lateral view **694** male terminalia: spiculum gastrale, ventral view **695** ditto, lateral view **696** male terminalia: aedeagus, dorsal view **697** ditto, lateral view.

Body length: PEL: 3.00–3.75 mm; EL: 1.90–2.50 mm; APW: 1.00–1.25 mm; PPW: 2.00–2.25 mm; EW: 2.25–2.85 mm. Body (Figs 679) ovoid, moderately convex, pronotum narrower than elytra; cuticle without metallic luster, dark brown to black; legs, antenna and mouthparts dark brown; antennal club yellow-reddish. Antennae (Figs 680–681) similar to those of *T. kuscheli*, sensory structures of antennal club not examined. Mandibles as with *T. kuscheli*; mentum (Fig. 682) in general also similar between species, but disc of *T. latipes* with fewer setae, median part of mentum almost smooth (compare Figs 663 and 682); cardo of maxilla with few short setae on lateral margin; stipes triangular, with several short setae; terminal maxillary palpomere elongated, its width about one-fourth its length, about three times as long as penultimate palpomere; rest of mouthparts not examined. Clypeus and frons (Fig. 680) similar to those of *T. kuscheli* (Fig. 661), but with very dense confluent elongate punctures; on posterior fifth of frons punctures rounded and sparser; eyes flattened, visible from above. Pronotal sides (Fig. 679) feebly convergent anteriorly, apical angles rather obtuse (prominent in *T. kuscheli*; compare Fig. 679 with 660), marginal pronotal stria complete, thin, slightly carinate; pronotal disc structurally otherwise almost identical to that of *T. kuscheli* but laterally punctures without isodiametric structures, and pre-scutellar depression much less impressed than in *T. kuscheli*, barely noticeable; pronotal hypomeron glabrous; scutellum small. Elytra almost identical to those of *T. kuscheli* (compare Figs 660 and 679), but first dorsal elytral stria in *T. latipes* well developed (not developed in *T. kuscheli*), elytral striae in punctures and sutural elytral stria slightly longer apically than in *T. kuscheli*; elytral punctation even denser and coarser, confluent. Punctures on apical elytral third distinctly aciculate in *T. latipes* whereas they are not aciculate in *T. kuscheli*. Propygidium and pygidium almost identical to that of *T. kuscheli*, but punctation in *T. latipes* even denser than that of *T. kuscheli* (compare Figs 664 and 683). Prosternum (Fig. 684) except for some minute details identical to that of *T. kuscheli* (compare Figs 665 and 684). Mesoventrite (Fig. 685) well impressed, somewhat carinate, anteriorly medially projected; disc flattened, laterally with deep large punctures separated several times their diameter, becoming finer and even sparser medially; meso-metaventral suture distinct, meso-metaventral sutural stria well impressed, undulate, slightly distanced from meso-metaventral suture. In general, mesoventrite almost identical to that of *T. kuscheli*, but punctures even larger and sparser and meso-metaventral sutural stria thinner. Intercoxal disc of metaventrite with regards to coarser punctation in *T. latipes* and some minute details absolutely identical to that of *T. kuscheli* (compare Figs 666 with 686). Intercoxal disc of first abdominal ventrite similar to that of *T. kuscheli*, but punctures laterally sparser and larger and medially almost smooth. Protibia (Fig. 687) flattened and dilated, outer margin with five very low teeth followed by 4 tiny denticles diminishing in size proximally; first two teeth approximate, separated from the second pair of teeth (which can also be approximate) by rather wide and deep gap. Fifth tooth separated likewise by wide, but by no means deep gap. Setae of outer row short, regular and sparse; setae of median row similar, but shorter and finer than those of outer row, growing in size distally; protarsal groove shallow; anterior protibial stria complete, costate; two thin, rather long tarsal denticles present apically; protibial spur short, straight, growing out from apical protibial margin; apical margin of protibia posteriorly with two or three apical denticles; outer part of posterior surface of protibia (Fig. 688) smooth, well divided from median part of posterior surface by ridge like stria; median part of posterior surface with two sparse rows of minuscule setae; posterior protibial stria complete with tightly-spaced short and stout denticles near apical margin; inner margin with double row of short setae (similar to that of *T. australis*, but more dilated and teeth on outer margin smaller; setae on outer row shorter). Mesotibia and metatibia basically similar to those of *T. kuscheli* or *T. australis*, but both more dilated and denticles on outer margin shifted onto the anterior surface of tibiae and therefore both rows of denticles growing out from there (furthermore claws of apical meso- and metatarsomeres shorter than those of afore-mentioned species). Male genitalia. Basically very similar to those of *T. kuscheli* (compare Figs 689–697 and 670–678), differing in more convergent eighth sternite and, especially, the shape of apical third of aedeagus, which is less dilated in *T. latipes* than in *T. kuscheli*.

#### Tomogenius motocola

Taxon classificationAnimaliaColeopteraHisteridae

Mazur, 1990

Figs 698 , 699–707 , 708–715 , 767

Tomogenius motocola Mazur, 1990: 774, figs. 1–4.

##### Type locality.

Australia: South Australia: 18 miles ESE of Penong.

**Figure 698.**
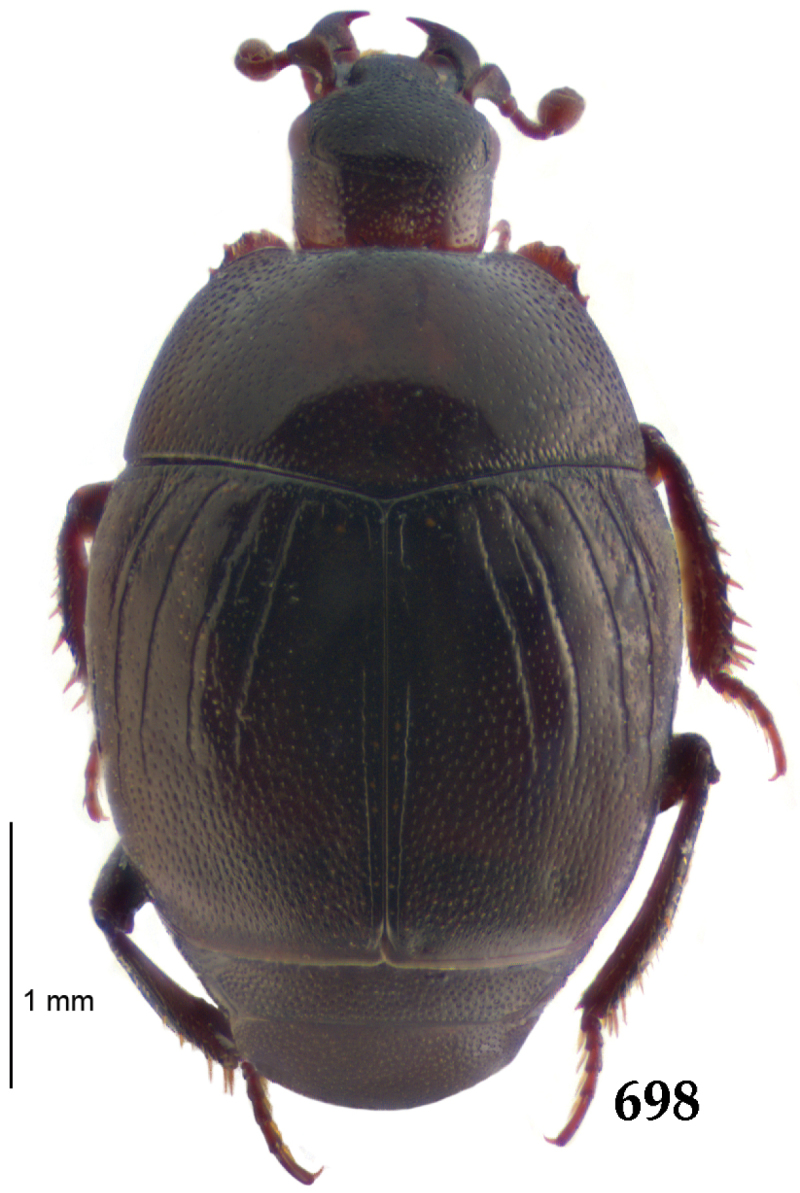
*Tomogenius motocola* Mazur, 1990 habitus, dorsal view.

##### Type material examined.

*Tomogenius motocola* Mazur, 1990: paratype, ♀, side-mounted on a triangular card: “32 mi, NW by N / of Eucla Motel / W.A. 18.Oct.1968 / Britton, Upton / Balderson” (printed); followed by: “ANIC Database No. / 25 054256” (printed); followed: “PARATYPE” (blue, printed label); followed by: “*Tomogenius* / *motocola* / det. S. Mazur” (printed-written); followed by: “Paratypus” (beige printed label); followed label: “Tomogenius / motocola” (written); followed by: “10-141” (yellow, pencil-written label; added by the senior author) (ANIC).

##### Additional material examined.

AUSTRALIA. Western Australia: 2 ♂♂, Nullarbor Pl., Dingo Cave, 28.x.1968, J. Lowry (ANIC; 1 ♂ in coll. TLAN); 1 ♀ & 1 ♂, Madura Cave, 27.x.1968, J. Lowry (ANIC); 2 specs., King George’s Sound, without further details (AMS). South Australia: 1 ♀, Koonalda Cave, 3.iv.1970, J. Lowry (ANIC); 12 specs., Fisher E.W. Line, coll. Toughlow & Wright, further details missing (AMS; 3 specs. in coll. TLAN); 1 spec., Fisher, Troughlow & Wright coll.; K.K.Spence collection, further details absent (AMS); 1 spec., Murray River, A.H. Elston coll., without further details (AMS).

##### Biology.

This species is found in caves.

##### Distribution.

South and Western Australia (Fig. 767).

##### Re-description.

Body length: PEL: 3.25–3.40 mm; APW: 1.50–1.60 mm; PPW: 2.50–2.60 mm; EL: 2.20–2.25 mm; EW: 2.75–2.85 mm.

Body (Fig. 698) ovoid, moderately convex from above, underside slightly flattened, pronotum narrower than elytra, cuticle chestnut brown, without metallic luster; legs, mouthparts and antennae appendages similarly colored.

**Figures 699–707.**
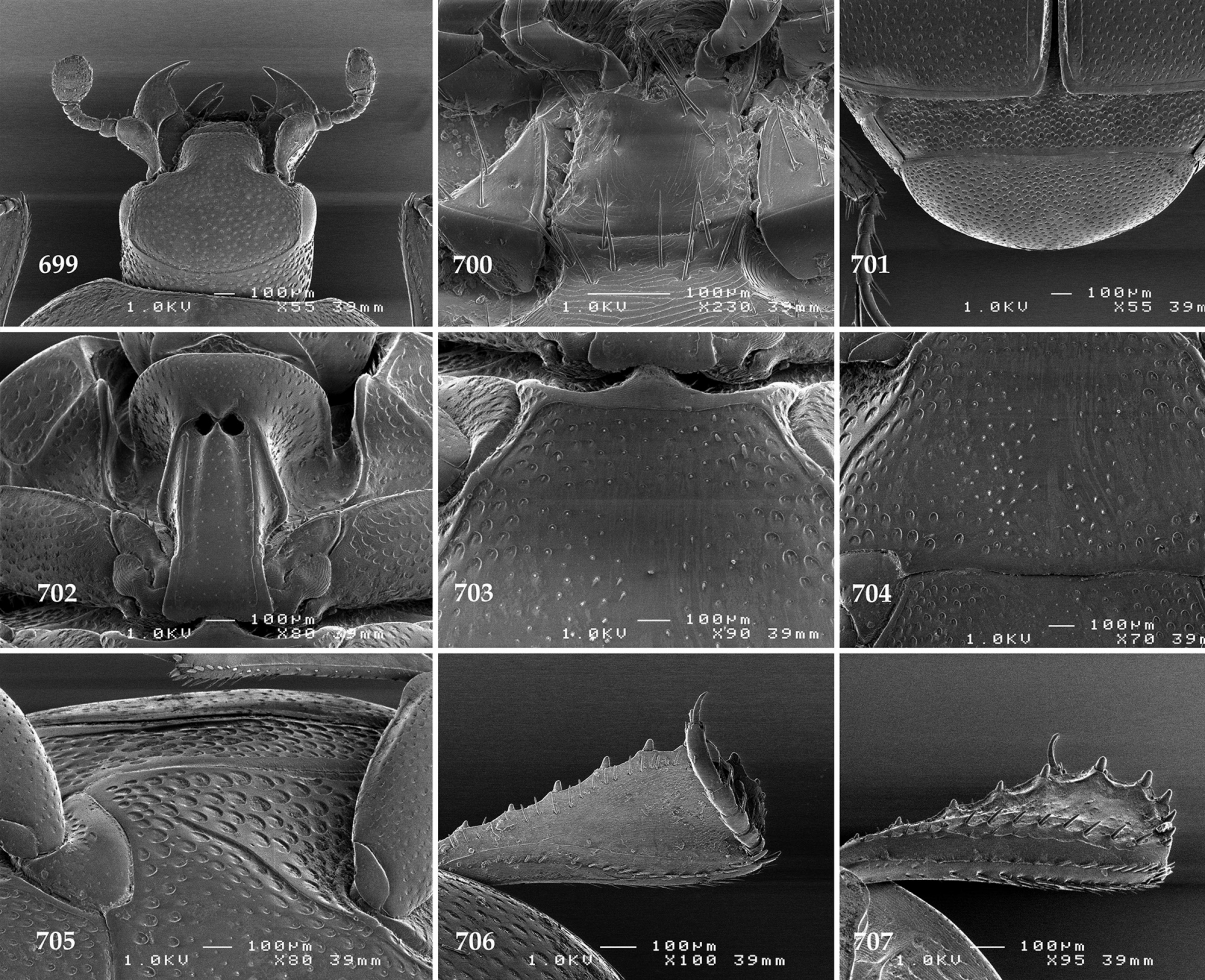
**699** *Tomogenius motocola* Mazur, 1990 head, dorsal view **700** mentum, ventral view **701** propygidium + pygidium **702** prosternum **703** mesoventrite **704** metaventrite **705** lateral disc of metaventrite + metepisternum **706** protibia, dorsal view **707** ditto, ventral view.

Antennal scape (Fig. 699) not particularly thickened, with two short setae; club rather large, oval, dorsally with two horizontal slit-like pits, ventrally with two vertical slit-like pits, entirely covered in dense short sensilla, intermingled with sparse longer erect sensilla; sensory structures of antennal club apart from sensory slit-like pits present dorsally and ventrally, not examined.

Mandibles (Fig. 699) impunctate, with rounded outer margin, acutely pointed, sub-apical tooth on inner margin of left mandible very small, almost inconspicuous; labrum even, sparsely punctate, approximately twice as wide as long, labral pits each with two moderately long labral setae; terminal labial palpomere elongated, its width about one-fourth its length; mentum (Fig. 700) sub-trapezoid, anterior angles slightly produced, anterior margin with a shallow median notch, surface around it two long setae from each side, lateral margins with a single one row of much shorter sparse ramose setae, disc with few scattered setae; rest of the mouthparts not examined.

Clypeus (Fig. 699) even, rounded laterally, on anterior half with sparse fine punctures, separated by several times their diameter, punctures becoming larger and coarser on posterior half; frontal and supraorbital striae absent, occipital stria present, fine; frontal disc (Fig. 699) with fine round to ellipsoid punctures separated by several times their diameter; eyes convex, well visible from above.

Pronotal sides (Fig. 698) feebly convergent anteriorly, apical angles obtuse, marginal pronotal stria complete, thin, slightly carinate, somewhat weakened behind head; disc entirely punctate, medially punctures separated by several times their diameter, fine, becoming coarser and denser laterally, especially near apical angles where they are separated by less than their own diameter, large and ovoid; pronotal hypomeron glabrous; scutellum very small.

Elytral epipleura with scattered punctures of various sizes; marginal epipleural stria double, both striae weakly impressed but complete; marginal elytral stria well impressed, continuous along elytral apex as very weakened apical elytral stria; humeral elytral stria inconspicuous (absent?); inner subhumeral stria present as a short median fragment; elytral disc with four dorsal elytral striae 1–4, all striae in punctures, first originating at elytral base, other three striae slightly distanced from elytral base and their basal ends curved; first and second reaching about two-thirds of elytral length apically, third and fourth striae slightly shorter than first and second, surpassing elytral length apically; between fourth dorsal elytral and sutural striae a characteristic hooked appendix present, basally connected to basal fragment of sutural elytral stria; sutural elytral stria apart from this basal fragment evanescent on basal third, on apical two-thirds (roughly) present, fine, becoming weaker apically. Entire elytral disc punctate, on basal half (roughly) punctures very fine and sparse, separated several times their diameter, on apical half (roughly) punctures larger and denser, separated approximately by their diameter, becoming denser apically; punctures near extreme elytral apex with minuscule striolae among them.

Propygidium (Fig. 701) transverse, about four times as broad as long, completely exposed, with very dense punctures separated by less than their diameter; pygidium (Fig. 701) with sparser round punctures, separated by about their diameter, becoming sparser and finer towards apex.

Anterior margin of median portion of prosternum (Fig. 702) rounded; marginal prosternal stria present only laterally; prosternal process slightly convex on apical third between carinal prosternal striae, broad, dorso-laterally with sparse fine punctures; carinal prosternal striae (Fig. 702) straight, parallel, terminating near large and deep apical foveae separated by apex of prosternal process; lateral prosternal striae carinate, slightly convergent anteriorly, attaining apices of carinal prosternal striae.

Discal marginal mesoventral stria (Fig. 703) well impressed, somewhat carinate, straight medially; disc flattened, with round punctures separated by about their diameter, punctures becoming finer medially, in female paratype examined punctures of mesoventrite with very short setae; meso-metaventral suture distinct, straight meso-metaventral sutural stria absent.

Intercoxal disc of metaventrite (Fig. 704) in examined female paratype medially with shallow depression; disc of metaventrite punctate, medially punctures finer and sparser, in male with minuscule setae becoming larger and coarser along lateral and basal margins. Lateral metaventral stria well impressed, carinate, slightly bisinuate, almost straight, not reaching metacoxa; lateral disc of metaventrite (Fig. 705) flattened, with round shallow large punctures; metepisternum + fused metepimeron (Fig. 705) evenly with much coarser and denser punctation; lateral metepisternal stria present, deeply impressed and almost complete.

Intercoxal disc of first abdominal ventrite flattened, completely striate laterally; surface of disc with scattered oblong punctation, punctures becoming sparser and finer medially.

Protibia (Fig. 706) flattened and somewhat dilated, outer margin with three low apical teeth topped by short denticle, followed by five widely spaced short denticles; setae of outer row short, moderately dense; setae of median row similarly dense and regular, slightly shorter than those of outer row; protarsal groove shallow; anterior protibial stria complete, costate; two thin, short tarsal denticles present apically; protibial spur short, straight, growing out from apical protibial margin; apical margin of protibia posteriorly with three tiny apical denticles; outer part of posterior surface of protibia (Fig. 707) finely imbricate, with a row of short setae; median part of posterior surface with an additional row of minuscule setae; posterior protibial stria complete, with scattered minuscule setae turning into a row of tightly-spaced short setae near apical margin; inner margin with double row of dense short setae.

Mesotibia slender, outer margin with a single row of dense thin denticles growing in size apically; setae of outer row sparse, regular, short, growing somewhat longer apically; setae of median row irregular, much shorter than those of outer row; posterior mesotibial stria complete; anterior surface of mesotibia with dense row of well sclerotized short denticles, with another similar row of much shorter and finer setae situated below it; anterior mesotibial stria complete, terminating in several tiny inner anterior denticles; mesotibial spur stout, short; apical margin with two tiny denticles; mesotarsus shorter than mesotibia; claws of apical tarsomere about half its length; metatibia basically similar to mesotibia, but denticles of outer margin much sparser than those of mesotibia; claws of apical tarsomere in both cases slightly bent, shorter than half its length.

**Figures 708–715.**
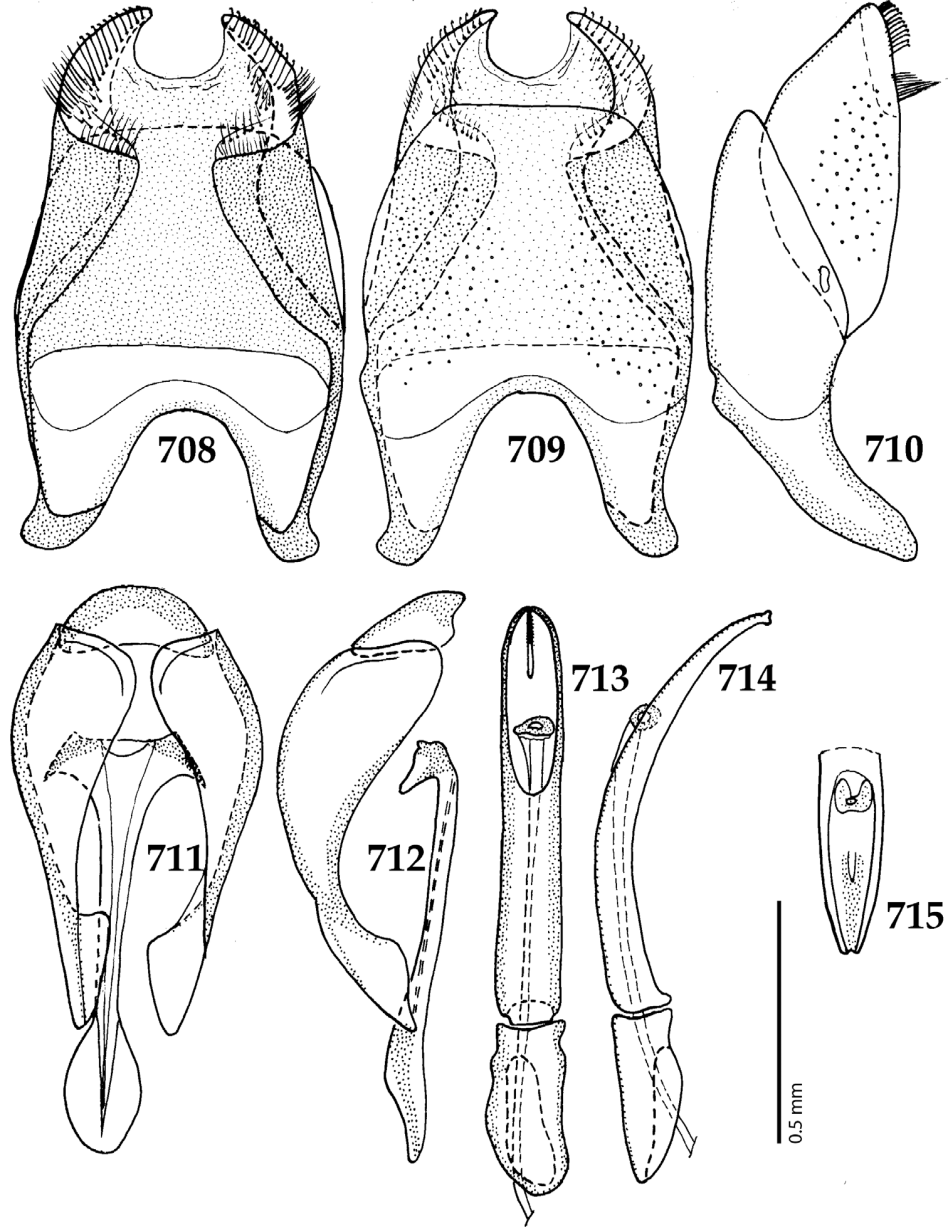
**708** *Tomogenius motocola* Mazur, 1990 male terminalia: 8^th^ sternite + 8^th^ tergite, ventral view **709** ditto, dorsal view **710** ditto, lateral view **711** male terminalia: 9^th^ + 10^th^ tergites, dorsal view; spiculum gastrale, ventral view **712** male terminalia: 9^th^ + 10^th^ tergites; spiculum gastrale, lateral view **713** male terminalia: aedeagus, dorsal view **714** ditto, lateral view **715** male terminalia: apex of aedeagus, frontal view.

Male genitalia. Eighth sternite (Figs 708–709) fused on its entire length; vela laterally with two brushes of regular short setae creating a ‘suction-cup’ like appearance best observable from lateral view; eighth tergite apically straight, laterally with numerous pseudo-pores; eighth tergite and eighth sternite not fused laterally (Fig. 710). Ninth tergite (Figs 711–712) longitudinally divided medially; tenth tergite conspicuously small, basally slightly inwardly arcuate, apically rounded; spiculum gastrale (Fig. 711) gradually dilated near its apical end, basal end slightly dilated, spoon-like, outwardly arcuate. Aedeagus (Figs 713–714) slender, parallel-sided, parameres not fused on their apical third, apex of aedeagus acute (Fig. 715); basal piece of aedeagus short, ratio of its length : length of parameres 1 : 3; aedeagus curved from lateral view (Fig. 714).

#### Tomogenius papuaensis

Taxon classificationAnimaliaColeopteraHisteridae

Gomy, 2007

Figs 716 , 717–722 , 723–731 , 755

Tomogenius papuaensis Gomy, 2007: 42, figs 2, 4, 6 and photographs.

##### Type locality.

Papua New Guinea: Tapini.

**Figure 716.**
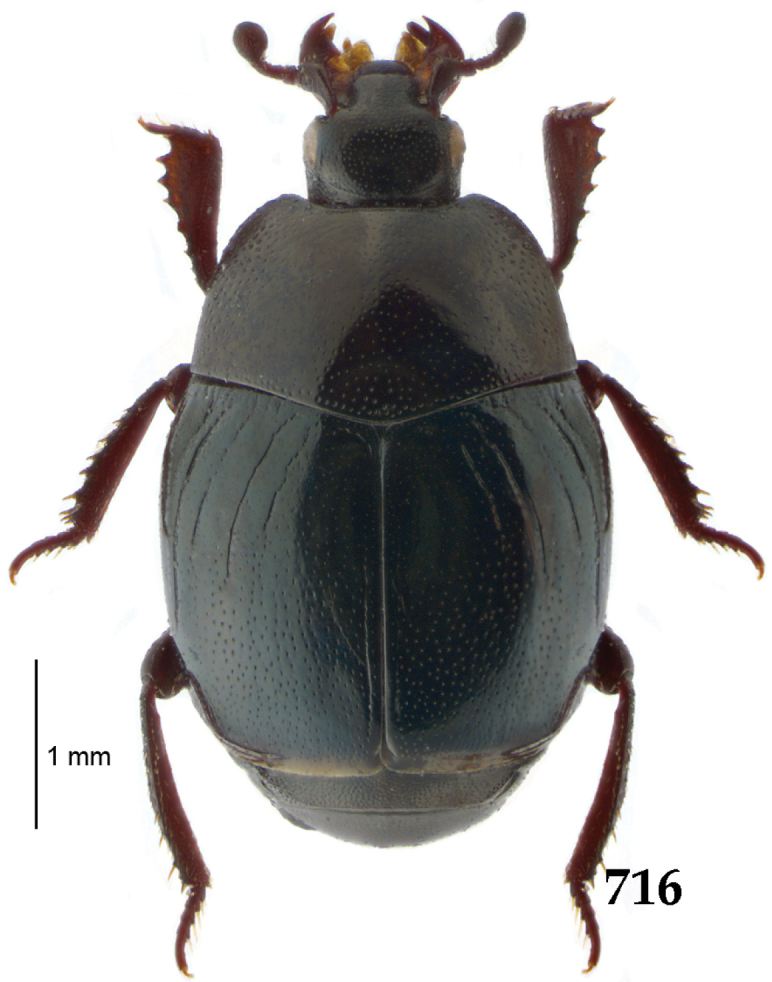
*Tomogenius papuaensis* Gomy, 2007 habitus, dorsal view.

##### Type material examined.

*Tomogenius papuaensis* Gomy, 2007: paratype, ♂, side-mounted on triangular mounting card, with genitalia glued to another triangular mounting card situated below the specimen, with the following labels: “Nlle. GUINEE / Tapini VII. 1968 / (J. POULLARD réc.)” (printed); followed by: “♂” (printed); followed by: “*Tomogenius* / *papuaensis* Gomy / Y. Gomy - Det. 2009” (written-printed); followed by: “Y. Gomy des. / PARATYPE” (red label, printed); followed by: “Collection / Y. GOMY” (printed); followed by: “09-095” (yellow label, pencil-written, added by the senior author) (TLAN).

##### Biology.

Found in a cave in bat guano (Gomy 2007).

##### Distribution.

Papua New Guinea: Tapini (Fig. 755).

##### Re-description.

**Figures 717–722.**
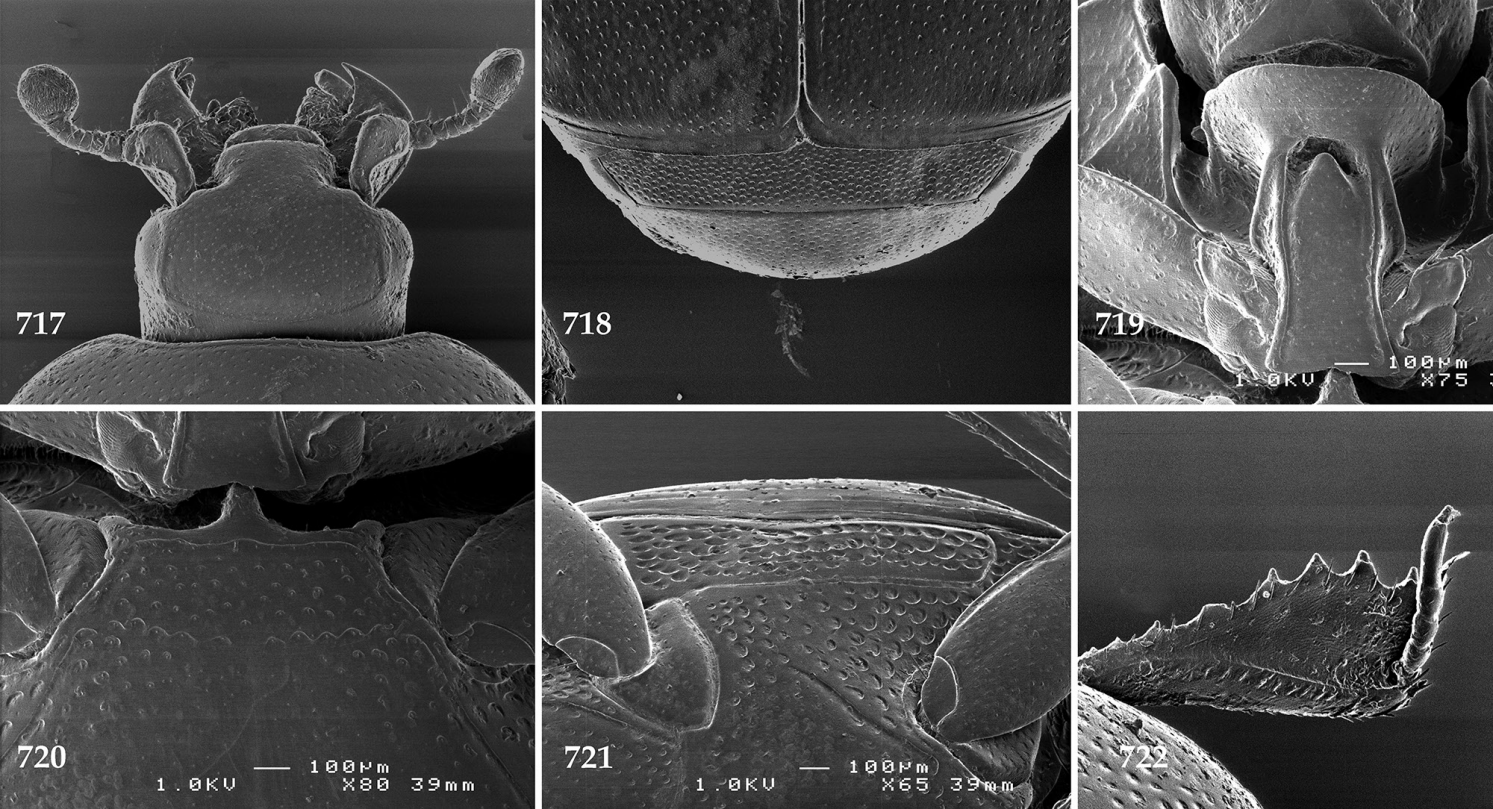
**722** *Tomogenius papuaensis* Gomy, 2007 head, dorsal view **718** propygidium + pygidium **719** prosternum **720** mesoventrite **721** lateral disc of metaventrite + metepisternum **722** protibia, dorsal view.

Body length: PEL: 3.35 mm; APW: 1.20 mm; PPW: 2.25 mm; EL: 2.25 mm; EW: 2.75 mm. Body (Fig. 716) ovoid, moderately convex from above, underside slightly flattened, cuticle almost black without metallic luster; legs and body appendages castaneous brown. Antennal scape (Fig. 717) not particularly thickened, with few short setae; club rather large, oval, entirely covered in dense short sensilla, intermingled with sparse longer erect sensilla; sensory structures of antennal club not examined.

Mandibles with rounded outer margin, acutely pointed, labrum slightly convex, sparsely punctate; mentum sub-trapezoid, anterior angles slightly produced, anterior margin with a shallow median notch, surface around it with several long setae, disc without setae; other mouthparts not examined.

Clypeus (Fig. 717) large, rectangular, flattened, with sparse fine punctures separated by several times their diameter; frontal stria absent; supraorbital stria vaguely present; frontal disc (Fig. 717) with sparse fine round punctures larger and coarser than those of clypeus; eyes convex, well visible from above.

Pronotal sides (Fig. 716) on basal two-thirds feebly convergent anteriorly, on apical third strongly convergent, apical angles obtuse, marginal pronotal stria complete, thin, slightly carinate; disc medially covered with sparse round fine punctation, punctures separated by several times their diameter, laterally punctation becomes coarser and denser; pronotal hypomeron glabrous; scutellum very small.

Elytral epipleura with very fine scattered punctures; marginal epipleural stria double, both striae weakly impressed but complete; marginal elytral stria well impressed, continuous along elytral apex as apical elytral stria, stopping in middle of elytral apical margin; humeral elytral stria well impressed on basal fourth; inner subhumeral stria absent, short; elytral disc with four dorsal elytral striae 1–4, first the longest, reaching about two-thirds of elytral length apically, second and third striae ending short of elytral half, fourth dorsal elytral stria the shortest, reaching about elytral third apically; characteristic hooked appendix present between fourth dorsal elytral and sutural striae in other species of the genus absent; sutural elytral stria weakened, intermittent, erased on basal third and apical tenth. Entire elytral disc punctate, on basal half (roughly) punctures very fine and sparse, on apical half (roughly) punctures larger and denser, but still rather sparse, separated approximately two-three times their diameter.

Propygidium (Fig. 718) transverse, about four times as broad as long, completely exposed, with dense and coarse punctures separated by less than their diameter; pygidium (Fig. 718) with sparser punctation, becoming even sparser and finer towards apex.

Anterior margin of median portion of prosternum (Fig. 719) rounded; marginal prosternal stria present only laterally; prosternal process flattened, broad, sparsely punctate; carinal prosternal striae (Fig. 719) slightly bisinuate, terminating near large and deep united apical foveae; lateral prosternal striae carinate, slightly convergent anteriorly, attaining apices of carinal prosternal striae. Lateral costa of antennal groove not reaching prosternal process.

Anterior margin of mesoventrite (Fig. 720) almost straight; discal marginal mesoventral stria well impressed, slightly carinate; disc flattened, with coarse punctures separated by about their diameter; meso-metaventral suture vague, meso-metaventral sutural stria undulate, intermittent.

Intercoxal disc of metaventrite medially with slight longitudinal depression creating near basal margin two obtuse tubercles; disc of metaventrite rather coarsely punctate near anterior angles, medially punctures finer and sparser, becoming larger and coarser again along lateral margin. Lateral metaventral stria well impressed, carinate, shortened; lateral disc of metaventrite (Fig. 721) flattened, with round shallow large punctures fringed with microscopic setae; metepisternum + fused metepimeron (Fig. 721) evenly covered with much coarser and denser punctation, punctures without setae; lateral metepisternal stria present, deeply impressed and almost complete.

Intercoxal disc of first abdominal ventrite almost completely striate laterally; surface of disc with scattered oblong punctation, punctures becoming sparser and finer medially.

Protibia (Fig. 722) flattened and somewhat dilated, outer margin with six low teeth, topped by short triangular denticles followed by two-three minuscule denticles; setae of outer row short, sparse; setae of median row denser, slightly shorter than those of outer row; protarsal groove shallow; anterior protibial stria complete, costate; two thin, rather long tarsal denticles present apically; protibial spur short, straight, growing out from apical protibial margin; apical margin of protibia posteriorly with four tiny apical denticles; outer part of posterior surface of protibia finely imbricate, with a row of short setae; median part of posterior surface with additional row of minuscule setae; posterior protibial stria complete, with scattered minuscule setae turning into several tightly-spaced short and stout denticles near apical margin; inner margin with double row of short lamellate setae.

Mesotibia slender, outer margin with a single row of dense thin denticles growing in size apically; setae of outer row and those of median row not examined; posterior mesotibial stria not examined; anterior surface of mesotibia with dense row of well sclerotized short setae, with another similar row of much shorter situated below it; anterior mesotibial stria complete, terminating in numerous tiny inner anterior denticles; mesotibial spur broken off; apical margin with two tiny denticles; mesotarsus shorter than mesotibia; claws of apical tarsomere about half its length; metatibia basically similar to mesotibia, but denticles of outer margin much sparser than those of mesotibia; claws of apical tarsomere somewhat shorter, about one-third its length.

**Figures 723–731.**
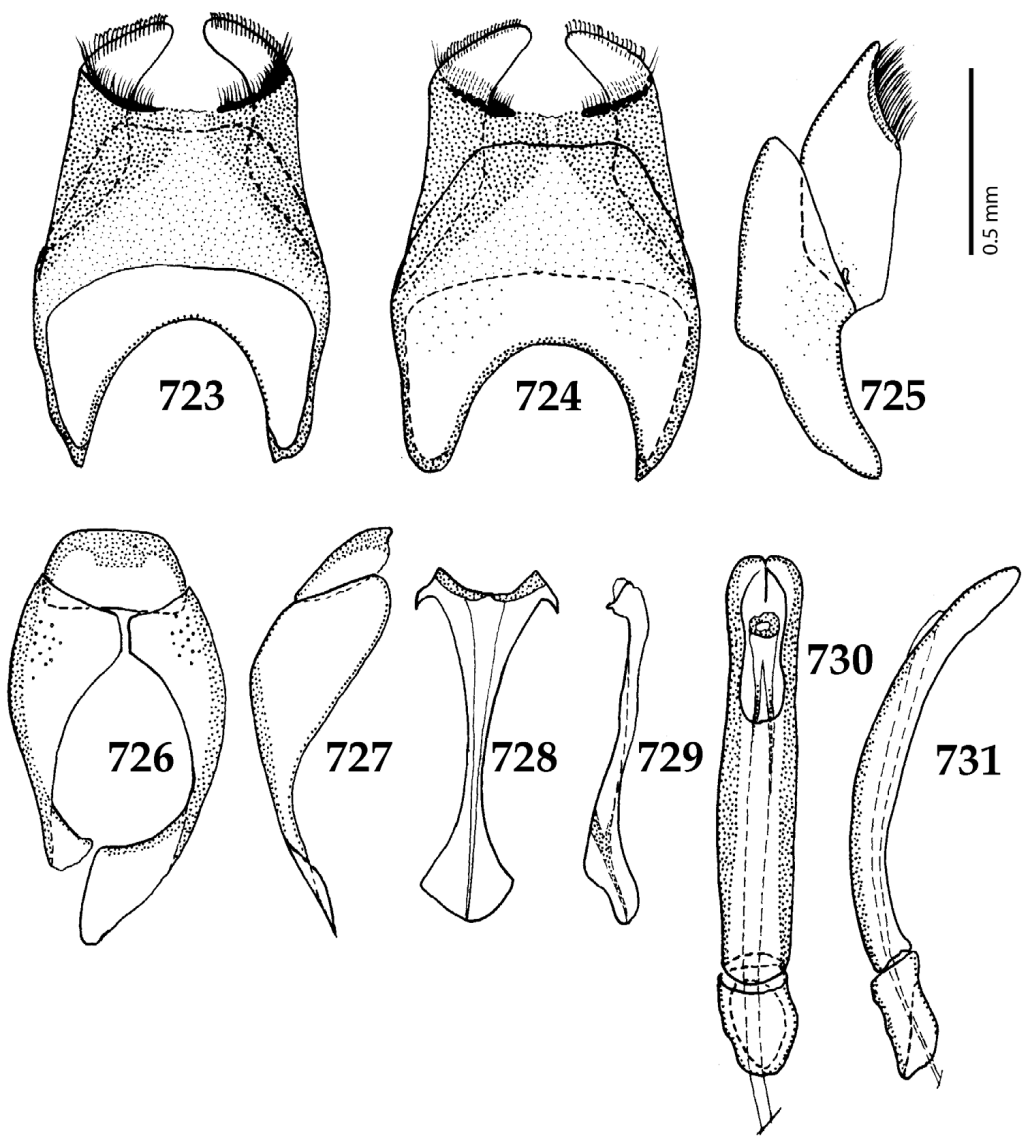
**723** *Tomogenius papuaensis* Gomy, 2007 male terminalia: 8^th^ sternite + 8^th^ tergite, ventral view **724** ditto, dorsal view **725** ditto, lateral view **726** male terminalia: 9^th^ + 10^th^ tergites, dorsal view **727** ditto, lateral view **728** male terminalia: spiculum gastrale, ventral view **729** ditto, lateral view **730** male terminalia: aedeagus, dorsal view **731** ditto, lateral view.

Male genitalia. Eighth sternite (Figs 723–724) fused medially; apically with a closely-set cluster of long, brush-like setae; eighth tergite and eighth sternite not fused laterally (Fig. 725). Ninth tergite (Figs 726–727) longitudinally divided medially; spiculum gastrale (Figs 728–729) gradually dilated in most of apical half, basal end dilated, spoon-like. Aedeagus (Figs 730–731) slender, subparallel, slightly bisinuate before apex; basal piece of aedeagus short, ratio of its length : length of parameres 1 : 4; parameres fused along their basal two-thirds (roughly); aedeagus slightly curved from lateral view (Fig. 731).

#### Tomogenius ripicola

Taxon classificationAnimaliaColeopteraHisteridae

(Marseul, 1870)

Figs 732 , 733–741 , 742–750 , 767

Saprinus ripicola Marseul, 1870: 118.

##### Type locality.

Australia: Murray River.

**Figure 732.**
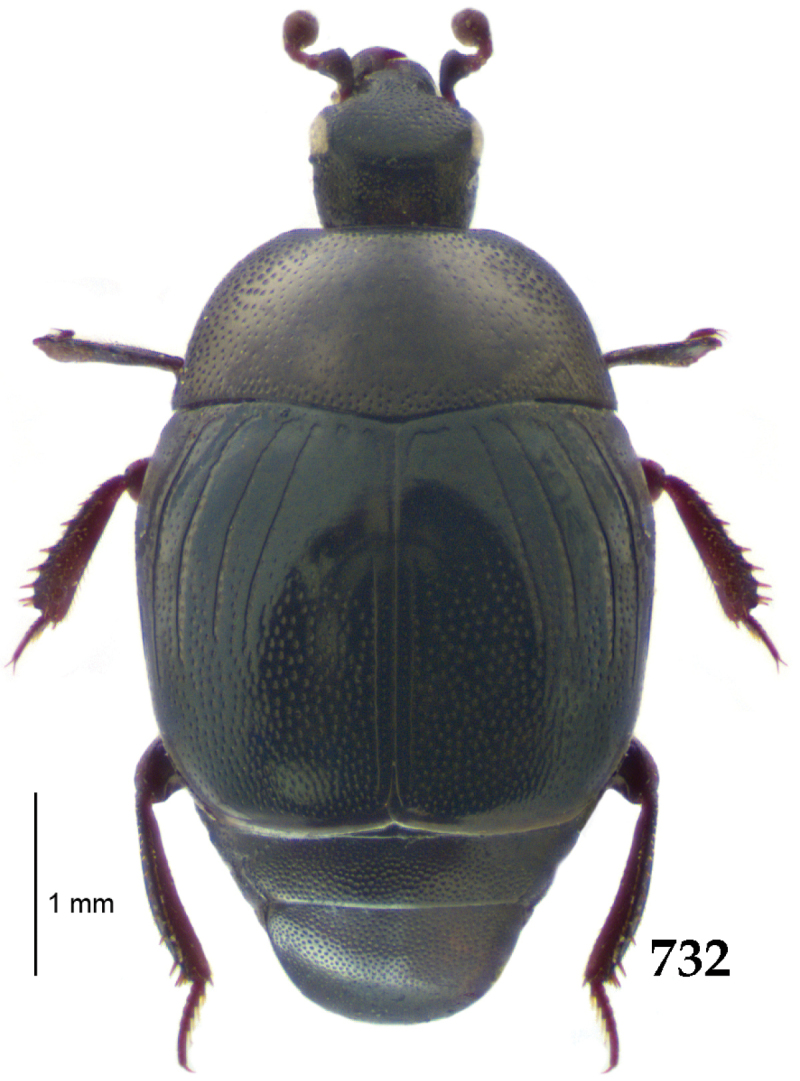
*Tomogenius ripicola* (Marseul, 1870) habitus, dorsal view.

##### Type material examined.

*Saprinus ripicola* Marseul, 1870: Lectotype (designated by Y. Gomy in 2004), ♂, side-mounted with extracted and dismembered genitalia glued onto the same mounting card as the specimen, both antennal clubs missing, both protarsi and right mesotarsus missing, both metatibiae broken off, glued to the same mounting card as the specimen, with the following labels: “Gnathoncus / ripicola M / Murray Riv. ♂ / illegible” (round pink label, written); followed by: “Murray / Riv.” (pencil-written); followed by: “MUSEUM PARIS / COLL. / DE MARSEUL 1890” (pink label, printed); followed by: “TOMOGENIUS / G. Dahlgren det / RIPICOLA MARS.” (printed-written); followed by: “♂” (written); followed by: “Tomogenius / ripicola / Mars. / Y. GOMY DET 2004” (written-printed); followed by: “Y. Gomy des. / LECTOTYPE” (red label, printed) (MNHN). Paralectotype, ♂, designated by Y. Gomy in 2004, side-mounted with genitalia extracted, dismembered and glued to the same mounting card as the specimen, right antennal funicle missing, right protibia, left protarus, both mesotarsi and right metatarsus missing, with the following labels: “♂” (written); followed by: “Gnathoncus ? / illegible / ♂ / Murray riv. / Cast. 69” (round, pink label, written); followed by: “TOMOGENIUS / RIPICOLA / TYPE” (pencil-written); followed by: “MUSEUM PARIS / COLL. / DE MARSEUL 1890” (pink label, printed); followed by: “TOMOGENIUS / G. Dahlgren det / RIPICOLA MARS.” (printed-written); followed by: “TYPE” (red-printed label); followed by: “Y. Gomy des. / PARALECTOTYPE” (red label, printed) (MNHN).

##### Additional material examined.

AUSTRALIA. Queensland: 4 ♀♀ & 6 ♂♂ Flogged Horse Cave, 20 km N of Rockhampton, 14.xi.1986, R.B. Halliday (ex bat guano) (ANIC); 8 ♀♀ & 10 ♂♂ & 1 spec., Mt. Etna, Johannsens Mine, 15.v.1986, E. Holm (ex ghost-bat guano) (ANIC); 1 ♂, Isla Gorge National Park, 5 km NE, 25°10'S, 150°01'E, 270 m, 3.–5.iv.1998, G. Monteith (O/F dung trap) (QM); 1 spec., Wyreema, no date, O.W. Tiegs (QM); 1 ♂, Boggom, 12/2 (Nathan G) via Taroom, 25°27'S, 150°08'E, 13.xi.1996–i.1997, Cook & Monteith (FIT trap, baited) (QM); 1 ♂, One Tree Hill, 5.5 km SE, 25°20'S, 151°55'E, 120 m, 26.ix.–14.xii.1999, D. & I. Cook (vine scrub intercept) (QM). New South Wales: 1 spec., New South Wales, without further data (BMNH); 3 specs., Forest Keep, Tammworth, Lea leg. (BMNH); 1 ♀, Salt Hole Creek, 38 km NE of Broken Hill, 27.ix.1975, Z. Liepa (ANIC); 1 ♀, Wahroonga, no date, H.J. Carter (ANIC); 1 ♂, 20 km W Kempsey, 14.i.1987, Yessabah Cave, E. Holm (bat guano) (ANIC); 1 ♂, Lithgow, 8 km SW, 33°31'S, 150°05'E, 17.xi.1991, Tom Gush (on dead kangaroo) (ANIC); 1 ♂, Lake George, 10.–12.i.1969, W.J.M. Vestjens (ANIC); 1 ♂, Acacia Plateau, H. Davidson (ANIC); 1 spec., Tamwroth, Lea (SAMA). Western Australia: 1 ♀ & 1 ♂, Weelawadji Cave, 29.45S 115.10E, 14–24 km N of Stockyard Gully, 17 km W of Eneabba, 8.x.1972, J. Lowry (ANIC); 3 specs., Mt. Barker; 1 ♂, Liefden-Lyndhurst, 2.iv.1960, P. Aitken (SAMA); 1 spec., Pelsart Island, Houtman’s Abrs., Lea (SAMA); 1 spec., New Norcia, x.1953, Demarz leg. (MNHN, coll. Thérond). South Australia: 1 ♂, Port Lincoln, Blackburn, 1884 (BMNH); 1 spec., S. Australia, without further data (BMNH); 1 spec., Adelaide (? - illegible), without further data (BMNH); 1 ♂, Flinders Island, 33.43S 134.31E, 27.vii.–2.viii.1987, J.E. Feehan (ANIC); 1 spec., Ardrossan, J.G.O. Tepper (SAMA); 1 spec., Ferries-McDonald National Park, 12.v.1977, E.G. Matthews (SAMA); 2 specs., Mt. Lofty, J.G.O. Tepper (SAMA); 4 specs., ditto, but S.H. Curnow (SAMA); 2 specs., Lucindale, Feuerheerdt (SAMA). Australian Capital Territory: 1 ♂, Gungahlin, 20.ix.1964, W.J.M.Vestjens; 1 ♀, ditto, but 13.ii.1966 (both exs. ANIC). Victoria: 1 ♀ & 1 spec., Victoria, M.F.L., no further data (BMNH); 2 specs., Birchip, J.C. Goudie (SAMA); 1 spec., Tawonga, 2 km W, 30°40'S, 147°08'E, 13.x.1990, Tom Gush (on dead sheep) (ANIC). Unknown localities. 1 spec., Galeon? (illegible), Pascoe Coll., no further data (BMNH); 2 ♂♂ & 2 specs., M.F.L vii.[19]35, Evansford. V., no further data (BMNH).

##### Biology.

Found on bat guano in caves as well as on carrion.

##### Distribution.

Australia: New South Wales, Queensland, Victoria, Australian Capital Territory, Western Australia and South Australia (Fig. 767).

##### Remarks.

It is unclear why Gomy designated a lectotype from a specimen that did not bear the “Type” label; perhaps because the specific epithet on the “Type”-labelled specimen did not read “*ripicola*” nor anything resembling it.

##### Re-description.

Body length PEL: 2.60–2.90 mm; APW: 1.00–1.15 mm; PPW: 1.85–2.10 mm; EW: 2.25–2.40 mm; EL: 1.65–1.90 mm. Body (Fig. 732) ovoid, moderately convex from above, underside slightly flattened, pronotum narrower than elytra, elytra widest at humeri; cuticle of elytra pitch-black with faint blueish hue, pronotum lighter, dark brown with faint bronze hue; legs, mouthparts and antennae castaneous.

**Figures 733–741.**
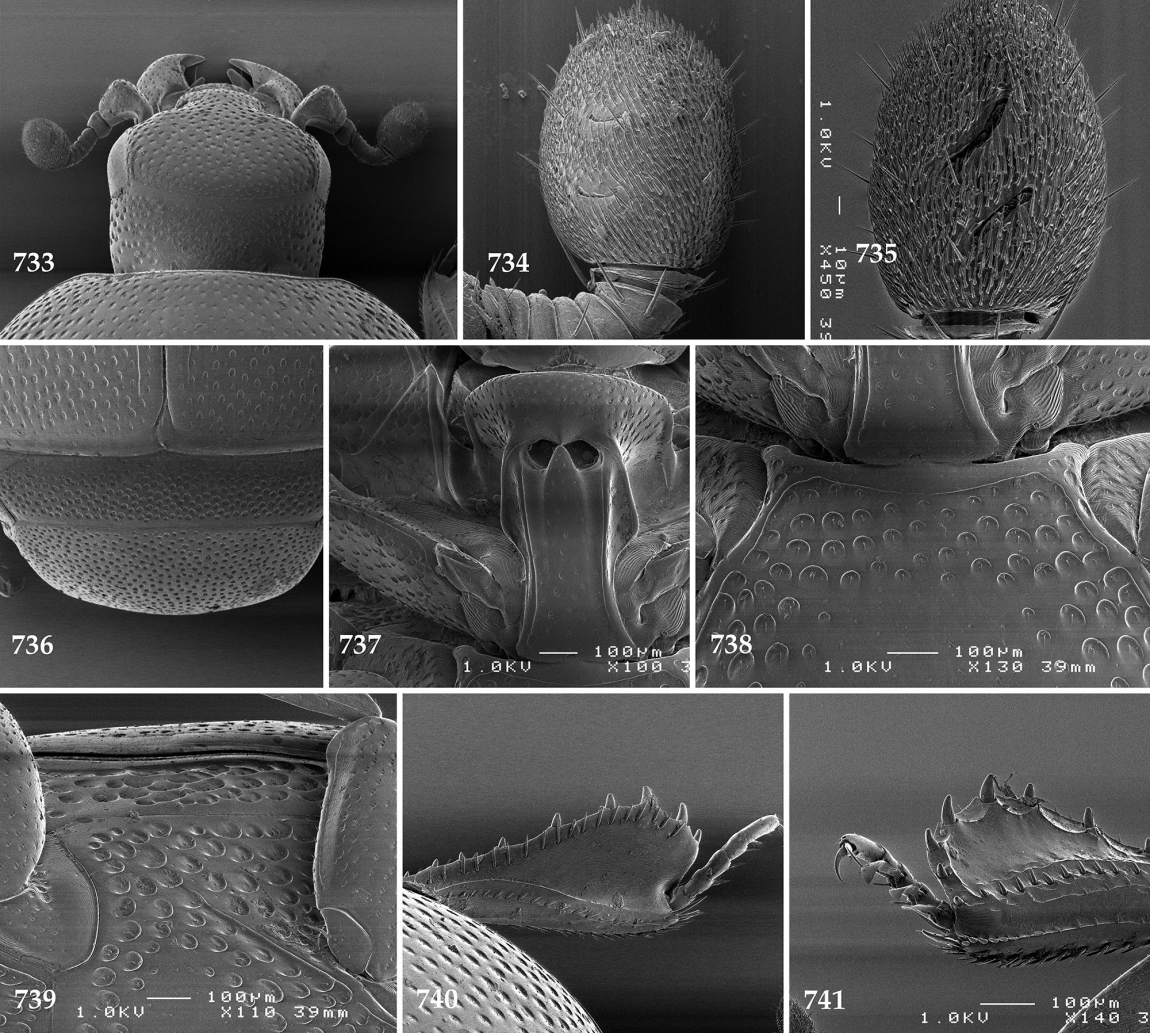
**733** *Tomogenius ripicola* (Marseul, 1870) head, dorsal view **734** antennal club, dorsal view **735** ditto, ventral view **736** propygidium + pygidium **737** prosternum **738** mesoventrite **739** lateral disc of metaventrite + metepisternum **740** protibia, dorsal view **741** ditto, ventral view.

Antennal scape (Fig. 733) not particularly thickened, with few microscopic setae; club (Fig. 734) rather large, oval, dorsally with two horizontal slit-like pits, ventrally (Fig. 735) with two vertical slit-like pits, entirely covered in dense short sensilla, intermingled with sparse longer erect sensilla; sensory structures of antennal club apart from sensory slit-like pits present dorsally and ventrally not examined.

Mandibles (Fig. 732) punctate, with rounded outer margin, acutely pointed, sub-apical tooth on inner margin of left mandible obtuse; labrum even, convex, punctate, approximately twice as wide as long, labral pits each with single long labral seta; terminal labial palpomere elongated, its width about one-fourth its length, pointed apically; mentum sub-trapezoid, anterior angles slightly produced, anterior margin with a shallow emargination medially, surface around it with two long setae from each side, lateral margins with a single one row of much shorter sparse ramose setae, disc with few scattered setae; terminal maxillary palpomere narrowing on apical third, apex pointed, slender, its width about one-fourth its length; rest of the mouthparts not examined.

Clypeus (Fig. 732) even, rounded laterally, punctate; frontal and supraorbital striae absent, occipital stria present, fine; frontal disc (Fig. 732) punctate, punctures deep, round and most dense medially, separated by their own to several times their diameter; eyes convex, well visible from above.

Pronotal sides (Fig. 731) convergent anteriorly, apical angles obtuse, marginal pronotal stria complete, thin, slightly carinate, somewhat weakened behind head; disc entirely punctate, medially punctures separated by several times their diameter, fine, becoming coarser and denser laterally, especially near apical angles where they are deep, ellipsoid and separated by less than their own diameter; pronotal hypomeron glabrous; occasionally small ante-scutellar fovea present; scutellum very small.

Elytral epipleura punctate; marginal epipleural stria double, one stria weakly impressed, while the second one deeply impressed and in punctures, both striae complete; marginal elytral stria, deeply impressed and in punctures, continuous along elytral apex as weakened apical elytral stria not attaining the apical end of sutural elytral one; humeral elytral stria deeply impressed on basal third, surface around it with minute strioles; outer (?) subhumeral stria present as a short median fragment; elytral disc with four dorsal elytral striae 1–4, all striae in fine punctures, stria 1 and 2 surpassing elytral half apically, striae 3 and 4 shorter, reaching approximately elytral half apically; basal ends of all striae curved; between fourth dorsal elytral and sutural striae a characteristic hooked appendix present, basally occasionally connected to basal fragment of sutural elytral stria; sutural elytral stria apart from this basal fragment interrupted on basal third, continued apical two-thirds (roughly), becoming weaker apically. Entire elytral disc punctate, but punctures on basal elytral third (roughly) very fine and sparse, separated several times their diameter, on apical two-thirds (roughly) punctures larger and denser, separated approximately by their diameter, becoming almost confluent apically; punctures near extreme elytral apex with minuscule striolae among them.

Propygidium (Fig. 736) transverse, about four times as broad as long, completely exposed, with very dense but small punctures separated by less than their diameter; pygidium (Fig. 736) with sparser round punctures, separated by about twice their diameter, becoming sparser and finer towards apex.

Anterior margin of median portion of prosternum (Fig. 737) rounded; marginal prosternal stria absent; prosternal process broad, flattened, dorso-laterally with punctures, apex of prosternal keel considerably elevated with respect to the rest of the surface; carinal prosternal striae (Fig. 737) sub-parallel, terminating near large and deep apical foveae separated by apex of prosternal prosternal process; lateral prosternal striae carinate, slightly convergent anteriorly, attaining apices of carinal prosternal striae.

Discal marginal mesoventral stria (Fig. 738) well impressed, somewhat carinate, slightly inwardly arcuate medially; disc flattened, with approximately 40–50 round deep punctures separated by about less than their diameter; meso-metaventral suture distinct, straight; meso-metaventral sutural stria absent.

Intercoxal disc of metaventrite in male medially with shallow depression, in female slightly convex; disc of metaventrite medially with very fine scattered punctures, laterally and basally (especially in the area behind metacoxa) punctures becoming coarser and denser. Lateral metaventral stria well impressed, carinate, straight, shortened apically; lateral disc of metaventrite (Fig. 739) flattened, with round shallow large punctures; metepisternum + fused metepimeron (Fig. 739) evenly covered with much coarser, deeper and denser punctation; lateral metepisternal stria present, deeply impressed and almost complete.

Intercoxal disc of first abdominal ventrite flattened, completely striate laterally; surface of disc with scattered oblong punctation, punctures becoming sparser and finer medially.

Protibia (Fig. 740) flattened and dilated, outer margin without visible teeth, with approximately nine short denticles growing out directly from outer protibial margin and becoming progressively smaller in proximal direction; setae of outer row short, moderately dense, regular; setae of median row similarly dense and regular, but shorter than those of outer row; protarsal groove shallow; anterior protibial stria complete, costate; two thin, short tarsal denticles present apically; protibial spur short and thin, straight, growing out from apical protibial margin from near tarsal insertion; apical margin of protibia posteriorly with two-three tiny apical denticles; outer part of posterior surface of protibia (Fig. 741) glabrous, with a row of short setae; median part of posterior surface with an additional row of minuscule setae; posterior protibial stria complete, with scattered minuscule setae turning into a row of tightly-spaced short setae near apical margin; inner margin with double row of dense short setae.

Mesotibia slender, outer margin with a single row of dense thin denticles growing in size apically; setae of outer row sparse, fine, regular and short, growing somewhat longer apically; setae of median row irregular, much shorter than those of outer row; posterior mesotibial stria complete; anterior surface of mesotibia with a dense row of well sclerotized short denticles, with another similar row of much shorter and finer setae situated below it; anterior mesotibial stria complete, terminating in several tiny inner anterior denticles; mesotibial spur stout, short; apical margin with two tiny denticles; mesotarsus shorter than mesotibia; claws of apical tarsomere about half its length; metatibia more slender and longer than mesotibia, three-four short denticles present on outer margin and three longer denticles present near metatibial apex; anterior face of metatibia with a single row of dense short and regular denticles; claws of apical tarsomere in both meso- and metatibia bent, shorter than half its length.

**Figures 742–750.**
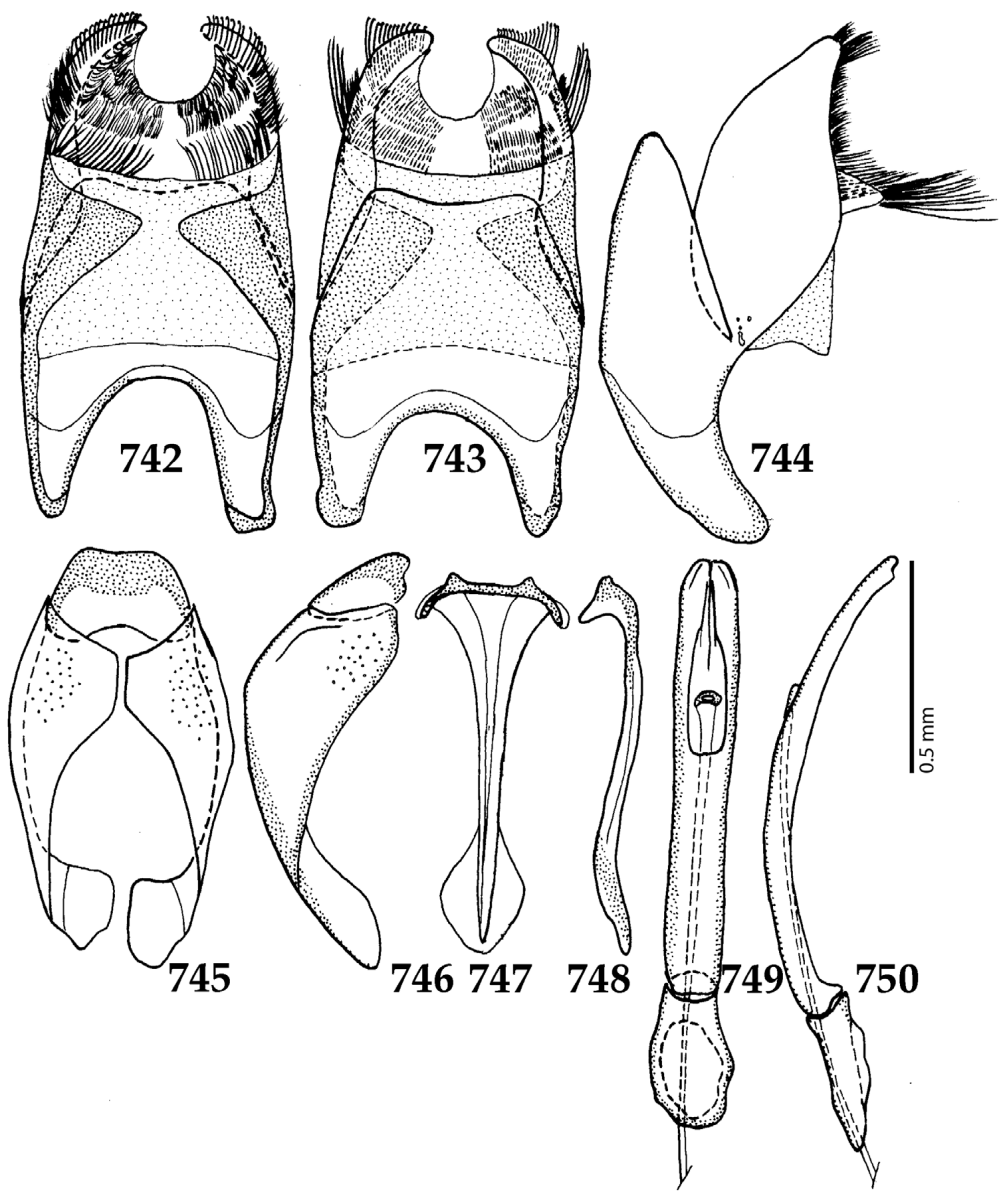
**742** *Tomogenius ripicola* (Marseul, 1870) male terminalia: 8^th^ sternite + 8^th^ tergite, ventral view **743** ditto, dorsal view **744** ditto, lateral view **745** male terminalia: 9^th^ + 10^th^ tergites, dorsal view **746** ditto, lateral view **747** male terminalia: spiculum gastrale, ventral view **748** ditto, lateral view **749** male terminalia: aedeagus, dorsal view **750** ditto, lateral view.

Male genitalia. Eighth sternite (Figs 742–743) fused on its entire length, but its sclerotization widely separated medially; with large velum bearing laterally a dense tuft of long setae (best observable from lateral view, Fig. 744), apex of 8^th^ sternite with a dense row of shorter hooked setae; rest of velum with several dense rows of setae becoming progressively shorter apically; eighth tergite apically slightly inwardly arcuate, covered with numerous pseudo-pores; eighth tergite and eighth sternite fused laterally (Fig. 744). Ninth tergite (Figs 745–746) longitudinally divided medially, with pores and pseudopores; tenth tergite rather small, inwardly arcuate basally, apically slightly inwardly arcuate; spiculum gastrale (Figs 747–748) gradually dilated from middle towards its apical end that is strongly sclerotized, with two short ‘horns’; basal end slightly dilated, spoon-like, outwardly arcuate. Aedeagus (Figs 749–750) slender, parallel-sided, parameres not fused on their apical third, apex of aedeagus blunt; basal piece of aedeagus short, ratio of its length : length of parameres 1 : 3; aedeagus curved from lateral view (Fig. 750).

## Biogeographic synthesis and discussion

As mentioned in the introduction, with only 40 species, several of which are introduced, the Saprininae fauna of Australopacific Region is remarkably poor when compared to other parts of the world (see Lackner 2014d) for details. We shall attempt here a biogeographic synthesis of all Saprininae fauna of Australopacific Region based on the available information, treating each ‘area’ separately, and focusing mainly on the species that are autochthonous to the region. For the exact distribution of taxa in each area the reader is referred to the table below (Table 1).

**Table 1.** Distribution and ecologies of the Australopacific Saprininae.

	**Taxon**	“**Area**”					**Biology**
		**New Guinea**	**New Caledonia**	**Australia**	**New Zealand**	**Pacific Region**	
1.	*Australopachylopus lepidulus*	–	–	–	x	–	Littoral psammophile
2.	*Chalcionellus aeneovirens*	–	–	x	–	–	Inroduced; saprobiont
3.	Eusp. (Neosaprinus) rubriculus	–	–	x	x	–	Inroduced; saprobiont?
4.	*Gnathoncus communis*	–	–	–	x	–	Introduced; inquiline
5.	*Gnathoncus rotundatus*	–	–	x	x	–	Introduced; inquiline
6.	Hypocacculus (H.) hyla	x	–	–	–	–	Saprobiont
7.	H. (Nessus) interpunctatus	–	–	x	–	–	Introduced; saprobiont
8.	Hypocaccus (H.) brasiliensis	x	x	x	–	–	Littoral psammophile
9.	Hypocaccus (H.) sinae	–	–	x	–	–	Littoral psammophile
10.	H. (Baeckmanniolus) varians	–	–	x	–	–	Littoral psammophile
11.	*Iridoprinus myrmecophilus*	–	–	x	–	–	Myrmecophile
12.	*Notosaprinus irinus*	–	–	x	–	–	Saprobiont
13.	*Reichardtia pedator*	–	–	–	x	–	Littoral psammophile
14.	*Saprinodes distinctus*	–	–	x	–	–	Unknown
15.	*Saprinodes falcifer*	–	–	x	–	–	Unknown
16.	Saprinus (S.) amethystinus	–	–	x	–	–	Unknown
17.	Saprinus (S.) artensis	–	x	–	–	–	Saprobiont ?
18.	Saprinus (S.) australis	–	–	x	–	–	Saprobiont
19.	Saprinus (S.) chalcites	–	–	x	–	–	Introduced; saprobiont
20.	Saprinus (S.) chathamensis	–	–	–	x	–	Saprobiont
21.	Saprinus (S.) cupreus	–	–	x	–	–	Introduced; saprobiont
22.	Saprinus (S.) cyaneus cyaneus	x	x	x	–	–	Saprobiont
23.	Saprinus (S.) detritus	–	–	–	x	–	Saprobiont
24.	Saprinus (S.) grandiclava	x	–	–	–	–	Saprobiont
25.	Saprinus (S.) laetus	–	–	x	–	–	Saprobiont
26.	Saprinus (S.) nitiduloides	x	–	–	–	–	Saprobiont
27.	Saprinus (S.) pacificus	–	–	–	–	x	Saprobiont
28.	Saprinus (S.) pseudodetritus	–	–	–	x	–	Saprobiont
29.	Saprinus (S.) rarus	–	–	x	–	–	Termitophile
30.	Saprinus (S.) splendens	x	–	x	–	–	Saprobiont
31.	Saprinus (S.) tyrrhenus	–	–	x	–	–	Saprobiont?
32.	Saprinus (S.) viridanus	–	–	x	–	–	Saprobiont
33.	Saprinus (S.) viridipennis	–	–	x	–	–	Saprobiont
34.	*Tomogenius australis*	–	–	–	x	–	Bird inquiline
35.	*Tomogenius incisus*	–	–	x	–	–	Troglophile
36.	*Tomogenius kuscheli*	–	–	–	x	–	Bird inquiline
37.	*Tomogenius latipes*	–	–	–	x	–	Bird inquiline
38.	*Tomogenius motocola*	–	–	x	–	–	Troglophile
39.	*Tomogenius papuaensis*	x	–	–	–	–	Troglophile
40.	*Tomogenius ripicola*	–	–	x	–	–	Troglophile, saprobiont

1) The island of New Guinea together with all surrounding islands (but without Moluccas), Aru Islands, New Britain, New Ireland as well as the Solomon Islands: this ‘area’ contains 7 native species. The majority of these (4) are typical saprobionts and members of the genus *Saprinus*: *S. cyaneus cyaneus* and *S. splendens* are most likely ‘northern invaders’ *sensu* Matthews and Bouchard (2008: 17) as they occur also in the Indo-Malayan subregion, are tropical and could have arrived into the region by ‘island-hopping’ from north, while the other two are endemic (*S. nitiduloides*; *S. grandiclava*). There are three additional Saprininae species occurring in this ‘area’: Hypocacculus (H.) hyla that is likewise a typical saprobiont spread also in the Indo-Malayan subregion and likely also a ‘northern invader’; Hypocaccus (H.) brasiliensis (a littoral psammophile with wide tropicopolitan distribution) and an endemic cavernicolous member of the typical Australopacific lineage, *Tomogenius papuensis*.

2) The island of New Caledonia, together with Vanuatu, Fiji, Tuvalu, Wallis and Futuna, American Samoa, Tokelau, Tonga, the Cook Islands, French Polynesia and Niue: this ‘area’ contains only three native species, two of which belong to *Saprinus*. One is the aforementioned *S. cyaneus cyaneus*, another one the New Caledonian endemic *S. artensis*. *S. artensis*, based on the general habitus and male genitalia, most likely shares a recent common ancestor with *S. cyaneus cyaneus* or is its derivate.

3) The continent of Australia with Lord Howe Island, Tasmania with surrounding islands, Kangaroo Island, Tiwi Islands and numerous small islands around Australia’s coasts. This, by size undoubtedly the largest ‘area’ contains the largest number of native species (19), plus 6 introduced species. Genus *Saprinus*, with 9 autochthonous and 2 adventive species, is the most species-rich here, which corresponds to its global distribution (with 157, worldwide distributed species *Saprinus* is the most species-rich genus of the subfamily). Autochthonous Australian *Saprinus* members can be further divided into: a) ‘northern invaders’ (*S. splendens* and *S. cyaneus cyaneus*, see also above with the New Guinea ‘area’); and b) endemic species of unknown origin. Among the endemic taxa, there are two subgroups, based on the genitalic morphology of aedeagus, created here for the practical reasons only: a) ‘separate parameres’ group (*S. amethystinus*, *S. australis*, *S. laetus* and *S. tyrrhenus*); and b) ‘fused parameres’ group (*S. rarus*, *S. viridanus* and *S. viridipennis*). Without having performed phylogenetic analysis of the genus, it would be premature to speculate on the relationships between the two subgroups or members within each group. However, the plesiomorphic aedeagal state of *Saprinus* (see also above) does not seem to be the ‘separated parameres’ state; this condition probably evolved separately and does not necessarily have a common origin. A systematic revision of the genus *Saprinus* is badly needed. All Australian species of *Saprinus*, as far as known, are typical free-living volant saprobiont predators, although the biology of two of them (*S. amethystinus*, *S. tyrrhenus*) is, due to their scarcity, completely unknown. *S. rarus* is unique among the Australian Saprininae since it apparently lives in the nests of the common arboreal termite *Nasutitermes walkeri*.

Another Australian endemic, the monotypic *Notosaprinus* is related to *Saprinus* (see Lackner 2014d) and often found together with various *Saprinus* species on carrion or in pitfall traps.

Australia houses three native species of *Hypocaccus* (H. (H.) sinae, H. (H.) brasiliensis and H. (B.) varians)), all are littoral psammophile species that arrived from the north (where they likewise occur) most likely by island-hopping. Members of the genus *Hypocaccus* are spread mainly on the beaches or riverbanks of warmer parts of the world, with the exception of most of South America and New Zealand.

As outlined in the table below (Table 1), there are three species of the Australopacific endemic genus *Tomogenius* in Australia: *T. incisus*, *T. motocola* and *T. ripicola*. While the latter is often found on carrion as well as in caves, the former two are chiefly found in caves where they feed on fly larvae developing in bat guano. *Tomogenius* is undoubtedly a primitive taxon having morphological plesiomorphic character states *sensu* Lackner (2014d); see also above.

We consider among the most noteworthy discoveries of our work the presence of the first truly myrmecophilous saprinine taxon (monotypic Australian *Iridoprinus*) and the first true Australopacific termitophilous saprinine (Australian *Saprinus rarus*). Although there are several Saprininae taxa around the world that invaded the nests of ants (Palaearctic *Myrmetes* Marseul, 1862, Neotropical *Paramyrmetes* Bruch, 1929; see also Lackner 2017 for details) or termites (Afrotropical *Pilisaprinus* Kanaar, 1996 and *Nannolepidius* Reichardt, 1932; see also Lackner 2013b for details), none were known from the Australopacific Region thus far.

The one last remaining enigma is the life mode of the very rare and extremely peculiar (morphologically and phylogenetically speaking) Australian endemic *Saprinodes*, with two described species. Its life history is completely unknown (most species were collected in pitfall traps) and obtaining freshly collected DNA-grade specimens would be very beneficial. *Saprinodes* was recovered (Lackner 2014d) sister to another Australian endemic (*Iridoprinus*); this clade was sister (albeit with weak support) to a Neotropical attaphilic Euspilotus (Platysaprinus) latimanus (see also Remarks section of *Saprinodes* for details).

4) New Zealand with the Chatham Islands and other tiny surrounding islands as well as subantarctic islands: this ‘area’ contains 8 native endemic species, as well as 3 introduced ones. Three of the autochthonous taxa again belong to the widely spread and species-rich saprobiont genus *Saprinus*, with a single species *S. detritus* present on the NZ mainland as well as the Chatham Islands and further two newly-described species (*S. chathamensis* and *S. pseudodetritus*) found on the Chatham Islands only. Observing the male genitalic characters of the three species in question and comparing them to the rest of the species from Australopacific Region one can conclude that the NZ species are not similar to any other species from the region, and the male genitalic characters, especially the apically separated parameres of the aedeagus are rather unique, if slightly similar to the New Guinean *S. grandiclava*.

On the NZ mainland there are three species of the typical Australopacific endemic genus *Tomogenius* present: *T. australis*, *T. kuscheli* and *T. latipes*. It is interesting to remark, that while their relatives in the region, the New Guinean *T. papuaensis* as well as the Australian *T. incisus*, *T. motocola* and (for a part) also *T. ripicola*, are mostly cavernicolous (but *T. incisus* was also collected in open forest and many specimens of *T. ripicola* are found on carrion in the open landscape), the NZ species are mostly inquilines of birds. While *T. australis* and *T. kuscheli* are known exclusively from bird’s nests, the most commonly collected species, *T. latipes* was also collected on moss and lichens, on tree trunks at night and in pitfall traps.

New Zealand hosts two further native littoral psammophilic taxa: the endemic and monotypic genera *Reichardtia* and *Australopachylopus*. Their origin is obscure and they were both in the most recent analysis (Lackner 2014d) members of a weakly supported large clade of mostly xerophilic, psammophilous taxa with unresolved relationships.

5) The Pacific Region of Löbl and Löbl (2015): this ‘area’ contains a single native species, the newly described *S. pacificus*, which is, based on its male genitalia, either a derivate of *S. cyaneus cyaneus* or shares with it a recent common ancestor.

The 7 non-native species form an artificial group in that they did not arrive to Australopacific Region naturally, but rather through human transport. Here belong: species of the genus *Gnathoncus*, *Chalcionellus aeneovirens*, Euspilotus (Neosaprinus) rubriculus, Hypocaccus (Nessus) interpunctatus interpunctatus and two species of *Saprinus* (*S. cupreus*, *S. chalcites*). Based on the origin of the adventive species, the observable patterns here are: two or even three of them could have come from Africa (*C. aeneovirens*, S. (S.) cupreus, H. (N.) interpunctatus interpunctatus; although this last-mentioned species has also been recorded from the Palaeacrctic Region); one (E. (N.) rubriculus) originates from South America; two members of *Gnathoncus* are normally found in the Palaearctic Region; and, finally, Saprinus (S.) chalcites is spread across the entire Mediterranean, the Arab Peninsula, Africa, and warmer parts of Asia (Mazur 2011).

As can be thus extrapolated from the above text, the native Saprininae fauna of Australopacific Region is probably a mixture of northern invaders that would have arrived to the region in early Cenozoic by ‘island hopping’ from north (*Hypocaccus*, Hypocacculus (H.) hyla, several *Saprinus*) and truly autochthonous taxa (*Iridoprinus*, *Saprinodes*, *Reichardtia*, *Australopachylopus*, *Notosaprinus*, most species of *Saprinus*, and *Tomogenius*).

This work is a result of approximately nine years of study of the Saprininae taxa from the Australopacific Region based on thousands of examined specimens from museums from all over the world. It treats a complex fauna that, except for a few cases (e.g. Dahlgren 1976) has seen almost no previous attention. It is hoped that it will become a standard reference in the area. Despite all the effort, however, we cannot be absolutely sure that there was not something overlooked and the number of species of the region can (and is expected to) eventually increase; we likewise cannot exclude further synonymies based on molecular characters. We hope, however, that our paper shall serve as a good guide for all pertinent future studies.

**Figure 751.**
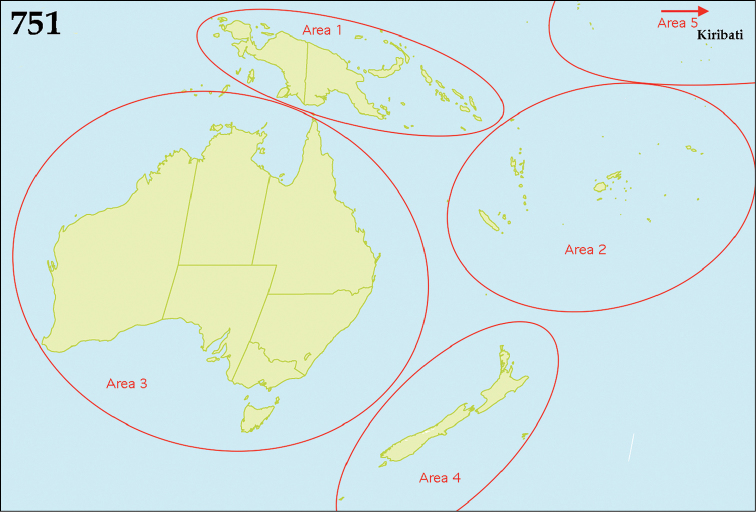
Australopacific Region with depicted ‘areas’.

**Figure 752.**
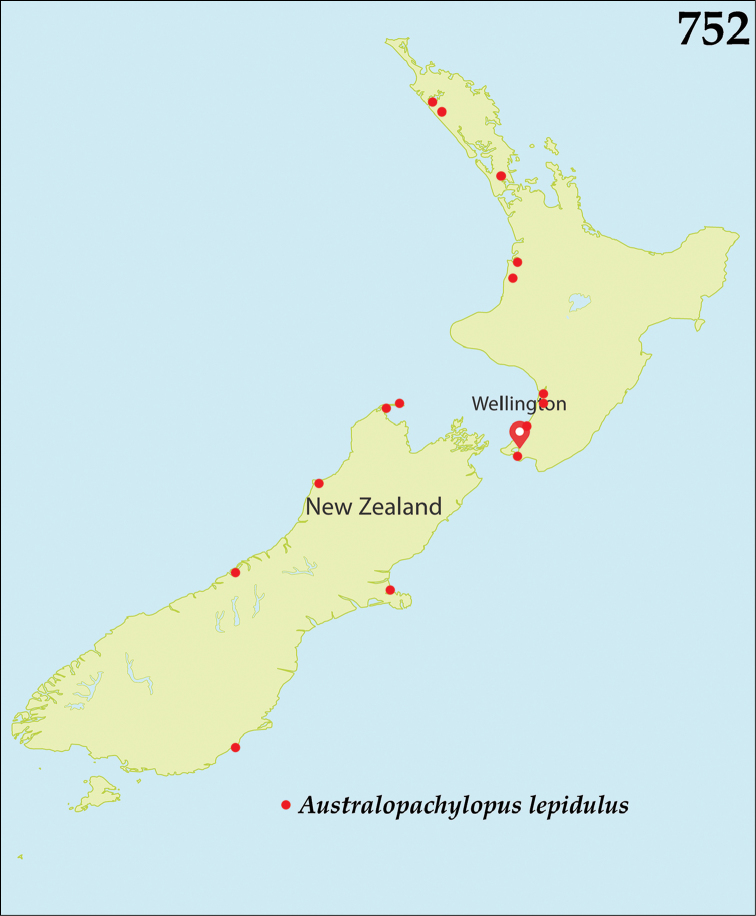
Distribution of *Australopachylopus lepidulus* (Broun, 1881) in New Zealand.

**Figure 753.**
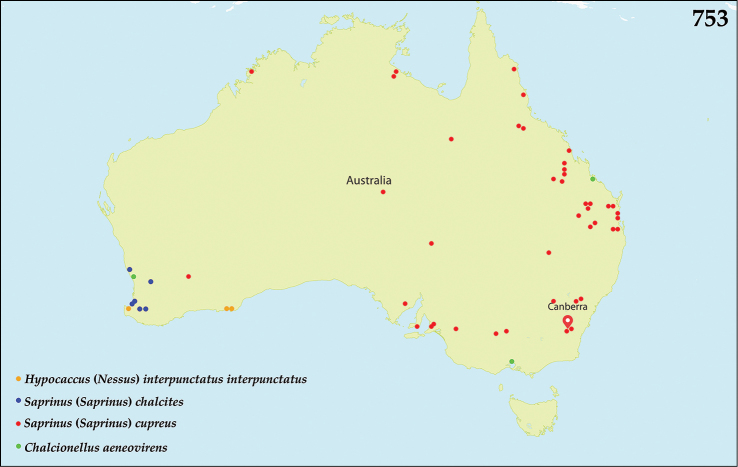
Distribution of four introduced Saprininae in Australia: *Chalcionellus aeneovirens* (Schmidt, 1890); Hypocaccus (Nessus) interpunctatus interpunctatus (Schmidt, 1885); Saprinus (Saprinus) cupreus Erichson, 1834 and Saprinus (Saprinus) chalcites (Illiger, 1807).

**Figure 754.**
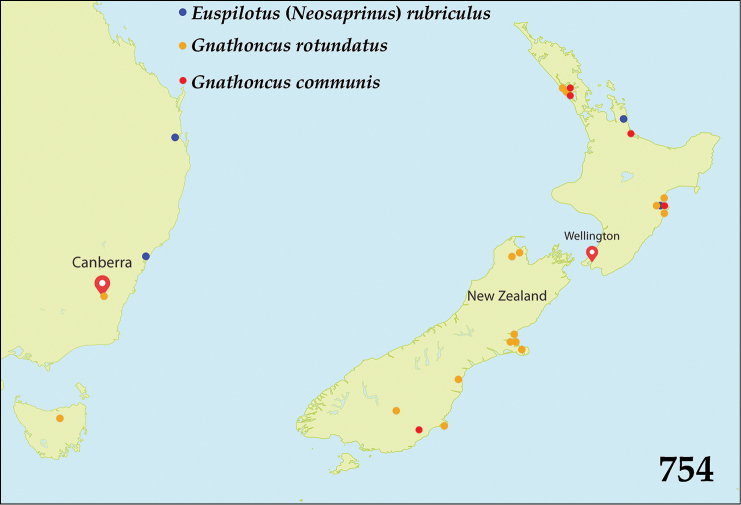
Distribution of Euspilotus (Neosaprinus) rubriculus (Marseul, 1855) in New Zealand and Australia; *Gnathoncus communis* (Marseul, 1862) in New Zealand and *Gnathoncus rotundatus* (Kugelann, 1792) in Australia and New Zealand.

**Figure 755.**
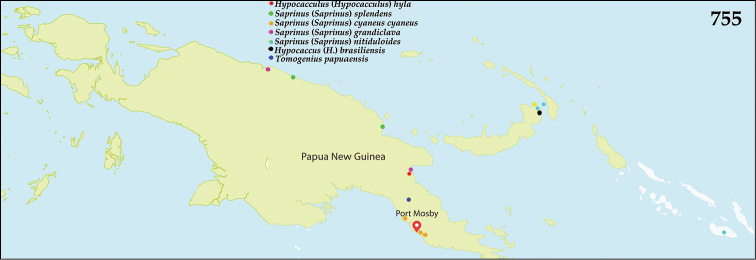
Distribution of Saprininae in New Guinea and Solomon Islands: Hypocacculus (Hypocacculus) hyla (Marseul, 1864) (New Guinea); Hypocaccus (Hypocaccus) brasiliensis (Paykull, 1811) (New Guinea: New Britain); Saprinus (Saprinus) cyaneus cyaneus (Fabricius, 1775) (New Guinea); Saprinus (Saprinus) grandiclava Kanaar, 1989 (New Guinea); Saprinus (Saprinus) nitiduloides Fairmaire, 1883 (New Guinea: New Britain; Solomon Islands); Saprinus (Saprinus) splendens (Paykull, 1811) (New Guinea) and *Tomogenius papuaensis* Gomy, 2007 (New Guinea).

**Figure 756.**
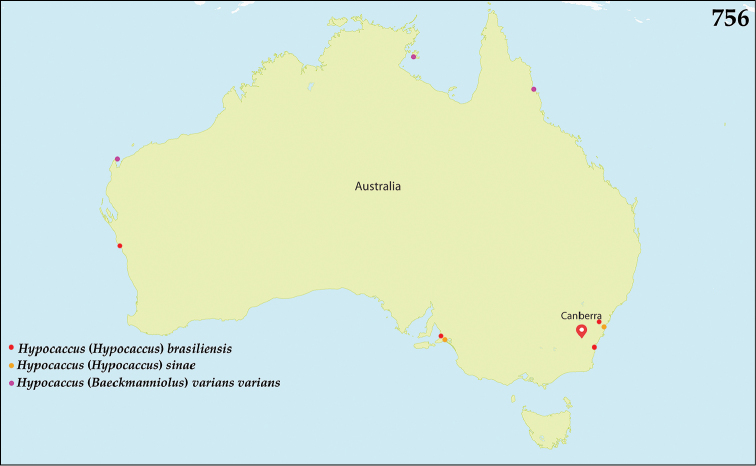
Distribution of *Hypocaccus* species in Australia: Hypocaccus (Hypocaccus) brasiliensis (Paykull, 1811); Hypocaccus (Hypocaccus) sinae (Marseul, 1862) and Hypocaccus (Baeckmanniolus) varians varians (Schmidt, 1890).

**Figure 757.**
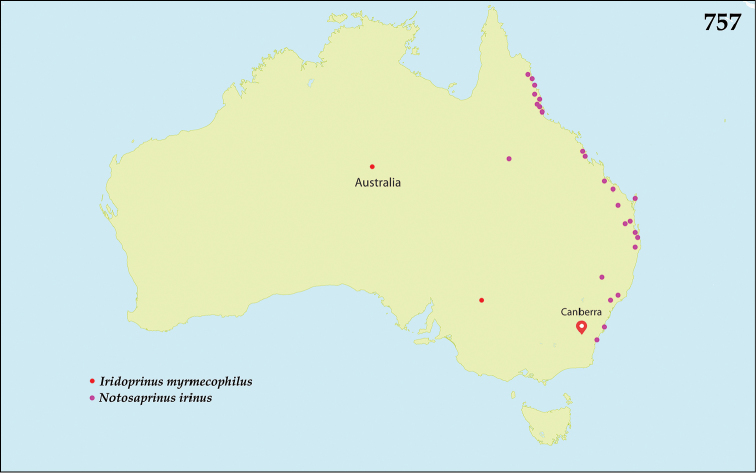
Distribution of *Iridoprinus myrmecophilus* gen. & sp. n. and *Notosaprinus irinus* (Marseul, 1862) in Australia.

**Figure 758.**
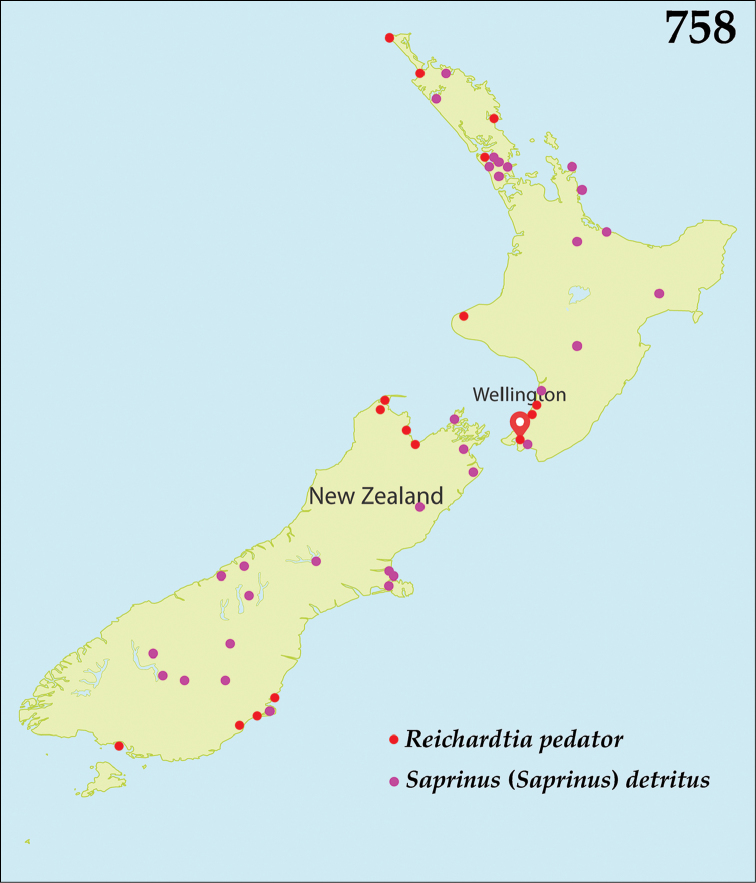
Distribution of *Reichardtia pedator* (Sharp, 1876) and Saprinus (Saprinus) detritus (Fabricius, 1775) in mainland New Zeland.

**Figure 759.**
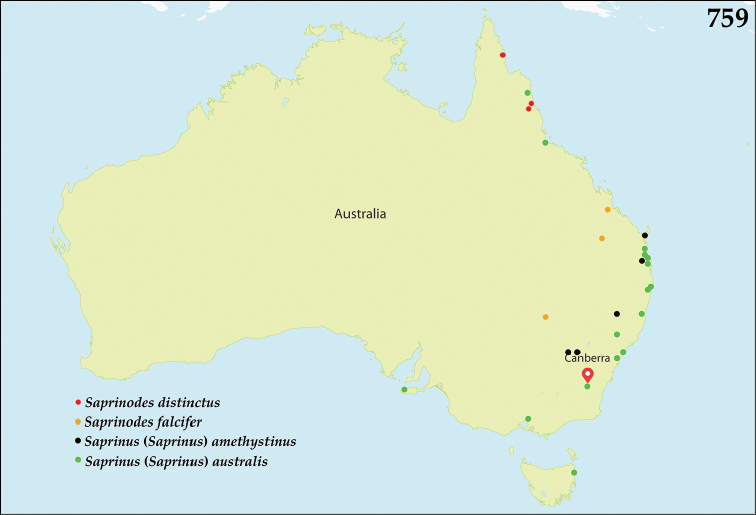
Distribution of *Saprinodes distinctus* Dégallier, 1993; *Saprinodes falcifer* Lewis, 1891; Saprinus (Saprinus) amethystinus Lewis, 1900 and Saprinus (Saprinus) australis (Boisduval, 1835) in Australia.

**Figure 760.**
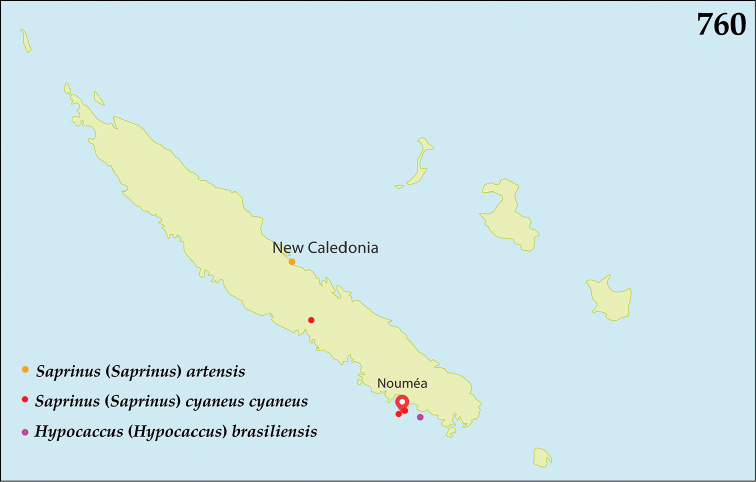
Distribution of Saprininae on New Caledonia: Saprinus (Saprinus) cyaneus cyaneus (Fabricius, 1775); Saprinus (Saprinus) artensis Marseul, 1862 and Hypocaccus (Hypocaccus) brasiliensis (Paykull, 1811).

**Figure 761.**
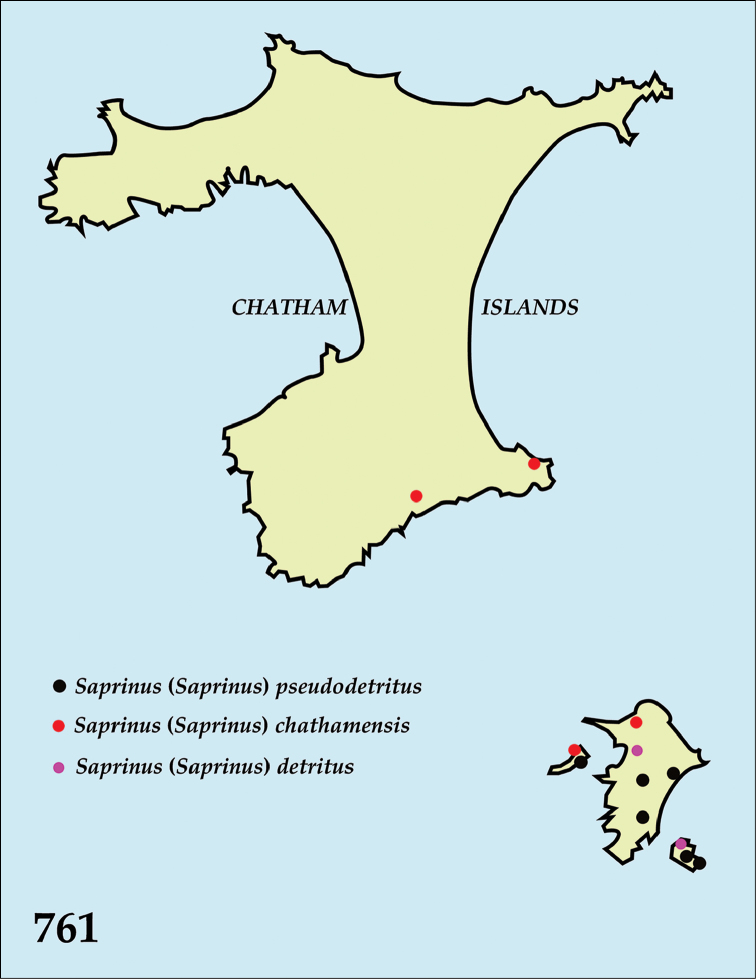
Distribution of the genus *Saprinus* Erichson, 1834 on the Chatham Islands: Saprinus (Saprinus) chathamensis sp. n.; Saprinus (Saprinus) detritus (Fabricius, 1775) and Saprinus (Saprinus) pseudodetritus sp. n.

**Figure 762.**
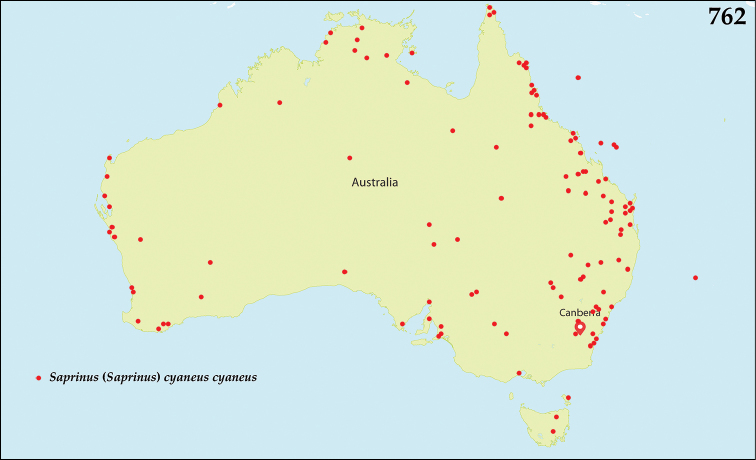
Distribution of Saprinus (Saprinus) cyaneus cyaneus (Fabricius, 1775) in Australia.

**Figure 763.**
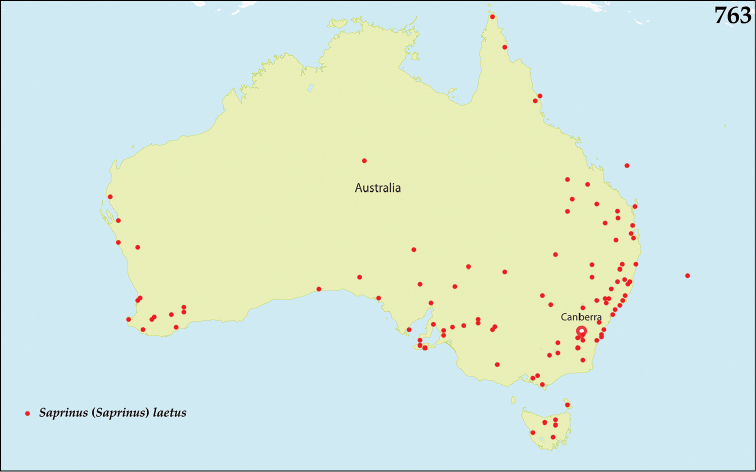
Distribution of Saprinus (Saprinus) laetus Erichson, 1834 in Australia.

**Figure 764.**
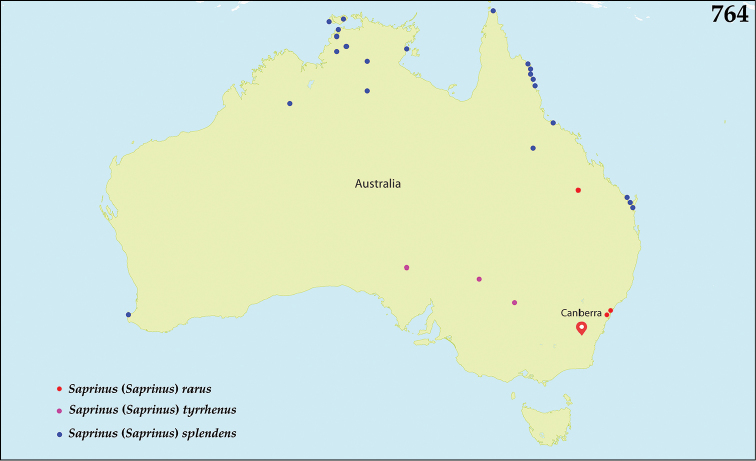
Distribution of Saprinus (Saprinus) rarus sp. n., Saprinus (Saprinus) splendens (Paykull, 1811) and Saprinus (Saprinus) tyrrhenus Blackburn, 1903 in Australia.

**Figure 765.**
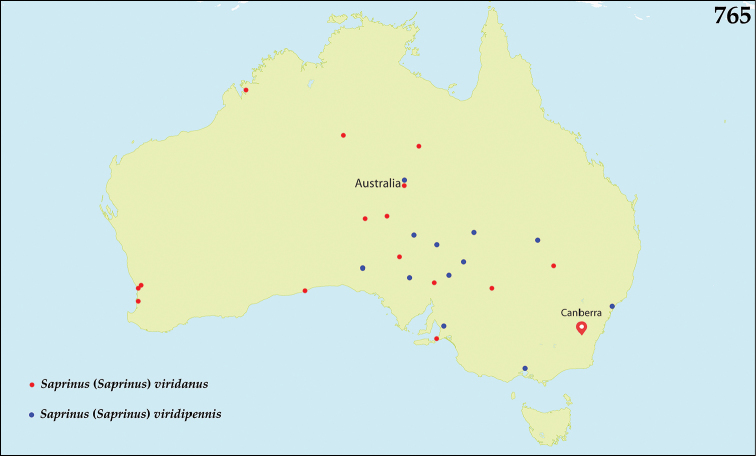
Distribution of Saprinus (Saprinus) viridanus Lewis, 1899 and Saprinus (Saprinus) viridipennis Lewis, 1901 in Australia.

**Figure 766.**
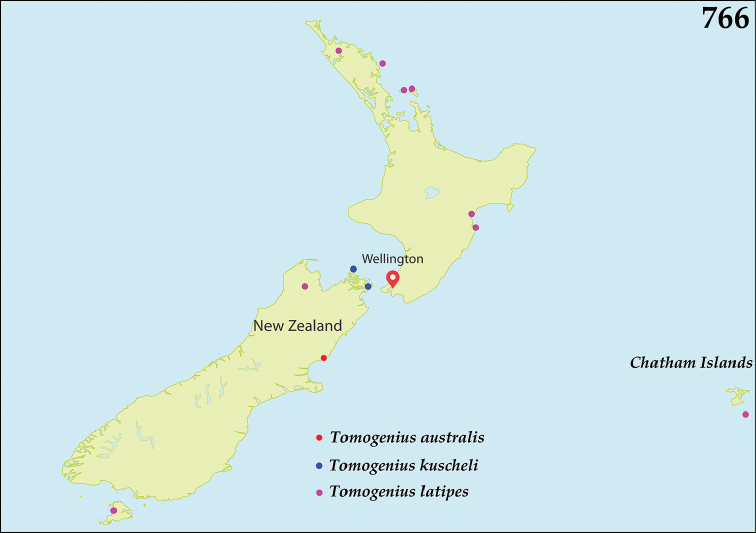
Distribution of the genus *Tomogenius* Marseul, 1862 in New Zeland: *Tomogenius australis* Dahlgren, 1976; *Tomogenius kuscheli* Dahlgren, 1976 and *Tomogenius latipes* (Broun, 1881).

**Figure 767.**
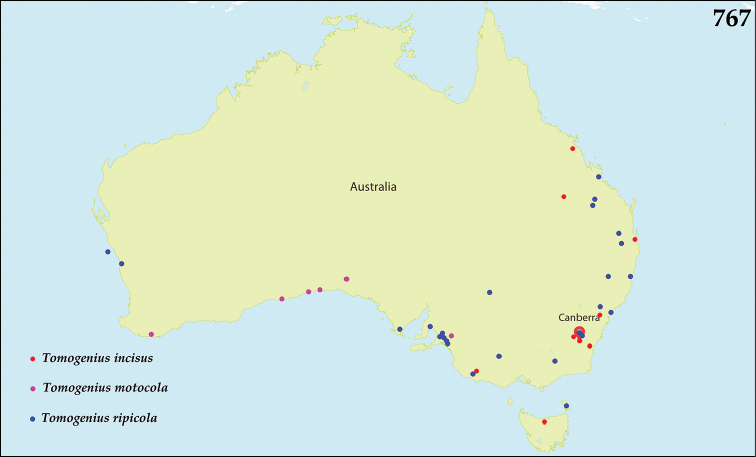
Distribution of the genus *Tomogenius* Marseul, 1862 in Australia: *Tomogenius incisus* (Erichson, 1842); *Tomogenius motocola* Mazur, 1990 and *Tomogenius ripicola* (Marseul, 1870).

## Supplementary Material

XML Treatment for Australopachylopus

XML Treatment for Australopachylopus lepidulus

XML Treatment for Chalcionellus

XML Treatment for Chalcionellus aeneovirens

XML Treatment for Euspilotus

XML Treatment for Neosaprinus

XML Treatment for Euspilotus (Neosaprinus) rubriculus

XML Treatment for Gnathoncus

XML Treatment for Gnathoncus communis

XML Treatment for Gnathoncus rotundatus

XML Treatment for Hypocacculus

XML Treatment for Hypocacculus

XML Treatment for Hypocacculus (Hypocacculus) hyla

XML Treatment for Hypocaccus

XML Treatment for Nessus

XML Treatment for Hypocaccus (Nessus) interpunctatusinterpunctatus

XML Treatment for Hypocaccus

XML Treatment for Hypocaccus (Hypocaccus) brasiliensis

XML Treatment for Hypocaccus (Hypocaccus) sinae

XML Treatment for Baeckmanniolus

XML Treatment for Hypocaccus (Baeckmanniolus) variansvarians

XML Treatment for Iridoprinus

XML Treatment for Iridoprinus myrmecophilus

XML Treatment for Notosaprinus

XML Treatment for Notosaprinus irinus

XML Treatment for Reichardtia

XML Treatment for Reichardtia pedator

XML Treatment for Saprinodes

XML Treatment for Saprinodes distinctus

XML Treatment for Saprinodes falcifer

XML Treatment for Saprinus

XML Treatment for Saprinus (Saprinus) amethystinus

XML Treatment for Saprinus (Saprinus) artensis

XML Treatment for Saprinus (Saprinus) australis

XML Treatment for Saprinus (Saprinus) chalcites

XML Treatment for Saprinus (Saprinus) chathamensis

XML Treatment for Saprinus (Saprinus) cupreus

XML Treatment for Saprinus (Saprinus) cyaneuscyaneus

XML Treatment for Saprinus (Saprinus) detritus

XML Treatment for Saprinus (Saprinus) grandiclava

XML Treatment for Saprinus (Saprinus) laetus

XML Treatment for Saprinus (Saprinus) nitiduloides

XML Treatment for Saprinus (Saprinus) pacificus

XML Treatment for Saprinus (Saprinus) pseudodetritus

XML Treatment for Saprinus (Saprinus) rarus

XML Treatment for Saprinus (Saprinus) splendens

XML Treatment for Saprinus (Saprinus) tyrrhenus

XML Treatment for Saprinus (Saprinus) viridanus

XML Treatment for Saprinus (Saprinus) viridipennis

XML Treatment for Saprinus lindrothi

XML Treatment for Tomogenius

XML Treatment for Tomogenius australis

XML Treatment for Tomogenius incisus

XML Treatment for Tomogenius kuscheli

XML Treatment for Tomogenius latipes

XML Treatment for Tomogenius motocola

XML Treatment for Tomogenius papuaensis

XML Treatment for Tomogenius ripicola
